# Phylogeography and colonization pattern of subendemic round-leaved oxeye daisy from the Dinarides to the Carpathians

**DOI:** 10.1038/s41598-022-19619-1

**Published:** 2022-09-30

**Authors:** Kamil Konowalik

**Affiliations:** Department of Botany and Plant Ecology, Wrocław University of Environmental and Life Sciences, pl. Grunwaldzki 24a, PL-50-363 Wroclaw, Poland

**Keywords:** Biogeography, Ecological modelling

## Abstract

The Carpathians are an important biodiversity hotspot and a link between mountain ranges on the European continent. This study investigated the phylogeography of one the Carpathian subendemics, *Leucanthemum rotundifolium*, which is distributed throughout the range and in one isolated population outside it. Range-wide sampling was used to examine phylogeographic patterns by sequencing uniparentally inherited chloroplast markers that exemplify seed dispersal. Reconstruct Ancestral State in Phylogenies (RASP) software, Bayesian binary Markov Chain Monte Carlo (BBM) analysis, and ecological niche modeling based on concatenated results of five algorithms were used to infer migration routes and examine links with other species through phylogeny. The round-leaved oxeye daisy is an example of organisms that reached the Carpathians through a southern “Dacian” migration route, most probably through long-distance dispersal. Dating placed the events in the Pleistocene and supported migrations during cooler periods and stasis/isolation followed by separation in the interglacials. Haplotype diversification indicated that after *L. rotundifolium* reached the area around the Fagaras Mountains, several migration events occurred leading to colonization of the Southern Carpathians followed by migration to the Apuseni Mountains, the Eastern Carpathians, and finally the Western Carpathians. The results are consistent with previous phylogeographic studies in this region and indicate several novel patterns.

## Introduction

The Carpathians are the main mountain range in Central Europe at the crossroads between the western–eastern and northern–southern European massifs. Their flora and fauna have been studied for decades, and feature in numerous biogeographic studies on plants and animals^1,2^. Being a hotspot of plant diversity at the pan-European scale the Carpathians harbor a remarkably diverse vascular flora^3–5^. Being surrounded mostly by lowlands, they also exhibit a pattern typical of mountain regions and encompass much wider climatic variability than neighboring areas^6^. However, unlike typical high mountain ranges, their specificity lies in the structure, which is dominated by mid-elevation heights and polonynas (montane meadows), whereas high-elevation peaks with an alpine zone create a chain of spatially isolated islands^7,8^. The biogeography of the Carpathians is also a topic of interest in modern times, and several important works have been published recently^3,9^. Many biogeographical studies have attempted to describe the connections between the Carpathian and other mountain systems, especially the Alps and the Balkan Ranges^3^. Migrations between these mountain chains shape the constituents and reflect the origins of Carpathian biodiversity. Plants can choose two routes during migration from the Alps. They may reach the Western Carpathians via the Eastern Alps (Illyric-Noric route) or reach the Southern Carpathians via the Dinarides and then migrate northwards (Dacian route)^10–12^. Connections between neighboring mountain ranges and the Carpathians are important, since colonization from the Carpathians is also occurring^3^. The Carpathians are interesting not only as a migration stop, but also as a biodiversity hotspot that harbors 420 endemic plant taxa at species and subspecies level^5,13,14^. Of these taxa, 39 are subendemic, and are characterized by some satellite populations outside the Carpathians. Recently, a new project was implemented to gain better knowledge about Carpathian endemic plants^4^.

Within the Carpathians, there are several taxa of the oxeye daisy genus *Leucanthemum* Mill. (Compositae; Anthemideae), which represent the northern group of the Leucanthemineae heavily influenced by hybridization and polyploidization. More than half of the species of this genus are polyploids, and most of the diploid species show signatures of past hybridization^15–18^. One of the oxeye daisies growing in the Carpathians is a subendemic taxon, the round-leaved oxeye daisy (*Leucanthemum rotundifolium* (Willd.) DC. (= *L. waldsteinii* (Sch.Bip.) Pouzar), one of the most divergent species in the genus and characterized by unique morphology (leaf shape) and habitat (preference for moist substrate). In various phylogenetic reconstructions, it is often placed as one of the earliest diverging lineages^15–17^. It owes its subendemic status to its disjunct distribution, with an isolated population found in the Vranica Mountains (Dinarides) ca. 370 km in a straight line from the nearest population in the Southern Carpathians. *L. rotundifolium* is associated with humid soils and is often found close to springs and stream banks. It may be regarded as a montane species because it has never been found below 500 m, which is the approximate lower border of the montane belt in the Carpathians^19,20^. Although it also occurs at higher elevations in the subalpine zone, it never forms numerous populations as it does in the montane zone. At higher elevations, it is similarly confined to stream banks, local depressions, or suitable microclimates created by shrub stands (typically consisting of *Pinus mugo* as a main component). Due to its disjunct distribution and subendemic status, it is an ideal candidate to study the phylogeography of the Carpathians.

Plants are valuable subjects for phylogeographic study because of their neutral dispersal patterns that reflect connections between areas in the past. This is also true of *L. rotundifolium* whose seeds lack any features that could directly facilitate dispersal^20^. After reaching maturity, its small achenes simply fall out of the capitulum, and most lie in proximity to the maternal plant at this stage. They may be fortuitously distributed further by wind, flowing water, or animals^20,21^. Seed dispersal reflects natural processes, such as those that create suitable habitat corridors that enable species to spread or form barriers that inhibit dispersal. At the species level, the emergence of such corridors or boundaries, frequently corresponding to biogeographical patterns, is often influenced by climate change.

The study of angiosperm seed dispersal is facilitated by the fact that chloroplasts are maternally inherited, and the analysis of chloroplast markers provides a direct method. The chloroplast genome also has other features that make it ideal for such studies, such as lack of hybridization and relatively rapid fixation in the population^22^. In contrast to nuclear markers, all chloroplast markers are single copy genes, which, therefore, provide an opportunity for analysis with less noise from hybridization or duplicate events. They have also been used to study the phylogeography of other groups of *Leucanthemum*^23–25^.

This study has two aims: first, investigation of the phylogeography of *L. rotundifolium,* a pan-Carpathian subendemic, which, in addition to its own merits, has the potential to provide general data on biogeographical patterns in the Carpathian Mountains; and second, inferring possible migration routes and tests for the existence of geographically structured genetic variation. More importantly this work aims to determine how and when the disjunction between the Dinarides and the Carpathians occurred and how migration within the Carpathians took place. The study will put these events into a time frame provided by the dating of phylogenetic trees and analyze changes in potential distribution through ecological niche modeling. This addresses issues raised by Ronikier^9^, including broad sampling of Carpathian populations, inclusion of dating to calibrate the time frame of the observed pattern, and exploration of the relationship between the Carpathians and the Dinarides.

## Materials and methods

### Study species

*Leucanthemum rotundifolium* is relatively common in most of the Carpathian ranges and has one disjunct population in the Vranica Mountains of the Dinarides. In the main part of its range, *L. rotundifolium* forms variously sized populations, from large ones thriving in stream valleys to single specimens at high elevations and in sunny habitats. It occurs between 525 and 2060 m a.s.l., but has mostly been found in the upper montane belt (mean 1250 (± 420) m a.s.l.). The fruits of *L. rotundifolium* are small achenes (each weighing approximately 0.4 mg). A single capitulum may contain numerous achenes (approximately 100–300), and a single plant may have one to a few flower heads. These characteristics do not impose any direct mode of dispersal; rather, they may lead to a high number of seeds and dispersal by random events that may include transportation downslope via flowing water and possibly also by wind or animals. In the field, this species is relatively easy to recognize and spot, especially during flowering. It is also morphologically distinct from sympatric congeneric species due to the unique shape of its basal leaves, stem leaves, and capitula.

### Sampling strategies

**Figure 1:**
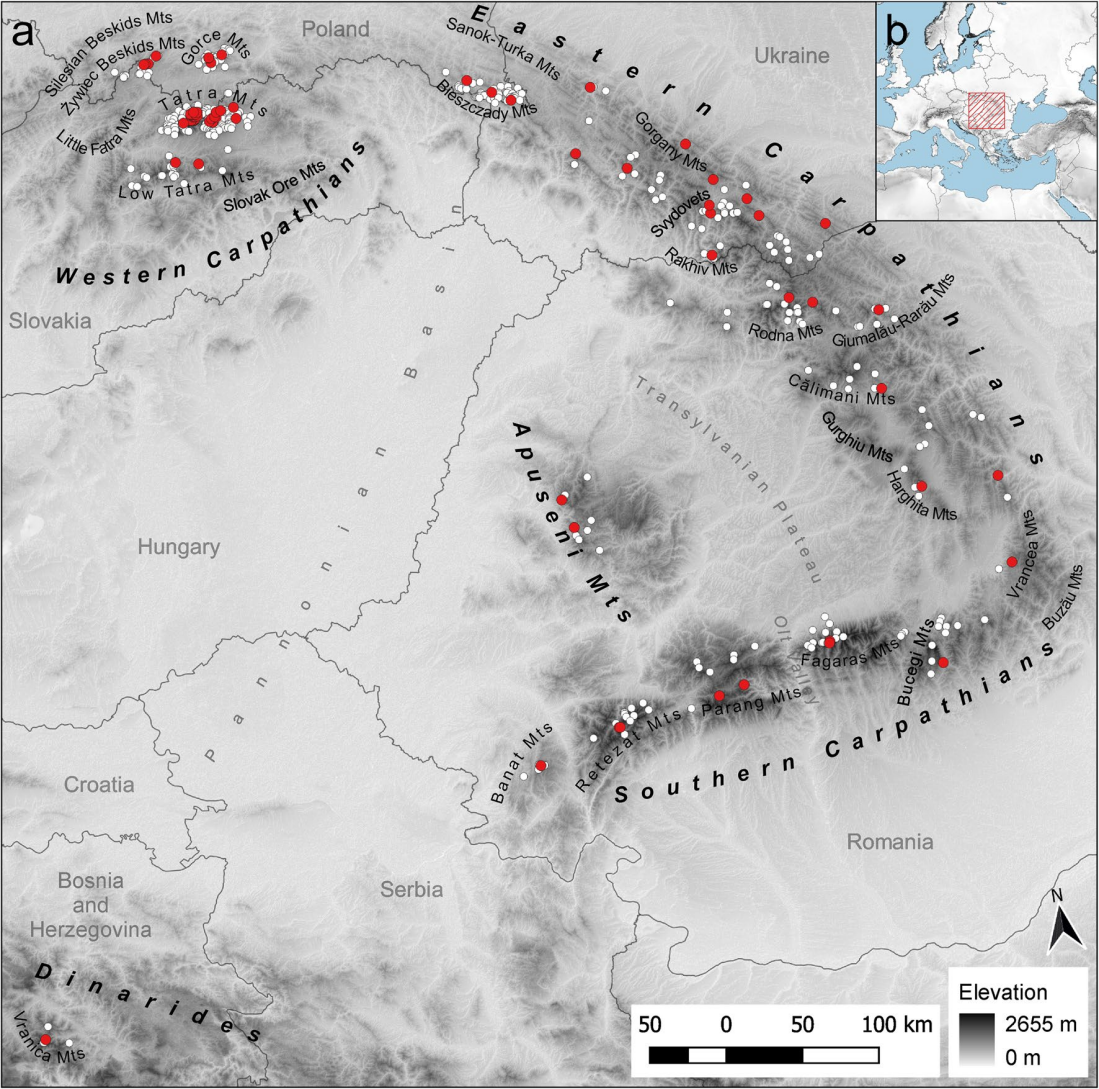
(**a**) The Carpathian region, the area of investigation of this study, with all geographic locations mentioned in the text. Red dots indicate locations sampled for the phylogeographic (cpDNA) study. White dots indicate locations used for ecological niche modeling. Country borders and names are shown for reference. (**b**) Placement of the study area. The underlying image represents the altitude derived from the ALOS DEM dataset^84^. The map is projected in ETRS89 (EPSG: 3035). The maps are drawn using R ver. 3.6.2^76^ and QGIS ver. 3.8^77^.

Species identification was carried out in the field. The sampling design followed the historical locations of *L. rotundifolium* recorded in Zelený (1970) and on herbarium sheets. In the field, each *L. rotundifolium* stand that was spatially isolated from other stands by at least 150 m or visible barriers was collected as a separate population. Following this scheme, samples from 68 populations were collected (Supplementary material S1, Fig. 1). Sampling covered the whole distributional area of the species (Fig. 1). In addition, all known sympatric species, *L. vulgare* Lam. (2*n* = 2*x* = 18), *L. ircutianum* DC. (2*n* = 4*x* = 36), *L. gaudinii* Dalla Torre (2*n* = 2*x* = 18), *L. margaritae* (Jáv.) Zeleny (2*n* = 6*x* = 54), and *L. illyricum* (Horvatić) Vogt & Greuter (2*n* = 8*x* = 72), were collected and later used as outgroups. Field studies on plants, including the collection of plant material, complied with relevant institutional, national, and international guidelines and legislation. The plant material was collected with the permission of the local authorities and according to national laws.

### DNA isolation

DNA was extracted from five individuals in each population, except for populations that were closer than 4 km from which only one to three individuals were sampled (depending on sampling density). DNA was extracted from silica-gel-dried leaves that were pulverized in an adjusted plastic rack attached to a reciprocating saw or jigsaw^26^. I used the “Sherlock AX” DNA extraction kit (A&A Biotechnology, Gdynia, Poland) according to the manufacturer’s protocol.

### PCR amplification

In the following analyses, I used two datasets: the first included samples of the representatives of the genus *Leucanthemum*, members of the subtribe Leucantheminae, and selected accessions of *L. rotundifolium*^16^; and the second consisted of all *L. rotundifolium* accessions and sympatric species. The first dataset used chloroplast markers and accessions that were used to reconstruct the phylogeny of *Leucanthemum*^16^. These included five intergenic spacer regions (*psb*A*-trn*H, *trn*L*-trn*F, *trn*C*-pet*N, *pet*N*-psb*M, and *trn*Q*-rps*16)^27^. Initial screening revealed very low variability of those markers within the studied species. Therefore, new primer pairs were designed for the most variable regions within the genus *Leucanthemum*^28^. First, I downloaded available whole plastome sequences for the Asteraceae from GenBank (www.ncbi.nlm.nih.gov/genbank) and searched for conserved regions spanning exons pinpointed by Scheunert et al.^28^. Then, I placed primers in places that would yield a PCR product around 1000 bp. Several such primers were constructed and after screening, two of these primer pairs, ndhCretF (AAGTTTCTCCGGTCCTTTGC)—trnV-comp-R (CTCTTTTCCTGTCCGAAATC) and *trn*T(GGU)-comp-F (AAGTGGACCTGACCCATTG)—*psb*D-comp-R (GACCATTTCCGAACACCTC), were selected based on their level of polymorphism and feasibility of amplification. The first pair was already proposed by Timme et al.^29^ as *ndh*CretF-*trn*VretR as a marker for the Asteraceae, but the reverse primer was shifted by a few nucleotides to exclude polymorphic sites. Together with *psb*A*-trn*H, these three primer pairs were amplified for all *L. rotundifolium* individuals and sympatric outgroup species and constitute a second dataset. All PCR reactions were performed in the T100 Thermal Cycler (Bio-Rad, Hercules, USA) with 15 µl volume using Taq 2 × Master Mix RED with 1.5 mM MgCl_2_ (Ampliqon, Odense M, Denmark) and 10 µmol of each primer. The PCR program consisted of the following steps: 5 min at 95 °C, followed by 35 cycles of 30 s at 95 °C, 40 s at 55 °C, and 30 s at 72 °C, followed by 10 min at 72 °C. A negative control was always included, and if any sign of contamination or cross-contamination was visible, all samples were discarded. The PCR products were cleaned with CleanNGS (CleanNA, Waddinxveen, The Netherlands) and sent to Macrogen Europe (Amsterdam, the Netherlands) for sequencing. Sequence chromatograms were visually inspected in Chromas (Technelysium Pty Ltd, South Brisbane, Australia) and then aligned in Bioedit^30^. In cases of poor read quality, sequencing was repeated using both forward and reverse primers. The few segments that could not be aligned unambiguously (mostly sites containing poly-T repeats of variable length) were deleted from the final alignment as a polymerase-induced error. Sequence data were submitted to the GenBank database under accession numbers OP451022 to OP451789, and alignments are available as Supplementary Material S2.

### Phylogenetic analyses and dating

I constructed phylogenetic trees using MrBayes 3.2.7a^31^ with all markers and indels as a separate partitions. Indels were coded using the simple gap coding^32^ implemented in SeqState 1.4.1^33^. For all partitions, the best fitting model was selected using jModelTest^34,35^, and the Jukes–Cantor model^36^ was applied for gaps. Two separate runs were performed, each consisting of four chains, 15 × 10^6^ generations, and sampling every 1000th tree. The first 25% of the trees were discarded as a burn-in period. The convergence of chains and ESS values were checked in the Tracer software^37^.

The first data set used the alignment of five intergenic spacer regions of the chloroplast genome (*psb*A*-trn*H, *trn*L*-trn*F, *trn*C*-pet*N, *pet*N*-psb*M, and *trn*Q*-rps*16) and contained all diploid *Leucanthemum* species, outgroups from the subtribe Leucantheminae, and selected *L. rotundifolium* accessions in order to produce a dated phylogeny comparable to that from a previous study^16^. The second data set contained all *L. rotundifolium* individuals and sympatric species as outgroups to reconstruct phylogeographic patterns for the focal species. Although previous dating estimates in the genus were obtained using BEAST software^16,17^ in this case, dating was performed using MEGA software employing the RelTime method^38–41^. BEAST^42^ showed problems in reaching chains convergence, which may be due to difficulties in analyzing the presence/absence data in the indels partition or due to the mixing of different sampling levels i.e. species mixed with a denser sampling of *L. rotundifolium* populations. MEGA can use trees produced by other programs (MrBayes in this case) and RelTime has been shown to perform well in cases where the taxa studied come from data sets containing a mix of samples at specific and subspecific levels^43^. I used four dating points: two geological events that separated an island endemic from a mainland relative, i.e., *Plagius flosculosus* from *P. maghrebinus* and *Mauranthemum paludosum* from *M. ebusitanum*^16^, the crown age of *Leucanthemum*^17^, and the split between *Artemisia* and other Anthemideae estimated from fossilized pollen^44^. The details are provided in Table 1. To date the tree obtained from the second dataset, I used nodes from the first analysis that included taxa present in both analyses (Table 1).

**Table 1:** Divergence times and 95% confidence intervals (CI) for nodes used for dating (nodes A–D from the 1st dataset and E–F from the 2nd dataset).

Node	Description	Prior distribution		Estimated distribution		References
		Mean age	95% CI	Mean age	95% CI	
A	Root age, divergence between Artemisia and Leucanthemineae	31.0	32.0–30.0	31.035	31.84–30.239	^41,80^
B	Divergence between *Mauranthemum paludosum* and *M. ebusitanum*	5.3	6.3–4.30	4.380	5.278–4.216	^14^
C	Divergence between *Plagius flosculosus* and *P. maghrebinus*	7.25	8.9–5.6	5.852	7.255–5.596	^14^
D	Crown age of *Leucanthemum*	1.93	2.71–1.15	1.417	2.111–1.208	^15^
E	Divergence between “*L. vulgare* group” and *L. rotundifolium*	1.42	2.21–0.63	1.397	1.87–0.953	This study
F	Divergence between “*L. ircutianum* group” and *L. rotundifolium*	0.65	1.0–0.30	0.657	0.866–0.479	This study

All dating points follow a normal distribution. Numbers are in million years.

### Haplotype network and clustering

To produce the haplotype network, I used a TCS algorithm^45^ implemented in the software PopART^46^. To assess the number of genetic clusters, I used Bayesian Analysis of Population Structure (BAPS)^47^, a hierarchical genetic clustering algorithm, available in the RhierBAPS package for R^48^. Three separate runs testing 2–34 populations and two levels were conducted.

### Bayesian binary Markov Chain Monte Carlo analysis

To estimate ancestral areas, I used the software Reconstruct Ancestral State in Phylogenies (RASP)^49^ and Bayesian binary Markov Chain Monte Carlo analysis (BBM)^50^ with the consensus trees. The advantage of this method for this data set was that it could deal with polytomies, while this feature of the phylogenetic trees prevented the use of other methods. The BBM method was independently run twice using 1 × 10^6^ generations, sampling every 1000^th^ generation, and discarding the first 25% as a burn-in. The maximum number of areas per node was set to four, the state frequencies were fixed following the Jukes–Cantor model, and the variation between sites was set to equal. The results of both runs were combined within the program.

### Species distribution modeling

Modeling followed the gold standards in ecological niche modeling^51^ and used an ensemble of several algorithms rather than a single model. To select the best-performing algorithms, I used the method proposed earlier for the same species^52^, and compared the same set of 13 algorithms as the original article and chose those with the highest AUC and MAE scores. AUC and MAE were calculated using the package Metrics in R^53^. The five selected algorithms were ANN^54,55^, CTA^55,56^, MAXENT^57^, BRT^58,59^, and BART^60^. Each algorithm was repeated 100 times; their settings were as described in Konowalik and Nosol^52^.

### Points for modeling

As a source of points for modeling, I used a combined data set, which joined data from Jasiewicz^61^, Kornaś^62^, and Zelený^20^ and georeferenced herbarium specimens and field trips. Irrespective of the source, I used only locations that could be unambiguously assigned to a certain place with an accuracy higher than the rasters used (0.5 km^2^). Occurrences within a single raster cell were reduced to one point. To further decrease spatial bias, I applied environmental filtering, which performs better than geographical filtering^63–65^. I used the algorithm provided by Varela et al. (2014) at https://github.com/SaraVarela/envSample. As filters, I used PCA maps computed from previously selected environmental variables and filtered presences and background using the same settings. The background was generated as in Konowalik and Nosol (2021) and consisted of all data points above 400 m within the Carpathians. All data points falling within one unit of principal component axis 1 and principal component axis 2 were trimmed to only one randomly selected data point. After all filtering procedures, 429 presence data points were left for the final analyses (Fig. 1).

### Variable selection

The analysis was restricted to the part of Central Europe that includes the Carpathians and neighboring regions, which define the most probable accessible area of the studied species^66^. I downloaded 19 bioclimatic variables for this region from CHELSA 1.2 with resolution of 30 arc seconds^68^. Pearson’s correlation coefficient was used to assess associations between variables, which were removed stepwise until all variables with ρ < 0.7 were removed. Variables showing linear dependencies and zero or near-zero variance were removed with the Caret package in R^69^. I wanted to further reduce the number of environmental variables to those that are influential for the studied species. A previous study indicated that the use of modeling algorithms to select variables may need further development^70^; therefore, I used a customized procedure. To reach consensus between different algorithms, an elimination strategy was used in which a variable that was less important than a random variable in at least three out of five algorithms was deleted. For this purpose, the spatialEco package in R^71^ was used to create random variables with the same range of values as those of the true variables. I ran models with true and random variables jointly and after each run, discarded a variable that performed worse than a random variable. This procedure was repeated for each algorithm until all true variables showed a higher contribution than any of the random variables. Finally, I compared the variables discarded by different algorithms and removed from the final data set those variables discarded by the majority of the algorithms (Table 2).

**Table 2:** Sets of variables used for modeling.

Period	Age/duration	References
Pleistocene, Marine Isotope Stage 19 interglaciation (MIS19)	ca. 787 ka	Brown et al., (2018)
Pleistocene, Last Interglacial	ca. 130 ka	Otto-Bliesner et al. (2006)
Pleistocene, Last Glacial Maximum	ca. 21 ka	Karger et al., (2021)
Pleistocene, Heinrich Stadial 1	17.0–14.7 ka	Fordham et al., (2017)
Pleistocene, Bølling–Allerød	14.7–12.9 ka	Fordham et al., (2017)
Pleistocene, Younger Dryas Stadial	12.9–11.7 ka	Fordham et al., (2017)
Pleistocene, early Holocene, Greenlandian	11.7–8.326 ka	Fordham et al., (2017)
Pleistocene, mid-Holocene, Northgrippian	8.326–4.2 ka	Fordham et al., (2017)
Pleistocene, late Holocene, Meghalayan	4.2–0.3 ka	Fordham et al., (2017)
Anthropocene	1979–2013	Karger et al., (2017, 2018)

### Predicting past distribution

To predict past distributions, I used variables available on PaleoClim.org^72^. These variables are computed using CHELSA^68^ and contain the same set of 19 bioclimatic variables available for the periods specified in Table 2. Models for potential past distributions were generated using the same methods and algorithms as for the present time.

### Model ensemble

Each algorithm was run individually using the same set of presence and background points. In order to find consensus between modeling algorithms that minimized discrepancies and reduced extreme predictions, I produced an ensemble model. It was calculated using principal component analysis (PCA), which is recommended for producing consensus forecasts^73,74^. As input, all the models produced for a given period were submitted to a standard PCA calculation. The first PCA component (PC1) shows the central tendency over all individual models and represents a consensus between them. The resulting files were rescaled to the 0–100 range, where 0 showed low suitability and 100 high suitability for the studied plant species. All procedures were carried out with the ‘raster’ package and using basic functions in R^75^. All GIS operations were done in R ver. 3.6.2^76^ and QGIS ver. 3.8^77^.

## Results

### Phylogenetic analyses and dating

**Figure 2:**
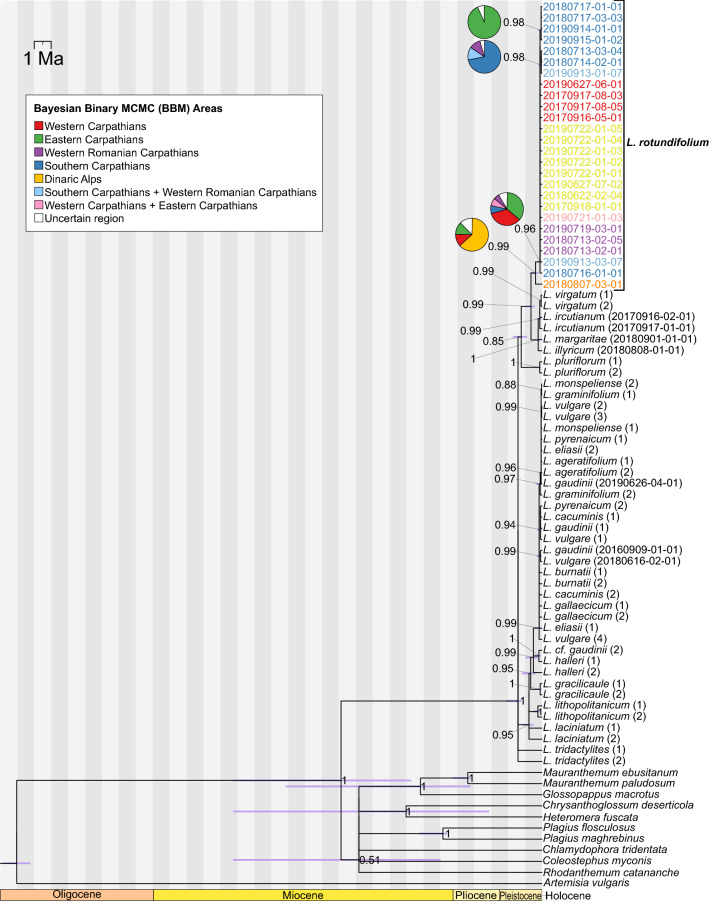
Phylogenetic tree constructed using five intergenic spacer regions in cpDNA (*psb*A*-trn*H, *trn*L*-trn*F, *trn*C*-pet*N, *pet*N*-psb*M, and *trn*Q*-rps*16). The numbers associated with the nodes indicate posterior probabilities from Bayesian analysis. Bars represent uncertainty in the dating of a particular node. Pie charts represent the most plausible ancestral locations inferred by the Bayesian binary Markov Chain Monte Carlo method (BBM), and are shown only for nodes associated with the focal species. The samples of *L. rotundifolium* are colored according to the results of the clustering algorithm (Fig. 4).

**Figure 3:**
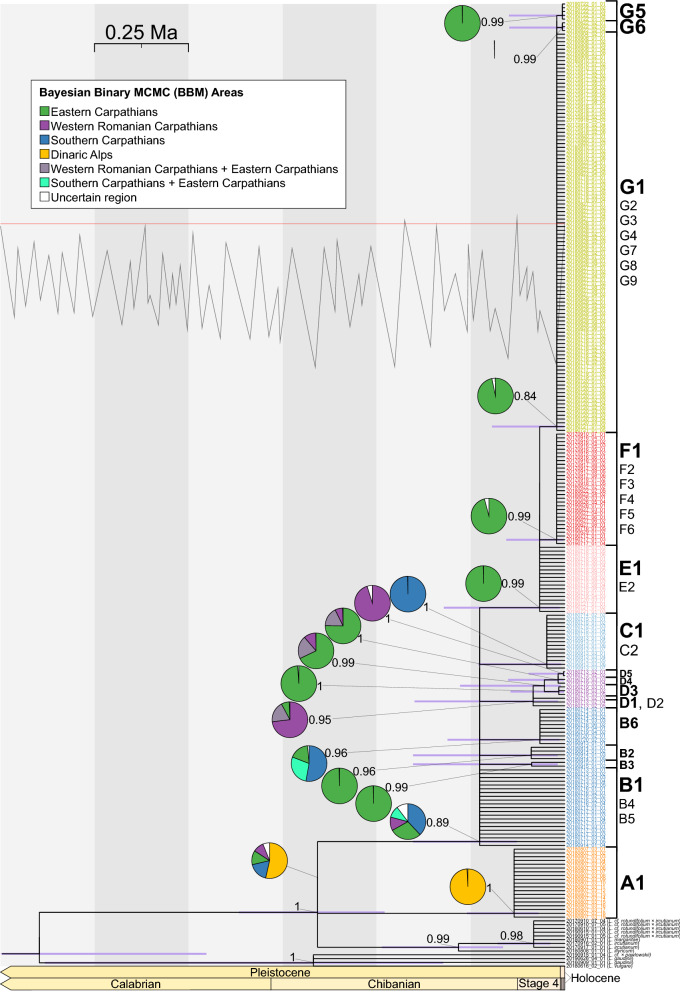
Phylogenetic tree constructed with three intergenic spacer regions in cpDNA (*psb*A*-trn*H, *ndh*C*ret-trn*V*-*comp, and *trn*T(GGU)*-*comp*-psb*D*-*comp). The numbers associated with the nodes indicate posterior probabilities from Bayesian analysis. Bars represent uncertainty of dating for a particular node. Pie charts represent the most plausible ancestral locations inferred by Bayesian binary Markov Chain Monte Carlo method (BBM) and are shown only for nodes associated with the focal species. The *L. rotundifolium* samples are colored according to the results of the phylogenetic analysis of the clustering algorithm (Fig. 4). The tree includes all sympatric species of *L. rotundifolium* (*L. vulgare* 2*x*, *L. gaudinii* 2*x*, *L. ircutianum* 4*x*, *L. margaritae* 6*x*, and *L. illyricum* 6*x*). The line in the upper part of the image represents changes in global temperature in the past. The horizontal red line indicates modern thermal conditions, while the peaks indicate higher temperatures and the troughs lower temperatures. The original figure was created by Robert A. Rohde, based on Lisiecki and Raymo (2005), and is available at https://commons.wikimedia.org/wiki/File:Five_Myr_Climate_Change.png.

In the phylogenetic tree constructed using the five intergenic spacers and their indels *L. rotundifolium* is part of the lineage composed of diploid *L. virgatum* and polyploids (Posterior probability PP = 0.99, Fig. 2). The stem age of this group is 0.65 Ma (Confidence interval CI 0.99–0.42). Within the *L. rotundifolium* clade, a major divergence dating from 0.38 Ma (CI 0.70–0.21) separates the Dinarides population from the rest of the species (PP = 0.99). This analysis was performed mainly to place the *L. rotundifolium* accessions within the framework previously proposed for the genus. However, due to low resolution, a full sampling was not carried out; more detailed relationships between the focal species are observable in the second data set. The second phylogenetic tree was constructed using three markers and displays a structure similar to that of the first tree (Fig. 3). A notable difference is associated with the MRCA of *L. rotundifolium*, at whose node the Dinarides population, polyploids, and the rest of the *L. rotundifolium* accessions are placed in a polytomy (PP = 1). This node dates from 0.66 Ma (CI 0.87–0.48). This may be the effect of not considering other diploid taxa included in the first analysis and could provide more information at this node. The base of the *L. rotundifolium* clade is formed by the plants from the Southern Carpathians; this node dates from 0.23 Ma (CI 0.40–0.13). The clade containing B3 and B2 haplotypes is significantly supported (PP > 0.96), and its origin is estimated to be around 0.09 Ma (CI 0.4032–0.186). The clade containing the B6 haplotype (PP = 0.96) is estimated to originate from 0.0661 Ma (CI 0.3117–0.014). A clade containing the cluster of D haplotypes (PP = 0.95) is estimated to have emerged 0.084 Ma (CI 0.399–0.017) and the youngest clade within this group containing the D5 haplotype (PP = 1) dates from 0.0036 Ma (CI 0.096–0.0001). The clade that includes the C1 haplotype (PP = 1) originated around 0.0475 Ma (CI 0.23–0.01). The clade containing the E1 haplotype (PP = 0.99) originated 0.0665 Ma (CI 0.3213–0.0138). The clade that stems from this group and contains the cluster of F haplotypes is estimated to originate around 0.0216 Ma (CI 0.1554–0.003). The clade containing haplotype G1 (PP = 0.84) is estimated to originate 0.0211 Ma (CI 0.1934–0.0029). There may be two supported clades within this group: one specific to the Gorgany Mountains originating 0.0062 Ma (CI 0.1483–0.0003) and the other specific to the Bieszczady Mountains originating 0.0061 Ma (CI 0.1474–0.0003) (Table 3).

**Table 3:** Divergence times of haplotypes obtained from the analysis of the phylogenetic tree constructed with three intergenic spacer regions in cpDNA (*psb*A*-trn*H, *ndh*C*ret-trn*V*-*comp, and *trn*T(GGU)*-*comp*-psb*D*-*comp).

Description	Estimated distribution		Posterior probablity
	Mean age	95% CI	
*L. rotundifolium* stem age	0.6571	0.866–0.4787	1.00
A1 crown age	0.1345	0.2598–0.0697	1.00
B1 crown age	0.2262	0.4032–0.1269	0.89
B2 crown age	0.0891	0.4032–0.0191	0.96
B3 crown age	0.0879	0.4032–0.0186	0.99
B6 crown age	0.0661	0.3117–0.014	0.96
C1 crown age	0.0475	0.2298–0.0098	1.00
D1 crown age	0.084	0.3992–0.0167	0.95
D3 crown age	0.0159	0.144–0.0817	1.00
D4 crown age	0.0176	0.1507–0.002	1.00
D5 crown age	0.0036	0.0961–0.0001	1.00
E1 crown age	0.0665	0.3213–0.0138	0.99
F1 crown age	0.0216	0.1554–0.003	0.99
G1 crown age	0.0211	0.1934–0.0029	0.84
G5 crown age	0.0062	0.1483–0.0003	0.99
G6 crown age	0.0061	0.1474–0.0003	0.99

Numbers are in million years.

### Haplotype network and clustering

**Figure 4:**
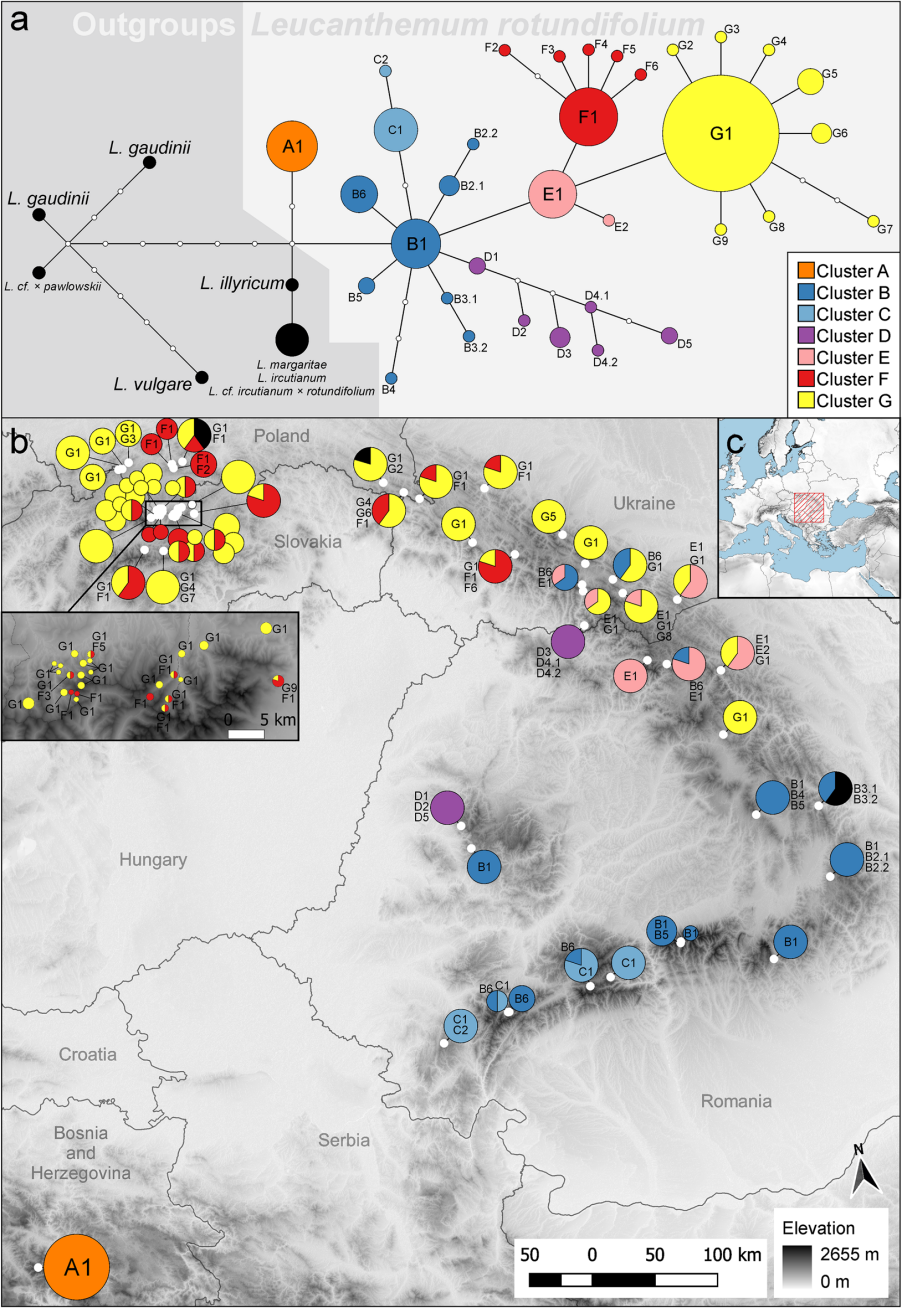
Haplotype network and haplotype distribution. (**a**) Haplotype network constructed with the TCS algorithm using three intergenic spacer regions in cpDNA (*psb*A*-trn*H, *ndh*C*ret-trn*V*-*comp, and *trn*T(GGU)*-*comp*-psb*D*-*comp). The network contains all representatives of *L. rotundifolium* included in this study and all sympatric species, but for the sake of clarity, sample names are not shown (for haplotype membership, see Supplementary Material S3). (**b**) Map showing the placement of haplotypes. The size of the pie charts is proportional to the number of sequenced individuals and the size of the slices is proportional to the percentage of individuals belonging to a certain haplotype. Due to dense sampling in the Tatra Mountains, the area is enlarged within an inset on the left. (**c**) Location of the study region. The underlying image (**b**,**c**) represents the altitude derived from the ALOS DEM dataset^84^. The map is projected in ETRS89 (EPSG: 3035). The maps are drawn using R ver. 3.6.2^76^ and QGIS ver. 3.8^77^.

The clustering applied to the phylogenetic tree constructed using three markers found that the optimal number of partitions is seven. This analysis divides *L. rotundifolium* into groups that correspond to geography and the haplotype network (Fig. 4). Similar to the phylogenetic tree, the haplotype network places a polytomy at the MRCA of *L. rotundifolium*, and the Dinarides population (haplotype: A1) is detached from the species (Fig. 4). The basal node of *L. rotundifolium* (PP = 0.89) consists of haplotype B1, which is placed in a polytomy with several well-supported groups. Haplotype B1 is distributed mainly in the Southern Carpathians. Haplotype B1 is related to several child haplotypes, B2–B6. Most of these haplotypes occur in the Southern Carpathians from the Banat Mountains to the Harghita Mountains. One notable exception is haplotype B6, which is found not only in the Retezat Mountains and the Parang Mountains, but also in the central part of the Eastern Carpathians from the Svydovets to the Rodna Mountains. Two distinct groups emerge from the B1 haplotype: the C1–C2 haplotypes and the D1–D5 haplotypes. Cluster C is found only in the Southwestern Carpathians from the Banat Mountains to the Parang Mountains, and its eastern border is marked by the Olt valley. The D cluster haplotypes occur in the northern part of the Apuseni Mountains and in the Rakhiv Mountains. The basal haplotypes, D1 and D2, which form a clade (PP = 0.95), occur in the Apuseni Mountains; the sister to this group is the clade composed of the D3 and D4 haplotypes in the Rakhiv Mountains and D5, again found only in the Apuseni Mountains. Since all these haplotypes form clades supported by a PP of > 0.95, this peculiar pattern may be explained as two waves of migration: the first colonizing the Rakhiv Mountains from the Apuseni Mountains, and the second colonizing the Apuseni Mountains with intermediate events that led to the separation and divergence of these haplotypes. Another group (PP = 0.99) stemming from the B1 haplotype is E1. This cluster is found in the central part of the Eastern Carpathians. From this haplotype emerged two new clusters, F and G, which spread further north and reached the Western Carpathians.

Despite the large area occupied by the plants belonging to the F and G clusters, they are somewhat less divergent and contain mostly satellite haplotypes that occur without a clear pattern and include only one to a few individuals. However, there are a few exceptions, such as haplotype G5, which groups all individuals in the Gorgany Mountains and does not occur anywhere else. Furthermore, haplotype G6 is found only in one place in the Bieszczady Mountains. The distribution of both F and G clusters is similar except that cluster G is more widespread. Both clusters occur in a mosaic in the Western Carpathians, especially in the Tatra Mountains. Most of the plants from the Gorce Mountains belong to the F1 haplotype, and all plants from the Żywiec Beskids Mountains belong to the G1 haplotype. During the analyses, a few specimens resembling *L. rotundifolium* were classified into the haplotypes assigned to the outgroup species. It is possible that they are hybrids between *L. rotundifolium* and other species, but this needs further examination.

### Bayesian binary Markov Chain Monte Carlo analysis

In the event matrix, RASP suggests the following scenario involving dispersal and then vicariance as the most probable for the MRCA node of *L. rotundifolium*: Dinarides → Dinarides + Southern Carpathians → Dinarides | Southern Carpathians. The Dinarides are also the most probable ancestral area for this node with a probability of 0.53 (while P = 0.18 for the Southern Carpathians, P = 0.13 for the Eastern Carpathians, and P = 0.1 for the Apuseni Mountains). The ancestral area of the node containing, among others, the B1 haplotype, is located in the Southern Carpathians (P = 0.38), the Eastern Carpathians (P = 0.29), the Apuseni Mountains (P = 0.12), or the Southern and Eastern Carpathians (P = 0.11). This node is characterized by multiple dispersal events that took place from the Southern Carpathians, which led to the colonization of other parts of the range plus the Apuseni Mountains and the Eastern Carpathians. The D cluster, as described previously, displays a peculiar pattern of origin in the northern part of the Apuseni Mountains (P = 0.73), dispersal to the Rakhiv Mountains where two new haplotypes originated (with P = 0.75 and P = 0.99), and then redispersal to the Apuseni Mountains where a new haplotype emerged (P = 0.95). The origin of the C cluster is in the Southern Carpathians (P = 0.99). The ancestral area of the node containing haplotype E1 is located in the Eastern Carpathians (P = 0.99). Similarly, the ancestral area of nodes containing the G1 and F1 haplotypes is located in the Eastern Carpathians (P = 0.97 and P = 0.96, respectively). The ancestral areas of nodes containing haplotypes G5 and G6 are also located in the Eastern Carpathians, and as described previously, they are found only locally within a small area.

### Species distribution modeling and variable selection

**Figure 5:**
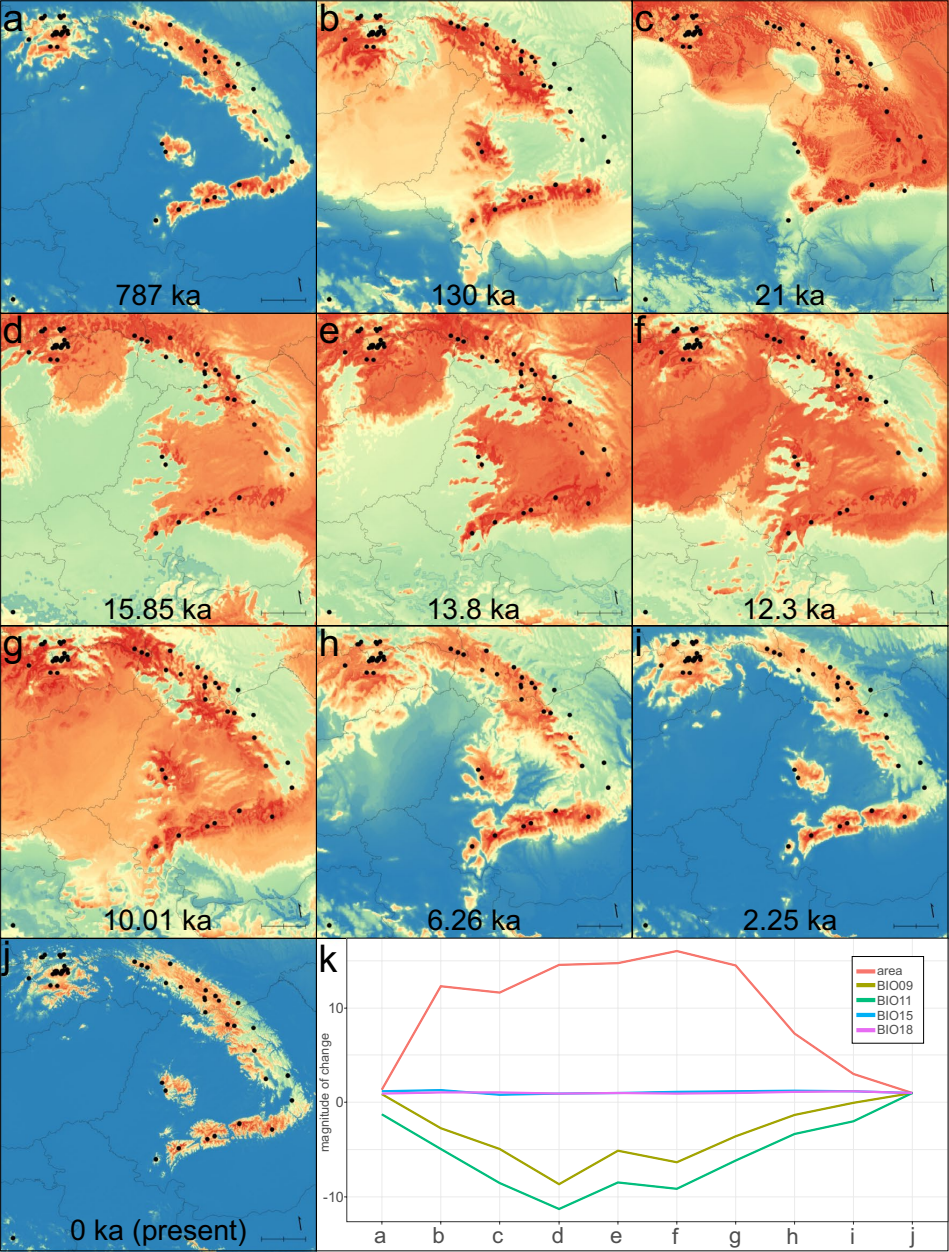
Potential distribution of *L. rotundifolium* throughout the Pleistocene epoch determined by five niche modeling algorithms (MAXENT, BRT, BART, CTA, and ANN) summarized by their first principal component axis. Warmer colors indicate higher suitability, while blue indicates unsuitable areas. (**a**) MIS 19 interglaciation ca. 787 ka; (**b**) last interglacial ca. 130 ka; (**c**) last glacial maximum ca. 21 ka; (**d**) Heinrich Stadial 1 17.0–14.7 ka; (**e**) Bølling-Allerød 14.7–12.9 ka; (**f**) Younger Dryas Stadial 12.9–11.7 ka; (**g**) Greenlandian 11.7–8.326 ka; (**h**) Northgrippian 8.326–4.2 ka; (**i**) Meghalayan 4.2–0.3 ka; (**j**) the Anthropocene, 1979–2013; and (**k**) trends in variables used in the modeling displayed as a mean across the map area, with x-axis labels corresponding to the facets of the figure. BIO09 = mean temperature of the driest quarter; BIO11 = mean temperature of the coldest quarter; BIO15 = seasonality of precipitation; BIO18 = precipitation of warmest quarter; and “area” corresponds to the number of pixels with suitability greater than 30%. The y-axis shows the magnitude of the change, where 1 is the value given for the present conditions. The scale bar in the bottom right corner of each map is equivalent to 100 km. The maps are projected using ETRS 89 (EPSG: 3035). Sampled populations used in this study are shown as black circles to indicate their present distributions. The maps are drawn using R ver. 3.6.2^76^ and QGIS ver. 3.8^77^.

The most important variables selected by majority consensus were mean temperature of the driest quarter (BIO09), mean temperature of the coldest quarter (BIO11), seasonality of precipitation (BIO15), and precipitation of the warmest quarter (BIO18). Generally, temperature-related variables had a highly important association with the area of the suitable niche as exemplified by Fig. 5, which shows a close relationship between predicted area and changes in temperature. These variables are also biologically relevant for a mountainous species associated with humid soils. First, temperature limits the vertical distribution of *L. rotundifolium* by excluding competition from lowland species not adapted to low temperatures and confining its range to a suitable area limited by harsher conditions present at higher elevations. Second, the amount of precipitation plays an important role during the warmest period when it is crucial for survival; relatively equal distribution of precipitation throughout the year without severe droughts is also a factor enabling long-term survival of this species.

### Model ensemble

In general, the models reconstructed similar patterns, although there were discrepancies in the extent of the potential suitable habitat and the assigned probability of occurrence. In this case, making an ensemble helped to obtain a less equivocal and more balanced results. The derived ensemble maps correlate well with the distribution recorded in the field. The ensemble shows that there are discontinuities within the range. These breaks are chiefly associated with valleys or lowlands. Within the mountains, the pattern is slightly more complicated, but high mountain peaks are not predicted as suitable (especially in the north), and suitability is higher for the eastern and northern slopes. Moderate suitability is shown for the Vranica Mountains in the Dinarides. There are no suitable locations between the Dinarides and the Southern Carpathians, except for a few areas with low suitability scores. Models also predict a high probability of occurrence in areas where species were not found. Such areas include the eastern part of the Apuseni Mountains, the Slovak Ore Mountains, the Sanok-Turka Mountains, the Gurghiu Mountains, and the Buzău Mountains.

### Predicting past distribution

The models built for the past show a consistent distribution pattern congruent with haplotype diversification. The earliest available datasets for paleoclimate come from the last interglacial 787 ka, which is later than the main diversification of *L. rotundifolium*. Yet, several haplotypes emerged within this period, for example, some that belong to cluster D. Although today there is no suitable habitat between the Rakhiv Mountains and the Apuseni Mountains, corridors or even larger suitable areas are shown to have been available during past climatic oscillations. The same is true for a connection between the Western and Eastern Carpathians. The main suitable areas are always located within the mountains, but depending on the epoch, additional areas appear or disappear. For example, the Transylvanian Plateau is predicted as a relatively suitable area in the period from 21 to 10 ka. Large areas in the east are reconstructed as suitable from 21 to 13.8 ka. During heavier cooling, higher elevations within the Western Carpathians became unsuitable. The suitability of the Eastern Carpathians also varied through time, and most of the time, it displayed a mosaic of areas with low and high suitability. As indicated by the evidence, the extent of the potentially suitable habitat is tightly linked to temperature. During colder times, the species may expand its range, as its niche covers a larger area, whereas during interglacial and warmer periods, it is confined to higher elevations (Fig. 5k). However, the pattern is not straightforward as the maximum extent of the range in the analyzed time period is during the Younger Dryas Stadial (the suitable area is 16 × larger compared to the present area), which is not the coldest period. Much colder conditions appeared during the Last Glacial Maximum, and the range in that period is smaller (this may also be seen as retreat from higher elevations). This indicates that climate change does not act linearly on *L. rotundifolium*, and that the best climate is one similar to the present climate of the montane zone of the Carpathians. Other factors, such as those related to precipitation (e.g., BIO15 and BIO18 in this study) may also play a role but are less influential than temperature-related variables.

## Discussion

### Migration into and colonization of the Carpathians

**Figure 6:**
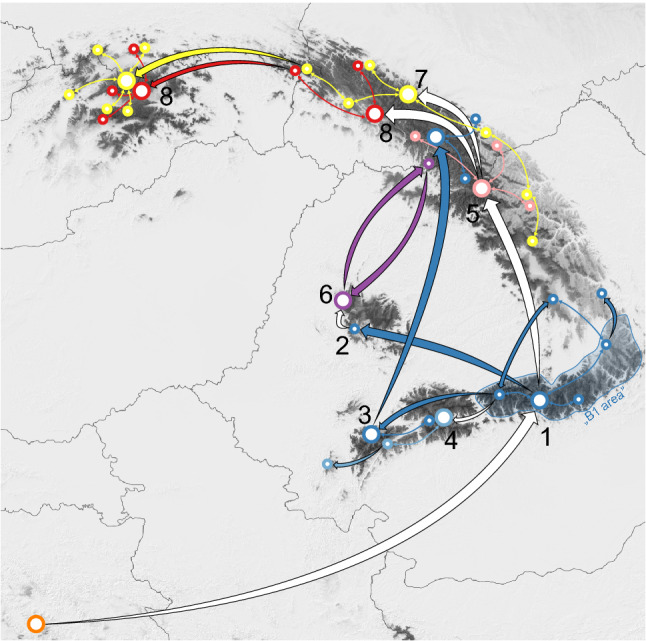
Hypothetical phylogeographic history and migration routes as inferred from haplotype network reconstruction, ecological niche modeling, and RASP analysis (Bayesian binary Markov Chain Monte Carlo). Dispersal and/or vicariance events are numbered and discussed in the text. Thick arrows indicate the emergence of a new haplotype (white arrows) or long-distance dispersal of the haplotypes belonging to the same cluster (arrows in color). Smaller bidirectional arrows indicate connections between populations where the same haplotypes were found. The colors correspond to those assigned to the haplotypes by the clustering algorithm (Fig. 4). The underlying map is generated from ecological niche modeling for the current period, and it is the first axis of the principal component analysis of the five algorithms (as in Fig. 5j) with darker colors indicating higher suitability. For ease of interpretation, political borders between countries are included. The maps are drawn using R ver. 3.6.2^76^ and QGIS ver. 3.8^77^.

This paper presents a hypothesis of dispersal and migration of the Carpathian subendemic *L. rotundifolium*. The hypothesis is based on evidence from chloroplast genetic markers, which, therefore, tightly links it to seed dispersal. According to the results obtained, the study was able to reconstruct migration routes; the most plausible hypothesis of migration is illustrated in Fig. 6. The ancestral range of *L. rotundifolium* can be confidently located in the Dinarides. Although there is only one confirmed population in this area, its range could be larger in the past (Fig. 5b–h) and subsequently reduced most probably by climatic changes. From this area, it spread to the Carpathians, likely as a result of long-distance dispersal. The area that was first colonized within the Carpathians is associated with the occurrence of haplotype B1 and includes the Fagaras Mountains and adjacent areas (Fig. 6, event 1). Dispersal to the Carpathians led to vicariance of the *L. rotundifolium* populations; since then, the Dinarides population has remained isolated. Similar findings have been reported for *Heliosperma* (Rchb.) Rchb. in which a major divergence occurred between Dinarides and Carpathian lineages and results similarly suggested colonization of the Carpathians from the Dinarides^78^. After initial establishment, *L. rotundifolium* spread within the area marked “B1 area” (Fig. 6) in the eastern part of the Southern Carpathians. Haplotype B1 gave rise to several other haplotypes that migrated to other parts of the Carpathians; this includes colonization of the Apuseni Mountains (Fig. 6, event 2), the Retezat Mountains (Fig. 6, event 3), the Parang Mountains (Fig. 6, event 4), and the Eastern Carpathians (Fig. 6, event 5). The Eastern Carpathians were colonized at least three times by plants belonging to different lineages (Fig. 6, events 3, 5 and 6). One of these dispersals is quite interesting as plants belonging to haplotype B6 are found in the Southwestern Carpathians, mainly in the Retezat Mountains, and in the Eastern Carpathians (Fig. 4). A similar pattern of distribution between the Southern and Eastern Carpathians is characteristic of some endemic species and subendemic or mountainous species^4,9^. Plants in cluster C colonized the southwestern part of the Carpathians, eventually reaching the Banat Mountains (Semenic Mountain), which is the westernmost location of *L. rotundifolium* in the Southern Carpathians. Very similar patterns (occurrence of a separate haplotype in the westernmost part of the Southern Carpathians and links between the Southern and Eastern Carpathians) have also been observed in *Campanula alpina* Jacq.^79^ and *Arabis alpina* L.^80^.

Another interesting pattern is observed in the Apuseni Mountains and the Rakhiv Mountains; it appears that there was connection between these two chains since they are inhibited by haplotypes which are not found in any other part. This migration occurred repeatedly and was influenced by isolation leading to the emergence of several haplotypes specific to cluster D. Corridors or even larger suitable areas were available during the past climatic oscillations (Fig. 5). Mráz et al. (2007) observed a similar pattern that linked the Apuseni Mountains with the Eastern Carpathians in the case of *Hypochaeris uniflora* Vill. Another haplotype emerging from the basal group is E1 (Fig. 6, event 5), which is distributed in the Eastern Carpathians. This connection between the Southern and Central Eastern Carpathians is a pattern exhibited by other species, e.g., *Campanula alpina*^79^; several endemic taxa may be found within this part of the Eastern Carpathians^8,9^. Two other haplotypes emerged from haplotype E1: clusters F and G. The ancestral area of both these clusters lies in the Eastern Carpathians. Since the admixture of the F and G haplotypes is quite high, the plausible scenario may involve the emergence of the two haplotypes in isolated centers and then subsequent colonization from these two points. The two clusters also reached the Western Carpathians. Plants in cluster G are more widespread and have colonized the southern parts of the Carpathians to a greater degree. Interestingly, parts of the Western Carpathians like the Żywiec Beskids Mountains and the Gorce Mountains could have been colonized by a single dispersal event since their haplotype constitution is quite homogenous.

Consequently, the emergence of new haplotypes may not reflect an ecological pattern or speciation, but migration through the Carpathian arc accompanied by isolation events. Overall linear migration, which may be compared to a stepping-stone model, is supported for movement across the whole mountain range, with the exception of the emergence of several new haplotypes that follow the island model. Linear migration from south to north and east to west is also supported by the lack of occupation of or extreme scarcity of records for other suitable mountain ranges lying along the margins of the Western Carpathians, such as the Malá Fatra Mountains and Silesian Beskids Mountains. This further supports the hypothesis of migration and explains the absence of *L. rotundifolium* in these mountain ranges as time-dependent: in other words, enough time has not elapsed for this species to reach these ranges because it conquered the Western Carpathians only recently.

### Implications for the phylogeography of the Carpathians

*Leucanthemum rotundifolium* exemplifies the colonization of the Carpathians along the arc from the south to the east, and then to the north and the west. This route has also been demonstrated for several other species^9^. Similar to our results, Šrámková et al.^81^ found a link between the Dinarides and the Southern Carpathians. In addition, *Heliosperma pusillum* (Waldst. & Kit.) Rchb. populations from the Dinarides and the Southern Carpathians seem to be related^78^. Several species show this distributional pattern and this route should be emphasized as one of the major routes via which the Carpathian Mountains were colonized. However, the agents that enabled this migration or facilitated long-range dispersal are unknown. The timing of the arrival from the Dinarides to the Carpathians is not certain; in this study, it was reconstructed as occurring 0.38 Ma (CI 0.70–0.21) or 0.66 Ma (CI 0.87–0.48). For this node, the first dating is arguably more accurate since it included more species and was not affected by the polytomy. This scenario receives further support from a climate perspective; hypothetical ancestors of *L. rotundifolium* and similar cold-dwelling species expanded their ranges during the Elster glaciation (Marine Isotope Stage 12, 0.478–0.424 Ma). This cold period was followed by the Holstein interglacial (Marine Isotope Stage 11, 0.424–0.374 Ma), which was also one of the longest interglacial periods that could break previously established connections and separate colonizers from source populations.

According to Hurdu et al.^7^ and Ronikier^9^, the distribution of haplotypes within the Carpathians follows a pattern characterized by a high proportion of endemic species. This further confirms the results of this study and previously inferred floristic barriers and refugial areas^7^ it has limited potential to support or delineate biogeographic regions within the Carpathians. This is especially the case for the border between the Western and Eastern Carpathians, which is often considered in phylogeographic studies^3,9^. This study found that this border is porous, and during cooler periods, migration between the two mountain chains was possible. The likely ecological characteristics of the species play a significant role: alpine species confined to higher elevations, which represent the most frequently studied group, do not have many chances to migrate across a border composed of hills and low mountains that may act as a bridge only during the coldest phases. In contrast, mid-elevation montane species such as *L. rotundifolium* have more opportunities to establish at lower elevations sooner and require only moderate cooling to start migration. The existence of corridors or areas suitable for montane species at lower elevations is also supported by fossil data^82^. Another established border located between the Eastern and the Southern Carpathians is stronger in a phylogeographical sense (i.e., without populations with mixed haplotypes), but difficult to define geographically. For *L. rotundifolium*, the border is located somewhere between the Călimani Mountains and the Harghita Mountains, but it is difficult to pinpoint any feature related to a valley or river. The exact placement of this border was also difficult for the Orthoptera because a strict boundary between the Eastern and Southern Carpathians was not clear^83^.

## Conclusions and future directions

This study presents a phylogeographical investigation based on chloroplast haplotypes of a Carpathian subendemic that may be regarded as an exemplar of seed dispersal. The results of this study broaden the understanding of *L. rotundifolium* distribution and shed light on possible migration routes within the Carpathian Mountains.

It is worth mentioning that there are several other subendemic species in the Carpathians^14^ that may also provide interesting results and generate further useful hypotheses about migration.

## Supplementary Information

Supplementary Information 1.

Supplementary Information 2.

Supplementary Information 3.
