# Supplementary material for: Phylogeography and colonization pattern of subendemic round-leaved oxeye daisy from the Dinarides to the Carpathians
Source: Sci Rep. 2022 Sep 30;12:16443. doi: 10.1038/s41598-022-19619-1 (PMC9525303; doi:10.1038/s41598-022-19619-1)
Supplement: Supplementary file 2 — Supplementary Information 2. [file 41598_2022_19619_MOESM2_ESM.docx]

**Supplementary material 2. Aligments of the DNA sequences used in this study.**

**Title: Phylogeography and colonization pattern of subendemic round-leaved oxeye daisy from the Dinarides to the Carpathians**

**Author: Kamil Konowalik**

#trnT(GUU)-comp-psbD-comp alignment

#NEXUS

BEGIN DATA;

dimensions ntax=256 nchar=939;

format missing=N

symbols="ABCDEFGHIKLMNOPQRSTUVWXYZ"

datatype=NUCLEOTIDE gap=- match=.;

matrix

001_20160904_02_01_trnT(GUU)-comp-psbD-comp CATAGAGATGAAATTGGAACAAGTTGACCCCCTTTTCTATTTATTTTCATTTCTTTGGACTCCGCAAGAATTTGCCGATATTTCCAATTCAATCGTCTTGTCTTGTTCCTAGATGTTCTATAGGAAAAAATTGTCATTTCGTTCCTCTACGGAGAACCTTTTATTCTAAATTCTAAATCACAGGATCTAAATTCTAAATCACAGGATAAGAAAAAAATTCACTATCTTTCTTTGATTACAGGATCAAGATTCATTTANNNNTTATCTATACTATATAATATATTCNNNNNNTATAGATATTTCTATTCTATATTTATATTTATAGCTATCAGATCGTGGCTTGATGTACCAAAAATTTCCATTTCGTTGCATCCAATATTTTTGTTCCGACCATCGTATGAAGAAAGCATGCAAGATAAATACTATCATTTCCAATCTCCTATTTTAATTTTAATATTGTANNNNNNTTGAAGTGAAGTAAAAATTTGGAAACCCTCTCTTTTCTAACAGGGAAAAATAAATCAAAAAATATTAGTAATTTAGTCTACATAAAAATTAGAATATAAAGAGAGTTCTTTTTCTTAATCTCATGAACAAGATCTAAGAATCCATTTAGTTGATGAAAGAACATGGGGGGGGGCAGGCCTGAGGATCAACCGGTAGTGGGTNNNNGGAAGGGGATTGCTTTTTCCTTGACCAATTCTTTCAAAAAACGAATCGGATTCATGGGTCATAATAAGATAATTCATGATTCAGATGCTTAATAATAAGATAATAAGAAGGAATAATCAAATTGAATTCATGAATTTACCCGGCAATTTATGGTCCAATAAAGGATTTTTATCTTCGAAACCCATTGGAAGGGGTAGTGCACGAGAAAAAAAATCATGCGGAAATGATCGACTCCTTGGATGCCCCAAAATACTATGAGTGTCGGAA

002_20160908_01_01_trnT(GUU)-comp-psbD-comp CATAGAGATGAAATTGGAACAAGTTGACCCCCTTTTCTATTTATTTTCATTTCTTTGGACTCCGCAAGAATTTGCCGATATTTCCAATTCAATCGTCTTGTCTTGTTCCTAGATGTTCTATAGGAAAAAATTGTCATTTCGTTCCTCTACGGAGAACCTTTTATTCTAAATTCTAAATCACAGGATCTAAATTCTAAATCACAGGATAAGAAAAAAATTCACTATCTTTCTTTGATTACAGGATCAAGATTCATTTANNNNTTATCTATACTATATAATATATTCNNNNNNTATAGATATTTCTATTCTATATTTATATTTATAGCTATCAGATCGTGGCTTGATGTACCAAAAATTTCCATTTCGTTGCATCCAATATTTTTGTTCCGACCATCGTATGAAGAAAGCATGCAAGATAAATACTATCATTTCCAATCTCCTATTTTAATTTTAATATTGTANNNNNNTTGAAGTGAAGTAAAAATTTGGAAACCCTCTCTTTTCTAACAGGGAAAAATAAATCAAAAAATATTAGTAATTTAGTCTACATAAAAATTAGAATATAAAGAGAGTTCTTTTTCTTAATCTCATGAACAAGATCTAAGAATCCATTTAGTTGATGAAAGAACATGGGGGGGGGCAGGCCTGAGGATCAACCGGTAGTGGGTNNNNGGAAGGGGATTGCTTTTTCCTTGACCAATTCTTTCAAAAAACGAATCGGATTCATGGGTCATAATAAGATAATTCATGATTCAGATGCTTAATAATAAGATAATAAGAAGGAATAATCAAATTGAATTCATGAATTTACCCGGCAATTTATGGTCCAATAAAGGATTTTTATCTTCGAAACCCATTGGAAGGGGTAGTGCACGAGAAAAAAAATCATGCGGAAATGATCGACTCCTTGGATGCCCCAAAATACTATGAGTGTCGGAA

003_20160908_01_02_trnT(GUU)-comp-psbD-comp CATAGAGATGAAATTGGAACAAGTTGACCCCCTTTTCTATTTATTTTCATTTCTTTGGACTCCGCAAGAATTTGCCGATATTTCCAATTCAATCGTCTTGTCTTGTTCCTAGATGTTCTATAGGAAAAAATTGTCATTTCGTTCCTCTACGGAGAACCTTTTATTCTAAATTCTAAATCACAGGATCTAAATTCTAAATCACAGGATAAGAAAAAAATTCACTATCTTTCTTTGATTACAGGATCAAGATTCATTTANNNNTTATCTATACTATATAATATATTCNNNNNNTATAGATATTTCTATTCTATATTTATATTTATAGCTATCAGATCGTGGCTTGATGTACCAAAAATTTCCATTTCGTTGCATCCAATATTTTTGTTCCGACCATCGTATGAAGAAAGCATGCAAGATAAATACTATCATTTCCAATCTCCTATTTTAATTTTAATATTGTANNNNNNTTGAAGTGAAGTAAAAATTTGGAAACCCTCTCTTTTCTAACAGGGAAAAATAAATCAAAAAATATTAGTAATTTAGTCTACATAAAAATTAGAATATAAAGAGAGTTCTTTTTCTTAATCTCATGAACAAGATCTAAGAATCCATTTAGTTGATGAAAGAACATGGGGGGGGGCAGGCCTGAGGATCAACCGGTAGTGGGTNNNNGGAAGGGGATTGCTTTTTCCTTGACCAATTCTTTCAAAAAACGAATCGGATTCATGGGTCATAATAAGATAATTCATGATTCAGATGCTTAATAATAAGATAATAAGAAGGAATAATCAAATTGAATTCATGAATTTACCCGGCAATTTATGGTCCAATAAAGGATTTTTATCTTCGAAACCCATTGGAAGGGGTAGTGCACGAGAAAAAAAATCATGCGGAAATGATCGACTCCTTGGATGCCCCAAAATACTATGAGTGTCGGAA

004_20160908_01_03_trnT(GUU)-comp-psbD-comp CATAGAGATGAAATTGGAACAAGTTGACCCCCTTTTCTATTTATTTTCATTTCTTTGGACTCCGCAAGAATTTGCCGATATTTCCAATTCAATCGTCTTGTCTTGTTCCTAGATGTTCTATAGGAAAAAATTGTCATTTCGTTCCTCTACGGAGAACCTTTTATTCTAAATTCTAAATCACAGGATCTAAATTCTAAATCACAGGATAAGAAAAAAATTCACTATCTTTCTTTGATTACAGGATCAAGATTCATTTANNNNTTATCTATACTATATAATATATTCNNNNNNTATAGATATTTCTATTCTATATTTATATTTATAGCTATCAGATCGTGGCTTGATGTACCAAAAATTTCCATTTCGTTGCATCCAATATTTTTGTTCCGACCATCGTATGAAGAAAGCATGCAAGATAAATACTATCATTTCCAATCTCCTATTTTAATTTTAATATTGTANNNNNNTTGAAGTGAAGTAAAAATTTGGAAACCCTCTCTTTTCTAACAGGGAAAAATAAATCAAAAAATATTAGTAATTTAGTCTACATAAAAATTAGAATATAAAGAGAGTTCTTTTTCTTAATCTCATGAACAAGATCTAAGAATCCATTTAGTTGATGAAAGAACATGGGGGGGGGCAGGCCTGAGGATCAACCGGTAGTGGGTNNNNGGAAGGGGATTGCTTTTTCCTTGACCAATTCTTTCAAAAAACGAATCGGATTCATGGGTCATAATAAGATAATTCATGATTCAGATGCTTAATAATAAGATAATAAGAAGGAATAATCAAATTGAATTCATGAATTTACCCGGCAATTTATGGTCCAATAAAGGATTTTTATCTTCGAAACCCATTGGAAGGGGTAGTGCACGAGAAAAAAAATCATGCGGAAATGATCGACTCCTTGGATGCCCCAAAATACTATGAGTGTCGGAA

005_20160908_01_04_trnT(GUU)-comp-psbD-comp CATAGAGATGAAATTGGAACAAGTTGACCCCCTTTTCTATTTATTTTCATTTCTTTGGACTCCGCAAGAATTTGCCGATATTTCCAATTCAATCGTCTTGTCTTGTTCCTAGATGTTCTATAGGAAAAAATTGTCATTTCGTTCCTCTACGGAGAACCTTTTATTCTAAATTCTAAATCACAGGATCTAAATTCTAAATCACAGGATAAGAAAAAAATTCACTATCTTTCTTTGATTACAGGATCAAGATTCATTTANNNNTTATCTATACTATATAATATATTCNNNNNNTATAGATATTTCTATTCTATATTTATATTTATAGCTATCAGATCGTGGCTTGATGTACCAAAAATTTCCATTTCGTTGCATCCAATATTTTTGTTCCGACCATCGTATGAAGAAAGCATGCAAGATAAATACTATCATTTCCAATCTCCTATTTTAATTTTAATATTGTANNNNNNTTGAAGTGAAGTAAAAATTTGGAAACCCTCTCTTTTCTAACAGGGAAAAATAAATCAAAAAATATTAGTAATTTAGTCTACATAAAAATTAGAATATAAAGAGAGTTCTTTTTCTTAATCTCATGAACAAGATCTAAGAATCCATTTAGTTGATGAAAGAACATGGGGGGGGGCAGGCCTGAGGATCAACCGGTAGTGGGTNNNNGGAAGGGGATTGCTTTTTCCTTGACCAATTCTTTCAAAAAACGAATCGGATTCATGGGTCATAATAAGATAATTCATGATTCAGATGCTTAATAATAAGATAATAAGAAGGAATAATCAAATTGAATTCATGAATTTACCCGGCAATTTATGGTCCAATAAAGGATTTTTATCTTCGAAACCCATTGGAAGGGGTAGTGCACGAGAAAAAAAATCATGCGGAAATGATCGACTCCTTGGATGCCCCAAAATACTATGAGTGTCGGAA

006_20160908_01_05_trnT(GUU)-comp-psbD-comp CATAGAGATGAAATTGGAACAAGTTGACCCCCTTTTCTATTTATTTTCATTTCTTTGGACTCCGCAAGAATTTGCCGATATTTCCAATTCAATCGTCTTGTCTTGTTCCTAGATGTTCTATAGGAAAAAATTGTCATTTCGTTCCTCTACGGAGAACCTTTTATTCTAAATTCTAAATCACAGGATCTAAATTCTAAATCACAGGATAAGAAAAAAATTCACTATCTTTCTTTGATTACAGGATCAAGATTCATTTANNNNTTATCTATACTATATAATATATTCNNNNNNTATAGATATTTCTATTCTATATTTATATTTATAGCTATCAGATCGTGGCTTGATGTACCAAAAATTTCCATTTCGTTGCATCCAATATTTTTGTTCCGACCATCGTATGAAGAAAGCATGCAAGATAAATACTATCATTTCCAATCTCCTATTTTAATTTTAATATTGTANNNNNNTTGAAGTGAAGTAAAAATTTGGAAACCCTCTCTTTTCTAACAGGGAAAAATAAATCAAAAAATATTAGTAATTTAGTCTACATAAAAATTAGAATATAAAGAGAGTTCTTTTTCTTAATCTCATGAACAAGATCTAAGAATCCATTTAGTTGATGAAAGAACATGGGGGGGGGCAGGCCTGAGGATCAACCGGTAGTGGGTNNNNGGAAGGGGATTGCTTTTTCCTTGACCAATTCTTTCAAAAAACGAATCGGATTCATGGGTCATAATAAGATAATTCATGATTCAGATGCTTAATAATAAGATAATAAGAAGGAATAATCAAATTGAATTCATGAATTTACCCGGCAATTTATGGTCCAATAAAGGATTTTTATCTTCGAAACCCATTGGAAGGGGTAGTGCACGAGAAAAAAAATCATGCGGAAATGATCGACTCCTTGGATGCCCCAAAATACTATGAGTGTCGGAA

007_20160909_02_01_trnT(GUU)-comp-psbD-comp CATAGAGATGAAATTGGAACAAGTTGACCCCCTTTTCTATTTATTTTCATTTCTTTGGACTCCGCAAGAATTTGCCGATATTTCCAATTCAATCGTCTTGTCTTGTTCCTAGATGTTCTATAGGAAAAAATTGTCATTTCGTTCCTCTACGGAGAACCTTTTATTCTAAATTCTAAATCACAGGATCTAAATTCTAAATCACAGGATAAGAAAAAAATTCACTATCTTTCTTTGATTACAGGATCAAGATTCATTTANNNNTTATCTATACTATATAATATATTCNNNNNNTATAGATATTTCTATTCTATATTTATATTTATAGCTATCAGATCGTGGCTTGATGTACCAAAAATTTCCATTTCGTTGCATCCAATATTTTTGTTCCGACCATCGTATGAAGAAAGCATGCAAGATAAATACTATCATTTCCAATCTCCTATTTTAATTTTAATATTGTANNNNNNTTGAAGTGAAGTAAAAATTTGGAAACCCTCTCTTTTCTAACAGGGAAAAATAAATCAAAAAATATTAGTAATTTAGTCTACATAAAAATTAGAATATAAAGAGAGTTCTTTTTCTTAATCTCATGAACAAGATCTAAGAATCCATTTAGTTGATGAAAGAACATGGGGGGGGGCAGGCCTGAGGATCAACCGGTAGTGGGTNNNNGGAAGGGGATTGCTTTTTCCTTGACCAATTCTTTCAAAAAACGAATCGGATTCATGGGTCATAATAAGATAATTCATGATTCAGATGCTTAATAATAAGATAATAAGAAGGAATAATCAAATTGAATTCATGAATTTACCCGGCAATTTATGGTCCAATAAAGGATTTTTATCTTCGAAACCCATTGGAAGGGGTAGTGCACGAGAAAAAAAATCATGCGGAAATGATCGACTCCTTGGATGCCCCAAAATACTATGAGTGTCGGAA

008_20160910_01_01_trnT(GUU)-comp-psbD-comp CATAGAGATGAAATTGGAACAAGTTGACCCCCTTTTCTATTTATTTTCATTTCTTTGGACTCCGCAAGAATTTGCCGATATTTCCAATTCAATCGTCTTGTCTTGTTCCTAGATGTTCTATAGGAAAAAATTGTCATTTCGTTCCTCTACGGAGAACCTTTTATTCTAAATTCTAAATCACAGGATCTAAATTCTAAATCACAGGATAAGAAAAAAATTCACTATCTTTCTTTGATTACAGGATCAAGATTCATTTANNNNTTATCTATACTATATAATATATTCNNNNNNTATAGATATTTCTATTCTATATTTATATTTATAGCTATCAGATCGTGGCTTGATGTACCAAAAATTTCCATTTCGTTGCATCCAATATTTTTGTTCCGACCATCGTATGAAGAAAGCATGCAAGATAAATACTATCATTTCCAATCTCCTATTTTAATTTTAATATTGTANNNNNNTTGAAGTGAAGTAAAAATTTGGAAACCCTCTCTTTTCTAACAGGGAAAAATAAATCAAAAAATATTAGTAATTTAGTCTACATAAAAATTAGAATATAAAGAGAGTTCTTTTTCTTAATCTCATGAACAAGATCTAAGAATCCATTTAGTTGATGAAAGAACATGGGGGGGGGCAGGCCTGAGGATCAACCGGTAGTGGGTNNNNGGAAGGGGATTGCTTTTTCCTTGACCAATTCTTTCAAAAAACGAATCGGATTCATGGGTCATAATAAGATAATTCATGATTCAGATGCTTAATAATAAGATAATAAGAAGGAATAATCAAATTGAATTCATGAATTTACCCGGCAATTTATGGTCCAATAAAGGATTTTTATCTTCGAAACCCATTGGAAGGGGTAGTGCACGAGAAAAAAAATCATGCGGAAATGATCGACTCCTTGGATGCCCCAAAATACTATGAGTGTCGGAA

009_20170910_07_01_trnT(GUU)-comp-psbD-comp CATAGAGATGAAATTGGAACAAGTTGACCCCCTTTTCTATTTATTTTCATTTCTTTGGACTCCGCAAGAATTTGCCGATATTTCCAATTCAATCGTCTTGTCTTGTTCCTAGATGTTCTATAGGAAAAAATTGTCATTTCGTTCCTCTACGGAGAACCTTTTATTCTAAATTCTAAATCACAGGATCTAAATTCTAAATCACAGGATAAGAAAAAAATTCACTATCTTTCTTTGATTACAGGATCAAGATTCATTTANNNNTTATCTATACTATATAATATATTCNNNNNNTATAGATATTTCTATTCTATATTTATATTTATAGCTATCAGATCGTGGCTTGATGTACCAAAAATTTCCATTTCGTTGCATCCAATATTTTTGTTCCGACCATCGTATGAAGAAAGCATGCAAGATAAATACTATCATTTCCAATCTCCTATTTTAATTTTAATATTGTANNNNNNTTGAAGTGAAGTAAAAATTTGGAAACCCTCTCTTTTCTAACAGGGAAAAATAAATCAAAAAATATTAGTAATTTAGTCTACATAAAAATTAGAATATAAAGAGAGTTCTTTTTCTTAATCTCATGAACAAGATCTAAGAATCCATTTAGTTGATGAAAGAACATGGGGGGGGGCAGGCCTGAGGATCAACCGGTAGTGGGTNNNNGGAAGGGGATTGCTTTTTCCTTGACCAATTCTTTCAAAAAACGAATCGGATTCATGGGTCATAATAAGATAATTCATGATTCAGATGCTTAATAATAAGATAATAAGAAGGAATAATCAAATTGAATTCATGAATTTACCCGGCAATTTATGGTCCAATAAAGGATTTTTATCTTCGAAACCCATTGGAAGGGGTAGTGCACGAGAAAAAAAATCATGCGGAAATGATCGACTCCTTGGATGCCCCAAAATACTATGAGTGTCGGAA

010_20170910_07_02_trnT(GUU)-comp-psbD-comp CATAGAGATGAAATTGGAACAAGTTGACCCCCTTTTCTATTTATTTTCATTTCTTTGGACTCCGCAAGAATTTGCCGATATTTCCAATTCAATCGTCTTGTCTTGTTCCTAGATGTTCTATAGGAAAAAATTGTCATTTCGTTCCTCTACGGAGAACCTTTTATTCTAAATTCTAAATCACAGGATCTAAATTCTAAATCACAGGATAAGAAAAAAATTCACTATCTTTCTTTGATTACAGGATCAAGATTCATTTANNNNTTATCTATACTATATAATATATTCNNNNNNTATAGATATTTCTATTCTATATTTATATTTATAGCTATCAGATCGTGGCTTGATGTACCAAAAATTTCCATTTCGTTGCATCCAATATTTTTGTTCCGACCATCGTATGAAGAAAGCATGCAAGATAAATACTATCATTTCCAATCTCCTATTTTAATTTTAATATTGTANNNNNNTTGAAGTGAAGTAAAAATTTGGAAACCCTCTCTTTTCTAACAGGGAAAAATAAATCAAAAAATATTAGTAATTTAGTCTACATAAAAATTAGAATATAAAGAGAGTTCTTTTTCTTAATCTCATGAACAAGATCTAAGAATCCATTTAGTTGATGAAAGAACATGGGGGGGGGCAGGCCTGAGGATCAACCGGTAGTGGGTNNNNGGAAGGGGATTGCTTTTTCCTTGACCAATTCTTTCAAAAAACGAATCGGATTCATGGGTCATAATAAGATAATTCATGATTCAGATGCTTAATAATAAGATAATAAGAAGGAATAATCAAATTGAATTCATGAATTTACCCGGCAATTTATGGTCCAATAAAGGATTTTTATCTTCGAAACCCATTGGAAGGGGTAGTGCACGAGAAAAAAAATCATGCGGAAATGATCGACTCCTTGGATGCCCCAAAATACTATGAGTGTCGGAA

011_20170910_07_03_trnT(GUU)-comp-psbD-comp CATAGAGATGAAATTGGAACAAGTTGACCCCCTTTTCTATTTATTTTCATTTCTTTGGACTCCGCAAGAATTTGCCGATATTTCCAATTCAATCGTCTTGTCTTGTTCCTAGATGTTCTATAGGAAAAAATTGTCATTTCGTTCCTCTACGGAGAACCTTTTATTCTAAATTCTAAATCACAGGATCTAAATTCTAAATCACAGGATAAGAAAAAAATTCACTATCTTTCTTTGATTACAGGATCAAGATTCATTTANNNNTTATCTATACTATATAATATATTCNNNNNNTATAGATATTTCTATTCTATATTTATATTTATAGCTATCAGATCGTGGCTTGATGTACCAAAAATTTCCATTTCGTTGCATCCAATATTTTTGTTCCGACCATCGTATGAAGAAAGCATGCAAGATAAATACTATCATTTCCAATCTCCTATTTTAATTTTAATATTGTANNNNNNTTGAAGTGAAGTAAAAATTTGGAAACCCTCTCTTTTCTAACAGGGAAAAATAAATCAAAAAATATTAGTAATTTAGTCTACATAAAAATTAGAATATAAAGAGAGTTCTTTTTCTTAATCTCATGAACAAGATCTAAGAATCCATTTAGTTGATGAAAGAACATGGGGGGGGGCAGGCCTGAGGATCAACCGGTAGTGGGTNNNNGGAAGGGGATTGCTTTTTCCTTGACCAATTCTTTCAAAAAACGAATCGGATTCATGGGTCATAATAAGATAATTCATGATTCAGATGCTTAATAATAAGATAATAAGAAGGAATAATCAAATTGAATTCATGAATTTACCCGGCAATTTATGGTCCAATAAAGGATTTTTATCTTCGAAACCCATTGGAAGGGGTAGTGCACGAGAAAAAAAATCATGCGGAAATGATCGACTCCTTGGATGCCCCAAAATACTATGAGTGTCGGAA

012_20170910_07_04_trnT(GUU)-comp-psbD-comp CATAGAGATGAAATTGGAACAAGTTGACCCCCTTTTCTATTTATTTTCATTTCTTTGGACTCCGCAAGAATTTGCCGATATTTCCAATTCAATCGTCTTGTCTTGTTCCTAGATGTTCTATAGGAAAAAATTGTCATTTCGTTCCTCTACGGAGAACCTTTTATNNNNNNNNNNNNNNNNNNNNNTCTAAATTCTAAATCACAGGATAAGAAAAAAATTCACTATCTTTCTTTGATTACAGGATCAAGATTCATTTATATCTTATCTATACTATATAATATATTCNNNNNNTATAGATATTTCTATTCTATATTTAGATTTATAGCTATCAGATCGTGGCTTGATGTACCAAAAATTTCCATTTCGTTGCATCCAATATTTTTGTTCCGACCATCGTATGAAGAAAGCATGCAAGATAAATACTATCATTTCCAATCTCCTATTTTAATTTTAATATTGTANNNNNNTTGAAGTGAAGTAAAAATTTGGAAACCCTCTCTTTTCTAACAGGGAAAAATAAATCAAAAAATATTAGTAATTTAGTCTACATAAAAATTAGAATATAAAGAGAGTTCTTTTTCTTAATCTCATGAACAAGATCTAAGAATCCATTTAGTTGATGAAAGAACATGGGGGGGGGCAGGCCTGAGGATCAACCGGTAGTGGGTNNNNGGAAGGGGATTGCTTTTTCCTTGACCAATTCTTTCAAAAAACGAATCGGATTCATGGGTCATAATAAGATAATTCATGATTCAGATGCTTAATAATAAGATAATAAGAAGGAATAATCAAATTGAATTCATGAATTTACCCGGCAATTTATGGTCCAATAAAGGATTTTTATCTTCGAAACCCATTGGAAGGGGTAGTGCACGAGAAAAAAAATCATGCGGAAATGATCGACTCCTTGGATGCCCCAAAATACTATGAGTGTCGGAA

013_20170910_07_05_trnT(GUU)-comp-psbD-comp CATAGAGATGAAATTGGAACAAGTTGACCCCCTTTTCTATTTATTTTCATTTCTTTGGACTCCGCAAGAATTTGCCGATATTTCCAATTCAATCGTCTTGTCTTGTTCCTAGATGTTCTATAGGAAAAAATTGTCATTTCGTTCCTCTACGGAGAACCTTTTATNNNNNNNNNNNNNNNNNNNNNTCTAAATTCTAAATCACAGGATAAGAAAAAAATTCACTATCTTTCTTTGATTACAGGATCAAGATTCATTTATATCTTATCTATACTATATAATATATTCNNNNNNTATAGATATTTCTATTCTATATTTAGATTTATAGCTATCAGATCGTGGCTTGATGTACCAAAAATTTCCATTTCGTTGCATCCAATATTTTTGTTCCGACCATCGTATGAAGAAAGCATGCAAGATAAATACTATCATTTCCAATCTCCTATTTTAATTTTAATATTGTANNNNNNTTGAAGTGAAGTAAAAATTTGGAAACCCTCTCTTTTCTAACAGGGAAAAATAAATCAAAAAATATTAGTAATTTAGTCTACATAAAAATTAGAATATAAAGAGAGTTCTTTTTCTTAATCTCATGAACAAGATCTAAGAATCCATTTAGTTGATGAAAGAACATGGGGGGGGGCAGGCCTGAGGATCAACCGGTAGTGGGTNNNNGGAAGGGGATTGCTTTTTCCTTGACCAATTCTTTCAAAAAACGAATCGGATTCATGGGTCATAATAAGATAATTCATGATTCAGATGCTTAATAATAAGATAATAAGAAGGAATAATCAAATTGAATTCATGAATTTACCCGGCAATTTATGGTCCAATAAAGGATTTTTATCTTCGAAACCCATTGGAAGGGGTAGTGCACGAGAAAAAAAATCATGCGGAAATGATCGACTCCTTGGATGCCCCAAAATACTATGAGTGTCGGAA

014_20170916_04_01_trnT(GUU)-comp-psbD-comp CATAGAGATGAAATTGGAACAAGTTGACCCCCTTTTCTATTTATTTTCATTTCTTTGGACTCCGCAAGAATTTGCCGATATTTCCAATTCAATCGTCTTGTCTTGTTCCTAGATGTTCTATAGGAAAAAATTGTCATTTCGTTCCTCTACGGAGAACCTTTTATTCTAAATTCTAAATCACAGGATCTAAATTCTAAATCACAGGATAAGAAAAAAATTCACTATCTTTCTTTGATTACAGGATCAAGATTCATTTANNNNTTATCTATACTATATAATATATTCNNNNNNTATAGATATTTCTATTCTATATTTATATTTATAGCTATCAGATCGTGGCTTGATGTACCAAAAATTTCCATTTCGTTGCATCCAATATTTTTGTTCCGACCATCGTATGAAGAAAGCATGCAAGATAAATACTATCATTTCCAATCTCCTATTTTAATTTTAATATTGTANNNNNNTTGAAGTGAAGTAAAAATTTGGAAACCCTCTCTTTTCTAACAGGGAAAAATAAATCAAAAAATATTAGTAATTTAGTCTACATAAAAATTAGAATATAAAGAGAGTTCTTTTTCTTAATCTCATGAACAAGATCTAAGAATCCATTTAGTTGATGAAAGAACATGGGGGGGGGCAGGCCTGAGGATCAACCGGTAGTGGGTNNNNGGAAGGGGATTGCTTTTTCCTTGACCAATTCTTTCAAAAAACGAATCGGATTCATGGGTCATAATAAGATAATTCATGATTCAGATGCTTAATAATAAGATAATAAGAAGGAATAATCAAATTGAATTCATGAATTTACCCGGCAATTTATGGTCCAATAAAGGATTTTTATCTTCGAAACCCATTGGAAGGGGTAGTGCACGAGAAAAAAAATCATGCGGAAATGATCGACTCCTTGGATGCCCCAAAATACTATGAGTGTCGGAA

015_20170916_04_02_trnT(GUU)-comp-psbD-comp CATAGAGATGAAATTGGAACAAGTTGACCCCCTTTTCTATTTATTTTCATTTCTTTGGACTCCGCAAGAATTTGCCGATATTTCCAATTCAATCGTCTTGTCTTGTTCCTAGATGTTCTATAGGAAAAAATTGTCATTTCGTTCCTCTACGGAGAACCTTTTATTCTAAATTCTAAATCACAGGATCTAAATTCTAAATCACAGGATAAGAAAAAAATTCACTATCTTTCTTTGATTACAGGATCAAGATTCATTTANNNNTTATCTATACTATATAATATATTCNNNNNNTATAGATATTTCTATTCTATATTTATATTTATAGCTATCAGATCGTGGCTTGATGTACCAAAAATTTCCATTTCGTTGCATCCAATATTTTTGTTCCGACCATCGTATGAAGAAAGCATGCAAGATAAATACTATCATTTCCAATCTCCTATTTTAATTTTAATATTGTANNNNNNTTGAAGTGAAGTAAAAATTTGGAAACCCTCTCTTTTCTAACAGGGAAAAATAAATCAAAAAATATTAGTAATTTAGTCTACATAAAAATTAGAATATAAAGAGAGTTCTTTTTCTTAATCTCATGAACAAGATCTAAGAATCCATTTAGTTGATGAAAGAACATGGGGGGGGGCAGGCCTGAGGATCAACCGGTAGTGGGTNNNNGGAAGGGGATTGCTTTTTCCTTGACCAATTCTTTCAAAAAACGAATCGGATTCATGGGTCATAATAAGATAATTCATGATTCAGATGCTTAATAATAAGATAATAAGAAGGAATAATCAAATTGAATTCATGAATTTACCCGGCAATTTATGGTCCAATAAAGGATTTTTATCTTCGAAACCCATTGGAAGGGGTAGTGCACGAGAAAAAAAATCATGCGGAAATGATCGACTCCTTGGATGCCCCAAAATACTATGAGTGTCGGAA

016_20170916_04_03_trnT(GUU)-comp-psbD-comp CATAGAGATGAAATTGGAACAAGTTGACCCCCTTTTCTATTTATTTTCATTTCTTTGGACTCCGCAAGAATTTGCCGATATTTCCAATTCAATCGTCTTGTCTTGTTCCTAGATGTTCTATAGGAAAAAATTGTCATTTCGTTCCTCTACGGAGAACCTTTTATTCTAAATTCTAAATCACAGGATCTAAATTCTAAATCACAGGATAAGAAAAAAATTCACTATCTTTCTTTGATTACAGGATCAAGATTCATTTANNNNTTATCTATACTATATAATATATTCNNNNNNTATAGATATTTCTATTCTATATTTATATTTATAGCTATCAGATCGTGGCTTGATGTACCAAAAATTTCCATTTCGTTGCATCCAATATTTTTGTTCCGACCATCGTATGAAGAAAGCATGCAAGATAAATACTATCATTTCCAATCTCCTATTTTAATTTTAATATTGTANNNNNNTTGAAGTGAAGTAAAAATTTGGAAACCCTCTCTTTTCTAACAGGGAAAAATAAATCAAAAAATATTAGTAATTTAGTCTACATAAAAATTAGAATATAAAGAGAGTTCTTTTTCTTAATCTCATGAACAAGATCTAAGAATCCATTTAGTTGATGAAAGAACATGGGGGGGGGCAGGCCTGAGGATCAACCGGTAGTGGGTNNNNGGAAGGGGATTGCTTTTTCCTTGACCAATTCTTTCAAAAAACGAATCGGATTCATGGGTCATAATAAGATAATTCATGATTCAGATGCTTAATAATAAGATAATAAGAAGGAATAATCAAATTGAATTCATGAATTTACCCGGCAATTTATGGTCCAATAAAGGATTTTTATCTTCGAAACCCATTGGAAGGGGTAGTGCACGAGAAAAAAAATCATGCGGAAATGATCGACTCCTTGGATGCCCCAAAATACTATGAGTGTCGGAA

017_20170916_05_01_trnT(GUU)-comp-psbD-comp CATAGAGATGAAATTGGAACAAGTTGACCCCCTTTTCTATTTATTTTCATTTCTTTGGACTCCGCAAGAATTTGCCGATATTTCCAATTCAATCGTCTTGTCTTGTTCCTAGATGTTCTATAGGAAAAAATTGTCATTTCGTTCCTCTACGGAGAACCTTTTATTCTAAATTCTAAATCACAGGATCTAAATTCTAAATCACAGGATAAGAAAAAAATTCACTATCTTTCTTTGATTACAGGATCAAGATTCATTTANNNNTTATCTATACTATATAATATATTCNNNNNNTATAGATATTTCTATTCTATATTTATATTTATAGCTATCAGATCGTGGCTTGATGTACCAAAAATTTCCATTTCGTTGCATCCAATATTTTTGTTCCGACCATCGTATGAAGAAAGCATGCAAGATAAATACTATCATTTCCAATCTCCTATTTTAATTTTAATATTGTANNNNNNTTGAAGTGAAGTAAAAATTTGGAAACCCTCTCTTTTCTAACAGGGAAAAATAAATCAAAAAATATTAGTAATTTAGTCTACATAAAAATTAGAATATAAAGAGAGTTCTTTTTCTTAATCTCATGAACAAGATCTAAGAATCCATTTAGTTGATGAAAGAACATGGGGGGGGGCAGGCCTGAGGATCAACCGGTAGTGGGTNNNNGGAAGGGGATTGCTTTTTCCTTGACCAATTCTTTCAAAAAACGAATCGGATTCATGGGTCATAATAAGATAATTCATGATTCAGATGCTTAATAATAAGATAATAAGAAGGAATAATCAAATTGAATTCATGAATTTACCCGGCAATTTATGGTCCAATAAAGGATTTTTATCTTCGAAACCCATTGGAAGGGGTAGTGCACGAGAAAAAAAATCATGCGGAAATGATCGACTCCTTGGATGCCCCAAAATACTATGAGTGTCGGAA

018_20170916_05_02_trnT(GUU)-comp-psbD-comp CATAGAGATGAAATTGGAACAAGTTGACCCCCTTTTCTATTTATTTTCATTTCTTTGGACTCCGCAAGAATTTGCCGATATTTCCAATTCAATCGTCTTGTCTTGTTCCTAGATGTTCTATAGGAAAAAATTGTCATTTCGTTCCTCTACGGAGAACCTTTTATTCTAAATTCTAAATCACAGGATCTAAATTCTAAATCACAGGATAAGAAAAAAATTCACTATCTTTCTTTGATTACAGGATCAAGATTCATTTANNNNTTATCTATACTATATAATATATTCNNNNNNTATAGATATTTCTATTCTATATTTATATTTATAGCTATCAGATCGTGGCTTGATGTACCAAAAATTTCCATTTCGTTGCATCCAATATTTTTGTTCCGACCATCGTATGAAGAAAGCATGCAAGATAAATACTATCATTTCCAATCTCCTATTTTAATTTTAATATTGTANNNNNNTTGAAGTGAAGTAAAAATTTGGAAACCCTCTCTTTTCTAACAGGGAAAAATAAATCAAAAAATATTAGTAATTTAGTCTACATAAAAATTAGAATATAAAGAGAGTTCTTTTTCTTAATCTCATGAACAAGATCTAAGAATCCATTTAGTTGATGAAAGAACATGGGGGGGGGCAGGCCTGAGGATCAACCGGTAGTGGGTNNNNGGAAGGGGATTGCTTTTTCCTTGACCAATTCTTTCAAAAAACGAATCGGATTCATGGGTCATAATAAGATAATTCATGATTCAGATGCTTAATAATAAGATAATAAGAAGGAATAATCAAATTGAATTCATGAATTTACCCGGCAATTTATGGTCCAATAAAGGATTTTTATCTTCGAAACCCATTGGAAGGGGTAGTGCACGAGAAAAAAAATCATGCGGAAATGATCGACTCCTTGGATGCCCCAAAATACTATGAGTGTCGGAA

019_20170916_06_01_trnT(GUU)-comp-psbD-comp CATAGAGATGAAATTGGAACAAGTTGACCCCCTTTTCTATTTATTTTCATTTCTTTGGACTCCGCAAGAATTTGCCGATATTTCCAATTCAATCGTCTTGTCTTGTTCCTAGATGTTCTATAGGAAAAAATTGTCATTTCGTTCCTCTACGGAGAACCTTTTATTCTAAATTCTAAATCACAGGATCTAAATTCTAAATCACAGGATAAGAAAAAAATTCACTATCTTTCTTTGATTACAGGATCAAGATTCATTTANNNNTTATCTATACTATATAATATATTCNNNNNNTATAGATATTTCTATTCTATATTTATATTTATAGCTATCAGATCGTGGCTTGATGTACCAAAAATTTCCATTTCGTTGCATCCAATATTTTTGTTCCGACCATCGTATGAAGAAAGCATGCAAGATAAATACTATCATTTCCAATCTCCTATTTTAATTTTAATATTGTANNNNNNTTGAAGTGAAGTAAAAATTTGGAAACCCTCTCTTTTCTAACAGGGAAAAATAAATCAAAAAATATTAGTAATTTAGTCTACATAAAAATTAGAATATAAAGAGAGTTCTTTTTCTTAATCTCATGAACAAGATCTAAGAATCCATTTAGTTGATGAAAGAACATGGGGGGGGGCAGGCCTGAGGATCAACCGGTAGTGGGTNNNNGGAAGGGGATTGCTTTTTCCTTGACCAATTCTTTCAAAAAACGAATCGGATTCATGGGTCATAATAAGATAATTCATGATTCAGATGCTTAATAATAAGATAATAAGAAGGAATAATCAAATTGAATTCATGAATTTACCCGGCAATTTATGGTCCAATAAAGGATTTTTATCTTCGAAACCCATTGGAAGGGGTAGTGCACGAGAAAAAAAATCATGCGGAAATGATCGACTCCTTGGATGCCCCAAAATACTATGAGTGTCGGAA

020_20170916_06_02_trnT(GUU)-comp-psbD-comp CATAGAGATGAAATTGGAACAAGTTGACCCCCTTTTCTATTTATTTTCATTTCTTTGGACTCCGCAAGAATTTGCCGATATTTCCAATTCAATCGTCTTGTCTTGTTCCTAGATGTTCTATAGGAAAAAATTGTCATTTCGTTCCTCTACGGAGAACCTTTTATTCTAAATTCTAAATCACAGGATCTAAATTCTAAATCACAGGATAAGAAAAAAATTCACTATCTTTCTTTGATTACAGGATCAAGATTCATTTANNNNTTATCTATACTATATAATATATTCNNNNNNTATAGATATTTCTATTCTATATTTATATTTATAGCTATCAGATCGTGGCTTGATGTACCAAAAATTTCCATTTCGTTGCATCCAATATTTTTGTTCCGACCATCGTATGAAGAAAGCATGCAAGATAAATACTATCATTTCCAATCTCCTATTTTAATTTTAATATTGTANNNNNNTTGAAGTGAAGTAAAAATTTGGAAACCCTCTCTTTTCTAACAGGGAAAAATAAATCAAAAAATATTAGTAATTTAGTCTACATAAAAATTAGAATATAAAGAGAGTTCTTTTTCTTAATCTCATGAACAAGATCTAAGAATCCATTTAGTTGATGAAAGAACATGGGGGGGGGCAGGCCTGAGGATCAACCGGTAGTGGGTNNNNGGAAGGGGATTGCTTTTTCCTTGACCAATTCTTTCAAAAAACGAATCGGATTCATGGGTCATAATAAGATAATTCATGATTCAGATGCTTAATAATAAGATAATAAGAAGGAATAATCAAATTGAATTCATGAATTTACCCGGCAATTTATGGTCCAATAAAGGATTTTTATCTTCGAAACCCATTGGAAGGGGTAGTGCACGAGAAAAAAAATCATGCGGAAATGATCGACTCCTTGGATGCCCCAAAATACTATGAGTGTCGGAA

021_20170917_02_01_trnT(GUU)-comp-psbD-comp CATAGAGATGAAATTGGAACAAGTTGACCCCCTTTTCTATTTATTTTCATTTCTTTGGACTCCGCAAGAATTTGCCGATATTTCCAATTCAATCGTCTTGTCTTGTTCCTAGATGTTCTATAGGAAAAAATTGTCATTTCGTTCCTCTACGGAGAACCTTTTATTCTAAATTCTAAATCACAGGATCTAAATTCTAAATCACAGGATAAGAAAAAAATTCACTATCTTTCTTTGATTACAGGATCAAGATTCATTTANNNNTTATCTATACTATATAATATATTCNNNNNNTATAGATATTTCTATTCTATATTTATATTTATAGCTATCAGATCGTGGCTTGATGTACCAAAAATTTCCATTTCGTTGCATCCAATATTTTTGTTCCGACCATCGTATGAAGAAAGCATGCAAGATAAATACTATCATTTCCAATCTCCTATTTTAATTTTAATATTGTANNNNNNTTGAAGTGAAGTAAAAATTTGGAAACCCTCTCTTTTCTAACAGGGAAAAATAAATCAAAAAATATTAGTAATTTAGTCTACATAAAAATTAGAATATAAAGAGAGTTCTTTTTCTTAATCTCATGAACAAGATCTAAGAATCCATTTAGTTGATGAAAGAACATGGGGGGGGGCAGGCCTGAGGATCAACCGGTAGTGGGTNNNNGGAAGGGGATTGCTTTTTCCTTGACCAATTCTTTCAAAAAACGAATCGGATTCATGGGTCATAATAAGATAATTCATGATTCAGATGCTTAATAATAAGATAATAAGAAGGAATAATCAAATTGAATTCATGAATTTACCCGGCAATTTATGGTCCAATAAAGGATTTTTATCTTCGAAACCCATTGGAAGGGGTAGTGCACGAGAAAAAAAATCATGCGGAAATGATCGACTCCTTGGATGCCCCAAAATACTATGAGTGTCGGAA

022_20170917_02_02_trnT(GUU)-comp-psbD-comp CATAGAGATGAAATTGGAACAAGTTGACCCCCTTTTCTATTTATTTTCATTTCTTTGGACTCCGCAAGAATTTGCCGATATTTCCAATTCAATCGTCTTGTCTTGTTCCTAGATGTTCTATAGGAAAAAATTGTCATTTCGTTCCTCTACGGAGAACCTTTTATTCTAAATTCTAAATCACAGGATCTAAATTCTAAATCACAGGATAAGAAAAAAATTCACTATCTTTCTTTGATTACAGGATCAAGATTCATTTANNNNTTATCTATACTATATAATATATTCNNNNNNTATAGATATTTCTATTCTATATTTATATTTATAGCTATCAGATCGTGGCTTGATGTACCAAAAATTTCCATTTCGTTGCATCCAATATTTTTGTTCCGACCATCGTATGAAGAAAGCATGCAAGATAAATACTATCATTTCCAATCTCCTATTTTAATTTTAATATTGTANNNNNNTTGAAGTGAAGTAAAAATTTGGAAACCCTCTCTTTTCTAACAGGGAAAAATAAATCAAAAAATATTAGTAATTTAGTCTACATAAAAATTAGAATATAAAGAGAGTTCTTTTTCTTAATCTCATGAACAAGATCTAAGAATCCATTTAGTTGATGAAAGAACATGGGGGGGGGCAGGCCTGAGGATCAACCGGTAGTGGGTNNNNGGAAGGGGATTGCTTTTTCCTTGACCAATTCTTTCAAAAAACGAATCGGATTCATGGGTCATAATAAGATAATTCATGATTCAGATGCTTAATAATAAGATAATAAGAAGGAATAATCAAATTGAATTCATGAATTTACCCGGCAATTTATGGTCCAATAAAGGATTTTTATCTTCGAAACCCATTGGAAGGGGTAGTGCACGAGAAAAAAAATCATGCGGAAATGATCGACTCCTTGGATGCCCCAAAATACTATGAGTGTCGGAA

023_20170917_02_03_trnT(GUU)-comp-psbD-comp CATAGAGATGAAATTGGAACAAGTTGACCCCCTTTTCTATTTATTTTCATTTCTTTGGACTCCGCAAGAATTTGCCGATATTTCCAATTCAATCGTCTTGTCTTGTTCCTAGATGTTCTATAGGAAAAAATTGTCATTTCGTTCCTCTACGGAGAACCTTTTATTCTAAATTCTAAATCACAGGATCTAAATTCTAAATCACAGGATAAGAAAAAAATTCACTATCTTTCTTTGATTACAGGATCAAGATTCATTTANNNNTTATCTATACTATATAATATATTCNNNNNNTATAGATATTTCTATTCTATATTTATATTTATAGCTATCAGATCGTGGCTTGATGTACCAAAAATTTCCATTTCGTTGCATCCAATATTTTTGTTCCGACCATCGTATGAAGAAAGCATGCAAGATAAATACTATCATTTCCAATCTCCTATTTTAATTTTAATATTGTANNNNNNTTGAAGTGAAGTAAAAATTTGGAAACCCTCTCTTTTCTAACAGGGAAAAATAAATCAAAAAATATTAGTAATTTAGTCTACATAAAAATTAGAATATAAAGAGAGTTCTTTTTCTTAATCTCATGAACAAGATCTAAGAATCCATTTAGTTGATGAAAGAACATGGGGGGGGGCAGGCCTGAGGATCAACCGGTAGTGGGTNNNNGGAAGGGGATTGCTTTTTCCTTGACCAATTCTTTCAAAAAACGAATCGGATTCATGGGTCATAATAAGATAATTCATGATTCAGATGCTTAATAATAAGATAATAAGAAGGAATAATCAAATTGAATTCATGAATTTACCCGGCAATTTATGGTCCAATAAAGGATTTTTATCTTCGAAACCCATTGGAAGGGGTAGTGCACGAGAAAAAAAATCATGCGGAAATGATCGACTCCTTGGATGCCCCAAAATACTATGAGTGTCGGAA

024_20170917_05_01_trnT(GUU)-comp-psbD-comp CATAGAGATGAAATTGGAACAAGTTGACCCCCTTTTCTATTTATTTTCATTTCTTTGGACTCCGCAAGAATTTGCCGATATTTCCAATTCAATCGTCTTGTCTTGTTCCTAGATGTTCTATAGGAAAAAATTGTCATTTCGTTCCTCTACGGAGAACCTTTTATTCTAAATTCTAAATCACAGGATCTAAATTCTAAATCACAGGATAAGAAAAAAATTCACTATCTTTCTTTGATTACAGGATCAAGATTCATTTANNNNTTATCTATACTATATAATATATTCNNNNNNTATAGATATTTCTATTCTATATTTATATTTATAGCTATCAGATCGTGGCTTGATGTACCAAAAATTTCCATTTCGTTGCATCCAATATTTTTGTTCCGACCATCGTATGAAGAAAGCATGCAAGATAAATACTATCATTTCCAATCTCCTATTTTAATTTTAATATTGTANNNNNNTTGAAGTGAAGTAAAAATTTGGAAACCCTCTCTTTTCTAACAGGGAAAAATAAATCAAAAAATATTAGTAATTTAGTCTACATAAAAATTAGAATATAAAGAGAGTTCTTTTTCTTAATCTCATGAACAAGATCTAAGAATCCATTTAGTTGATGAAAGAACATGGGGGGGGGCAGGCCTGAGGATCAACCGGTAGTGGGTNNNNGGAAGGGGATTGCTTTTTCCTTGACCAATTCTTTCAAAAAACGAATCGGATTCATGGGTCATAATAAGATAATTCATGATTCAGATGCTTAATAATAAGATAATAAGAAGGAATAATCAAATTGAATTCATGAATTTACCCGGCAATTTATGGTCCAATAAAGGATTTTTATCTTCGAAACCCATTGGAAGGGGTAGTGCACGAGAAAAAAAATCATGCGGAAATGATCGACTCCTTGGATGCCCCAAAATACTATGAGTGTCGGAA

025_20170917_05_02_trnT(GUU)-comp-psbD-comp CATAGAGATGAAATTGGAACAAGTTGACCCCCTTTTCTATTTATTTTCATTTCTTTGGACTCCGCAAGAATTTGCCGATATTTCCAATTCAATCGTCTTGTCTTGTTCCTAGATGTTCTATAGGAAAAAATTGTCATTTCGTTCCTCTACGGAGAACCTTTTATTCTAAATTCTAAATCACAGGATCTAAATTCTAAATCACAGGATAAGAAAAAAATTCACTATCTTTCTTTGATTACAGGATCAAGATTCATTTANNNNTTATCTATACTATATAATATATTCNNNNNNTATAGATATTTCTATTCTATATTTATATTTATAGCTATCAGATCGTGGCTTGATGTACCAAAAATTTCCATTTCGTTGCATCCAATATTTTTGTTCCGACCATCGTATGAAGAAAGCATGCAAGATAAATACTATCATTTCCAATCTCCTATTTTAATTTTAATATTGTANNNNNNTTGAAGTGAAGTAAAAATTTGGAAACCCTCTCTTTTCTAACAGGGAAAAATAAATCAAAAAATATTAGTAATTTAGTCTACATAAAAATTAGAATATAAAGAGAGTTCTTTTTCTTAATCTCATGAACAAGATCTAAGAATCCATTTAGTTGATGAAAGAACATGGGGGGGGGCAGGCCTGAGGATCAACCGGTAGTGGGTNNNNGGAAGGGGATTGCTTTTTCCTTGACCAATTCTTTCAAAAAACGAATCGGATTCATGGGTCATAATAAGATAATTCATGATTCAGATGCTTAATAATAAGATAATAAGAAGGAATAATCAAATTGAATTCATGAATTTACCCGGCAATTTATGGTCCAATAAAGGATTTTTATCTTCGAAACCCATTGGAAGGGGTAGTGCACGAGAAAAAAAATCATGCGGAAATGATCGACTCCTTGGATGCCCCAAAATACTATGAGTGTCGGAA

026_20170917_05_03_trnT(GUU)-comp-psbD-comp CATAGAGATGAAATTGGAACAAGTTGACCCCCTTTTCTATTTATTTTCATTTCTTTGGACTCCGCAAGAATTTGCCGATATTTCCAATTCAATCGTCTTGTCTTGTTCCTAGATGTTCTATAGGAAAAAATTGTCATTTCGTTCCTCTACGGAGAACCTTTTATTCTAAATTCTAAATCACAGGATCTAAATTCTAAATCACAGGATAAGAAAAAAATTCACTATCTTTCTTTGATTACAGGATCAAGATTCATTTANNNNTTATCTATACTATATAATATATTCNNNNNNTATAGATATTTCTATTCTATATTTATATTTATAGCTATCAGATCGTGGCTTGATGTACCAAAAATTTCCATTTCGTTGCATCCAATATTTTTGTTCCGACCATCGTATGAAGAAAGCATGCAAGATAAATACTATCATTTCCAATCTCCTATTTTAATTTTAATATTGTANNNNNNTTGAAGTGAAGTAAAAATTTGGAAACCCTCTCTTTTCTAACAGGGAAAAATAAATCAAAAAATATTAGTAATTTAGTCTACATAAAAATTAGAATATAAAGAGAGTTCTTTTTCTTAATCTCATGAACAAGATCTAAGAATCCATTTAGTTGATGAAAGAACATGGGGGGGGGCAGGCCTGAGGATCAACCGGTAGTGGGTNNNNGGAAGGGGATTGCTTTTTCCTTGACCAATTCTTTCAAAAAACGAATCGGATTCATGGGTCATAATAAGATAATTCATGATTCAGATGCTTAATAATAAGATAATAAGAAGGAATAATCAAATTGAATTCATGAATTTACCCGGCAATTTATGGTCCAATAAAGGATTTTTATCTTCGAAACCCATTGGAAGGGGTAGTGCACGAGAAAAAAAATCATGCGGAAATGATCGACTCCTTGGATGCCCCAAAATACTATGAGTGTCGGAA

027_20170917_05_04_trnT(GUU)-comp-psbD-comp CATAGAGATGAAATTGGAACAAGTTGACCCCCTTTTCTATTTATTTTCATTTCTTTGGACTCCGCAAGAATTTGCCGATATTTCCAATTCAATCGTCTTGTCTTGTTCCTAGATGTTCTATAGGAAAAAATTGTCATTTCGTTCCTCTACGGAGAACCTTTTATTCTAAATTCTAAATCACAGGATCTAAATTCTAAATCACAGGATAAGAAAAAAATTCACTATCTTTCTTTGATTACAGGATCAAGATTCATTTANNNNTTATCTATACTATATAATATATTCNNNNNNTATAGATATTTCTATTCTATATTTATATTTATAGCTATCAGATCGTGGCTTGATGTACCAAAAATTTCCATTTCGTTGCATCCAATATTTTTGTTCCGACCATCGTATGAAGAAAGCATGCAAGATAAATACTATCATTTCCAATCTCCTATTTTAATTTTAATATTGTANNNNNNTTGAAGTGAAGTAAAAATTTGGAAACCCTCTCTTTTCTAACAGGGAAAAATAAATCAAAAAATATTAGTAATTTAGTCTACATAAAAATTAGAATATAAAGAGAGTTCTTTTTCTTAATCTCATGAACAAGATCTAAGAATCCATTTAGTTGATGAAAGAACATGGGGGGGGGCAGGCCTGAGGATCAACCGGTAGTGGGTNNNNGGAAGGGGATTGCTTTTTCCTTGACCAATTCTTTCAAAAAACGAATCGGATTCATGGGTCATAATAAGATAATTCATGATTCAGATGCTTAATAATAAGATAATAAGAAGGAATAATCAAATTGAATTCATGAATTTACCCGGCAATTTATGGTCCAATAAAGGATTTTTATCTTCGAAACCCATTGGAAGGGGTAGTGCACGAGAAAAAAAATCATGCGGAAATGATCGACTCCTTGGATGCCCCAAAATACTATGAGTGTCGGAA

028_20170917_05_05_trnT(GUU)-comp-psbD-comp CATAGAGATGAAATTGGAACAAGTTGACCCCCTTTTCTATTTATTTTCATTTCTTTGGACTCCGCAAGAATTTGCCGATATTTCCAATTCAATCGTCTTGTCTTGTTCCTAGATGTTCTATAGGAAAAAATTGTCATTTCGTTCCTCTACGGAGAACCTTTTATTCTAAATTCTAAATCACAGGATCTAAATTCTAAATCACAGGATAAGAAAAAAATTCACTATCTTTCTTTGATTACAGGATCAAGATTCATTTANNNNTTATCTATACTATATAATATATTCNNNNNNTATAGATATTTCTATTCTATATTTATATTTATAGCTATCAGATCGTGGCTTGATGTACCAAAAATTTCCATTTCGTTGCATCCAATATTTTTGTTCCGACCATCGTATGAAGAAAGCATGCAAGATAAATACTATCATTTCCAATCTCCTATTTTAATTTTAATATTGTANNNNNNTTGAAGTGAAGTAAAAATTTGGAAACCCTCTCTTTTCTAACAGGGAAAAATAAATCAAAAAATATTAGTAATTTAGTCTACATAAAAATTAGAATATAAAGAGAGTTCTTTTTCTTAATCTCATGAACAAGATCTAAGAATCCATTTAGTTGATGAAAGAACATGGGGGGGGGCAGGCCTGAGGATCAACCGGTAGTGGGTNNNNGGAAGGGGATTGCTTTTTCCTTGACCAATTCTTTCAAAAAACGAATCGGATTCATGGGTCATAATAAGATAATTCATGATTCAGATGCTTAATAATAAGATAATAAGAAGGAATAATCAAATTGAATTCATGAATTTACCCGGCAATTTATGGTCCAATAAAGGATTTTTATCTTCGAAACCCATTGGAAGGGGTAGTGCACGAGAAAAAAAATCATGCGGAAATGATCGACTCCTTGGATGCCCCAAAATACTATGAGTGTCGGAA

029_20170917_08_02_trnT(GUU)-comp-psbD-comp CATAGAGATGAAATTGGAACAAGTTGACCCCCTTTTCTATTTATTTTCATTTCTTTGGACTCCGCAAGAATTTGCCGATATTTCCAATTCAATCGTCTTGTCTTGTTCCTAGATGTTCTATAGGAAAAAATTGTCATTTCGTTCCTCTACGGAGAACCTTTTATTCTAAATTCTAAATCACAGGATCTAAATTCTAAATCACAGGATAAGAAAAAAATTCACTATCTTTCTTTGATTACAGGATCAAGATTCATTTANNNNTTATCTATACTATATAATATATTCNNNNNNTATAGATATTTCTATTCTATATTTATATTTATAGCTATCAGATCGTGGCTTGATGTACCAAAAATTTCCATTTCGTTGCATCCAATATTTTTGTTCCGACCATCGTATGAAGAAAGCATGCAAGATAAATACTATCATTTCCAATCTCCTATTTTAATTTTAATATTGTANNNNNNTTGAAGTGAAGTAAAAATTTGGAAACCCTCTCTTTTCTAACAGGGAAAAATAAATCAAAAAATATTAGTAATTTAGTCTACATAAAAATTAGAATATAAAGAGAGTTCTTTTTCTTAATCTCATGAACAAGATCTAAGAATCCATTTAGTTGATGAAAGAACATGGGGGGGGGCAGGCCTGAGGATCAACCGGTAGTGGGTNNNNGGAAGGGGATTGCTTTTTCCTTGACCAATTCTTTCAAAAAACGAATCGGATTCATGGGTCATAATAAGATAATTCATGATTCAGATGCTTAATAATAAGATAATAAGAAGGAATAATCAAATTGAATTCATGAATTTACCCGGCAATTTATGGTCCAATAAAGGATTTTTATCTTCGAAACCCATTGGAAGGGGTAGTGCACGAGAAAAAAAATCATGCGGAAATGATCGACTCCTTGGATGCCCCAAAATACTATGAGTGTCGGAA

030_20170917_08_03_trnT(GUU)-comp-psbD-comp CATAGAGATGAAATTGGAACAAGTTGACCCCCTTTTCTATTTATTTTCATTTCTTTGGACTCCGCAAGAATTTGCCGATATTTCCAATTCAATCGTCTTGTCTTGTTCCTAGATGTTCTATAGGAAAAAATTGTCATTTCGTTCCTCTACGGAGAACCTTTTATTCTAAATTCTAAATCACAGGATCTAAATTCTAAATCACAGGATAAGAAAAAAATTCACTATCTTTCTTTGATTACAGGATCAAGATTCATTTANNNNTTATCTATACTATATAATATATTCNNNNNNTATAGATATTTCTATTCTATATTTATATTTATAGCTATCAGATCGTGGCTTGATGTACCAAAAATTTCCATTTCGTTGCATCCAATATTTTTGTTCCGACCATCGTATGAAGAAAGCATGCAAGATAAATACTATCATTTCCAATCTCCTATTTTAATTTTAATATTGTANNNNNNTTGAAGTGAAGTAAAAATTTGGAAACCCTCTCTTTTCTAACAGGGAAAAATAAATCAAAAAATATTAGTAATTTAGTCTACATAAAAATTAGAATATAAAGAGAGTTCTTTTTCTTAATCTCATGAACAAGATCTAAGAATCCATTTAGTTGATGAAAGAACATGGGGGGGGGCAGGCCTGAGGATCAACCGGTAGTGGGTNNNNGGAAGGGGATTGCTTTTTCCTTGACCAATTCTTTCAAAAAACGAATCGGATTCATGGGTCATAATAAGATAATTCATGATTCAGATGCTTAATAATAAGATAATAAGAAGGAATAATCAAATTGAATTCATGAATTTACCCGGCAATTTATGGTCCAATAAAGGATTTTTATCTTCGAAACCCATTGGAAGGGGTAGTGCACGAGAAAAAAAATCATGCGGAAATGATCGACTCCTTGGATGCCCCAAAATACTATGAGTGTCGGAA

031_20170917_08_04_trnT(GUU)-comp-psbD-comp CATAGAGATGAAATTGGAACAAGTTGACCCCCTTTTCTATTTATTTTCATTTCTTTGGACTCCGCAAGAATTTGCCGATATTTCCAATTCAATCGTCTTGTCTTGTTCCTAGATGTTCTATAGGAAAAAATTGTCATTTCGTTCCTCTACGGAGAACCTTTTATTCTAAATTCTAAATCACAGGATCTAAATTCTAAATCACAGGATAAGAAAAAAATTCACTATCTTTCTTTGATTACAGGATCAAGATTCATTTANNNNTTATCTATACTATATAATATATTCNNNNNNTATAGATATTTCTATTCTATATTTATATTTATAGCTATCAGATCGTGGCTTGATGTACCAAAAATTTCCATTTCGTTGCATCCAATATTTTTGTTCCGACCATCGTATGAAGAAAGCATGCAAGATAAATACTATCATTTCCAATCTCCTNNNNNNATTTTAATATTGTANNNNNNTTGAAGTGAAGTAAAAATTTGGAAACCCTCTCTTTTCTAACAGGGAAAAATAAATCAAAAAATATTAGTAATTTAGTCTACATAAAAATTAGAATATAAAGAGAGTTCTTTTTCTTAATCTCATGAACAAGATCTAAGAATCCATTTAGTTGATGAAAGAACATGGGGGGGGGCAGGCCTGAGGATCAACCGGTAGTGGGTNNNNGGAAGGGGATTGCTTTTTCCTTGACCAATTCTTTCAAAAAACGAATCGGATTCATGGGTCATAATAAGATAATTCATGATTCAGATGCTTAATAATAAGATAATAAGAAGGAATAATCAAATTGAATTCATGAATTTACCCGGCAATTTATGGTCCAATAAAGGATTTTTATCTTCGAAACCCATTGGAAGGGGTAGTGCACGAGAAAAAAAATCATGCGGAAATGATCGACTCCTTGGATGCCCCAAAATACTATGAGTGTCGGAA

032_20170917_08_05_trnT(GUU)-comp-psbD-comp CATAGAGATGAAATTGGAACAAGTTGACCCCCTTTTCTATTTATTTTCATTTCTTTGGACTCCGCAAGAATTTGCCGATATTTCCAATTCAATCGTCTTGTCTTGTTCCTAGATGTTCTATAGGAAAAAATTGTCATTTCGTTCCTCTACGGAGAACCTTTTATTCTAAATTCTAAATCACAGGATCTAAATTCTAAATCACAGGATAAGAAAAAAATTCACTATCTTTCTTTGATTACAGGATCAAGATTCATTTANNNNTTATCTATACTATATAATATATTCNNNNNNTATAGATATTTCTATTCTATATTTATATTTATAGCTATCAGATCGTGGCTTGATGTACCAAAAATTTCCATTTCGTTGCATCCAATATTTTTGTTCCGACCATCGTATGAAGAAAGCATGCAAGATAAATACTATCATTTCCAATCTCCTATTTTAATTTTAATATTGTANNNNNNTTGAAGTGAAGTAAAAATTTGGAAACCCTCTCTTTTCTAACAGGGAAAAATAAATCAAAAAATATTAGTAATTTAGTCTACATAAAAATTAGAATATAAAGAGAGTTCTTTTTCTTAATCTCATGAACAAGATCTAAGAATCCATTTAGTTGATGAAAGAACATGGGGGGGGGCAGGCCTGAGGATCAACCGGTAGTGGGTNNNNGGAAGGGGATTGCTTTTTCCTTGACCAATTCTTTCAAAAAACGAATCGGATTCATGGGTCATAATAAGATAATTCATGATTCAGATGCTTAATAATAAGATAATAAGAAGGAATAATCAAATTGAATTCATGAATTTACCCGGCAATTTATGGTCCAATAAAGGATTTTTATCTTCGAAACCCATTGGAAGGGGTAGTGCACGAGAAAAAAAATCATGCGGAAATGATCGACTCCTTGGATGCCCCAAAATACTATGAGTGTCGGAA

033_20170917_08_06_trnT(GUU)-comp-psbD-comp CATAGAGATGAAATTGGAACAAGTTGACCCCCTTTTCTATTTATTTTCATTTCTTTGGACTCCGCAAGAATTTGCCGATATTTCCAATTCAATCGTCTTGTCTTGTTCCTAGATGTTCTATAGGAAAAAATTGTCATTTCGTTCCTCTACGGAGAACCTTTTATTCTAAATTCTAAATCACAGGATCTAAATTCTAAATCACAGGATAAGAAAAAAATTCACTATCTTTCTTTGATTACAGGATCAAGATTCATTTANNNNTTATCTATACTATATAATATATTCNNNNNNTATAGATATTTCTATTCTATATTTATATTTATAGCTATCAGATCGTGGCTTGATGTACCAAAAATTTCCATTTCGTTGCATCCAATATTTTTGTTCCGACCATCGTATGAAGAAAGCATGCAAGATAAATACTATCATTTCCAATCTCCTATTTTAATTTTAATATTGTANNNNNNTTGAAGTGAAGTAAAAATTTGGAAACCCTCTCTTTTCTAACAGGGAAAAATAAATCAAAAAATATTAGTAATTTAGTCTACATAAAAATTAGAATATAAAGAGAGTTCTTTTTCTTAATCTCATGAACAAGATCTAAGAATCCATTTAGTTGATGAAAGAACATGGGGGGGGGCAGGCCTGAGGATCAACCGGTAGTGGGTNNNNGGAAGGGGATTGCTTTTTCCTTGACCAATTCTTTCAAAAAACGAATCGGATTCATGGGTCATAATAAGATAATTCATGATTCAGATGCTTAATAATAAGATAATAAGAAGGAATAATCAAATTGAATTCATGAATTTACCCGGCAATTTATGGTCCAATAAAGGATTTTTATCTTCGAAACCCATTGGAAGGGGTAGTGCACGAGAAAAAAAATCATGCGGAAATGATCGACTCCTTGGATGCCCCAAAATACTATGAGTGTCGGAA

034_20170918_01_01_trnT(GUU)-comp-psbD-comp CATAGAGATGAAATTGGAACAAGTTGACCCCCTTTTCTATTTATTTTCATTTCTTTGGACTCCGCAAGAATTTGCCGATATTTCCAATTCAATCGTCTTGTCTTGTTCCTAGATGTTCTATAGGAAAAAATTGTCATTTCGTTCCTCTACGGAGAACCTTTTATTCTAAATTCTAAATCACAGGATCTAAATTCTAAATCACAGGATAAGAAAAAAATTCACTATCTTTCTTTGATTACAGGATCAAGATTCATTTANNNNTTATCTATACTATATAATATATTCNNNNNNTATAGATATTTCTATTCTATATTTATATTTATAGCTATCAGATCGTGGCTTGATGTACCAAAAATTTCCATTTCGTTGCATCCAATATTTTTGTTCCGACCATCGTATGAAGAAAGCATGCAAGATAAATACTATCATTTCCAATCTCCTATTTTAATTTTAATATTGTANNNNNNTTGAAGTGAAGTAAAAATTTGGAAACCCTCTCTTTTCTAACAGGGAAAAATAAATCAAAAAATATTAGTAATTTAGTCTACATAAAAATTAGAATATAAAGAGAGTTCTTTTTCTTAATCTCATGAACAAGATCTAAGAATCCATTTAGTTGATGAAAGAACATGGGGGGGGGCAGGCCTGAGGATCAACCGGTAGTGGGTNNNNGGAAGGGGATTGCTTTTTCCTTGACCAATTCTTTCAAAAAACGAATCGGATTCATGGGTCATAATAAGATAATTCATGATTCAGATGCTTAATAATAAGATAATAAGAAGGAATAATCAAATTGAATTCATGAATTTACCCGGCAATTTATGGTCCAATAAAGGATTTTTATCTTCGAAACCCATTGGAAGGGGTAGTGCACGAGAAAAAAAATCATGCGGAAATGATCGACTCCTTGGATGCCCCAAAATACTATGAGTGTCGGAA

035_20170918_01_02_trnT(GUU)-comp-psbD-comp CATAGAGATGAAATTGGAACAAGTTGACCCCCTTTTCTATTTATTTTCATTTCTTTGGACTCCGCAAGAATTTGCCGATATTTCCAATTCAATCGTCTTGTCTTGTTCCTAGATGTTCTATAGGAAAAAATTGTCATTTCGTTCCTCTACGGAGAACCTTTTATTCTAAATTCTAAATCACAGGATCTAAATTCTAAATCACAGGATAAGAAAAAAATTCACTATCTTTCTTTGATTACAGGATCAAGATTCATTTANNNNTTATCTATACTATATAATATATTCNNNNNNTATAGATATTTCTATTCTATATTTATATTTATAGCTATCAGATCGTGGCTTGATGTACCAAAAATTTCCATTTCGTTGCATCCAATATTTTTGTTCCGACCATCGTATGAAGAAAGCATGCAAGATAAATACTATCATTTCCAATCTCCTATTTTAATTTTAATATTGTANNNNNNTTGAAGTGAAGTAAAAATTTGGAAACCCTCTCTTTTCTAACAGGGAAAAATAAATCAAAAAATATTAGTAATTTAGTCTACATAAAAATTAGAATATAAAGAGAGTTCTTTTTCTTAATCTCATGAACAAGATCTAAGAATCCATTTAGTTGATGAAAGAACATGGGGGGGGGCAGGCCTGAGGATCAACCGGTAGTGGGTNNNNGGAAGGGGATTGCTTTTTCCTTGACCAATTCTTTCAAAAAACGAATCGGATTCATGGGTCATAATAAGATAATTCATGATTCAGATGCTTAATAATAAGATAATAAGAAGGAATAATCAAATTGAATTCATGAATTTACCCGGCAATTTATGGTCCAATAAAGGATTTTTATCTTCGAAACCCATTGGAAGGGGTAGTGCACGAGAAAAAAAATCATGCGGAAATGATCGACTCCTTGGATGCCCCAAAATACTATGAGTGTCGGAA

036_20170918_01_03_trnT(GUU)-comp-psbD-comp CATAGAGATGAAATTGGAACAAGTTGACCCCCTTTTCTATTTATTTTCATTTCTTTGGACTCCGCAAGAATTTGCCGATATTTCCAATTCAATCGTCTTGTCTTGTTCCTAGATGTTCTATAGGAAAAAATTGTCATTTCGTTCCTCTACGGAGAACCTTTTATTCTAAATTCTAAATCACAGGATCTAAATTCTAAATCACAGGATAAGAAAAAAATTCACTATCTTTCTTTGATTACAGGATCAAGATTCATTTANNNNTTATCTATACTATATAATATATTCNNNNNNTATAGATATTTCTATTCTATATTTATATTTATAGCTATCAGATCGTGGCTTGATGTACCAAAAATTTCCATTTCGTTGCATCCAATATTTTTGTTCCGACCATCGTATGAAGAAAGCATGCAAGATAAATACTATCATTTCCAATCTCCTATTTTAATTTTAATATTGTANNNNNNTTGAAGTGAAGTAAAAATTTGGAAACCCTCTCTTTTCTAACAGGGAAAAATAAATCAAAAAATATTAGTAATTTAGTCTACATAAAAATTAGAATATAAAGAGAGTTCTTTTTCTTAATCTCATGAACAAGATCTAAGAATCCATTTAGTTGATGAAAGAACATGGGGGGGGGCAGGCCTGAGGATCAACCGGTAGTGGGTNNNNGGAAGGGGATTGCTTTTTCCTTGACCAATTCTTTCAAAAAACGAATCGGATTCATGGGTCATAATAAGATAATTCATGATTCAGATGCTTAATAATAAGATAATAAGAAGGAATAATCAAATTGAATTCATGAATTTACCCGGCAATTTATGGTCCAATAAAGGATTTTTATCTTCGAAACCCATTGGAAGGGGTAGTGCACGAGAAAAAAAATCATGCGGAAATGATCGACTCCTTGGATGCCCCAAAATACTATGAGTGTCGGAA

037_20170918_01_04_trnT(GUU)-comp-psbD-comp CATAGAGATGAAATTGGAACAAGTTGACCCCCTTTTCTATTTATTTTCATTTCTTTGGACTCCGCAAGAATTTGCCGATATTTCCAATTCAATCGTCTTGTCTTGTTCCTAGATGTTCTATAGGAAAAAATTGTCATTTCGTTCCTCTACGGAGAACCTTTTATTCTAAATTCTAAATCACAGGATCTAAATTCTAAATCACAGGATAAGAAAAAAATTCACTATCTTTCTTTGATTACAGGATCAAGATTCATTTANNNNTTATCTATACTATATAATATATTCNNNNNNTATAGATATTTCTATTCTATATTTATATTTATAGCTATCAGATCGTGGCTTGATGTACCAAAAATTTCCATTTCGTTGCATCCAATATTTTTGTTCCGACCATCGTATGAAGAAAGCATGCAAGATAAATACTATCATTTCCAATCTCCTATTTTAATTTTAATATTGTANNNNNNTTGAAGTGAAGTAAAAATTTGGAAACCCTCTCTTTTCTAACAGGGAAAAATAAATCAAAAAATATTAGTAATTTAGTCTACATAAAAATTAGAATATAAAGAGAGTTCTTTTTCTTAATCTCATGAACAAGATCTAAGAATCCATTTAGTTGATGAAAGAACATGGGGGGGGGCAGGCCTGAGGATCAACCGGTAGTGGGTNNNNGGAAGGGGATTGCTTTTTCCTTGACCAATTCTTTCAAAAAACGAATCGGATTCATGGGTCATAATAAGATAATTCATGATTCAGATGCTTAATAATAAGATAATAAGAAGGAATAATCAAATTGAATTCATGAATTTACCCGGCAATTTATGGTCCAATAAAGGATTTTTATCTTCGAAACCCATTGGAAGGGGTAGTGCACGAGAAAAAAAATCATGCGGAAATGATCGACTCCTTGGATGCCCCAAAATACTATGAGTGTCGGAA

038_20170918_01_05_trnT(GUU)-comp-psbD-comp CATAGAGATGAAATTGGAACAAGTTGACCCCCTTTTCTATTTATTTTCATTTCTTTGGACTCCGCAAGAATTTGCCGATATTTCCAATTCAATCGTCTTGTCTTGTTCCTAGATGTTCTATAGGAAAAAATTGTCATTTCGTTCCTCTACGGAGAACCTTTTATTCTAAATTCTAAATCACAGGATCTAAATTCTAAATCACAGGATAAGAAAAAAATTCACTATCTTTCTTTGATTACAGGATCAAGATTCATTTANNNNTTATCTATACTATATAATATATTCNNNNNNTATAGATATTTCTATTCTATATTTATATTTATAGCTATCAGATCGTGGCTTGATGTACCAAAAATTTCCATTTCGTTGCATCCAATATTTTTGTTCCGACCATCGTATGAAGAAAGCATGCAAGATAAATACTATCATTTCCAATCTCCTATTTTAATTTTAATATTGTANNNNNNTTGAAGTGAAGTAAAAATTTGGAAACCCTCTCTTTTCTAACAGGGAAAAATAAATCAAAAAATATTAGTAATTTAGTCTACATAAAAATTAGAATATAAAGAGAGTTCTTTTTCTTAATCTCATGAACAAGATCTAAGAATCCATTTAGTTGATGAAAGAACATGGGGGGGGGCAGGCCTGAGGATCAACCGGTAGTGGGTNNNNGGAAGGGGATTGCTTTTTCCTTGACCAATTCTTTCAAAAAACGAATCGGATTCATGGGTCATAATAAGATAATTCATGATTCAGATGCTTAATAATAAGATAATAAGAAGGAATAATCAAATTGAATTCATGAATTTACCCGGCAATTTATGGTCCAATAAAGGATTTTTATCTTCGAAACCCATTGGAAGGGGTAGTGCACGAGAAAAAAAATCATGCGGAAATGATCGACTCCTTGGATGCCCCAAAATACTATGAGTGTCGGAA

039_20170918_02_02_trnT(GUU)-comp-psbD-comp CATAGAGATGAAATTGGAACAAGTTGACCCCCTTTTCTATTTATTTTCATTTCTTTGGACTCCGCAAGAATTTGCCGATATTTCCAATTCAATCGTCTTGTCTTGTTCCTAGATGTTCTATAGGAAAAAATTGTCATTTCGTTCCTCTACGGAGAACCTTTTATTCTAAATTCTAAATCACAGGATCTAAATTCTAAATCACAGGATAAGAAAAAAATTCACTATCTTTCTTTGATTACAGGATCAAGATTCATTTANNNNTTATCTATACTATATAATATATTCNNNNNNTATAGATATTTCTATTCTATATTTATATTTATAGCTATCAGATCGTGGCTTGATGTACCAAAAATTTCCATTTCGTTGCATCCAATATTTTTGTTCCGACCATCGTATGAAGAAAGCATGCAAGATAAATACTATCATTTCCAATCTCCTATTTTAATTTTAATATTGTANNNNNNTTGAAGTGAAGTAAAAATTTGGAAACCCTCTCTTTTCTAACAGGGAAAAATAAATCAAAAAATATTAGTAATTTAGTCTACATAAAAATTAGAATATAAAGAGAGTTCTTTTTCTTAATCTCATGAACAAGATCTAAGAATCCATTTAGTTGATGAAAGAACATGGGGGGGGGCAGGCCTGAGGATCAACCGGTAGTGGGTNNNNGGAAGGGGATTGCTTTTTCCTTGACCAATTCTTTCAAAAAACGAATCGGATTCATGGGTCATAATAAGATAATTCATGATTCAGATGCTTAATAATAAGATAATAAGAAGGAATAATCAAATTGAATTCATGAATTTACCCGGCAATTTATGGTCCAATAAAGGATTTTTATCTTCGAAACCCATTGGAAGGGGTAGTGCACGAGAAAAAAAATCATGCGGAAATGATCGACTCCTTGGATGCCCCAAAATACTATGAGTGTCGGAA

040_20170918_02_03_trnT(GUU)-comp-psbD-comp CATAGAGATGAAATTGGAACAAGTTGACCCCCTTTTCTATTTATTTTCATTTCTTTGGACTCCGCAAGAATTTGCCGATATTTCCAATTCAATCGTCTTGTCTTGTTCCTAGATGTTCTATAGGAAAAAATTGTCATTTCGTTCCTCTACGGAGAACCTTTTATTCTAAATTCTAAATCACAGGATCTAAATTCTAAATCACAGGATAAGAAAAAAATTCACTATCTTTCTTTGATTACAGGATCAAGATTCATTTANNNNTTATCTATACTATATAATATATTCNNNNNNTATAGATATTTCTATTCTATATTTATATTTATAGCTATCAGATCGTGGCTTGATGTACCAAAAATTTCCATTTCGTTGCATCCAATATTTTTGTTCCGACCATCGTATGAAGAAAGCATGCAAGATAAATACTATCATTTCCAATCTCCTATTTTAATTTTAATATTGTANNNNNNTTGAAGTGAAGTAAAAATTTGGAAACCCTCTCTTTTCTAACAGGGAAAAATAAATCAAAAAATATTAGTAATTTAGTCTACATAAAAATTAGAATATAAAGAGAGTTCTTTTTCTTAATCTCATGAACAAGATCTAAGAATCCATTTAGTTGATGAAAGAACATGGGGGGGGGCAGGCCTGAGGATCAACCGGTAGTGGGTNNNNGGAAGGGGATTGCTTTTTCCTTGACCAATTCTTTCAAAAAACGAATCGGATTCATGGGTCATAATAAGATAATTCATGATTCAGATGCTTAATAATAAGATAATAAGAAGGAATAATCAAATTGAATTCATGAATTTACCCGGCAATTTATGGTCCAATAAAGGATTTTTATCTTCGAAACCCATTGGAAGGGGTAGTGCACGAGAAAAAAAATCATGCGGAAATGATCGACTCCTTGGATGCCCCAAAATACTATGAGTGTCGGAA

041_20170918_02_04_trnT(GUU)-comp-psbD-comp CATAGAGATGAAATTGGAACAAGTTGACCCCCTTTTCTATTTATTTTCATTTCTTTGGACTCCGCAAGAATTTGCCGATATTTCCAATTCAATCGTCTTGTCTTGTTCCTAGATGTTCTATAGGAAAAAATTGTCATTTCGTTCCTCTACGGAGAACCTTTTATTCTAAATTCTAAATCACAGGATCTAAATTCTAAATCACAGGATAAGAAAAAAATTCACTATCTTTCTTTGATTACAGGATCAAGATTCATTTANNNNTTATCTATACTATATAATATATTCNNNNNNTATAGATATTTCTATTCTATATTTATATTTATAGCTATCAGATCGTGGCTTGATGTACCAAAAATTTCCATTTCGTTGCATCCAATATTTTTGTTCCGACCATCGTATGAAGAAAGCATGCAAGATAAATACTATCATTTCCAATCTCCTATTTTAATTTTAATATTGTANNNNNNTTGAAGTGAAGTAAAAATTTGGAAACCCTCTCTTTTCTAACAGGGAAAAATAAATCAAAAAATATTAGTAATTTAGTCTACATAAAAATTAGAATATAAAGAGAGTTCTTTTTCTTAATCTCATGAACAAGATCTAAGAATCCATTTAGTTGATGAAAGAACATGGGGGGGGGCAGGCCTGAGGATCAACCGGTAGTGGGTNNNNGGAAGGGGATTGCTTTTTCCTTGACCAATTCTTTCAAAAAACGAATCGGATTCATGGGTCATAATAAGATAATTCATGATTCAGATGCTTAATAATAAGATAATAAGAAGGAATAATCAAATTGAATTCATGAATTTACCCGGCAATTTATGGTCCAATAAAGGATTTTTATCTTCGAAACCCATTGGGAGGGGTAGTGCACGAGAAAAAAAATCATGCGGAAATGATCGACTCCTTGGATGCCCCAAAATACTATGAGTGTCGGAA

042_20170918_02_05_trnT(GUU)-comp-psbD-comp CATAGAGATGAAATTGGAACAAGTTGACCCCCTTTTCTATTTATTTTCATTTCTTTGGACTCCGCAAGAATTTGCCGATATTTCCAATTCAATCGTCTTGTCTTGTTCCTAGATGTTCTATAGGAAAAAATTGTCATTTCGTTCCTCTACGGAGAACCTTTTATTCTAAATTCTAAATCACAGGATCTAAATTCTAAATCACAGGATAAGAAAAAAATTCACTATCTTTCTTTGATTACAGGATCAAGATTCATTTANNNNTTATCTATACTATATAATATATTCNNNNNNTATAGATATTTCTATTCTATATTTATATTTATAGCTATCAGATCGTGGCTTGATGTACCAAAAATTTCCATTTCGTTGCATCCAATATTTTTGTTCCGACCATCGTATGAAGAAAGCATGCAAGATAAATACTATCATTTCCAATCTCCTATTTTAATTTTAATATTGTANNNNNNTTGAAGTGAAGTAAAAATTTGGAAACCCTCTCTTTTCTAACAGGGAAAAATAAATCAAAAAATATTAGTAATTTAGTCTACATAAAAATTAGAATATAAAGAGAGTTCTTTTTCTTAATCTCATGAACAAGATCTAAGAATCCATTTAGTTGATGAAAGAACATGGGGGGGGGCAGGCCTGAGGATCAACCGGTAGTGGGTNNNNGGAAGGGGATTGCTTTTTCCTTGACCAATTCTTTCAAAAAACGAATCGGATTCATGGGTCATAATAAGATAATTCATGATTCAGATGCTTAATAATAAGATAATAAGAAGGAATAATCAAATTGAATTCATGAATTTACCCGGCAATTTATGGTCCAATAGAGGATTTTTATCTTCGAAACCCATTGGAAGGGGTAGTGCACGAGAAAAAAAATCATGCGGAAATGATCGACTCCATGGATGCCCCGAAATACTATGAGTGTCGGAA

043_20170918_02_06_trnT(GUU)-comp-psbD-comp CATAGAGATGAAATTGGAACAAGTTGACCCCCTTTTCTATTTATTTTCATTTCTTTGGACTCCGCAAGAATTTGCCGATATTTCCAATTCAATCGTCTTGTCTTGTTCCTAGATGTTCTATAGGAAAAAATTGTCATTTCGTTCCTCTACGGAGAACCTTTTATTCTAAATTCTAAATCACAGGATCTAAATTCTAAATCACAGGATAAGAAAAAAATTCACTATCTTTCTTTGATTACAGGATCAAGATTCATTTANNNNTTATCTATACTATATAATATATTCNNNNNNTATAGATATTTCTATTCTATATTTATATTTATAGCTATCAGATCGTGGCTTGATGTACCAAAAATTTCCATTTCGTTGCATCCAATATTTTTGTTCCGACCATCGTATGAAGAAAGCATGCAAGATAAATACTATCATTTCCAATCTCCTATTTTAATTTTAATATTGTANNNNNNTTGAAGTGAAGTAAAAATTTGGAAACCCTCTCTTTTCTAACAGGGAAAAATAAATCAAAAAATATTAGTAATTTAGTCTACATAAAAATTAGAATATAAAGAGAGTTCTTTTTCTTAATCTCATGAACAAGATCTAAGAATCCATTTAGTTGATGAAAGAACATGGGGGGGGGCAGGCCTGAGGATCAACCGGTAGTGGGTNNNNGGAAGGGGATTGCTTTTTCCTTGACCAATTCTTTCAAAAAACGAATCGGATTCATGGGTCATAATAAGATAATTCATGATTCAGATGCTTAATAATAAGATAATAAGAAGGAATAATCAAATTGAATTCATGAATTTACCCGGCAATTTATGGTCCAATAAAGGATTTTTATCTTCGAAACCCATTGGAAGGGGTAGTGCACGAGAAAAAAAATCATGCGGAAATGATCGACTCCTTGGATGCCCCAAAATACTATGAGTGTCGGAA

044_20170920_01_01_trnT(GUU)-comp-psbD-comp CATAGAGATGAAATTGGAACAAGTTGACCCCCTTTTCTATTTATTTTCATTTCTTTGGACTCCGCAAGAATTTGCCGATATTTCCAATTCAATCGTCTTGTCTTGTTCCTAGATGTTCTATAGGAAAAAATTGTCATTTCGTTCCTCTACGGAGAACCTTTTATTCTAAATTCTAAATCACAGGATCTAAATTCTAAATCACAGGATAAGAAAAAAATTCACTATCTTTCTTTGATTACAGGATCAAGATTCATTTANNNNTTATCTATACTATATAATATATTCNNNNNNTATAGATATTTCTATTCTATATTTATATTTATAGCTATCAGATCGTGGCTTGATGTACCAAAAATTTCCATTTCGTTGCATCCAATATTTTTGTTCCGACCATCGTATGAAGAAAGCATGCAAGATAAATACTATCATTTCCAATCTCCTATTTTAATTTTAATATTGTANNNNNNTTGAAGTGAAGTAAAAATTTGGAAACCCTCTCTTTTCTAACAGGGAAAAATAAATCAAAAAATATTAGTAATTTAGTCTACATAAAAATTAGAATATAAAGAGAGTTCTTTTTCTTAATCTCATGAACAAGATCTAAGAATCCATTTAGTTGATGAAAGAACATGGGGGGGGGCAGGCCTGAGGATCAACCGGTAGTGGGTNNNNGGAAGGGGATTGCTTTTTCCTTGACCAATTCTTTCAAAAAACGAATCGGATTCATGGGTCATAATAAGATAATTCATGATTCAGATGCTTAATAATAAGATAATAAGAAGGAATAATCAAATTGAATTCATGAATTTACCCGGCAATTTATGGTCCAATAAAGGATTTTTATCTTCGAAACCCATTGGAAGGGGTAGTGCACGAGAAAAAAAATCATGCGGAAATGATCGACTCCTTGGATGCCCCAAAATACTATGAGTGTCGGAA

045_20170920_01_02_trnT(GUU)-comp-psbD-comp CATAGAGATGAAATTGGAACAAGTTGACCCCCTTTTCTATTTATTTTCATTTCTTTGGACTCCGCAAGAATTTGCCGATATTTCCAATTCAATCGTCTTGTCTTGTTCCTAGATGTTCTATAGGAAAAAATTGTCATTTCGTTCCTCTACGGAGAACCTTTTATTCTAAATTCTAAATCACAGGATCTAAATTCTAAATCACAGGATAAGAAAAAAATTCACTATCTTTCTTTGATTACAGGATCAAGATTCATTTANNNNTTATCTATACTATATAATATATTCNNNNNNTATAGATATTTCTATTCTATATTTATATTTATAGCTATCAGATCGTGGCTTGATGTACCAAAAATTTCCATTTCGTTGCATCCAATATTTTTGTTCCGACCATCGTATGAAGAAAGCATGCAAGATAAATACTATCATTTCCAATCTCCTATTTTAATTTTAATATTGTANNNNNNTTGAAGTGAAGTAAAAATTTGGAAACCCTCTCTTTTCTAACAGGGAAAAATAAATCAAAAAATATTAGTAATTTAGTCTACATAAAAATTAGAATATAAAGAGAGTTCTTTTTCTTAATCTCATGAACAAGATCTAAGAATCCATTTAGTTGATGAAAGAACATGGGGGGGGGCAGGCCTGAGGATCAACCGGTAGTGGGTNNNNGGAAGGGGATTGCTTTTTCCTTGACCAATTCTTTCAAAAAACGAATCGGATTCATGGGTCATAATAAGATAATTCATGATTCAGATGCTTAATAATAAGATAATAAGAAGGAATAATCAAATTGAATTCATGAATTTACCCGGCAATTTATGGTCCAATAAAGGATTTTTATCTTCGAAACCCATTGGAAGGGGTAGTGCACGAGAAAAAAAATCATGCGGAAATGATCGACTCCTTGGATGCCCCAAAATACTATGAGTGTCGGAA

046_20170920_01_03_trnT(GUU)-comp-psbD-comp CATAGAGATGAAATTGGAACAAGTTGACCCCCTTTTCTATTTATTTTCATTTCTTTGGACTCCGCAAGAATTTGCCGATATTTCCAATTCAATCGTCTTGTCTTGTTCCTAGATGTTCTATAGGAAAAAATTGTCATTTCGTTCCTCTACGGAGAACCTTTTATTCTAAATTCTAAATCACAGGATCTAAATTCTAAATCACAGGATAAGAAAAAAATTCACTATCTTTCTTTGATTACAGGATCAAGATTCATTTANNNNTTATCTATACTATATAATATATTCNNNNNNTATAGATATTTCTATTCTATATTTATATTTATAGCTATCAGATCGTGGCTTGATGTACCAAAAATTTCCATTTCGTTGCATCCAATATTTTTGTTCCGACCATCGTATGAAGAAAGCATGCAAGATAAATACTATCATTTCCAATCTCCTATTTTAATTTTAATATTGTANNNNNNTTGAAGTGAAGTAAAAATTTGGAAACCCTCTCTTTTCTAACAGGGAAAAATAAATCAAAAAATATTAGTAATTTAGTCTACATAAAAATTAGAATATAAAGAGAGTTCTTTTTCTTAATCTCATGAACAAGATCTAAGAATCCATTTAGTTGATGAAAGAACATGGGGGGGGGCAGGCCTGAGGATCAACCGGTAGTGGGTNNNNGGAAGGGGATTGCTTTTTCCTTGACCAATTCTTTCAAAAAACGAATCGGATTCATGGGTCATAATAAGATAATTCATGATTCAGATGCTTAATAATAAGATAATAAGAAGGAATAATCAAATTGAATTCATGAATTTACCCGGCAATTTATGGTCCAATAAAGGATTTTTATCTTCGAAACCCATTGGAAGGGGTAGTGCACGAGAAAAAAAATCATGCGGAAATGATCGACTCCTTGGATGCCCCAAAATACTATGAGTGTCGGAA

047_20180610_01_01_trnT(GUU)-comp-psbD-comp CATAGAGATGAAATTGGAACAAGTTGACCCCCTTTTCTATTTATTTTCATTTCTTTGGACTCCGCAAGAATTTGCCGATATTTCCAATTCAATCGTCTTGTCTTGTTCCTAGATGTTCTATAGGAAAAAATTGTCATTTCGTTCCTCTACGGAGAACCTTTTATTCTAAATTCTAAATCACAGGATCTAAATTCTAAATCACAGGATAAGAAAAAAATTCACTATCTTTCTTTGATTACAGGATCAAGATTCATTTANNNNTTATCTATACTATATAATATATTCNNNNNNTATAGATATTTCTATTCTATATTTATATTTATAGCTATCAGATCGTGGCTTGATGTACCAAAAATTTCCATTTCGTTGCATCCAATATTTTTGTTCCGACCATCGTATGAAGAAAGCATGCAAGATAAATACTATCATTTCCAATCTCCTATTTTAATTTTAATATTGTANNNNNNTTGAAGTGAAGTAAAAATTTGGAAACCCTCTCTTTTCTAACAGGGAAAAATAAATCAAAAAATATTAGTAATTTAGTCTACATAAAAATTAGAATATAAAGAGAGTTCTTTTTCTTAATCTCATGAACAAGATCTAAGAATCCATTTAGTTGATGAAAGAACATGGGGGGGGGCAGGCCTGAGGATCAACCGGTAGTGGGTNNNNGGAAGGGGATTGCTTTTTCCTTGACCAATTCTTTCAAAAAACGAATCGGATTCATGGGTCATAATAAGATAATTCATGATTCAGATGCTTAATAATAAGATAATAAGAAGGAATAATCAAATTGAATTCATGAATTTACCCGGCAATTTATGGTCCAATAAAGGATTTTTATCTTCGAAACCCATTGGAAGGGGTAGTGCACGAGAAAAAAAATCATGCGGAAATGATCGACTCCTTGGATGCCCCAAAATACTATGAGTGTCGGAA

048_20180610_01_02_trnT(GUU)-comp-psbD-comp CATAGAGATGAAATTGGAACAAGTTGACCCCCTTTTCTATTTATTTTCATTTCTTTGGACTCCGCAAGAATTTGCCGATATTTCCAATTCAATCGTCTTGTCTTGTTCCTAGATGTTCTATAGGAAAAAATTGTCATTTCGTTCCTCTACGGAGAACCTTTTATTCTAAATTCTAAATCACAGGATCTAAATTCTAAATCACAGGATAAGAAAAAAATTCACTATCTTTCTTTGATTACAGGATCAAGATTCATTTANNNNTTATCTATACTATATAATATATTCNNNNNNTATAGATATTTCTATTCTATATTTATATTTATAGCTATCAGATCGTGGCTTGATGTACCAAAAATTTCCATTTCGTTGCATCCAATATTTTTGTTCCGACCATCGTATGAAGAAAGCATGCAAGATAAATACTATCATTTCCAATCTCCTATTTTAATTTTAATATTGTANNNNNNTTGAAGTGAAGTAAAAATTTGGAAACCCTCTCTTTTCTAACAGGGAAAAATAAATCAAAAAATATTAGTAATTTAGTCTACATAAAAATTAGAATATAAAGAGAGTTCTTTTTCTTAATCTCATGAACAAGATCTAAGAATCCATTTAGTTGATGAAAGAACATGGGGGGGGGCAGGCCTGAGGATCAACCGGTAGTGGGTNNNNGGAAGGGGATTGCTTTTTCCTTGACCAATTCTTTCAAAAAACGAATCGGATTCATGGGTCATAATAAGATAATTCATGATTCAGATGCTTAATAATAAGATAATAAGAAGGAATAATCAAATTGAATTCATGAATTTACCCGGCAATTTATGGTCCAATAAAGGATTTTTATCTTCGAAACCCATTGGAAGGGGTAGTGCACGAGAAAAAAAATCATGCGGAAATGATCGACTCCTTGGATGCCCCAAAATACTATGAGTGTCGGAA

049_20180610_01_03_trnT(GUU)-comp-psbD-comp CATAGAGATGAAATTGGAACAAGTTGACCCCCTTTTCTATTTATTTTCATTTCTTTGGACTCCGCAAGAATTTGCCGATATTTCCAATTCAATCGTCTTGTCTTGTTCCTAGATGTTCTATAGGAAAAAATTGTCATTTCGTTCCTCTACGGAGAACCTTTTATTCTAAATTCTAAATCACAGGATCTAAATTCTAAATCACAGGATAAGAAAAAAATTCACTATCTTTCTTTGATTACAGGATCAAGATTCATTTANNNNTTATCTATACTATATAATATATTCNNNNNNTATAGATATTTCTATTCTATATTTATATTTATAGCTATCAGATCGTGGCTTGATGTACCAAAAATTTCCATTTCGTTGCATCCAATATTTTTGTTCCGACCATCGTATGAAGAAAGCATGCAAGATAAATACTATCATTTCCAATCTCCTATTTTAATTTTAATATTGTANNNNNNTTGAAGTGAAGTAAAAATTTGGAAACCCTCTCTTTTCTAACAGGGAAAAATAAATCAAAAAATATTAGTAATTTAGTCTACATAAAAATTAGAATATAAAGAGAGTTCTTTTTCTTAATCTCATGAACAAGATCTAAGAATCCATTTAGTTGATGAAAGAACATGGGGGGGGGCAGGCCTGAGGATCAACCGGTAGTGGGTNNNNGGAAGGGGATTGCTTTTTCCTTGACCAATTCTTTCAAAAAACGAATCGGATTCATGGGTCATAATAAGATAATTCATGATTCAGATGCTTAATAATAAGATAATAAGAAGGAATAATCAAATTGAATTCATGAATTTACCCGGCAATTTATGGTCCAATAAAGGATTTTTATCTTCGAAACCCATTGGAAGGGGTAGTGCACGAGAAAAAAAATCATGCGGAAATGATCGACTCCTTGGATGCCCCAAAATACTATGAGTGTCGGAA

050_20180610_01_04_trnT(GUU)-comp-psbD-comp CATAGAGATGAAATTGGAACAAGTTGACCCCCTTTTCTATTTATTTTCATTTCTTTGGACTCCGCAAGAATTTGCCGATATTTCCAATTCAATCGTCTTGTCTTGTTCCTAGATGTTCTATAGGAAAAAATTGTCATTTCGTTCCTCTACGGAGAACCTTTTATNNNNNNNNNNNNNNNNNNNNNTCTAAATTCTAAATCACAGGATAAGAAAAAAATTCACTATCTTTCTTTGATTACAGGATCAAGATTCATTTATATCTTATCTATACTATATAATATATTCNNNNNNTATAGATATTTCTATTCTATATTTAGATTTATAGCTATCAGATCGTGGCTTGATGTACCAAAAATTTCCATTTCGTTGCATCCAATATTTTTGTTCCGACCATCGTATGAAGAAAGCATGCAAGATAAATACTATCATTTCCAATCTCCTATTTTAATTTTAATATTGTANNNNNNTTGAAGTGAAGTAAAAATTTGGAAACCCTCTCTTTTCTAACAGGGAAAAATAAATCAAAAAATATTAGTAATTTAGTCTACATAAAAATTAGAATATAAAGAGAGTTCTTTTTCTTAATCTCATGAACAAGATCTAAGAATCCATTTAGTTGATGAAAGAACATGGGGGGGGGCAGGCCTGAGGATCAACCGGTAGTGGGTNNNNGGAAGGGGATTGCTTTTTCCTTGACCAATTCTTTCAAAAAACGAATCGGATTCATGGGTCATAATAAGATAATTCATGATTCAGATGCTTAATAATAAGATAATAAGAAGGAATAATCAAATTGAATTCATGAATTTACCCGGCAATTTATGGTCCAATAAAGGATTTTTATCTTCGAAACCCATTGGAAGGGGTAGTGCACGAGAAAAAAAATCATGCGGAAATGATCGACTCCTTGGATGCCCCAAAATACTATGAGTGTCGGAA

051_20180610_01_06_trnT(GUU)-comp-psbD-comp CATAGAGATGAAATTGGAACAAGTTGACCCCCTTTTCTATTTATTTTCATTTCTTTGGACTCCGCAAGAATTTGCCGATATTTCCAATTCAATCGTCTTGTCTTGTTCCTAGATGTTCTATAGGAAAAAATTGTCATTTCGTTCCTCTACGGAGAACCTTTTATTCTAAATTCTAAATCACAGGATCTAAATTCTAAATCACAGGATAAGAAAAAAATTCACTATCTTTCTTTGATTACAGGATCAAGATTCATTTANNNNTTATCTATACTATATAATATATTCNNNNNNTATAGATATTTCTATTCTATATTTATATTTATAGCTATCAGATCGTGGCTTGATGTACCAAAAATTTCCATTTCGTTGCATCCAATATTTTTGTTCCGACCATCGTATGAAGAAAGCATGCAAGATAAATACTATCATTTCCAATCTCCTATTTTAATTTTAATATTGTANNNNNNTTGAAGTGAAGTAAAAATTTGGAAACCCTCTCTTTTCTAACAGGGAAAAATAAATCAAAAAATATTAGTAATTTAGTCTACATAAAAATTAGAATATAAAGAGAGTTCTTTTTCTTAATCTCATGAACAAGATCTAAGAATCCATTTAGTTGATGAAAGAACATGGGGGGGGGCAGGCCTGAGGATCAACCGGTAGTGGGTNNNNGGAAGGGGATTGCTTTTTCCTTGACCAATTCTTTCAAAAAACGAATCGGATTCATGGGTCATAATAAGATAATTCATGATTCAGATGCTTAATAATAAGATAATAAGAAGGAATAATCAAATTGAATTCATGAATTTACCCGGCAATTTATGGTCCAATAAAGGATTTTTATCTTCGAAACCCATTGGAAGGGGTAGTGCACGAGAAAAAAAATCATGCGGAAATGATCGACTCCTTGGATGCCCCAAAATACTATGAGTGTCGGAA

052_20180622_02_01_trnT(GUU)-comp-psbD-comp CATAGAGATGAAATTGGAACAAGTTGACCCCCTTTTCTATTTATTTTCATTTCTTTGGACTCCGCAAGAATTTGCCGATATTTCCAATTCAATCGTCTTGTCTTGTTCCTAGATGTTCTATAGGAAAAAATTGTCATTTCGTTCCTCTACGGAGAACCTTTTATTCTAAATTCTAAATCACAGGATCTAAATTCTAAATCACAGGATAAGAAAAAAATTCACTATCTTTCTTTGATTACAGGATCAAGATTCATTTANNNNTTATCTATACTATATAATATATTCNNNNNNTATAGATATTTCTATTCTATATTTATATTTATAGCTATCAGATCGTGGCTTGATGTACCAAAAATTTCCATTTCGTTGCATCCAATATTTTTGTTCCGACCATCGTATGAAGAAAGCATGCAAGATAAATACTATCATTTCCAATCTCCTATTTTAATTTTAATATTGTANNNNNNTTGAAGTGAAGTAAAAATTTGGAAACCCTCTCTTTTCTAACAGGGAAAAATAAATCAAAAAATATTAGTAATTTAGTCTACATAAAAATTAGAATATAAAGAGAGTTCTTTTTCTTAATCTCATGAACAAGATCTAAGAATCCATTTAGTTGATGAAAGAACATGGGGGGGGGCAGGCCTGAGGATCAACCGGTAGTGGGTNNNNGGAAGGGGATTGCTTTTTCCTTGACCAATTCTTTCAAAAAACGAATCGGATTCATGGGTCATAATAAGATAATTCATGATTCAGATGCTTAATAATAAGATAATAAGAAGGAATAATCAAATTGAATTCATGAATTTACCCGGCAATTTATGGTCCAATAAAGGATTTTTATCTTCGAAACCCATTGGAAGGGGTAGTGCACGAGAAAAAAAATCATGCGGAAATGATCGACTCCTTGGATGCCCCAAAATACTATGAGTGTCGGAA

053_20180622_02_02_trnT(GUU)-comp-psbD-comp CATAGAGATGAAATTGGAACAAGTTGACCCCCTTTTCTATTTATTTTCATTTCTTTGGACTCCGCAAGAATTTGCCGATATTTCCAATTCAATCGTCTTGTCTTGTTCCTAGATGTTCTATAGGAAAAAATTGTCATTTCGTTCCTCTACGGAGAACCTTTTATTCTAAATTCTAAATCACAGGATCTAAATTCTAAATCACAGGATAAGAAAAAAATTCACTATCTTTCTTTGATTACAGGATCAAGATTCATTTANNNNTTATCTATACTATATAATATATTCNNNNNNTATAGATATTTCTATTCTATATTTATATTTATAGCTATCAGATCGTGGCTTGATGTACCAAAAATTTCCATTTCGTTGCATCCAATATTTTTGTTCCGACCATCGTATGAAGAAAGCATGCAAGATAAATACTATCATTTCCAATCTCCTATTTTAATTTTAATATTGTANNNNNNTTGAAGTGAAGTAAAAATTTGGAAACCCTCTCTTTTCTAACAGGGAAAAATAAATCAAAAAATATTAGTAATTTAGTCTACATAAAAATTAGAATATAAAGAGAGTTCTTTTTCTTAATCTCATGAACAAGATCTAAGAATCCATTTAGTTGATGAAAGAACATGGGGGGGGGCAGGCCTGAGGATCAACCGGTAGTGGGTNNNNGGAAGGGGATTGCTTTTTCCTTGACCAATTCTTTCAAAAAACGAATCGGATTCATGGGTCATAATAAGATAATTCATGATTCAGATGCTTAATAATAAGATAATAAGAAGGAATAATCAAATTGAATTCATGAATTTACCCGGCAATTTATGGTCCAATAAAGGATTTTTATCTTCGAAACCCATTGGAAGGGGTAGTGCACGAGAAAAAAAATCATGCGGAAATGATCGACTCCTTGGATGCCCCAAAATACTATGAGTGTCGGAA

054_20180622_02_03_trnT(GUU)-comp-psbD-comp CATAGAGATGAAATTGGAACAAGTTGACCCCCTTTTCTATTTATTTTCATTTCTTTGGACTCCGCAAGAATTTGCCGATATTTCCAATTCAATCGTCTTGTCTTGTTCCTAGATGTTCTATAGGAAAAAATTGTCATTTCGTTCCTCTACGGAGAACCTTTTATTCTAAATTCTAAATCACAGGATCTAAATTCTAAATCACAGGATAAGAAAAAAATTCACTATCTTTCTTTGATTACAGGATCAAGATTCATTTANNNNTTATCTATACTATATAATATATTCNNNNNNTATAGATATTTCTATTCTATATTTATATTTATAGCTATCAGATCGTGGCTTGATGTACCAAAAATTTCCATTTCGTTGCATCCAATATTTTTGTTCCGACCATCGTATGAAGAAAGCATGCAAGATAAATACTATCATTTCCAATCTCCTATTTTAATTTTAATATTGTANNNNNNTTGAAGTGAAGTAAAAATTTGGAAACCCTCTCTTTTCTAACAGGGAAAAATAAATCAAAAAATATTAGTAATTTAGTCTACATAAAAATTAGAATATAAAGAGAGTTCTTTTTCTTAATCTCATGAACAAGATCTAAGAATCCATTTAGTTGATGAAAGAACATGGGGGGGGGCAGGCCTGAGGATCAACCGGTAGTGGGTNNNNGGAAGGGGATTGCTTTTTCCTTGACCAATTCTTTCAAAAAACGAATCGGATTCATGGGTCATAATAAGATAATTCATGATTCAGATGCTTAATAATAAGATAATAAGAAGGAATAATCAAATTGAATTCATGAATTTACCCGGCAATTTATGGTCCAATAAAGGATTTTTATCTTCGAAACCCATTGGAAGGGGTAGTGCACGAGAAAAAAAATCATGCGGAAATGATCGACTCCTTGGATGCCCCAAAATACTATGAGTGTCGGAA

055_20180622_02_04_trnT(GUU)-comp-psbD-comp CATAGAGATGAAATTGGAACAAGTTGACCCCCTTTTCTATTTATTTTCATTTCTTTGGACTCCGCAAGAATTTGCCGATATTTCCAATTCAATCGTCTTGTCTTGTTCCTAGATGTTCTATAGGAAAAAATTGTCATTTCGTTCCTCTACGGAGAACCTTTTATTCTAAATTCTAAATCACAGGATCTAAATTCTAAATCACAGGATAAGAAAAAAATTCACTATCTTTCTTTGATTACAGGATCAAGATTCATTTANNNNTTATCTATACTATATAATATATTCNNNNNNTATAGATATTTCTATTCTATATTTATATTTATAGCTATCAGATCGTGGCTTGATGTACCAAAAATTTCCATTTCGTTGCATCCAATATTTTTGTTCCGACCATCGTATGAAGAAAGCATGCAAGATAAATACTATCATTTCCAATCTCCTATTTTAATTTTAATATTGTANNNNNNTTGAAGTGAAGTAAAAATTTGGAAACCCTCTCTTTTCTAACAGGGAAAAATAAATCAAAAAATATTAGTAATTTAGTCTACATAAAAATTAGAATATAAAGAGAGTTCTTTTTCTTAATCTCATGAACAAGATCTAAGAATCCATTTAGTTGATGAAAGAACATGGGGGGGGGCAGGCCTGAGGATCAACCGGTAGTGGGTNNNNGGAAGGGGATTGCTTTTTCCTTGACCAATTCTTTCAAAAAACGAATCGGATTCATGGGTCATAATAAGATAATTCATGATTCAGATGCTTAATAATAAGATAATAAGAAGGAATAATCAAATTGAATTCATGAATTTACCCGGCAATTTATGGTCCAATAAAGGATTTTTATCTTCGAAACCCATTGGAAGGGGTAGTGCACGAGAAAAAAAATCATGCGGAAATGATCGACTCCTTGGATGCCCCAAAATACTATGAGTGTCGGAA

056_20180622_02_05_trnT(GUU)-comp-psbD-comp CATAGAGATGAAATTGGAACAAGTTGACCCCCTTTTCTATTTATTTTCATTTCTTTGGACTCCGCAAGAATTTGCCGATATTTCCAATTCAATCGTCTTGTCTTGTTCCTAGATGTTCTATAGGAAAAAATTGTCATTTCGTTCCTCTACGGAGAACCTTTTATTCTAAATTCTAAATCACAGGATCTAAATTCTAAATCACAGGATAAGAAAAAAATTCACTATCTTTCTTTGATTACAGGATCAAGATTCATTTANNNNTTATCTATACTATATAATATATTCNNNNNNTATAGATATTTCTATTCTATATTTATATTTATAGCTATCAGATCGTGGCTTGATGTACCAAAAATTTCCATTTCGTTGCATCCAATATTTTTGTTCCGACCATCGTATGAAGAAAGCATGCAAGATAAATACTATCATTTCCAATCTCCTATTTTAATTTTAATATTGTANNNNNNTTGAAGTGAAGTAAAAATTTGGAAACCCTCTCTTTTCTAACAGGGAAAAATAAATCAAAAAATATTAGTAATTTAGTCTACATAAAAATTAGAATATAAAGAGAGTTCTTTTTCTTAATCTCATGAACAAGATCTAAGAATCCATTTAGTTGATGAAAGAACATGGGGGGGGGCAGGCCTGAGGATCAACCGGTAGTGGGTNNNNGGAAGGGGATTGCTTTTTCCTTGACCAATTCTTTCAAAAAACGAATCGGATTCATGGGTCATAATAAGATAATTCATGATTCAGATGCTTAATAATAAGATAATAAGAAGGAATAATCAAATTGAATTCATGAATTTACCCGGCAATTTATGGTCCAATAAAGGATTTTTATCTTCGAAACCCATTGGAAGGGGTAGTGCACGAGAAAAAAAATCATGCGGAAATGATCGACTCCTTGGATGCCCCAAAATACTATGAGTGTCGGAA

057_20180623_04_01_trnT(GUU)-comp-psbD-comp CATAGAGATGAAATTGGAACAAGTTGACCCCCTTTTCTATTTATTTTCATTTCTTTGGACTCCGCAAGAATTTGCCGATATTTCCAATTCAATCGTCTTGTCTTGTTCCTAGATGTTCTATAGGAAAAAATTGTCATTTCGTTCCTCTACGGAGAACCTTTTATTCTAAATTCTAAATCACAGGATCTAAATTCTAAATCACAGGATAAGAAAAAAATTCACTATCTTTCTTTGATTACAGGATCAAGATTCATTTANNNNTTATCTATACTATATAATATATTCNNNNNNTATAGATATTTCTATTCTATATTTATATTTATAGCTATCAGATCGTGGCTTGATGTACCAAAAATTTCCATTTCGTTGCATCCAATATTTTTGTTCCGACCATCGTATGAAGAAAGCATGCAAGATAAATACTATCATTTCCAATCTCCTATTTTAATTTTAATATTGTANNNNNNTTGAAGTGAAGTAAAAATTTGGAAACCCTCTCTTTTCTAACAGGGAAAAATAAATCAAAAAATATTAGTAATTTAGTCTACATAAAAATTAGAATATAAAGAGAGTTCTTTTTCTTAATCTCATGAACAAGATCTAAGAATCCATTTAGTTGATGAAAGAACATGGGGGGGGGCAGGCCTGAGGATCAACCGGTAGTGGGTNNNNGGAAGGGGATTGCTTTTTCCTTGACCAATTCTTTCAAAAAACGAATCGGATTCATGGGTCATAATAAGATAATTCATGATTCAGATGCTTAATAATAAGATAATAAGAAGGAATAATCAAATTGAATTCATGAATTTACCCGGCAATTTATGGTCCAATAAAGGATTTTTATCTTCGAAACCCATTGGAAGGGGTAGTGCACGAGAAAAAAAATCATGCGGAAATGATCGACTCCTTGGATGCCCCAAAATACTATGAGTGTCGGAA

058_20180623_04_02_trnT(GUU)-comp-psbD-comp CATAGAGATGAAATTGGAACAAGTTGACCCCCTTTTCTATTTATTTTCATTTCTTTGGACTCCGCAAGAATTTGCCGATATTTCCAATTCAATCGTCTTGTCTTGTTCCTAGATGTTCTATAGGAAAAAATTGTCATTTCGTTCCTCTACGGAGAACCTTTTATTCTAAATTCTAAATCACAGGATCTAAATTCTAAATCACAGGATAAGAAAAAAATTCACTATCTTTCTTTGATTACAGGATCAAGATTCATTTANNNNTTATCTATACTATATAATATATTCNNNNNNTATAGATATTTCTATTCTATATTTATATTTATAGCTATCAGATCGTGGCTTGATGTACCAAAAATTTCCATTTCGTTGCATCCAATATTTTTGTTCCGACCATCGTATGAAGAAAGCATGCAAGATAAATACTATCATTTCCAATCTCCTATTTTAATTTTAATATTGTANNNNNNTTGAAGTGAAGTAAAAATTTGGAAACCCTCTCTTTTCTAACAGGGAAAAATAAATCAAAAAATATTAGTAATTTAGTCTACATAAAAATTAGAATATAAAGAGAGTTCTTTTTCTTAATCTCATGAACAAGATCTAAGAATCCATTTAGTTGATGAAAGAACATGGGGGGGGGCAGGCCTGAGGATCAACCGGTAGTGGGTNNNNGGAAGGGGATTGCTTTTTCCTTGACCAATTCTTTCAAAAAACGAATCGGATTCATGGGTCATAATAAGATAATTCATGATTCAGATGCTTAATAATAAGATAATAAGAAGGAATAATCAAATTGAATTCATGAATTTACCCGGCAATTTATGGTCCAATAAAGGATTTTTATCTTCGAAACCCATTGGAAGGGGTAGTGCACGAGAAAAAAAATCATGCGGAAATGATCGACTCCTTGGATGCCCCAAAATACTATGAGTGTCGGAA

059_20180623_04_03_trnT(GUU)-comp-psbD-comp CATAGAGATGAAATTGGAACAAGTTGACCCCCTTTTCTATTTATTTTCATTTCTTTGGACTCCGCAAGAATTTGCCGATATTTCCAATTCAATCGTCTTGTCTTGTTCCTAGATGTTCTATAGGAAAAAATTGTCATTTCGTTCCTCTACGGAGAACCTTTTATTCTAAATTCTAAATCACAGGATCTAAATTCTAAATCACAGGATAAGAAAAAAATTCACTATCTTTCTTTGATTACAGGATCAAGATTCATTTANNNNTTATCTATACTATATAATATATTCNNNNNNTATAGATATTTCTATTCTATATTTATATTTATAGCTATCAGATCGTGGCTTGATGTACCAAAAATTTCCATTTCGTTGCATCCAATATTTTTGTTCCGACCATCGTATGAAGAAAGCATGCAAGATAAATACTATCATTTCCAATCTCCTATTTTAATTTTAATATTGTANNNNNNTTGAAGTGAAGTAAAAATTTGGAAACCCTCTCTTTTCTAACAGGGAAAAATAAATCAAAAAATATTAGTAATTTAGTCTACATAAAAATTAGAATATAAAGAGAGTTCTTTTTCTTAATCTCATGAACAAGATCTAAGAATCCATTTAGTTGATGAAAGAACATGGGGGGGGGCAGGCCTGAGGATCAACCGGTAGTGGGTNNNNGGAAGGGGATTGCTTTTTCCTTGACCAATTCTTTCAAAAAACGAATCGGATTCATGGGTCATAATAAGATAATTCATGATTCAGATGCTTAATAATAAGATAATAAGAAGGAATAATCAAATTGAATTCATGAATTTACCCGGCAATTTATGGTCCAATAAAGGATTTTTATCTTCGAAACCCATTGGAAGGGGTAGTGCACGAGAAAAAAAATCATGCGGAAATGATCGACTCCTTGGATGCCCCAAAATACTATGAGTGTCGGAA

060_20180623_04_04_trnT(GUU)-comp-psbD-comp CATAGAGATGAAATTGGAACAAGTTGACCCCCTTTTCTATTTATTTTCATTTCTTTGGACTCCGCAAGAATTTGCCGATATTTCCAATTCAATCGTCTTGTCTTGTTCCTAGATGTTCTATAGGAAAAAATTGTCATTTCGTTCCTCTACGGAGAACCTTTTATTCTAAATTCTAAATCACAGGATCTAAATTCTAAATCACAGGATAAGAAAAAAATTCACTATCTTTCTTTGATTACAGGATCAAGATTCATTTANNNNTTATCTATACTATATAATATATTCNNNNNNTATAGATATTTCTATTCTATATTTATATTTATAGCTATCAGATCGTGGCTTGATGTACCAAAAATTTCCATTTCGTTGCATCCAATATTTTTGTTCCGACCATCGTATGAAGAAAGCATGCAAGATAAATACTATCATTTCCAATCTCCTATTTTAATTTTAATATTGTANNNNNNTTGAAGTGAAGTAAAAATTTGGAAACCCTCTCTTTTCTAACAGGGAAAAATAAATCAAAAAATATTAGTAATTTAGTCTACATAAAAATTAGAATATAAAGAGAGTTCTTTTTCTTAATCTCATGAACAAGATCTAAGAATCCATTTAGTTGATGAAAGAACATGGGGGGGGGCAGGCCTGAGGATCAACCGGTAGTGGGTNNNNGGAAGGGGATTGCTTTTTCCTTGACCAATTCTTTCAAAAAACGAATCGGATTCATGGGTCATAATAAGATAATTCATGATTCAGATGCTTAATAATAAGATAATAAGAAGGAATAATCAAATTGAATTCATGAATTTACCCGGCAATTTATGGTCCAATAAAGGATTTTTATCTTCGAAACCCATTGGAAGGGGTAGTGCACGAGAAAAAAAATCATGCGGAAATGATCGACTCCTTGGATGCCCCAAAATACTATGAGTGTCGGAA

061_20180623_04_05_trnT(GUU)-comp-psbD-comp CATAGAGATGAAATTGGAACAAGTTGACCCCCTTTTCTATTTATTTTCATTTCTTTGGACTCCGCAAGAATTTGCCGATATTTCCAATTCAATCGTCTTGTCTTGTTCCTAGATGTTCTATAGGAAAAAATTGTCATTTCGTTCCTCTACGGAGAACCTTTTATTCTAAATTCTAAATCACAGGATCTAAATTCTAAATCACAGGATAAGAAAAAAATTCACTATCTTTCTTTGATTACAGGATCAAGATTCATTTANNNNTTATCTATACTATATAATATATTCNNNNNNTATAGATATTTCTATTCTATATTTATATTTATAGCTATCAGATCGTGGCTTGATGTACCAAAAATTTCCATTTCGTTGCATCCAATATTTTTGTTCCGACCATCGTATGAAGAAAGCATGCAAGATAAATACTATCATTTCCAATCTCCTATTTTAATTTTAATATTGTANNNNNNTTGAAGTGAAGTAAAAATTTGGAAACCCTCTCTTTTCTAACAGGGAAAAATAAATCAAAAAATATTAGTAATTTAGTCTACATAAAAATTAGAATATAAAGAGAGTTCTTTTTCTTAATCTCATGAACAAGATCTAAGAATCCATTTAGTTGATGAAAGAACATGGGGGGGGGCAGGCCTGAGGATCAACCGGTAGTGGGTNNNNGGAAGGGGATTGCTTTTTCCTTGACCAATTCTTTCAAAAAACGAATCGGATTCATGGGTCATAATAAGATAATTCATGATTCAGATGCTTAATAATAAGATAATAAGAAGGAATAATCAAATTGAATTCATGAATTTACCCGGCAATTTATGGTCCAATAAAGGATTTTTATCTTCGAAACCCATTGGAAGGGGTAGTGCACGAGAAAAAAAATCATGCGGAAATGATCGACTCCTTGGATGCCCCAAAATACTATGAGTGTCGGAA

062_20180713_02_01_trnT(GUU)-comp-psbD-comp CATAGAGATGAAATTGGAACAAGTTGACCCCCTTTTCTATTTATTTTCATTTCTTTGGACTCCGCAAGAATTTGCCGATATTTCCAATTCAATCGTCTTGTCTTGTTCCTAGATGTTCTATAGGAAAAAATTGTCATTTCGTTCCTCTACGGAGAACCTTTTATTCTAAATTCTAAATCACAGGATCTAAATTCTAAATCACAGGATAAGAAAAAAATTCACTATCTTTCTTTGATTACAGGATCAAGATTCATTTANNNNTTATCTATACTATATAATATATTCNNNNNNTATAGATATTTCTATTCTATATTTAGATTTATAGCTATCAGATCGTGGCTTGATGTACCAAAAATTTCCATTTCGTTGCATCCAATATTTTTGTTCCGACCATCGTATGAAGAAAGCATGCAAGATAAATACTATCATTTCCAATCTCCTATTTTAATTTTAATATTGTANNNNNNTTGAAGTGAAGTAAAAATTTGGAAACCCTCTCTTTTCTAACAGGGAAAAATAAATCAAAAAATATTAGTAATTTAGTCTACATAAAAATTAGAATATAAAGAGAGTTCTTTTTCTTAATCTCATGAACAAGATCTAAGAATCCATTTAGTTGATGAAAGAACATGGGGGGGGGCAGGCCTGAGGATCAACCGGTAGTGGGTTGGGTGAAGGGGATTGCTTTTTCCTTGACCAATTCTTTCAAAAAACGAATCGGATTCATGGGTCATAATAAGATAATTCATGATTCAGATGCTTAATAATAAGATAATAAGAAGGAATAATCAAATTGAATTCATGAATTTACCCGGCAATTTATGGTCCAATAAAGGATTTTTATCTTCGAAACCCATTGGAAGGGGTAGTGCACGAGAAAAAAAATCATGCGGAAATGATCGACTCCTTGGATGCCCCAAAATACTATGAGTGTCGGAA

063_20180713_02_02_trnT(GUU)-comp-psbD-comp CATAGAGATGAAATTGGAACAAGTTGACCCCCTTTTCTATTTATTTTCATTTCTTTGGACTCCGCAAGAATTTGCCGATATTTCCAATTCAATCGTCTTGTCTTGTTCCTAGATGTTCTATAGGAAAAAATTGTCATTTCGTTCCTCTACGGAGAACCTTTTATTCTAAATTCTAAATCACAGGATCTAAATTCTAAATCACAGGATAAGAAAAAAATTCACTATCTTTCTTTGATTACAGGATCAAGATTCATTTANNNNTTATCTATACTATATAATATATTCNNNNNNTATAGATATTTCTATTCTATATTTAGATTTATAGCTATCAGATCGTGGCTTGATGTACCAAAAATTTCCATTTCGTTGCATCCAATATTTTTGTTCCGACCATCGTATGAAGAAAGCATGCAAGATAAATACTATCATTTCCAATCTCCTATTTTAATTTTAATATTGTANNNNNNTTGAAGTGAAGTAAAAATTTGGAAACCCTCTCTTTTCTAACAGGGAAAAATAAATCAAAAAATATTAGTAATTTAGTCTACATAAAAATTAGAATATAAAGAGAGTTCTTTTTCTTAATCTCATGAACAAGATCTAAGAATCCATTTAGTTGATGAAAGAACATGGGGGGGGGCAGGCCTGAGGATCAACCGGTAGTGGGTTGGGTGAAGGGGATTGCTTTTTCCTTGACCAATTCTTTCAAAAAACGAATCGGATTCATGGGTCATAATAAGATAATTCATGATTCAGATGCTTAATAATAAGATAATAAGAAGGAATAATCAAATTGAATTCATGAATTTACCCGGCAATTTATGGTCCAATAAAGGATTTTTATCTTCGAAACCCATTGGAAGGGGTAGTGCACGAGAAAAAAAATCATGCGGAAATGATCGACTCCTTGGATGCCCCAAAATACTATGAGTGTCGGAA

064_20180713_02_03_trnT(GUU)-comp-psbD-comp CATAGAGATGAAATTGGAACAAGTTGACCCCCTTTTCTATTTATTTTCATTTCTTTGGACTCCGCAAGAATTTGCCGATATTTCCAATTCAATCGTCTTGTCTTGTTCCTAGATGTTCTATAGGAAAAAATTGTCATTTCGTTCCTCTACGGAGAACCTTTTATTCTAAATTCTAAATCACAGGATCTAAATTCTAAATCACAGGATAAGAAAAAAATTCACTATCTTTCTTTGATTACAGGATCAAGATTCATTTANNNNTTATCTATACTATATAATATATTCNNNNNNTATAGATATTTCTATTCTATATTTAGATTTATAGCTATCAGATCGTGGCTTGATGTACCAAAAATTTCCATTTCGTTGCATCCAATATTTTTGTTCCGACCATCGTATGAAGAAAGCATGCAAGATAAATACTATCATTTCCAATCTCCTATTTTAATTTTAATATTGTANNNNNNTTGAAGTGAAGTAAAAATTTGGAAACCCTCTCTTTTCTAACAGGGAAAAATAAATCAAAAAATATTAGTAATTTAGTCTACATAAAAATTAGAATATAAAGAGAGTTCTTTTTCTTAATCTCATGAACAAGATCTAAGAATCCATTTAGTTGATGAAAGAACATGGGGGGGGGCAGGCCTGAGGATCAACCGGTAGTGGGTNNNNGGAAGGGGATTGCTTTTTCCTTGACCAATTCTTTCAAAAAACGAATCGGATTCATGGGTCATAATAAGATAATTCATGATTCAGATGCTTAATAATAAGATAATAAGAAGGAATAATCAAATTGAATTCATGAATTTACCCGGCAATTTATGGTCCAATAAAGGATTTTTATCTTCGAAACCCATTGGAAGGGGTAGTGCACGAGAAAAAAAATCATGCGGAAATGATCGACTCCTTGGATGCCCCAAAATACTATGAGTGTCGGAA

065_20180713_02_04_trnT(GUU)-comp-psbD-comp CATAGAGATGAAATTGGAACAAGTTGACCCCCTTTTCTATTTATTTTCATTTCTTTGGACTCCGCAAGAATTTGCCGATATTTCCAATTCAATCGTCTTGTCTTGTTCCTAGATGTTCTATAGGAAAAAATTGTCATTTCGTTCCTCTACGGAGAACCTTTTATTCTAAATTCTAAATCACAGGATCTAAATTCTAAATCACAGGATAAGAAAAAAATTCACTATCTTTCTTTGATTACAGGATCAAGATTCATTTANNNNTTATCTATACTATATAATATATTCNNNNNNTATAGATATTTCTATTCTATATTTAGATTTATAGCTATCAGATCGTGGCTTGATGTACCAAAAATTTCCATTTCGTTGCATCCAATATTTTTGTTCCGACCATCGTATGAAGAAAGCATGCAAGATAAATACTATCATTTCCAATCTCCTATTTTAATTTTAATATTGTANNNNNNTTGAAGTGAAGTAAAAATTTGGAAACCCTCTCTTTTCTAACAGGGAAAAATAAATCAAAAAATATTAGTAATTTAGTCTACATAAAAATTAGAATATAAAGAGAGTTCTTTTTCTTAATCTCATGAACAAGATCTAAGAATCCATTTAGTTGATGAAAGAACATGGGGGGGGGCAGGCCTGAGGATCAACCGGTAGTGGGTNNNNGGAAGGGGATTGCTTTTTCCTTGACCAATTCTTTCAAAAAACGAATCGGATTCATGGGTCATAATAAGATAATTCATGATTCAGATGCTTAATAATAAGATAATAAGAAGGAATAATCAAATTGAATTCATGAATTTACCCGGCAATTTATGGTCCAATAAAGGATTTTTATCTTCGAAACCCATTGGAAGGGGTAGTGCACGAGAAAAAAAATCATGCGGAAATGATCGACTCCTTGGATGCCCCAAAATACTATGAGTGTCGGAA

066_20180713_02_05_trnT(GUU)-comp-psbD-comp CATAGAGATGAAATTGGAACAAGTTGACCCCCTTTTCTATTTATTTTCATTTCTTTGGACTCCGCAAGAATTTGCCGATATTTCCAATTCAATCGTCTTGTCTTGTTCCTAGATGTTCTATAGGAAAAAATTGTCATTTCGTTCCTCTACGGAGAACCTTTTATTCTAAATTCTAAATCACAGGATCTAAATTCTAAATCACAGGATAAGAAAAAAATTCACTATCTTTCTTTGATTACAGGATCAAGATTCATTTANNNNTTATCTATACTATATAATATATTCNNNNNNTATAGATATTTCTATTCTATATTTAGATTTATAGCTATCAGATCGTGGCTTGATGTACCAAAAATTTCCATTTCGTTGCATCCAATATTTTTGTTCCGACCATCGTATGAAGAAAGCATGCAAGATAAATACTATCATTTCCAATCTCCTATTTTAATTTTAATATTGTANNNNNNTTGAAGTGAAGTAAAAATTTGGAAACCCTCTCTTTTCTAACAGGGAAAAATAAATCAAAAAATATTAGTAATTTAGTCTACATAAAAATTAGAATATAAAGAGAGTTCTTTTTCTTAATCTCATGAACAAGATCTAAGAATCCATTTAGTTGATGAAAGAACATGGGGGGGGGCAGGCCTGAGGATCAACCGGTAGTGGGTNNNNGGAAGGGGATTGCTTTTTCCTTGACCAATTCTTTCAAAAAACGAATCGGATTCATGGGTCATAATAAGATAATTCATGATTCAGATGCTTAATAATAAGATAATAAGAAGGAATAATCAAATTGAATTCATGAATTTACCCGGCAATTTATGGTCCAATAAAGGATTTTTATCTTCGAAACCCATTGGAAGGGGTAGTGCACGAGAAAAAAAATCATGCGGAAATGATCGACTCCTTGGATGCCCCAAAATACTATGAGTGTCGGAA

067_20180713_03_01_trnT(GUU)-comp-psbD-comp CATAGAGATGAAATTGGAACAAGTTGACCCCCTTTTCTATTTATTTTCATTTCTTTGGACTCCGCAAGAATTTGCCGATATTTCCAATTCAATCGTCTTGTCTTGTTCCTAGATGTTCTATAGGAAAAAATTGTCATTTCGTTCCTCTACGGAGAACCTTTTATTCTAAATTCTAAATCACAGGATCTAAATTCTAAATCACAGGATAAGAAAAAAATTCACTATCTTTCTTTGATTACAGGATCAAGATTCATTTANNNNTTATCTATACTATATAATATATTCNNNNNNTATAGATATTTCTATTCTATATTTAGATTTATAGCTATCAGATCGTGGCTTGATGTACCAAAAATTTCCATTTCGTTGCATCCAATATTTTTGTTCCGACCATCGTATGAAGAAAGCATGCAAGATAAATACTATCATTTCCAATCTCCTATTTTAATTTTAATATTGTANNNNNNTTGAAGTGAAGTAAAAATTTGGAAACCCTCTCTTTTCTAACAGGGAAAAATAAATCAAAAAATATTAGTAATTTAGTCTACATAAAAATTAGAATATAAAGAGAGTTCTTTTTCTTAATCTCATGAACAAGATCTAAGAATCCATTTAGTTGATGAAAGAACATGGGGGGGGGCAGGCCTGAGGATCAACCGGTAGTGGGTNNNNGGAAGGGGATTGCTTTTTCCTTGACCAATTCTTTCAAAAAACGAATCGGATTCATGGGTCATAATAAGATAATTCATGATTCAGATGCTTAATAATAAGATAATAAGAAGGAATAATCAAATTGAATTCATGAATTTACCCGGCAATTTATGGTCCAATAAAGGATTTTTATCTTCGAAACCCATTGGAAGGGGTAGTGCACGAGAAAAAAAATCATGCGGAAATGATCGACTCCTTGGATGCCCCAAAATACTATGAGTGTCGGAA

068_20180713_03_02_trnT(GUU)-comp-psbD-comp CATAGAGATGAAATTGGAACAAGTTGACCCCCTTTTCTATTTATTTTCATTTCTTTGGACTCCGCAAGAATTTGCCGATATTTCCAATTCAATCGTCTTGTCTTGTTCCTAGATGTTCTATAGGAAAAAATTGTCATTTCGTTCCTCTACGGAGAACCTTTTATTCTAAATTCTAAATCACAGGATCTAAATTCTAAATCACAGGATAAGAAAAAAATTCACTATCTTTCTTTGATTACAGGATCAAGATTCATTTANNNNTTATCTATACTATATAATATATTCNNNNNNTATAGATATTTCTATTCTATATTTAGATTTATAGCTATCAGATCGTGGCTTGATGTACCAAAAATTTCCATTTCGTTGCATCCAATATTTTTGTTCCGACCATCGTATGAAGAAAGCATGCAAGATAAATACTATCATTTCCAATCTCCTATTTTAATTTTAATATTGTANNNNNNTTGAAGTGAAGTAAAAATTTGGAAACCCTCTCTTTTCTAACAGGGAAAAATAAATCAAAAAATATTAGTAATTTAGTCTACATAAAAATTAGAATATAAAGAGAGTTCTTTTTCTTAATCTCATGAACAAGATCTAAGAATCCATTTAGTTGATGAAAGAACATGGGGGGGGGCAGGCCTGAGGATCAACCGGTAGTGGGTNNNNGGAAGGGGATTGCTTTTTCCTTGACCAATTCTTTCAAAAAACGAATCGGATTCATGGGTCATAATAAGATAATTCATGATTCAGATGCTTAATAATAAGATAATAAGAAGGAATAATCAAATTGAATTCATGAATTTACCCGGCAATTTATGGTCCAATAAAGGATTTTTATCTTCGAAACCCATTGGAAGGGGTAGTGCACGAGAAAAAAAATCATGCGGAAATGATCGACTCCTTGGATGCCCCAAAATACTATGAGTGTCGGAA

069_20180713_03_03_trnT(GUU)-comp-psbD-comp CATAGAGATGAAATTGGAACAAGTTGACCCCCTTTTCTATTTATTTTCATTTCTTTGGACTCCGCAAGAATTTGCCGATATTTCCAATTCAATCGTCTTGTCTTGTTCCTAGATGTTCTATAGGAAAAAATTGTCATTTCGTTCCTCTACGGAGAACCTTTTATTCTAAATTCTAAATCACAGGATCTAAATTCTAAATCACAGGATAAGAAAAAAATTCACTATCTTTCTTTGATTACAGGATCAAGATTCATTTANNNNTTATCTATACTATATAATATATTCNNNNNNTATAGATATTTCTATTCTATATTTAGATTTATAGCTATCAGATCGTGGCTTGATGTACCAAAAATTTCCATTTCGTTGCATCCAATATTTTTGTTCCGACCATCGTATGAAGAAAGCATGCAAGATAAATACTATCATTTCCAATCTCCTATTTTAATTTTAATATTGTANNNNNNTTGAAGTGAAGTAAAAATTTGGAAACCCTCTCTTTTCTAACAGGGAAAAATAAATCAAAAAATATTAGTAATTTAGTCTACATAAAAATTAGAATATAAAGAGAGTTCTTTTTCTTAATCTCATGAACAAGATCTAAGAATCCATTTAGTTGATGAAAGAACATGGGGGGGGGCAGGCCTGAGGATCAACCGGTAGTGGGTNNNNGGAAGGGGATTGCTTTTTCCTTGACCAATTCTTTCAAAAAACGAATCGGATTCATGGGTCATAATAAGATAATTCATGATTCAGATGCTTAATAATAAGATAATAAGAAGGAATAATCAAATTGAATTCATGAATTTACCCGGCAATTTATGGTCCAATAAAGGATTTTTATCTTCGAAACCCATTGGAAGGGGTAGTGCACGAGAAAAAAAATCATGCGGAAATGATCGACTCCTTGGATGCCCCAAAATACTATGAGTGTCGGAA

070_20180713_03_04_trnT(GUU)-comp-psbD-comp CATAGAGATGAAATTGGAACAAGTTGACCCCCTTTTCTATTTATTTTCATTTCTTTGGACTCCGCAAGAATTTGCCGATATTTCCAATTCAATCGTCTTGTCTTGTTCCTAGATGTTCTATAGGAAAAAATTGTCATTTCGTTCCTCTACGGAGAACCTTTTATTCTAAATTCTAAATCACAGGATCTAAATTCTAAATCACAGGATAAGAAAAAAATTCACTATCTTTCTTTGATTACAGGATCAAGATTCATTTANNNNTTATCTATACTATATAATATATTCNNNNNNTATAGATATTTCTATTCTATATTTAGATTTATAGCTATCAGATCGTGGCTTGATGTACCAAAAATTTCCATTTCGTTGCATCCAATATTTTTGTTCCGACCATCGTATGAAGAAAGCATGCAAGATAAATACTATCATTTCCAATCTCCTATTTTAATTTTAATATTGTANNNNNNTTGAAGTGAAGTAAAAATTTGGAAACCCTCTCTTTTCTAACAGGGAAAAATAAATCAAAAAATATTAGTAATTTAGTCTACATAAAAATTAGAATATAAAGAGAGTTCTTTTTCTTAATCTCATGAACAAGATCTAAGAATCCATTTAGTTGATGAAAGAACATGGGGGGGGGCAGGCCTGAGGATCAACCGGTAGTGGGTNNNNGGAAGGGGATTGCTTTTTCCTTGACCAATTCTTTCAAAAAACGAATCGGATTCATGGGTCATAATAAGATAATTCATGATTCAGATGCTTAATAATAAGATAATAAGAAGGAATAATCAAATTGAATTCATGAATTTACCCGGCAATTTATGGTCCAATAAAGGATTTTTATCTTCGAAACCCATTGGAAGGGGTAGTGCACGAGAAAAAAAATCATGCGGAAATGATCGACTCCTTGGATGCCCCAAAATACTATGAGTGTCGGAA

071_20180713_03_05_trnT(GUU)-comp-psbD-comp CATAGAGATGAAATTGGAACAAGTTGACCCCCTTTTCTATTTATTTTCATTTCTTTGGACTCCGCAAGAATTTGCCGATATTTCCAATTCAATCGTCTTGTCTTGTTCCTAGATGTTCTATAGGAAAAAATTGTCATTTCGTTCCTCTACGGAGAACCTTTTATTCTAAATTCTAAATCACAGGATCTAAATTCTAAATCACAGGATAAGAAAAAAATTCACTATCTTTCTTTGATTACAGGATCAAGATTCATTTANNNNTTATCTATACTATATAATATATTCNNNNNNTATAGATATTTCTATTCTATATTTAGATTTATAGCTATCAGATCGTGGCTTGATGTACCAAAAATTTCCATTTCGTTGCATCCAATATTTTTGTTCCGACCATCGTATGAAGAAAGCATGCAAGATAAATACTATCATTTCCAATCTCCTATTTTAATTTTAATATTGTANNNNNNTTGAAGTGAAGTAAAAATTTGGAAACCCTCTCTTTTCTAACAGGGAAAAATAAATCAAAAAATATTAGTAATTTAGTCTACATAAAAATTAGAATATAAAGAGAGTTCTTTTTCTTAATCTCATGAACAAGATCTAAGAATCCATTTAGTTGATGAAAGAACATGGGGGGGGGCAGGCCTGAGGATCAACCGGTAGTGGGTNNNNGGAAGGGGATTGCTTTTTCCTTGACCAATTCTTTCAAAAAACGAATCGGATTCATGGGTCATAATAAGATAATTCATGATTCAGATGCTTAATAATAAGATAATAAGAAGGAATAATCAAATTGAATTCATGAATTTACCCGGCAATTTATGGTCCAATAAAGGATTTTTATCTTCGAAACCCATTGGAAGGGGTAGTGCACGAGAAAAAAAATCATGCGGAAATGATCGACTCCTTGGATGCCCCAAAATACTATGAGTGTCGGAA

072_20180714_02_01_trnT(GUU)-comp-psbD-comp CATAGAGATGAAATTGGAACAAGTTGACCCCCTTTTCTATTTATTTTCATTTCTTTGGACTCCGCAAGAATTTGCCGATATTTCCAATTCAATCGTCTTGTCTTGTTCCTAGATGTTCTATAGGAAAAAATTGTCATTTCGTTCCTCTACGGAGAACCTTTTATTCTAAATTCTAAATCACAGGATCTAAATTCTAAATCACAGGATAAGAAAAAAATTCACTATCTTTCTTTGATTACAGGATCAAGATTCATTTANNNNTTATCTATACTATATAATATATTCNNNNNNTATAGATATTTCTATTCTATATTTAGATTTATAGCTATCAGATCGTGGCTTGATGTACCAAAAATTTCCATTTCGTTGCATCCAATATTTTTGTTCCGACCATCGTATGAAGAAAGCATGCAAGATAAATACTATCATTTCCAATCTCCTNNNNNNATTTTAATATTGTANNNNNNTTGAAGTGAAGTAAAAATTTGGAAACCCTCTCTTTTCTAACAGGGAAAAATAAATCAAAAAATATTAGTAATTTAGTCTACATAAAAATTAGAATATAAAGAGAGTTCTTTTTCTTAATCTCATGAACAAGATCTAAGAATCCATTTAGTTGATGAAAGAACATGGGGGGGGGCAGGCCTGAGGATCAACCGGTAGTGGGTNNNNGGAAGGGGATTGCTTTTTCCTTGACCAATTCTTTCAAAAAACGAATCGGATTCATGGGTCATAATAAGATAATTCATGATTCAGATGCTTAATAATAAGATAATAAGAAGGAATAATCAAATTGAATTCATGAATTTACCCGGCAATTTATGGTCCAATAAAGGATTTTTATCTTCGAAACCCATTGGAAGGGGTAGTGCACGAGAAAAAAAATCATGCGGAAATGATCGACTCCTTGGATGCCCCAAAATACTATGAGTGTCGGAA

073_20180714_02_02_trnT(GUU)-comp-psbD-comp CATAGAGATGAAATTGGAACAAGTTGACCCCCTTTTCTATTTATTTTCATTTCTTTGGACTCCGCAAGAATTTGCCGATATTTCCAATTCAATCGTCTTGTCTTGTTCCTAGATGTTCTATAGGAAAAAATTGTCATTTCGTTCCTCTACGGAGAACCTTTTATNNNNNNNNNNNNNNNNNNNNNTCTAAATTCTAAATCACAGGATAAGAAAAAAATTCACTATCTTTCTTTGATTACAGGATCAAGATTCATTTANNNNTTATCTATACTATATAATATATTCNNNNNNTATAGATATTTCTATTCTATATTTAGATTTATAGCTATCAGATCGTGGCTTGATGTACCAAAAATTTCCATTTCGTTGCATCCAATATTTTTGTTCCGACCATCGTATGAAGAAAGCATGCAAGATAAATACTATCATTTCCAATCTCCTATTTTAATTTTAATATTGTANNNNNNTTGAAGTGAAGTAAAAATTTGGAAACCCTCTCTTTTCTAACAGGGAAAAATAAATCAAAAAATATTAGTAATTTAGTCTACATAAAAATTAGAATATAAAGAGAGTTCTTTTTCTTAATCTCATGAACAAGATCTAAGAATCCATTTAGTTGATGAAAGAACATGGGGGGGGGCAGGCCTGAGGATCAACCGGTAGTGGGTNNNNGGAAGGGGATTGCTTTTTCCTTGACCAATTCTTTCAAAAAACGAATCGGATTCATGGGTCATAATAAGATAATTCATGATTCAGATGCTTAATAATAAGATAATAAGAAGGAATAATCAAATTGAATTCATGAATTTACCCGGCAATTTATGGTCCAATAAAGGATTTTTATCTTCGAAACCCATTGGAAGGGGTAGTGCACGAGAAAAAAAATCATGCGGAAATGATCGACTCCTTGGATGCCCCAAAATACTATGAGTGTCGGAA

074_20180714_03_01_trnT(GUU)-comp-psbD-comp CATAGAGATGAAATTGGAACAAGTTGACCCCCTTTTCTATTTATTTTCATTTCTTTGGACTCCGCAAGAATTTGCCGATATTTCCAATTCAATCGTCTTGTCTTGTTCCTAGATGTTCTATAGGAAAAAATTGTCATTTCGTTCCTCTACGGAGAACCTTTTATTCTAAATTCTAAATCACAGGATCTAAATTCTAAATCACAGGATAAGAAAAAAATTCACTATCTTTCTTTGATTACAGGATCAAGATTCATTTANNNNTTATCTATACTATATAATATATTCNNNNNNTATAGATATTTCTATTCTATATTTAGATTTATAGCTATCAGATCGTGGCTTGATGTACCAAAAATTTCCATTTCGTTGCATCCAATATTTTTGTTCCGACCATCGTATGAAGAAAGCATGCAAGATAAATACTATCATTTCCAATCTCCTNNNNNNATTTTAATATTGTANNNNNNTTGAAGTGAAGTAAAAATTTGGAAACCCTCTCTTTTCTAACAGGGAAAAATAAATCAAAAAATATTAGTAATTTAGTCTACATAAAAATTAGAATATAAAGAGAGTTCTTTTTCTTAATCTCATGAACAAGATCTAAGAATCCATTTAGTTGATGAAAGAACATGGGGGGGGGCAGGCCTGAGGATCAACCGGTAGTGGGTNNNNGGAAGGGGATTGCTTTTTCCTTGACCAATTCTTTCAAAAAACGAATCGGATTCATGGGTCATAATAAGATAATTCATGATTCAGATGCTTAATAATAAGATAATAAGAAGGAATAATCAAATTGAATTCATGAATTTACCCGGCAATTTATGGTCCAATAAAGGATTTTTATCTTCGAAACCCATTGGAAGGGGTAGTGCACGAGAAAAAAAATCATGCGGAAATGATCGACTCCTTGGATGCCCCAAAATACTATGAGTGTCGGAA

075_20180714_03_02_trnT(GUU)-comp-psbD-comp CATAGAGATGAAATTGGAACAAGTTGACCCCCTTTTCTATTTATTTTCATTTCTTTGGACTCCGCAAGAATTTGCCGATATTTCCAATTCAATCGTCTTGTCTTGTTCCTAGATGTTCTATAGGAAAAAATTGTCATTTCGTTCCTCTACGGAGAACCTTTTATTCTAAATTCTAAATCACAGGATCTAAATTCTAAATCACAGGATAAGAAAAAAATTCACTATCTTTCTTTGATTACAGGATCAAGATTCATTTANNNNTTATCTATACTATATAATATATTCNNNNNNTATAGATATTTCTATTCTATATTTAGATTTATAGCTATCAGATCGTGGCTTGATGTACCAAAAATTTCCATTTCGTTGCATCCAATATTTTTGTTCCGACCATCGTATGAAGAAAGCATGCAAGATAAATACTATCATTTCCAATCTCCTNNNNNNATTTTAATATTGTANNNNNNTTGAAGTGAAGTAAAAATTTGGAAACCCTCTCTTTTCTAACAGGGAAAAATAAATCAAAAAATATTAGTAATTTAGTCTACATAAAAATTAGAATATAAAGAGAGTTCTTTTTCTTAATCTCATGAACAAGATCTAAGAATCCATTTAGTTGATGAAAGAACATGGGGGGGGGCAGGCCTGAGGATCAACCGGTAGTGGGTNNNNGGAAGGGGATTGCTTTTTCCTTGACCAATTCTTTCAAAAAACGAATCGGATTCATGGGTCATAATAAGATAATTCATGATTCAGATGCTTAATAATAAGATAATAAGAAGGAATAATCAAATTGAATTCATGAATTTACCCGGCAATTTATGGTCCAATAAAGGATTTTTATCTTCGAAACCCATTGGAAGGGGTAGTGCACGAGAAAAAAAATCATGCGGAAATGATCGACTCCTTGGATGCCCCAAAATACTATGAGTGTCGGAA

076_20180714_03_03_trnT(GUU)-comp-psbD-comp CATAGAGATGAAATTGGAACAAGTTGACCCCCTTTTCTATTTATTTTCATTTCTTTGGACTCCGCAAGAATTTGCCGATATTTCCAATTCAATCGTCTTGTCTTGTTCCTAGATGTTCTATAGGAAAAAATTGTCATTTCGTTCCTCTACGGAGAACCTTTTATTCTAAATTCTAAATCACAGGATCTAAATTCTAAATCACAGGATAAGAAAAAAATTCACTATCTTTCTTTGATTACAGGATCAAGATTCATTTANNNNTTATCTATACTATATAATATATTCNNNNNNTATAGATATTTCTATTCTATATTTAGATTTATAGCTATCAGATCGTGGCTTGATGTACCAAAAATTTCCATTTCGTTGCATCCAATATTTTTGTTCCGACCATCGTATGAAGAAAGCATGCAAGATAAATACTATCATTTCCAATCTCCTNNNNNNATTTTAATATTGTANNNNNNTTGAAGTGAAGTAAAAATTTGGAAACCCTCTCTTTTCTAACAGGGAAAAATAAATCAAAAAATATTAGTAATTTAGTCTACATAAAAATTAGAATATAAAGAGAGTTCTTTTTCTTAATCTCATGAACAAGATCTAAGAATCCATTTAGTTGATGAAAGAACATGGGGGGGGGCAGGCCTGAGGATCAACCGGTAGTGGGTNNNNGGAAGGGGATTGCTTTTTCCTTGACCAATTCTTTCAAAAAACGAATCGGATTCATGGGTCATAATAAGATAATTCATGATTCAGATGCTTAATAATAAGATAATAAGAAGGAATAATCAAATTGAATTCATGAATTTACCCGGCAATTTATGGTCCAATAAAGGATTTTTATCTTCGAAACCCATTGGAAGGGGTAGTGCACGAGAAAAAAAATCATGCGGAAATGATCGACTCCTTGGATGCCCCAAAATACTATGAGTGTCGGAA

077_20180715_01_01_trnT(GUU)-comp-psbD-comp CATAGAGATGAAATTGGAACAAGTTGACCCCCTTTTCTATTTATTTTCATTTCTTTGGACTCCGCAAGAATTTGCCGATATTTCCAATTCAATCGTCTTGTCTTGTTCCTAGATGTTCTATAGGAAAAAATTGTCATTTCGTTCCTCTACGGAGAACCTTTTATNNNNNNNNNNNNNNNNNNNNNTCTAAATTCTAAATCACAGGATAAGAAAAAAATTCACTATCTTTCTTTGATTACAGGATCAAGATTCATTTANNNNTTATCTATACTATATAATATATTCNNNNNNTATAGATATTTCTATTCTATATTTAGATTTATAGCTATCAGATCGTGGCTTGATGTACCAAAAATTTCCATTTCGTTGCATCCAATATTTTTGTTCCGACCATCGTATGAAGAAAGCATGCAAGATAAATACTATCATTTCCAATCTCCTATTTTAATTTTAATATTGTANNNNNNTTGAAGTGAAGTAAAAATTTGGAAACCCTCTCTTTTCTAACAGGGAAAAATAAATCAAAAAATATTAGTAATTTAGTCTACATAAAAATTAGAATATAAAGAGAGTTCTTTTTCTTAATCTCATGAACAAGATCTAAGAATCCATTTAGTTGATGAAAGAACATGGGGGGGGGCAGGCCTGAGGATCAACCGGTAGTGGGTNNNNGGAAGGGGATTGCTTTTTCCTTGACCAATTCTTTCAAAAAACGAATCGGATTCATGGGTCATAATAAGATAATTCATGATTCAGATGCTTAATAATAAGATAATAAGAAGGAATAATCAAATTGAATTCATGAATTTACCCGGCAATTTATGGTCCAATAAAGGATTTTTATCTTCGAAACCCATTGGAAGGGGTAGTGCACGAGAAAAAAAATCATGCGGAAATGATCGACTCCTTGGATGCCCCAAAATACTATGAGTGTCGGAA

078_20180715_01_02_trnT(GUU)-comp-psbD-comp CATAGAGATGAAATTGGAACAAGTTGACCCCCTTTTCTATTTATTTTCATTTCTTTGGACTCCGCAAGAATTTGCCGATATTTCCAATTCAATCGTCTTGTCTTGTTCCTAGATGTTCTATAGGAAAAAATTGTCATTTCGTTCCTCTACGGAGAACCTTTTATNNNNNNNNNNNNNNNNNNNNNTCTAAATTCTAAATCACAGGATAAGAAAAAAATTCACTATCTTTCTTTGATTACAGGATCAAGATTCATTTANNNNTTATCTATACTATATAATATATTCNNNNNNTATAGATATTTCTATTCTATATTTAGATTTATAGCTATCAGATCGTGGCTTGATGTACCAAAAATTTCCATTTCGTTGCATCCAATATTTTTGTTCCGACCATCGTATGAAGAAAGCATGCAAGATAAATACTATCATTTCCAATCTCCTATTTTAATTTTAATATTGTANNNNNNTTGAAGTGAAGTAAAAATTTGGAAACCCTCTCTTTTCTAACAGGGAAAAATAAATCAAAAAATATTAGTAATTTAGTCTACATAAAAATTAGAATATAAAGAGAGTTCTTTTTCTTAATCTCATGAACAAGATCTAAGAATCCATTTAGTTGATGAAAGAACATGGGGGGGGGCAGGCCTGAGGATCAACCGGTAGTGGGTNNNNGGAAGGGGATTGCTTTTTCCTTGACCAATTCTTTCAAAAAACGAATCGGATTCATGGGTCATAATAAGATAATTCATGATTCAGATGCTTAATAATAAGATAATAAGAAGGAATAATCAAATTGAATTCATGAATTTACCCGGCAATTTATGGTCCAATAAAGGATTTTTATCTTCGAAACCCATTGGAAGGGGTAGTGCACGAGAAAAAAAATCATGCGGAAATGATCGACTCCTTGGATGCCCCAAAATACTATGAGTGTCGGAA

079_20180715_01_03_trnT(GUU)-comp-psbD-comp CATAGAGATGAAATTGGAACAAGTTGACCCCCTTTTCTATTTATTTTCATTTCTTTGGACTCCGCAAGAATTTGCCGATATTTCCAATTCAATCGTCTTGTCTTGTTCCTAGATGTTCTATAGGAAAAAATTGTCATTTCGTTCCTCTACGGAGAACCTTTTATNNNNNNNNNNNNNNNNNNNNNTCTAAATTCTAAATCACAGGATAAGAAAAAAATTCACTATCTTTCTTTGATTACAGGATCAAGATTCATTTANNNNTTATCTATACTATATAATATATTCNNNNNNTATAGATATTTCTATTCTATATTTAGATTTATAGCTATCAGATCGTGGCTTGATGTACCAAAAATTTCCATTTCGTTGCATCCAATATTTTTGTTCCGACCATCGTATGAAGAAAGCATGCAAGATAAATACTATCATTTCCAATCTCCTATTTTAATTTTAATATTGTANNNNNNTTGAAGTGAAGTAAAAATTTGGAAACCCTCTCTTTTCTAACAGGGAAAAATAAATCAAAAAATATTAGTAATTTAGTCTACATAAAAATTAGAATATAAAGAGAGTTCTTTTTCTTAATCTCATGAACAAGATCTAAGAATCCATTTAGTTGATGAAAGAACATGGGGGGGGGCAGGCCTGAGGATCAACCGGTAGTGGGTNNNNGGAAGGGGATTGCTTTTTCCTTGACCAATTCTTTCAAAAAACGAATCGGATTCATGGGTCATAATAAGATAATTCATGATTCAGATGCTTAATAATAAGATAATAAGAAGGAATAATCAAATTGAATTCATGAATTTACCCGGCAATTTATGGTCCAATAAAGGATTTTTATCTTCGAAACCCATTGGAAGGGGTAGTGCACGAGAAAAAAAATCATGCGGAAATGATCGACTCCTTGGATGCCCCAAAATACTATGAGTGTCGGAA

080_20180715_01_04_trnT(GUU)-comp-psbD-comp CATAGAGATGAAATTGGAACAAGTTGACCCCCTTTTCTATTTATTTTCATTTCTTTGGACTCCGCAAGAATTTGCCGATATTTCCAATTCAATCGTCTTGTCTTGTTCCTAGATGTTCTATAGGAAAAAATTGTCATTTCGTTCCTCTACGGAGAACCTTTTATNNNNNNNNNNNNNNNNNNNNNTCTAAATTCTAAATCACAGGATAAGAAAAAAATTCACTATCTTTCTTTGATTACAGGATCAAGATTCATTTANNNNTTATCTATACTATATAATATATTCNNNNNNTATAGATATTTCTATTCTATATTTAGATTTATAGCTATCAGATCGTGGCTTGATGTACCAAAAATTTCCATTTCGTTGCATCCAATATTTTTGTTCCGACCATCGTATGAAGAAAGCATGCAAGATAAATACTATCATTTCCAATCTCCTATTTTAATTTTAATATTGTANNNNNNTTGAAGTGAAGTAAAAATTTGGAAACCCTCTCTTTTCTAACAGGGAAAAATAAATCAAAAAATATTAGTAATTTAGTCTACATAAAAATTAGAATATAAAGAGAGTTCTTTTTCTTAATCTCATGAACAAGATCTAAGAATCCATTTAGTTGATGAAAGAACATGGGGGGGGGCAGGCCTGAGGATCAACCGGTAGTGGGTNNNNGGAAGGGGATTGCTTTTTCCTTGACCAATTCTTTCAAAAAACGAATCGGATTCATGGGTCATAATAAGATAATTCATGATTCAGATGCTTAATAATAAGATAATAAGAAGGAATAATCAAATTGAATTCATGAATTTACCCGGCAATTTATGGTCCAATAAAGGATTTTTATCTTCGAAACCCATTGGAAGGGGTAGTGCACGAGAAAAAAAATCATGCGGAAATGATCGACTCCTTGGATGCCCCAAAATACTATGAGTGTCGGAA

081_20180715_01_05_trnT(GUU)-comp-psbD-comp CATAGAGATGAAATTGGAACAAGTTGACCCCCTTTTCTATTTATTTTCATTTCTTTGGACTCCGCAAGAATTTGCCGATATTTCCAATTCAATCGTCTTGTCTTGTTCCTAGATGTTCTATAGGAAAAAATTGTCATTTCGTTCCTCTACGGAGAACCTTTTATNNNNNNNNNNNNNNNNNNNNNTCTAAATTCTAAATCACAGGATAAGAAAAAAATTCACTATCTTTCTTTGATTACAGGATCAAGATTCATTTANNNNTTATCTATACTATATAATATATTCNNNNNNTATAGATATTTCTATTCTATATTTAGATTTATAGCTATCAGATCGTGGCTTGATGTACCAAAAATTTCCATTTCGTTGCATCCAATATTTTTGTTCCGACCATCGTATGAAGAAAGCATGCAAGATAAATACTATCATTTCCAATCTCCTATTTTAATTTTAATATTGTANNNNNNTTGAAGTGAAGTAAAAATTTGGAAACCCTCTCTTTTCTAACAGGGAAAAATAAATCAAAAAATATTAGTAATTTAGTCTACATAAAAATTAGAATATAAAGAGAGTTCTTTTTCTTAATCTCATGAACAAGATCTAAGAATCCATTTAGTTGATGAAAGAACATGGGGGGGGGCAGGCCTGAGGATCAACCGGTAGTGGGTNNNNGGAAGGGGATTGCTTTTTCCTTGACCAATTCTTTCAAAAAACGAATCGGATTCATGGGTCATAATAAGATAATTCATGATTCAGATGCTTAATAATAAGATAATAAGAAGGAATAATCAAATTGAATTCATGAATTTACCCGGCAATTTATGGTCCAATAAAGGATTTTTATCTTCGAAACCCATTGGAAGGGGTAGTGCACGAGAAAAAAAATCATGCGGAAATGATCGACTCCTTGGATGCCCCAAAATACTATGAGTGTCGGAA

082_20180716_01_01_trnT(GUU)-comp-psbD-comp CATAGAGATGAAATTGGAACAAGTTGACCCCCTTTTCTATTTATTTTCATTTCTTTGGACTCCGCAAGAATTTGCCGATATTTCCAATTCAATCGTCTTGTCTTGTTCCTAGATGTTCTATAGGAAAAAATTGTCATTTCGTTCCTCTACGGAGAACCTTTTATTCTAAATTCTAAATCACAGGATCTAAATTCTAAATCACAGGATAAGAAAAAAATTCACTATCTTTCTTTGATTACAGGATCAAGATTCATTTANNNNTTATCTATACTATATAATATATTCNNNNNNTATAGATATTTCTATTCTATATTTAGATTTATAGCTATCAGATCGTGGCTTGATGTACCAAAAATTTCCATTTCGTTGCATCCAATATTTTTGTTCCGACCATCGTATGAAGAAAGCATGCAAGATAAATACTATCATTTCCAATCTCCTATTTTAATTTTAATATTGTANNNNNNTTGAAGTGAAGTAAAAATTTGGAAACCCTCTCTTTTCTAACAGGGAAAAATAAATCAAAAAATATTAGTAATTTAGTCTACATAAAAATTAGAATATAAAGAGAGTTCTTTTTCTTAATCTCATGAACAAGATCTAAGAATCCATTTAGTTGATGAAAGAACATGGGGGGGGGCAGGCCTGAGGATCAACCGGTAGTGGGTNNNNGGAAGGGGATTGCTTTTTCCTTGACCAATTCTTTCAAAAAACGAATCGGATTCATGGGTCATAATAAGATAATTCATGATTCAGATGCTTAATAATAAGATAATAAGAAGGAATAATCAAATTGAATTCATGAATTTACCCGGCAATTTATGGTCCAATAAAGGATTTTTATCTTCGAAACCCATTGGAAGGGGTAGTGCACGAGAAAAAAAATCATGCGGAAATGATCGACTCCTTGGATGCCCCAAAATACTATGAGTGTCGGAA

083_20180716_02_01_trnT(GUU)-comp-psbD-comp CATAGAGATGAAATTGGAACAAGTTGACCCCCTTTTCTATTTATTTTCATTTCTTTGGACTCCGCAAGAATTTGCCGATATTTCCAATTCAATCGTCTTGTCTTGTTCCTAGATGTTCTATAGGAAAAAATTGTCATTTCGTTCCTCTACGGAGAACCTTTTATTCTAAATTCTAAATCACAGGATCTAAATTCTAAATCACAGGATAAGAAAAAAATTCACTATCTTTCTTTGATTACAGGATCAAGATTCATTTANNNNTTATCTATACTATATAATATATTCNNNNNNTATAGATATTTCTATTCTATATTTAGATTTATAGCTATCAGATCGTGGCTTGATGTACCAAAAATTTCCATTTCGTTGCATCCAATATTTTTGTTCCGACCATCGTATGAAGAAAGCATGCAAGATAAATACTATCATTTCCAATCTCCTATTTTAATTTTAATATTGTANNNNNNTTGAAGTGAAGTAAAAATTTGGAAACCCTCTCTTTTCTAACAGGGAAAAATAAATCAAAAAATATTAGTAATTTAGTCTACATAAAAATTAGAATATAAAGAGAGTTCTTTTTCTTAATCTCATGAACAAGATCTAAGAATCCATTTAGTTGATGAAAGAACATGGGGGGGGGCAGGCCTGAGGATCAACCGGTAGTGGGTNNNNGGAAGGGGATTGCTTTTTCCTTGACCAATTCTTTCAAAAAACGAATCGGATTCATGGGTCATAATAAGATAATTCATGATTCAGATGCTTAATAATAAGATAATAAGAAGGAATAATCAAATTGAATTCATGAATTTACCCGGCAATTTATGGTCCAATAAAGGATTTTTATCTTCGAAACCCATTGGAAGGGGTAGTGCACGAGAAAAAAAATCATGCGGAAATGATCGACTCCTTGGATGCCCCAAAATACTATGAGTGTCGGAA

084_20180716_02_02_trnT(GUU)-comp-psbD-comp CATAGAGATGAAATTGGAACAAGTTGACCCCCTTTTCTATTTATTTTCATTTCTTTGGACTCCGCAAGAATTTGCCGATATTTCCAATTCAATCGTCTTGTCTTGTTCCTAGATGTTCTATAGGAAAAAATTGTCATTTCGTTCCTCTACGGAGAACCTTTTATTCTAAATTCTAAATCACAGGATCTAAATTCTAAATCACAGGATAAGAAAAAAATTCACTATCTTTCTTTGATTACAGGATCAAGATTCATTTANNNNTTATCTATACTATATAATATATTCNNNNNNTATAGATATTTCTATTCTATATTTAGATTTATAGCTATCAGATCGTGGCTTGATGTACCAAAAATTTCCATTTCGTTGCATCCAATATTTTTGTTCCGACCATCGTATGAAGAAAGCATGCAAGATAAATACTATCATTTCCAATCTCCTATTTTAATTTTAATATTGTANNNNNNTTGAAGTGAAGTAAAAATTTGGAAACCCTCTCTTTTCTAACAGGGAAAAATAAATCAAAAAATATTAGTAATTTAGTCTACATAAAAATTAGAATATAAAGAGAGTTCTTTTTCTTAATCTCATGAACAAGATCTAAGAATCCATTTAGTTGATGAAAGAACATGGGGGGGGGCAGGCCTGAGGATCAACCGGTAGTGGGTNNNNGGAAGGGGATTGCTTTTTCCTTGACCAATTCTTTCAAAAAACGAATCGGATTCATGGGTCATAATAAGATAATTCATGATTCAGATGCTTAATAATAAGATAATAAGAAGGAATAATCAAATTGAATTCATGAATTTACCCGGCAATTTATGGTCCAATAAAGGATTTTTATCTTCGAAACCCATTGGAAGGGGTAGTGCACGAGAAAAAAAATCATGCGGAAATGATCGACTCCTTGGATGCCCCAAAATACTATGAGTGTCGGAA

085_20180716_02_03_trnT(GUU)-comp-psbD-comp CATAGAGATGAAATTGGAACAAGTTGACCCCCTTTTCTATTTATTTTCATTTCTTTGGACTCCGCAAGAATTTGCCGATATTTCCAATTCAATCGTCTTGTCTTGTTCCTAGATGTTCTATAGGAAAAAATTGTCATTTCGTTCCTCTACGGAGAACCTTTTATTCTAAATTCTAAATCACAGGATCTAAATTCTAAATCACAGGATAAGAAAAAAATTCACTATCTTTCTTTGATTACAGGATCAAGATTCATTTANNNNTTATCTATACTATATAATATATTCNNNNNNTATAGATATTTCTATTCTATATTTAGATTTATAGCTATCAGATCGTGGCTTGATGTACCAAAAATTTCCATTTCGTTGCATCCAATATTTTTGTTCCGACCATCGTATGAAGAAAGCATGCAAGATAAATACTATCATTTCCAATCTCCTATTTTAATTTTAATATTGTANNNNNNTTGAAGTGAAGTAAAAATTTGGAAACCCTCTCTTTTCTAACAGGGAAAAATAAATCAAAAAATATTAGTAATTTAGTCTACATAAAAATTAGAATATAAAGAGAGTTCTTTTTCTTAATCTCATGAACAAGATCTAAGAATCCATTTAGTTGATGAAAGAACATGGGGGGGGGCAGGCCTGAGGATCAACCGGTAGTGGGTNNNNGGAAGGGGATTGCTTTTTCCTTGACCAATTCTTTCAAAAAACGAATCGGATTCATGGGTCATAATAAGATAATTCATGATTCAGATGCTTAATAATAAGATAATAAGAAGGAATAATCAAATTGAATTCATGAATTTACCCGGCAATTTATGGTCCAATAAAGGATTTTTATCTTCGAAACCCATTGGAAGGGGTAGTGCACGAGAAAAAAAATCATGCGGAAATGATCGACTCCTTGGATGCCCCAAAATACTATGAGTGTCGGAA

086_20180716_02_04_trnT(GUU)-comp-psbD-comp CATAGAGATGAAATTGGAACAAGTTGACCCCCTTTTCTATTTATTTTCATTTCTTTGGACTCCGCAAGAATTTGCCGATATTTCCAATTCAATCGTCTTGTCTTGTTCCTAGATGTTCTATAGGAAAAAATTGTCATTTCGTTCCTCTACGGAGAACCTTTTATTCTAAATTCTAAATCACAGGATCTAAATTCTAAATCACAGGATAAGAAAAAAATTCACTATCTTTCTTTGATTACAGGATCAAGATTCATTTANNNNTTATCTATACTATATAATATATTCNNNNNNTATAGATATTTCTATTCTATATTTAGATTTATAGCTATCAGATCGTGGCTTGATGTACCAAAAATTTCCATTTCGTTGCATCCAATATTTTTGTTCCGACCATCGTATGAAGAAAGCATGCAAGATAAATACTATCATTTCCAATCTCCTATTTTAATTTTAATATTGTANNNNNNTTGAAGTGAAGTAAAAATTTGGAAACCCTCTCTTTTCTAACAGGGAAAAATAAATCAAAAAATATTAGTAATTTAGTCTACATAAAAATTAGAATATAAAGAGAGTTCTTTTTCTTAATCTCATGAACAAGATCTAAGAATCCATTTAGTTGATGAAAGAACATGGGGGGGGGCAGGCCTGAGGATCAACCGGTAGTGGGTNNNNGGAAGGGGATTGCTTTTTCCTTGACCAATTCTTTCAAAAAACGAATCGGATTCATGGGTCATAATAAGATAATTCATGATTCAGATGCTTAATAATAAGATAATAAGAAGGAATAATCAAATTGAATTCATGAATTTACCCGGCAATTTATGGTCCAATAAAGGATTTTTATCTTCGAAACCCATTGGAAGGGGTAGTGCACGAGAAAAAAAATCATGCGGAAATGATCGACTCCTTGGATGCCCCAAAATACTATGAGTGTCGGAA

087_20180717_01_01_trnT(GUU)-comp-psbD-comp CATAGAGATGAAATTGGAACAAGTTGACCCCCTTTTCTATTTATTTTCATTTCTTTGGACTCCGCAAGAATTTGCCGATATTTCCAATTCAATCGTCTTGTCTTGTTCCTAGATGTTCTATAGGAAAAAATTGTCATTTCGTTCCTCTACGGAGAACCTTTTATTCTAAATTCTAAATCACAGGATCTAAATTCTAAATCACAGGATAAGAAAAAAATTCACTATCTTTCTTTGATTACAGGATCAAGATTCATTTANNNNTTATCTATACTATATAATATATTCNNNNNNTATAGATATTTCTATTCTATATTTAGATTTATAGCTATCAGATCGTGGCTTGATGTACCAAAAATTTCCATTTCGTTGCATCCAATATTTTTGTTCCGACCATCGTATGAAGAAAGCATGCAAGATAAATACTATCATTTCCAATCTCCTATTTTAATTTTAATATTGTANNNNNNTTGAAGTGAAGTAAAAATTTGGAAACCCTCTCTTTTCTAACAGGGAAAAATAAATCAAAAAATATTAGTAATTTAGTCTACATAAAAATTAGAATATAAAGAGAGTTCTTTTTCTTAATCTCATGAACAAGATCTAAGAATCCATTTAGTTGATGAAAGAACATGGGGGGGGGCAGGCCTGAGGATCAACCGGTAGTGGGTNNNNGGAAGGGGATTGCTTTTTCCTTGACCAATTCTTTCAAAAAACGAATCGGATTCATGGGTCATAATAAGATAATTCATGATTCAGATGCTTAATAATAAGATAATAAGAAGGAATAATCAAATTGAATTCATGAATTTACCCGGCAATTTATGGTCCAATAAAGGATTTTTATCTTCGAAACCCATTGGAAGGGGTAGTGCACGAGAAAAAAAATCATGCGGAAATGATCGACTCCTTGGATGCCCCAAAATACTATGAGTGTCGGAA

088_20180717_01_02_trnT(GUU)-comp-psbD-comp CATAGAGATGAAATTGGAACAAGTTGACCCCCTTTTCTATTTATTTTCATTTCTTTGGACTCCGCAAGAATTTGCCGATATTTCCAATTCAATCGTCTTGTCTTGTTCCTAGATGTTCTATAGGAAAAAATTGTCATTTCGTTCCTCTACGGAGAACCTTTTATTCTAAATTCTAAATCACAGGATCTAAATTCTAAATCACAGGATAAGAAAAAAATTCACTATCTTTCTTTGATTACAGGATCAAGATTCATTTANNNNTTATCTATACTATATAATATATTCNNNNNNTATAGATATTTCTATTCTATATTTAGATTTATAGCTATCAGATCGTGGCTTGATGTACCAAAAATTTCCATTTCGTTGCATCCAATATTTTTGTTCCGACCATCGTATGAAGAAAGCATGCAAGATAAATACTATCATTTCCAATCTCCTATTTTAATTTTAATATTGTANNNNNNTTGAAGTGAAGTAAAAATTTGGAAACCCTCTCTTTTCTAACAGGGAAAAATAAATCAAAAAATATTAGTAATTTAGTCTACATAAAAATTAGAATATAAAGAGAGTTCTTTTTCTTAATCTCATGAACAAGATCTAAGAATCCATTTAGTTGATGAAAGAACATGGGGGGGGGCAGGCCTGAGGATCAACCGGTAGTGGGTNNNNGGAAGGGGATTGCTTTTTCCTTGACCAATTCTTTCAAAAAACGAATCGGATTCATGGGTCATAATAAGATAATTCATGATTCAGATGCTTAATAATAAGATAATAAGAAGGAATAATCAAATTGAATTCATGAATTTACCCGGCAATTTATGGTCCAATAAAGGATTTTTATCTTCGAAACCCATTGGAAGGGGTAGTGCACGAGAAAAAAAATCATGCGGAAATGATCGACTCCTTGGATGCCCCAAAATACTATGAGTGTCGGAA

089_20180717_01_03_trnT(GUU)-comp-psbD-comp CATAGAGATGAAATTGGAACAAGTTGACCCCCTTTTCTATTTATTTTCATTTCTTTGGACTCCGCAAGAATTTGCCGATATTTCCAATTCAATCGTCTTGTCTTGTTCCTAGATGTTCTATAGGAAAAAATTGTCATTTCGTTCCTCTACGGAGAACCTTTTATTCTAAATTCTAAATCACAGGATCTAAATTCTAAATCACAGGATAAGAAAAAAATTCACTATCTTTCTTTGATTACAGGATCAAGATTCATTTANNNNTTATCTATACTATATAATATATTCNNNNNNTATAGATATTTCTATTCTATATTTAGATTTATAGCTATCAGATCGTGGCTTGATGTACCAAAAATTTCCATTTCGTTGCATCCAATATTTTTGTTCCGACCATCGTATGAAGAAAGCATGCAAGATAAATACTATCATTTCCAATCTCCTATTTTAATTTTAATATTGTANNNNNNTTGAAGTGAAGTAAAAATTTGGAAACCCTCTCTTTTCTAACAGGGAAAAATAAATCAAAAAATATTAGTAATTTAGTCTACATAAAAATTAGAATATAAAGAGAGTTCTTTTTCTTAATCTCATGAACAAGATCTAAGAATCCATTTAGTTGATGAAAGAACATGGGGGGGGGCAGGCCTGAGGATCAACCGGTAGTGGGTNNNNGGAAGGGGATTGCTTTTTCCTTGACCAATTCTTTCAAAAAACGAATCGGATTCATGGGTCATAATAAGATAATTCATGATTCAGATGCTTAATAATAAGATAATAAGAAGGAATAATCAAATTGAATTCATGAATTTACCCGGCAATTTATGGTCCAATAAAGGATTTTTATCTTCGAAACCCATTGGAAGGGGTAGTGCACGAGAAAAAAAATCATGCGGAAATGATCGACTCCTTGGATGCCCCAAAATACTATGAGTGTCGGAA

090_20180717_01_04_trnT(GUU)-comp-psbD-comp CATAGAGATGAAATTGGAACAAGTTGACCCCCTTTTCTATTTATTTTCATTTCTTTGGACTCCGCAAGAATTTGCCGATATTTCCAATTCAATCGTCTTGTCTTGTTCCTAGATGTTCTATAGGAAAAAATTGTCATTTCGTTCCTCTACGGAGAACCTTTTATTCTAAATTCTAAATCACAGGATCTAAATTCTAAATCACAGGATAAGAAAAAAATTCACTATCTTTCTTTGATTACAGGATCAAGATTCATTTANNNNTTATCTATACTATATAATATATTCNNNNNNTATAGATATTTCTATTCTATATTTAGATTTATAGCTATCAGATCGTGGCTTGATGTACCAAAAATTTCCATTTCGTTGCATCCAATATTTTTGTTCCGACCATCGTATGAAGAAAGCATGCAAGATAAATACTATCATTTCCAATCTCCTATTTTAATTTTAATATTGTANNNNNNTTGAAGTGAAGTAAAAATTTGGAAACCCTCTCTTTTCTAACAGGGAAAAATAAATCAAAAAATATTAGTAATTTAGTCTACATAAAAATTAGAATATAAAGAGAGTTCTTTTTCTTAATCTCATGAACAAGATCTAAGAATCCATTTAGTTGATGAAAGAACATGGGGGGGGGCAGGCCTGAGGATCAACCGGTAGTGGGTNNNNGGAAGGGGATTGCTTTTTCCTTGACCAATTCTTTCAAAAAACGAATCGGATTCATGGGTCATAATAAGATAATTCATGATTCAGATGCTTAATAATAAGATAATAAGAAGGAATAATCAAATTGAATTCATGAATTTACCCGGCAATTTATGGTCCAATAAAGGATTTTTATCTTCGAAACCCATTGGAAGGGGTAGTGCACGAGAAAAAAAATCATGCGGAAATGATCGACTCCTTGGATGCCCCAAAATACTATGAGTGTCGGAA

091_20180717_01_05_trnT(GUU)-comp-psbD-comp CATAGAGATGAAATTGGAACAAGTTGACCCCCTTTTCTATTTATTTTCATTTCTTTGGACTCCGCAAGAATTTGCCGATATTTCCAATTCAATCGTCTTGTCTTGTTCCTAGATGTTCTATAGGAAAAAATTGTCATTTCGTTCCTCTACGGAGAACCTTTTATTCTAAATTCTAAATCACAGGATCTAAATTCTAAATCACAGGATAAGAAAAAAATTCACTATCTTTCTTTGATTACAGGATCAAGATTCATTTANNNNTTATCTATACTATATAATATATTCNNNNNNTATAGATATTTCTATTCTATATTTAGATTTATAGCTATCAGATCGTGGCTTGATGTACCAAAAATTTCCATTTCGTTGCATCCAATATTTTTGTTCCGACCATCGTATGAAGAAAGCATGCAAGATAAATACTATCATTTCCAATCTCCTATTTTAATTTTAATATTGTANNNNNNTTGAAGTGAAGTAAAAATTTGGAAACCCTCTCTTTTCTAACAGGGAAAAATAAATCAAAAAATATTAGTAATTTAGTCTACATAAAAATTAGAATATAAAGAGAGTTCTTTTTCTTAATCTCATGAACAAGATCTAAGAATCCATTTAGTTGATGAAAGAACATGGGGGGGGGCAGGCCTGAGGATCAACCGGTAGTGGGTNNNNGGAAGGGGATTGCTTTTTCCTTGACCAATTCTTTCAAAAAACGAATCGGATTCATGGGTCATAATAAGATAATTCATGATTCAGATGCTTAATAATAAGATAATAAGAAGGAATAATCAAATTGAATTCATGAATTTACCCGGCAATTTATGGTCCAATAAAGGATTTTTATCTTCGAAACCCATTGGAAGGGGTAGTGCACGAGAAAAAAAATCATGCGGAAATGATCGACTCCTTGGATGCCCCAAAATACTATGAGTGTCGGAA

092_20180717_03_01_trnT(GUU)-comp-psbD-comp CATAGAGATGAAATTGGAACAAGTTGACCCCCTTTTCTATTTATTTTCATTTCTTTGGACTCCGCAAGAATTTGCCGATATTTCCAATTCAATCGTCTTGTCTTGTTCCTAGATGTTCTATAGGAAAAAATTGTCATTTCGTTCCTCTACGGAGAACCTTTTATTCTAAATTCTAAATCACAGGATCTAAATTCTAAATCACAGGATAAGAAAAAAATTCACTATCTTTCTTTGATTACAGGATCAAGATTCATTTANNNNTTATCTATACTATATAATATATTCNNNNNNTATAGATATTTCTATTCTATATTTAGATTTATAGCTATCAGATCGTGGCTTGATGTACCAAAAATTTCCATTTCGTTGCATCCAATATTTTTGTTCCGACCATCGTATGAAGAAAGCATGCAAGATAAATACTATCATTTCCAATCTCCTATTTTAATTTTAATATTGTANNNNNNTTGAAGTGAAGTAAAAATTTGGAAACCCTCTCTTTTCTAACAGGGAAAAATAAATCAAAAAATATTAGTAATTTAGTCTACATAAAAATTAGAATATAAAGAGAGTTCTTTTTCTTAATCTCATGAACAAGATCTAAGAATCCATTTAGTTGATGAAAGAACATGGGGGGGGGCAGGCCTGAGGATCAACCGGTAGTGGGTNNNNGGAAGGGGATTGCTTTTTCCTTGACCAATTCTTTCAAAAAACGAATCGGATTCATGGGTCATAATAAGATAATTCATGATTCAGATGCTTAATAATAAGATAATAAGAAGGAATAATCAAATTGAATTCATGAATTTACCCGGCAATTTATGGTCCAATAAAGGATTTTTATCTTCGAAACCCATTGGAAGGGGTAGTGCACGAGAAAAAAAATCATGCGGAAATGATCGACTCCTTGGATGCCCCAAAATACTATGAGTGTCGGAA

093_20180717_03_02_trnT(GUU)-comp-psbD-comp CATAGAGATGAAATTGGAACAAGTTGACCCCCTTTTCTATTTATTTTCATTTCTTTGGACTCCGCAAGAATTTGCCGATATTTCCAATTCAATCGTCTTGTCTTGTTCCTAGATGTTCTATAGGAAAAAATTGTCATTTCGTTCCTCTACGGAGAACCTTTTATTCTAAATTCTAAATCACAGGATCTAAATTCTAAATCACAGGATAAGAAAAAAATTCACTATCTTTCTTTGATTACAGGATCAAGATTCATTTANNNNTTATCTATACTATATAATATATTCNNNNNNTATAGATATTTCTATTCTATATTTAGATTTATAGCTATCAGATCGTGGCTTGATGTACCAAAAATTTCCATTTCGTTGCATCCAATATTTTTGTTCCGACCATCGTATGAAGAAAGCATGCAAGATAAATACTATCATTTCCAATCTCCTATTTTAATTTTAATATTGTANNNNNNTTGAAGTGAAGTAAAAATTTGGAAACCCTCTCTTTTCTAACAGGGAAAAATAAATCAAAAAATATTAGTAATTTAGTCTACATAAAAATTAGAATATAAAGAGAGTTCTTTTTCTTAATCTCATGAACAAGATCTAAGAATCCATTTAGTTGATGAAAGAACATGGGGGGGGGCAGGCCTGAGGATCAACCGGTAGTGGGTNNNNGGAAGGGGATTGCTTTTTCCTTGACCAATTCTTTCAAAAAACGAATCGGATTCATGGGTCATAATAAGATAATTCATGATTCAGATGCTTAATAATAAGATAATAAGAAGGAATAATCAAATTGAATTCATGAATTTACCCGGCAATTTATGGTCCAATAAAGGATTTTTATCTTCGAAACCCATTGGAAGGGGTAGTGCACGAGAAAAAAAATCATGCGGAAATGATCGACTCCTTGGATGCCCCAAAATACTATGAGTGTCGGAA

094_20180717_03_03_trnT(GUU)-comp-psbD-comp CATAGAGATGAAATTGGAACAAGTTGACCCCCTTTTCTATTTATTTTCATTTCTTTGGACTCCGCAAGAATTTGCCGATATTTCCAATTCAATCGTCTTGTCTTGTTCCTAGATGTTCTATAGGAAAAAATTGTCATTTCGTTCCTCTACGGAGAACCTTTTATNNNNNNNNNNNNNNNNNNNNNTCTAAATTCTAAATCACAGGATAAGAAAAAAATTCACTATCTTTCTTTGATTACAGGATCAAGATTCATTTANNNNTTATCTATACTATATAATATATTCNNNNNNTATAGATATTTCTATTCTATATTTAGATTTATAGCTATCAGATCGTGGCTTGATGTACCAAAAATTTCCATTTCGTTGCATCCAATATTTTTGTTCCGACCATCGTATGAAGAAAGCATGCAAGATAAATACTATCATTTCCAATCTCCTATTTTAATTTTAATATTGTANNNNNNTTGAAGTGAAGTAAAAATTTGGAAACCCTCTCTTTTCTAACAGGGAAAAATAAATCAAAAAATATTAGTAATTTAGTCTACATAAAAATTAGAATATAAAGAGAGTTCTTTTTCTTAATCTCATGAACAAGATCTAAGAATCCATTTAGTTGATGAAAGAACATGGGGGGGGGCAGGCCTGAGGATCAACCGGTAGTGGGTNNNNGGAAGGGGATTGCTTTTTCCTTGACCAATTCTTTCAAAAAACGAATCGGATTCATGGGTCATAATAAGATAATTCATGATTCAGATGCTTAATAATAAGATAATAAGAAGGAATAATCAAATTGAATTCATGAATTTACCCGGCAATTTATGGTCCAATAAAGGATTTTTATCTTCGAAACCCATTGGAAGGGGTAGTGCACGAGAAAAAAAATCATGCGGAAATGATCGACTCCTTGGATGCCCCAAAATACTATGAGTGTCGGAA

095_20180717_03_04_trnT(GUU)-comp-psbD-comp CATAGAGATGAAATTGGAACAAGTTGACCCCCTTTTCTATTTATTTTCATTTCTTTGGACTCCGCAAGAATTTGCCGATATTTCCAATTCAATCGTCTTGTCTTGTTCCTAGATGTTCTATAGGAAAAAATTGTCATTTCGTTCCTCTACGGAGAACCTTTTATTCTAAATTCTAAATCACAGGATCTAAATTCTAAATCACAGGATAAGAAAAAAATTCACTATCTTTCTTTGATTACAGGATCAAGATTCATTTANNNNTTATCTATACTATATAATATATTCNNNNNNTATAGATATTTCTATTCTATATTTAGATTTATAGCTATCAGATCGTGGCTTGATGTACCAAAAATTTCCATTTCGTTGCATCCAATATTTTTGTTCCGACCATCGTATGAAGAAAGCATGCAAGATAAATACTATCATTTCCAATCTCCTATTTTAATTTTAATATTGTANNNNNNTTGAAGTGAAGTAAAAATTTGGAAACCCTCTCTTTTCTAACAGGGAAAAATAAATCAAAAAATATTAGTAATTTAGTCTACATAAAAATTAGAATATAAAGAGAGTTCTTTTTCTTAATCTCATGAACAAGATCTAAGAATCCATTTAGTTGATGAAAGAACATGGGGGGGGGCAGGCCTGAGGATCAACCGGTAGTGGGTNNNNGGAAGGGGATTGCTTTTTCCTTGACCAATTCTTTCAAAAAACGAATCGGATTCATGGGTCATAATAAGATAATTCATGATTCAGATGCTTAATATTACGATAATAAGAAGGAATAATCAAATTGAATTCATGAATTTACCCGGCAATTTATGGTCCAATAAAGGATTTTTATCTTCGAAACCCATTGGAAGGGGTAGTGCACGAGAAAAAAAATCATGCGGAAATGATCGACTCCTTGCATGCCCCAAAATACTATGAGTGTCGGAA

096_20180717_03_05_trnT(GUU)-comp-psbD-comp CATAGAGATGAAATTGGAACAAGTTGACCCCCTTTTCTATTTATTTTCATTTCTTTGGACTCCGCAAGAATTTGCCGATATTTCCAATTCAATCGTCTTGTCTTGTTCCTAGATGTTCTATAGGAAAAAATTGTCATTTCGTTCCTCTACGGAGAACCTTTTATTCTAAATTCTAAATCACAGGATCTAAATTCTAAATCACAGGATAAGAAAAAAATTCACTATCTTTCTTTGATTACAGGATCAAGATTCATTTANNNNTTATCTATACTATATAATATATTCNNNNNNTATAGATATTTCTATTCTATATTTAGATTTATAGCTATCAGATCGTGGCTTGATGTACCAAAAATTTCCATTTCGTTGCATCCAATATTTTTGTTCCGACCATCGTATGAAGAAAGCATGCAAGATAAATACTATCATTTCCAATCTCCTATTTTAATTTTAATATTGTANNNNNNTTGAAGTGAAGTAAAAATTTGGAAACCCTCTCTTTTCTAACAGGGAAAAATAAATCAAAAAATATTAGTAATTTAGTCTACATAAAAATTAGAATATAAAGAGAGTTCTTTTTCTTAATCTCATGAACAAGATCTAAGAATCCATTTAGTTGATGAAAGAACATGGGGGGGGGCAGGCCTGAGGATCAACCGGTAGTGGGTNNNNGGAAGGGGATTGCTTTTTCCTTGACCAATTCTTTCAAAAAACGAATCGGATTCATGGGTCATAATAAGATAATTCATGATTCAGATGCTTAATAATAAGATAATAAGAAGGAATAATCAAATTGAATTCATGAATTTACCCGGCAATTTATGGTCCAATAAAGGATTTTTATCTTCGAAACCCATTGGAAGGGGTAGTGCACGAGAAAAAAAATCATGCGGAAATGATCGACTCCTTGGATGCCCCAAAATACTATGAGTGTCGGAA

097_20180718_01_01_trnT(GUU)-comp-psbD-comp CATAGAGATGAAATTGGAACAAGTTGACCCCCTTTTCTATTTATTTTCATTTCTTTGGACTCCGCAAGAATTTGCCGATATTTCCAATTCAATCGTCTTGTCTTGTTCCTAGATGTTCTATAGGAAAAAATTGTCATTTCGTTCCTCTACGGAGAACCTTTTATTCTAAATTCTAAATCACAGGATCTAAATTCTAAATCACAGGATAAGAAAAAAATTCACTATCTTTCTTTGATTACAGGATCAAGATTCATTTANNNNTTATCTATACTATATAATATATTCNNNNNNTATAGATATTTCTATTCTATATTTATATTTATAGCTATCAGATCGTGGCTTGATGTACCAAAAATTTCCATTTCGTTGCATCCAATATTTTTGTTCCGACCATCGTATGAAGAAAGCATGCAAGATAAATACTATCATTTCCAATCTCCTATTTTAATTTTAATATTGTANNNNNNTTGAAGTGAAGTAAAAATTTGGAAACCCTCTCTTTTCTAACAGGGAAAAATAAATCAAAAAATATTAGTAATTTAGTCTACATAAAAATTAGAATATAAAGAGAGTTCTTTTTCTTAATCTCATGAACAAGATCTAAGAATCCATTTAGTTGATGAAAGAACATGGGGGGGGGCAGGCCTGAGGATCAACCGGTAGTGGGTNNNNGGAAGGGGATTGCTTTTTCCTTGACCAATTCTTTCAAAAAACGAATCGGATTCATGGGTCATAATAAGATAATTCATGATTCAGATGCTTAATAATAAGATAATAAGAAGGAATAATCAAATTGAATTCATGAATTTACCCGGCAATTTATGGTCCAATAAAGGATTTTTATCTTCGAAACCCATTGGAAGGGGTAGTGCACGAGAAAAAAAATCATGCGGAAATGATCGACTCCTTGGATGCCCCAAAATACTATGAGTGTCGGAA

098_20180718_01_02_trnT(GUU)-comp-psbD-comp CATAGAGATGAAATTGGAACAAGTTGACCCCCTTTTCTATTTATTTTCATTTCTTTGGACTCCGCAAGAATTTGCCGATATTTCCAATTCAATCGTCTTGTCTTGTTCCTAGATGTTCTATAGGAAAAAATTGTCATTTCGTTCCTCTACGGAGAACCTTTTATTCTAAATTCTAAATCACAGGATCTAAATTCTAAATCACAGGATAAGAAAAAAATTCACTATCTTTCTTTGATTACAGGATCAAGATTCATTTANNNNTTATCTATACTATATAATATATTCNNNNNNTATAGATATTTCTATTCTATATTTATATTTATAGCTATCAGATCGTGGCTTGATGTACCAAAAATTTCCATTTCGTTGCATCCAATATTTTTGTTCCGACCATCGTATGAAGAAAGCATGCAAGATAAATACTATCATTTCCAATCTCCTATTTTAATTTTAATATTGTANNNNNNTTGAAGTGAAGTAAAAATTTGGAAACCCTCTCTTTTCTAACAGGGAAAAATAAATCAAAAAATATTAGTAATTTAGTCTACATAAAAATTAGAATATAAAGAGAGTTCTTTTTCTTAATCTCATGAACAAGATCTAAGAATCCATTTAGTTGATGAAAGAACATGGGGGGGGGCAGGCCTGAGGATCAACCGGTAGTGGGTNNNNGGAAGGGGATTGCTTTTTCCTTGACCAATTCTTTCAAAAAACGAATCGGATTCATGGGTCATAATAAGATAATTCATGATTCAGATGCTTAATAATAAGATAATAAGAAGGAATAATCAAATTGAATTCATGAATTTACCCGGCAATTTATGGTCCAATAAAGGATTTTTATCTTCGAAACCCATTGGAAGGGGTAGTGCACGAGAAAAAAAATCATGCGGAAATGATCGACTCCTTGGATGCCCCAAAATACTATGAGTGTCGGAA

099_20180718_01_03_trnT(GUU)-comp-psbD-comp CATAGAGATGAAATTGGAACAAGTTGACCCCCTTTTCTATTTATTTTCATTTCTTTGGACTCCGCAAGAATTTGCCGATATTTCCAATTCAATCGTCTTGTCTTGTTCCTAGATGTTCTATAGGAAAAAATTGTCATTTCGTTCCTCTACGGAGAACCTTTTATTCTAAATTCTAAATCACAGGATCTAAATTCTAAATCACAGGATAAGAAAAAAATTCACTATCTTTCTTTGATTACAGGATCAAGATTCATTTANNNNTTATCTATACTATATAATATATTCNNNNNNTATAGATATTTCTATTCTATATTTATATTTATAGCTATCAGATCGTGGCTTGATGTACCAAAAATTTCCATTTCGTTGCATCCAATATTTTTGTTCCGACCATCGTATGAAGAAAGCATGCAAGATAAATACTATCATTTCCAATCTCCTATTTTAATTTTAATATTGTANNNNNNTTGAAGTGAAGTAAAAATTTGGAAACCCTCTCTTTTCTAACAGGGAAAAATAAATCAAAAAATATTAGTAATTTAGTCTACATAAAAATTAGAATATAAAGAGAGTTCTTTTTCTTAATCTCATGAACAAGATCTAAGAATCCATTTAGTTGATGAAAGAACATGGGGGGGGGCAGGCCTGAGGATCAACCGGTAGTGGGTNNNNGGAAGGGGATTGCTTTTTCCTTGACCAATTCTTTCAAAAAACGAATCGGATTCATGGGTCATAATAAGATAATTCATGATTCAGATGCTTAATAATAAGATAATAAGAAGGAATAATCAAATTGAATTCATGAATTTACCCGGCAATTTATGGTCCAATAAAGGATTTTTATCTTCGAAACCCATTGGAAGGGGTAGTGCACGAGAAAAAAAATCATGCGGAAATGATCGACTCCTTGGATGCCCCAAAATACTATGAGTGTCGGAA

100_20180718_01_04_trnT(GUU)-comp-psbD-comp CATAGAGATGAAATTGGAACAAGTTGACCCCCTTTTCTATTTATTTTCATTTCTTTGGACTCCGCAAGAATTTGCCGATATTTCCAATTCAATCGTCTTGTCTTGTTCCTAGATGTTCTATAGGAAAAAATTGTCATTTCGTTCCTCTACGGAGAACCTTTTATTCTAAATTCTAAATCACAGGATCTAAATTCTAAATCACAGGATAAGAAAAAAATTCACTATCTTTCTTTGATTACAGGATCAAGATTCATTTANNNNTTATCTATACTATATAATATATTCNNNNNNTATAGATATTTCTATTCTATATTTATATTTATAGCTATCAGATCGTGGCTTGATGTACCAAAAATTTCCATTTCGTTGCATCCAATATTTTTGTTCCGACCATCGTATGAAGAAAGCATGCAAGATAAATACTATCATTTCCAATCTCCTATTTTAATTTTAATATTGTANNNNNNTTGAAGTGAAGTAAAAATTTGGAAACCCTCTCTTTTCTAACAGGGAAAAATAAATCAAAAAATATTAGTAATTTAGTCTACATAAAAATTAGAATATAAAGAGAGTTCTTTTTCTTAATCTCATGAACAAGATCTAAGAATCCATTTAGTTGATGAAAGAACATGGGGGGGGGCAGGCCTGAGGATCAACCGGTAGTGGGTNNNNGGAAGGGGATTGCTTTTTCCTTGACCAATTCTTTCAAAAAACGAATCGGATTCATGGGTCATAATAAGATAATTCATGATTCAGATGCTTAATAATAAGATAATAAGAAGGAATAATCAAATTGAATTCATGAATTTACCCGGCAATTTATGGTCCAATAAAGGATTTTTATCTTCGAAACCCATTGGAAGGGGTAGTGCACGAGAAAAAAAATCATGCGGAAATGATCGACTCCTTGGATGCCCCAAAATACTATGAGTGTCGGAA

101_20180718_01_05_trnT(GUU)-comp-psbD-comp CATAGAGATGAAATTGGAACAAGTTGACCCCCTTTTCTATTTATTTTCATTTCTTTGGACTCCGCAAGAATTTGCCGATATTTCCAATTCAATCGTCTTGTCTTGTTCCTAGATGTTCTATAGGAAAAAATTGTCATTTCGTTCCTCTACGGAGAACCTTTTATTCTAAATTCTAAATCACAGGATCTAAATTCTAAATCACAGGATAAGAAAAAAATTCACTATCTTTCTTTGATTACAGGATCAAGATTCATTTANNNNTTATCTATACTATATAATATATTCNNNNNNTATAGATATTTCTATTCTATATTTATATTTATAGCTATCAGATCGTGGCTTGATGTACCAAAAATTTCCATTTCGTTGCATCCAATATTTTTGTTCCGACCATCGTATGAAGAAAGCATGCAAGATAAATACTATCATTTCCAATCTCCTATTTTAATTTTAATATTGTANNNNNNTTGAAGTGAAGTAAAAATTTGGAAACCCTCTCTTTTCTAACAGGGAAAAATAAATCAAAAAATATTAGTAATTTAGTCTACATAAAAATTAGAATATAAAGAGAGTTCTTTTTCTTAATCTCATGAACAAGATCTAAGAATCCATTTAGTTGATGAAAGAACATGGGGGGGGGCAGGCCTGAGGATCAACCGGTAGTGGGTNNNNGGAAGGGGATTGCTTTTTCCTTGACCAATTCTTTCAAAAAACGAATCGGATTCATGGGTCATAATAAGATAATTCATGATTCAGATGCTTAATAATAAGATAATAAGAAGGAATAATCAAATTGAATTCATGAATTTACCCGGCAATTTATGGTCCAATAAAGGATTTTTATCTTCGAAACCCATTGGAAGGGGTAGTGCACGAGAAAAAAAATCATGCGGAAATGATCGACTCCTTGGATGCCCCAAAATACTATGAGTGTCGGAA

102_20180718_03_02_trnT(GUU)-comp-psbD-comp CATAGAGATGAAATTGGAACAAGTTGACCCCCTTTTCTATTTATTTTCATTTCTTTGGACTCCGCAAGAATTTGCCGATATTTCCAATTCAATCGTCTTGTCTTGTTCCTAGATGTTCTATAGGAAAAAATTGTCATTTCGTTCCTCTACGGAGAACCTTTTATTCTAAATTCTAAATCACAGGATCTAAATTCTAAATCACAGGATAAGAAAAAAATTCACTATCTTTCTTTGATTACAGGATCAAGATTCATTTANNNNTTATCTATACTATATAATATATTCNNNNNNTATAGATATTTCTATTCTATATTTATATTTATAGCTATCAGATCGTGGCTTGATGTACCAAAAATTTCCATTTCGTTGCATCCAATATTTTTGTTCCGACCATCGTATGAAGAAAGCATGCAAGATAAATACTATCATTTCCAATCTCCTATTTTAATTTTAATATTGTANNNNNNTTGAAGTGAAGTAAAAATTTGGAAACCCTCTCTTTTCTAACAGGGAAAAATAAATCAAAAAATATTAGTAATTTAGTCTACATAAAAATTAGAATATAAAGAGAGTTCTTTTTCTTAATCTCATGAACAAGATCTAAGAATCCATTTAGTTGATGAAAGAACATGGGGGGGGGCAGGCCTGAGGATCAACCGGTAGTGGGTNNNNGGAAGGGGATTGCTTTTTCCTTGACCAATTCTTTCAAAAAACGAATCGGATTCATGGGTCATAATAAGATAATTCATGATTCAGATGCTTAATAATAAGATAATAAGAAGGAATAATCAAATTGAATTCATGAATTTACCCGGCAATTTATGGTCCAATAAAGGATTTTTATCTTCGAAACCCATTGGAAGGGGTAGTGCACGAGAAAAAAAATCATGCGGAAATGATCGACTCCTTGGATGCCCCAAAATACTATGAGTGTCGGAA

103_20180718_03_03_trnT(GUU)-comp-psbD-comp CATAGAGATGAAATTGGAACAAGTTGACCCCCTTTTCTATTTATTTTCATTTCTTTGGACTCCGCAAGAATTTGCCGATATTTCCAATTCAATCGTCTTGTCTTGTTCCTAGATGTTCTATAGGAAAAAATTGTCATTTCGTTCCTCTACGGAGAACCTTTTATTCTAAATTCTAAATCACAGGATCTAAATTCTAAATCACAGGATAAGAAAAAAATTCACTATCTTTCTTTGATTACAGGATCAAGATTCATTTANNNNTTATCTATACTATATAATATATTCNNNNNNTATAGATATTTCTATTCTATATTTATATTTATAGCTATCAGATCGTGGCTTGATGTACCAAAAATTTCCATTTCGTTGCATCCAATATTTTTGTTCCGACCATCGTATGAAGAAAGCATGCAAGATAAATACTATCATTTCCAATCTCCTATTTTAATTTTAATATTGTANNNNNNTTGAAGTGAAGTAAAAATTTGGAAACCCTCTCTTTTCTAACAGGGAAAAATAAATCAAAAAATATTAGTAATTTAGTCTACATAAAAATTAGAATATAAAGAGAGTTCTTTTTCTTAATCTCATGAACAAGATCTAAGAATCCATTTAGTTGATGAAAGAACATGGGGGGGGGCAGGCCTGAGGATCAACCGGTAGTGGGTNNNNGGAAGGGGATTGCTTTTTCCTTGACCAATTCTTTCAAAAAACGAATCGGATTCATGGGTCATAATAAGATAATTCATGATTCAGATGCTTAATAATAAGATAATAAGAAGGAATAATCAAATTGAATTCATGAATTTACCCGGCAATTTATGGTCCAATAAAGGATTTTTATCTTCGAAACCCATTGGAAGGGGTAGTGCACGAGAAAAAAAATCATGCGGAAATGATCGACTCCTTGGATGCCCCAAAATACTATGAGTGTCGGAA

104_20180718_03_04_trnT(GUU)-comp-psbD-comp CATAGAGATGAAATTGGAACAAGTTGACCCCCTTTTCTATTTATTTTCATTTCTTTGGACTCCGCAAGAATTTGCCGATATTTCCAATTCAATCGTCTTGTCTTGTTCCTAGATGTTCTATAGGAAAAAATTGTCATTTCGTTCCTCTACGGAGAACCTTTTATTCTAAATTCTAAATCACAGGATCTAAATTCTAAATCACAGGATAAGAAAAAAATTCACTATCTTTCTTTGATTACAGGATCAAGATTCATTTANNNNTTATCTATACTATATAATATATTCNNNNNNTATAGATATTTCTATTCTATATTTATATTTATAGCTATCAGATCGTGGCTTGATGTACCAAAAATTTCCATTTCGTTGCATCCAATATTTTTGTTCCGACCATCGTATGAAGAAAGCATGCAAGATAAATACTATCATTTCCAATCTCCTATTTTAATTTTAATATTGTANNNNNNTTGAAGTGAAGTAAAAATTTGGAAACCCTCTCTTTTCTAACAGGGAAAAATAAATCAAAAAATATTAGTAATTTAGTCTACATAAAAATTAGAATATAAAGAGAGTTCTTTTTCTTAATCTCATGAACAAGATCTAAGAATCCATTTAGTTGATGAAAGAACATGGGGGGGGGCAGGCCTGAGGATCAACCGGTAGTGGGTNNNNGGAAGGGGATTGCTTTTTCCTTGACCAATTCTTTCAAAAAACGAATCGGATTCATGGGTCATAATAAGATAATTCATGATTCAGATGCTTAATAATAAGATAATAAGAAGGAATAATCAAATTGAATTCATGAATTTACCCGGCAATTTATGGTCCAATAAAGGATTTTTATCTTCGAAACCCATTGGAAGGGGTAGTGCACGAGAAAAAAAATCATGCGGAAATGATCGACTCCTTGGATGCCCCAAAATACTATGAGTGTCGGAA

105_20180718_03_05_trnT(GUU)-comp-psbD-comp CATAGAGATGAAATTGGAACAAGTTGACCCCCTTTTCTATTTATTTTCATTTCTTTGGACTCCGCAAGAATTTGCCGATATTTCCAATTCAATCGTCTTGTCTTGTTCCTAGATGTTCTATAGGAAAAAATTGTCATTTCGTTCCTCTACGGAGAACCTTTTATTCTAAATTCTAAATCACAGGATCTAAATTCTAAATCACAGGATAAGAAAAAAATTCACTATCTTTCTTTGATTACAGGATCAAGATTCATTTANNNNTTATCTATACTATATAATATATTCNNNNNNTATAGATATTTCTATTCTATATTTATATTTATAGCTATCAGATCGTGGCTTGATGTACCAAAAATTTCCATTTCGTTGCATCCAATATTTTTGTTCCGACCATCGTATGAAGAAAGCATGCAAGATAAATACTATCATTTCCAATCTCCTATTTTAATTTTAATATTGTANNNNNNTTGAAGTGAAGTAAAAATTTGGAAACCCTCTCTTTTCTAACAGGGAAAAATAAATCAAAAAATATTAGTAATTTAGTCTACATAAAAATTAGAATATAAAGAGAGTTCTTTTTCTTAATCTCATGAACAAGATCTAAGAATCCATTTAGTTGATGAAAGAACATGGGGGGGGGCAGGCCTGAGGATCAACCGGTAGTGGGTNNNNGGAAGGGGATTGCTTTTTCCTTGACCAATTCTTTCAAAAAACGAATCGGATTCATGGGTCATAATAAGATAATTCATGATTCAGATGCTTAATAATAAGATAATAAGAAGGAATAATCAAATTGAATTCATGAATTTACCCGGCAATTTATGGTCCAATAAAGGATTTTTATCTTCGAAACCCATTGGAAGGGGTAGTGCACGAGAAAAAAAATCATGCGGAAATGATCGACTCCTTGGATGCCCCAAAATACTATGAGTGTCGGAA

106_20180718_03_06_trnT(GUU)-comp-psbD-comp CATAGAGATGAAATTGGAACAAGTTGACCCCCTTTTCTATTTATTTTCATTTCTTTGGACTCCGCAAGAATTTGCCGATATTTCCAATTCAATCGTCTTGTCTTGTTCCTAGATGTTCTATAGGAAAAAATTGTCATTTCGTTCCTCTACGGAGAACCTTTTATTCTAAATTCTAAATCACAGGATCTAAATTCTAAATCACAGGATAAGAAAAAAATTCACTATCTTTCTTTGATTACAGGATCAAGATTCATTTANNNNTTATCTATACTATATAATATATTCNNNNNNTATAGATATTTCTATTCTATATTTATATTTATAGCTATCAGATCGTGGCTTGATGTACCAAAAATTTCCATTTCGTTGCATCCAATATTTTTGTTCCGACCATCGTATGAAGAAAGCATGCAAGATAAATACTATCATTTCCAATCTCCTATTTTAATTTTAATATTGTANNNNNNTTGAAGTGAAGTAAAAATTTGGAAACCCTCTCTTTTCTAACAGGGAAAAATAAATCAAAAAATATTAGTAATTTAGTCTACATAAAAATTAGAATATAAAGAGAGTTCTTTTTCTTAATCTCATGAACAAGATCTAAGAATCCATTTAGTTGATGAAAGAACATGGGGGGGGGCAGGCCTGAGGATCAACCGGTAGTGGGTNNNNGGAAGGGGATTGCTTTTTCCTTGACCAATTCTTTCAAAAAACGAATCGGATTCATGGGTCATAATAAGATAATTCATGATTCAGATGCTTAATAATAAGATAATAAGAAGGAATAATCAAATTGAATTCATGAATTTACCCGGCAATTTATGGTCCAATAAAGGATTTTTATCTTCGAAACCCATTGGAAGGGGTAGTGCACGAGAAAAAAAATCATGCGGAAATGATCGACTCCTTGGATGCCCCAAAATACTATGAGTGTCGGAA

107_20180718_06_01_trnT(GUU)-comp-psbD-comp CATAGAGATGAAATTGGAACAAGTTGACCCCCTTTTCTATTTATTTTCATTTCTTTGGACTCCGCAAGAATTTGCCGATATTTCCAATTCAATCGTCTTGTCTTGTTCCTAGATGTTCTATAGGAAAAAATTGTCATTTCGTTCCTCTACGGAGAACCTTTTATTCTAAATTCTAAATCACAGGATCTAAATTCTAAATCACAGGATAAGAAAAAAATTCACTATCTTTCTTTGATTACAGGATCAAGATTCATTTANNNNTTATCTATACTATATAATATATTCNNNNNNTATAGATATTTCTATTCTATATTTATATTTATAGCTATCAGATCGTGGCTTGATGTACCAAAAATTTCCATTTCGTTGCATCCAATATTTTTGTTCCGACCATCGTATGAAGAAAGCATGCAAGATAAATACTATCATTTCCAATCTCCTATTTTAATTTTAATATTGTANNNNNNTTGAAGTGAAGTAAAAATTTGGAAACCCTCTCTTTTCTAACAGGGAAAAATAAATCAAAAAATATTAGTAATTTAGTCTACATAAAAATTAGAATATAAAGAGAGTTCTTTTTCTTAATCTCATGAACAAGATCTAAGAATCCATTTAGTTGATGAAAGAACATGGGGGGGGGCAGGCCTGAGGATCAACCGGTAGTGGGTNNNNGGAAGGGGATTGCTTTTTCCTTGACCAATTCTTTCAAAAAACGAATCGGATTCATGGGTCATAATAAGATAATTCATGATTCAGATGCTTAATAATAAGATAATAAGAAGGAATAATCAAATTGAATTCATGAATTTACCCGGCAATTTATGGTCCAATAAAGGATTTTTATCTTCGAAACCCATTGGAAGGGGTAGTGCACGAGAAAAAAAATCATGCGGAAATGATCGACTCCTTGGATGCCCCAAAATACTATGAGTGTCGGAA

108_20180718_06_02_trnT(GUU)-comp-psbD-comp CATAGAGATGAAATTGGAACAAGTTGACCCCCTTTTCTATTTATTTTCATTTCTTTGGACTCCGCAAGAATTTGCCGATATTTCCAATTCAATCGTCTTGTCTTGTTCCTAGATGTTCTATAGGAAAAAATTGTCATTTCGTTCCTCTACGGAGAACCTTTTATTCTAAATTCTAAATCACAGGATCTAAATTCTAAATCACAGGATAAGAAAAAAATTCACTATCTTTCTTTGATTACAGGATCAAGATTCATTTANNNNTTATCTATACTATATAATATATTCNNNNNNTATAGATATTTCTATTCTATATTTAGATTTATAGCTATCAGATCGTGGCTTGATGTACCAAAAATTTCCATTTCGTTGCATCCAATATTTTTGTTCCGACCATCGTATGAAGAAAGCATGCAAGATAAATACTATCATTTCCAATCTCCTNNNNNNATTTTAATATTGTANNNNNNTTGAAGTGAAGTAAAAATTTGGAAACCCTCTCTTTTCTAACAGGGAAAAATAAATCAAAAAATATTAGTAATTTAGTCTACATAAAAATTAGAATATAAAGAGAGTTCTTTTTCTTAATCTCATGAACAAGATCTAAGAATCCATTTAGTTGATGAAAGAACATGGGGGGGGGCAGGCCTGAGGATCAACCGGTAGTGGGTNNNNGGAAGGGGATTGCTTTTTCCTTGACCAATTCTTTCAAAAAACGAATCGGATTCATGGGTCATAATAAGATAATTCATGATTCAGATGCTTAATAATAAGATAATAAGAAGGAATAATCAAATTGAATTCATGAATTTACCCGGCAATTTATGGTCCAATAAAGGATTTTTATCTTCGAAACCCATTGGAAGGGGTAGTGCACGAGAAAAAAAATCATGCGGAAATGATCGACTCCTTGGATGCCCCAAAATACTATGAGTGTCGGAA

109_20180718_06_03_trnT(GUU)-comp-psbD-comp CATAGAGATGAAATTGGAACAAGTTGACCCCCTTTTCTATTTATTTTCATTTCTTTGGACTCCGCAAGAATTTGCCGATATTTCCAATTCAATCGTCTTGTCTTGTTCCTAGATGTTCTATAGGAAAAAATTGTCATTTCGTTCCTCTACGGAGAACCTTTTATTCTAAATTCTAAATCACAGGATCTAAATTCTAAATCACAGGATAAGAAAAAAATTCACTATCTTTCTTTGATTACAGGATCAAGATTCATTTANNNNTTATCTATACTATATAATATATTCNNNNNNTATAGATATTTCTATTCTATATTTATATTTATAGCTATCAGATCGTGGCTTGATGTACCAAAAATTTCCATTTCGTTGCATCCAATATTTTTGTTCCGACCATCGTATGAAGAAAGCATGCAAGATAAATACTATCATTTCCAATCTCCTATTTTAATTTTAATATTGTANNNNNNTTGAAGTGAAGTAAAAATTTGGAAACCCTCTCTTTTCTAACAGGGAAAAATAAATCAAAAAATATTAGTAATTTAGTCTACATAAAAATTAGAATATAAAGAGAGTTCTTTTTCTTAATCTCATGAACAAGATCTAAGAATCCATTTAGTTGATGAAAGAACATGGGGGGGGGCAGGCCTGAGGATCAACCGGTAGTGGGTNNNNGGAAGGGGATTGCTTTTTCCTTGACCAATTCTTTCAAAAAACGAATCGGATTCATGGGTCATAATAAGATAATTCATGATTCAGATGCTTAATAATAAGATAATAAGAAGGAATAATCAAATTGAATTCATGAATTTACCCGGCAATTTATGGTCCAATAAAGGATTTTTATCTTCGAAACCCATTGGAAGGGGTAGTGCACGAGAAAAAAAATCATGCGGAAATGATCGACTCCTTGGATGCCCCAAAATACTATGAGTGTCGGAA

110_20180718_06_04_trnT(GUU)-comp-psbD-comp CATAGAGATGAAATTGGAACAAGTTGACCCCCTTTTCTATTTATTTTCATTTCTTTGGACTCCGCAAGAATTTGCCGATATTTCCAATTCAATCGTCTTGTCTTGTTCCTAGATGTTCTATAGGAAAAAATTGTCATTTCGTTCCTCTACGGAGAACCTTTTATTCTAAATTCTAAATCACAGGATCTAAATTCTAAATCACAGGATAAGAAAAAAATTCACTATCTTTCTTTGATTACAGGATCAAGATTCATTTANNNNTTATCTATACTATATAATATATTCNNNNNNTATAGATATTTCTATTCTATATTTATATTTATAGCTATCAGATCGTGGCTTGATGTACCAAAAATTTCCATTTCGTTGCATCCAATATTTTTGTTCCGACCATCGTATGAAGAAAGCATGCAAGATAAATACTATCATTTCCAATCTCCTATTTTAATTTTAATATTGTANNNNNNTTGAAGTGAAGTAAAAATTTGGAAACCCTCTCTTTTCTAACAGGGAAAAATAAATCAAAAAATATTAGTAATTTAGTCTACATAAAAATTAGAATATAAAGAGAGTTCTTTTTCTTAATCTCATGAACAAGATCTAAGAATCCATTTAGTTGATGAAAGAACATGGGGGGGGGCAGGCCTGAGGATCAACCGGTAGTGGGTNNNNGGAAGGGGATTGCTTTTTCCTTGACCAATTCTTTCAAAAAACGAATCGGATTCATGGGTCATAATAAGATAATTCATGATTCAGATGCTTAATAATAAGATAATAAGAAGGAATAATCAAATTGAATTCATGAATTTACCCGGCAATTTATGGTCCAATAAAGGATTTTTATCTTCGAAACCCATTGGAAGGGGTAGTGCACGAGAAAAAAAATCATGCGGAAATGATCGACTCCTTGGATGCCCCAAAATACTATGAGTGTCGGAA

111_20180718_06_05_trnT(GUU)-comp-psbD-comp CATAGAGATGAAATTGGAACAAGTTGACCCCCTTTTCTATTTATTTTCATTTCTTTGGACTCCGCAAGAATTTGCCGATATTTCCAATTCAATCGTCTTGTCTTGTTCCTAGATGTTCTATAGGAAAAAATTGTCATTTCGTTCCTCTACGGAGAACCTTTTATTCTAAATTCTAAATCACAGGATCTAAATTCTAAATCACAGGATAAGAAAAAAATTCACTATCTTTCTTTGATTACAGGATCAAGATTCATTTANNNNTTATCTATACTATATAATATATTCNNNNNNTATAGATATTTCTATTCTATATTTATATTTATAGCTATCAGATCGTGGCTTGATGTACCAAAAATTTCCATTTCGTTGCATCCAATATTTTTGTTCCGACCATCGTATGAAGAAAGCATGCAAGATAAATACTATCATTTCCAATCTCCTATTTTAATTTTAATATTGTANNNNNNTTGAAGTGAAGTAAAAATTTGGAAACCCTCTCTTTTCTAACAGGGAAAAATAAATCAAAAAATATTAGTAATTTAGTCTACATAAAAATTAGAATATAAAGAGAGTTCTTTTTCTTAATCTCATGAACAAGATCTAAGAATCCATTTAGTTGATGAAAGAACATGGGGGGGGGCAGGCCTGAGGATCAACCGGTAGTGGGTNNNNGGAAGGGGATTGCTTTTTCCTTGACCAATTCTTTCAAAAAACGAATCGGATTCATGGGTCATAATAAGATAATTCATGATTCAGATGCTTAATAATAAGATAATAAGAAGGAATAATCAAATTGAATTCATGAATTTACCCGGCAATTTATGGTCCAATAAAGGATTTTTATCTTCGAAACCCATTGGAAGGGGTAGTGCACGAGAAAAAAAATCATGCGGAAATGATCGACTCCTTGGATGCCCCAAAATACTATGAGTGTCGGAA

112_20180718_08_01_trnT(GUU)-comp-psbD-comp CATAGAGATGAAATTGGAACAAGTTGACCCCCTTTTCTATTTATTTTCATTTCTTTGGACTCCGCAAGAATTTGCCGATATTTCCAATTCAATCGTCTTGTCTTGTTCCTAGATGTTCTATAGGAAAAAATTGTCATTTCGTTCCTCTACGGAGAACCTTTTATTCTAAATTCTAAATCACAGGATCTAAATTCTAAATCACAGGATAAGAAAAAAATTCACTATCTTTCTTTGATTACAGGATCAAGATTCATTTANNNNTTATCTATACTATATAATATATTCNNNNNNTATAGATATTTCTATTCTATATTTATATTTATAGCTATCAGATCGTGGCTTGATGTACCAAAAATTTCCATTTCGTTGCATCCAATATTTTTGTTCCGACCATCGTATGAAGAAAGCATGCAAGATAAATACTATCATTTCCAATCTCCTATTTTAATTTTAATATTGTANNNNNNTTGAAGTGAAGTAAAAATTTGGAAACCCTCTCTTTTCTAACAGGGAAAAATAAATCAAAAAATATTAGTAATTTAGTCTACATAAAAATTAGAATATAAAGAGAGTTCTTTTTCTTAATCTCATGAACAAGATCTAAGAATCCATTTAGTTGATGAAAGAACATGGGGGGGGGCAGGCCTGAGGATCAACCGGTAGTGGGTNNNNGGAAGGGGATTGCTTTTTCCTTGACCAATTCTTTCAAAAAACGAATCGGATTCATGGGTCATAATAAGATAATTCATGATTCAGATGCTTAATAATAAGATAATAAGAAGGAATAATCAAATTGAATTCATGAATTTACCCGGCAATTTATGGTCCAATAAAGGATTTTTATCTTCGAAACCCATTGGAAGGGGTAGTGCACGAGAAAAAAAATCATGCGGAAATGATCGACTCCTTGGATGCCCCAAAATACTATGAGTGTCGGAA

113_20180718_08_02_trnT(GUU)-comp-psbD-comp CATAGAGATGAAATTGGAACAAGTTGACCCCCTTTTCTATTTATTTTCATTTCTTTGGACTCCGCAAGAATTTGCCGATATTTCCAATTCAATCGTCTTGTCTTGTTCCTAGATGTTCTATAGGAAAAAATTGTCATTTCGTTCCTCTACGGAGAACCTTTTATTCTAAATTCTAAATCACAGGATCTAAATTCTAAATCACAGGATAAGAAAAAAATTCACTATCTTTCTTTGATTACAGGATCAAGATTCATTTANNNNTTATCTATACTATATAATATATTCNNNNNNTATAGATATTTCTATTCTATATTTATATTTATAGCTATCAGATCGTGGCTTGATGTACCAAAAATTTCCATTTCGTTGCATCCAATATTTTTGTTCCGACCATCGTATGAAGAAAGCATGCAAGATAAATACTATCATTTCCAATCTCCTATTTTAATTTTAATATTGTANNNNNNTTGAAGTGAAGTAAAAATTTGGAAACCCTCTCTTTTCTAACAGGGAAAAATAAATCAAAAAATATTAGTAATTTAGTCTACATAAAAATTAGAATATAAAGAGAGTTCTTTTTCTTAATCTCATGAACAAGATCTAAGAATCCATTTAGTTGATGAAAGAACATGGGGGGGGGCAGGCCTGAGGATCAACCGGTAGTGGGTNNNNGGAAGGGGATTGCTTTTTCCTTGACCAATTCTTTCAAAAAACGAATCGGATTCATGGGTCATAATAAGATAATTCATGATTCAGATGCTTAATAATAAGATAATAAGAAGGAATAATCAAATTGAATTCATGAATTTACCCGGCAATTTATGGTCCAATAAAGGATTTTTATCTTCGAAACCCATTGGAAGGGGTAGTGCACGAGAAAAAAAATCATGCGGAAATGATCGACTCCTTGGATGCCCCAAAATACTATGAGTGTCGGAA

114_20180718_08_03_trnT(GUU)-comp-psbD-comp CATAGAGATGAAATTGGAACAAGTTGACCCCCTTTTCTATTTATTTTCATTTCTTTGGACTCCGCAAGAATTTGCCGATATTTCCAATTCAATCGTCTTGTCTTGTTCCTAGATGTTCTATAGGAAAAAATTGTCATTTCGTTCCTCTACGGAGAACCTTTTATTCTAAATTCTAAATCACAGGATCTAAATTCTAAATCACAGGATAAGAAAAAAATTCACTATCTTTCTTTGATTACAGGATCAAGATTCATTTANNNNTTATCTATACTATATAATATATTCNNNNNNTATAGATATTTCTATTCTATATTTATATTTATAGCTATCAGATCGTGGCTTGATGTACCAAAAATTTCCATTTCGTTGCATCCAATATTTTTGTTCCGACCATCGTATGAAGAAAGCATGCAAGATAAATACTATCATTTCCAATCTCCTATTTTAATTTTAATATTGTANNNNNNTTGAAGTGAAGTAAAAATTTGGAAACCCTCTCTTTTCTAACAGGGAAAAATAAATCAAAAAATATTAGTAATTTAGTCTACATAAAAATTAGAATATAAAGAGAGTTCTTTTTCTTAATCTCATGAACAAGATCTAAGAATCCATTTAGTTGATGAAAGAACATGGGGGGGGGCAGGCCTGAGGATCAACCGGTAGTGGGTNNNNGGAAGGGGATTGCTTTTTCCTTGACCAATTCTTTCAAAAAACGAATCGGATTCATGGGTCATAATAAGATAATTCATGATTCAGATGCTTAATAATAAGATAATAAGAAGGAATAATCAAATTGAATTCATGAATTTACCCGGCAATTTATGGTCCAATAAAGGATTTTTATCTTCGAAACCCATTGGAAGGGGTAGTGCACGAGAAAAAAAATCATGCGGAAATGATCGACTCCTTGGATGCCCCAAAATACTATGAGTGTCGGAA

115_20180718_08_04_trnT(GUU)-comp-psbD-comp CATAGAGATGAAATTGGAACAAGTTGACCCCCTTTTCTATTTATTTTCATTTCTTTGGACTCCGCAAGAATTTGCCGATATTTCCAATTCAATCGTCTTGTCTTGTTCCTAGATGTTCTATAGGAAAAAATTGTCATTTCGTTCCTCTACGGAGAACCTTTTATTCTAAATTCTAAATCACAGGATCTAAATTCTAAATCACAGGATAAGAAAAAAATTCACTATCTTTCTTTGATTACAGGATCAAGATTCATTTANNNNTTATCTATACTATATAATATATTCNNNNNNTATAGATATTTCTATTCTATATTTATATTTATAGCTATCAGATCGTGGCTTGATGTACCAAAAATTTCCATTTCGTTGCATCCAATATTTTTGTTCCGACCATCGTATGAAGAAAGCATGCAAGATAAATACTATCATTTCCAATCTCCTATTTTAATTTTAATATTGTANNNNNNTTGAAGTGAAGTAAAAATTTGGAAACCCTCTCTTTTCTAACAGGGAAAAATAAATCAAAAAATATTAGTAATTTAGTCTACATAAAAATTAGAATATAAAGAGAGTTCTTTTTCTTAATCTCATGAACAAGATCTAAGAATCCATTTAGTTGATGAAAGAACATGGGGGGGGGCAGGCCTGAGGATCAACCGGTAGTGGGTNNNNGGAAGGGGATTGCTTTTTCCTTGACCAATTCTTTCAAAAAACGAATCGGATTCATGGGTCATAATAAGATAATTCATGATTCAGATGCTTAATAATAAGATAATAAGAAGGAATAATCAAATTGAATTCATGAATTTACCCGGCAATTTATGGTCCAATAAAGGATTTTTATCTTCGAAACCCATTGGAAGGGGTAGTGCACGAGAAAAAAAATCATGCGGAAATGATCGACTCCTTGGATGCCCCAAAATACTATGAGTGTCGGAA

116_20180718_08_05_trnT(GUU)-comp-psbD-comp CATAGAGATGAAATTGGAACAAGTTGACCCCCTTTTCTATTTATTTTCATTTCTTTGGACTCCGCAAGAATTTGCCGATATTTCCAATTCAATCGTCTTGTCTTGTTCCTAGATGTTCTATAGGAAAAAATTGTCATTTCGTTCCTCTACGGAGAACCTTTTATTCTAAATTCTAAATCACAGGATCTAAATTCTAAATCACAGGATAAGAAAAAAATTCACTATCTTTCTTTGATTACAGGATCAAGATTCATTTANNNNTTATCTATACTATATAATATATTCNNNNNNTATAGATATTTCTATTCTATATTTATATTTATAGCTATCAGATCGTGGCTTGATGTACCAAAAATTTCCATTTCGTTGCATCCAATATTTTTGTTCCGACCATCGTATGAAGAAAGCATGCAAGATAAATACTATCATTTCCAATCTCCTATTTTAATTTTAATATTGTANNNNNNTTGAAGTGAAGTAAAAATTTGGAAACCCTCTCTTTTCTAACAGGGAAAAATAAATCAAAAAATATTAGTAATTTAGTCTACATAAAAATTAGAATATAAAGAGAGTTCTTTTTCTTAATCTCATGAACAAGATCTAAGAATCCATTTAGTTGATGAAAGAACATGGGGGGGGGCAGGCCTGAGGATCAACCGGTAGTGGGTNNNNGGAAGGGGATTGCTTTTTCCTTGACCAATTCTTTCAAAAAACGAATCGGATTCATGGGTCATAATAAGATAATTCATGATTCAGATGCTTAATAATAAGATAATAAGAAGGAATAATCAAATTGAATTCATGAATTTACCCGGCAATTTATGGTCCAATAAAGGATTTTTATCTTCGAAACCCATTGGAAGGGGTAGTGCACGAGAAAAAAAATCATGCGGAAATGATCGACTCCTTGGATGCCCCAAAATACTATGAGTGTCGGAA

117_20180807_03_01_trnT(GUU)-comp-psbD-comp CATAGAGATGAAATTGGAACAAGTTGACCCCCTTTTCTATTTATTTTCATTTCTTTGGACTCCGCAAGAATTTGCCGATATTTCCAATTCAATCGTCTTGTCTTGTTCCTAGATGTTCTATAGGAAAAAATTGTCATTTCGTTCCTCTACGGAGAACCTTTTATNNNNNNNNNNNNNNNNNNNNNTCTAAATTCTAAATCACAGGATAAGAAAAAAATTCACTATCTTTCTTTGATTACAGGATCAAGATTCATTTATATCTTATCTATACTATATAATATATTCNNNNNNTATAGATATTTCTATTCTATATTTAGATTTATAGCTATCAGATCGTGGCTTGATGTACCAAAAATTTCCATTTCGTTGCATCCAATATTTTTGTTCCGACCATCGTATGAAGAAAGCATGCAAGATAAATACTATCATTTCCAATCTCCTATTTTAATTTTAATATTGTANNNNNNTTGAAGTGAAGTAAAAATTTGGAAACCCTCTCTTTTCTAACAGGGAAAAATAAATCAAAAAATATTAGTAATTTAGTCTACATAAAAATTAGAATATAAAGAGAGTTCTTTTTCTTAATCTCATGAACAAGATCTAAGAATCCATTTAGTTGATGAAAGAACATGGGGGGGGGCAGGCCTGAGGATCAACCGGTAGTGGGTNNNNGGAAGGGGATTGCTTTTTCCTTGACCAATTCTTTCAAAAAACGAATCGGATTCATGGGTCATAATAAGATAATTCATGATTCAGATGCTTAATAATAAGATAATAAGAAGGAATAATCAAATTGAATTCATGAATTTACCCGGCAATTTATGGTCCAATAAAGGATTTTTATCTTCGAAACCCATTGGAAGGGGTAGTGCACGAGAAAAAAAATCATGCGGAAATGATCGACTCCTTGGATGCCCCAAAATACTATGAGTGTCGGAA

118_20180807_03_02_trnT(GUU)-comp-psbD-comp CATAGAGATGAAATTGGAACAAGTTGACCCCCTTTTCTATTTATTTTCATTTCTTTGGACTCCGCAAGAATTTGCCGATATTTCCAATTCAATCGTCTTGTCTTGTTCCTAGATGTTCTATAGGAAAAAATTGTCATTTCGTTCCTCTACGGAGAACCTTTTATNNNNNNNNNNNNNNNNNNNNNTCTAAATTCTAAATCACAGGATAAGAAAAAAATTCACTATCTTTCTTTGATTACAGGATCAAGATTCATTTATATCTTATCTATACTATATAATATATTCNNNNNNTATAGATATTTCTATTCTATATTTAGATTTATAGCTATCAGATCGTGGCTTGATGTACCAAAAATTTCCATTTCGTTGCATCCAATATTTTTGTTCCGACCATCGTATGAAGAAAGCATGCAAGATAAATACTATCATTTCCAATCTCCTATTTTAATTTTAATATTGTANNNNNNTTGAAGTGAAGTAAAAATTTGGAAACCCTCTCTTTTCTAACAGGGAAAAATAAATCAAAAAATATTAGTAATTTAGTCTACATAAAAATTAGAATATAAAGAGAGTTCTTTTTCTTAATCTCATGAACAAGATCTAAGAATCCATTTAGTTGATGAAAGAACATGGGGGGGGGCAGGCCTGAGGATCAACCGGTAGTGGGTNNNNGGAAGGGGATTGCTTTTTCCTTGACCAATTCTTTCAAAAAACGAATCGGATTCATGGGTCATAATAAGATAATTCATGATTCAGATGCTTAATAATAAGATAATAAGAAGGAATAATCAAATTGAATTCATGAATTTACCCGGCAATTTATGGTCCAATAAAGGATTTTTATCTTCGAAACCCATTGGAAGGGGTAGTGCACGAGAAAAAAAATCATGCGGAAATGATCGACTCCTTGGATGCCCCAAAATACTATGAGTGTCGGAA

119_20180807_03_03_trnT(GUU)-comp-psbD-comp CATAGAGATGAAATTGGAACAAGTTGACCCCCTTTTCTATTTATTTTCATTTCTTTGGACTCCGCAAGAATTTGCCGATATTTCCAATTCAATCGTCTTGTCTTGTTCCTAGATGTTCTATAGGAAAAAATTGTCATTTCGTTCCTCTACGGAGAACCTTTTATNNNNNNNNNNNNNNNNNNNNNTCTAAATTCTAAATCACAGGATAAGAAAAAAATTCACTATCTTTCTTTGATTACAGGATCAAGATTCATTTATATCTTATCTATACTATATAATATATTCNNNNNNTATAGATATTTCTATTCTATATTTAGATTTATAGCTATCAGATCGTGGCTTGATGTACCAAAAATTTCCATTTCGTTGCATCCAATATTTTTGTTCCGACCATCGTATGAAGAAAGCATGCAAGATAAATACTATCATTTCCAATCTCCTATTTTAATTTTAATATTGTANNNNNNTTGAAGTGAAGTAAAAATTTGGAAACCCTCTCTTTTCTAACAGGGAAAAATAAATCAAAAAATATTAGTAATTTAGTCTACATAAAAATTAGAATATAAAGAGAGTTCTTTTTCTTAATCTCATGAACAAGATCTAAGAATCCATTTAGTTGATGAAAGAACATGGGGGGGGGCAGGCCTGAGGATCAACCGGTAGTGGGTNNNNGGAAGGGGATTGCTTTTTCCTTGACCAATTCTTTCAAAAAACGAATCGGATTCATGGGTCATAATAAGATAATTCATGATTCAGATGCTTAATAATAAGATAATAAGAAGGAATAATCAAATTGAATTCATGAATTTACCCGGCAATTTATGGTCCAATAAAGGATTTTTATCTTCGAAACCCATTGGAAGGGGTAGTGCACGAGAAAAAAAATCATGCGGAAATGATCGACTCCTTGGATGCCCCAAAATACTATGAGTGTCGGAA

120_20180807_03_04_trnT(GUU)-comp-psbD-comp CATAGAGATGAAATTGGAACAAGTTGACCCCCTTTTCTATTTATTTTCATTTCTTTGGACTCCGCAAGAATTTGCCGATATTTCCAATTCAATCGTCTTGTCTTGTTCCTAGATGTTCTATAGGAAAAAATTGTCATTTCGTTCCTCTACGGAGAACCTTTTATNNNNNNNNNNNNNNNNNNNNNTCTAAATTCTAAATCACAGGATAAGAAAAAAATTCACTATCTTTCTTTGATTACAGGATCAAGATTCATTTATATCTTATCTATACTATATAATATATTCNNNNNNTATAGATATTTCTATTCTATATTTAGATTTATAGCTATCAGATCGTGGCTTGATGTACCAAAAATTTCCATTTCGTTGCATCCAATATTTTTGTTCCGACCATCGTATGAAGAAAGCATGCAAGATAAATACTATCATTTCCAATCTCCTATTTTAATTTTAATATTGTANNNNNNTTGAAGTGAAGTAAAAATTTGGAAACCCTCTCTTTTCTAACAGGGAAAAATAAATCAAAAAATATTAGTAATTTAGTCTACATAAAAATTAGAATATAAAGAGAGTTCTTTTTCTTAATCTCATGAACAAGATCTAAGAATCCATTTAGTTGATGAAAGAACATGGGGGGGGGCAGGCCTGAGGATCAACCGGTAGTGGGTNNNNGGAAGGGGATTGCTTTTTCCTTGACCAATTCTTTCAAAAAACGAATCGGATTCATGGGTCATAATAAGATAATTCATGATTCAGATGCTTAATAATAAGATAATAAGAAGGAATAATCAAATTGAATTCATGAATTTACCCGGCAATTTATGGTCCAATAAAGGATTTTTATCTTCGAAACCCATTGGAAGGGGTAGTGCACGAGAAAAAAAATCATGCGGAAATGATCGACTCCTTGGATGCCCCAAAATACTATGAGTGTCGGAA

121_20180807_03_05_trnT(GUU)-comp-psbD-comp CATAGAGATGAAATTGGAACAAGTTGACCCCCTTTTCTATTTATTTTCATTTCTTTGGACTCCGCAAGAATTTGCCGATATTTCCAATTCAATCGTCTTGTCTTGTTCCTAGATGTTCTATAGGAAAAAATTGTCATTTCGTTCCTCTACGGAGAACCTTTTATNNNNNNNNNNNNNNNNNNNNNTCTAAATTCTAAATCACAGGATAAGAAAAAAATTCACTATCTTTCTTTGATTACAGGATCAAGATTCATTTATATCTTATCTATACTATATAATATATTCNNNNNNTATAGATATTTCTATTCTATATTTAGATTTATAGCTATCAGATCGTGGCTTGATGTACCAAAAATTTCCATTTCGTTGCATCCAATATTTTTGTTCCGACCATCGTATGAAGAAAGCATGCAAGATAAATACTATCATTTCCAATCTCCTATTTTAATTTTAATATTGTANNNNNNTTGAAGTGAAGTAAAAATTTGGAAACCCTCTCTTTTCTAACAGGGAAAAATAAATCAAAAAATATTAGTAATTTAGTCTACATAAAAATTAGAATATAAAGAGAGTTCTTTTTCTTAATCTCATGAACAAGATCTAAGAATCCATTTAGTTGATGAAAGAACATGGGGGGGGGCAGGCCTGAGGATCAACCGGTAGTGGGTNNNNGGAAGGGGATTGCTTTTTCCTTGACCAATTCTTTCAAAAAACGAATCGGATTCATGGGTCATAATAAGATAATTCATGATTCAGATGCTTAATAATAAGATAATAAGAAGGAATAATCAAATTGAATTCATGAATTTACCCGGCAATTTATGGTCCAATAAAGGATTTTTATCTTCGAAACCCATTGGAAGGGGTAGTGCACGAGAAAAAAAATCATGCGGAAATGATCGACTCCTTGGATGCCCCAAAATACTATGAGTGTCGGAA

122_20180827_01_01_trnT(GUU)-comp-psbD-comp CATAGAGATGAAATTGGAACAAGTTGACCCCCTTTTCTATTTATTTTCATTTCTTTGGACTCCGCAAGAATTTGCCGATATTTCCAATTCAATCGTCTTGTCTTGTTCCTAGATGTTCTATAGGAAAAAATTGTCATTTCGTTCCTCTACGGAGAACCTTTTATTCTAAATTCTAAATCACAGGATCTAAATTCTAAATCACAGGATAAGAAAAAAATTCACTATCTTTCTTTGATTACAGGATCAAGATTCATTTANNNNTTATCTATACTATATAATATATTCNNNNNNTATAGATATTTCTATTCTATATTTATATTTATAGCTATCAGATCGTGGCTTGATGTACCAAAAATTTCCATTTCGTTGCATCCAATATTTTTGTTCCGACCATCGTATGAAGAAAGCATGCAAGATAAATACTATCATTTCCAATCTCCTATTTTAATTTTAATATTGTANNNNNNTTGAAGTGAAGTAAAAATTTGGAAACCCTCTCTTTTCTAACAGGGAAAAATAAATCAAAAAATATTAGTAATTTAGTCTACATAAAAATTAGAATATAAAGAGAGTTCTTTTTCTTAATCTCATGAACAAGATCTAAGAATCCATTTAGTTGATGAAAGAACATGGGGGGGGGCAGGCCTGAGGATCAACCGGTAGTGGGTNNNNGGAAGGGGATTGCTTTTTCCTTGACCAATTCTTTCAAAAAACGAATCGGATTCATGGGTCATAATAAGATAATTCATGATTCAGATGCTTAATAATAAGATAATAAGAAGGAATAATCAAATTGAATTCATGAATTTACCCGGCAATTTATGGTCCAATAAAGGATTTTTATCTTCGAAACCCATTGGAAGGGGTAGTGCACGAGAAAAAAAATCATGCGGAAATGATCGACTCCTTGGATGCCCCAAAATACTATGAGTGTCGGAA

123_20180827_01_02_trnT(GUU)-comp-psbD-comp CATAGAGATGAAATTGGAACAAGTTGACCCCCTTTTCTATTTATTTTCATTTCTTTGGACTCCGCAAGAATTTGCCGATATTTCCAATTCAATCGTCTTGTCTTGTTCCTAGATGTTCTATAGGAAAAAATTGTCATTTCGTTCCTCTACGGAGAACCTTTTATTCTAAATTCTAAATCACAGGATCTAAATTCTAAATCACAGGATAAGAAAAAAATTCACTATCTTTCTTTGATTACAGGATCAAGATTCATTTANNNNTTATCTATACTATATAATATATTCNNNNNNTATAGATATTTCTATTCTATATTTATATTTATAGCTATCAGATCGTGGCTTGATGTACCAAAAATTTCCATTTCGTTGCATCCAATATTTTTGTTCCGACCATCGTATGAAGAAAGCATGCAAGATAAATACTATCATTTCCAATCTCCTATTTTAATTTTAATATTGTANNNNNNTTGAAGTGAAGTAAAAATTTGGAAACCCTCTCTTTTCTAACAGGGAAAAATAAATCAAAAAATATTAGTAATTTAGTCTACATAAAAATTAGAATATAAAGAGAGTTCTTTTTCTTAATCTCATGAACAAGATCTAAGAATCCATTTAGTTGATGAAAGAACATGGGGGGGGGCAGGCCTGAGGATCAACCGGTAGTGGGTNNNNGGAAGGGGATTGCTTTTTCCTTGACCAATTCTTTCAAAAAACGAATCGGATTCATGGGTCATAATAAGATAATTCATGATTCAGATGCTTAATAATAAGATAATAAGAAGGAATAATCAAATTGAATTCATGAATTTACCCGGCAATTTATGGTCCAATAAAGGATTTTTATCTTCGAAACCCATTGGAAGGGGTAGTGCACGAGAAAAAAAATCATGCGGAAATGATCGACTCCTTGGATGCCCCAAAATACTATGAGTGTCGGAA

124_20180827_01_03_trnT(GUU)-comp-psbD-comp CATAGAGATGAAATTGGAACAAGTTGACCCCCTTTTCTATTTATTTTCATTTCTTTGGACTCCGCAAGAATTTGCCGATATTTCCAATTCAATCGTCTTGTCTTGTTCCTAGATGTTCTATAGGAAAAAATTGTCATTTCGTTCCTCTACGGAGAACCTTTTATTCTAAATTCTAAATCACAGGATCTAAATTCTAAATCACAGGATAAGAAAAAAATTCACTATCTTTCTTTGATTACAGGATCAAGATTCATTTANNNNTTATCTATACTATATAATATATTCNNNNNNTATAGATATTTCTATTCTATATTTATATTTATAGCTATCAGATCGTGGCTTGATGTACCAAAAATTTCCATTTCGTTGCATCCAATATTTTTGTTCCGACCATCGTATGAAGAAAGCATGCAAGATAAATACTATCATTTCCAATCTCCTATTTTAATTTTAATATTGTANNNNNNTTGAAGTGAAGTAAAAATTTGGAAACCCTCTCTTTTCTAACAGGGAAAAATAAATCAAAAAATATTAGTAATTTAGTCTACATAAAAATTAGAATATAAAGAGAGTTCTTTTTCTTAATCTCATGAACAAGATCTAAGAATCCATTTAGTTGATGAAAGAACATGGGGGGGGGCAGGCCTGAGGATCAACCGGTAGTGGGTNNNNGGAAGGGGATTGCTTTTTCCTTGACCAATTCTTTCAAAAAACGAATCGGATTCATGGGTCATAATAAGATAATTCATGATTCAGATGCTTAATAATAAGATAATAAGAAGGAATAATCAAATTGAATTCATGAATTTACCCGGCAATTTATGGTCCAATAAAGGATTTTTATCTTCGAAACCCATTGGAAGGGGTAGTGCACGAGAAAAAAAATCATGCGGAAATGATCGACTCCTTGGATGCCCCAAAATACTATGAGTGTCGGAA

125_20180827_01_04_trnT(GUU)-comp-psbD-comp CATAGAGATGAAATTGGAACAAGTTGACCCCCTTTTCTATTTATTTTCATTTCTTTGGACTCCGCAAGAATTTGCCGATATTTCCAATTCAATCGTCTTGTCTTGTTCCTAGATGTTCTATAGGAAAAAATTGTCATTTCGTTCCTCTACGGAGAACCTTTTATTCTAAATTCTAAATCACAGGATCTAAATTCTAAATCACAGGATAAGAAAAAAATTCACTATCTTTCTTTGATTACAGGATCAAGATTCATTTANNNNTTATCTATACTATATAATATATTCNNNNNNTATAGATATTTCTATTCTATATTTATATTTATAGCTATCAGATCGTGGCTTGATGTACCAAAAATTTCCATTTCGTTGCATCCAATATTTTTGTTCCGACCATCGTATGAAGAAAGCATGCAAGATAAATACTATCATTTCCAATCTCCTATTTTAATTTTAATATTGTANNNNNNTTGAAGTGAAGTAAAAATTTGGAAACCCTCTCTTTTCTAACAGGGAAAAATAAATCAAAAAATATTAGTAATTTAGTCTACATAAAAATTAGAATATAAAGAGAGTTCTTTTTCTTAATCTCATGAACAAGATCTAAGAATCCATTTAGTTGATGAAAGAACATGGGGGGGGGCAGGCCTGAGGATCAACCGGTAGTGGGTNNNNGGAAGGGGATTGCTTTTTCCTTGACCAATTCTTTCAAAAAACGAATCGGATTCATGGGTCATAATAAGATAATTCATGATTCAGATGCTTAATAATAAGATAATAAGAAGGAATAATCAAATTGAATTCATGAATTTACCCGGCAATTTATGGTCCAATAAAGGATTTTTATCTTCGAAACCCATTGGAAGGGGTAGTGCACGAGAAAAAAAATCATGCGGAAATGATCGACTCCTTGGATGCCCCAAAATACTATGAGTGTCGGAA

126_20180827_01_05_trnT(GUU)-comp-psbD-comp CATAGAGATGAAATTGGAACAAGTTGACCCCCTTTTCTATTTATTTTCATTTCTTTGGACTCCGCAAGAATTTGCCGATATTTCCAATTCAATCGTCTTGTCTTGTTCCTAGATGTTCTATAGGAAAAAATTGTCATTTCGTTCCTCTACGGAGAACCTTTTATTCTAAATTCTAAATCACAGGATCTAAATTCTAAATCACAGGATAAGAAAAAAATTCACTATCTTTCTTTGATTACAGGATCAAGATTCATTTANNNNTTATCTATACTATATAATATATTCNNNNNNTATAGATATTTCTATTCTATATTTATATTTATAGCTATCAGATCGTGGCTTGATGTACCAAAAATTTCCATTTCGTTGCATCCAATATTTTTGTTCCGACCATCGTATGAAGAAAGCATGCAAGATAAATACTATCATTTCCAATCTCCTATTTTAATTTTAATATTGTANNNNNNTTGAAGTGAAGTAAAAATTTGGAAACCCTCTCTTTTCTAACAGGGAAAAATAAATCAAAAAATATTAGTAATTTAGTCTACATAAAAATTAGAATATAAAGAGAGTTCTTTTTCTTAATCTCATGAACAAGATCTAAGAATCCATTTAGTTGATGAAAGAACATGGGGGGGGGCAGGCCTGAGGATCAACCGGTAGTGGGTNNNNGGAAGGGGATTGCTTTTTCCTTGACCAATTCTTTCAAAAAACGAATCGGATTCATGGGTCATAATAAGATAATTCATGATTCAGATGCTTAATAATAAGATAATAAGAAGGAATAATCAAATTGAATTCATGAATTTACCCGGCAATTTATGGTCCAATAAAGGATTTTTATCTTCGAAACCCATTGGAAGGGGTAGTGCACGAGAAAAAAAATCATGCGGAAATGATCGACTCCTTGGATGCCCCAAAATACTATGAGTGTCGGAA

127_20180828_01_01_trnT(GUU)-comp-psbD-comp CATAGAGATGAAATTGGAACAAGTTGACCCCCTTTTCTATTTATTTTCATTTCTTTGGACTCCGCAAGAATTTGCCGATATTTCCAATTCAATCGTCTTGTCTTGTTCCTAGATGTTCTATAGGAAAAAATTGTCATTTCGTTCCTCTACGGAGAACCTTTTATTCTAAATTCTAAATCACAGGATCTAAATTCTAAATCACAGGATAAGAAAAAAATTCACTATCTTTCTTTGATTACAGGATCAAGATTCATTTANNNNTTATCTATACTATATAATATATTCNNNNNNTATAGATATTTCTATTCTATATTTATATTTATAGCTATCAGATCGTGGCTTGATGTACCAAAAATTTCCATTTCGTTGCATCCAATATTTTTGTTCCGACCATCGTATGAAGAAAGCATGCAAGATAAATACTATCATTTCCAATCTCCTATTTTAATTTTAATATTGTANNNNNNTTGAAGTGAAGTAAAAATTTGGAAACCCTCTCTTTTCTAACAGGGAAAAATAAATCAAAAAATATTAGTAATTTAGTCTACATAAAAATTAGAATATAAAGAGAGTTCTTTTTCTTAATCTCATGAACAAGATCTAAGAATCCATTTAGTTGATGAAAGAACATGGGGGGGGGCAGGCCTGAGGATCAACCGGTAGTGGGTNNNNGGAAGGGGATTGCTTTTTCCTTGACCAATTCTTTCAAAAAACGAATCGGATTCATGGGTCATAATAAGATAATTCATGATTCAGATGCTTAATAATAAGATAATAAGAAGGAATAATCAAATTGAATTCATGAATTTACCCGGCAATTTATGGTCCAATAAAGGATTTTTATCTTCGAAACCCATTGGAAGGGGTAGTGCACGAGAAAAAAAATCATGCGGAAATGATCGACTCCTTGGATGCCCCAAAATACTATGAGTGTCGGAA

128_20180828_01_02_trnT(GUU)-comp-psbD-comp CATAGAGATGAAATTGGAACAAGTTGACCCCCTTTTCTATTTATTTTCATTTCTTTGGACTCCGCAAGAATTTGCCGATATTTCCAATTCAATCGTCTTGTCTTGTTCCTAGATGTTCTATAGGAAAAAATTGTCATTTCGTTCCTCTACGGAGAACCTTTTATTCTAAATTCTAAATCACAGGATCTAAATTCTAAATCACAGGATAAGAAAAAAATTCACTATCTTTCTTTGATTACAGGATCAAGATTCATTTANNNNTTATCTATACTATATAATATATTCNNNNNNTATAGATATTTCTATTCTATATTTATATTTATAGCTATCAGATCGTGGCTTGATGTACCAAAAATTTCCATTTCGTTGCATCCAATATTTTTGTTCCGACCATCGTATGAAGAAAGCATGCAAGATAAATACTATCATTTCCAATCTCCTATTTTAATTTTAATATTGTANNNNNNTTGAAGTGAAGTAAAAATTTGGAAACCCTCTCTTTTCTAACAGGGAAAAATAAATCAAAAAATATTAGTAATTTAGTCTACATAAAAATTAGAATATAAAGAGAGTTCTTTTTCTTAATCTCATGAACAAGATCTAAGAATCCATTTAGTTGATGAAAGAACATGGGGGGGGGCAGGCCTGAGGATCAACCGGTAGTGGGTNNNNGGAAGGGGATTGCTTTTTCCTTGACCAATTCTTTCAAAAAACGAATCGGATTCATGGGTCATAATAAGATAATTCATGATTCAGATGCTTAATAATAAGATAATAAGAAGGAATAATCAAATTGAATTCATGAATTTACCCGGCAATTTATGGTCCAATAAAGGATTTTTATCTTCGAAACCCATTGGAAGGGGTAGTGCACGAGAAAAAAAATCATGCGGAAATGATCGACTCCTTGGATGCCCCAAAATACTATGAGTGTCGGAA

129_20180828_01_03_trnT(GUU)-comp-psbD-comp CATAGAGATGAAATTGGAACAAGTTGACCCCCTTTTCTATTTATTTTCATTTCTTTGGACTCCGCAAGAATTTGCCGATATTTCCAATTCAATCGTCTTGTCTTGTTCCTAGATGTTCTATAGGAAAAAATTGTCATTTCGTTCCTCTACGGAGAACCTTTTATTCTAAATTCTAAATCACAGGATCTAAATTCTAAATCACAGGATAAGAAAAAAATTCACTATCTTTCTTTGATTACAGGATCAAGATTCATTTANNNNTTATCTATACTATATAATATATTCNNNNNNTATAGATATTTCTATTCTATATTTATATTTATAGCTATCAGATCGTGGCTTGATGTACCAAAAATTTCCATTTCGTTGCATCCAATATTTTTGTTCCGACCATCGTATGAAGAAAGCATGCAAGATAAATACTATCATTTCCAATCTCCTATTTTAATTTTAATATTGTANNNNNNTTGAAGTGAAGTAAAAATTTGGAAACCCTCTCTTTTCTAACAGGGAAAAATAAATCAAAAAATATTAGTAATTTAGTCTACATAAAAATTAGAATATAAAGAGAGTTCTTTTTCTTAATCTCATGAACAAGATCTAAGAATCCATTTAGTTGATGAAAGAACATGGGGGGGGGCAGGCCTGAGGATCAACCGGTAGTGGGTNNNNGGAAGGGGATTGCTTTTTCCTTGACCAATTCTTTCAAAAAACGAATCGGATTCATGGGTCATAATAAGATAATTCATGATTCAGATGCTTAATAATAAGATAATAAGAAGGAATAATCAAATTGAATTCATGAATTTACCCGGCAATTTATGGTCCAATAAAGGATTTTTATCTTCGAAACCCATTGGAAGGGGTAGTGCACGAGAAAAAAAATCATGCGGAAATGATCGACTCCTTGGATGCCCCAAAATACTATGAGTGTCGGAA

130_20180828_02_01_trnT(GUU)-comp-psbD-comp CATAGAGATGAAATTGGAACAAGTTGACCCCCTTTTCTATTTATTTTCATTTCTTTGGACTCCGCAAGAATTTGCCGATATTTCCAATTCAATCGTCTTGTCTTGTTCCTAGATGTTCTATAGGAAAAAATTGTCATTTCGTTCCTCTACGGAGAACCTTTTATTCTAAATTCTAAATCACAGGATCTAAATTCTAAATCACAGGATAAGAAAAAAATTCACTATCTTTCTTTGATTACAGGATCAAGATTCATTTANNNNTTATCTATACTATATAATATATTCNNNNNNTATAGATATTTCTATTCTATATTTATATTTATAGCTATCAGATCGTGGCTTGATGTACCAAAAATTTCCATTTCGTTGCATCCAATATTTTTGTTCCGACCATCGTATGAAGAAAGCATGCAAGATAAATACTATCATTTCCAATCTCCTATTTTAATTTTAATATTGTANNNNNNTTGAAGTGAAGTAAAAATTTGGAAACCCTCTCTTTTCTAACAGGGAAAAATAAATCAAAAAATATTAGTAATTTAGTCTACATAAAAATTAGAATATAAAGAGAGTTCTTTTTCTTAATCTCATGAACAAGATCTAAGAATCCATTTAGTTGATGAAAGAACATGGGGGGGGGCAGGCCTGAGGATCAACCGGTAGTGGGTNNNNGGAAGGGGATTGCTTTTTCCTTGACCAATTCTTTCAAAAAACGAATCGGATTCATGGGTCATAATAAGATAATTCATGATTCAGATGCTTAATAATAAGATAATAAGAAGGAATAATCAAATTGAATTCATGAATTTACCCGGCAATTTATGGTCCAATAAAGGATTTTTATCTTCGAAACCCATTGGAAGGGGTAGTGCACGAGAAAAAAAATCATGCGGAAATGATCGACTCCTTGGATGCCCCAAAATACTATGAGTGTCGGAA

131_20180828_02_02_trnT(GUU)-comp-psbD-comp CATAGAGATGAAATTGGAACAAGTTGACCCCCTTTTCTATTTATTTTCATTTCTTTGGACTCCGCAAGAATTTGCCGATATTTCCAATTCAATCGTCTTGTCTTGTTCCTAGATGTTCTATAGGAAAAAATTGTCATTTCGTTCCTCTACGGAGAACCTTTTATTCTAAATTCTAAATCACAGGATCTAAATTCTAAATCACAGGATAAGAAAAAAATTCACTATCTTTCTTTGATTACAGGATCAAGATTCATTTANNNNTTATCTATACTATATAATATATTCNNNNNNTATAGATATTTCTATTCTATATTTATATTTATAGCTATCAGATCGTGGCTTGATGTACCAAAAATTTCCATTTCGTTGCATCCAATATTTTTGTTCCGACCATCGTATGAAGAAAGCATGCAAGATAAATACTATCATTTCCAATCTCCTATTTTAATTTTAATATTGTANNNNNNTTGAAGTGAAGTAAAAATTTGGAAACCCTCTCTTTTCTAACAGGGAAAAATAAATCAAAAAATATTAGTAATTTAGTCTACATAAAAATTAGAATATAAAGAGAGTTCTTTTTCTTAATCTCATGAACAAGATCTAAGAATCCATTTAGTTGATGAAAGAACATGGGGGGGGGCAGGCCTGAGGATCAACCGGTAGTGGGTNNNNGGAAGGGGATTGCTTTTTCCTTGACCAATTCTTTCAAAAAACGAATCGGATTCATGGGTCATAATAAGATAATTCATGATTCAGATGCTTAATAATAAGATAATAAGAAGGAATAATCAAATTGAATTCATGAATTTACCCGGCAATTTATGGTCCAATAAAGGATTTTTATCTTCGAAACCCATTGGAAGGGGTAGTGCACGAGAAAAAAAATCATGCGGAAATGATCGACTCCTTGGATGCCCCAAAATACTATGAGTGTCGGAA

132_20180828_02_03_trnT(GUU)-comp-psbD-comp CATAGAGATGAAATTGGAACAAGTTGACCCCCTTTTCTATTTATTTTCATTTCTTTGGACTCCGCAAGAATTTGCCGATATTTCCAATTCAATCGTCTTGTCTTGTTCCTAGATGTTCTATAGGAAAAAATTGTCATTTCGTTCCTCTACGGAGAACCTTTTATTCTAAATTCTAAATCACAGGATCTAAATTCTAAATCACAGGATAAGAAAAAAATTCACTATCTTTCTTTGATTACAGGATCAAGATTCATTTANNNNTTATCTATACTATATAATATATTCNNNNNNTATAGATATTTCTATTCTATATTTATATTTATAGCTATCAGATCGTGGCTTGATGTACCAAAAATTTCCATTTCGTTGCATCCAATATTTTTGTTCCGACCATCGTATGAAGAAAGCATGCAAGATAAATACTATCATTTCCAATCTCCTATTTTAATTTTAATATTGTANNNNNNTTGAAGTGAAGTAAAAATTTGGAAACCCTCTCTTTTCTAACAGGGAAAAATAAATCAAAAAATATTAGTAATTTAGTCTACATAAAAATTAGAATATAAAGAGAGTTCTTTTTCTTAATCTCATGAACAAGATCTAAGAATCCATTTAGTTGATGAAAGAACATGGGGGGGGGCAGGCCTGAGGATCAACCGGTAGTGGGTNNNNGGAAGGGGATTGCTTTTTCCTTGACCAATTCTTTCAAAAAACGAATCGGATTCATGGGTCATAATAAGATAATTCATGATTCAGATGCTTAATAATAAGATAATAAGAAGGAATAATCAAATTGAATTCATGAATTTACCCGGCAATTTATGGTCCAATAAAGGATTTTTATCTTCGAAACCCATTGGAAGGGGTAGTGCACGAGAAAAAAAATCATGCGGAAATGATCGACTCCTTGGATGCCCCAAAATACTATGAGTGTCGGAA

133_20190626_01_01_trnT(GUU)-comp-psbD-comp CATAGAGATGAAATTGGAACAAGTTGACCCCCTTTTCTATTTATTTTCATTTCTTTGGACTCCGCAAGAATTTGCCGATATTTCCAATTCAATCGTCTTGTCTTGTTCCTAGATGTTCTATAGGAAAAAATTGTCATTTCGTTCCTCTACGGAGAACCTTTTATTCTAAATTCTAAATCACAGGATCTAAATTCTAAATCACAGGATAAGAAAAAAATTCACTATCTTTCTTTGATTACAGGATCAAGATTCATTTANNNNTTATCTATACTATATAATATATTCNNNNNNTATAGATATTTCTATTCTATATTTATATTTATAGCTATCAGATCGTGGCTTGATGTACCAAAAATTTCCATTTCGTTGCATCCAATATTTTTGTTCCGACCATCGTATGAAGAAAGCATGCAAGATAAATACTATCATTTCCAATCTCCTATTTTAATTTTAATATTGTANNNNNNTTGAAGTGAAGTAAAAATTTGGAAACCCTCTCTTTTCTAACAGGGAAAAATAAATCAAAAAATATTAGTAATTTAGTCTACATAAAAATTAGAATATAAAGAGAGTTCTTTTTCTTAATCTCATGAACAAGATCTAAGAATCCATTTAGTTGATGAAAGAACATGGGGGGGGGCAGGCCTGAGGATCAACCGGTAGTGGGTNNNNGGAAGGGGATTGCTTTTTCCTTGACCAATTCTTTCAAAAAACGAATCGGATTCATGGGTCATAATAAGATAATTCATGATTCAGATGCTTAATAATAAGATAATAAGAAGGAATAATCAAATTGAATTCATGAATTTACCCGGCAATTTATGGTCCAATAAAGGATTTTTATCTTCGAAACCCATTGGAAGGGGTAGTGCACGAGAAAAAAAATCATGCGGAAATGATCGACTCCTTGGATGCCCCAAAATACTATGAGTGTCGGAA

134_20190626_01_02_trnT(GUU)-comp-psbD-comp CATAGAGATGAAATTGGAACAAGTTGACCCCCTTTTCTATTTATTTTCATTTCTTTGGACTCCGCAAGAATTTGCCGATATTTCCAATTCAATCGTCTTGTCTTGTTCCTAGATGTTCTATAGGAAAAAATTGTCATTTCGTTCCTCTACGGAGAACCTTTTATTCTAAATTCTAAATCACAGGATCTAAATTCTAAATCACAGGATAAGAAAAAAATTCACTATCTTTCTTTGATTACAGGATCAAGATTCATTTANNNNTTATCTATACTATATAATATATTCNNNNNNTATAGATATTTCTATTCTATATTTATATTTATAGCTATCAGATCGTGGCTTGATGTACCAAAAATTTCCATTTCGTTGCATCCAATATTTTTGTTCCGACCATCGTATGAAGAAAGCATGCAAGATAAATACTATCATTTCCAATCTCCTATTTTAATTTTAATATTGTANNNNNNTTGAAGTGAAGTAAAAATTTGGAAACCCTCTCTTTTCTAACAGGGAAAAATAAATCAAAAAATATTAGTAATTTAGTCTACATAAAAATTAGAATATAAAGAGAGTTCTTTTTCTTAATCTCATGAACAAGATCTAAGAATCCATTTAGTTGATGAAAGAACATGGGGGGGGGCAGGCCTGAGGATCAACCGGTAGTGGGTNNNNGGAAGGGGATTGCTTTTTCCTTGACCAATTCTTTCAAAAAACGAATCGGATTCATGGGTCATAATAAGATAATTCATGATTCAGATGCTTAATAATAAGATAATAAGAAGGAATAATCAAATTGAATTCATGAATTTACCCGGCAATTTATGGTCCAATAAAGGATTTTTATCTTCGAAACCCATTGGAAGGGGTAGTGCACGAGAAAAAAAATCATGCGGAAATGATCGACTCCTTGGATGCCCCAAAATACTATGAGTGTCGGAA

135_20190626_02_01_trnT(GUU)-comp-psbD-comp CATAGAGATGAAATTGGAACAAGTTGACCCCCTTTTCTATTTATTTTCATTTCTTTGGACTCCGCAAGAATTTGCCGATATTTCCAATTCAATCGTCTTGTCTTGTTCCTAGATGTTCTATAGGAAAAAATTGTCATTTCGTTCCTCTACGGAGAACCTTTTATTCTAAATTCTAAATCACAGGATCTAAATTCTAAATCACAGGATAAGAAAAAAATTCACTATCTTTCTTTGATTACAGGATCAAGATTCATTTANNNNTTATCTATACTATATAATATATTCNNNNNNTATAGATATTTCTATTCTATATTTATATTTATAGCTATCAGATCGTGGCTTGATGTACCAAAAATTTCCATTTCGTTGCATCCAATATTTTTGTTCCGACCATCGTATGAAGAAAGCATGCAAGATAAATACTATCATTTCCAATCTCCTATTTTAATTTTAATATTGTANNNNNNTTGAAGTGAAGTAAAAATTTGGAAACCCTCTCTTTTCTAACAGGGAAAAATAAATCAAAAAATATTAGTAATTTAGTCTACATAAAAATTAGAATATAAAGAGAGTTCTTTTTCTTAATCTCATGAACAAGATCTAAGAATCCATTTAGTTGATGAAAGAACATGGGGGGGGGCAGGCCTGAGGATCAACCGGTAGTGGGTNNNNGGAAGGGGATTGCTTTTTCCTTGACCAATTCTTTCAAAAAACGAATCGGATTCATGGGTCATAATAAGATAATTCATGATTCAGATGCTTAATAATAAGATAATAAGAAGGAATAATCAAATTGAATTCATGAATTTACCCGGCAATTTATGGTCCAATAAAGGATTTTTATCTTCGAAACCCATTGGAAGGGGTAGTGCACGAGAAAAAAAATCATGCGGAAATGATCGACTCCTTGGATGCCCCAAAATACTATGAGTGTCGGAA

136_20190626_03_01_trnT(GUU)-comp-psbD-comp CATAGAGATGAAATTGGAACAAGTTGACCCCCTTTTCTATTTATTTTCATTTCTTTGGACTCCGCAAGAATTTGCCGATATTTCCAATTCAATCGTCTTGTCTTGTTCCTAGATGTTCTATAGGAAAAAATTGTCATTTCGTTCCTCTACGGAGAACCTTTTATTCTAAATTCTAAATCACAGGATCTAAATTCTAAATCACAGGATAAGAAAAAAATTCACTATCTTTCTTTGATTACAGGATCAAGATTCATTTANNNNTTATCTATACTATATAATATATTCNNNNNNTATAGATATTTCTATTCTATATTTATATTTATAGCTATCAGATCGTGGCTTGATGTACCAAAAATTTCCATTTCGTTGCATCCAATATTTTTGTTCCGACCATCGTATGAAGAAAGCATGCAAGATAAATACTATCATTTCCAATCTCCTATTTTAATTTTAATATTGTANNNNNNTTGAAGTGAAGTAAAAATTTGGAAACCCTCTCTTTTCTAACAGGGAAAAATAAATCAAAAAATATTAGTAATTTAGTCTACATAAAAATTAGAATATAAAGAGAGTTCTTTTTCTTAATCTCATGAACAAGATCTAAGAATCCATTTAGTTGATGAAAGAACATGGGGGGGGGCAGGCCTGAGGATCAACCGGTAGTGGGTNNNNGGAAGGGGATTGCTTTTTCCTTGACCAATTCTTTCAAAAAACGAATCGGATTCATGGGTCATAATAAGATAATTCATGATTCAGATGCTTAATAATAAGATAATAAGAAGGAATAATCAAATTGAATTCATGAATTTACCCGGCAATTTATGGTCCAATAAAGGATTTTTATCTTCGAAACCCATTGGAAGGGGTAGTGCACGAGAAAAAAAATCATGCGGAAATGATCGACTCCTTGGATGCCCCAAAATACTATGAGTGTCGGAA

137_20190626_05_04_trnT(GUU)-comp-psbD-comp CATAGAGATGAAATTGGAACAAGTTGACCCCCTTTTCTATTTATTTTCATTTCTTTGGACTCCGCAAGAATTTGCCGATATTTCCAATTCAATCGTCTTGTCTTGTTCCTAGATGTTCTATAGGAAAAAATTGTCATTTCGTTCCTCTACGGAGAACCTTTTATTCTAAATTCTAAATCACAGGATCTAAATTCTAAATCACAGGATAAGAAAAAAATTCACTATCTTTCTTTGATTACAGGATCAAGATTCATTTANNNNTTATCTATACTATATAATATATTCNNNNNNTATAGATATTTCTATTCTATATTTATATTTATAGCTATCAGATCGTGGCTTGATGTACCAAAAATTTCCATTTCGTTGCATCCAATATTTTTGTTCCGACCATCGTATGAAGAAAGCATGCAAGATAAATACTATCATTTCCAATCTCCTATTTTAATTTTAATATTGTANNNNNNTTGAAGTGAAGTAAAAATTTGGAAACCCTCTCTTTTCTAACAGGGAAAAATAAATCAAAAAATATTAGTAATTTAGTCTACATAAAAATTAGAATATAAAGAGAGTTCTTTTTCTTAATCTCATGAACAAGATCTAAGAATCCATTTAGTTGATGAAAGAACATGGGGGGGGGCAGGCCTGAGGATCAACCGGTAGTGGGTNNNNGGAAGGGGATTGCTTTTTCCTTGACCAATTCTTTCAAAAAACGAATCGGATTCATGGGTCATAATAAGATAATTCATGATTCAGATGCTTAATAATAAGATAATAAGAAGGAATAATCAAATTGAATTCATGAATTTACCCGGCAATTTATGGTCCAATAAAGGATTTTTATCTTCGAAACCCATTGGAAGGGGTAGTGCACGAGAAAAAAAATCATGCGGAAATGATCGACTCCTTGGATGCCCCAAAATACTATGAGTGTCGGAA

138_20190626_07_01_trnT(GUU)-comp-psbD-comp CATAGAGATGAAATTGGAACAAGTTGACCCCCTTTTCTATTTATTTTCATTTCTTTGGACTCCGCAAGAATTTGCCGATATTTCCAATTCAATCGTCTTGTCTTGTTCCTAGATGTTCTATAGGAAAAAATTGTCATTTCGTTCCTCTACGGAGAACCTTTTATTCTAAATTCTAAATCACAGGATCTAAATTCTAAATCACAGGATAAGAAAAAAATTCACTATCTTTCTTTGATTACAGGATCAAGATTCATTTANNNNTTATCTATACTATATAATATATTCNNNNNNTATAGATATTTCTATTCTATATTTATATTTATAGCTATCAGATCGTGGCTTGATGTACCAAAAATTTCCATTTCGTTGCATCCAATATTTTTGTTCCGACCATCGTATGAAGAAAGCATGCAAGATAAATACTATCATTTCCAATCTCCTATTTTAATTTTAATATTGTANNNNNNTTGAAGTGAAGTAAAAATTTGGAAACCCTCTCTTTTCTAACAGGGAAAAATAAATCAAAAAATATTAGTAATTTAGTCTACATAAAAATTAGAATATAAAGAGAGTTCTTTTTCTTAATCTCATGAACAAGATCTAAGAATCCATTTAGTTGATGAAAGAACATGGGGGGGGGCAGGCCTGAGGATCAACCGGTAGTGGGTNNNNGGAAGGGGATTGCTTTTTCCTTGACCAATTCTTTCAAAAAACGAATCGGATTCATGGGTCATAATAAGATAATTCATGATTCAGATGCTTAATAATAAGATAATAAGAAGGAATAATCAAATTGAATTCATGAATTTACCCGGCAATTTATGGTCCAATAAAGGATTTTTATCTTCGAAACCCATTGGAAGGGGTAGTGCACGAGAAAAAAAATCATGCGGAAATGATCGACTCCTTGGATGCCCCAAAATACTATGAGTGTCGGAA

139_20190626_07_02_trnT(GUU)-comp-psbD-comp CATAGAGATGAAATTGGAACAAGTTGACCCCCTTTTCTATTTATTTTCATTTCTTTGGACTCCGCAAGAATTTGCCGATATTTCCAATTCAATCGTCTTGTCTTGTTCCTAGATGTTCTATAGGAAAAAATTGTCATTTCGTTCCTCTACGGAGAACCTTTTATTCTAAATTCTAAATCACAGGATCTAAATTCTAAATCACAGGATAAGAAAAAAATTCACTATCTTTCTTTGATTACAGGATCAAGATTCATTTANNNNTTATCTATACTATATAATATATTCNNNNNNTATAGATATTTCTATTCTATATTTATATTTATAGCTATCAGATCGTGGCTTGATGTACCAAAAATTTCCATTTCGTTGCATCCAATATTTTTGTTCCGACCATCGTATGAAGAAAGCATGCAAGATAAATACTATCATTTCCAATCTCCTATTTTAATTTTAATATTGTANNNNNNTTGAAGTGAAGTAAAAATTTGGAAACCCTCTCTTTTCTAACAGGGAAAAATAAATCAAAAAATATTAGTAATTTAGTCTACATAAAAATTAGAATATAAAGAGAGTTCTTTTTCTTAATCTCATGAACAAGATCTAAGAATCCATTTAGTTGATGAAAGAACATGGGGGGGGGCAGGCCTGAGGATCAACCGGTAGTGGGTNNNNGGAAGGGGATTGCTTTTTCCTTGACCAATTCTTTCAAAAAACGAATCGGATTCATGGGTCATAATAAGATAATTCATGATTCAGATGCTTAATAATAAGATAATAAGAAGGAATAATCAAATTGAATTCATGAATTTACCCGGCAATTTATGGTCCAATAAAGGATTTTTATCTTCGAAACCCATTGGAAGGGGTAGTGCACGAGAAAAAAAATCATGCGGAAATGATCGACTCCTTGGATGCCCCAAAATACTATGAGTGTCGGAA

140_20190626_08_01_trnT(GUU)-comp-psbD-comp CATAGAGATGAAATTGGAACAAGTTGACCCCCTTTTCTATTTATTTTCATTTCTTTGGACTCCGCAAGAATTTGCCGATATTTCCAATTCAATCGTCTTGTCTTGTTCCTAGATGTTCTATAGGAAAAAATTGTCATTTCGTTCCTCTACGGAGAACCTTTTATTCTAAATTCTAAATCACAGGATCTAAATTCTAAATCACAGGATAAGAAAAAAATTCACTATCTTTCTTTGATTACAGGATCAAGATTCATTTANNNNTTATCTATACTATATAATATATTCNNNNNNTATAGATATTTCTATTCTATATTTATATTTATAGCTATCAGATCGTGGCTTGATGTACCAAAAATTTCCATTTCGTTGCATCCAATATTTTTGTTCCGACCATCGTATGAAGAAAGCATGCAAGATAAATACTATCATTTCCAATCTCCTATTTTAATTTTAATATTGTANNNNNNTTGAAGTGAAGTAAAAATTTGGAAACCCTCTCTTTTCTAACAGGGAAAAATAAATCAAAAAATATTAGTAATTTAGTCTACATAAAAATTAGAATATAAAGAGAGTTCTTTTTCTTAATCTCATGAACAAGATCTAAGAATCCATTTAGTTGATGAAAGAACATGGGGGGGGGCAGGCCTGAGGATCAACCGGTAGTGGGTNNNNGGAAGGGGATTGCTTTTTCCTTGACCAATTCTTTCAAAAAACGAATCGGATTCATGGGTCATAATAAGATAATTCATGATTCAGATGCTTAATAATAAGATAATAAGAAGGAATAATCAAATTGAATTCATGAATTTACCCGGCAATTTATGGTCCAATAAAGGATTTTTATCTTCGAAACCCATTGGAAGGGGTAGTGCACGAGAAAAAAAATCATGCGGAAATGATCGACTCCTTGGATGCCCCAAAATACTATGAGTGTCGGAA

141_20190626_09_02_trnT(GUU)-comp-psbD-comp CATAGAGATGAAATTGGAACAAGTTGACCCCCTTTTCTATTTATTTTCATTTCTTTGGACTCCGCAAGAATTTGCCGATATTTCCAATTCAATCGTCTTGTCTTGTTCCTAGATGTTCTATAGGAAAAAATTGTCATTTCGTTCCTCTACGGAGAACCTTTTATTCTAAATTCTAAATCACAGGATCTAAATTCTAAATCACAGGATAAGAAAAAAATTCACTATCTTTCTTTGATTACAGGATCAAGATTCATTTANNNNTTATCTATACTATATAATATATTCNNNNNNTATAGATATTTCTATTCTATATTTATATTTATAGCTATCAGATCGTGGCTTGATGTACCAAAAATTTCCATTTCGTTGCATCCAATATTTTTGTTCCGACCATCGTATGAAGAAAGCATGCAAGATAAATACTATCATTTCCAATCTCCTATTTTAATTTTAATATTGTANNNNNNTTGAAGTGAAGTAAAAATTTGGAAACCCTCTCTTTTCTAACAGGGAAAAATAAATCAAAAAATATTAGTAATTTAGTCTACATAAAAATTAGAATATAAAGAGAGTTCTTTTTCTTAATCTCATGAACAAGATCTAAGAATCCATTTAGTTGATGAAAGAACATGGGGGGGGGCAGGCCTGAGGATCAACCGGTAGTGGGTNNNNGGAAGGGGATTGCTTTTTCCTTGACCAATTCTTTCAAAAAACGAATCGGATTCATGGGTCATAATAAGATAATTCATGATTCAGATGCTTAATAATAAGATAATAAGAAGGAATAATCAAATTGAATTCATGAATTTACCCGGCAATTTATGGTCCAATAAAGGATTTTTATCTTCGAAACCCATTGGAAGGGGTAGTGCACGAGAAAAAAAATCATGCGGAAATGATCGACTCCTTGGATGCCCCAAAATACTATGAGTGTCGGAA

142_20190627_01_01_trnT(GUU)-comp-psbD-comp CATAGAGATGAAATTGGAACAAGTTGACCCCCTTTTCTATTTATTTTCATTTCTTTGGACTCCGCAAGAATTTGCCGATATTTCCAATTCAATCGTCTTGTCTTGTTCCTAGATGTTCTATAGGAAAAAATTGTCATTTCGTTCCTCTACGGAGAACCTTTTATTCTAAATTCTAAATCACAGGATCTAAATTCTAAATCACAGGATAAGAAAAAAATTCACTATCTTTCTTTGATTACAGGATCAAGATTCATTTANNNNTTATCTATACTATATAATATATTCNNNNNNTATAGATATTTCTATTCTATATTTATATTTATAGCTATCAGATCGTGGCTTGATGTACCAAAAATTTCCATTTCGTTGCATCCAATATTTTTGTTCCGACCATCGTATGAAGAAAGCATGCAAGATAAATACTATCATTTCCAATCTCCTATTTTAATTTTAATATTGTANNNNNNTTGAAGTGAAGTAAAAATTTGGAAACCCTCTCTTTTCTAACAGGGAAAAATAAATCAAAAAATATTAGTAATTTAGTCTACATAAAAATTAGAATATAAAGAGAGTTCTTTTTCTTAATCTCATGAACAAGATCTAAGAATCCATTTAGTTGATGAAAGAACATGGGGGGGGGCAGGCCTGAGGATCAACCGGTAGTGGGTNNNNGGAAGGGGATTGCTTTTTCCTTGACCAATTCTTTCAAAAAACGAATCGGATTCATGGGTCATAATAAGATAATTCATGATTCAGATGCTTAATAATAAGATAATAAGAAGGAATAATCAAATTGAATTCATGAATTTACCCGGCAATTTATGGTCCAATAAAGGATTTTTATCTTCGAAACCCATTGGAAGGGGTAGTGCACGAGAAAAAAAATCATGCGGAAATGATCGACTCCTTGGATGCCCCAAAATACTATGAGTGTCGGAA

143_20190627_03_01_trnT(GUU)-comp-psbD-comp CATAGAGATGAAATTGGAACAAGTTGACCCCCTTTTCTATTTATTTTCATTTCTTTGGACTCCGCAAGAATTTGCCGATATTTCCAATTCAATCGTCTTGTCTTGTTCCTAGATGTTCTATAGGAAAAAATTGTCATTTCGTTCCTCTACGGAGAACCTTTTATTCTAAATTCTAAATCACAGGATCTAAATTCTAAATCACAGGATAAGAAAAAAATTCACTATCTTTCTTTGATTACAGGATCAAGATTCATTTANNNNTTATCTATACTATATAATATATTCNNNNNNTATAGATATTTCTATTCTATATTTATATTTATAGCTATCAGATCGTGGCTTGATGTACCAAAAATTTCCATTTCGTTGCATCCAATATTTTTGTTCCGACCATCGTATGAAGAAAGCATGCAAGATAAATACTATCATTTCCAATCTCCTATTTTAATTTTAATATTGTANNNNNNTTGAAGTGAAGTAAAAATTTGGAAACCCTCTCTTTTCTAACAGGGAAAAATAAATCAAAAAATATTAGTAATTTAGTCTACATAAAAATTAGAATATAAAGAGAGTTCTTTTTCTTAATCTCATGAACAAGATCTAAGAATCCATTTAGTTGATGAAAGAACATGGGGGGGGGCAGGCCTGAGGATCAACCGGTAGTGGGTNNNNGGAAGGGGATTGCTTTTTCCTTGACCAATTCTTTCAAAAAACGAATCGGATTCATGGGTCATAATAAGATAATTCATGATTCAGATGCTTAATAATAAGATAATAAGAAGGAATAATCAAATTGAATTCATGAATTTACCCGGCAATTTATGGTCCAATAAAGGATTTTTATCTTCGAAACCCATTGGAAGGGGTAGTGCACGAGAAAAAAAATCATGCGGAAATGATCGACTCCTTGGATGCCCCAAAATACTATGAGTGTCGGAA

144_20190627_03_02_trnT(GUU)-comp-psbD-comp CATAGAGATGAAATTGGAACAAGTTGACCCCCTTTTCTATTTATTTTCATTTCTTTGGACTCCGCAAGAATTTGCCGATATTTCCAATTCAATCGTCTTGTCTTGTTCCTAGATGTTCTATAGGAAAAAATTGTCATTTCGTTCCTCTACGGAGAACCTTTTATTCTAAATTCTAAATCACAGGATCTAAATTCTAAATCACAGGATAAGAAAAAAATTCACTATCTTTCTTTGATTACAGGATCAAGATTCATTTANNNNTTATCTATACTATATAATATATTCNNNNNNTATAGATATTTCTATTCTATATTTATATTTATAGCTATCAGATCGTGGCTTGATGTACCAAAAATTTCCATTTCGTTGCATCCAATATTTTTGTTCCGACCATCGTATGAAGAAAGCATGCAAGATAAATACTATCATTTCCAATCTCCTATTTTAATTTTAATATTGTANNNNNNTTGAAGTGAAGTAAAAATTTGGAAACCCTCTCTTTTCTAACAGGGAAAAATAAATCAAAAAATATTAGTAATTTAGTCTACATAAAAATTAGAATATAAAGAGAGTTCTTTTTCTTAATCTCATGAACAAGATCTAAGAATCCATTTAGTTGATGAAAGAACATGGGGGGGGGCAGGCCTGAGGATCAACCGGTAGTGGGTNNNNGGAAGGGGATTGCTTTTTCCTTGACCAATTCTTTCAAAAAACGAATCGGATTCATGGGTCATAATAAGATAATTCATGATTCAGATGCTTAATAATAAGATAATAAGAAGGAATAATCAAATTGAATTCATGAATTTACCCGGCAATTTATGGTCCAATAAAGGATTTTTATCTTCGAAACCCATTGGAAGGGGTAGTGCACGAGAAAAAAAATCATGCGGAAATGATCGACTCCTTGGATGCCCCAAAATACTATGAGTGTCGGAA

145_20190627_04_01_trnT(GUU)-comp-psbD-comp CATAGAGATGAAATTGGAACAAGTTGACCCCCTTTTCTATTTATTTTCATTTCTTTGGACTCCGCAAGAATTTGCCGATATTTCCAATTCAATCGTCTTGTCTTGTTCCTAGATGTTCTATAGGAAAAAATTGTCATTTCGTTCCTCTACGGAGAACCTTTTATTCTAAATTCTAAATCACAGGATCTAAATTCTAAATCACAGGATAAGAAAAAAATTCACTATCTTTCTTTGATTACAGGATCAAGATTCATTTANNNNTTATCTATACTATATAATATATTCNNNNNNTATAGATATTTCTATTCTATATTTATATTTATAGCTATCAGATCGTGGCTTGATGTACCAAAAATTTCCATTTCGTTGCATCCAATATTTTTGTTCCGACCATCGTATGAAGAAAGCATGCAAGATAAATACTATCATTTCCAATCTCCTATTTTAATTTTAATATTGTANNNNNNTTGAAGTGAAGTAAAAATTTGGAAACCCTCTCTTTTCTAACAGGGAAAAATAAATCAAAAAATATTAGTAATTTAGTCTACATAAAAATTAGAATATAAAGAGAGTTCTTTTTCTTAATCTCATGAACAAGATCTAAGAATCCATTTAGTTGATGAAAGAACATGGGGGGGGGCAGGCCTGAGGATCAACCGGTAGTGGGTNNNNGGAAGGGGATTGCTTTTTCCTTGACCAATTCTTTCAAAAAACGAATCGGATTCATGGGTCATAATAAGATAATTCATGATTCAGATGCTTAATAATAAGATAATAAGAAGGAATAATCAAATTGAATTCATGAATTTACCCGGCAATTTATGGTCCAATAAAGGATTTTTATCTTCGAAACCCATTGGAAGGGGTAGTGCACGAGAAAAAAAATCATGCGGAAATGATCGACTCCTTGGATGCCCCAAAATACTATGAGTGTCGGAA

146_20190627_04_02_trnT(GUU)-comp-psbD-comp CATAGAGATGAAATTGGAACAAGTTGACCCCCTTTTCTATTTATTTTCATTTCTTTGGACTCCGCAAGAATTTGCCGATATTTCCAATTCAATCGTCTTGTCTTGTTCCTAGATGTTCTATAGGAAAAAATTGTCATTTCGTTCCTCTACGGAGAACCTTTTATTCTAAATTCTAAATCACAGGATCTAAATTCTAAATCACAGGATAAGAAAAAAATTCACTATCTTTCTTTGATTACAGGATCAAGATTCATTTANNNNTTATCTATACTATATAATATATTCNNNNNNTATAGATATTTCTATTCTATATTTATATTTATAGCTATCAGATCGTGGCTTGATGTACCAAAAATTTCCATTTCGTTGCATCCAATATTTTTGTTCCGACCATCGTATGAAGAAAGCATGCAAGATAAATACTATCATTTCCAATCTCCTATTTTAATTTTAATATTGTANNNNNNTTGAAGTGAAGTAAAAATTTGGAAACCCTCTCTTTTCTAACAGGGAAAAATAAATCAAAAAATATTAGTAATTTAGTCTACATAAAAATTAGAATATAAAGAGAGTTCTTTTTCTTAATCTCATGAACAAGATCTAAGAATCCATTTAGTTGATGAAAGAACATGGGGGGGGGCAGGCCTGAGGATCAACCGGTAGTGGGTNNNNGGAAGGGGATTGCTTTTTCCTTGACCAATTCTTTCAAAAAACGAATCGGATTCATGGGTCATAATAAGATAATTCATGATTCAGATGCTTAATAATAAGATAATAAGAAGGAATAATCAAATTGAATTCATGAATTTACCCGGCAATTTATGGTCCAATAAAGGATTTTTATCTTCGAAACCCATTGGAAGGGGTAGTGCACGAGAAAAAAAATCATGCGGAAATGATCGACTCCTTGGATGCCCCAAAATACTATGAGTGTCGGAA

147_20190627_06_01_trnT(GUU)-comp-psbD-comp CATAGAGATGAAATTGGAACAAGTTGACCCCCTTTTCTATTTATTTTCATTTCTTTGGACTCCGCAAGAATTTGCCGATATTTCCAATTCAATCGTCTTGTCTTGTTCCTAGATGTTCTATAGGAAAAAATTGTCATTTCGTTCCTCTACGGAGAACCTTTTATTCTAAATTCTAAATCACAGGATCTAAATTCTAAATCACAGGATAAGAAAAAAATTCACTATCTTTCTTTGATTACAGGATCAAGATTCATTTANNNNTTATCTATACTATATAATATATTCNNNNNNTATAGATATTTCTATTCTATATTTATATTTATAGCTATCAGATCGTGGCTTGATGTACCAAAAATTTCCATTTCGTTGCATCCAATATTTTTGTTCCGACCATCGTATGAAGAAAGCATGCAAGATAAATACTATCATTTCCAATCTCCTATTTTAATTTTAATATTGTANNNNNNTTGAAGTGAAGTAAAAATTTGGAAACCCTCTCTTTTCTAACAGGGAAAAATAAATCAAAAAATATTAGTAATTTAGTCTACATAAAAATTAGAATATAAAGAGAGTTCTTTTTCTTAATCTCATGAACAAGATCTAAGAATCCATTTAGTTGATGAAAGAACATGGGGGGGGGCAGGCCTGAGGATCAACCGGTAGTGGGTNNNNGGAAGGGGATTGCTTTTTCCTTGACCAATTCTTTCAAAAAACGAATCGGATTCATGGGTCATAATAAGATAATTCATGATTCAGATGCTTAATAATAAGATAATAAGAAGGAATAATCAAATTGAATTCATGAATTTACCCGGCAATTTATGGTCCAATAAAGGATTTTTATCTTCGAAACCCATTGGAAGGGGTAGTGCACGAGAAAAAAAATCATGCGGAAATGATCGACTCCTTGGATGCCCCAAAATACTATGAGTGTCGGAA

148_20190627_06_02_trnT(GUU)-comp-psbD-comp CATAGAGATGAAATTGGAACAAGTTGACCCCCTTTTCTATTTATTTTCATTTCTTTGGACTCCGCAAGAATTTGCCGATATTTCCAATTCAATCGTCTTGTCTTGTTCCTAGATGTTCTATAGGAAAAAATTGTCATTTCGTTCCTCTACGGAGAACCTTTTATTCTAAATTCTAAATCACAGGATCTAAATTCTAAATCACAGGATAAGAAAAAAATTCACTATCTTTCTTTGATTACAGGATCAAGATTCATTTANNNNTTATCTATACTATATAATATATTCNNNNNNTATAGATATTTCTATTCTATATTTATATTTATAGCTATCAGATCGTGGCTTGATGTACCAAAAATTTCCATTTCGTTGCATCCAATATTTTTGTTCCGACCATCGTATGAAGAAAGCATGCAAGATAAATACTATCATTTCCAATCTCCTATTTTAATTTTAATATTGTANNNNNNTTGAAGTGAAGTAAAAATTTGGAAACCCTCTCTTTTCTAACAGGGAAAAATAAATCAAAAAATATTAGTAATTTAGTCTACATAAAAATTAGAATATAAAGAGAGTTCTTTTTCTTAATCTCATGAACAAGATCTAAGAATCCATTTAGTTGATGAAAGAACATGGGGGGGGGCAGGCCTGAGGATCAACCGGTAGTGGGTNNNNGGAAGGGGATTGCTTTTTCCTTGACCAATTCTTTCAAAAAACGAATCGGATTCATGGGTCATAATAAGATAATTCATGATTCAGATGCTTAATAATAAGATAATAAGAAGGAATAATCAAATTGAATTCATGAATTTACCCGGCAATTTATGGTCCAATAAAGGATTTTTATCTTCGAAACCCATTGGAAGGGGTAGTGCACGAGAAAAAAAATCATGCGGAAATGATCGACTCCTTGGATGCCCCAAAATACTATGAGTGTCGGAA

149_20190627_07_01_trnT(GUU)-comp-psbD-comp CATAGAGATGAAATTGGAACAAGTTGACCCCCTTTTCTATTTATTTTCATTTCTTTGGACTCCGCAAGAATTTGCCGATATTTCCAATTCAATCGTCTTGTCTTGTTCCTAGATGTTCTATAGGAAAAAATTGTCATTTCGTTCCTCTACGGAGAACCTTTTATTCTAAATTCTAAATCACAGGATCTAAATTCTAAATCACAGGATAAGAAAAAAATTCACTATCTTTCTTTGATTACAGGATCAAGATTCATTTANNNNTTATCTATACTATATAATATATTCNNNNNNTATAGATATTTCTATTCTATATTTATATTTATAGCTATCAGATCGTGGCTTGATGTACCAAAAATTTCCATTTCGTTGCATCCAATATTTTTGTTCCGACCATCGTATGAAGAAAGCATGCAAGATAAATACTATCATTTCCAATCTCCTATTTTAATTTTAATATTGTANNNNNNTTGAAGTGAAGTAAAAATTTGGAAACCCTCTCTTTTCTAACAGGGAAAAATAAATCAAAAAATATTAGTAATTTAGTCTACATAAAAATTAGAATATAAAGAGAGTTCTTTTTCTTAATCTCATGAACAAGATCTAAGAATCCATTTAGTTGATGAAAGAACATGGGGGGGGGCAGGCCTGAGGATCAACCGGTAGTGGGTNNNNGGAAGGGGATTGCTTTTTCCTTGACCAATTCTTTCAAAAAACGAATCGGATTCATGGGTCATAATAAGATAATTCATGATTCAGATGCTTAATAATAAGATAATAAGAAGGAATAATCAAATTGAATTCATGAATTTACCCGGCAATTTATGGTCCAATAAAGGATTTTTATCTTCGAAACCCATTGGAAGGGGTAGTGCACGAGAAAAAAAATCATGCGGAAATGATCGACTCCTTGGATGCCCCAAAATACTATGAGTGTCGGAA

150_20190627_07_02_trnT(GUU)-comp-psbD-comp CATAGAGATGAAATTGGAACAAGTTGACCCCCTTTTCTATTTATTTTCATTTCTTTGGACTCCGCAAGAATTTGCCGATATTTCCAATTCAATCGTCTTGTCTTGTTCCTAGATGTTCTATAGGAAAAAATTGTCATTTCGTTCCTCTACGGAGAACCTTTTATTCTAAATTCTAAATCACAGGATCTAAATTCTAAATCACAGGATAAGAAAAAAATTCACTATCTTTCTTTGATTACAGGATCAAGATTCATTTANNNNTTATCTATACTATATAATATATTCNNNNNNTATAGATATTTCTATTCTATATTTATATTTATAGCTATCAGATCGTGGCTTGATGTACCAAAAATTTCCATTTCGTTGCATCCAATATTTTTGTTCCGACCATCGTATGAAGAAAGCATGCAAGATAAATACTATCATTTCCAATCTCCTATTTTAATTTTAATATTGTANNNNNNTTGAAGTGAAGTAAAAATTTGGAAACCCTCTCTTTTCTAACAGGGAAAAATAAATCAAAAAATATTAGTAATTTAGTCTACATAAAAATTAGAATATAAAGAGAGTTCTTTTTCTTAATCTCATGAACAAGATCTAAGAATCCATTTAGTTGATGAAAGAACATGGGGGGGGGCAGGCCTGAGGATCAACCGGTAGTGGGTNNNNGGAAGGGGATTGCTTTTTCCTTGACCAATTCTTTCAAAAAACGAATCGGATTCATGGGTCATAATAAGATAATTCATGATTCAGATGCTTAATAATAAGATAATAAGAAGGAATAATCAAATTGAATTCATGAATTTACCCGGCAATTTATGGTCCAATAAAGGATTTTTATCTTCGAAACCCATTGGAAGGGGTAGTGCACGAGAAAAAAAATCATGCGGAAATGATCGACTCCTTGGATGCCCCAAAATACTATGAGTGTCGGAA

151_20190627_08_01_trnT(GUU)-comp-psbD-comp CATAGAGATGAAATTGGAACAAGTTGACCCCCTTTTCTATTTATTTTCATTTCTTTGGACTCCGCAAGAATTTGCCGATATTTCCAATTCAATCGTCTTGTCTTGTTCCTAGATGTTCTATAGGAAAAAATTGTCATTTCGTTCCTCTACGGAGAACCTTTTATTCTAAATTCTAAATCACAGGATCTAAATTCTAAATCACAGGATAAGAAAAAAATTCACTATCTTTCTTTGATTACAGGATCAAGATTCATTTANNNNTTATCTATACTATATAATATATTCNNNNNNTATAGATATTTCTATTCTATATTTATATTTATAGCTATCAGATCGTGGCTTGATGTACCAAAAATTTCCATTTCGTTGCATCCAATATTTTTGTTCCGACCATCGTATGAAGAAAGCATGCAAGATAAATACTATCATTTCCAATCTCCTATTTTAATTTTAATATTGTANNNNNNTTGAAGTGAAGTAAAAATTTGGAAACCCTCTCTTTTCTAACAGGGAAAAATAAATCAAAAAATATTAGTAATTTAGTCTACATAAAAATTAGAATATAAAGAGAGTTCTTTTTCTTAATCTCATGAACAAGATCTAAGAATCCATTTAGTTGATGAAAGAACATGGGGGGGGGCAGGCCTGAGGATCAACCGGTAGTGGGTNNNNGGAAGGGGATTGCTTTTTCCTTGACCAATTCTTTCAAAAAACGAATCGGATTCATGGGTCATAATAAGATAATTCATGATTCAGATGCTTAATAATAAGATAATAAGAAGGAATAATCAAATTGAATTCATGAATTTACCCGGCAATTTATGGTCCAATAAAGGATTTTTATCTTCGAAACCCATTGGAAGGGGTAGTGCACGAGAAAAAAAATCATGCGGAAATGATCGACTCCTTGGATGCCCCAAAATACTATGAGTGTCGGAA

152_20190627_08_02_trnT(GUU)-comp-psbD-comp CATAGAGATGAAATTGGAACAAGTTGACCCCCTTTTCTATTTATTTTCATTTCTTTGGACTCCGCAAGAATTTGCCGATATTTCCAATTCAATCGTCTTGTCTTGTTCCTAGATGTTCTATAGGAAAAAATTGTCATTTCGTTCCTCTACGGAGAACCTTTTATTCTAAATTCTAAATCACAGGATCTAAATTCTAAATCACAGGATAAGAAAAAAATTCACTATCTTTCTTTGATTACAGGATCAAGATTCATTTANNNNTTATCTATACTATATAATATATTCNNNNNNTATAGATATTTCTATTCTATATTTATATTTATAGCTATCAGATCGTGGCTTGATGTACCAAAAATTTCCATTTCGTTGCATCCAATATTTTTGTTCCGACCATCGTATGAAGAAAGCATGCAAGATAAATACTATCATTTCCAATCTCCTATTTTAATTTTAATATTGTANNNNNNTTGAAGTGAAGTAAAAATTTGGAAACCCTCTCTTTTCTAACAGGGAAAAATAAATCAAAAAATATTAGTAATTTAGTCTACATAAAAATTAGAATATAAAGAGAGTTCTTTTTCTTAATCTCATGAACAAGATCTAAGAATCCATTTAGTTGATGAAAGAACATGGGGGGGGGCAGGCCTGAGGATCAACCGGTAGTGGGTNNNNGGAAGGGGATTGCTTTTTCCTTGACCAATTCTTTCAAAAAACGAATCGGATTCATGGGTCATAATAAGATAATTCATGATTCAGATGCTTAATAATAAGATAATAAGAAGGAATAATCAAATTGAATTCATGAATTTACCCGGCAATTTATGGTCCAATAAAGGATTTTTATCTTCGAAACCCATTGGAAGGGGTAGTGCACGAGAAAAAAAATCATGCGGAAATGATCGACTCCTTGGATGCCCCAAAATACTATGAGTGTCGGAA

153_20190627_09_01_trnT(GUU)-comp-psbD-comp CATAGAGATGAAATTGGAACAAGTTGACCCCCTTTTCTATTTATTTTCATTTCTTTGGACTCCGCAAGAATTTGCCGATATTTCCAATTCAATCGTCTTGTCTTGTTCCTAGATGTTCTATAGGAAAAAATTGTCATTTCGTTCCTCTACGGAGAACCTTTTATTCTAAATTCTAAATCACAGGATCTAAATTCTAAATCACAGGATAAGAAAAAAATTCACTATCTTTCTTTGATTACAGGATCAAGATTCATTTANNNNTTATCTATACTATATAATATATTCNNNNNNTATAGATATTTCTATTCTATATTTATATTTATAGCTATCAGATCGTGGCTTGATGTACCAAAAATTTCCATTTCGTTGCATCCAATATTTTTGTTCCGACCATCGTATGAAGAAAGCATGCAAGATAAATACTATCATTTCCAATCTCCTATTTTAATTTTAATATTGTANNNNNNTTGAAGTGAAGTAAAAATTTGGAAACCCTCTCTTTTCTAACAGGGAAAAATAAATCAAAAAATATTAGTAATTTAGTCTACATAAAAATTAGAATATAAAGAGAGTTCTTTTTCTTAATCTCATGAACAAGATCTAAGAATCCATTTAGTTGATGAAAGAACATGGGGGGGGGCAGGCCTGAGGATCAACCGGTAGTGGGTNNNNGGAAGGGGATTGCTTTTTCCTTGACCAATTCTTTCAAAAAACGAATCGGATTCATGGGTCATAATAAGATAATTCATGATTCAGATGCTTAATAATAAGATAATAAGAAGGAATAATCAAATTGAATTCATGAATTTACCCGGCAATTTATGGTCCAATAAAGGATTTTTATCTTCGAAACCCATTGGAAGGGGTAGTGCACGAGAAAAAAAATCATGCGGAAATGATCGACTCCTTGGATGCCCCAAAATACTATGAGTGTCGGAA

154_20190627_09_02_trnT(GUU)-comp-psbD-comp CATAGAGATGAAATTGGAACAAGTTGACCCCCTTTTCTATTTATTTTCATTTCTTTGGACTCCGCAAGAATTTGCCGATATTTCCAATTCAATCGTCTTGTCTTGTTCCTAGATGTTCTATAGGAAAAAATTGTCATTTCGTTCCTCTACGGAGAACCTTTTATTCTAAATTCTAAATCACAGGATCTAAATTCTAAATCACAGGATAAGAAAAAAATTCACTATCTTTCTTTGATTACAGGATCAAGATTCATTTANNNNTTATCTATACTATATAATATATTCNNNNNNTATAGATATTTCTATTCTATATTTATATTTATAGCTATCAGATCGTGGCTTGATGTACCAAAAATTTCCATTTCGTTGCATCCAATATTTTTGTTCCGACCATCGTATGAAGAAAGCATGCAAGATAAATACTATCATTTCCAATCTCCTATTTTAATTTTAATATTGTANNNNNNTTGAAGTGAAGTAAAAATTTGGAAACCCTCTCTTTTCTAACAGGGAAAAATAAATCAAAAAATATTAGTAATTTAGTCTACATAAAAATTAGAATATAAAGAGAGTTCTTTTTCTTAATCTCATGAACAAGATCTAAGAATCCATTTAGTTGATGAAAGAACATGGGGGGGGGCAGGCCTGAGGATCAACCGGTAGTGGGTNNNNGGAAGGGGATTGCTTTTTCCTTGACCAATTCTTTCAAAAAACGAATCGGATTCATGGGTCATAATAAGATAATTCATGATTCAGATGCTTAATAATAAGATAATAAGAAGGAATAATCAAATTGAATTCATGAATTTACCCGGCAATTTATGGTCCAATAAAGGATTTTTATCTTCGAAACCCATTGGAAGGGGTAGTGCACGAGAAAAAAAATCATGCGGAAATGATCGACTCCTTGGATGCCCCAAAATACTATGAGTGTCGGAA

155_20190629_01_01_trnT(GUU)-comp-psbD-comp CATAGAGATGAAATTGGAACAAGTTGACCCCCTTTTCTATTTATTTTCATTTCTTTGGACTCCGCAAGAATTTGCCGATATTTCCAATTCAATCGTCTTGTCTTGTTCCTAGATGTTCTATAGGAAAAAATTGTCATTTCGTTCCTCTACGGAGAACCTTTTATTCTAAATTCTAAATCACAGGATCTAAATTCTAAATCACAGGATAAGAAAAAAATTCACTATCTTTCTTTGATTACAGGATCAAGATTCATTTANNNNTTATCTATACTATATAATATATTCNNNNNNTATAGATATTTCTATTCTATATTTATATTTATAGCTATCAGATCGTGGCTTGATGTACCAAAAATTTCCATTTCGTTGCATCCAATATTTTTGTTCCGACCATCGTATGAAGAAAGCATGCAAGATAAATACTATCATTTCCAATCTCCTATTTTAATTTTAATATTGTANNNNNNTTGAAGTGAAGTAAAAATTTGGAAACCCTCTCTTTTCTAACAGGGAAAAATAAATCAAAAAATATTAGTAATTTAGTCTACATAAAAATTAGAATATAAAGAGAGTTCTTTTTCTTAATCTCATGAACAAGATCTAAGAATCCATTTAGTTGATGAAAGAACATGGGGGGGGGCAGGCCTGAGGATCAACCGGTAGTGGGTNNNNGGAAGGGGATTGCTTTTTCCTTGACCAATTCTTTCAAAAAACGAATCGGATTCATGGGTCATAATAAGATAATTCATGATTCAGATGCTTAATAATAAGATAATAAGAAGGAATAATCAAATTGAATTCATGAATTTACCCGGCAATTTATGGTCCAATAAAGGATTTTTATCTTCGAAACCCATTGGAAGGGGTAGTGCACGAGAAAAAAAATCATGCGGAAATGATCGACTCCTTGGATGCCCCAAAATACTATGAGTGTCGGAA

156_20190629_01_02_trnT(GUU)-comp-psbD-comp CATAGAGATGAAATTGGAACAAGTTGACCCCCTTTTCTATTTATTTTCATTTCTTTGGACTCCGCAAGAATTTGCCGATATTTCCAATTCAATCGTCTTGTCTTGTTCCTAGATGTTCTATAGGAAAAAATTGTCATTTCGTTCCTCTACGGAGAACCTTTTATTCTAAATTCTAAATCACAGGATCTAAATTCTAAATCACAGGATAAGAAAAAAATTCACTATCTTTCTTTGATTACAGGATCAAGATTCATTTANNNNTTATCTATACTATATAATATATTCNNNNNNTATAGATATTTCTATTCTATATTTATATTTATAGCTATCAGATCGTGGCTTGATGTACCAAAAATTTCCATTTCGTTGCATCCAATATTTTTGTTCCGACCATCGTATGAAGAAAGCATGCAAGATAAATACTATCATTTCCAATCTCCTATTTTAATTTTAATATTGTANNNNNNTTGAAGTGAAGTAAAAATTTGGAAACCCTCTCTTTTCTAACAGGGAAAAATAAATCAAAAAATATTAGTAATTTAGTCTACATAAAAATTAGAATATAAAGAGAGTTCTTTTTCTTAATCTCATGAACAAGATCTAAGAATCCATTTAGTTGATGAAAGAACATGGGGGGGGGCAGGCCTGAGGATCAACCGGTAGTGGGTNNNNGGAAGGGGATTGCTTTTTCCTTGACCAATTCTTTCAAAAAACGAATCGGATTCATGGGTCATAATAAGATAATTCATGATTCAGATGCTTAATAATAAGATAATAAGAAGGAATAATCAAATTGAATTCATGAATTTACCCGGCAATTTATGGTCCAATAAAGGATTTTTATCTTCGAAACCCATTGGAAGGGGTAGTGCACGAGAAAAAAAATCATGCGGAAATGATCGACTCCTTGGATGCCCCAGAATACTATGAGTGTCGGAA

157_20190629_02_01_trnT(GUU)-comp-psbD-comp CATAGAGATGAAATTGGAACAAGTTGACCCCCTTTTCTATTTATTTTCATTTCTTTGGACTCCGCAAGAATTTGCCGATATTTCCAATTCAATCGTCTTGTCTTGTTCCTAGATGTTCTATAGGAAAAAATTGTCATTTCGTTCCTCTACGGAGAACCTTTTATTCTAAATTCTAAATCACAGGATCTAAATTCTAAATCACAGGATAAGAAAAAAATTCACTATCTTTCTTTGATTACAGGATCAAGATTCATTTANNNNTTATCTATACTATATAATATATTCNNNNNNTATAGATATTTCTATTCTATATTTATATTTATAGCTATCAGATCGTGGCTTGATGTACCAAAAATTTCCATTTCGTTGCATCCAATATTTTTGTTCCGACCATCGTATGAAGAAAGCATGCAAGATAAATACTATCATTTCCAATCTCCTATTTTAATTTTAATATTGTANNNNNNTTGAAGTGAAGTAAAAATTTGGAAACCCTCTCTTTTCTAACAGGGAAAAATAAATCAAAAAATATTAGTAATTTAGTCTACATAAAAATTAGAATATAAAGAGAGTTCTTTTTCTTAATCTCATGAACAAGATCTAAGAATCCATTTAGTTGATGAAAGAACATGGGGGGGGGCAGGCCTGAGGATCAACCGGTAGTGGGTNNNNGGAAGGGGATTGCTTTTTCCTTGACCAATTCTTTCAAAAAACGAATCGGATTCATGGGTCATAATAAGATAATTCATGATTCAGATGCTTAATAATAAGATAATAAGAAGGAATAATCAAATTGAATTCATGAATTTACCCGGCAATTTATGGTCCAATAAAGGATTTTTATCTTCGAAACCCATTGGAAGGGGTAGTGCACGAGAAAAAAAATCATGCGGAAATGATCGACTCCTTGGATGCCCCAAAATACTATGAGTGTCGGAA

158_20190629_02_02_trnT(GUU)-comp-psbD-comp CATAGAGATGAAATTGGAACAAGTTGACCCCCTTTTCTATTTATTTTCATTTCTTTGGACTCCGCAAGAATTTGCCGATATTTCCAATTCAATCGTCTTGTCTTGTTCCTAGATGTTCTATAGGAAAAAATTGTCATTTCGTTCCTCTACGGAGAACCTTTTATTCTAAATTCTAAATCACAGGATCTAAATTCTAAATCACAGGATAAGAAAAAAATTCACTATCTTTCTTTGATTACAGGATCAAGATTCATTTANNNNTTATCTATACTATATAATATATTCNNNNNNTATAGATATTTCTATTCTATATTTATATTTATAGCTATCAGATCGTGGCTTGATGTACCAAAAATTTCCATTTCGTTGCATCCAATATTTTTGTTCCGACCATCGTATGAAGAAAGCATGCAAGATAAATACTATCATTTCCAATCTCCTATTTTAATTTTAATATTGTANNNNNNTTGAAGTGAAGTAAAAATTTGGAAACCCTCTCTTTTCTAACAGGGAAAAATAAATCAAAAAATATTAGTAATTTAGTCTACATAAAAATTAGAATATAAAGAGAGTTCTTTTTCTTAATCTCATGAACAAGATCTAAGAATCCATTTAGTTGATGAAAGAACATGGGGGGGGGCAGGCCTGAGGATCAACCGGTAGTGGGTNNNNGGAAGGGGATTGCTTTTTCCTTGACCAATTCTTTCAAAAAACGAATCGGATTCATGGGTCATAATAAGATAATTCATGATTCAGATGCTTAATAATAAGATAATAAGAAGGAATAATCAAATTGAATTCATGAATTTACCCGGCAATTTATGGTCCAATAAAGGATTTTTATCTTCGAAACCCATTGGAAGGGGTAGTGCACGAGAAAAAAAATCATGCGGAAATGATCGACTCCTTGGATGCCCCAAAATACTATGAGTGTCGGAA

159_20190629_04_01_trnT(GUU)-comp-psbD-comp CATAGAGATGAAATTGGAACAAGTTGACCCCCTTTTCTATTTATTTTCATTTCTTTGGACTCCGCAAGAATTTGCCGATATTTCCAATTCAATCGTCTTGTCTTGTTCCTAGATGTTCTATAGGAAAAAATTGTCATTTCGTTCCTCTACGGAGAACCTTTTATTCTAAATTCTAAATCACAGGATCTAAATTCTAAATCACAGGATAAGAAAAAAATTCACTATCTTTCTTTGATTACAGGATCAAGATTCATTTANNNNTTATCTATACTATATAATATATTCNNNNNNTATAGATATTTCTATTCTATATTTATATTTATAGCTATCAGATCGTGGCTTGATGTACCAAAAATTTCCATTTCGTTGCATCCAATATTTTTGTTCCGACCATCGTATGAAGAAAGCATGCAAGATAAATACTATCATTTCCAATCTCCTATTTTAATTTTAATATTGTANNNNNNTTGAAGTGAAGTAAAAATTTGGAAACCCTCTCTTTTCTAACAGGGAAAAATAAATCAAAAAATATTAGTAATTTAGTCTACATAAAAATTAGAATATAAAGAGAGTTCTTTTTCTTAATCTCATGAACAAGATCTAAGAATCCATTTAGTTGATGAAAGAACATGGGGGGGGGCAGGCCTGAGGATCAACCGGTAGTGGGTNNNNGGAAGGGGATTGCTTTTTCCTTGACCAATTCTTTCAAAAAACGAATCGGATTCATGGGTCATAATAAGATAATTCATGATTCAGATGCTTAATAATAAGATAATAAGAAGGAATAATCAAATTGAATTCATGAATTTACCCGGCAATTTATGGTCCAATAAAGGATTTTTATCTTCGAAACCCATTGGAAGGGGTAGTGCACGAGAAAAAAAATCATGCGGAAATGATCGACTCCTTGGATGCCCCAAAATACTATGAGTGTCGGAA

160_20190629_04_02_trnT(GUU)-comp-psbD-comp CATAGAGATGAAATTGGAACAAGTTGACCCCCTTTTCTATTTATTTTCATTTCTTTGGACTCCGCAAGAATTTGCCGATATTTCCAATTCAATCGTCTTGTCTTGTTCCTAGATGTTCTATAGGAAAAAATTGTCATTTCGTTCCTCTACGGAGAACCTTTTATTCTAAATTCTAAATCACAGGATCTAAATTCTAAATCACAGGATAAGAAAAAAATTCACTATCTTTCTTTGATTACAGGATCAAGATTCATTTANNNNTTATCTATACTATATAATATATTCNNNNNNTATAGATATTTCTATTCTATATTTATATTTATAGCTATCAGATCGTGGCTTGATGTACCAAAAATTTCCATTTCGTTGCATCCAATATTTTTGTTCCGACCATCGTATGAAGAAAGCATGCAAGATAAATACTATCATTTCCAATCTCCTATTTTAATTTTAATATTGTANNNNNNTTGAAGTGAAGTAAAAATTTGGAAACCCTCTCTTTTCTAACAGGGAAAAATAAATCAAAAAATATTAGTAATTTAGTCTACATAAAAATTAGAATATAAAGAGAGTTCTTTTTCTTAATCTCATGAACAAGATCTAAGAATCCATTTAGTTGATGAAAGAACATGGGGGGGGGCAGGCCTGAGGATCAACCGGTAGTGGGTNNNNGGAAGGGGATTGCTTTTTCCTTGACCAATTCTTTCAAAAAACGAATCGGATTCATGGGTCATAATAAGATAATTCATGATTCAGATGCTTAATAATAAGATAATAAGAAGGAATAATCAAATTGAATTCATGAATTTACCCGGCAATTTATGGTCCAATAAAGGATTTTTATCTTCGAAACCCATTGGAAGGGGTAGTGCACGAGAAAAAAAATCATGCGGAAATGATCGACTCCTTGGATGCCCCAAAATACTATGAGTGTCGGAA

161_20190629_05_01_trnT(GUU)-comp-psbD-comp CATAGAGATGAAATTGGAACAAGTTGACCCCCTTTTCTATTTATTTTCATTTCTTTGGACTCCGCAAGAATTTGCCGATATTTCCAATTCAATCGTCTTGTCTTGTTCCTAGATGTTCTATAGGAAAAAATTGTCATTTCGTTCCTCTACGGAGAACCTTTTATTCTAAATTCTAAATCACAGGATCTAAATTCTAAATCACAGGATAAGAAAAAAATTCACTATCTTTCTTTGATTACAGGATCAAGATTCATTTANNNNTTATCTATACTATATAATATATTCNNNNNNTATAGATATTTCTATTCTATATTTATATTTATAGCTATCAGATCGTGGCTTGATGTACCAAAAATTTCCATTTCGTTGCATCCAATATTTTTGTTCCGACCATCGTATGAAGAAAGCATGCAAGATAAATACTATCATTTCCAATCTCCTATTTTAATTTTAATATTGTANNNNNNTTGAAGTGAAGTAAAAATTTGGAAACCCTCTCTTTTCTAACAGGGAAAAATAAATCAAAAAATATTAGTAATTTAGTCTACATAAAAATTAGAATATAAAGAGAGTTCTTTTTCTTAATCTCATGAACAAGATCTAAGAATCCATTTAGTTGATGAAAGAACATGGGGGGGGGCAGGCCTGAGGATCAACCGGTAGTGGGTNNNNGGAAGGGGATTGCTTTTTCCTTGACCAATTCTTTCAAAAAACGAATCGGATTCATGGGTCATAATAAGATAATTCATGATTCAGATGCTTAATAATAAGATAATAAGAAGGAATAATCAAATTGAATTCATGAATTTACCCGGCAATTTATGGTCCAATAAAGGATTTTTATCTTCGAAACCCATTGGAAGGGGTAGTGCACGAGAAAAAAAATCATGCGGAAATGATCGACTCCTTGGATGCCCCAAAATACTATGAGTGTCGGAA

162_20190629_05_02_trnT(GUU)-comp-psbD-comp CATAGAGATGAAATTGGAACAAGTTGACCCCCTTTTCTATTTATTTTCATTTCTTTGGACTCCGCAAGAATTTGCCGATATTTCCAATTCAATCGTCTTGTCTTGTTCCTAGATGTTCTATAGGAAAAAATTGTCATTTCGTTCCTCTACGGAGAACCTTTTATTCTAAATTCTAAATCACAGGATCTAAATTCTAAATCACAGGATAAGAAAAAAATTCACTATCTTTCTTTGATTACAGGATCAAGATTCATTTANNNNTTATCTATACTATATAATATATTCNNNNNNTATAGATATTTCTATTCTATATTTATATTTATAGCTATCAGATCGTGGCTTGATGTACCAAAAATTTCCATTTCGTTGCATCCAATATTTTTGTTCCGACCATCGTATGAAGAAAGCATGCAAGATAAATACTATCATTTCCAATCTCCTATTTTAATTTTAATATTGTANNNNNNTTGAAGTGAAGTAAAAATTTGGAAACCCTCTCTTTTCTAACAGGGAAAAATAAATCAAAAAATATTAGTAATTTAGTCTACATAAAAATTAGAATATAAAGAGAGTTCTTTTTCTTAATCTCATGAACAAGATCTAAGAATCCATTTAGTTGATGAAAGAACATGGGGGGGGGCAGGCCTGAGGATCAACCGGTAGTGGGTNNNNGGAAGGGGATTGCTTTTTCCTTGACCAATTCTTTCAAAAAACGAATCGGATTCATGGGTCATAATAAGATAATTCATGATTCAGATGCTTAATAATAAGATAATAAGAAGGAATAATCAAATTGAATTCATGAATTTACCCGGCAATTTATGGTCCAATAAAGGATTTTTATCTTCGAAACCCATTGGAAGGGGTAGTGCACGAGAAAAAAAATCATGCGGAAATGATCGACTCCTTGGATGCCCCAAAATACTATGAGTGTCGGAA

163_20190629_06_01_trnT(GUU)-comp-psbD-comp CATAGAGATGAAATTGGAACAAGTTGACCCCCTTTTCTATTTATTTTCATTTCTTTGGACTCCGCAAGAATTTGCCGATATTTCCAATTCAATCGTCTTGTCTTGTTCCTAGATGTTCTATAGGAAAAAATTGTCATTTCGTTCCTCTACGGAGAACCTTTTATTCTAAATTCTAAATCACAGGATCTAAATTCTAAATCACAGGATAAGAAAAAAATTCACTATCTTTCTTTGATTACAGGATCAAGATTCATTTANNNNTTATCTATACTATATAATATATTCNNNNNNTATAGATATTTCTATTCTATATTTATATTTATAGCTATCAGATCGTGGCTTGATGTACCAAAAATTTCCATTTCGTTGCATCCAATATTTTTGTTCCGACCATCGTATGAAGAAAGCATGCAAGATAAATACTATCATTTCCAATCTCCTATTTTAATTTTAATATTGTANNNNNNTTGAAGTGAAGTAAAAATTTGGAAACCCTCTCTTTTCTAACAGGGAAAAATAAATCAAAAAATATTAGTAATTTAGTCTACATAAAAATTAGAATATAAAGAGAGTTCTTTTTCTTAATCTCATGAACAAGATCTAAGAATCCATTTAGTTGATGAAAGAACATGGGGGGGGGCAGGCCTGAGGATCAACCGGTAGTGGGTNNNNGGAAGGGGATTGCTTTTTCCTTGACCAATTCTTTCAAAAAACGAATCGGATTCATGGGTCATAATAAGATAATTCATGATTCAGATGCTTAATAATAAGATAATAAGAAGGAATAATCAAATTGAATTCATGAATTTACCCGGCAATTTATGGTCCAATAAAGGATTTTTATCTTCGAAACCCATTGGAAGGGGTAGTGCACGAGAAAAAAAATCATGCGGAAATGATCGACTCCTTGGATGCCCCAAAATACTATGAGTGTCGGAA

164_20190629_06_02_trnT(GUU)-comp-psbD-comp CATAGAGATGAAATTGGAACAAGTTGACCCCCTTTTCTATTTATTTTCATTTCTTTGGACTCCGCAAGAATTTGCCGATATTTCCAATTCAATCGTCTTGTCTTGTTCCTAGATGTTCTATAGGAAAAAATTGTCATTTCGTTCCTCTACGGAGAACCTTTTATTCTAAATTCTAAATCACAGGATCTAAATTCTAAATCACAGGATAAGAAAAAAATTCACTATCTTTCTTTGATTACAGGATCAAGATTCATTTANNNNTTATCTATACTATATAATATATTCNNNNNNTATAGATATTTCTATTCTATATTTATATTTATAGCTATCAGATCGTGGCTTGATGTACCAAAAATTTCCATTTCGTTGCATCCAATATTTTTGTTCCGACCATCGTATGAAGAAAGCATGCAAGATAAATACTATCATTTCCAATCTCCTATTTTAATTTTAATATTGTANNNNNNTTGAAGTGAAGTAAAAATTTGGAAACCCTCTCTTTTCTAACAGGGAAAAATAAATCAAAAAATATTAGTAATTTAGTCTACATAAAAATTAGAATATAAAGAGAGTTCTTTTTCTTAATCTCATGAACAAGATCTAAGAATCCATTTAGTTGATGAAAGAACATGGGGGGGGGCAGGCCTGAGGATCAACCGGTAGTGGGTNNNNGGAAGGGGATTGCTTTTTCCTTGACCAATTCTTTCAAAAAACGAATCGGATTCATGGGTCATAATAAGATAATTCATGATTCAGATGCTTAATAATAAGATAATAAGAAGGAATAATCAAATTGAATTCATGAATTTACCCGGCAATTTATGGTCCAATAAAGGATTTTTATCTTCGAAACCCATTGGAAGGGGTAGTGCACGAGAAAAAAAATCATGCGGAAATGATCGACTCCTTGGATGCCCCAAAATACTATGAGTGTCGGAA

165_20190716_01_01_trnT(GUU)-comp-psbD-comp CATAGAGATGAAATTGGAACAAGTTGACCCCCTTTTCTATTTATTTTCATTTCTTTGGACTCCGCAAGAATTTGCCGATATTTCCAATTCAATCGTCTTGTCTTGTTCCTAGATGTTCTATAGGAAAAAATTGTCATTTCGTTCCTCTACGGAGAACCTTTTATTCTAAATTCTAAATCACAGGATCTAAATTCTAAATCACAGGATAAGAAAAAAATTCACTATCTTTCTTTGATTACAGGATCAAGATTCATTTANNNNTTATCTATACTATATAATATATTCNNNNNNTATAGATATTTCTATTCTATATTTATATTTATAGCTATCAGATCGTGGCTTGATGTACCAAAAATTTCCATTTCGTTGCATCCAATATTTTTGTTCCGACCATCGTATGAAGAAAGCATGCAAGATAAATACTATCATTTCCAATCTCCTATTTTAATTTTAATATTGTANNNNNNTTGAAGTGAAGTAAAAATTTGGAAACCCTCTCTTTTCTAACAGGGAAAAATAAATCAAAAAATATTAGTAATTTAGTCTACATAAAAATTAGAATATAAAGAGAGTTCTTTTTCTTAATCTCATGAACAAGATCTAAGAATCCATTTAGTTGATGAAAGAACATGGGGGGGGGCAGGCCTGAGGATCAACCGGTAGTGGGTNNNNGGAAGGGGATTGCTTTTTCCTTGACCAATTCTTTCAAAAAACGAATCGGATTCATGGGTCATAATAAGATAATTCATGATTCAGATGCTTAATAATAAGATAATAAGAAGGAATAATCAAATTGAATTCATGAATTTACCCGGCAATTTATGGTCCAATAAAGGATTTTTATCTTCGAAACCCATTGGAAGGGGTAGTGCACGAGAAAAAAAATCATGCGGAAATGATCGACTCCTTGGATGCCCCAAAATACTATGAGTGTCGGAA

166_20190716_01_02_trnT(GUU)-comp-psbD-comp CATAGAGATGAAATTGGAACAAGTTGACCCCCTTTTCTATTTATTTTCATTTCTTTGGACTCCGCAAGAATTTGCCGATATTTCCAATTCAATCGTCTTGTCTTGTTCCTAGATGTTCTATAGGAAAAAATTGTCATTTCGTTCCTCTACGGAGAACCTTTTATTCTAAATTCTAAATCACAGGATCTAAATTCTAAATCACAGGATAAGAAAAAAATTCACTATCTTTCTTTGATTACAGGATCAAGATTCATTTANNNNTTATCTATACTATATAATATATTCNNNNNNTATAGATATTTCTATTCTATATTTATATTTATAGCTATCAGATCGTGGCTTGATGTACCAAAAATTTCCATTTCGTTGCATCCAATATTTTTGTTCCGACCATCGTATGAAGAAAGCATGCAAGATAAATACTATCATTTCCAATCTCCTATTTTAATTTTAATATTGTANNNNNNTTGAAGTGAAGTAAAAATTTGGAAACCCTCTCTTTTCTAACAGGGAAAAATAAATCAAAAAATATTAGTAATTTAGTCTACATAAAAATTAGAATATAAAGAGAGTTCTTTTTCTTAATCTCATGAACAAGATCTAAGAATCCATTTAGTTGATGAAAGAACATGGGGGGGGGCAGGCCTGAGGATCAACCGGTAGTGGGTNNNNGGAAGGGGATTGCTTTTTCCTTGACCAATTCTTTCAAAAAACGAATCGGATTCATGGGTCATAATAAGATAATTCATGATTCAGATGCTTAATAATAAGATAATAAGAAGGAATAATCAAATTGAATTCATGAATTTACCCGGCAATTTATGGTCCAATAAAGGATTTTTATCTTCGAAACCCATTGGAAGGGGTAGTGCACGAGAAAAAAAATCATGCGGAAATGATCGACTCCTTGGATGCCCCAAAATACTATGAGTGTCGGAA

167_20190716_01_03_trnT(GUU)-comp-psbD-comp CATAGAGATGAAATTGGAACAAGTTGACCCCCTTTTCTATTTATTTTCATTTCTTTGGACTCCGCAAGAATTTGCCGATATTTCCAATTCAATCGTCTTGTCTTGTTCCTAGATGTTCTATAGGAAAAAATTGTCATTTCGTTCCTCTACGGAGAACCTTTTATTCTAAATTCTAAATCACAGGATCTAAATTCTAAATCACAGGATAAGAAAAAAATTCACTATCTTTCTTTGATTACAGGATCAAGATTCATTTANNNNTTATCTATACTATATAATATATTCNNNNNNTATAGATATTTCTATTCTATATTTATATTTATAGCTATCAGATCGTGGCTTGATGTACCAAAAATTTCCATTTCGTTGCATCCAATATTTTTGTTCCGACCATCGTATGAAGAAAGCATGCAAGATAAATACTATCATTTCCAATCTCCTATTTTAATTTTAATATTGTANNNNNNTTGAAGTGAAGTAAAAATTTGGAAACCCTCTCTTTTCTAACAGGGAAAAATAAATCAAAAAATATTAGTAATTTAGTCTACATAAAAATTAGAATATAAAGAGAGTTCTTTTTCTTAATCTCATGAACAAGATCTAAGAATCCATTTAGTTGATGAAAGAACATGGGGGGGGGCAGGCCTGAGGATCAACCGGTAGTGGGTNNNNGGAAGGGGATTGCTTTTTCCTTGACCAATTCTTTCAAAAAACGAATCGGATTCATGGGTCATAATAAGATAATTCATGATTCAGATGCTTAATAATAAGATAATAAGAAGGAATAATCAAATTGAATTCATGAATTTACCCGGCAATTTATGGTCCAATAAAGGATTTTTATCTTCGAAACCCATTGGAAGGGGTAGTGCACGAGAAAAAAAATCATGCGGAAATGATCGACTCCTTGGATGCCCCAAAATACTATGAGTGTCGGAA

168_20190716_01_04_trnT(GUU)-comp-psbD-comp CATAGAGATGAAATTGGAACAAGTTGACCCCCTTTTCTATTTATTTTCATTTCTTTGGACTCCGCAAGAATTTGCCGATATTTCCAATTCAATCGTCTTGTCTTGTTCCTAGATGTTCTATAGGAAAAAATTGTCATTTCGTTCCTCTACGGAGAACCTTTTATTCTAAATTCTAAATCACAGGATCTAAATTCTAAATCACAGGATAAGAAAAAAATTCACTATCTTTCTTTGATTACAGGATCAAGATTCATTTANNNNTTATCTATACTATATAATATATTCNNNNNNTATAGATATTTCTATTCTATATTTATATTTATAGCTATCAGATCGTGGCTTGATGTACCAAAAATTTCCATTTCGTTGCATCCAATATTTTTGTTCCGACCATCGTATGAAGAAAGCATGCAAGATAAATACTATCATTTCCAATCTCCTATTTTAATTTTAATATTGTANNNNNNTTGAAGTGAAGTAAAAATTTGGAAACCCTCTCTTTTCTAACAGGGAAAAATAAATCAAAAAATATTAGTAATTTAGTCTACATAAAAATTAGAATATAAAGAGAGTTCTTTTTCTTAATCTCATGAACAAGATCTAAGAATCCATTTAGTTGATGAAAGAACATGGGGGGGGGCAGGCCTGAGGATCAACCGGTAGTGGGTNNNNGGAAGGGGATTGCTTTTTCCTTGACCAATTCTTTCAAAAAACGAATCGGATTCATGGGTCATAATAAGATAATTCATGATTCAGATGCTTAATAATAAGATAATAAGAAGGAATAATCAAATTGAATTCATGAATTTACCCGGCAATTTATGGTCCAATAAAGGATTTTTATCTTCGAAACCCATTGGAAGGGGTAGTGCACGAGAAAAAAAATCATGCGGAAATGATCGACTCCTTGGATGCCCCAAAATACTATGAGTGTCGGAA

169_20190716_01_05_trnT(GUU)-comp-psbD-comp CATAGAGATGAAATTGGAACAAGTTGACCCCCTTTTCTATTTATTTTCATTTCTTTGGACTCCGCAAGAATTTGCCGATATTTCCAATTCAATCGTCTTGTCTTGTTCCTAGATGTTCTATAGGAAAAAATTGTCATTTCGTTCCTCTACGGAGAACCTTTTATTCTAAATTCTAAATCACAGGATCTAAATTCTAAATCACAGGATAAGAAAAAAATTCACTATCTTTCTTTGATTACAGGATCAAGATTCATTTANNNNTTATCTATACTATATAATATATTCNNNNNNTATAGATATTTCTATTCTATATTTATATTTATAGCTATCAGATCGTGGCTTGATGTACCAAAAATTTCCATTTCGTTGCATCCAATATTTTTGTTCCGACCATCGTATGAAGAAAGCATGCAAGATAAATACTATCATTTCCAATCTCCTATTTTAATTTTAATATTGTANNNNNNTTGAAGTGAAGTAAAAATTTGGAAACCCTCTCTTTTCTAACAGGGAAAAATAAATCAAAAAATATTAGTAATTTAGTCTACATAAAAATTAGAATATAAAGAGAGTTCTTTTTCTTAATCTCATGAACAAGATCTAAGAATCCATTTAGTTGATGAAAGAACATGGGGGGGGGCAGGCCTGAGGATCAACCGGTAGTGGGTNNNNGGAAGGGGATTGCTTTTTCCTTGACCAATTCTTTCAAAAAACGAATCGGATTCATGGGTCATAATAAGATAATTCATGATTCAGATGCTTAATAATAAGATAATAAGAAGGAATAATCAAATTGAATTCATGAATTTACCCGGCAATTTATGGTCCAATAAAGGATTTTTATCTTCGAAACCCATTGGAAGGGGTAGTGCACGAGAAAAAAAATCATGCGGAAATGATCGACTCCTTGGATGCCCCAAAATACTATGAGTGTCGGAA

170_20190717_01_01_trnT(GUU)-comp-psbD-comp CATAGAGATGAAATTGGAACAAGTTGACCCCCTTTTCTATTTATTTTCATTTCTTTGGACTCCGCAAGAATTTGCCGATATTTCCAATTCAATCGTCTTGTCTTGTTCCTAGATGTTCTATAGGAAAAAATTGTCATTTCGTTCCTCTACGGAGAACCTTTTATTCTAAATTCTAAATCACAGGATCTAAATTCTAAATCACAGGATAAGAAAAAAATTCACTATCTTTCTTTGATTACAGGATCAAGATTCATTTANNNNTTATCTATACTATATAATATATTCNNNNNNTATAGATATTTCTATTCTATATTTATATTTATAGCTATCAGATCGTGGCTTGATGTACCAAAAATTTCCATTTCGTTGCATCCAATATTTTTGTTCCGACCATCGTATGAAGAAAGCATGCAAGATAAATACTATCATTTCCAATCTCCTATTTTAATTTTAATATTGTANNNNNNTTGAAGTGAAGTAAAAATTTGGAAACCCTCTCTTTTCTAACAGGGAAAAATAAATCAAAAAATATTAGTAATTTAGTCTACATAAAAATTAGAATATAAAGAGAGTTCTTTTTCTTAATCTCATGAACAAGATCTAAGAATCCATTTAGTTGATGAAAGAACATGGGGGGGGGCAGGCCTGAGGATCAACCGGTAGTGGGTNNNNGGAAGGGGATTGCTTTTTCCTTGACCAATTCTTTCAAAAAACGAATCGGATTCATGGGTCATAATAAGATAATTCATGATTCAGATGCTTAATAATAAGATAATAAGAAGGAATAATCAAATTGAATTCATGAATTTACCCGGCAATTTATGGTCCAATAAAGGATTTTTATCTTCGAAACCCATTGGAAGGGGTAGTGCACGAGAAAAAAAATCATGCGGAAATGATCGACTCCTTGGATGCCCCAAAATACTATGAGTGTCGGAA

171_20190717_01_02_trnT(GUU)-comp-psbD-comp CATAGAGATGAAATTGGAACAAGTTGACCCCCTTTTCTATTTATTTTCATTTCTTTGGACTCCGCAAGAATTTGCCGATATTTCCAATTCAATCGTCTTGTCTTGTTCCTAGATGTTCTATAGGAAAAAATTGTCATTTCGTTCCTCTACGGAGAACCTTTTATTCTAAATTCTAAATCACAGGATCTAAATTCTAAATCACAGGATAAGAAAAAAATTCACTATCTTTCTTTGATTACAGGATCAAGATTCATTTANNNNTTATCTATACTATATAATATATTCNNNNNNTATAGATATTTCTATTCTATATTTATATTTATAGCTATCAGATCGTGGCTTGATGTACCAAAAATTTCCATTTCGTTGCATCCAATATTTTTGTTCCGACCATCGTATGAAGAAAGCATGCAAGATAAATACTATCATTTCCAATCTCCTATTTTAATTTTAATATTGTANNNNNNTTGAAGTGAAGTAAAAATTTGGAAACCCTCTCTTTTCTAACAGGGAAAAATAAATCAAAAAATATTAGTAATTTAGTCTACATAAAAATTAGAATATAAAGAGAGTTCTTTTTCTTAATCTCATGAACAAGATCTAAGAATCCATTTAGTTGATGAAAGAACATGGGGGGGGGCAGGCCTGAGGATCAACCGGTAGTGGGTNNNNGGAAGGGGATTGCTTTTTCCTTGACCAATTCTTTCAAAAAACGAATCGGATTCATGGGTCATAATAAGATAATTCATGATTCAGATGCTTAATAATAAGATAATAAGAAGGAATAATCAAATTGAATTCATGAATTTACCCGGCAATTTATGGTCCAATAAAGGATTTTTATCTTCGAAACCCATTGGAAGGGGTAGTGCACGAGAAAAAAAATCATGCGGAAATGATCGACTCCTTGGATGCCCCAAAATACTATGAGTGTCGGAA

172_20190717_01_03_trnT(GUU)-comp-psbD-comp CATAGAGATGAAATTGGAACAAGTTGACCCCCTTTTCTATTTATTTTCATTTCTTTGGACTCCGCAAGAATTTGCCGATATTTCCAATTCAATCGTCTTGTCTTGTTCCTAGATGTTCTATAGGAAAAAATTGTCATTTCGTTCCTCTACGGAGAACCTTTTATTCTAAATTCTAAATCACAGGATCTAAATTCTAAATCACAGGATAAGAAAAAAATTCACTATCTTTCTTTGATTACAGGATCAAGATTCATTTANNNNTTATCTATACTATATAATATATTCNNNNNNTATAGATATTTCTATTCTATATTTATATTTATAGCTATCAGATCGTGGCTTGATGTACCAAAAATTTCCATTTCGTTGCATCCAATATTTTTGTTCCGACCATCGTATGAAGAAAGCATGCAAGATAAATACTATCATTTCCAATCTCCTATTTTAATTTTAATATTGTANNNNNNTTGAAGTGAAGTAAAAATTTGGAAACCCTCTCTTTTCTAACAGGGAAAAATAAATCAAAAAATATTAGTAATTTAGTCTACATAAAAATTAGAATATAAAGAGAGTTCTTTTTCTTAATCTCATGAACAAGATCTAAGAATCCATTTAGTTGATGAAAGAACATGGGGGGGGGCAGGCCTGAGGATCAACCGGTAGTGGGTNNNNGGAAGGGGATTGCTTTTTCCTTGACCAATTCTTTCAAAAAACGAATCGGATTCATGGGTCATAATAAGATAATTCATGATTCAGATGCTTAATAATAAGATAATAAGAAGGAATAATCAAATTGAATTCATGAATTTACCCGGCAATTTATGGTCCAATAAAGGATTTTTATCTTCGAAACCCATTGGAAGGGGTAGTGCACGAGAAAAAAAATCATGCGGAAATGATCGACTCCTTGGATGCCCCAAAATACTATGAGTGTCGGAA

173_20190717_01_04_trnT(GUU)-comp-psbD-comp CATAGAGATGAAATTGGAACAAGTTGACCCCCTTTTCTATTTATTTTCATTTCTTTGGACTCCGCAAGAATTTGCCGATATTTCCAATTCAATCGTCTTGTCTTGTTCCTAGATGTTCTATAGGAAAAAATTGTCATTTCGTTCCTCTACGGAGAACCTTTTATTCTAAATTCTAAATCACAGGATCTAAATTCTAAATCACAGGATAAGAAAAAAATTCACTATCTTTCTTTGATTACAGGATCAAGATTCATTTANNNNTTATCTATACTATATAATATATTCNNNNNNTATAGATATTTCTATTCTATATTTATATTTATAGCTATCAGATCGTGGCTTGATGTACCAAAAATTTCCATTTCGTTGCATCCAATATTTTTGTTCCGACCATCGTATGAAGAAAGCATGCAAGATAAATACTATCATTTCCAATCTCCTATTTTAATTTTAATATTGTANNNNNNTTGAAGTGAAGTAAAAATTTGGAAACCCTCTCTTTTCTAACAGGGAAAAATAAATCAAAAAATATTAGTAATTTAGTCTACATAAAAATTAGAATATAAAGAGAGTTCTTTTTCTTAATCTCATGAACAAGATCTAAGAATCCATTTAGTTGATGAAAGAACATGGGGGGGGGCAGGCCTGAGGATCAACCGGTAGTGGGTNNNNGGAAGGGGATTGCTTTTTCCTTGACCAATTCTTTCAAAAAACGAATCGGATTCATGGGTCATAATAAGATAATTCATGATTCAGATGCTTAATAATAAGATAATAAGAAGGAATAATCAAATTGAATTCATGAATTTACCCGGCAATTTATGGTCCAATAAAGGATTTTTATCTTCGAAACCCATTGGAAGGGGTAGTGCACGAGAAAAAAAATCATGCGGAAATGATCGACTCCTTGGATGCCCCAAAATACTATGAGTGTCGGAA

174_20190717_01_05_trnT(GUU)-comp-psbD-comp CATAGAGATGAAATTGGAACAAGTTGACCCCCTTTTCTATTTATTTTCATTTCTTTGGACTCCGCAAGAATTTGCCGATATTTCCAATTCAATCGTCTTGTCTTGTTCCTAGATGTTCTATAGGAAAAAATTGTCATTTCGTTCCTCTACGGAGAACCTTTTATTCTAAATTCTAAATCACAGGATCTAAATTCTAAATCACAGGATAAGAAAAAAATTCACTATCTTTCTTTGATTACAGGATCAAGATTCATTTANNNNTTATCTATACTATATAATATATTCNNNNNNTATAGATATTTCTATTCTATATTTATATTTATAGCTATCAGATCGTGGCTTGATGTACCAAAAATTTCCATTTCGTTGCATCCAATATTTTTGTTCCGACCATCGTATGAAGAAAGCATGCAAGATAAATACTATCATTTCCAATCTCCTATTTTAATTTTAATATTGTANNNNNNTTGAAGTGAAGTAAAAATTTGGAAACCCTCTCTTTTCTAACAGGGAAAAATAAATCAAAAAATATTAGTAATTTAGTCTACATAAAAATTAGAATATAAAGAGAGTTCTTTTTCTTAATCTCATGAACAAGATCTAAGAATCCATTTAGTTGATGAAAGAACATGGGGGGGGGCAGGCCTGAGGATCAACCGGTAGTGGGTNNNNGGAAGGGGATTGCTTTTTCCTTGACCAATTCTTTCAAAAAACGAATCGGATTCATGGGTCATAATAAGATAATTCATGATTCAGATGCTTAATAATAAGATAATAAGAAGGAATAATCAAATTGAATTCATGAATTTACCCGGCAATTTATGGTCCAATAAAGGATTTTTATCTTCGAAACCCATTGGAAGGGGTAGTGCACGAGAAAAAAAATCATGCGGAAATGATCGACTCCTTGGATGCCCCAAAATACTATGAGTGTCGGAA

175_20190716_02_01_trnT(GUU)-comp-psbD-comp CATAGAGATGAAATTGGAACAAGTTGACCCCCTTTTCTATTTATTTTCATTTCTTTGGACTCCGCAAGAATTTGCCGATATTTCCAATTCAATCGTCTTGTCTTGTTCCTAGATGTTCTATAGGAAAAAATTGTCATTTCGTTCCTCTACGGAGAACCTTTTATTCTAAATTCTAAATCACAGGATCTAAATTCTAAATCACAGGATAAGAAAAAAATTCACTATCTTTCTTTGATTACAGGATCAAGATTCATTTANNNNTTATCTATACTATATAATATATTCNNNNNNTATAGATATTTCTATTCTATATTTATATTTATAGCTATCAGATCGTGGCTTGATGTACCAAAAATTTCCATTTCGTTGCATCCAATATTTTTGTTCCGACCATCGTATGAAGAAAGCATGCAAGATAAATACTATCATTTCCAATCTCCTATTTTAATTTTAATATTGTANNNNNNTTGAAGTGAAGTAAAAATTTGGAAACCCTCTCTTTTCTAACAGGGAAAAATAAATCAAAAAATATTAGTAATTTAGTCTACATAAAAATTAGAATATAAAGAGAGTTCTTTTTCTTAATCTCATGAACAAGATCTAAGAATCCATTTAGTTGATGAAAGAACATGGGGGGGGGCAGGCCTGAGGATCAACCGGTAGTGGGTNNNNGGAAGGGGATTGCTTTTTCCTTGACCAATTCTTTCAAAAAACGAATCGGATTCATGGGTCATAATAAGATAATTCATGATTCAGATGCTTAATAATAAGATAATAAGAAGGAATAATCAAATTGAATTCATGAATTTACCCGGCAATTTATGGTCCAATAAAGGATTTTTATCTTCGAAACCCATTGGAAGGGGTAGTGCACGAGAAAAAAAATCATGCGGAAATGATCGACTCCTTGGATGCCCCAAAATACTATGAGTGTCGGAA

176_20190716_02_02_trnT(GUU)-comp-psbD-comp CATAGAGATGAAATTGGAACAAGTTGACCCCCTTTTCTATTTATTTTCATTTCTTTGGACTCCGCAAGAATTTGCCGATATTTCCAATTCAATCGTCTTGTCTTGTTCCTAGATGTTCTATAGGAAAAAATTGTCATTTCGTTCCTCTACGGAGAACCTTTTATTCTAAATTCTAAATCACAGGATCTAAATTCTAAATCACAGGATAAGAAAAAAATTCACTATCTTTCTTTGATTACAGGATCAAGATTCATTTANNNNTTATCTATACTATATAATATATTCNNNNNNTATAGATATTTCTATTCTATATTTATATTTATAGCTATCAGATCGTGGCTTGATGTACCAAAAATTTCCATTTCGTTGCATCCAATATTTTTGTTCCGACCATCGTATGAAGAAAGCATGCAAGATAAATACTATCATTTCCAATCTCCTATTTTAATTTTAATATTGTANNNNNNTTGAAGTGAAGTAAAAATTTGGAAACCCTCTCTTTTCTAACAGGGAAAAATAAATCAAAAAATATTAGTAATTTAGTCTACATAAAAATTAGAATATAAAGAGAGTTCTTTTTCTTAATCTCATGAACAAGATCTAAGAATCCATTTAGTTGATGAAAGAACATGGGGGGGGGCAGGCCTGAGGATCAACCGGTAGTGGGTNNNNGGAAGGGGATTGCTTTTTCCTTGACCAATTCTTTCAAAAAACGAATCGGATTCATGGGTCATAATAAGATAATTCATGATTCAGATGCTTAATAATAAGATAATAAGAAGGAATAATCAAATTGAATTCATGAATTTACCCGGCAATTTATGGTCCAATAAAGGATTTTTATCTTCGAAACCCATTGGAAGGGGTAGTGCACGAGAAAAAAAATCATGCGGAAATGATCGACTCCTTGGATGCCCCAAAATACTATGAGTGTCGGAA

177_20190716_02_03_trnT(GUU)-comp-psbD-comp CATAGAGATGAAATTGGAACAAGTTGACCCCCTTTTCTATTTATTTTCATTTCTTTGGACTCCGCAAGAATTTGCCGATATTTCCAATTCAATCGTCTTGTCTTGTTCCTAGATGTTCTATAGGAAAAAATTGTCATTTCGTTCCTCTACGGAGAACCTTTTATTCTAAATTCTAAATCACAGGATCTAAATTCTAAATCACAGGATAAGAAAAAAATTCACTATCTTTCTTTGATTACAGGATCAAGATTCATTTANNNNTTATCTATACTATATAATATATTCNNNNNNTATAGATATTTCTATTCTATATTTATATTTATAGCTATCAGATCGTGGCTTGATGTACCAAAAATTTCCATTTCGTTGCATCCAATATTTTTGTTCCGACCATCGTATGAAGAAAGCATGCAAGATAAATACTATCATTTCCAATCTCCTATTTTAATTTTAATATTGTANNNNNNTTGAAGTGAAGTAAAAATTTGGAAACCCTCTCTTTTCTAACAGGGAAAAATAAATCAAAAAATATTAGTAATTTAGTCTACATAAAAATTAGAATATAAAGAGAGTTCTTTTTCTTAATCTCATGAACAAGATCTAAGAATCCATTTAGTTGATGAAAGAACATGGGGGGGGGCAGGCCTGAGGATCAACCGGTAGTGGGTNNNNGGAAGGGGATTGCTTTTTCCTTGACCAATTCTTTCAAAAAACGAATCGGATTCATGGGTCATAATAAGATAATTCATGATTCAGATGCTTAATAATAAGATAATAAGAAGGAATAATCAAATTGAATTCATGAATTTACCCGGCAATTTATGGTCCAATAAAGGATTTTTATCTTCGAAACCCATTGGAAGGGGTAGTGCACGAGAAAAAAAATCATGCGGAAATGATCGACTCCTTGGATGCCCCAAAATACTATGAGTGTCGGAA

178_20190716_02_04_trnT(GUU)-comp-psbD-comp CATAGAGATGAAATTGGAACAAGTTGACCCCCTTTTCTATTTATTTTCATTTCTTTGGACTCCGCAAGAATTTGCCGATATTTCCAATTCAATCGTCTTGTCTTGTTCCTAGATGTTCTATAGGAAAAAATTGTCATTTCGTTCCTCTACGGAGAACCTTTTATTCTAAATTCTAAATCACAGGATCTAAATTCTAAATCACAGGATAAGAAAAAAATTCACTATCTTTCTTTGATTACAGGATCAAGATTCATTTANNNNTTATCTATACTATATAATATATTCNNNNNNTATAGATATTTCTATTCTATATTTATATTTATAGCTATCAGATCGTGGCTTGATGTACCAAAAATTTCCATTTCGTTGCATCCAATATTTTTGTTCCGACCATCGTATGAAGAAAGCATGCAAGATAAATACTATCATTTCCAATCTCCTATTTTAATTTTAATATTGTANNNNNNTTGAAGTGAAGTAAAAATTTGGAAACCCTCTCTTTTCTAACAGGGAAAAATAAATCAAAAAATATTAGTAATTTAGTCTACATAAAAATTAGAATATAAAGAGAGTTCTTTTTCTTAATCTCATGAACAAGATCTAAGAATCCATTTAGTTGATGAAAGAACATGGGGGGGGGCAGGCCTGAGGATCAACCGGTAGTGGGTNNNNGGAAGGGGATTGCTTTTTCCTTGACCAATTCTTTCAAAAAACGAATCGGATTCATGGGTCATAATAAGATAATTCATGATTCAGATGCTTAATAATAAGATAATAAGAAGGAATAATCAAATTGAATTCATGAATTTACCCGGCAATTTATGGTCCAATAAAGGATTTTTATCTTCGAAACCCATTGGAAGGGGTAGTGCACGAGAAAAAAAATCATGCGGAAATGATCGACTCCTTGGATGCCCCAAAATACTATGAGTGTCGGAA

179_20190716_02_05_trnT(GUU)-comp-psbD-comp CATAGAGATGAAATTGGAACAAGTTGACCCCCTTTTCTATTTATTTTCATTTCTTTGGACTCCGCAAGAATTTGCCGATATTTCCAATTCAATCGTCTTGTCTTGTTCCTAGATGTTCTATAGGAAAAAATTGTCATTTCGTTCCTCTACGGAGAACCTTTTATTCTAAATTCTAAATCACAGGATCTAAATTCTAAATCACAGGATAAGAAAAAAATTCACTATCTTTCTTTGATTACAGGATCAAGATTCATTTANNNNTTATCTATACTATATAATATATTCNNNNNNTATAGATATTTCTATTCTATATTTATATTTATAGCTATCAGATCGTGGCTTGATGTACCAAAAATTTCCATTTCGTTGCATCCAATATTTTTGTTCCGACCATCGTATGAAGAAAGCATGCAAGATAAATACTATCATTTCCAATCTCCTATTTTAATTTTAATATTGTANNNNNNTTGAAGTGAAGTAAAAATTTGGAAACCCTCTCTTTTCTAACAGGGAAAAATAAATCAAAAAATATTAGTAATTTAGTCTACATAAAAATTAGAATATAAAGAGAGTTCTTTTTCTTAATCTCATGAACAAGATCTAAGAATCCATTTAGTTGATGAAAGAACATGGGGGGGGGCAGGCCTGAGGATCAACCGGTAGTGGGTNNNNGGAAGGGGATTGCTTTTTCCTTGACCAATTCTTTCAAAAAACGAATCGGATTCATGGGTCATAATAAGATAATTCATGATTCAGATGCTTAATAATAAGATAATAAGAAGGAATAATCAAATTGAATTCATGAATTTACCCGGCAATTTATGGTCCAATAAAGGATTTTTATCTTCGAAACCCATTGGAAGGGGTAGTGCACGAGAAAAAAAATCATGCGGAAATGATCGACTCCTTGGATGCCCCAAAATACTATGAGTGTCGGAA

180_20190719_01_01_trnT(GUU)-comp-psbD-comp CATAGAGATGAAATTGGAACAAGTTGACCCCCTTTTCTATTTATTTTCATTTCTTTGGACTCCGCAAGAATTTGCCGATATTTCCAATTCAATCGTCTTGTCTTGTTCCTAGATGTTCTATAGGAAAAAATTGTCATTTCGTTCCTCTACGGAGAACCTTTTATTCTAAATTCTAAATCACAGGATCTAAATTCTAAATCACAGGATAAGAAAAAAATTCACTATCTTTCTTTGATTACAGGATCAAGATTCATTTANNNNTTATCTATACTATATAATATATTCNNNNNNTATAGATATTTCTATTCTATATTTATATTTATAGCTATCAGATCGTGGCTTGATGTACCAAAAATTTCCATTTCGTTGCATCCAATATTTTTGTTCCGACCATCGTATGAAGAAAGCATGCAAGATAAATACTATCATTTCCAATCTCCTATTTTAATTTTAATATTGTANNNNNNTTGAAGTGAAGTAAAAATTTGGAAACCCTCTCTTTTCTAACAGGGAAAAATAAATCAAAAAATATTAGTAATTTAGTCTACATAAAAATTAGAATATAAAGAGAGTTCTTTTTCTTAATCTCATGAACAAGATCTAAGAATCCATTTAGTTGATGAAAGAACATGGGGGGGGGCAGGCCTGAGGATCAACCGGTAGTGGGTNNNNGGAAGGGGATTGCTTTTTCCTTGACCAATTCTTTCAAAAAACGAATCGGATTCATGGGTCATAATAAGATAATTCATGATTCAGATGCTTAATAATAAGATAATAAGAAGGAATAATCAAATTGAATTCATGAATTTACCCGGCAATTTATGGTCCAATAAAGGATTTTTATCTTCGAAACCCATTGGAAGGGGTAGTGCACGAGAAAAAAAATCATGCGGAAATGATCGACTCCTTGGATGCCCCAAAATACTATGAGTGTCGGAA

181_20190719_01_02_trnT(GUU)-comp-psbD-comp CATAGAGATGAAATTGGAACAAGTTGACCCCCTTTTCTATTTATTTTCATTTCTTTGGACTCCGCAAGAATTTGCCGATATTTCCAATTCAATCGTCTTGTCTTGTTCCTAGATGTTCTATAGGAAAAAATTGTCATTTCGTTCCTCTACGGAGAACCTTTTATTCTAAATTCTAAATCACAGGATCTAAATTCTAAATCACAGGATAAGAAAAAAATTCACTATCTTTCTTTGATTACAGGATCAAGATTCATTTANNNNTTATCTATACTATATAATATATTCNNNNNNTATAGATATTTCTATTCTATATTTATATTTATAGCTATCAGATCGTGGCTTGATGTACCAAAAATTTCCATTTCGTTGCATCCAATATTTTTGTTCCGACCATCGTATGAAGAAAGCATGCAAGATAAATACTATCATTTCCAATCTCCTATTTTAATTTTAATATTGTANNNNNNTTGAAGTGAAGTAAAAATTTGGAAACCCTCTCTTTTCTAACAGGGAAAAATAAATCAAAAAATATTAGTAATTTAGTCTACATAAAAATTAGAATATAAAGAGAGTTCTTTTTCTTAATCTCATGAACAAGATCTAAGAATCCATTTAGTTGATGAAAGAACATGGGGGGGGGCAGGCCTGAGGATCAACCGGTAGTGGGTNNNNGGAAGGGGATTGCTTTTTCCTTGACCAATTCTTTCAAAAAACGAATCGGATTCATGGGTCATAATAAGATAATTCATGATTCAGATGCTTAATAATAAGATAATAAGAAGGAATAATCAAATTGAATTCATGAATTTACCCGGCAATTTATGGTCCAATAAAGGATTTTTATCTTCGAAACCCATTGGAAGGGGTAGTGCACGAGAAAAAAAATCATGCGGAAATGATCGACTCCTTGGATGCCCCAAAATACTATGAGTGTCGGAA

182_20190719_01_03_trnT(GUU)-comp-psbD-comp CATAGAGATGAAATTGGAACAAGTTGACCCCCTTTTCTATTTATTTTCATTTCTTTGGACTCCGCAAGAATTTGCCGATATTTCCAATTCAATCGTCTTGTCTTGTTCCTAGATGTTCTATAGGAAAAAATTGTCATTTCGTTCCTCTACGGAGAACCTTTTATTCTAAATTCTAAATCACAGGATCTAAATTCTAAATCACAGGATAAGAAAAAAATTCACTATCTTTCTTTGATTACAGGATCAAGATTCATTTANNNNTTATCTATACTATATAATATATTCNNNNNNTATAGATATTTCTATTCTATATTTATATTTATAGCTATCAGATCGTGGCTTGATGTACCAAAAATTTCCATTTCGTTGCATCCAATATTTTTGTTCCGACCATCGTATGAAGAAAGCATGCAAGATAAATACTATCATTTCCAATCTCCTATTTTAATTTTAATATTGTANNNNNNTTGAAGTGAAGTAAAAATTTGGAAACCCTCTCTTTTCTAACAGGGAAAAATAAATCAAAAAATATTAGTAATTTAGTCTACATAAAAATTAGAATATAAAGAGAGTTCTTTTTCTTAATCTCATGAACAAGATCTAAGAATCCATTTAGTTGATGAAAGAACATGGGGGGGGGCAGGCCTGAGGATCAACCGGTAGTGGGTNNNNGGAAGGGGATTGCTTTTTCCTTGACCAATTCTTTCAAAAAACGAATCGGATTCATGGGTCATAATAAGATAATTCATGATTCAGATGCTTAATAATAAGATAATAAGAAGGAATAATCAAATTGAATTCATGAATTTACCCGGCAATTTATGGTCCAATAAAGGATTTTTATCTTCGAAACCCATTGGAAGGGGTAGTGCACGAGAAAAAAAATCATGCGGAAATGATCGACTCCTTGGATGCCCCAAAATACTATGAGTGTCGGAA

183_20190719_03_01_trnT(GUU)-comp-psbD-comp CATAGAGATGAAATTGGAACAAGTTGACCCCCTTTTCTATTTATTTTCATTTCTTTGGACTCCGCAAGAATTTGCCGATATTTCCAATTCAATCGTCTTGTCTTGTTCCTAGATGTTCTATAGGAAAAAATTGTCATTTCGTTCCTCTACGGAGAACCTTTTATTCTAAATTCTAAATCACAGGATCTAAATTCTAAATCACAGGATAAGAAAAAAATTCACTATCTTTCTTTGATTACAGGATCAAGATTCATTTANNNNTTATCTATACTATATAATATATTCNNNNNNTATAGATATTTCTATTCTATATTTAGATTTATAGCTATCAGATCGTGGCTTGATGTACCAAAAATTTCCATTTCGTTGCATCCAATATTTTTGTTCCGACCATCGTATGAAGAAAGCATGCAAGATAAATACTATCATTTCCAATCTCCTATTTTAATTTTAATATTGTANNNNNNTTGAAGTGAAGTAAAAATTTGGAAACCCTCTCTTTTCTAACAGGGAAAAATGAATCAAAAAATATTAGTAATTTAGTCTACATAAAAATTAGAATATAAAGAGAGTTCTTTTTCTTAATCTCATGAACAAGATCTAAGAATCCATTTAGTTGATGAAAGAACATGGGGGGGGGCAGGCCTGAGGATCAACCGGTAGTGGGTNNNNGGAAGGGGATTGCTTTTTCCTTGACCAATTCTTTCAAAAAACGAATCGGATTCATGGGTCATAATAAGATAATTCATGATTCAGATGCTTAATAATAAGATAATAAGAAGGAATAATCAAATTGAATTCATGAATTTACCCGGCAATTTATGGTCCAATAAAGGATTTTTATCTTCGAAACCCATTGGAAGGGGTAGTGCACGAGAAAAAAAATCATGCGGAAATGATCGACTCCTTGGATGCCCCAAAATACTATGAGTGTCGGAA

184_20190719_03_02_trnT(GUU)-comp-psbD-comp CATAGAGATGAAATTGGAACAAGTTGACCCCCTTTTCTATTTATTTTCATTTCTTTGGACTCCGCAAGAATTTGCCGATATTTCCAATTCAATCGTCTTGTCTTGTTCCTAGATGTTCTATAGGAAAAAATTGTCATTTCGTTCCTCTACGGAGAACCTTTTATTCTAAATTCTAAATCACAGGATCTAAATTCTAAATCACAGGATAAGAAAAAAATTCACTATCTTTCTTTGATTACAGGATCAAGATTCATTTANNNNTTATCTATACTATATAATATATTCNNNNNNTATAGATATTTCTATTCTATATTTAGATTTATAGCTATCAGATCGTGGCTTGATGTACCAAAAATTTCCATTTCGTTGCATCCAATATTTTTGTTCCGACCATCGTATGAAGAAAGCATGCAAGATAAATACTATCATTTCCAATCTCCTATTTTAATTTTAATATTGTANNNNNNTTGAAGTGAAGTAAAAATTTGGAAACCCTCTCTTTTCTAACAGGGAAAAATGAATCAAAAAATATTAGTAATTTAGTCTACATAAAAATTAGAATATAAAGAGAGTTCTTTTTCTTAATCTCATGAACAAGATCTAAGAATCCATTTAGTTGATGAAAGAACATGGGGGGGGGCAGGCCTGAGGATCAACCGGTAGTGGGTNNNNGGAAGGGGATTGCTTTTTCCTTGACCAATTCTTTCAAAAAACGAATCGGATTCATGGGTCATAATAAGATAATTCATGATTCAGATGCTTAATAATAAGATAATAAGAAGGAATAATCAAATTGAATTCATGAATTTACCCGGCAATTTATGGTCCAATAAAGGATTTTTATCTTCGAAACCCATTGGAAGGGGTAGTGCACGAGAAAAAAAATCATGCGGAAATGATCGACTCCTTGGATGCCCCAAAATACTATGAGTGTCGGAA

185_20190719_03_03_trnT(GUU)-comp-psbD-comp CATAGAGATGAAATTGGAACAAGTTGACCCCCTTTTCTATTTATTTTCATTTCTTTGGACTCCGCAAGAATTTGCCGATATTTCCAATTCAATCGTCTTGTCTTGTTCCTAGATGTTCTATAGGAAAAAATTGTCATTTCGTTCCTCTACGGAGAACCTTTTATTCTAAATTCTAAATCACAGGATCTAAATTCTAAATCACAGGATAAGAAAAAAATTCACTATCTTTCTTTGATTACAGGATCAAGATTCATTTANNNNTTATCTATACTATATAATATATTCNNNNNNTATAGATATTTCTATTCTATATTTAGATTTATAGCTATCAGATCGTGGCTTGATGTACCAAAAATTTCCATTTCGTTGCATCCAATATTTTTGTTCCGACCATCGTATGAAGAAAGCATGCAAGATAAATACTATCATTTCCAATCTCCTATTTTAATTTTAATATTGTANNNNNNTTGAAGTGAAGTAAAAATTTGGAAACCCTCTCTTTTCTAACAGGGAAAAATAAATCAAAAAATATTAGTAATTTAGTCTACATAAAAATTAGAATATAAAGAGAGTTCTTTTTCTTAATCTCATGAACAAGATCTAAGAATCCATTTAGTTGATGAAAGAACATGGGGGGGGGCAGGCCTGAGGATCAACCGGTAGTGGGTNNNNGGAAGGGGATTGCTTTTTCCTTGACCAATTCTTTCAAAAAACGAATCGGATTCATGGGTCATAATAAGATAATTCATGATTCAGATGCTTAATAATAAGATAATAAGAAGGAATAATCAAATTGAATTCATGAATTTACCCGGCAATTTATGGTCCAATAAAGGATTTTTATCTTCGAAACCCATTGGAAGGGGTAGTGCACGAGAAAAAAAATCATGCGGAAATGATCGACTCCTTGGATGCCCCAAAATACTATGAGTGTCGGAA

186_20190719_03_04_trnT(GUU)-comp-psbD-comp CATAGAGATGAAATTGGAACAAGTTGACCCCCTTTTCTATTTATTTTCATTTCTTTGGACTCCGCAAGAATTTGCCGATATTTCCAATTCAATCGTCTTGTCTTGTTCCTAGATGTTCTATAGGAAAAAATTGTCATTTCGTTCCTCTACGGAGAACCTTTTATTCTAAATTCTAAATCACAGGATCTAAATTCTAAATCACAGGATAAGAAAAAAATTCACTATCTTTCTTTGATTACAGGATCAAGATTCATTTANNNNTTATCTATACTATATAATATATTCNNNNNNTATAGATATTTCTATTCTATATTTAGATTTATAGCTATCAGATCGTGGCTTGATGTACCAAAAATTTCCATTTCGTTGCATCCAATATTTTTGTTCCGACCATCGTATGAAGAAAGCATGCAAGATAAATACTATCATTTCCAATCTCCTATTTTAATTTTAATATTGTANNNNNNTTGAAGTGAAGTAAAAATTTGGAAACCCTCTCTTTTCTAACAGGGAAAAATAAATCAAAAAATATTAGTAATTTAGTCTACATAAAAATTAGAATATAAAGAGAGTTCTTTTTCTTAATCTCATGAACAAGATCTAAGAATCCATTTAGTTGATGAAAGAACATGGGGGGGGGCAGGCCTGAGGATCAACCGGTAGTGGGTNNNNGGAAGGGGATTGCTTTTTCCTTGACCAATTCTTTCAAAAAACGAATCGGATTCATGGGTCATAATAAGATAATTCATGATTCAGATGCTTAATAATAAGATAATAAGAAGGAATAATCAAATTGAATTCATGAATTTACCCGGCAATTTATGGTCCAATAAAGGATTTTTATCTTCGAAACCCATTGGAAGGGGTAGTGCACGAGAAAAAAAATCATGCGGAAATGATCGACTCCTTGGATGCCCCAAAATACTATGAGTGTCGGAA

187_20190719_03_05_trnT(GUU)-comp-psbD-comp CATAGAGATGAAATTGGAACAAGTTGACCCCCTTTTCTATTTATTTTCATTTCTTTGGACTCCGCAAGAATTTGCCGATATTTCCAATTCAATCGTCTTGTCTTGTTCCTAGATGTTCTATAGGAAAAAATTGTCATTTCGTTCCTCTACGGAGAACCTTTTATTCTAAATTCTAAATCACAGGATCTAAATTCTAAATCACAGGATAAGAAAAAAATTCACTATCTTTCTTTGATTACAGGATCAAGATTCATTTANNNNTTATCTATACTATATAATATATTCNNNNNNTATAGATATTTCTATTCTATATTTAGATTTATAGCTATCAGATCGTGGCTTGATGTACCAAAAATTTCCATTTCGTTGCATCCAATATTTTTGTTCCGACCATCGTATGAAGAAAGCATGCAAGATAAATACTATCATTTCCAATCTCCTATTTTAATTTTAATATTGTANNNNNNTTGAAGTGAAGTAAAAATTTGGAAACCCTCTCTTTTCTAACAGGGAAAAATGAATCAAAAAATATTAGTAATTTAGTCTACATAAAAATTAGAATATAAAGAGAGTTCTTTTTCTTAATCTCATGAACAAGATCTAAGAATCCATTTAGTTGATGAAAGAACATGGGGGGGGGCAGGCCTGAGGATCAACCGGTAGTGGGTNNNNGGAAGGGGATTGCTTTTTCCTTGACCAATTCTTTCAAAAAACGAATCGGATTCATGGGTCATAATAAGATAATTCATGATTCAGATGCTTAATAATAAGATAATAAGAAGGAATAATCAAATTGAATTCATGAATTTACCCGGCAATTTATGGTCCAATAAAGGATTTTTATCTTCGAAACCCATTGGAAGGGGTAGTGCACGAGAAAAAAAATCATGCGGAAATGATCGACTCCTTGGATGCCCCAAAATACTATGAGTGTCGGAA

188_20190719_04_01_trnT(GUU)-comp-psbD-comp CATAGAGATGAAATTGGAACAAGTTGACCCCCTTTTCTATTTATTTTCATTTCTTTGGACTCCGCAAGAATTTGCCGATATTTCCAATTCAATCGTCTTGTCTTGTTCCTAGATGTTCTATAGGAAAAAATTGTCATTTCGTTCCTCTACGGAGAACCTTTTATTCTAAATTCTAAATCACAGGATCTAAATTCTAAATCACAGGATAAGAAAAAAATTCACTATCTTTCTTTGATTACAGGATCAAGATTCATTTANNNNTTATCTATACTATATAATATATTCNNNNNNTATAGATATTTCTATTCTATATTTAGATTTATAGCTATCAGATCGTGGCTTGATGTACCAAAAATTTCCATTTCGTTGCATCCAATATTTTTGTTCCGACCATCGTATGAAGAAAGCATGCAAGATAAATACTATCATTTCCAATCTCCTNNNNNNATTTTAATATTGTANNNNNNTTGAAGTGAAGTAAAAATTTGGAAACCCTCTCTTTTCTAACAGGGAAAAATAAATCAAAAAATATTAGTAATTTAGTCTACATAAAAATTAGAATATAAAGAGAGTTCTTTTTCTTAATCTCATGAACAAGATCTAAGAATCCATTTAGTTGATGAAAGAACATGGGGGGGGGCAGGCCTGAGGATCAACCGGTAGTGGGTNNNNGGAAGGGGATTGCTTTTTCCTTGACCAATTCTTTCAAAAAACGAATCGGATTCATGGGTCATAATAAGATAATTCATGATTCAGATGCTTAATAATAAGATAATAAGAAGGAATAATCAAATTGAATTCATGAATTTACCCGGCAATTTATGGTCCAATAAAGGATTTTTATCTTCGAAACCCATTGGAAGGGGTAGTGCACGAGAAAAAAAATCATGCGGAAATGATCGACTCCTTGGATGCCCCAAAATACTATGAGTGTCGGAA

189_20190719_04_02_trnT(GUU)-comp-psbD-comp CATAGAGATGAAATTGGAACAAGTTGACCCCCTTTTCTATTTATTTTCATTTCTTTGGACTCCGCAAGAATTTGCCGATATTTCCAATTCAATCGTCTTGTCTTGTTCCTAGATGTTCTATAGGAAAAAATTGTCATTTCGTTCCTCTACGGAGAACCTTTTATTCTAAATTCTAAATCACAGGATCTAAATTCTAAATCACAGGATAAGAAAAAAATTCACTATCTTTCTTTGATTACAGGATCAAGATTCATTTANNNNTTATCTATACTATATAATATATTCNNNNNNTATAGATATTTCTATTCTATATTTAGATTTATAGCTATCAGATCGTGGCTTGATGTACCAAAAATTTCCATTTCGTTGCATCCAATATTTTTGTTCCGACCATCGTATGAAGAAAGCATGCAAGATAAATACTATCATTTCCAATCTCCTNNNNNNATTTTAATATTGTANNNNNNTTGAAGTGAAGTAAAAATTTGGAAACCCTCTCTTTTCTAACAGGGAAAAATAAATCAAAAAATATTAGTAATTTAGTCTACATAAAAATTAGAATATAAAGAGAGTTCTTTTTCTTAATCTCATGAACAAGATCTAAGAATCCATTTAGTTGATGAAAGAACATGGGGGGGGGCAGGCCTGAGGATCAACCGGTAGTGGGTNNNNGGAAGGGGATTGCTTTTTCCTTGACCAATTCTTTCAAAAAACGAATCGGATTCATGGGTCATAATAAGATAATTCATGATTCAGATGCTTAATAATAAGATAATAAGAAGGAATAATCAAATTGAATTCATGAATTTACCCGGCAATTTATGGTCCAATAAAGGATTTTTATCTTCGAAACCCATTGGAAGGGGTAGTGCACGAGAAAAAAAATCATGCGGAAATGATCGACTCCTTGGATGCCCCAAAATACTATGAGTGTCGGAA

190_20190719_04_03_trnT(GUU)-comp-psbD-comp CATAGAGATGAAATTGGAACAAGTTGACCCCCTTTTCTATTTATTTTCATTTCTTTGGACTCCGCAAGAATTTGCCGATATTTCCAATTCAATCGTCTTGTCTTGTTCCTAGATGTTCTATAGGAAAAAATTGTCATTTCGTTCCTCTACGGAGAACCTTTTATTCTAAATTCTAAATCACAGGATCTAAATTCTAAATCACAGGATAAGAAAAAAATTCACTATCTTTCTTTGATTACAGGATCAAGATTCATTTANNNNTTATCTATACTATATAATATATTCNNNNNNTATAGATATTTCTATTCTATATTTATATTTATAGCTATCAGATCGTGGCTTGATGTACCAAAAATTTCCATTTCGTTGCATCCAATATTTTTGTTCCGACCATCGTATGAAGAAAGCATGCAAGATAAATACTATCATTTCCAATCTCCTATTTTAATTTTAATATTGTANNNNNNTTGAAGTGAAGTAAAAATTTGGAAACCCTCTCTTTTCTAACAGGGAAAAATAAATCAAAAAATATTAGTAATTTAGTCTACATAAAAATTAGAATATAAAGAGAGTTCTTTTTCTTAATCTCATGAACAAGATCTAAGAATCCATTTAGTTGATGAAAGAACATGGGGGGGGGCAGGCCTGAGGATCAACCGGTAGTGGGTNNNNGGAAGGGGATTGCTTTTTCCTTGACCAATTCTTTCAAAAAACGAATCGGATTCATGGGTCATAATAAGATAATTCATGATTCAGATGCTTAATAATAAGATAATAAGAAGGAATAATCAAATTGAATTCATGAATTTACCCGGCAATTTATGGTCCAATAAAGGATTTTTATCTTCGAAACCCATTGGAAGGGGTAGTGCACGAGAAAAAAAATCATGCGGAAATGATCGACTCCTTGGATGCCCCAAAATACTATGAGTGTCGGAA

191_20190720_01_01_trnT(GUU)-comp-psbD-comp CATAGAGATGAAATTGGAACAAGTTGACCCCCTTTTCTATTTATTTTCATTTCTTTGGACTCCGCAAGAATTTGCCGATATTTCCAATTCAATCGTCTTGTCTTGTTCCTAGATGTTCTATAGGAAAAAATTGTCATTTCGTTCCTCTACGGAGAACCTTTTATTCTAAATTCTAAATCACAGGATCTAAATTCTAAATCACAGGATAAGAAAAAAATTCACTATCTTTCTTTGATTACAGGATCAAGATTCATTTANNNNTTATCTATACTATATAATATATTCNNNNNNTATAGATATTTCTATTCTATATTTATATTTATAGCTATCAGATCGTGGCTTGATGTACCAAAAATTTCCATTTCGTTGCATCCAATATTTTTGTTCCGACCATCGTATGAAGAAAGCATGCAAGATAAATACTATCATTTCCAATCTCCTATTTTAATTTTAATATTGTANNNNNNTTGAAGTGAAGTAAAAATTTGGAAACCCTCTCTTTTCTAACAGGGAAAAATAAATCAAAAAATATTAGTAATTTAGTCTACATAAAAATTAGAATATAAAGAGAGTTCTTTTTCTTAATCTCATGAACAAGATCTAAGAATCCATTTAGTTGATGAAAGAACATGGGGGGGGGCAGGCCTGAGGATCAACCGGTAGTGGGTNNNNGGAAGGGGATTGCTTTTTCCTTGACCAATTCTTTCAAAAAACGAATCGGATTCATGGGTCATAATAAGATAATTCATGATTCAGATGCTTAATAATAAGATAATAAGAAGGAATAATCAAATTGAATTCATGAATTTACCCGGCAATTTATGGTCCAATAAAGGATTTTTATCTTCGAAACCCATTGGAAGGGGTAGTGCACGAGAAAAAAAATCATGCGGAAATGATCGACTCCTTGGATGCCCCAAAATACTATGAGTGTCGGAA

192_20190720_01_02_trnT(GUU)-comp-psbD-comp CATAGAGATGAAATTGGAACAAGTTGACCCCCTTTTCTATTTATTTTCATTTCTTTGGACTCCGCAAGAATTTGCCGATATTTCCAATTCAATCGTCTTGTCTTGTTCCTAGATGTTCTATAGGAAAAAATTGTCATTTCGTTCCTCTACGGAGAACCTTTTATTCTAAATTCTAAATCACAGGATCTAAATTCTAAATCACAGGATAAGAAAAAAATTCACTATCTTTCTTTGATTACAGGATCAAGATTCATTTANNNNTTATCTATACTATATAATATATTCNNNNNNTATAGATATTTCTATTCTATATTTATATTTATAGCTATCAGATCGTGGCTTGATGTACCAAAAATTTCCATTTCGTTGCATCCAATATTTTTGTTCCGACCATCGTATGAAGAAAGCATGCAAGATAAATACTATCATTTCCAATCTCCTATTTTAATTTTAATATTGTANNNNNNTTGAAGTGAAGTAAAAATTTGGAAACCCTCTCTTTTCTAACAGGGAAAAATAAATCAAAAAATATTAGTAATTTAGTCTACATAAAAATTAGAATATAAAGAGAGTTCTTTTTCTTAATCTCATGAACAAGATCTAAGAATCCATTTAGTTGATGAAAGAACATGGGGGGGGGCAGGCCTGAGGATCAACCGGTAGTGGGTNNNNGGAAGGGGATTGCTTTTTCCTTGACCAATTCTTTCAAAAAACGAATCGGATTCATGGGTCATAATAAGATAATTCATGATTCAGATGCTTAATAATAAGATAATAAGAAGGAATAATCAAATTGAATTCATGAATTTACCCGGCAATTTATGGTCCAATAAAGGATTTTTATCTTCGAAACCCATTGGAAGGGGTAGTGCACGAGAAAAAAAATCATGCGGAAATGATCGACTCCTTGGATGCCCCAAAATACTATGAGTGTCGGAA

193_20190720_01_03_trnT(GUU)-comp-psbD-comp CATAGAGATGAAATTGGAACAAGTTGACCCCCTTTTCTATTTATTTTCATTTCTTTGGACTCCGCAAGAATTTGCCGATATTTCCAATTCAATCGTCTTGTCTTGTTCCTAGATGTTCTATAGGAAAAAATTGTCATTTCGTTCCTCTACGGAGAACCTTTTATTCTAAATTCTAAATCACAGGATCTAAATTCTAAATCACAGGATAAGAAAAAAATTCACTATCTTTCTTTGATTACAGGATCAAGATTCATTTANNNNTTATCTATACTATATAATATATTCNNNNNNTATAGATATTTCTATTCTATATTTATATTTATAGCTATCAGATCGTGGCTTGATGTACCAAAAATTTCCATTTCGTTGCATCCAATATTTTTGTTCCGACCATCGTATGAAGAAAGCATGCAAGATAAATACTATCATTTCCAATCTCCTATTTTAATTTTAATATTGTANNNNNNTTGAAGTGAAGTAAAAATTTGGAAACCCTCTCTTTTCTAACAGGGAAAAATAAATCAAAAAATATTAGTAATTTAGTCTACATAAAAATTAGAATATAAAGAGAGTTCTTTTTCTTAATCTCATGAACAAGATCTAAGAATCCATTTAGTTGATGAAAGAACATGGGGGGGGGCAGGCCTGAGGATCAACCGGTAGTGGGTNNNNGGAAGGGGATTGCTTTTTCCTTGACCAATTCTTTCAAAAAACGAATCGGATTCATGGGTCATAATAAGATAATTCATGATTCAGATGCTTAATAATAAGATAATAAGAAGGAATAATCAAATTGAATTCATGAATTTACCCGGCAATTTATGGTCCAATAAAGGATTTTTATCTTCGAAACCCATTGGAAGGGGTAGTGCACGAGAAAAAAAATCATGCGGAAATGATCGACTCCTTGGATGCCCCAAAATACTATGAGTGTCGGAA

194_20190720_01_04_trnT(GUU)-comp-psbD-comp CATAGAGATGAAATTGGAACAAGTTGACCCCCTTTTCTATTTATTTTCATTTCTTTGGACTCCGCAAGAATTTGCCGATATTTCCAATTCAATCGTCTTGTCTTGTTCCTAGATGTTCTATAGGAAAAAATTGTCATTTCGTTCCTCTACGGAGAACCTTTTATTCTAAATTCTAAATCACAGGATCTAAATTCTAAATCACAGGATAAGAAAAAAATTCACTATCTTTCTTTGATTACAGGATCAAGATTCATTTANNNNTTATCTATACTATATAATATATTCNNNNNNTATAGATATTTCTATTCTATATTTATATTTATAGCTATCAGATCGTGGCTTGATGTACCAAAAATTTCCATTTCGTTGCATCCAATATTTTTGTTCCGACCATCGTATGAAGAAAGCATGCAAGATAAATACTATCATTTCCAATCTCCTATTTTAATTTTAATATTGTANNNNNNTTGAAGTGAAGTAAAAATTTGGAAACCCTCTCTTTTCTAACAGGGAAAAATAAATCAAAAAATATTAGTAATTTAGTCTACATAAAAATTAGAATATAAAGAGAGTTCTTTTTCTTAATCTCATGAACAAGATCTAAGAATCCATTTAGTTGATGAAAGAACATGGGGGGGGGCAGGCCTGAGGATCAACCGGTAGTGGGTNNNNGGAAGGGGATTGCTTTTTCCTTGACCAATTCTTTCAAAAAACGAATCGGATTCATGGGTCATAATAAGATAATTCATGATTCAGATGCTTAATAATAAGATAATAAGAAGGAATAATCAAATTGAATTCATGAATTTACCCGGCAATTTATGGTCCAATAAAGGATTTTTATCTTCGAAACCCATTGGAAGGGGTAGTGCACGAGAAAAAAAATCATGCGGAAATGATCGACTCCTTGGATGCCCCAAAATACTATGAGTGTCGGAA

195_20190720_01_05_trnT(GUU)-comp-psbD-comp CATAGAGATGAAATTGGAACAAGTTGACCCCCTTTTCTATTTATTTTCATTTCTTTGGACTCCGCAAGAATTTGCCGATATTTCCAATTCAATCGTCTTGTCTTGTTCCTAGATGTTCTATAGGAAAAAATTGTCATTTCGTTCCTCTACGGAGAACCTTTTATTCTAAATTCTAAATCACAGGATCTAAATTCTAAATCACAGGATAAGAAAAAAATTCACTATCTTTCTTTGATTACAGGATCAAGATTCATTTANNNNTTATCTATACTATATAATATATTCNNNNNNTATAGATATTTCTATTCTATATTTATATTTATAGCTATCAGATCGTGGCTTGATGTACCAAAAATTTCCATTTCGTTGCATCCAATATTTTTGTTCCGACCATCGTATGAAGAAAGCATGCAAGATAAATACTATCATTTCCAATCTCCTATTTTAATTTTAATATTGTANNNNNNTTGAAGTGAAGTAAAAATTTGGAAACCCTCTCTTTTCTAACAGGGAAAAATAAATCAAAAAATATTAGTAATTTAGTCTACATAAAAATTAGAATATAAAGAGAGTTCTTTTTCTTAATCTCATGAACAAGATCTAAGAATCCATTTAGTTGATGAAAGAACATGGGGGGGGGCAGGCCTGAGGATCAACCGGTAGTGGGTNNNNGGAAGGGGATTGCTTTTTCCTTGACCAATTCTTTCAAAAAACGAATCGGATTCATGGGTCATAATAAGATAATTCATGATTCAGATGCTTAATAATAAGATAATAAGAAGGAATAATCAAATTGAATTCATGAATTTACCCGGCAATTTATGGTCCAATAAAGGATTTTTATCTTCGAAACCCATTGGAAGGGGTAGTGCACGAGAAAAAAAATCATGCGGAAATGATCGACTCCTTGGATGCCCCAAAATACTATGAGTGTCGGAA

196_20190720_02_01_trnT(GUU)-comp-psbD-comp CATAGAGATGAAATTGGAACAAGTTGACCCCCTTTTCTATTTATTTTCATTTCTTTGGACTCCGCAAGAATTTGCCGATATTTCCAATTCAATCGTCTTGTCTTGTTCCTAGATGTTCTATAGGAAAAAATTGTCATTTCGTTCCTCTACGGAGAACCTTTTATTCTAAATTCTAAATCACAGGATCTAAATTCTAAATCACAGGATAAGAAAAAAATTCACTATCTTTCTTTGATTACAGGATCAAGATTCATTTANNNNTTATCTATACTATATAATATATTCNNNNNNTATAGATATTTCTATTCTATATTTAGATTTATAGCTATCAGATCGTGGCTTGATGTACCAAAAATTTCCATTTCGTTGCATCCAATATTTTTGTTCCGACCATCGTATGAAGAAAGCATGCAAGATAAATACTATCATTTCCAATCTCCTNNNNNNATTTTAATATTGTANNNNNNTTGAAGTGAAGTAAAAATTTGGAAACCCTCTCTTTTCTAACAGGGAAAAATAAATCAAAAAATATTAGTAATTTAGTCTACATAAAAATTAGAATATAAAGAGAGTTCTTTTTCTTAATCTCATGAACAAGATCTAAGAATCCATTTAGTTGATGAAAGAACATGGGGGGGGGCAGGCCTGAGGATCAACCGGTAGTGGGTNNNNGGAAGGGGATTGCTTTTTCCTTGACCAATTCTTTCAAAAAACGAATCGGATTCATGGGTCATAATAAGATAATTCATGATTCAGATGCTTAATAATAAGATAATAAGAAGGAATAATCAAATTGAATTCATGAATTTACCCGGCAATTTATGGTCCAATAAAGGATTTTTATCTTCGAAACCCATTGGAAGGGGTAGTGCACGAGAAAAAAAATCATGCGGAAATGATCGACTCCTTGGATGCCCCAAAATACTATGAGTGTCGGAA

197_20190720_02_02_trnT(GUU)-comp-psbD-comp CATAGAGATGAAATTGGAACAAGTTGACCCCCTTTTCTATTTATTTTCATTTCTTTGGACTCCGCAAGAATTTGCCGATATTTCCAATTCAATCGTCTTGTCTTGTTCCTAGATGTTCTATAGGAAAAAATTGTCATTTCGTTCCTCTACGGAGAACCTTTTATTCTAAATTCTAAATCACAGGATCTAAATTCTAAATCACAGGATAAGAAAAAAATTCACTATCTTTCTTTGATTACAGGATCAAGATTCATTTANNNNTTATCTATACTATATAATATATTCNNNNNNTATAGATATTTCTATTCTATATTTAGATTTATAGCTATCAGATCGTGGCTTGATGTACCAAAAATTTCCATTTCGTTGCATCCAATATTTTTGTTCCGACCATCGTATGAAGAAAGCATGCAAGATAAATACTATCATTTCCAATCTCCTNNNNNNATTTTAATATTGTANNNNNNTTGAAGTGAAGTAAAAATTTGGAAACCCTCTCTTTTCTAACAGGGAAAAATAAATCAAAAAATATTAGTAATTTAGTCTACATAAAAATTAGAATATAAAGAGAGTTCTTTTTCTTAATCTCATGAACAAGATCTAAGAATCCATTTAGTTGATGAAAGAACATGGGGGGGGGCAGGCCTGAGGATCAACCGGTAGTGGGTNNNNGGAAGGGGATTGCTTTTTCCTTGACCAATTCTTTCAAAAAACGAATCGGATTCATGGGTCATAATAAGATAATTCATGATTCAGATGCTTAATAATAAGATAATAAGAAGGAATAATCAAATTGAATTCATGAATTTACCCGGCAATTTATGGTCCAATAAAGGATTTTTATCTTCGAAACCCATTGGAAGGGGTAGTGCACGAGAAAAAAAATCATGCGGAAATGATCGACTCCTTGGATGCCCCAAAATACTATGAGTGTCGGAA

198_20190720_02_03_trnT(GUU)-comp-psbD-comp CATAGAGATGAAATTGGAACAAGTTGACCCCCTTTTCTATTTATTTTCATTTCTTTGGACTCCGCAAGAATTTGCCGATATTTCCAATTCAATCGTCTTGTCTTGTTCCTAGATGTTCTATAGGAAAAAATTGTCATTTCGTTCCTCTACGGAGAACCTTTTATTCTAAATTCTAAATCACAGGATCTAAATTCTAAATCACAGGATAAGAAAAAAATTCACTATCTTTCTTTGATTACAGGATCAAGATTCATTTANNNNTTATCTATACTATATAATATATTCNNNNNNTATAGATATTTCTATTCTATATTTATATTTATAGCTATCAGATCGTGGCTTGATGTACCAAAAATTTCCATTTCGTTGCATCCAATATTTTTGTTCCGACCATCGTATGAAGAAAGCATGCAAGATAAATACTATCATTTCCAATCTCCTATTTTAATTTTAATATTGTANNNNNNTTGAAGTGAAGTAAAAATTTGGAAACCCTCTCTTTTCTAACAGGGAAAAATAAATCAAAAAATATTAGTAATTTAGTCTACATAAAAATTAGAATATAAAGAGAGTTCTTTTTCTTAATCTCATGAACAAGATCTAAGAATCCATTTAGTTGATGAAAGAACATGGGGGGGGGCAGGCCTGAGGATCAACCGGTAGTGGGTNNNNGGAAGGGGATTGCTTTTTCCTTGACCAATTCTTTCAAAAAACGAATCGGATTCATGGGTCATAATAAGATAATTCATGATTCAGATGCTTAATAATAAGATAATAAGAAGGAATAATCAAATTGAATTCATGAATTTACCCGGCAATTTATGGTCCAATAAAGGATTTTTATCTTCGAAACCCATTGGAAGGGGTAGTGCACGAGAAAAAAAATCATGCGGAAATGATCGACTCCTTGGATGCCCCAAAATACTATGAGTGTCGGAA

199_20190720_02_04_trnT(GUU)-comp-psbD-comp CATAGAGATGAAATTGGAACAAGTTGACCCCCTTTTCTATTTATTTTCATTTCTTTGGACTCCGCAAGAATTTGCCGATATTTCCAATTCAATCGTCTTGTCTTGTTCCTAGATGTTCTATAGGAAAAAATTGTCATTTCGTTCCTCTACGGAGAACCTTTTATTCTAAATTCTAAATCACAGGATCTAAATTCTAAATCACAGGATAAGAAAAAAATTCACTATCTTTCTTTGATTACAGGATCAAGATTCATTTANNNNTTATCTATACTATATAATATATTCNNNNNNTATAGATATTTCTATTCTATATTTATATTTATAGCTATCAGATCGTGGCTTGATGTACCAAAAATTTCCATTTCGTTGCATCCAATATTTTTGTTCCGACCATCGTATGAAGAAAGCATGCAAGATAAATACTATCATTTCCAATCTCCTATTTTAATTTTAATATTGTANNNNNNTTGAAGTGAAGTAAAAATTTGGAAACCCTCTCTTTTCTAACAGGGAAAAATAAATCAAAAAATATTAGTAATTTAGTCTACATAAAAATTAGAATATAAAGAGAGTTCTTTTTCTTAATCTCATGAACAAGATCTAAGAATCCATTTAGTTGATGAAAGAACATGGGGGGGGGCAGGCCTGAGGATCAACCGGTAGTGGGTNNNNGGAAGGGGATTGCTTTTTCCTTGACCAATTCTTTCAAAAAACGAATCGGATTCATGGGTCATAATAAGATAATTCATGATTCAGATGCTTAATAATAAGATAATAAGAAGGAATAATCAAATTGAATTCATGAATTTACCCGGCAATTTATGGTCCAATAAAGGATTTTTATCTTCGAAACCCATTGGAAGGGGTAGTGCACGAGAAAAAAAATCATGCGGAAATGATCGACTCCTTGGATGCCCCAAAATACTATGAGTGTCGGAA

200_20190720_02_05_trnT(GUU)-comp-psbD-comp CATAGAGATGAAATTGGAACAAGTTGACCCCCTTTTCTATTTATTTTCATTTCTTTGGACTCCGCAAGAATTTGCCGATATTTCCAATTCAATCGTCTTGTCTTGTTCCTAGATGTTCTATAGGAAAAAATTGTCATTTCGTTCCTCTACGGAGAACCTTTTATTCTAAATTCTAAATCACAGGATCTAAATTCTAAATCACAGGATAAGAAAAAAATTCACTATCTTTCTTTGATTACAGGATCAAGATTCATTTANNNNTTATCTATACTATATAATATATTCNNNNNNTATAGATATTTCTATTCTATATTTATATTTATAGCTATCAGATCGTGGCTTGATGTACCAAAAATTTCCATTTCGTTGCATCCAATATTTTTGTTCCGACCATCGTATGAAGAAAGCATGCAAGATAAATACTATCATTTCCAATCTCCTATTTTAATTTTAATATTGTANNNNNNTTGAAGTGAAGTAAAAATTTGGAAACCCTCTCTTTTCTAACAGGGAAAAATAAATCAAAAAATATTAGTAATTTAGTCTACATAAAAATTAGAATATAAAGAGAGTTCTTTTTCTTAATCTCATGAACAAGATCTAAGAATCCATTTAGTTGATGAAAGAACATGGGGGGGGGCAGGCCTGAGGATCAACCGGTAGTGGGTNNNNGGAAGGGGATTGCTTTTTCCTTGACCAATTCTTTCAAAAAACGAATCGGATTCATGGGTCATAATAAGATAATTCATGATTCAGATGCTTAATAATAAGATAATAAGAAGGAATAATCAAATTGAATTCATGAATTTACCCGGCAATTTATGGTCCAATAAAGGATTTTTATCTTCGAAACCCATTGGAAGGGGTAGTGCACGAGAAAAAAAATCATGCGGAAATGATCGACTCCTTGGATGCCCCAAAATACTATGAGTGTCGGAA

201_20190720_03_01_trnT(GUU)-comp-psbD-comp CATAGAGATGAAATTGGAACAAGTTGACCCCCTTTTCTATTTATTTTCATTTCTTTGGACTCCGCAAGAATTTGCCGATATTTCCAATTCAATCGTCTTGTCTTGTTCCTAGATGTTCTATAGGAAAAAATTGTCATTTCGTTCCTCTACGGAGAACCTTTTATTCTAAATTCTAAATCACAGGATCTAAATTCTAAATCACAGGATAAGAAAAAAATTCACTATCTTTCTTTGATTACAGGATCAAGATTCATTTANNNNTTATCTATACTATATAATATATTCNNNNNNTATAGATATTTCTATTCTATATTTATATTTATAGCTATCAGATCGTGGCTTGATGTACCAAAAATTTCCATTTCGTTGCATCCAATATTTTTGTTCCGACCATCGTATGAAGAAAGCATGCAAGATAAATACTATCATTTCCAATCTCCTATTTTAATTTTAATATTGTANNNNNNTTGAAGTGAAGTAAAAATTTGGAAACCCTCTCTTTTCTAACAGGGAAAAATAAATCAAAAAATATTAGTAATTTAGTCTACATAAAAATTAGAATATAAAGAGAGTTCTTTTTCTTAATCTCATGAACAAGATCTAAGAATCCATTTAGTTGATGAAAGAACATGGGGGGGGGCAGGCCTGAGGATCAACCGGTAGTGGGTNNNNGGAAGGGGATTGCTTTTTCCTTGACCAATTCTTTCAAAAAACGAATCGGATTCATGGGTCATAATAAGATAATTCATGATTCAGATGCTTAATAATAAGATAATAAGAAGGAATAATCAAATTGAATTCATGAATTTACCCGGCAATTTATGGTCCAATAAAGGATTTTTATCTTCGAAACCCATTGGAAGGGGTAGTGCACGAGAAAAAAAATCATGCGGAAATGATCGACTCCTTGGATGCCCCAAAATACTATGAGTGTCGGAA

202_20190720_03_02_trnT(GUU)-comp-psbD-comp CATAGAGATGAAATTGGAACAAGTTGACCCCCTTTTCTATTTATTTTCATTTCTTTGGACTCCGCAAGAATTTGCCGATATTTCCAATTCAATCGTCTTGTCTTGTTCCTAGATGTTCTATAGGAAAAAATTGTCATTTCGTTCCTCTACGGAGAACCTTTTATTCTAAATTCTAAATCACAGGATCTAAATTCTAAATCACAGGATAAGAAAAAAATTCACTATCTTTCTTTGATTACAGGATCAAGATTCATTTANNNNTTATCTATACTATATAATATATTCNNNNNNTATAGATATTTCTATTCTATATTTATATTTATAGCTATCAGATCGTGGCTTGATGTACCAAAAATTTCCATTTCGTTGCATCCAATATTTTTGTTCCGACCATCGTATGAAGAAAGCATGCAAGATAAATACTATCATTTCCAATCTCCTATTTTAATTTTAATATTGTANNNNNNTTGAAGTGAAGTAAAAATTTGGAAACCCTCTCTTTTCTAACAGGGAAAAATAAATCAAAAAATATTAGTAATTTAGTCTACATAAAAATTAGAATATAAAGAGAGTTCTTTTTCTTAATCTCATGAACAAGATCTAAGAATCCATTTAGTTGATGAAAGAACATGGGGGGGGGCAGGCCTGAGGATCAACCGGTAGTGGGTNNNNGGAAGGGGATTGCTTTTTCCTTGACCAATTCTTTCAAAAAACGAATCGGATTCATGGGTCATAATAAGATAATTCATGATTCAGATGCTTAATAATAAGATAATAAGAAGGAATAATCAAATTGAATTCATGAATTTACCCGGCAATTTATGGTCCAATAAAGGATTTTTATCTTCGAAACCCATTGGAAGGGGTAGTGCACGAGAAAAAAAATCATGCGGAAATGATCGACTCCTTGGATGCCCCAAAATACTATGAGTGTCGGAA

203_20190720_03_03_trnT(GUU)-comp-psbD-comp CATAGAGATGAAATTGGAACAAGTTGACCCCCTTTTCTATTTATTTTCATTTCTTTGGACTCCGCAAGAATTTGCCGATATTTCCAATTCAATCGTCTTGTCTTGTTCCTAGATGTTCTATAGGAAAAAATTGTCATTTCGTTCCTCTACGGAGAACCTTTTATTCTAAATTCTAAATCACAGGATCTAAATTCTAAATCACAGGATAAGAAAAAAATTCACTATCTTTCTTTGATTACAGGATCAAGATTCATTTANNNNTTATCTATACTATATAATATATTCNNNNNNTATAGATATTTCTATTCTATATTTATATTTATAGCTATCAGATCGTGGCTTGATGTACCAAAAATTTCCATTTCGTTGCATCCAATATTTTTGTTCCGACCATCGTATGAAGAAAGCATGCAAGATAAATACTATCATTTCCAATCTCCTATTTTAATTTTAATATTGTANNNNNNTTGAAGTGAAGTAAAAATTTGGAAACCCTCTCTTTTCTAACAGGGAAAAATAAATCAAAAAATATTAGTAATTTAGTCTACATAAAAATTAGAATATAAAGAGAGTTCTTTTTCTTAATCTCATGAACAAGATCTAAGAATCCATTTAGTTGATGAAAGAACATGGGGGGGGGCAGGCCTGAGGATCAACCGGTAGTGGGTNNNNGGAAGGGGATTGCTTTTTCCTTGACCAATTCTTTCAAAAAACGAATCGGATTCATGGGTCATAATAAGATAATTCATGATTCAGATGCTTAATAATAAGATAATAAGAAGGAATAATCAAATTGAATTCATGAATTTACCCGGCAATTTATGGTCCAATAAAGGATTTTTATCTTCGAAACCCATTGGAAGGGGTAGTGCACGAGAAAAAAAATCATGCGGAAATGATCGACTCCTTGGATGCCCCAAAATACTATGAGTGTCGGAA

204_20190720_03_04_trnT(GUU)-comp-psbD-comp CATAGAGATGAAATTGGAACAAGTTGACCCCCTTTTCTATTTATTTTCATTTCTTTGGACTCCGCAAGAATTTGCCGATATTTCCAATTCAATCGTCTTGTCTTGTTCCTAGATGTTCTATAGGAAAAAATTGTCATTTCGTTCCTCTACGGAGAACCTTTTATTCTAAATTCTAAATCACAGGATCTAAATTCTAAATCACAGGATAAGAAAAAAATTCACTATCTTTCTTTGATTACAGGATCAAGATTCATTTANNNNTTATCTATACTATATAATATATTCNNNNNNTATAGATATTTCTATTCTATATTTATATTTATAGCTATCAGATCGTGGCTTGATGTACCAAAAATTTCCATTTCGTTGCATCCAATATTTTTGTTCCGACCATCGTATGAAGAAAGCATGCAAGATAAATACTATCATTTCCAATCTCCTATTTTAATTTTAATATTGTANNNNNNTTGAAGTGAAGTAAAAATTTGGAAACCCTCTCTTTTCTAACAGGGAAAAATAAATCAAAAAATATTAGTAATTTAGTCTACATAAAAATTAGAATATAAAGAGAGTTCTTTTTCTTAATCTCATGAACAAGATCTAAGAATCCATTTAGTTGATGAAAGAACATGGGGGGGGGCAGGCCTGAGGATCAACCGGTAGTGGGTNNNNGGAAGGGGATTGCTTTTTCCTTGACCAATTCTTTCAAAAAACGAATCGGATTCATGGGTCATAATAAGATAATTCATGATTCAGATGCTTAATAATAAGATAATAAGAAGGAATAATCAAATTGAATTCATGAATTTACCCGGCAATTTATGGTCCAATAAAGGATTTTTATCTTCGAAACCCATTGGAAGGGGTAGTGCACGAGAAAAAAAATCATGCGGAAATGATCGACTCCTTGGATGCCCCAAAATACTATGAGTGTCGGAA

205_20190720_03_05_trnT(GUU)-comp-psbD-comp CATAGAGATGAAATTGGAACAAGTTGACCCCCTTTTCTATTTATTTTCATTTCTTTGGACTCCGCAAGAATTTGCCGATATTTCCAATTCAATCGTCTTGTCTTGTTCCTAGATGTTCTATAGGAAAAAATTGTCATTTCGTTCCTCTACGGAGAACCTTTTATTCTAAATTCTAAATCACAGGATCTAAATTCTAAATCACAGGATAAGAAAAAAATTCACTATCTTTCTTTGATTACAGGATCAAGATTCATTTANNNNTTATCTATACTATATAATATATTCNNNNNNTATAGATATTTCTATTCTATATTTATATTTATAGCTATCAGATCGTGGCTTGATGTACCAAAAATTTCCATTTCGTTGCATCCAATATTTTTGTTCCGACCATCGTATGAAGAAAGCATGCAAGATAAATACTATCATTTCCAATCTCCTATTTTAATTTTAATATTGTANNNNNNTTGAAGTGAAGTAAAAATTTGGAAACCCTCTCTTTTCTAACAGGGAAAAATAAATCAAAAAATATTAGTAATTTAGTCTACATAAAAATTAGAATATAAAGAGAGTTCTTTTTCTTAATCTCATGAACAAGATCTAAGAATCCATTTAGTTGATGAAAGAACATGGGGGGGGGCAGGCCTGAGGATCAACCGGTAGTGGGTNNNNGGAAGGGGATTGCTTTTTCCTTGACCAATTCTTTCAAAAAACGAATCGGATTCATGGGTCATAATAAGATAATTCATGATTCAGATGCTTAATAATAAGATAATAAGAAGGAATAATCAAATTGAATTCATGAATTTACCCGGCAATTTATGGTCCAATAAAGGATTTTTATCTTCGAAACCCATTGGAAGGGGTAGTGCACGAGAAAAAAAATCATGCGGAAATGATCGACTCCTTGGATGCCCCAAAATACTATGAGTGTCGGAA

206_20190721_01_01_trnT(GUU)-comp-psbD-comp CATAGAGATGAAATTGGAACAAGTTGACCCCCTTTTCTATTTATTTTCATTTCTTTGGACTCCGCAAGAATTTGCCGATATTTCCAATTCAATCGTCTTGTCTTGTTCCTAGATGTTCTATAGGAAAAAATTGTCATTTCGTTCCTCTACGGAGAACCTTTTATNNNNNNNNNNNNNNNNNNNNNTCTAAATTCTAAATCACAGGATAAGAAAAAAATTCACTATCTTTCTTTGATTACAGGATCAAGATTCATTTANNNNTTATCTATACTATATAATATATTCNNNNNNTATAGATATTTCTATTCTATATTTATATTTATAGCTATCAGATCGTGGCTTGATGTACCAAAAATTTCCATTTCGTTGCATCCAATATTTTTGTTCCGACCATCGTATGAAGAAAGCATGCAAGATAAATACTATCATTTCCAATCTCCTATTTTAATTTTAATATTGTANNNNNNTTGAAGTGAAGTAAAAATTTGGAAACCCTCTCTTTTCTAACAGGGAAAAATAAATCAAAAAATATTAGTAATTTAGTCTACATAAAAATTAGAATATAAAGAGAGTTCTTTTTCTTAATCTCATGAACAAGATCTAAGAATCCATTTAGTTGATGAAAGAACATGGGGGGGGGCAGGCCTGAGGATCAACCGGTAGTGGGTNNNNGGAAGGGGATTGCTTTTTCCTTGACCAATTCTTTCAAAAAACGAATCGGATTCATGGGTCATAATAAGATAATTCATGATTCAGATGCTTAATAATAAGATAATAAGAAGGAATAATCAAATTGAATTCATGAATTTACCCGGCAATTTATGGTCCAATAAAGGATTTTTATCTTCGAAACCCATTGGAAGGGGTAGTGCACGAGAAAAAAAATCATGCGGAAATGATCGACTCCTTGGATGCCCCAAAATACTATGAGTGTCGGAA

207_20190721_01_02_trnT(GUU)-comp-psbD-comp CATAGAGATGAAATTGGAACAAGTTGACCCCCTTTTCTATTTATTTTCATTTCTTTGGACTCCGCAAGAATTTGCCGATATTTCCAATTCAATCGTCTTGTCTTGTTCCTAGATGTTCTATAGGAAAAAATTGTCATTTCGTTCCTCTACGGAGAACCTTTTATNNNNNNNNNNNNNNNNNNNNNTCTAAATTCTAAATCACAGGATAAGAAAAAAATTCACTATCTTTCTTTGATTACAGGATCAAGATTCATTTANNNNTTATCTATACTATATAATATATTCNNNNNNTATAGATATTTCTATTCTATATTTATATTTATAGCTATCAGATCGTGGCTTGATGTACCAAAAATTTCCATTTCGTTGCATCCAATATTTTTGTTCCGACCATCGTATGAAGAAAGCATGCAAGATAAATACTATCATTTCCAATCTCCTATTTTAATTTTAATATTGTANNNNNNTTGAAGTGAAGTAAAAATTTGGAAACCCTCTCTTTTCTAACAGGGAAAAATAAATCAAAAAATATTAGTAATTTAGTCTACATAAAAATTAGAATATAAAGAGAGTTCTTTTTCTTAATCTCATGAACAAGATCTAAGAATCCATTTAGTTGATGAAAGAACATGGGGGGGGGCAGGCCTGAGGATCAACCGGTAGTGGGTNNNNGGAAGGGGATTGCTTTTTCCTTGACCAATTCTTTCAAAAAACGAATCGGATTCATGGGTCATAATAAGATAATTCATGATTCAGATGCTTAATAATAAGATAATAAGAAGGAATAATCAAATTGAATTCATGAATTTACCCGGCAATTTATGGTCCAATAAAGGATTTTTATCTTCGAAACCCATTGGAAGGGGTAGTGCACGAGAAAAAAAATCATGCGGAAATGATCGACTCCTTGGATGCCCCAAAATACTATGAGTGTCGGAA

208_20190721_01_03_trnT(GUU)-comp-psbD-comp CATAGAGATGAAATTGGAACAAGTTGACCCCCTTTTCTATTTATTTTCATTTCTTTGGACTCCGCAAGAATTTGCCGATATTTCCAATTCAATCGTCTTGTCTTGTTCCTAGATGTTCTATAGGAAAAAATTGTCATTTCGTTCCTCTACGGAGAACCTTTTATTCTAAATTCTAAATCACAGGATCTAAATTCTAAATCACAGGATAAGAAAAAAATTCACTATCTTTCTTTGATTACAGGATCAAGATTCATTTANNNNTTATCTATACTATATAATATATTCNNNNNNTATAGATATTTCTATTCTATATTTATATTTATAGCTATCAGATCGTGGCTTGATGTACCAAAAATTTCCATTTCGTTGCATCCAATATTTTTGTTCCGACCATCGTATGAAGAAAGCATGCAAGATAAATACTATCATTTCCAATCTCCTATTTTAATTTTAATATTGTANNNNNNTTGAAGTGAAGTAAAAATTTGGAAACCCTCTCTTTTCTAACAGGGAAAAATAAATCAAAAAATATTAGTAATTTAGTCTACATAAAAATTAGAATATAAAGAGAGTTCTTTTTCTTAATCTCATGAACAAGATCTAAGAATCCATTTAGTTGATGAAAGAACATGGGGGGGGGCAGGCCTGAGGATCAACCGGTAGTGGGTNNNNGGAAGGGGATTGCTTTTTCCTTGACCAATTCTTTCAAAAAACGAATCGGATTCATGGGTCATAATAAGATAATTCATGATTCAGATGCTTAATAATAAGATAATAAGAAGGAATAATCAAATTGAATTCATGAATTTACCCGGCAATTTATGGTCCAATAAAGGATTTTTATCTTCGAAACCCATTGGAAGGGGTAGTGCACGAGAAAAAAAATCATGCGGAAATGATCGACTCCTTGGATGCCCCAAAATACTATGAGTGTCGGAA

209_20190721_01_04_trnT(GUU)-comp-psbD-comp CATAGAGATGAAATTGGAACAAGTTGACCCCCTTTTCTATTTATTTTCATTTCTTTGGACTCCGCAAGAATTTGCCGATATTTCCAATTCAATCGTCTTGTCTTGTTCCTAGATGTTCTATAGGAAAAAATTGTCATTTCGTTCCTCTACGGAGAACCTTTTATNNNNNNNNNNNNNNNNNNNNNTCTAAATTCTAAATCACAGGATAAGAAAAAAATTCACTATCTTTCTTTGATTACAGGATCAAGATTCATTTANNNNTTATCTATACTATATAATATATTCNNNNNNTATAGATATTTCTATTCTATATTTATATTTATAGCTATCAGATCGTGGCTTGATGTACCAAAAATTTCCATTTCGTTGCATCCAATATTTTTGTTCCGACCATCGTATGAAGAAAGCATGCAAGATAAATACTATCATTTCCAATCTCCTATTTTAATTTTAATATTGTANNNNNNTTGAAGTGAAGTAAAAATTTGGAAACCCTCTCTTTTCTAACAGGGAAAAATAAATCAAAAAATATTAGTAATTTAGTCTACATAAAAATTAGAATATAAAGAGAGTTCTTTTTCTTAATCTCATGAACAAGATCTAAGAATCCATTTAGTTGATGAAAGAACATGGGGGGGGGCAGGCCTGAGGATCAACCGGTAGTGGGTNNNNGGAAGGGGATTGCTTTTTCCTTGACCAATTCTTTCAAAAAACGAATCGGATTCATGGGTCATAATAAGATAATTCATGATTCAGATGCTTAATAATAAGATAATAAGAAGGAATAATCAAATTGAATTCATGAATTTACCCGGCAATTTATGGTCCAATAAAGGATTTTTATCTTCGAAACCCATTGGAAGGGGTAGTGCACGAGAAAAAAAATCATGCGGAAATGATCGACTCCTTGGATGCCCCAAAATACTATGAGTGTCGGAA

210_20190721_01_05_trnT(GUU)-comp-psbD-comp CATAGAGATGAAATTGGAACAAGTTGACCCCCTTTTCTATTTATTTTCATTTCTTTGGACTCCGCAAGAATTTGCCGATATTTCCAATTCAATCGTCTTGTCTTGTTCCTAGATGTTCTATAGGAAAAAATTGTCATTTCGTTCCTCTACGGAGAACCTTTTATNNNNNNNNNNNNNNNNNNNNNTCTAAATTCTAAATCACAGGATAAGAAAAAAATTCACTATCTTTCTTTGATTACAGGATCAAGATTCATTTANNNNTTATCTATACTATATAATATATTCNNNNNNTATAGATATTTCTATTCTATATTTATATTTATAGCTATCAGATCGTGGCTTGATGTACCAAAAATTTCCATTTCGTTGCATCCAATATTTTTGTTCCGACCATCGTATGAAGAAAGCATGCAAGATAAATACTATCATTTCCAATCTCCTATTTTAATTTTAATATTGTANNNNNNTTGAAGTGAAGTAAAAATTTGGAAACCCTCTCTTTTCTAACAGGGAAAAATAAATCAAAAAATATTAGTAATTTAGTCTACATAAAAATTAGAATATAAAGAGAGTTCTTTTTCTTAATCTCATGAACAAGATCTAAGAATCCATTTAGTTGATGAAAGAACATGGGGGGGGGCAGGCCTGAGGATCAACCGGTAGTGGGTNNNNGGAAGGGGATTGCTTTTTCCTTGACCAATTCTTTCAAAAAACGAATCGGATTCATGGGTCATAATAAGATAATTCATGATTCAGATGCTTAATAATAAGATAATAAGAAGGAATAATCAAATTGAATTCATGAATTTACCCGGCAATTTATGGTCCAATAAAGGATTTTTATCTTCGAAACCCATTGGAAGGGGTAGTGCACGAGAAAAAAAATCATGCGGAAATGATCGACTCCTTGGATGCCCCAAAATACTATGAGTGTCGGAA

211_20190722_01_01_trnT(GUU)-comp-psbD-comp CATAGAGATGAAATTGGAACAAGTTGACCCCCTTTTCTATTTATTTTCATTTCTTTGGACTCCGCAAGAATTTGCCGATATTTCCAATTCAATCGTCTTGTCTTGTTCCTAGATGTTCTATAGGAAAAAATTGTCATTTCGTTCCTCTACGGAGAACCTTTTATTCTAAATTCTAAATCACAGGATCTAAATTCTAAATCACAGGATAAGAAAAAAATTCACTATCTTTCTTTGATTACAGGATCAAGATTCATTTANNNNTTATCTATACTATATAATATATTCNNNNNNTATAGATATTTCTATTCTATATTTATATTTATAGCTATCAGATCGTGGCTTGATGTACCAAAAATTTCCATTTCGTTGCATCCAATATTTTTGTTCCGACCATCGTATGAAGAAAGCATGCAAGATAAATACTATCATTTCCAATCTCCTATTTTAATTTTAATATTGTANNNNNNTTGAAGTGAAGTAAAAATTTGGAAACCCTCTCTTTTCTAACAGGGAAAAATAAATCAAAAAATATTAGTAATTTAGTCTACATAAAAATTAGAATATAAAGAGAGTTCTTTTTCTTAATCTCATGAACAAGATCTAAGAATCCATTTAGTTGATGAAAGAACATGGGGGGGGGCAGGCCTGAGGATCAACCGGTAGTGGGTNNNNGGAAGGGGATTGCTTTTTCCTTGACCAATTCTTTCAAAAAACGAATCGGATTCATGGGTCATAATAAGATAATTCATGATTCAGATGCTTAATAATAAGATAATAAGAAGGAATAATCAAATTGAATTCATGAATTTACCCGGCAATTTATGGTCCAATAAAGGATTTTTATCTTCGAAACCCATTGGAAGGGGTAGTGCACGAGAAAAAAAATCATGCGGAAATGATCGACTCCTTGGATGCCCCAAAATACTATGAGTGTCGGAA

212_20190722_01_02_trnT(GUU)-comp-psbD-comp CATAGAGATGAAATTGGAACAAGTTGACCCCCTTTTCTATTTATTTTCATTTCTTTGGACTCCGCAAGAATTTGCCGATATTTCCAATTCAATCGTCTTGTCTTGTTCCTAGATGTTCTATAGGAAAAAATTGTCATTTCGTTCCTCTACGGAGAACCTTTTATTCTAAATTCTAAATCACAGGATCTAAATTCTAAATCACAGGATAAGAAAAAAATTCACTATCTTTCTTTGATTACAGGATCAAGATTCATTTANNNNTTATCTATACTATATAATATATTCNNNNNNTATAGATATTTCTATTCTATATTTATATTTATAGCTATCAGATCGTGGCTTGATGTACCAAAAATTTCCATTTCGTTGCATCCAATATTTTTGTTCCGACCATCGTATGAAGAAAGCATGCAAGATAAATACTATCATTTCCAATCTCCTATTTTAATTTTAATATTGTANNNNNNTTGAAGTGAAGTAAAAATTTGGAAACCCTCTCTTTTCTAACAGGGAAAAATAAATCAAAAAATATTAGTAATTTAGTCTACATAAAAATTAGAATATAAAGAGAGTTCTTTTTCTTAATCTCATGAACAAGATCTAAGAATCCATTTAGTTGATGAAAGAACATGGGGGGGGGCAGGCCTGAGGATCAACCGGTAGTGGGTNNNNGGAAGGGGATTGCTTTTTCCTTGACCAATTCTTTCAAAAAACGAATCGGATTCATGGGTCATAATAAGATAATTCATGATTCAGATGCTTAATAATAAGATAATAAGAAGGAATAATCAAATTGAATTCATGAATTTACCCGGCAATTTATGGTCCAATAAAGGATTTTTATCTTCGAAACCCATTGGAAGGGGTAGTGCACGAGAAAAAAAATCATGCGGAAATGATCGACTCCTTGGATGCCCCAAAATACTATGAGTGTCGGAA

213_20190722_01_03_trnT(GUU)-comp-psbD-comp CATAGAGATGAAATTGGAACAAGTTGACCCCCTTTTCTATTTATTTTCATTTCTTTGGACTCCGCAAGAATTTGCCGATATTTCCAATTCAATCGTCTTGTCTTGTTCCTAGATGTTCTATAGGAAAAAATTGTCATTTCGTTCCTCTACGGAGAACCTTTTATTCTAAATTCTAAATCACAGGATCTAAATTCTAAATCACAGGATAAGAAAAAAATTCACTATCTTTCTTTGATTACAGGATCAAGATTCATTTANNNNTTATCTATACTATATAATATATTCNNNNNNTATAGATATTTCTATTCTATATTTATATTTATAGCTATCAGATCGTGGCTTGATGTACCAAAAATTTCCATTTCGTTGCATCCAATATTTTTGTTCCGACCATCGTATGAAGAAAGCATGCAAGATAAATACTATCATTTCCAATCTCCTATTTTAATTTTAATATTGTANNNNNNTTGAAGTGAAGTAAAAATTTGGAAACCCTCTCTTTTCTAACAGGGAAAAATAAATCAAAAAATATTAGTAATTTAGTCTACATAAAAATTAGAATATAAAGAGAGTTCTTTTTCTTAATCTCATGAACAAGATCTAAGAATCCATTTAGTTGATGAAAGAACATGGGGGGGGGCAGGCCTGAGGATCAACCGGTAGTGGGTNNNNGGAAGGGGATTGCTTTTTCCTTGACCAATTCTTTCAAAAAACGAATCGGATTCATGGGTCATAATAAGATAATTCATGATTCAGATGCTTAATAATAAGATAATAAGAAGGAATAATCAAATTGAATTCATGAATTTACCCGGCAATTTATGGTCCAATAAAGGATTTTTATCTTCGAAACCCATTGGAAGGGGTAGTGCACGAGAAAAAAAATCATGCGGAAATGATCGACTCCTTGGATGCCCCAAAATACTATGAGTGTCGGAA

214_20190722_01_04_trnT(GUU)-comp-psbD-comp CATAGAGATGAAATTGGAACAAGTTGACCCCCTTTTCTATTTATTTTCATTTCTTTGGACTCCGCAAGAATTTGCCGATATTTCCAATTCAATCGTCTTGTCTTGTTCCTAGATGTTCTATAGGAAAAAATTGTCATTTCGTTCCTCTACGGAGAACCTTTTATTCTAAATTCTAAATCACAGGATCTAAATTCTAAATCACAGGATAAGAAAAAAATTCACTATCTTTCTTTGATTACAGGATCAAGATTCATTTANNNNTTATCTATACTATATAATATATTCNNNNNNTATAGATATTTCTATTCTATATTTATATTTATAGCTATCAGATCGTGGCTTGATGTACCAAAAATTTCCATTTCGTTGCATCCAATATTTTTGTTCCGACCATCGTATGAAGAAAGCATGCAAGATAAATACTATCATTTCCAATCTCCTATTTTAATTTTAATATTGTANNNNNNTTGAAGTGAAGTAAAAATTTGGAAACCCTCTCTTTTCTAACAGGGAAAAATAAATCAAAAAATATTAGTAATTTAGTCTACATAAAAATTAGAATATAAAGAGAGTTCTTTTTCTTAATCTCATGAACAAGATCTAAGAATCCATTTAGTTGATGAAAGAACATGGGGGGGGGCAGGCCTGAGGATCAACCGGTAGTGGGTNNNNGGAAGGGGATTGCTTTTTCCTTGACCAATTCTTTCAAAAAACGAATCGGATTCATGGGTCATAATAAGATAATTCATGATTCAGATGCTTAATAATAAGATAATAAGAAGGAATAATCAAATTGAATTCATGAATTTACCCGGCAATTTATGGTCCAATAAAGGATTTTTATCTTCGAAACCCATTGGAAGGGGTAGTGCACGAGAAAAAAAATCATGCGGAAATGATCGACTCCTTGGATGCCCCAAAATACTATGAGTGTCGGAA

215_20190722_01_05_trnT(GUU)-comp-psbD-comp CATAGAGATGAAATTGGAACAAGTTGACCCCCTTTTCTATTTATTTTCATTTCTTTGGACTCCGCAAGAATTTGCCGATATTTCCAATTCAATCGTCTTGTCTTGTTCCTAGATGTTCTATAGGAAAAAATTGTCATTTCGTTCCTCTACGGAGAACCTTTTATTCTAAATTCTAAATCACAGGATCTAAATTCTAAATCACAGGATAAGAAAAAAATTCACTATCTTTCTTTGATTACAGGATCAAGATTCATTTANNNNTTATCTATACTATATAATATATTCNNNNNNTATAGATATTTCTATTCTATATTTATATTTATAGCTATCAGATCGTGGCTTGATGTACCAAAAATTTCCATTTCGTTGCATCCAATATTTTTGTTCCGACCATCGTATGAAGAAAGCATGCAAGATAAATACTATCATTTCCAATCTCCTATTTTAATTTTAATATTGTANNNNNNTTGAAGTGAAGTAAAAATTTGGAAACCCTCTCTTTTCTAACAGGGAAAAATAAATCAAAAAATATTAGTAATTTAGTCTACATAAAAATTAGAATATAAAGAGAGTTCTTTTTCTTAATCTCATGAACAAGATCTAAGAATCCATTTAGTTGATGAAAGAACATGGGGGGGGGCAGGCCTGAGGATCAACCGGTAGTGGGTNNNNGGAAGGGGATTGCTTTTTCCTTGACCAATTCTTTCAAAAAACGAATCGGATTCATGGGTCATAATAAGATAATTCATGATTCAGATGCTTAATAATAAGATAATAAGAAGGAATAATCAAATTGAATTCATGAATTTACCCGGCAATTTATGGTCCAATAAAGGATTTTTATCTTCGAAACCCATTGGAAGGGGTAGTGCACGAGAAAAAAAATCATGCGGAAATGATCGACTCCTTGGATGCCCCAAAATACTATGAGTGTCGGAA

216_20190913_01_01_trnT(GUU)-comp-psbD-comp CATAGAGATGAAATTGGAACAAGTTGACCCCCTTTTCTATTTATTTTCATTTCTTTGGACTCCGCAAGAATTTGCCGATATTTCCAATTCAATCGTCTTGTCTTGTTCCTAGATGTTCTATAGGAAAAAATTGTCATTTCGTTCCTCTACGGAGAACCTTTTATTCTAAATTCTAAATCACAGGATCTAAATTCTAAATCACAGGATAAGAAAAAAATTCACTATCTTTCTTTGATTACAGGATCAAGATTCATTTANNNNTTATCTATACTATATAATATATTCNNNNNNTATAGATATTTCTATTCTATATTTAGATTTATAGCTATCAGATCGTGGCTTGATGTACCAAAAATTTCCATTTCGTTGCATCCAATATTTTTGTTCCGACCATCGTATGAAGAAAGCATGCAAGATAAATACTATCATTTCCAATCTCCTNNNNNNATTTTAATATTGTANNNNNNTTGAAGTGAAGTAAAAATTTGGAAACCCTCTCTTTTCTAACAGGGAAAAATAAATCAAAAAATATTAGTAATTTAGTCTACATAAAAATTAGAATATAAAGAGAGTTCTTTTTCTTAATCTCATGAACAAGATCTAAGAATCCATTTAGTTGATGAAAGAACATGGGGGGGGGCAGGCCTGAGGATCAACCGGTAGTGGGTNNNNGGAAGGGGATTGCTTTTTCCTTGACCAATTCTTTCAAAAAACGAATCGGATTCATGGGTCATAATAAGATAATTCATGATTCAGATGCTTAATAATAAGATAATAAGAAGGAATAATCAAATTGAATTCATGAATTTACCCGGCAATTTATGGTCCAATAAAGGATTTTTATCTTCGAAACCCATTGGAAGGGGTAGTGCACGAGAAAAAAAATCATGCGGAAATGATCGACTCCTTGGATGCCCCAAAATACTATGAGTGTCGGAA

217_20190913_01_04_trnT(GUU)-comp-psbD-comp CATAGAGATGAAATTGGAACAAGTTGACCCCCTTTTCTATTTATTTTCATTTCTTTGGACTCCGCAAGAATTTGCCGATATTTCCAATTCAATCGTCTTGTCTTGTTCCTAGATGTTCTATAGGAAAAAATTGTCATTTCGTTCCTCTACGGAGAACCTTTTATNNNNNNNNNNNNNNNNNNNNNTCTAAATTCTAAATCACAGGATAAGAAAAAAATTCACTATCTTTCTTTGATTACAGGATCAAGATTCATTTANNNNTTATCTATACTATATAATATATTCNNNNNNTATAGATATTTCTATTCTATATTTAGATTTATAGCTATCAGATCGTGGCTTGATGTACCAAAAATTTCCATTTCGTTGCATCCAATATTTTTGTTCCGACCATCGTATGAAGAAAGCATGCAAGATAAATACTATCATTTCCAATCTCCTATTTTAATTTTAATATTGTANNNNNNTTGAAGTGAAGTAAAAATTTGGAAACCCTCTCTTTTCTAACAGGGAAAAATAAATCAAAAAATATTAGTAATTTAGTCTACATAAAAATTAGAATATAAAGAGAGTTCTTTTTCTTAATCTCATGAACAAGATCTAAGAATCCATTTAGTTGATGAAAGAACATGGGGGGGGGCAGGCCTGAGGATCAACCGGTAGTGGGTNNNNGGAAGGGGATTGCTTTTTCCTTGACCAATTCTTTCAAAAAACGAATCGGATTCATGGGTCATAATAAGATAATTCATGATTCAGATGCTTAATAATAAGATAATAAGAAGGAATAATCAAATTGAATTCATGAATTTACCCGGCAATTTATGGTCCAATAAAGGATTTTTATCTTCGAAACCCATTGGAAGGGGTAGTGCACGAGAAAAAAAATCATGCGGAAATGATCGACTCCTTGGATGCCCCAAAATACTATGAGTGTCGGAA

218_20190913_01_05_trnT(GUU)-comp-psbD-comp CATAGAGATGAAATTGGAACAAGTTGACCCCCTTTTCTATTTATTTTCATTTCTTTGGACTCCGCAAGAATTTGCCGATATTTCCAATTCAATCGTCTTGTCTTGTTCCTAGATGTTCTATAGGAAAAAATTGTCATTTCGTTCCTCTACGGAGAACCTTTTATNNNNNNNNNNNNNNNNNNNNNTCTAAATTCTAAATCACAGGATAAGAAAAAAATTCACTATCTTTCTTTGATTACAGGATCAAGATTCATTTANNNNTTATCTATACTATATAATATATTCNNNNNNTATAGATATTTCTATTCTATATTTAGATTTATAGCTATCAGATCGTGGCTTGATGTACCAAAAATTTCCATTTCGTTGCATCCAATATTTTTGTTCCGACCATCGTATGAAGAAAGCATGCAAGATAAATACTATCATTTCCAATCTCCTATTTTAATTTTAATATTGTANNNNNNTTGAAGTGAAGTAAAAATTTGGAAACCCTCTCTTTTCTAACAGGGAAAAATAAATCAAAAAATATTAGTAATTTAGTCTACATAAAAATTAGAATATAAAGAGAGTTCTTTTTCTTAATCTCATGAACAAGATCTAAGAATCCATTTAGTTGATGAAAGAACATGGGGGGGGGCAGGCCTGAGGATCAACCGGTAGTGGGTNNNNGGAAGGGGATTGCTTTTTCCTTGACCAATTCTTTCAAAAAACGAATCGGATTCATGGGTCATAATAAGATAATTCATGATTCAGATGCTTAATAATAAGATAATAAGAAGGAATAATCAAATTGAATTCATGAATTTACCCGGCAATTTATGGTCCAATAAAGGATTTTTATCTTCGAAACCCATTGGAAGGGGTAGTGCACGAGAAAAAAAATCATGCGGAAATGATCGACTCCTTGGATGCCCCAAAATACTATGAGTGTCGGAA

219_20190913_01_06_trnT(GUU)-comp-psbD-comp CATAGAGATGAAATTGGAACAAGTTGACCCCCTTTTCTATTTATTTTCATTTCTTTGGACTCCGCAAGAATTTGCCGATATTTCCAATTCAATCGTCTTGTCTTGTTCCTAGATGTTCTATAGGAAAAAATTGTCATTTCGTTCCTCTACGGAGAACCTTTTATNNNNNNNNNNNNNNNNNNNNNTCTAAATTCTAAATCACAGGATAAGAAAAAAATTCACTATCTTTCTTTGATTACAGGATCAAGATTCATTTANNNNTTATCTATACTATATAATATATTCNNNNNNTATAGATATTTCTATTCTATATTTAGATTTATAGCTATCAGATCGTGGCTTGATGTACCAAAAATTTCCATTTCGTTGCATCCAATATTTTTGTTCCGACCATCGTATGAAGAAAGCATGCAAGATAAATACTATCATTTCCAATCTCCTATTTTAATTTTAATATTGTANNNNNNTTGAAGTGAAGTAAAAATTTGGAAACCCTCTCTTTTCTAACAGGGAAAAATAAATCAAAAAATATTAGTAATTTAGTCTACATAAAAATTAGAATATAAAGAGAGTTCTTTTTCTTAATCTCATGAACAAGATCTAAGAATCCATTTAGTTGATGAAAGAACATGGGGGGGGGCAGGCCTGAGGATCAACCGGTAGTGGGTNNNNGGAAGGGGATTGCTTTTTCCTTGACCAATTCTTTCAAAAAACGAATCGGATTCATGGGTCATAATAAGATAATTCATGATTCAGATGCTTAATAATAAGATAATAAGAAGGAATAATCAAATTGAATTCATGAATTTACCCGGCAATTTATGGTCCAATAAAGGATTTTTATCTTCGAAACCCATTGGAAGGGGTAGTGCACGAGAAAAAAAATCATGCGGAAATGATCGACTCCTTGGATGCCCCAAAATACTATGAGTGTCGGAA

220_20190913_01_07_trnT(GUU)-comp-psbD-comp CATAGAGATGAAATTGGAACAAGTTGACCCCCTTTTCTATTTATTTTCATTTCTTTGGACTCCGCAAGAATTTGCCGATATTTCCAATTCAATCGTCTTGTCTTGTTCCTAGATGTTCTATAGGAAAAAATTGTCATTTCGTTCCTCTACGGAGAACCTTTTATNNNNNNNNNNNNNNNNNNNNNTCTAAATTCTAAATCACAGGATAAGAAAAAAATTCACTATCTTTCTTTGATTACAGGATCAAGATTCATTTANNNNTTATCTATACTATATAATATATTCNNNNNNTATAGATATTTCTATTCTATATTTAGATTTATAGCTATCAGATCGTGGCTTGATGTACCAAAAATTTCCATTTCGTTGCATCCAATATTTTTGTTCCGACCATCGTATGAAGAAAGCATGCAAGATAAATACTATCATTTCCAATCTCCTATTTTAATTTTAATATTGTANNNNNNTTGAAGTGAAGTAAAAATTTGGAAACCCTCTCTTTTCTAACAGGGAAAAATAAATCAAAAAATATTAGTAATTTAGTCTACATAAAAATTAGAATATAAAGAGAGTTCTTTTTCTTAATCTCATGAACAAGATCTAAGAATCCATTTAGTTGATGAAAGAACATGGGGGGGGGCAGGCCTGAGGATCAACCGGTAGTGGGTNNNNGGAAGGGGATTGCTTTTTCCTTGACCAATTCTTTCAAAAAACGAATCGGATTCATGGGTCATAATAAGATAATTCATGATTCAGATGCTTAATAATAAGATAATAAGAAGGAATAATCAAATTGAATTCATGAATTTACCCGGCAATTTATGGTCCAATAAAGGATTTTTATCTTCGAAACCCATTGGAAGGGGTAGTGCACGAGAAAAAAAATCATGCGGAAATGATCGACTCCTTGGATGCCCCAAAATACTATGAGTGTCGGAA

221_20190913_03_01_trnT(GUU)-comp-psbD-comp CATAGAGATGAAATTGGAACAAGTTGACCCCCTTTTCTATTTATTTTCATTTCTTTGGACTCCGCAAGAATTTGCCGATATTTCCAATTCAATCGTCTTGTCTTGTTCCTAGATGTTCTATAGGAAAAAATTGTCATTTCGTTCCTCTACGGAGAACCTTTTATNNNNNNNNNNNNNNNNNNNNNTCTAAATTCTAAATCACAGGATAAGAAAAAAATTCACTATCTTTCTTTGATTACAGGATCAAGATTCATTTANNNNTTATCTATACTATATAATATATTCNNNNNNTATAGATATTTCTATTCTATATTTAGATTTATAGCTATCAGATCGTGGCTTGATGTACCAAAAATTTCCATTTCGTTGCATCCAATATTTTTGTTCCGACCATCGTATGAAGAAAGCATGCAAGATAAATACTATCATTTCCAATCTCCTATTTTAATTTTAATATTGTANNNNNNTTGAAGTGAAGTAAAAATTTGGAAACCCTCTCTTTTCTAACAGGGAAAAATAAATCAAAAAATATTAGTAATTTAGTCTACATAAAAATTAGAATATAAAGAGAGTTCTTTTTCTTAATCTCATGAACAAGATCTAAGAATCCATTTAGTTGATGAAAGAACATGGGGGGGGGCAGGCCTGAGGATCAACCGGTAGTGGGTNNNNGGAAGGGGATTGCTTTTTCCTTGACCAATTCTTTCAAAAAACGAATCGGATTCATGGGTCATAATAAGATAATTCATGATTCAGATGCTTAATAATAAGATAATAAGAAGGAATAATCAAATTGAATTCATGAATTTACCCGGCAATTTATGGTCCAATAAAGGATTTTTATCTTCGAAACCCATTGGAAGGGGTAGTGCACGAGAAAAAAAATCATGCGGAAATGATCGACTCCTTGGATGCCCCAAAATACTATGAGTGTCGGAA

222_20190913_03_04_trnT(GUU)-comp-psbD-comp CATAGAGATGAAATTGGAACAAGTTGACCCCCTTTTCTATTTATTTTCATTTCTTTGGACTCCGCAAGAATTTGCCGATATTTCCAATTCAATCGTCTTGTCTTGTTCCTAGATGTTCTATAGGAAAAAATTGTCATTTCGTTCCTCTACGGAGAACCTTTTATNNNNNNNNNNNNNNNNNNNNNTCTAAATTCTAAATCACAGGATAAGAAAAAAATTCACTATCTTTCTTTGATTACAGGATCAAGATTCATTTANNNNTTATCTATACTATATAATATATTCNNNNNNTATAGATATTTCTATTCTATATTTAGATTTATAGCTATCAGATCGTGGCTTGATGTACCAAAAATTTCCATTTCGTTGCATCCAATATTTTTGTTCCGACCATCGTATGAAGAAAGCATGCAAGATAAATACTATCATTTCCAATCTCCTATTTTAATTTTAATATTGTANNNNNNTTGAAGTGAAGTAAAAATTTGGAAACCCTCTCTTTTCTAACAGGGAAAAATAAATCAAAAAATATTAGTAATTTAGTCTACATAAAAATTAGAATATAAAGAGAGTTCTTTTTCTTAATCTCATGAACAAGATCTAAGAATCCATTTAGTTGATGAAAGAACATGGGGGGGGGCAGGCCTGAGGATCAACCGGTAGTGGGTNNNNGGAAGGGGATTGCTTTTTCCTTGACCAATTCTTTCAAAAAACGAATCGGATTCATGGGTCATAATAAGATAATTCATGATTCAGATGCTTAATAATAAGATAATAAGAAGGAATAATCAAATTGAATTCATGAATTTACCCGGCAATTTATGGTCCAATAAAGGATTTTTATCTTCGAAACCCATTGGAAGGGGTAGTGCACGAGAAAAAAAATCATGCGGAAATGATCGACTCCTTGGATGCCCCAAAATACTATGAGTGTCGGAA

223_20190913_03_05_trnT(GUU)-comp-psbD-comp CATAGAGATGAAATTGGAACAAGTTGACCCCCTTTTCTATTTATTTTCATTTCTTTGGACTCCGCAAGAATTTGCCGATATTTCCAATTCAATCGTCTTGTCTTGTTCCTAGATGTTCTATAGGAAAAAATTGTCATTTCGTTCCTCTACGGAGAACCTTTTATNNNNNNNNNNNNNNNNNNNNNTCTAAATTCTAAATCACAGGATAAGAAAAAAATTCACTATCTTTCTTTGATTACAGGATCAAGATTCATTTANNNNTTATCTATACTATATAATATATTCNNNNNNTATAGATATTTCTATTCTATATTTAGATTTATAGCTATCAGATCGTGGCTTGATGTACCAAAAATTTCCATTTCGTTGCATCCAATATTTTTGTTCCGACCATCGTATGAAGAAAGCATGCAAGATAAATACTATCATTTCCAATCTCCTATTTTAATTTTAATATTGTANNNNNNTTGAAGTGAAGTAAAAATTTGGAAACCCTCTCTTTTCTAACAGGGAAAAATAAATCAAAAAATATTAGTAATTTAGTCTACATAAAAATTAGAATATAAAGAGAGTTCTTTTTCTTAATCTCATGAACAAGATCTAAGAATCCATTTAGTTGATGAAAGAACATGGGGGGGGGCAGGCCTGAGGATCAACCGGTAGTGGGTNNNNGGAAGGGGATTGCTTTTTCCTTGACCAATTCTTTCAAAAAACGAATCGGATTCATGGGTCATAATAAGATAATTCATGATTCAGATGCTTAATAATAAGATAATAAGAAGGAATAATCAAATTGAATTCATGAATTTACCCGGCAATTTATGGTCCAATAAAGGATTTTTATCTTCGAAACCCATTGGAAGGGGTAGTGCACGAGAAAAAAAATCATGCGGAAATGATCGACTCCTTGGATGCCCCAAAATACTATGAGTGTCGGAA

224_20190913_03_06_trnT(GUU)-comp-psbD-comp CATAGAGATGAAATTGGAACAAGTTGACCCCCTTTTCTATTTATTTTCATTTCTTTGGACTCCGCAAGAATTTGCCGATATTTCCAATTCAATCGTCTTGTCTTGTTCCTAGATGTTCTATAGGAAAAAATTGTCATTTCGTTCCTCTACGGAGAACCTTTTATNNNNNNNNNNNNNNNNNNNNNTCTAAATTCTAAATCACAGGATAAGAAAAAAATTCACTATCTTTCTTTGATTACAGGATCAAGATTCATTTANNNNTTATCTATACTATATAATATATTCNNNNNNTATAGATATTTCTATTCTATATTTAGATTTATAGCTATCAGATCGTGGCTTGATGTACCAAAAATTTCCATTTCGTTGCATCCAATATTTTTGTTCCGACCATCGTATGAAGAAAGCATGCAAGATAAATACTATCATTTCCAATCTCCTATTTTAATTTTAATATTGTANNNNNNTTGAAGTGAAGTAAAAATTTGGAAACCCTCTCTTTTCTAACAGGGAAAAATAAATCAAAAAATATTAGTAATTTAGTCTACATAAAAATTAGAATATAAAGAGAGTTCTTTTTCTTAATCTCATGAACAAGATCTAAGAATCCATTTAGTTGATGAAAGAACATGGGGGGGGGCAGGCCTGAGGATCAACCGGTAGTGGGTNNNNGGAAGGGGATTGCTTTTTCCTTGACCAATTCTTTCAAAAAACGAATCGGATTCATGGGTCATAATAAGATAATTCATGATTCAGATGCTTAATAATAAGATAATAAGAAGGAATAATCAAATTGAATTCATGAATTTACCCGGCAATTTATGGTCCAATAAAGGATTTTTATCTTCGAAACCCATTGGAAGGGGTAGTGCACGAGAAAAAAAATCATGCGGAAATGATCGACTCCTTGGATGCCCCAAAATACTATGAGTGTCGGAA

225_20190913_03_07_trnT(GUU)-comp-psbD-comp CATAGAGATGAAATTGGAACAAGTTGACCCCCTTTTCTATTTATTTTCATTTCTTTGGACTCCGCAAGAATTTGCCGATATTTCCAATTCAATCGTCTTGTCTTGTTCCTAGATGTTCTATAGGAAAAAATTGTCATTTCGTTCCTCTACGGAGAACCTTTTATNNNNNNNNNNNNNNNNNNNNNTCTAAATTCTAAATCACAGGATAAGAAAAAAATTCACTATCTTTCTTTGATTACAGGATCAAGATTCATTTANNNNTTATCTATACTATATAATATATTCNNNNNNTATAGATATTTCTATTCTATATTTAGATTTATAGCTATCAGATCGTGGCTTGATGTACCAAAAATTTCCATTTCGTTGCATCCAATATTTTTGTTCCGACCATCGTATGAAGAAAGCATGCAAGATAAATACTATCATTTCCAATCTCCTATTTTAATTTTAATATTGTANNNNNNTTGAAGTGAAGTAAAAATTTGGAAACCCTCTCTTTTCTAACAGGGAAAAATAAATCAAAAAATATTAGTAATTTAGTCTACATAAAAATTAGAATATAAAGAGAGTTCTTTTTCTTAATCTCATGAACAAGATCTAAGAATCCATTTAGTTGATGAAAGAACATGGGGGGGGGCAGGCCTGAGGATCAACCGGTAGTGGGTNNNNGGAAGGGGATTGCTTTTTCCTTGACCAATTCTTTCAAAAAACGAATCGGATTCATGGGTCATAATAAGATAATTCATGATTCAGATGCTTAATAATAAGATAATAAGAAGGAATAATCAAATTGAATTCATGAATTTACCCGGCAATTTATGGTCCAATAAAGGATTTTTATCTTCGAAACCCATTGGAAGGGGTAGTGCACGAGAAAAAAAATCATGCGGAAATGATCGACTCCTTGGATGCCCCAAAATACTATGAGTGTCGGAA

226_20190914_01_01_trnT(GUU)-comp-psbD-comp CATAGAGATGAAATTGGAACAAGTTGACCCCCTTTTCTATTTATTTTCATTTCTTTGGACTCCGCAAGAATTTGCCGATATTTCCAATTCAATCGTCTTGTCTTGTTCCTAGATGTTCTATAGGAAAAAATTGTCATTTCGTTCCTCTACGGAGAACCTTTTATTCTAAATTCTAAATCACAGGATCTAAATTCTAAATCACAGGATAAGAAAAAAATTCACTATCTTTCTTTGATTACAGGATCAAGATTCATTTANNNNTTATCTATACTATATAATATATTCNNNNNNTATAGATATTTCTATTCTATATTTAGATTTATAGCTATCAGATCGTGGCTTGATGTACCAAAAATTTCCATTTCGTTGCATCCAATATTTTTGTTCCGACCATCGTATGAAGAAAGCATGCAAGATAAATACTATCATTTCCAATCTCCTATTTTAATTTTAATATTGTANNNNNNTTGAAGTGAAGTAAAAATTTGGAAACCCTCTCTTTTCTAACAGGGAAAAATAAATCAAAAAATATTAGTAATTTAGTCTACATAAAAATTAGAATATAAAGAGAGTTCTTTTTCTTAATCTCATGAACAAGATCTAAGAATCCATTTAGTTGATGAAAGAACATGGGGGGGGGCAGGCCTGAGGATCAACCGGTAGTGGGTNNNNGGAAGGGGATTGCTTTTTCCTTGACCAATTCTTTCAAAAAACGAATCGGATTCATGGGTCATAATAAGATAATTCATGATTCAGATGCTTAATAATAAGATAATAAGAAGGAATAATCAAATTGAATTCATGAATTTACCCGGCAATTTATGGTCCAATAAAGGATTTTTATCTTCGAAACCCATTGGAAGGGGTAGTGCACGAGAAAAAAAATCATGCGGAAATGATCGACTCCTTGGATGCCCCAAAATACTATGAGTGTCGGAA

227_20190914_01_02_trnT(GUU)-comp-psbD-comp CATAGAGATGAAATTGGAACAAGTTGACCCCCTTTTCTATTTATTTTCATTTCTTTGGACTCCGCAAGAATTTGCCGATATTTCCAATTCAATCGTCTTGTCTTGTTCCTAGATGTTCTATAGGAAAAAATTGTCATTTCGTTCCTCTACGGAGAACCTTTTATTCTAAATTCTAAATCACAGGATCTAAATTCTAAATCACAGGATAAGAAAAAAATTCACTATCTTTCTTTGATTACAGGATCAAGATTCATTTANNNNTTATCTATACTATATAATATATTCNNNNNNTATAGATATTTCTATTCTATATTTAGATTTATAGCTATCAGATCGTGGCTTGATGTACCAAAAATTTCCATTTCGTTGCATCCAATATTTTTGTTCCGACCATCGTATGAAGAAAGCATGCAAGATAAATACTATCATTTCCAATCTCCTATTTTAATTTTAATATTGTANNNNNNTTGAAGTGAAGTAAAAATTTGGAAACCCTCTCTTTTCTAACAGGGAAAAATAAATCAAAAAATATTAGTAATTTAGTCTACATAAAAATTAGAATATAAAGAGAGTTCTTTTTCTTAATCTCATGAACAAGATCTAAGAATCCATTTAGTTGATGAAAGAACATGGGGGGGGGCAGGCCTGAGGATCAACCGGTAGTGGGTNNNNGGAAGGGGATTGCTTTTTCCTTGACCAATTCTTTCAAAAAACGAATCGGATTCATGGGTCATAATAAGATAATTCATGATTCAGATGCTTAATAATAAGATAATAAGAAGGAATAATCAAATTGAATTCATGAATTTACCCGGCAATTTATGGTCCAATAAAGGATTTTTATCTTCGAAACCCATTGGAAGGGGTAGTGCACGAGAAAAAAAATCATGCGGAAATGATCGACTCCTTGGATGCCCCAAAATACTATGAGTGTCGGAA

228_20190914_01_03_trnT(GUU)-comp-psbD-comp CATAGAGATGAAATTGGAACAAGTTGACCCCCTTTTCTATTTATTTTCATTTCTTTGGACTCCGCAAGAATTTGCCGATATTTCCAATTCAATCGTCTTGTCTTGTTCCTAGATGTTCTATAGGAAAAAATTGTCATTTCGTTCCTCTACGGAGAACCTTTTATTCTAAATTCTAAATCACAGGATCTAAATTCTAAATCACAGGATAAGAAAAAAATTCACTATCTTTCTTTGATTACAGGATCAAGATTCATTTANNNNTTATCTATACTATATAATATATTCNNNNNNTATAGATATTTCTATTCTATATTTAGATTTATAGCTATCAGATCGTGGCTTGATGTACCAAAAATTTCCATTTCGTTGCATCCAATATTTTTGTTCCGACCATCGTATGAAGAAAGCATGCAAGATAAATACTATCATTTCCAATCTCCTATTTTAATTTTAATATTGTANNNNNNTTGAAGTGAAGTAAAAATTTGGAAACCCTCTCTTTTCTAACAGGGAAAAATAAATCAAAAAATATTAGTAATTTAGTCTACATAAAAATTAGAATATAAAGAGAGTTCTTTTTCTTAATCTCATGAACAAGATCTAAGAATCCATTTAGTTGATGAAAGAACATGGGGGGGGGCAGGCCTGAGGATCAACCGGTAGTGGGTNNNNGGAAGGGGATTGCTTTTTCCTTGACCAATTCTTTCAAAAAACGAATCGGATTCATGGGTCATAATAAGATAATTCATGATTCAGATGCTTAATAATAAGATAATAAGAAGGAATAATCAAATTGAATTCATGAATTTACCCGGCAATTTATGGTCCAATAAAGGATTTTTATCTTCGAAACCCATTGGAAGGGGTAGTGCACGAGAAAAAAAATCATGCGGAAATGATCGACTCCTTGGATGCCCCAAAATACTATGAGTGTCGGAA

229_20190914_01_04_trnT(GUU)-comp-psbD-comp CATAGAGATGAAATTGGAACAAGTTGACCCCCTTTTCTATTTATTTTCATTTCTTTGGACTCCGCAAGAATTTGCCGATATTTCCAATTCAATCGTCTTGTCTTGTTCCTAGATGTTCTATAGGAAAAAATTGTCATTTCGTTCCTCTACGGAGAACCTTTTATNNNNNNNNNNNNNNNNNNNNNTCTAAATTCTAAATCACAGGATAAGAAAAAAATTCACTATCTTTCTTTGATTACAGGATCAAGATTCATTTANNNNTTATCTATACTATATAATATATTCNNNNNNTATAGATATTTCTATTCTATATTTAGATTTATAGCTATCAGATCGTGGCTTGATGTACCAAAAATTTCCATTTCGTTGCATCCAATATTTTTGTTCCGACCATCGTATGAAGAAAGCATGCAAGATAAATACTATCATTTCCAATCTCCTATTTTAATTTTAATATTGTANNNNNNTTGAAGTGAAGTAAAAATTTGGAAACCCTCTCTTTTCTAACAGGGAAAAATAAATCAAAAAATATTAGTAATTTAGTCTACATAAAAATTAGAATATAAAGAGAGTTCTTTTTCTTAATCTCATGAACAAGATCTAAGAATCCATTTAGTTGATGAAAGAACATGGGGGGGGGCAGGCCTGAGGATCAACCGGTAGTGGGTNNNNGGAAGGGGATTGCTTTTTCCTTGACCAATTCTTTCAAAAAACGAATCGGATTCATGGGTCATAATAAGATAATTCATGATTCAGATGCTTAATAATAAGATAATAAGAAGGAATAATCAAATTGAATTCATGAATTTACCCGGCAATTTATGGTCCAATAAAGGATTTTTATCTTCGAAACCCATTGGAAGGGGTAGTGCACGAGAAAAAAAATCATGCGGAAATGATCGACTCCTTGGATGCCCCAAAATACTATGAGTGTCGGAA

230_20190914_01_05_trnT(GUU)-comp-psbD-comp CATAGAGATGAAATTGGAACAAGTTGACCCCCTTTTCTATTTATTTTCATTTCTTTGGACTCCGCAAGAATTTGCCGATATTTCCAATTCAATCGTCTTGTCTTGTTCCTAGATGTTCTATAGGAAAAAATTGTCATTTCGTTCCTCTACGGAGAACCTTTTATTCTAAATTCTAAATCACAGGATCTAAATTCTAAATCACAGGATAAGAAAAAAATTCACTATCTTTCTTTGATTACAGGATCAAGATTCATTTANNNNTTATCTATACTATATAATATATTCNNNNNNTATAGATATTTCTATTCTATATTTAGATTTATAGCTATCAGATCGTGGCTTGATGTACCAAAAATTTCCATTTCGTTGCATCCAATATTTTTGTTCCGACCATCGTATGAAGAAAGCATGCAAGATAAATACTATCATTTCCAATCTCCTATTTTAATTTGAATATTGTANNNNNNTTGAAGTGAAGTAAAAATTTGGAAACCCTCTCTTTTCTAACAGGGAAAAATAAATCAAAAAATATTAGTAATTTAGTCTACATAAAAATTAGAATATAAAGAGAGTTCTTTTTCTTAATCTCATGAACAAGATCTAAGAATCCATTTAGTTGATGAAAGAACATGGGGGGGGGCAGGCCTGAGGATCAACCGGTAGTGGGTNNNNGGAAGGGGATTGCTTTTTCCTTGACCAATTCTTTCAAAAAACGAATCGGATTCATGGGTCATAATAAGATAATTCATGATTCAGATGCTTAATAATAAGATAATAAGAAGGAATAATCAAATTGAATTCATGAATTTACCCGGCAATTTATGGTCCAATAAAGGATTTTTATCTTCGAAACCCATTGGAAGGGGTAGTGCACGAGAAAAAAAATCATGCGGAAATGATCGACTCCTTGGATGCCCCAAAATACTATGAGTGTCGGAA

231_20190915_01_01_trnT(GUU)-comp-psbD-comp CATAGAGATGAAATTGGAACAAGTTGACCCCCTTTTCTATTTATTTTCATTTCTTTGGACTCCGCAAGAATTTGCCGATATTTCCAATTCAATCGTCTTGTCTTGTTCCTAGATGTTCTATAGGAAAAAATTGTCATTTCGTTCCTCTACGGAGAACCTTTTATTCTAAATTCTAAATCACAGGATCTAAATTCTAAATCACAGGATAAGAAAAAAATTCACTATCTTTCTTTGATTACAGGATCAAGATTCATTTANNNNTTATCTATACTATATAATATATTCNNNNNNTATAGATATTTCTATTCTATATTTAGATTTATAGCTATCAGATCGTGGCTTGATGTACCAAAAATTTCCATTTCGTTGCATCCAATATTTTTGTTCCGACCATCGTATGAAGAAAGCATGCAAGATAAATACTATCATTTCCAATCTCCTATTTTAATTTTAATATTGTANNNNNNTTGAAGTGAAGTAAAAATTTGGAAACCCTCTCTTTTCTAACAGGGAAAAATAAATCAAAAAATATTAGTAATTTAGTCTACATAAAAATTAGAATATAAAGAGAGTTCTTTTTCTTAATCTCATGAACAAGATCTAAGAATCCATTTAGTTGATGAAAGAACATGGGGGGGGGCAGGCCTGAGGATCAACCGGTAGTGGGTNNNNGGAAGGGGATTGCTTTTTCCTTGACCAATTCTTTCAAAAAACGAATCGGATTCATGGGTCATAATAAGATAATTCATGATTCAGATGCTTAATAATAAGATAATAAGAAGGAATAATCAAATTGAATTCATGAATTTACCCGGCAATTTATGGTCCAATAAAGGATTTTTATCTTCGAAACCCATTGGAAGGGGTAGTGCACGAGAAAAAAAATCATGCGGAAATGATCGACTCCTTGGATGCCCCAAAATACTATGAGTGTCGGAA

232_20190915_01_02_trnT(GUU)-comp-psbD-comp CATAGAGATGAAATTGGAACAAGTTGACCCCCTTTTCTATTTATTTTCATTTCTTTGGACTCCGCAAGAATTTGCCGATATTTCCAATTCAATCGTCTTGTCTTGTTCCTAGATGTTCTATAGGAAAAAATTGTCATTTCGTTCCTCTACGGAGAACCTTTTATTCTAAATTCTAAATCACAGGATCTAAATTCTAAATCACAGGATAAGAAAAAAATTAACTATCTTTCTTTGATTACAGGATCAAGATTCATTTANNNNTTATCTATACTATATAATATATTCNNNNNNTATAGATATTTCTATTCTATATTTAGATTTATAGCTATCAGATCGTGGCTTGATGTACCAAAAATTTCCATTTCGTTGCATCCAATATTTTTGTTCCGACCATCGTATGAAGAAAGCATGCAAGATAAATACTATCATTTCCAATCTCCTATTTTAATTTTAATATTGTANNNNNNTTGAAGTGAAGTAAAAATTTGGAAACCCTCTCTTTTCTAACAGGGAAAAATAAATCAAAAAATATTAGTAATTTAGTCTACATAAAAATTAGAATATAAAGAGAGTTCTTTTTCTTAATCTCATGAACAAGATCTAAGAATCCATTTAGTTGATGAAAGAACATGGGGGGGGGCAGGCCTGAGGATCAACCGGTAGTGGGTNNNNGGAAGGGGATTGCTTTTTCCTTGACCAATTCTTTCAAAAAACGAATCGGATTCATGGGTCATAATAAGATAATTCATGATTCAGATGCTTAATAATAAGATAATAAGAAGGAATAATCAAATTGAATTCATGAATTTACCCGGCAATTTATGGTCCAATAAAGGATTTTTATCTTCGAAACCCATTGGAAGGGGTAGTGCACGAGAAAAAAAATCATGCGGAAATGATCGACTCCTTGGATGCCCCAAAATACTATGAGTGTCGGAA

233_20190915_01_03_trnT(GUU)-comp-psbD-comp CATAGAGATGAAATTGGAACAAGTTGACCCCCTTTTCTATTTATTTTCATTTCTTTGGACTCCGCAAGAATTTGCCGATATTTCCAATTCAATCGTCTTGTCTTGTTCCTAGATGTTCTATAGGAAAAAATTGTCATTTCGTTCCTCTACGGAGAACCTTTTATNNNNNNNNNNNNNNNNNNNNNTCTAAATTCTAAATCACAGGATAAGAAAAAAATTCACTATCTTTCTTTGATTACAGGATCAAGATTCATTTATATCTTATCTATACTATATAATATATTCNNNNNNTATAGATATTTCTATTCTATATTTAGATTTATAGCTATCAGATCGTGGCTTGATGTACCAAAAATTTCCATTTCGTTGCATCCAATATTTTTGTTCCGACCATCGTATGAAGAAAGCATGCAAGATAAATACTATCATTTCCAATCTCCTATTTTAATTTTAATATTGTANNNNNNTTGAAGTGAAGTAAAAATTTGGAAACCCTCTCTTTTCTAACAGGGAAAAATAAATCAAAAAATATTAGTAATTTAGTCTACATAAAAATTAGAATATAAAGAGAGTTCTTTTTCTTAATCTCATGAACAAGATCTAAGAATCCATTTAGTTGATGAAAGAACATGGGGGGGGGCAGGCCTGAGGATCAACCGGTAGTGGGTNNNNGGAAGGGGATTGCTTTTTCCTTGACCAATTCTTTCAAAAAACGAATCGGATTCATGGGTCATAATAAGATAATTCATGATTCAGATGCTTAATAATAAGATAATAAGAAGGAATAATCAAATTGAATTCATGAATTTACCCGGCAATTTATGGTCCAATAAAGGATTTTTATCTTCGAAACCCATTGGAAGGGGTAGTGCACGAGAAAAAAAATCATGCGGAAATGATCGACTCCTTGGATGCCCCAAAATACTATGAGTGTCGGAA

234_20190915_01_04_trnT(GUU)-comp-psbD-comp CATAGAGATGAAATTGGAACAAGTTGACCCCCTTTTCTATTTATTTTCATTTCTTTGGACTCCGCAAGAATTTGCCGATATTTCCAATTCAATCGTCTTGTCTTGTTCCTAGATGTTCTATAGGAAAAAATTGTCATTTCGTTCCTCTACGGAGAACCTTTTATNNNNNNNNNNNNNNNNNNNNNTCTAAATTCTAAATCACAGGATAAGAAAAAAATTCACTATCTTTCTTTGATTACAGGATCAAGATTCATTTATATCTTATCTATACTATATAATATATTCTATAGATATAGATATTTCTATTCTATATTTAGATTTATAGCTATCAGATCGTGGCTTGATGTACCAAAAATTTCCATTTCGTTGCATCCAATATTTTTGTTCCGACCATCGTATGAAGAAAGGATGCAAGATAAATACTATCATTTCCAATCTCCTATTTTAATTTTAATATTGTATTGAAGTTGAAGTGAAGTAAAAAATTGGAAACCCTCTCTTTTCTAACAGGGAAAAATAAATCAAAAAATATTAGTAATTTAGTCTACATAAAAATTAGAATATAAAGAGAGTTCTTTTTCTTAATCTCATGAACAAGATCTAAGAATCCATTTAGTTGATGGAAGAACATGGGGGGGGGCAGGCCTGAGGATCAACCGGTAGTGGGTNNNNGGAAGGGGATTGCTTTTTCCTTGACCAATTCTTTCAAAAAACGAATCGGATTCATGGGTCATAATAAGATAATTCATGATTCAGATGCTTAATAATAAGATAATAAGAAGGAATAATCAAATTGAATTCATGAATTTACCCGGCAATTTATGGTCCAATAAAGGATTTTTATCTTCGAAACCCATTGGAAGGGGTAGTGCACGAGAAAAAAAATCATGCGGAAATGATCGACTCCTTGGATGCCCCAAAATACTATGAGTGTCGGAA

235_20190915_01_05_trnT(GUU)-comp-psbD-comp CATAGAGATGAAATTGGAACAAGTTGACCCCCTTTTCTATTTATTTTCATTTCTTTGGACTCCGCAAGAATTTGCCGATATTTCCAATTCAATCGTCTTGTCTTGTTCCTAGATGTTCTATAGGAAAAAATTGTCATTTCGTTCCTCTACGGAGAACCTTTTATNNNNNNNNNNNNNNNNNNNNNTCTAAATTCTAAATCACAGGATAAGAAAAAAATTCACTATCTTTCTTTGATTACAGGATCAAGATTCATTTATATCTTATCTATACTATATAATATATTCNNNNNNTATAGATATTTCTATTCTATATTTAGATTTATAGCTATCAGATCGTGGCTTGATGTACCAAAAATTTCCATTTCGTTGCATCCAATATTTTTGTTCCGACCATCGTATGAAGAAAGCATGCAAGATAAATACTATCATTTCCAATCTCCTATTTTAATTTTAATATTGTANNNNNNTTGAAGTGAAGTAAAAATTTGGAAACCCTCTCTTTTCTAACAGGGAAAAATAAATCAAAAAATATTAGTAATTTAGTCTACATAAAAATTAGAATATAAAGAGAGTTCTTTTTCTTAATCTCATGAACAAGATCTAAGAATCCATTTAGTTGATGAAAGAACATGGGGGGGGGCAGGCCTGAGGATCAACCGGTAGTGGGTNNNNGGAAGGGGATTGCTTTTTCCTTGACCAATTCTTTCAAAAAACGAATCGGATTCATGGGTCATAATAAGATAATTCATGATTCAGATGCTTAATAATAAGATAATAAGAAGGAATAATCAAATTGAATTCATGAATTTACCCGGCAATTTATGGTCCAATAAAGGATTTTTATCTTCGAAACCCATTGGAAGGGGTAGTGCACGAGAAAAAAAATCATGCGGAAATGATCGACTCCTTGGATGCCCCAAAATACTATGAGTGTCGGAA

236_20180901_01_01_trnT(GUU)-comp-psbD-comp CATAGAGATGAAATTGGAACAAGTTGACCCCCTTTTCTATTTATTTTCATTTCTTTGGACTCCGCAAGAATTTGCCGATATTTCCAATTCAATCGTCTTGTCTTGTTCCTAGATGTTCTATAGGAAAAAATTGTCATTTCGTTCCTCTACGGAGAACCTTTTATNNNNNNNNNNNNNNNNNNNNNTCTAAATTCTAAATCACAGGATAAGAAAAAAATTCACTATCTTTCTTTGATTACAGGATCAAGATTCATTTATATCTTATCTATACTATATAATATATTCNNNNNNTATAGATATTTCTATTCTATATTTAGATTTATAGCTATCAGATCGTGGCTTGATGTACCAAAAATTTCCATTTCGTTGCATCCAATATTTTTGTTCCGACCATCGTATGAAGAAAGCATGCAAGATAAATACTATCATTTCCAATCTCCTATTTTAATTTTAATATTGTANNNNNNTTGAAGTGAAGTAAAAATTTGGAAACCCTCTCTTTTCTAACAGGGAAAAATAAATCAAAAAATATTAGTAATTTAGTCTACATAAAAATTAGAATATAAAGAGAGTTCTTTTTCTTAATCTCATGAACAAGATCTAAGAATCCATTTAGTTGATGAAAGAACATGGGGGGGGGCAGGCCTGAGGATCAACCGGTAGTGGGTNNNNGGAAGGGGATTGCTTTTTCCTTGACCAATTCTTTCAAAAAACGAATCGGATTCATGGGTCATAATAAGATAATTCATGATTCAGATGCTTAATAATAAGATAATAAGAAGGAATAATCAAATTGAATTCATGAATTTACCCGGCAATTTATGGTCCAATAAAGGATTTTTATCTTCGAAACCCATTGGAAGGGGTAGTGCACGAGAAAAAAAATCATGCGGAAATGATCGACTCCTTGGATGCCCCAAAATACTATGAGTGTCGGAA

237_20190626_04_01_trnT(GUU)-comp-psbD-comp CATAGAGATGAAATTGGAACAAGTTGACCCCCTTTTCTATTTATTTTCATTTCTTTGGACTCCGCAAGAATTTGCCGATATTTCCAATTCAATCGTCTTGTCTTGTTCCTAGATGTTCTATAGGAAAAAATTGTCATTTCGTTCCTCTACGGAGAACCTTTTATNNNNNNNNNNNNNNNNNNNNNTCTAAATTCTAAATCACAGGATAAGAAAAAAATTCACTATCTTTCTTTGATTACAGGATCAAGATTCATTTATATCTTATCTATACTATATAATATATTCTATAGATATAGATATTTCTATTCTATATTTAGATTTATAGCTATCAGATCGTGGCTTGATGTACCAAAAATTTCCATTTCGTTGCATCCAATATTTTTGTTCCGACCATCGTATGAAGAAAGGATGCAAGATAAATACTATCATTTCCAATCTCCTATTTTAATTTGAATATGGTATTGAAGTTGAAGTGAAGTAAAAAATTGGAAACCCTCTCTTTTCTAACAGGGAAAAATAAATCAAAAAATATTAGTAATTTAGTCTACATAAAAATTAGAATATAAAGAGAGTTCTTTTTCTTAATCTCATGAACAAGATCTAAGAATCCATTTAGTTGATGGAAGAACATGGGGGGGGGCAGGCCTGAGGATCAACCGGTAGTGGGTNNNNGGAAGGGGATTGCTTTTTCCTTGACCAATTCTTTCAAAAAACGAATCGGATTCATGGGTCATAATAAGATAATTCATGATTCAGATGCTTAATAATAAGATAATAAGAAGGAATAATCAAATTGAATTCATGAATTTACCCGGCAATTTATGGTCCAATAAAGGATTTTTATCTTCGAAACCCATTGGAAGGGGTAGTGCACGAGAAAAAAAATCATGCGGAAATGATCGACTCCTTGGATGCCCCAAAATACTATGAGTGTCGGAA

238_20160909_01_01_trnT(GUU)-comp-psbD-comp CATAGAGATGAAATTGGAACAAGTTGACCCCCTTTTCTATTTATTTTCATTTCTTTGGACTCCGCAAGAATTTGCCGATATTTCCAATTCAATCGTCTTGTCTTGTTCCTAGATGTTCTATAGGAAAAAATTGTCATTTCGTTCCTCTACGGAGAACCTTTTATNNNNNNNNNNNNNNNNNNNNNTCTAAATTCTAAATCACAGGATAAGAAAAAAATTCACTATCTTTCTTTGATTACAGGATCAAGATTCATTTATATCTTATCTATACTATATAATATATTCTATAGATATAGATATTTCTATTCTATATTTAGATTTATAGCTATCAGATCGTGGCTTGATGTACCAAAAATTTCCATTTCGTTGCATCCAATATTTTTGTTCCGACCATCGTATGAAGAAAGGATGCAAGATAAATACTATCATTTCCAATCTCCTATTTTAATTTTAATATTGTATTGAAGTTGAAGTGAAGTAAAAAATTGGAAACCCTCTCTTTTCTAACAGGGAAAAATAAATCAAAAAATATTAGTAATTTAGTCTACATAAAAATTAGAATATAAAGAGAGTTCTTTTTCTTAATCTCATGAACAAGATCTAAGAATCCATTTAGTTGATGGAAGAACATGGGGGGGGGCAGGCCTGAGGATCAACCGGTAGTGGGTNNNNGGAAGGGGATTGCTTTTTCCTTGACCAATTCTTTCAAAAAACGAATCGGATTCATGGGTCATAATAAGATAATTCATGATTCAGATGCTTAATAATAAGATAATAAGAAGGAATAATCAAATTGAATTCATGAATTTACCCGGCAATTTATGGTCCAATAAAGGATTTTTATCTTCGAAACCCATTGGAAGGGGTAGTGCACGAGAAAAAAAATCATGCGGAAATGATCGACTCCTTGGATGCCCCAAAATACTATGAGTGTCGGAA

239_20180616_02_01_trnT(GUU)-comp-psbD-comp CATAGAGATGAAATTGGAACAAGTTGACCCCCTTTTCTATTTATTTTCATTTCTTTGGACTCCGCAAGAATTTGCCGATATTTCCAATTCAATCGTCTTGTCTTGTTCCTAGATGTTCTATAGGAAAAAATTGTCATTTCGTTCCTCTACGGAGAACCTTTTATNNNNNNNNNNNNNNNNNNNNNTCTAAATTCTAAATCACAGGATAAGAAAAAAATTCACTATCTTTCTTTGATTACAGGATCAAGATTCATTTATATCTTATCTATACTATATAATATATTCTATAGATATAGATATTTCTATTCTATATTTAGATTTATAGCTATCAGATCGTGGCTTGATGTACCAAAAATTTCCATTTCGTTGCATCCAATATTTTTGTTCCGACCATCGTATGAAGAAAGGATGCAAGATAAATACTATCATTTCCAATCTCCTATTTTAATTTTAATATTGTATTGAAGTTGAAGTGAAGTAAAAAATTGGAAACCCTCTCTTTTCTAACAGGGAAAAAAAAATCAAAAAATATTAGTAATTTAGTCTACATAAAAATTAGAATATAAAGAGAGTTCTTTTTCTTAATCTCATGAACAAGATCTAAGAATCCATTTAGTTGATGGAAGAACATGGGGGGGGNNNNNNNNNNNNNNNNNNNNNNNNNNNNNNNNNGGAAGGGGATTGCTTTTTCTTTGACCATTTCTTTCAAAAAACGAATCGGATTCATGGGTCATAATAAGATAATTCATGATTCAGATGCTTAATAATAAGATAATAAGAAGGAATAATCAAATTGAATTCATGAATTTACCCGGCAATTTATGGTCCAATAAAGGATTTTTATCTTCGAAACCCATTGGAAGGGGTAGTGCACGAGAAAAAAAATCATGCGGAAATGATCGACTCCTTGGATGCCCCAAAATACTATGAGTGTCGGAA

240_20170916_02_01_trnT(GUU)-comp-psbD-comp CATAGAGATGAAATTGGAACAAGTTGACCCCCTTTTCTATTTATTTTCATTTCTTTGGACTCCGCAAGAATTTGCCGATATTTCCAATTCAATCGTCTTGTCTTGTTCCTAGATGTTCTATAGGAAAAAATTGTCATTTCGTTCCTCTACGGAGAACCTTTTATNNNNNNNNNNNNNNNNNNNNNTCTAAATTCTAAATCACAGGATAAGAAAAAAATTCACTATCTTTCTTTGATTACAGGATCAAGATTCATTTATATCTTATCTATACTATATAATATATTCNNNNNNTATAGATATTTCTATTCTATATTTAGATTTATAGCTATCAGATCGTGGCTTGATGTACCAAAAATTTCCATTTCGTTGCATCCAATATTTTTGTTCCGACCATCGTATGAAGAAAGCATGCAAGATAAATACTATCATTTCCAATCTCCTATTTTAATTTTAATATTGTANNNNNNTTGAAGTGAAGTAAAAATTTGGAAACCCTCTCTTTTCTAACAGGGAAAAATAAATCAAAAAATATTAGTAATTTAGTCTACATAAAAATTAGAATATAAAGAGAGTTCTTTTTCTTAATCTCATGAACAAGATCTAAGAATCCATTTAGTTGATGAAAGAACATGGGGGGGGGCAGGCCTGAGGATCAACCGGTAGTGGGTNNNNGGAAGGGGATTGCTTTTTCCTTGACCAATTCTTTCAAAAAACGAATCGGATTCATGGGTCATAATAAGATAATTCATGATTCAGATGCTTAATAATAAGATAATAAGAAGGAATAATCAAATTGAATTCATGAATTTACCCGGCAATTTATGGTCCAATAAAGGATTTTTATCTTCGAAACCCATTGGAAGGGGTAGTGCACGAGAAAAAAAATCATGCGGAAATGATCGACTCCTTGGATGCCCCAAAATACTATGAGTGTCGGAA

241_20180808_01_01_trnT(GUU)-comp-psbD-comp CATAGAGATGAAATTGGAACAAGTTGACCCCCTTTTCTATTTATTTTCATTTCTTTGGACTCCGCAAGAATTTGCCGATATTTCCAATTCAATCGTCTTGTCTTGTTCCTAGATGTTCTATAGGAAAAAATTGTCATTTCGTTCCTCTACGGAGAACCTTTTATNNNNNNNNNNNNNNNNNNNNNTCTAAATTCTAAATCACAGGATAAGAAAAAAATTCACTATCTTTCTTTGATTACAGGATCAAGATTCATTTATATCTTATCTATACTATATAATATATTCNNNNNNTATAGATATTTCTATTCTATATTTAGATTTATAGCTATCAGATCGTGGCTTGATGTACCAAAAATTTCCATTTCGTTGCATCCAATATTTTTGTTCCGACCATCGTATGAAGAAAGCATGCAAGATAAATACTATCATTTCCAATCTCCTATTTTAATTTTAATATTGTANNNNNNTTGAAGTGAAGTAAAAATTTGGAAACCCTCTCTTTTCTAACAGGGAAAAATAAATCAAAAAATATTAGTAATTTAGTCTACATAAAAATTAGAATATAAAGAGAGTTCTTTTTCTTAATCTCATGAACAAGATCTAAGAATCCATTTAGTTGATGAAAGAACATGGGGGGGGGCAGGCCTGAGGATCAACCGGTAGTGGGTNNNNGGAAGGGGATTGCTTTTTCCTTGACCAATTCTTTCAAAAAACGAATCGGATTCATGGGTCATAATAAGATAATTCATGATTCAGATGCTTAATAATAAGATAATAAGAAGGAATAATCAAATTGAATTCATGAATTTACCCGGCAATTTATGGTCCAATAAAGGATTTTTATCTTCGAAACCCATTGGAAGGGGTAGTGCACGAGAAAAAAAATCATGCGGAAATGATCGACTCCTTGGATGCCCCAAAATACTATGAGTGTCGGAA

242_20170917_01_01_trnT(GUU)-comp-psbD-comp CATAGAGATGAAATTGGAACAAGTTGACCCCCTTTTCTATTTATTTTCATTTCTTTGGACTCCGCAAGAATTTGCCGATATTTCCAATTCAATCGTCTTGTCTTGTTCCTAGATGTTCTATAGGAAAAAATTGTCATTTCGTTCCTCTACGGAGAACCTTTTATNNNNNNNNNNNNNNNNNNNNNTCTAAATTCTAAATCACAGGATAAGAAAAAAATTCACTATCTTTCTTTGATTACAGGATCAAGATTCATTTATATCTTATCTATACTATATAATATATTCNNNNNNTATAGATATTTCTATTCTATATTTAGATTTATAGCTATCAGATCGTGGCTTGATGTACCAAAAATTTCCATTTCGTTGCATCCAATATTTTTGTTCCGACCATCGTATGAAGAAAGCATGCAAGATAAATACTATCATTTCCAATCTCCTATTTTAATTTTAATATTGTANNNNNNTTGAAGTGAAGTAAAAATTTGGAAACCCTCTCTTTTCTAACAGGGAAAAATAAATCAAAAAATATTAGTAATTTAGTCTACATAAAAATTAGAATATAAAGAGAGTTCTTTTTCTTAATCTCATGAACAAGATCTAAGAATCCATTTAGTTGATGAAAGAACATGGGGGGGGGCAGGCCTGAGGATCAACCGGTAGTGGGTNNNNGGAAGGGGATTGCTTTTTCCTTGACCAATTCTTTCAAAAAACGAATCGGATTCATGGGTCATAATAAGATAATTCATGATTCAGATGCTTAATAATAAGATAATAAGAAGGAATAATCAAATTGAATTCATGAATTTACCCGGCAATTTATGGTCCAATAAAGGATTTTTATCTTCGAAACCCATTGGAAGGGGTAGTGCACGAGAAAAAAAATCATGCGGAAATGATCGACTCCTTGGATGCCCCAAAATACTATGAGTGTCGGAA

243_20180807_03_06_trnT(GUU)-comp-psbD-comp CATAGAGATGAAATTGGAACAAGTTGACCCCCTTTTCTATTTATTTTCATTTCTTTGGACTCCGCAAGAATTTGCCGATATTTCCAATTCAATCGTCTTGTCTTGTTCCTAGATGTTCTATAGGAAAAAATTGTCATTTCGTTCCTCTACGGAGAACCTTTTATNNNNNNNNNNNNNNNNNNNNNTCTAAATTCTAAATCACAGGATAAGAAAAAAATTCACTATCTTTCTTTGATTACAGGATCAAGATTCATTTATATCTTATCTATACTATATAATATATTCNNNNNNTATAGATATTTCTATTCTATATTTAGATTTATAGCTATCAGATCGTGGCTTGATGTACCAAAAATTTCCATTTCGTTGCATCCAATATTTTTGTTCCGACCATCGTATGAAGAAAGCATGCAAGATAAATACTATCATTTCCAATCTCCTATTTTAATTTTAATATTGTANNNNNNTTGAAGTGAAGTAAAAATTTGGAAACCCTCTCTTTTCTAACAGGGAAAAATAAATCAAAAAATATTAGTAATTTAGTCTACATAAAAATTAGAATATAAAGAGAGTTCTTTTTCTTAATCTCATGAACAAGATCTAAGAATCCATTTAGTTGATGAAAGAACATGGGGGGGGGCAGGCCTGAGGATCAACCGGTAGTGGGTNNNNGGAAGGGGATTGCTTTTTCCTTGACCAATTCTTTCAAAAAACGAATCGGATTCATGGGTCATAATAAGATAATTCATGATTCAGATGCTTAATAATAAGATAATAAGAAGGAATAATCAAATTGAATTCATGAATTTACCCGGCAATTTATGGTCCAATAAAGGATTTTTATCTTCGAAACCCATTGGAAGGGGTAGTGCACGAGAAAAAAAATCATGCGGAAATGATCGACTCCTTGGATGCCCCAAAATACTATGAGTGTCGGAA

244_20180807_03_07_trnT(GUU)-comp-psbD-comp CATAGAGATGAAATTGGAACAAGTTGACCCCCTTTTCTATTTATTTTCATTTCTTTGGACTCCGCAAGAATTTGCCGATATTTCCAATTCAATCGTCTTGTCTTGTTCCTAGATGTTCTATAGGAAAAAATTGTCATTTCGTTCCTCTACGGAGAACCTTTTATNNNNNNNNNNNNNNNNNNNNNTCTAAATTCTAAATCACAGGATAAGAAAAAAATTCACTATCTTTCTTTGATTACAGGATCAAGATTCATTTATATCTTATCTATACTATATAATATATTCNNNNNNTATAGATATTTCTATTCTATATTTAGATTTATAGCTATCAGATCGTGGCTTGATGTACCAAAAATTTCCATTTCGTTGCATCCAATATTTTTGTTCCGACCATCGTATGAAGAAAGCATGCAAGATAAATACTATCATTTCCAATCTCCTATTTTAATTTTAATATTGTANNNNNNTTGAAGTGAAGTAAAAATTTGGAAACCCTCTCTTTTCTAACAGGGAAAAATAAATCAAAAAATATTAGTAATTTAGTCTACATAAAAATTAGAATATAAAGAGAGTTCTTTTTCTTAATCTCATGAACAAGATCTAAGAATCCATTTAGTTGATGAAAGAACATGGGGGGGGGCAGGCCTGAGGATCAACCGGTAGTGGGTNNNNGGAAGGGGATTGCTTTTTCCTTGACCAATTCTTTCAAAAAACGAATCGGATTCATGGGTCATAATAAGATAATTCATGATTCAGATGCTTAATAATAAGATAATAAGAAGGAATAATCAAATTGAATTCATGAATTTACCCGGCAATTTATGGTCCAATAAAGGATTTTTATCTTCGAAACCCATTGGAAGGGGTAGTGCACGAGAAAAAAAATCATGCGGAAATGATCGACTCCTTGGATGCCCCAAAATACTATGAGTGTCGGAA

245_20180807_03_08_trnT(GUU)-comp-psbD-comp CATAGAGATGAAATTGGAACAAGTTGACCCCCTTTTCTATTTATTTTCATTTCTTTGGACTCCGCAAGAATTTGCCGATATTTCCAATTCAATCGTCTTGTCTTGTTCCTAGATGTTCTATAGGAAAAAATTGTCATTTCGTTCCTCTACGGAGAACCTTTTATNNNNNNNNNNNNNNNNNNNNNTCTAAATTCTAAATCACAGGATAAGAAAAAAATTCACTATCTTTCTTTGATTACAGGATCAAGATTCATTTATATCTTATCTATACTATATAATATATTCNNNNNNTATAGATATTTCTATTCTATATTTAGATTTATAGCTATCAGATCGTGGCTTGATGTACCAAAAATTTCCATTTCGTTGCATCCAATATTTTTGTTCCGACCATCGTATGAAGAAAGCATGCAAGATAAATACTATCATTTCCAATCTCCTATTTTAATTTTAATATTGTANNNNNNTTGAAGTGAAGTAAAAATTTGGAAACCCTCTCTTTTCTAACAGGGAAAAATAAATCAAAAAATATTAGTAATTTAGTCTACATAAAAATTAGAATATAAAGAGAGTTCTTTTTCTTAATCTCATGAACAAGATCTAAGAATCCATTTAGTTGATGAAAGAACATGGGGGGGGGCAGGCCTGAGGATCAACCGGTAGTGGGTNNNNGGAAGGGGATTGCTTTTTCCTTGACCAATTCTTTCAAAAAACGAATCGGATTCATGGGTCATAATAAGATAATTCATGATTCAGATGCTTAATAATAAGATAATAAGAAGGAATAATCAAATTGAATTCATGAATTTACCCGGCAATTTATGGTCCAATAAAGGATTTTTATCTTCGAAACCCATTGGAAGGGGTAGTGCACGAGAAAAAAAATCATGCGGAAATGATCGACTCCTTGGATGCCCCAAAATACTATGAGTGTCGGAA

246_20180807_03_09_trnT(GUU)-comp-psbD-comp CATAGAGATGAAATTGGAACAAGTTGACCCCCTTTTCTATTTATTTTCATTTCTTTGGACTCCGCAAGAATTTGCCGATATTTCCAATTCAATCGTCTTGTCTTGTTCCTAGATGTTCTATAGGAAAAAATTGTCATTTCGTTCCTCTACGGAGAACCTTTTATNNNNNNNNNNNNNNNNNNNNNTCTAAATTCTAAATCACAGGATAAGAAAAAAATTCACTATCTTTCTTTGATTACAGGATCAAGATTCATTTATATCTTATCTATACTATATAATATATTCNNNNNNTATAGATATTTCTATTCTATATTTAGATTTATAGCTATCAGATCGTGGCTTGATGTACCAAAAATTTCCATTTCGTTGCATCCAATATTTTTGTTCCGACCATCGTATGAAGAAAGCATGCAAGATAAATACTATCATTTCCAATCTCCTATTTTAATTTTAATATTGTANNNNNNTTGAAGTGAAGTAAAAATTTGGAAACCCTCTCTTTTCTAACAGGGAAAAATAAATCAAAAAATATTAGTAATTTAGTCTACATAAAAATTAGAATATAAAGAGAGTTCTTTTTCTTAATCTCATGAACAAGATCTAAGAATCCATTTAGTTGATGAAAGAACATGGGGGGGGGCAGGCCTGAGGATCAACCGGTAGTGGGTNNNNGGAAGGGGATTGCTTTTTCCTTGACCAATTCTTTCAAAAAACGAATCGGATTCATGGGTCATAATAAGATAATTCATGATTCAGATGCTTAATAATAAGATAATAAGAAGGAATAATCAAATTGAATTCATGAATTTACCCGGCAATTTATGGTCCAATAAAGGATTTTTATCTTCGAAACCCATTGGAAGGGGTAGTGCACGAGAAAAAAAATCATGCGGAAATGATCGACTCCTTGGATGCCCCAAAATACTATGAGTGTCGGAA

247_20180807_03_10_trnT(GUU)-comp-psbD-comp CATAGAGATGAAATTGGAACAAGTTGACCCCCTTTTCTATTTATTTTCATTTCTTTGGACTCCGCAAGAATTTGCCGATATTTCCAATTCAATCGTCTTGTCTTGTTCCTAGATGTTCTATAGGAAAAAATTGTCATTTCGTTCCTCTACGGAGAACCTTTTATNNNNNNNNNNNNNNNNNNNNNTCTAAATTCTAAATCACAGGATAAGAAAAAAATTCACTATCTTTCTTTGATTACAGGATCAAGATTCATTTATATCTTATCTATACTATATAATATATTCNNNNNNTATAGATATTTCTATTCTATATTTAGATTTATAGCTATCAGATCGTGGCTTGATGTACCAAAAATTTCCATTTCGTTGCATCCAATATTTTTGTTCCGACCATCGTATGAAGAAAGCATGCAAGATAAATACTATCATTTCCAATCTCCTATTTTAATTTTAATATTGTANNNNNNTTGAAGTGAAGTAAAAATTTGGAAACCCTCTCTTTTCTAACAGGGAAAAATAAATCAAAAAATATTAGTAATTTAGTCTACATAAAAATTAGAATATAAAGAGAGTTCTTTTTCTTAATCTCATGAACAAGATCTAAGAATCCATTTAGTTGATGAAAGAACATGGGGGGGGGCAGGCCTGAGGATCAACCGGTAGTGGGTNNNNGGAAGGGGATTGCTTTTTCCTTGACCAATTCTTTCAAAAAACGAATCGGATTCATGGGTCATAATAAGATAATTCATGATTCAGATGCTTAATAATAAGATAATAAGAAGGAATAATCAAATTGAATTCATGAATTTACCCGGCAATTTATGGTCCAATAAAGGATTTTTATCTTCGAAACCCATTGGAAGGGGTAGTGCACGAGAAAAAAAATCATGCGGAAATGATCGACTCCTTGGATGCCCCAAAATACTATGAGTGTCGGAA

248_20180807_03_11_trnT(GUU)-comp-psbD-comp CATAGAGATGAAATTGGAACAAGTTGACCCCCTTTTCTATTTATTTTCATTTCTTTGGACTCCGCAAGAATTTGCCGATATTTCCAATTCAATCGTCTTGTCTTGTTCCTAGATGTTCTATAGGAAAAAATTGTCATTTCGTTCCTCTACGGAGAACCTTTTATNNNNNNNNNNNNNNNNNNNNNTCTAAATTCTAAATCACAGGATAAGAAAAAAATTCACTATCTTTCTTTGATTACAGGATCAAGATTCATTTATATCTTATCTATACTATATAATATATTCNNNNNNTATAGATATTTCTATTCTATATTTAGATTTATAGCTATCAGATCGTGGCTTGATGTACCAAAAATTTCCATTTCGTTGCATCCAATATTTTTGTTCCGACCATCGTATGAAGAAAGCATGCAAGATAAATACTATCATTTCCAATCTCCTATTTTAATTTTAATATTGTANNNNNNTTGAAGTGAAGTAAAAATTTGGAAACCCTCTCTTTTCTAACAGGGAAAAATAAATCAAAAAATATTAGTAATTTAGTCTACATAAAAATTAGAATATAAAGAGAGTTCTTTTTCTTAATCTCATGAACAAGATCTAAGAATCCATTTAGTTGATGAAAGAACATGGGGGGGGGCAGGCCTGAGGATCAACCGGTAGTGGGTNNNNGGAAGGGGATTGCTTTTTCCTTGACCAATTCTTTCAAAAAACGAATCGGATTCATGGGTCATAATAAGATAATTCATGATTCAGATGCTTAATAATAAGATAATAAGAAGGAATAATCAAATTGAATTCATGAATTTACCCGGCAATTTATGGTCCAATAAAGGATTTTTATCTTCGAAACCCATTGGAAGGGGTAGTGCACGAGAAAAAAAATCATGCGGAAATGATCGACTCCTTGGATGCCCCAAAATACTATGAGTGTCGGAA

249_20180807_03_12_trnT(GUU)-comp-psbD-comp CATAGAGATGAAATTGGAACAAGTTGACCCCCTTTTCTATTTATTTTCATTTCTTTGGACTCCGCAAGAATTTGCCGATATTTCCAATTCAATCGTCTTGTCTTGTTCCTAGATGTTCTATAGGAAAAAATTGTCATTTCGTTCCTCTACGGAGAACCTTTTATNNNNNNNNNNNNNNNNNNNNNTCTAAATTCTAAATCACAGGATAAGAAAAAAATTCACTATCTTTCTTTGATTACAGGATCAAGATTCATTTATATCTTATCTATACTATATAATATATTCNNNNNNTATAGATATTTCTATTCTATATTTAGATTTATAGCTATCAGATCGTGGCTTGATGTACCAAAAATTTCCATTTCGTTGCATCCAATATTTTTGTTCCGACCATCGTATGAAGAAAGCATGCAAGATAAATACTATCATTTCCAATCTCCTATTTTAATTTTAATATTGTANNNNNNTTGAAGTGAAGTAAAAATTTGGAAACCCTCTCTTTTCTAACAGGGAAAAATAAATCAAAAAATATTAGTAATTTAGTCTACATAAAAATTAGAATATAAAGAGAGTTCTTTTTCTTAATCTCATGAACAAGATCTAAGAATCCATTTAGTTGATGAAAGAACATGGGGGGGGGCAGGCCTGAGGATCAACCGGTAGTGGGTNNNNGGAAGGGGATTGCTTTTTCCTTGACCAATTCTTTCAAAAAACGAATCGGATTCATGGGTCATAATAAGATAATTCATGATTCAGATGCTTAATAATAAGATAATAAGAAGGAATAATCAAATTGAATTCATGAATTTACCCGGCAATTTATGGTCCAATAAAGGATTTTTATCTTCGAAACCCATTGGAAGGGGTAGTGCACGAGAAAAAAAATCATGCGGAAATGATCGACTCCTTGGATGCCCCAAAATACTATGAGTGTCGGAA

250_20180807_03_13_trnT(GUU)-comp-psbD-comp CATAGAGATGAAATTGGAACAAGTTGACCCCCTTTTCTATTTATTTTCATTTCTTTGGACTCCGCAAGAATTTGCCGATATTTCCAATTCAATCGTCTTGTCTTGTTCCTAGATGTTCTATAGGAAAAAATTGTCATTTCGTTCCTCTACGGAGAACCTTTTATNNNNNNNNNNNNNNNNNNNNNTCTAAATTCTAAATCACAGGATAAGAAAAAAATTCACTATCTTTCTTTGATTACAGGATCAAGATTCATTTATATCTTATCTATACTATATAATATATTCNNNNNNTATAGATATTTCTATTCTATATTTAGATTTATAGCTATCAGATCGTGGCTTGATGTACCAAAAATTTCCATTTCGTTGCATCCAATATTTTTGTTCCGACCATCGTATGAAGAAAGCATGCAAGATAAATACTATCATTTCCAATCTCCTATTTTAATTTTAATATTGTANNNNNNTTGAAGTGAAGTAAAAATTTGGAAACCCTCTCTTTTCTAACAGGGAAAAATAAATCAAAAAATATTAGTAATTTAGTCTACATAAAAATTAGAATATAAAGAGAGTTCTTTTTCTTAATCTCATGAACAAGATCTAAGAATCCATTTAGTTGATGAAAGAACATGGGGGGGGGCAGGCCTGAGGATCAACCGGTAGTGGGTNNNNGGAAGGGGATTGCTTTTTCCTTGACCAATTCTTTCAAAAAACGAATCGGATTCATGGGTCATAATAAGATAATTCATGATTCAGATGCTTAATAATAAGATAATAAGAAGGAATAATCAAATTGAATTCATGAATTTACCCGGCAATTTATGGTCCAATAAAGGATTTTTATCTTCGAAACCCATTGGAAGGGGTAGTGCACGAGAAAAAAAATCATGCGGAAATGATCGACTCCTTGGATGCCCCAAAATACTATGAGTGTCGGAA

251_20180807_03_14_trnT(GUU)-comp-psbD-comp CATAGAGATGAAATTGGAACAAGTTGACCCCCTTTTCTATTTATTTTCATTTCTTTGGACTCCGCAAGAATTTGCCGATATTTCCAATTCAATCGTCTTGTCTTGTTCCTAGATGTTCTATAGGAAAAAATTGTCATTTCGTTCCTCTACGGAGAACCTTTTATNNNNNNNNNNNNNNNNNNNNNTCTAAATTCTAAATCACAGGATAAGAAAAAAATTCACTATCTTTCTTTGATTACAGGATCAAGATTCATTTATATCTTATCTATACTATATAATATATTCNNNNNNTATAGATATTTCTATTCTATATTTAGATTTATAGCTATCAGATCGTGGCTTGATGTACCAAAAATTTCCATTTCGTTGCATCCAATATTTTTGTTCCGACCATCGTATGAAGAAAGCATGCAAGATAAATACTATCATTTCCAATCTCCTATTTTAATTTTAATATTGTANNNNNNTTGAAGTGAAGTAAAAATTTGGAAACCCTCTCTTTTCTAACAGGGAAAAATAAATCAAAAAATATTAGTAATTTAGTCTACATAAAAATTAGAATATAAAGAGAGTTCTTTTTCTTAATCTCATGAACAAGATCTAAGAATCCATTTAGTTGATGAAAGAACATGGGGGGGGGCAGGCCTGAGGATCAACCGGTAGTGGGTNNNNGGAAGGGGATTGCTTTTTCCTTGACCAATTCTTTCAAAAAACGAATCGGATTCATGGGTCATAATAAGATAATTCATGATTCAGATGCTTAATAATAAGATAATAAGAAGGAATAATCAAATTGAATTCATGAATTTACCCGGCAATTTATGGTCCAATAAAGGATTTTTATCTTCGAAACCCATTGGAAGGGGTAGTGCACGAGAAAAAAAATCATGCGGAAATGATCGACTCCTTGGATGCCCCAAAATACTATGAGTGTCGGAA

252_20180807_03_15_trnT(GUU)-comp-psbD-comp CATAGAGATGAAATTGGAACAAGTTGACCCCCTTTTCTATTTATTTTCATTTCTTTGGACTCCGCAAGAATTTGCCGATATTTCCAATTCAATCGTCTTGTCTTGTTCCTAGATGTTCTATAGGAAAAAATTGTCATTTCGTTCCTCTACGGAGAACCTTTTATNNNNNNNNNNNNNNNNNNNNNTCTAAATTCTAAATCACAGGATAAGAAAAAAATTCACTATCTTTCTTTGATTACAGGATCAAGATTCATTTATATCTTATCTATACTATATAATATATTCNNNNNNTATAGATATTTCTATTCTATATTTAGATTTATAGCTATCAGATCGTGGCTTGATGTACCAAAAATTTCCATTTCGTTGCATCCAATATTTTTGTTCCGACCATCGTATGAAGAAAGCATGCAAGATAAATACTATCATTTCCAATCTCCTATTTTAATTTTAATATTGTANNNNNNTTGAAGTGAAGTAAAAATTTGGAAACCCTCTCTTTTCTAACAGGGAAAAATAAATCAAAAAATATTAGTAATTTAGTCTACATAAAAATTAGAATATAAAGAGAGTTCTTTTTCTTAATCTCATGAACAAGATCTAAGAATCCATTTAGTTGATGAAAGAACATGGGGGGGGGCAGGCCTGAGGATCAACCGGTAGTGGGTNNNNGGAAGGGGATTGCTTTTTCCTTGACCAATTCTTTCAAAAAACGAATCGGATTCATGGGTCATAATAAGATAATTCATGATTCAGATGCTTAATAATAAGATAATAAGAAGGAATAATCAAATTGAATTCATGAATTTACCCGGCAATTTATGGTCCAATAAAGGATTTTTATCTTCGAAACCCATTGGAAGGGGTAGTGCACGAGAAAAAAAATCATGCGGAAATGATCGACTCCTTGGATGCCCCAAAATACTATGAGTGTCGGAA

253_20180807_03_16_trnT(GUU)-comp-psbD-comp CATAGAGATGAAATTGGAACAAGTTGACCCCCTTTTCTATTTATTTTCATTTCTTTGGACTCCGCAAGAATTTGCCGATATTTCCAATTCAATCGTCTTGTCTTGTTCCTAGATGTTCTATAGGAAAAAATTGTCATTTCGTTCCTCTACGGAGAACCTTTTATNNNNNNNNNNNNNNNNNNNNNTCTAAATTCTAAATCACAGGATAAGAAAAAAATTCACTATCTTTCTTTGATTACAGGATCAAGATTCATTTATATCTTATCTATACTATATAATATATTCNNNNNNTATAGATATTTCTATTCTATATTTAGATTTATAGCTATCAGATCGTGGCTTGATGTACCAAAAATTTCCATTTCGTTGCATCCAATATTTTTGTTCCGACCATCGTATGAAGAAAGCATGCAAGATAAATACTATCATTTCCAATCTCCTATTTTAATTTTAATATTGTANNNNNNTTGAAGTGAAGTAAAAATTTGGAAACCCTCTCTTTTCTAACAGGGAAAAATAAATCAAAAAATATTAGTAATTTAGTCTACATAAAAATTAGAATATAAAGAGAGTTCTTTTTCTTAATCTCATGAACAAGATCTAAGAATCCATTTAGTTGATGAAAGAACATGGGGGGGGGCAGGCCTGAGGATCAACCGGTAGTGGGTNNNNGGAAGGGGATTGCTTTTTCCTTGACCAATTCTTTCAAAAAACGAATCGGATTCATGGGTCATAATAAGATAATTCATGATTCAGATGCTTAATAATAAGATAATAAGAAGGAATAATCAAATTGAATTCATGAATTTACCCGGCAATTTATGGTCCAATAAAGGATTTTTATCTTCGAAACCCATTGGAAGGGGTAGTGCACGAGAAAAAAAATCATGCGGAAATGATCGACTCCTTGGATGCCCCAAAATACTATGAGTGTCGGAA

254_20180807_03_17_trnT(GUU)-comp-psbD-comp CATAGAGATGAAATTGGAACAAGTTGACCCCCTTTTCTATTTATTTTCATTTCTTTGGACTCCGCAAGAATTTGCCGATATTTCCAATTCAATCGTCTTGTCTTGTTCCTAGATGTTCTATAGGAAAAAATTGTCATTTCGTTCCTCTACGGAGAACCTTTTATNNNNNNNNNNNNNNNNNNNNNTCTAAATTCTAAATCACAGGATAAGAAAAAAATTCACTATCTTTCTTTGATTACAGGATCAAGATTCATTTATATCTTATCTATACTATATAATATATTCNNNNNNTATAGATATTTCTATTCTATATTTAGATTTATAGCTATCAGATCGTGGCTTGATGTACCAAAAATTTCCATTTCGTTGCATCCAATATTTTTGTTCCGACCATCGTATGAAGAAAGCATGCAAGATAAATACTATCATTTCCAATCTCCTATTTTAATTTTAATATTGTANNNNNNTTGAAGTGAAGTAAAAATTTGGAAACCCTCTCTTTTCTAACAGGGAAAAATAAATCAAAAAATATTAGTAATTTAGTCTACATAAAAATTAGAATATAAAGAGAGTTCTTTTTCTTAATCTCATGAACAAGATCTAAGAATCCATTTAGTTGATGAAAGAACATGGGGGGGGGCAGGCCTGAGGATCAACCGGTAGTGGGTNNNNGGAAGGGGATTGCTTTTTCCTTGACCAATTCTTTCAAAAAACGAATCGGATTCATGGGTCATAATAAGATAATTCATGATTCAGATGCTTAATAATAAGATAATAAGAAGGAATAATCAAATTGAATTCATGAATTTACCCGGCAATTTATGGTCCAATAAAGGATTTTTATCTTCGAAACCCATTGGAAGGGGTAGTGCACGAGAAAAAAAATCATGCGGAAATGATCGACTCCTTGGATGCCCCAAAATACTATGAGTGTCGGAA

255_20180807_03_18_trnT(GUU)-comp-psbD-comp CATAGAGATGAAATTGGAACAAGTTGACCCCCTTTTCTATTTATTTTCATTTCTTTGGACTCCGCAAGAATTTGCCGATATTTCCAATTCAATCGTCTTGTCTTGTTCCTAGATGTTCTATAGGAAAAAATTGTCATTTCGTTCCTCTACGGAGAACCTTTTATNNNNNNNNNNNNNNNNNNNNNTCTAAATTCTAAATCACAGGATAAGAAAAAAATTCACTATCTTTCTTTGATTACAGGATCAAGATTCATTTATATCTTATCTATACTATATAATATATTCNNNNNNTATAGATATTTCTATTCTATATTTAGATTTATAGCTATCAGATCGTGGCTTGATGTACCAAAAATTTCCATTTCGTTGCATCCAATATTTTTGTTCCGACCATCGTATGAAGAAAGCATGCAAGATAAATACTATCATTTCCAATCTCCTATTTTAATTTTAATATTGTANNNNNNTTGAAGTGAAGTAAAAATTTGGAAACCCTCTCTTTTCTAACAGGGAAAAATAAATCAAAAAATATTAGTAATTTAGTCTACATAAAAATTAGAATATAAAGAGAGTTCTTTTTCTTAATCTCATGAACAAGATCTAAGAATCCATTTAGTTGATGAAAGAACATGGGGGGGGGCAGGCCTGAGGATCAACCGGTAGTGGGTNNNNGGAAGGGGATTGCTTTTTCCTTGACCAATTCTTTCAAAAAACGAATCGGATTCATGGGTCATAATAAGATAATTCATGATTCAGATGCTTAATAATAAGATAATAAGAAGGAATAATCAAATTGAATTCATGAATTTACCCGGCAATTTATGGTCCAATAAAGGATTTTTATCTTCGAAACCCATTGGAAGGGGTAGTGCACGAGAAAAAAAATCATGCGGAAATGATCGACTCCTTGGATGCCCCAAAATACTATGAGTGTCGGAA

256_20180807_03_19_trnT(GUU)-comp-psbD-comp CATAGAGATGAAATTGGAACAAGTTGACCCCCTTTTCTATTTATTTTCATTTCTTTGGACTCCGCAAGAATTTGCCGATATTTCCAATTCAATCGTCTTGTCTTGTTCCTAGATGTTCTATAGGAAAAAATTGTCATTTCGTTCCTCTACGGAGAACCTTTTATNNNNNNNNNNNNNNNNNNNNNTCTAAATTCTAAATCACAGGATAAGAAAAAAATTCACTATCTTTCTTTGATTACAGGATCAAGATTCATTTATATCTTATCTATACTATATAATATATTCNNNNNNTATAGATATTTCTATTCTATATTTAGATTTATAGCTATCAGATCGTGGCTTGATGTACCAAAAATTTCCATTTCGTTGCATCCAATATTTTTGTTCCGACCATCGTATGAAGAAAGCATGCAAGATAAATACTATCATTTCCAATCTCCTATTTTAATTTTAATATTGTANNNNNNTTGAAGTGAAGTAAAAATTTGGAAACCCTCTCTTTTCTAACAGGGAAAAATAAATCAAAAAATATTAGTAATTTAGTCTACATAAAAATTAGAATATAAAGAGAGTTCTTTTTCTTAATCTCATGAACAAGATCTAAGAATCCATTTAGTTGATGAAAGAACATGGGGGGGGGCAGGCCTGAGGATCAACCGGTAGTGGGTNNNNGGAAGGGGATTGCTTTTTCCTTGACCAATTCTTTCAAAAAACGAATCGGATTCATGGGTCATAATAAGATAATTCATGATTCAGATGCTTAATAATAAGATAATAAGAAGGAATAATCAAATTGAATTCATGAATTTACCCGGCAATTTATGGTCCAATAAAGGATTTTTATCTTCGAAACCCATTGGAAGGGGTAGTGCACGAGAAAAAAAATCATGCGGAAATGATCGACTCCTTGGATGCCCCAAAATACTATGAGTGTCGGAA

;

end;

#########################

#ndhCret-trnV-comp alignment

#NEXUS

BEGIN DATA;

dimensions ntax=256 nchar=993;

format missing=N

symbols="ABCDEFGHIKLMNOPQRSTUVWXYZ"

datatype=NUCLEOTIDE gap=- match=.;

matrix

001_20160904_02_01_ndhCret-trnV-comp AATACCAAAATCGGAATAAGACTTGATATTATCAGAAATGCCCAAAAAATATCATATTCGTAAAGCAGAAACATAGATGCACTCCTATGAACATGGAAAATATACCGGATTAGTCAATCCGACTTGAAATTCTTAAGTCGTTCATAACTGTTTAGTTAAAGTAAGAATTGCTTTTGATCGAACCATCTAGTTTCCTTTCAAGATTAATCAATTAGAATCCATTTTTGTTTTTTTATTTTTTNNNAGTTTATAGTTAAAACTAACTATTGCTCTTATGCAAATTCTCTTGTTTTCATCTCAATCTTACCGAGGATTCTCTCTAAAGAAAAGGGATTCCAAATAGAATTCTCATTTTTTTCTATTTTTTATTAGTTATAGTTAAGTTATTAGGAATTGGTCAAAATTGACATCGATTTGCATTATCTTTTCTATTTTATTATCNNNNNNNNNNNNNNTTTGTTTATATTTATTTCAATTTTTTATGAATATGTCCATTTGCCTCTTTTTTTAGTGGTTTAGAATAGAACAAGTAATAAAACGTCTAGGAATTTTAANNNNNNNNNNNNNNNNNNNTATCAAGTCAAGATTGATTTATAGTATAGAATTGGTATATTNNNNNNNNNNNNNNNNNNNNCCTTTTATTTATTATTTATACCTTTTATTTATTNNNNNNNNNNNNATTTAATTAGAATCCAATCTCGAGGGAGGATTTTCTATCGAAAGAAAAGAGAAGACTAGGTCTAAGTAAGGTATACGAAGGAAAGCCCCTTTTACGTTGTTGACAATGTTTACAATGAAACTTAAATTAGCAAATTGGTTTTTAAAGCTTTATCTTGTGCACAACTACCGCACAATACCTGCGTAAATTACTATTAGATTTGATTGGGGTTGGGTTTTTTTAACCAGACTCGGGTCATCATACAGAATTCACTCGAATCGGATATACCAAGGGTAGTATAATTAACTCGTTGATTCGATCTTTTCTCTCTTTTC

002_20160908_01_01_ndhCret-trnV-comp AATACCAAAATCGGAATAAGACTTGATATTATCAGAAATGCCCAAAAAATATCATATTCGTAAAGCAGAAACATAGATGCACTCCTATGAACATGGAAAATATACCGGATTAGTCAATCCGACTTGAAATTCTTAAGTCGTTCATAACTGTTTAGTTAAAGTAAGAATTGCTTTTGATCGAACCATCTAGTTTCCTTTCAAGATTAATCAATTAGAATCCATTTTTGTTTTTTTATTTTTTNNNAGTTTATAGTTAAAACTAACTATTGCTCTTATGCAAATTCTCTTGTTTTCATCTCAATCTTACCGAGGATTCTCTCTAAAGAAAAGGGATTCCAAATAGAATTCTCATTTTTTTCTATTTTTTATTAGTTATAGTTAAGTTATTAGGAATTGGTCAAAATTGACATCGATTTGCATTATCTTTTCTATTTTATTATCNNNNNNNNNNNNNNTTTGTTTATATTTATTTCAATTTTTTATGAATATGTCCATTTGCCTCTTTTTTTAGTGGTTTAGAATAGAACAAGTAATAAAACGTCTAGGAATTTTAANNNNNNNNNNNNNNNNNNNTATCAAGTCAAGATTGATTTATAGTATAGAATTGGTATATTNNNNNNNNNNNNNNNNNNNNCCTTTTATTTATTATTTATACCTTTTATTTATTNNNNNNNNNNNNATTTAATTAGAATCCAATCTCGAGGGAGGATTTTCTATCGAAAGAAAAGAGAAGACTAGGTCTAAGTAAGGTATACGAAGGAAAGCCCCTTTTACGTTGTTGACAATGTTTACAATGAAACTTAAATTAGCAAATTGGTTTTTAAAGCTTTATCTTGTGCACAACTACCGCACAATACCTGCGTAAATTACTATTAGATTTGATTGGGGTTGGGTTTTTTTAACCAGACTCGGGTCATCATACAGAATTCACTCGAATCGGATATACCAAGGGTAGTATAATTAACTCGTTGATTCGATCTTTTCTCTCTTTTC

003_20160908_01_02_ndhCret-trnV-comp AATACCAAAATCGGAATAAGACTTGATATTATCAGAAATGCCCAAAAAATATCATATTCGTAAAGCAGAAACATAGATGCACTCCTATGAACATGGAAAATATACCGGATTAGTCAATCCGACTTGAAATTCTTAAGTCGTTCATAACTGTTTAGTTAAAGTAAGAATTGCTTTTGATCGAACCATCTAGTTTCCTTTCAAGATTAATCAATTAGAATCCATTTTTGTTTTTTTATTTTTTNNNAGTTTATAGTTAAAACTAACTATTGCTCTTATGCAAATTCTCTTGTTTTCATCTCAATCTTACCGAGGATTCTCTCTAAAGAAAAGGGATTCCAAATAGAATTCTCATTTTTTTCTATTTTTTATTAGTTATAGTTAAGTTATTAGGAATTGGTCAAAATTGACATCGATTTGCATTATCTTTTCTATTTTATTATCNNNNNNNNNNNNNNTTTGTTTATATTTATTTCAATTTTTTATGAATATGTCCATTTGCCTCTTTTTTTAGTGGTTTAGAATAGAACAAGTAATAAAACGTCTAGGAATTTTAANNNNNNNNNNNNNNNNNNNTATCAAGTCAAGATTGATTTATAGTATAGAATTGGTATATTNNNNNNNNNNNNNNNNNNNNCCTTTTATTTATTATTTATACCTTTTATTTATTNNNNNNNNNNNNATTTAATTAGAATCCAATCTCGAGGGAGGATTTTCTATCGAAAGAAAAGAGAAGACTAGGTCTAAGTAAGGTATACGAAGGAAAGCCCCTTTTACGTTGTTGACAATGTTTACAATGAAACTTAAATTAGCAAATTGGTTTTTAAAGCTTTATCTTGTGCACAACTACCGCACAATACCTGCGTAAATTACTATTAGATTTGATTGGGGTTGGGTTTTTTTAACCAGACTCGGGTCATCATACAGAATTCACTCGAATCGGATATACCAAGGGTAGTATAATTAACTCGTTGATTCGATCTTTTCTCTCTTTTC

004_20160908_01_03_ndhCret-trnV-comp AATACCAAAATCGGAATAAGACTTGATATTATCAGAAATGCCCAAAAAATATCATATTCGTAAAGCAGAAACATAGATGCACTCCTATGAACATGGAAAATATACCGGATTAGTCAATCCGACTTGAAATTCTTAAGTCGTTCATAACTGTTTAGTTAAAGTAAGAATTGCTTTTGATCGAACCATCTAGTTTCCTTTCAAGATTAATCAATTAGAATCCATTTTTGTTTTTTTATTTTTTNNNAGTTTATAGTTAAAACTAACTATTGCTCTTATGCAAATTCTCTTGTTTTCATCTCAATCTTACCGAGGATTCTCTCTAAAGAAAAGGGATTCCAAATAGAATTCTCATTTTTTTCTATTTTTTATTAGTTATAGTTAAGTTATTAGGAATTGGTCAAAATTGACATCGATTTGCATTATCTTTTCTATTTTATTATCNNNNNNNNNNNNNNTTTGTTTATATTTATTTCAATTTTTTATGAATATGTCCATTTGCCTCTTTTTTTAGTGGTTTAGAATAGAACAAGTAATAAAACGTCTAGGAATTTTAANNNNNNNNNNNNNNNNNNNTATCAAGTCAAGATTGATTTATAGTATAGAATTGGTATATTNNNNNNNNNNNNNNNNNNNNCCTTTTATTTATTATTTATACCTTTTATTTATTNNNNNNNNNNNNATTTAATTAGAATCCAATCTCGAGGGAGGATTTTCTATCGAAAGAAAAGAGAAGACTAGGTCTAAGTAAGGTATACGAAGGAAAGCCCCTTTTACGTTGTTGACAATGTTTACAATGAAACTTAAATTAGCAAATTGGTTTTTAAAGCTTTATCTTGTGCACAACTACCGCACAATACCTGCGTAAATTACTATTAGATTTGATTGGGGTTGGGTTTTTTTAACCAGACTCGGGTCATCATACAGAATTCACTCGAATCGGATATACCAAGGGTAGTATAATTAACTCGTTGATTCGATCTTTTCTCTCTTTTC

005_20160908_01_04_ndhCret-trnV-comp AATACCAAAATCGGAATAAGACTTGATATTATCAGAAATGCCCAAAAAATATCATATTCGTAAAGCAGAAACATAGATGCACTCCTATGAACATGGAAAATATACCGGATTAGTCAATCCGACTTGAAATTCTTAAGTCGTTCATAACTGTTTAGTTAAAGTAAGAATTGCTTTTGATCGAACCATCTAGTTTCCTTTCAAGATTAATCAATTAGAATCCATTTTTGTTTTTTTATTTTTTNNNAGTTTATAGTTAAAACTAACTATTGCTCTTATGCAAATTCTCTTGTTTTCATCTCAATCTTACCGAGGATTCTCTCTAAAGAAAAGGGATTCCAAATAGAATTCTCATTTTTTTCTATTTTTTATTAGTTATAGTTAAGTTATTAGGAATTGGTCAAAATTGACATCGATTTGCATTATCTTTTCTATTTTATTATCNNNNNNNNNNNNNNTTTGTTTATATTTATTTCAATTTTTTATGAATATGTCCATTTGCCTCTTTTTTTAGTGGTTTAGAATAGAACAAGTAATAAAACGTCTAGGAATTTTAANNNNNNNNNNNNNNNNNNNTATCAAGTCAAGATTGATTTATAGTATAGAATTGGTATATTNNNNNNNNNNNNNNNNNNNNCCTTTTATTTATTATTTATACCTTTTATTTATTNNNNNNNNNNNNATTTAATTAGAATCCAATCTCGAGGGAGGATTTTCTATCGAAAGAAAAGAGAAGACTAGGTCTAAGTAAGGTATACGAAGGAAAGCCCCTTTTACGTTGTTGACAATGTTTACAATGAAACTTAAATTAGCAAATTGGTTTTTAAAGCTTTATCTTGTGCACAACTACCGCACAATACCTGCGTAAATTACTATTAGATTTGATTGGGGTTGGGTTTTTTTAACCAGACTCGGGTCATCATACAGAATTCACTCGAATCGGATATACCAAGGGTAGTATAATTAACTCGTTGATTCGATCTTTTCTCTCTTTTC

006_20160908_01_05_ndhCret-trnV-comp AATACCAAAATCGGAATAAGACTTGATATTATCAGAAATGCCCAAAAAATATCATATTCGTAAAGCAGAAACATAGATGCACTCCTATGAACATGGAAAATATACCGGATTAGTCAATCCGACTTGAAATTCTTAAGTCGTTCATAACTGTTTAGTTAAAGTAAGAATTGCTTTTGATCGAACCATCTAGTTTCCTTTCAAGATTAATCAATTAGAATCCATTTTTGTTTTTTTATTTTTTNNNAGTTTATAGTTAAAACTAACTATTGCTCTTATGCAAATTCTCTTGTTTTCATCTCAATCTTACCGAGGATTCTCTCTAAAGAAAAGGGATTCCAAATAGAATTCTCATTTTTTTCTATTTTTTATTAGTTATAGTTAAGTTATTAGGAATTGGTCAAAATTGACATCGATTTGCATTATCTTTTCTATTTTATTATCNNNNNNNNNNNNNNTTTGTTTATATTTATTTCAATTTTTTATGAATATGTCCATTTGCCTCTTTTTTTAGTGGTTTAGAATAGAACAAGTAATAAAACGTCTAGGAATTTTAANNNNNNNNNNNNNNNNNNNTATCAAGTCAAGATTGATTTATAGTATAGAATTGGTATATTNNNNNNNNNNNNNNNNNNNNCCTTTTATTTATTATTTATACCTTTTATTTATTNNNNNNNNNNNNATTTAATTAGAATCCAATCTCGAGGGAGGATTTTCTATCGAAAGAAAAGAGAAGACTAGGTCTAAGTAAGGTATACGAAGGAAAGCCCCTTTTACGTTGTTGACAATGTTTACAATGAAACTTAAATTAGCAAATTGGTTTTTAAAGCTTTATCTTGTGCACAACTACCGCACAATACCTGCGTAAATTACTATTAGATTTGATTGGGGTTGGGTTTTTTTAACCAGACTCGGGTCATCATACAGAATTCACTCGAATCGGATATACCAAGGGTAGTATAATTAACTCGTTGATTCGATCTTTTCTCTCTTTTC

007_20160909_02_01_ndhCret-trnV-comp AATACCAAAATCGGAATAAGACTTGATATTATCAGAAATGCCCAAAAAATATCATATTCGTAAAGCAGAAACATAGATGCACTCCTATGAACATGGAAAATATACCGGATTAGTCAATCCGACTTGAAATTCTTAAGTCGTTCATAACTGTTTAGTTAAAGTAAGAATTGCTTTTGATCGAACCATCTAGTTTCCTTTCAAGATTAATCAATTAGAATCCATTTTTGTTTTTTTATTTTTTNNNAGTTTATAGTTAAAACTAACTATTGCTCTTATGCAAATTCTCTTGTTTTCATCTCAATCTTACCGAGGATTCTCTCTAAAGAAAAGGGATTCCAAATAGAATTCTCATTTTTTTCTATTTTTTATTAGTTATAGTTAAGTTATTAGGAATTGGTCAAAATTGACATCGATTTGCATTATCTTTTCTATTTTATTATCNNNNNNNNNNNNNNTTTGTTTATATTTATTTCAATTTTTTATGAATATGTCCATTTGCCTCTTTTTTTAGTGGTTTAGAATAGAACAAGTAATAAAACGTCTAGGAATTTTAANNNNNNNNNNNNNNNNNNNTATCAAGTCAAGATTGATTTATAGTATAGAATTGGTATATTNNNNNNNNNNNNNNNNNNNNCCTTTTATTTATTATTTATACCTTTTATTTATTNNNNNNNNNNNNATTTAATTAGAATCCAATCTCGAGGGAGGATTTTCTATCGAAAGAAAAGAGAAGACTAGGTCTAAGTAAGGTATACGAAGGAAAGCCCCTTTTACGTTGTTGACAATGTTTACAATGAAACTTAAATTAGCAAATTGGTTTTTAAAGCTTTATCTTGTGCACAACTACCGCACAATACCTGCGTAAATTACTATTAGATTTGATTGGGGTTGGGTTTTTTTAACCAGACTCGGGTCATCATACAGAATTCACTCGAATCGGATATACCAAGGGTAGTATAATTAACTCGTTGATTCGATCTTTTCTCTCTTTTC

008_20160910_01_01_ndhCret-trnV-comp AATACCAAAATCGGAATAAGACTTGATATTATCAGAAATGCCCAAAAAATATCATATTCGTAAAGCAGAAACATAGATGCACTCCTATGAACATGGAAAATATACCGGATTAGTCAATCCGACTTGAAATTCTTAAGTCGTTCATAACTGTTTAGTTAAAGTAAGAATTGCTTTTGATCGAACCATCTAGTTTCCTTTCAAGATTAATCAATTAGAATCCATTTTTGTTTTTTTATTTTTTNNNAGTTTATAGTTAAAACTAACTATTGCTCTTATGCAAATTCTCTTGTTTTCATCTCAATCTTACCGAGGATTCTCTCTAAAGAAAAGGGATTCCAAATAGAATTCTCATTTTTTTCTATTTTTTATTAGTTATAGTTAAGTTATTAGGAATTGGTCAAAATTGACATCGATTTGCATTATCTTTTCTATTTTATTATCNNNNNNNNNNNNNNTTTGTTTATATTTATTTCAATTTTTTATGAATATGTCCATTTGCCTCTTTTTTTAGTGGTTTAGAATAGAACAAGTAATAAAACGTCTAGGAATTTTAANNNNNNNNNNNNNNNNNNNTATCAAGTCAAGATTGATTTATAGTATAGAATTGGTATATTNNNNNNNNNNNNNNNNNNNNCCTTTTATTTATTATTTATACCTTTTATTTATTNNNNNNNNNNNNATTTAATTAGAATCCAATCTCGAGGGAGGATTTTCTATCGAAAGAAAAGAGAAGACTAGGTCTAAGTAAGGTATACGAAGGAAAGCCCCTTTTACGTTGTTGACAATGTTTACAATGAAACTTAAATTAGCAAATTGGTTTTTAAAGCTTTATCTTGTGCACAACTACCGCACAATACCTGCGTAAATTACTATTAGATTTGATTGGGGTTGGGTTTTTTTAACCAGACTCGGGTCATCATACAGAATTCACTCGAATCGGATATACCAAGGGTAGTATAATTAACTCGTTGATTCGATCTTTTCTCTCTTTTC

009_20170910_07_01_ndhCret-trnV-comp AATACCAAAATCGGAATAAGACTTGATATTATCAGAAATGCCCAAAAAATATCATATTCGTAAAGCAGAAACATAGATGCACTCCTATGAACATGGAAAATATACCGGATTAGTCAATCCGACTTGAAATTCTTAAGTCGTTCATAACTGTTTAGTTAAAGTAAGAATTGCTTTTGATCGAACCATCTAGTTTCCTTTCAAGATTAATCAATTAGAATCCATTTTTGTTTTTTTATTTTTTTTTAGTTTTTAGTTAAAACTAACTATTGCTCTTATGCAAATTCTCTTGTTTTCATCTCAATCTTACCGAGGATTCTCTCTAAAGAAAAGGGATTCCAAATAGAATTCTCATTTTTTTCTATTTTTTATTAGTTATAGTTAAGTTATTAGGAATTGGTCAAAATTGACATCGATTTGCATTATCTTTTCTATTTTATTATCNNNNNNNNNNNNNNTTTGTTTATATTTATTTCAATTTTTTATGAATATGTCCATTTGCCTCTTTTTTTAGTGGTTTAGAATAGAACAAGTAATAAAACGTCTAGGAATTTTAANNNNNNNNNNNNNNNNNNNTATCAAGTCAAGATTGATTTATAGTATAGAATTGGTATATTNNNNNNNNNNNNNNNNNNNNCCTTTTATTTATTATTTATACCTTTTATTTATTNNNNNNNNNNNNATTTAATTAGAATCCAATCTCGAGGGAGGATTTTCTATCGAAAGAAAAGAGAAGACTAGGTCTAAGTAAGGTATACGAAGGAAAGCCCCTTTTACGTTGTTGACAATGTTTACAATGAAACTTAAATTAGCAAATTGGTTTTTAAAGCTTTATCTTGTGCACAACTACCGCACAATACCTGCGTAAATTACTATTAGATTTGATTGGGGTTGGGTTTTTTTAACCAGACTCGGGTCATCATACAGAATTCACTCGAATCGGATATACCAAGGGTAGTATAATTAACTCGTTGATTCGATCTTTTCTCTCCTTTC

010_20170910_07_02_ndhCret-trnV-comp AATACCAAAATCGGAATAAGACTTGATATTATCAGAAATGCCCAAAAAATATCATATTCGTAAAGCAGAAACATAGATGCACTCCTATGAACATGGAAAATATACCGGATTAGTCAATCCGACTTGAAATTCTTAAGTCGTTCATAACTGTTTAGTTAAAGTAAGAATTGCTTTTGATCGAACCATCTAGTTTCCTTTCAAGATTAATCAATTAGAATCCATTTTTGTTTTTTTATTTTTTNNNAGTTTATAGTTAAAACTAACTATTGCTCTTATGCAAATTCTCTTGTTTTCATCTCAATCTTACCGAGGATTCTCTCTAAAGAAAAGGGATTCCAAATAGAATTCTCATTTTTTTCTATTTTTTATTAGTTATAGTTAAGTTATTAGGAATTGGTCAAAATTGACATCGATTTGCATTATCTTTTCTATTTTATTATCNNNNNNNNNNNNNNTTTGTTTATATTTATTTCAATTTTTTATGAATATGTCCATTTGCCTCTTTTTTTAGTGGTTTAGAATAGAACAAGTAATAAAACGTCTAGGAATTTTAANNNNNNNNNNNNNNNNNNNTATCAAGTCAAGATTGATTTATAGTATAGAATTGGTATATTNNNNNNNNNNNNNNNNNNNNCCTTTTATTTATTATTTATACCTTTTATTTATTNNNNNNNNNNNNATTTAATTAGAATCCAATCTCGAGGGAGGATTTTCTATCGAAAGAAAAGAGAAGACTAGGTCTAAGTAAGGTATACGAAGGAAAGCCCCTTTTACGTTGTTGACAATGTTTACAATGAAACTTAAATTAGCAAATTGGTTTTTAAAGCTTTATCTTGTGCACAACTACCGCACAATACCTGCGTAAATTACTATTAGATTTGATTGGGGTTGGGTTTTTTTAACCAGACTCGGGTCATCATACAGAATTCACTCGAATCGGATATACCAAGGGTAGTATAATTAACTCGTTGATTCGATCTTTTCTCTCTTTTC

011_20170910_07_03_ndhCret-trnV-comp AATACCAAAATCGGAATAAGACTTGATATTATCAGAAATGCCCAAAAAATATCATATTCGTAAAGCAGAAACATAGATGCACTCCTATGAACATGGAAAATATACCGGATTAGTCAATCCGACTTGAAATTCTTAAGTCGTTCATAACTGTTTAGTTAAAGTAAGAATTGCTTTTGATCGAACCATCTAGTTTCCTTTCAAGATTAATCAATTAGAATCCATTTTTGTTTTTTTATTTTTTNNNAGTTTATAGTTAAAACTAACTATTGCTCTTATGCAAATTCTCTTGTTTTCATCTCAATCTTACCGAGGATTCTCTCTAAAGAAAAGGGATTCCAAATAGAATTCTCATTTTTTTCTATTTTTTATTAGTTATAGTTAAGTTATTAGGAATTGGTCAAAATTGACATCGATTTGCATTATCTTTTCTATTTTATTATCNNNNNNNNNNNNNNTTTGTTTATATTTATTTCAATTTTTTATGAATATGTCCATTTGCCTCTTTTTTTAGTGGTTTAGAATAGAACAAGTAATAAAACGTCTAGGAATTTTAANNNNNNNNNNNNNNNNNNNTATCAAGTCAAGATTGATTTATAGTATAGAATTGGTATATTNNNNNNNNNNNNNNNNNNNNCCTTTTATTTATTATTTATACCTTTTATTTATTNNNNNNNNNNNNATTTAATTAGAATCCAATCTCGAGGGAGGATTTTCTATCGAAAGAAAAGAGAAGACTAGGTCTAAGTAAGGTATACGAAGGAAAGCCCCTTTTACGTTGTTGACAATGTTTACAATGAAACTTAAATTAGCAAATTGGTTTTTAAAGCTTTATCTTGTGCACAACTACCGCACAATACCTGCGTAAATTACTATTAGATTTGATTGGGGTTGGGTTTTTTTAACCAGACTCGGGTCATCATACAGAATTCACTCGAATCGGATATACCAAGGGTAGTATAATTAACTCGTTGATTCGATCTTTTCTCTCTTTTC

012_20170910_07_04_ndhCret-trnV-comp AATACCAAAATCGGAATAAGACTTGATATTATCAGAAATGCCCAAAAAATATCATATTCGTAAAGCAGAAACATAGATGCACTCCTATGAACATGGAAAATATACCGGATTAGTCAATCCGACTTGAAATTCTTAAGTCGTTCATAACTGTTTAGTTAAAGTAAGAATTGCTTTTGATCGAACCATCTAGTTTCCTTTCAAGATTAATCAATTAGAATCCATTTTTGTTTTTTTATTTTTTTTTAGTTTATAGTTAAAACTAACTATTGCTCTTATGCAAATTCTCTTGTTTTCATCTCAATCTTACCGAGGATTCTCTCTAAAGAAAAGGGATTCCAAATAGAATTCTCATTTTTTTCTATTTTTTATTAGTTATAGTTAAGTTATTAGGAATTGGTCAAAATTGACATCGATTTGCATTATCTTTTCTATTTTATTATCNNNNNNNNNNNNNNTTTGTTTATATTTATTTCAATTTTTTATGAATATGTCCATTTGCCTCTTTTTTTAGTGGTTTAGAATAGAACAAGTAATAAAACGTCTAGGAATTTTAATATCAAGTCAAGATTGATTTATCAAGTCAAGATTGATTTATAGTATAGAATTGGTATATTCCTTTTATTTATTATTTATACCTTTTATTTATTATTTATACCTTTTATTTATTNNNNNNNNNNNNATTTAATTAGAATCCAATCTCGAGGGAGTATTTTCTATCGAAAGAAAAGAGAAGACTAGGTCTAAGTAAGGTATACGAAGGAAAGCCCCTTTTACGTTGTTGACAATGTTTACAATGAAACTTAAATTAGCAAATTGGTTTTTAAAGCTTTATCTTGTGCACAACTACCGCACAATACCTGCGTAAATTACTATTAGATTTGATTGGGGTTGGGTTTTTTTAACCAGACTCGGGTCATCATACAGAATTCACTCGAATCGGATATACCAAGGGTAGTATAATTAACTCGTTGATTCGATCTTCTCTCTCCTCTC

013_20170910_07_05_ndhCret-trnV-comp AATACCAAAATCGGAATAAGACTTGATATTATCAGAAATGCCCAAAAAATATCATATTCGTAAAGCAGAAACATAGATGCACTCCTATGAACATGGAAAATATACCGGATTAGTCAATCCGACTTGAAATTCTTAAGTCGTTCATAACTGTTTAGTTAAAGTAAGAATTGCTTTTGATCGAACCATCTAGTTTCCTTTCAAGATTAATCAATTAGAATCCATTTTTGTTTTTTTATTTTTTTTTAGTTTATAGTTAAAACTAACTATTGCTCTTATGCAAATTCTCTTGTTTTCATCTCAATCTTACCGAGGATTCTCTCTAAAGAAAAGGGATTCCAAATAGAATTCTCATTTTTTTCTATTTTTTATTAGTTATAGTTAAGTTATTAGGAATTGGTCAAAATTGACATCGATTTGCATTATCTTTTCTATTTTATTATCNNNNNNNNNNNNNNTTTGTTTATATTTATTTCAATTTTTTATGAATATGTCCATTTGCCTCTTTTTTTAGTGGTTTAGAATAGAACAAGTAATAAAACGTCTAGGAATTTTAATATCAAGTCAAGATTGATTTATCAAGTCAAGATTGATTTATAGTATAGAATTGGTATATTCCTTTTATTTATTATTTATACCTTTTATTTATTATTTATACCTTTTATTTATTNNNNNNNNNNNNATTTAATTAGAATCCAATCTCGAGGGAGTATTTTCTATCGAAAGAAAAGAGAAGACTAGGTCTAAGTAAGGTATACGAAGGAAAGCCCCTTTTACGTTGTTGACAATGTTTACAATGAAACTTAAATTAGCAAATTGGTTTTTAAAGCTTTATCTTGTGCACAACTACCGCACAATACCTGCGTAAATTACTATTAGATTTGATTGGGGTTGGGTTTTTTTAACCAGACTCGGGTCATCATACAGAATTCACTCGAATCGGATATACCAAGGGTAGTATAATTAACTCGTTGATTCGATCTTCTCTCTCCTCTC

014_20170916_04_01_ndhCret-trnV-comp AATACCAAAATCGGAATAAGACTTGATATTATCAGAAATGCCCAAAAAATATCATATTCGTAAAGCAGAAACATAGATGCACTCCTATGAACATGGAAAATATACCGGATTAGTCAATCCGACTTGAAATTCTTAAGTCGTTCATAACTGTTTAGTTAAAGTAAGAATTGCTTTTGATCGAACCATCTAGTTTCCTTTCAAGATTAATCAATTAGAATCCATTTTTGTTTTTTTATTTTTTTTTAGTTTTTAGTTAAAACTAACTATTGCTCTTATGCAAATTCTCTTGTTTTCATCTCAATCTTACCGAGGATTCTCTCTAAAGAAAAGGGATTCCAAATAGAATTCTCATTTTTTTCTATTTTTTATTAGTTATAGTTAAGTTATTAGGAATTGGTCAAAATTGACATCGATTTGCATTATCTTTTCTATTTTATTATCNNNNNNNNNNNNNNTTTGTTTATATTTATTTCAATTTTTTATGAATATGTCCATTTGCCTCTTTTTTTAGTGGTTTAGAATAGAACAAGTAATAAAACGTCTAGGAATTTTAANNNNNNNNNNNNNNNNNNNTATCAAGTCAAGATTGATTTATAGTATAGAATTGGTATATTNNNNNNNNNNNNNNNNNNNNCCTTTTATTTATTATTTATACCTTTTATTTATTNNNNNNNNNNNNATTTAATTAGAATCCAATCTCGAGGGAGGATTTTCTATCGAAAGAAAAGAGAAGACTAGGTCTAAGTAAGGTATACGAAGGAAAGCCCCTTTTACGTTGTTGACAATGTTTACAATGAAACTTAAATTAGCAAATTGGTTTTTAAAGCTTTATCTTGTGCACAACTACCGCACAATACCTGCGTAAATTACTATTAGATTTGATTGGGGTTGGGTTTTTTTAACCAGACTCGGGTCATCATACAGAATTCACTCGAATCGGATATACCAAGGGTAGTATAATTAACTCGTTGATTCGATCTTTTCTCTCCTTTC

015_20170916_04_02_ndhCret-trnV-comp AATACCAAAATCGGAATAAGACTTGATATTATCAGAAATGCCCAAAAAATATCATATTCGTAAAGCAGAAACATAGATGCACTCCTATGAACATGGAAAATATACCGGATTAGTCAATCCGACTTGAAATTCTTAAGTCGTTCATAACTGTTTAGTTAAAGTAAGAATTGCTTTTGATCGAACCATCTAGTTTCCTTTCAAGATTAATCAATTAGAATCCATTTTTGTTTTTTTATTTTTTTTTAGTTTTTAGTTAAAACTAACTATTGCTCTTATGCAAATTCTCTTGTTTTCATCTCAATCTTACCGAGGATTCTCTCTAAAGAAAAGGGATTCCAAATAGAATTCTCATTTTTTTCTATTTTTTATTAGTTATAGTTAAGTTATTAGGAATTGGTCAAAATTGACATCGATTTGCATTATCTTTTCTATTTTATTATCNNNNNNNNNNNNNNTTTGTTTATATTTATTTCAATTTTTTATGAATATGTCCATTTGCCTCTTTTTTTAGTGGTTTAGAATAGAACAAGTAATAAAACGTCTAGGAATTTTAANNNNNNNNNNNNNNNNNNNTATCAAGTCAAGATTGATTTATAGTATAGAATTGGTATATTNNNNNNNNNNNNNNNNNNNNCCTTTTATTTATTATTTATACCTTTTATTTATTNNNNNNNNNNNNATTTAATTAGAATCCAATCTCGAGGGAGGATTTTCTATCGAAAGAAAAGAGAAGACTAGGTCTAAGTAAGGTATACGAAGGAAAGCCCCTTTTACGTTGTTGACAATGTTTACAATGAAACTTAAATTAGCAAATTGGTTTTTAAAGCTTTATCTTGTGCACAACTACCGCACAATACCTGCGTAAATTACTATTAGATTTGATTGGGGTTGGGTTTTTTTAACCAGACTCGGGTCATCATACAGAATTCACTCGAATCGGATATACCAAGGGTAGTATAATTAACTCGTTGATTCGATCTTTTCTCTCCTTTC

016_20170916_04_03_ndhCret-trnV-comp AATACCAAAATCGGAATAAGACTTGATATTATCAGAAATGCCCAAAAAATATCATATTCGTAAAGCAGAAACATAGATGCACTCCTATGAACATGGAAAATATACCGGATTAGTCAATCCGACTTGAAATTCTTAAGTCGTTCATAACTGTTTAGTTAAAGTAAGAATTGCTTTTGATCGAACCATCTAGTTTCCTTTCAAGATTAATCAATTAGAATCCATTTTTGTTTTTTTATNNNNNNNNNNTTTTTAGTTAAAACTAACTATTGCTCTTATGCAAATTCTCTTGTTTTCATCTCAATCTTACCGAGGATTCTCTCTAAAGAAAAGGGATTCCAAATAGAATTCTCATTTTTTTCTATTTTTTATTAGTTATAGTTAAGTTATTAGGAATTGGTCAAAATTGACATCGATTTGCATTATCTTTTCTATTTTATTATCNNNNNNNNNNNNNNTTTGTTTATATTTATTTCAATTTTTTATGAATATGTCCATTTGCCTCTTTTTTTAGTGGTTTAGAATAGAACAAGTAATAAAACGTCTAGGAATTTTAANNNNNNNNNNNNNNNNNNNTATCAAGTCAAGATTGATTTATAGTATAGAATTGGTATATTNNNNNNNNNNNNNNNNNNNNCCTTTTATTTATTATTTATACCTTTTATTTATTNNNNNNNNNNNNATTTAATTAGAATCCAATCTCGAGGGAGGATTTTCTATCGAAAGAAAAGAGAAGACTAGGTCTAAGTAAGGTATACGAAGGAAAGCCCCTTTTACGTTGTTGACAATGTTTACAATGAAACTTAAATTAGCAAATTGGTTTTTAAAGCTTTATCTTGTGCACAACTACCGCACAATACCTGCGTAAATTACTATTAGATTTGATTGGGGTTGGGTTTTTTTAACCAGACTCGGGTCATCATACAGAATTCACTCGAATCGGATATACCAAGGGTAGTATAATTAACTCGTTGATTCGATCTTTTCTCTTNTTTC

017_20170916_05_01_ndhCret-trnV-comp AATACCAAAATCGGAATAAGACTTGATATTATCAGAAATGCCCAAAAAATATCATATTCGTAAAGCAGAAACATAGATGCACTCCTATGAACATGGAAAATATACCGGATTAGTCAATCCGACTTGAAATTCTTAAGTCGTTCATAACTGTTTAGTTAAAGTAAGAATTGCTTTTGATCGAACCATCTAGTTTCCTTTCAAGATTAATCAATTAGAATCCATTTTTGTTTTTTTATTTTTTTTTAGTTTTTAGTTAAAACTAACTATTGCTCTTATGCAAATTCTCTTGTTTTCATCTCAATCTTACCGAGGATTCTCTCTAAAGAAAAGGGATTCCAAATAGAATTCTCATTTTTTTCTATTTTTTATTAGTTATAGTTAAGTTATTAGGAATTGGTCAAAATTGACATCGATTTGCATTATCTTTTCTATTTTATTATCNNNNNNNNNNNNNNTTTGTTTATATTTATTTCAATTTTTTATGAATATGTCCATTTGCCTCTTTTTTTAGTGGTTTAGAATAGAACAAGTAATAAAACGTCTAGGAATTTTAANNNNNNNNNNNNNNNNNNNTATCAAGTCAAGATTGATTTATAGTATAGAATTGGTATATTNNNNNNNNNNNNNNNNNNNNCCTTTTATTTATTATTTATACCTTTTATTTATTNNNNNNNNNNNNATTTAATTAGAATCCAATCTCGAGGGAGGATTTTCTATCGAAAGAAAAGAGAAGACTAGGTCTAAGTAAGGTATACGAAGGAAAGCCCCTTTTACGTTGTTGACAATGTTTACAATGAAACTTAAATTAGCAAATTGGTTTTTAAAGCTTTATCTTGTGCACAACTACCGCACAATACCTGCGTAAATTACTATTAGATTTGATTGGGGTTGGGTTTTTTTAACCAGACTCGGGTCATCATACAGAATTCACTCGAATCGGATATACCAAGGGTAGTATAATTAACTCGTTGATTCGATCTTTTCTCTCCTTTC

018_20170916_05_02_ndhCret-trnV-comp AATACCAAAATCGGAATAAGACTTGATATTATCAGAAATGCCCAAAAAATATCATATTCGTAAAGCAGAAACATAGATGCACTCCTATGAACATGGAAAATATACCGGATTAGTCAATCCGACTTGAAATTCTTAAGTCGTTCATAACTGTTTAGTTAAAGTAAGAATTGCTTTTGATCGAACCATCTAGTTTCCTTTCAAGATTAATCAATTAGAATCCATTTTTGTTTTTTTATTTTTTTTTAGTTTTTAGTTAAAACTAACTATTGCTCTTATGCAAATTCTCTTGTTTTCATCTCAATCTTACCGAGGATTCTCTCTAAAGAAAAGGGATTCCAAATAGAATTCTCATTTTTTTCTATTTTTTATTAGTTATAGTTAAGTTATTAGGAATTGGTCAAAATTGACATCGATTTGCATTATCTTTTCTATTTTATTATCNNNNNNNNNNNNNNTTTGTTTATATTTATTTCAATTTTTTATGAATATGTCCATTTGCCTCTTTTTTTAGTGGTTTAGAATAGAACAAGTAATAAAACGTCTAGGAATTTTAANNNNNNNNNNNNNNNNNNNTATCAAGTCAAGATTGATTTATAGTATAGAATTGGTATATTNNNNNNNNNNNNNNNNNNNNCCTTTTATTTATTATTTATACCTTTTATTTATTNNNNNNNNNNNNATTTAATTAGAATCCAATCTCGAGGGAGGATTTTCTATCGAAAGAAAAGAGAAGACTAGGTCTAAGTAAGGTATACGAAGGAAAGCCCCTTTTACGTTGTTGACAATGTTTACAATGAAACTTAAATTAGCAAATTGGTTTTTAAAGCTTTATCTTGTGCACAACTACCGCACAATACCTGCGTAAATTACTATTAGATTTGATTGGGGTTGGGTTTTTTTAACCAGACTCGGGTCATCATACAGAATTCACTCGAATCGGATATACCAAGGGTAGTATAATTAACTCGTTGATTCGATCTTTTCTCTCCTTTC

019_20170916_06_01_ndhCret-trnV-comp AATACCAAAATCGGAATAAGACTTGATATTATCAGAAATGCCCAAAAAATATCATATTCGTAAAGCAGAAACATAGATGCACTCCTATGAACATGGAAAATATACCGGATTAGTCAATCCGACTTGAAATTCTTAAGTCGTTCATAACTGTTTAGTTAAAGTAAGAATTGCTTTTGATCGAACCATCTAGTTTCCTTTCAAGATTAATCAATTAGAATCCATTTTTGTTTTTTTATTTTTTTTTAGTTTTTAGTTAAAACTAACTATTGCTCTTATGCAAATTCTCTTGTTTTCATCTCAATCTTACCGAGGATTCTCTCTAAAGAAAAGGGATTCCAAATAGAATTCTCATTTTTTTCTATTTTTTATTAGTTATAGTTAAGTTATTAGGAATTGGTCAAAATTGACATCGATTTGCATTATCTTTTCTATTTTATTATCNNNNNNNNNNNNNNTTTGTTTATATTTATTTCAATTTTTTATGAATATGTCCATTTGCCTCTTTTTTTAGTGGTTTAGAATAGAACAAGTAATAAAACGTCTAGGAATTTTAANNNNNNNNNNNNNNNNNNNTATCAAGTCAAGATTGATTTATAGTATAGAATTGGTATATTNNNNNNNNNNNNNNNNNNNNCCTTTTATTTATTATTTATACCTTTTATTTATTNNNNNNNNNNNNATTTAATTAGAATCCAATCTCGAGGGAGGATTTTCTATCGAAAGAAAAGAGAAGACTAGGTCTAAGTAAGGTATACGAAGGAAAGCCCCTTTTACGTTGTTGACAATGTTTACAATGAAACTTAAATTAGCAAATTGGTTTTTAAAGCTTTATCTTGTGCACAACTACCGCACAATACCTGCGTAAATTACTATTAGATTTGATTGGGGTTGGGTTTTTTTAACCAGACTCGGGTCATCATACAGAATTCACTCGAATCGGATATACCAAGGGTAGTATAATTAACTCGTTGATTCGATCTTTTCTCTCCTTTC

020_20170916_06_02_ndhCret-trnV-comp AATACCAAAATCGGAATAAGACTTGATATTATCAGAAATGCCCAAAAAATATCATATTCGTAAAGCAGAAACATAGATGCACTCCTATGAACATGGAAAATATACCGGATTAGTCAATCCGACTTGAAATTCTTAAGTCGTTCATAACTGTTTAGTTAAAGTAAGAATTGCTTTTGATCGAACCATCTAGTTTCCTTTCAAGATTAATCAATTAGAATCCATTTTTGTTTTTTTATTTTTTTTTAGTTTTTAGTTAAAACTAACTATTGCTCTTATGCAAATTCTCTTGTTTTCATCTCAATCTTACCGAGGATTCTCTCTAAAGAAAAGGGATTCCAAATAGAATTCTCATTTTTTTCTATTTTTTATTAGTTATAGTTAAGTTATTAGGAATTGGTCAAAATTGACATCGATTTGCATTATCTTTTCTATTTTATTATCNNNNNNNNNNNNNNTTTGTTTATATTTATTTCAATTTTTTATGAATATGTCCATTTGCCTCTTTTTTTAGTGGTTTAGAATAGAACAAGTAATAAAACGTCTAGGAATTTTAANNNNNNNNNNNNNNNNNNNTATCAAGTCAAGATTGATTTATAGTATAGAATTGGTATATTNNNNNNNNNNNNNNNNNNNNCCTTTTATTTATTATTTATACCTTTTATTTATTNNNNNNNNNNNNATTTAATTAGAATCCAATCTCGAGGGAGGATTTTCTATCGAAAGAAAAGAGAAGACTAGGTCTAAGTAAGGTATACGAAGGAAAGCCCCTTTTACGTTGTTGACAATGTTTACAATGAAACTTAAATTAGCAAATTGGTTTTTAAAGCTTTATCTTGTGCACAACTACCGCACAATACCTGCGTAAATTACTATTAGATTTGATTGGGGTTGGGTTTTTTTAACCAGACTCGGGTCATCATACAGAATTCACTCGAATCGGATATACCAAGGGTAGTATAATTAACTCGTTGATTCGATCTTTTCTCTCCTTTC

021_20170917_02_01_ndhCret-trnV-comp AATACCAAAATCGGAATAAGACTTGATATTATCAGAAATGCCCAAAAAATATCATATTCGTAAAGCAGAAACATAGATGCACTCCTATGAACATGGAAAATATACCGGATTAGTCAATCCGACTTGAAATTCTTAAGTCGTTCATAACTGTTTAGTTAAAGTAAGAATTGCTTTTGATCGAACCATCTAGTTTCCTTTCAAGATTAATCAATTAGAATCCATTTTTGTTTTTTTATTTTTTNNNAGTTTATAGTTAAAACTAACTATTGCTCTTATGCAAATTCTCTTGTTTTCATCTCAATCTTACCGAGGATTCTCTCTAAAGAAAAGGGATTCCAAATAGAATTCTCATTTTTTTCTATTTTTTATTAGTTATAGTTAAGTTATTAGGAATTGGTCAAAATTGACATCGATTTGCATTATCTTTTCTATTTTATTATCNNNNNNNNNNNNNNTTTGTTTATATTTATTTCAATTTTTTATGAATATGTCCATTTGCCTCTTTTTTTAGTGGTTTAGAATAGAACAAGTAATAAAACGTCTAGGAATTTTAANNNNNNNNNNNNNNNNNNNTATCAAGTCAAGATTGATTTATAGTATAGAATTGGTATATTNNNNNNNNNNNNNNNNNNNNCCTTTTATTTATTATTTATACCTTTTATTTATTNNNNNNNNNNNNATTTAATTAGAATCCAATCTCGAGGGAGGATTTTCTATCGAAAGAAAAGAGAAGACTAGGTCTAAGTAAGGTATACGAAGGAAAGCCCCTTTTACGTTGTTGACAATGTTTACAATGAAACTTAAATTAGCAAATTGGTTTTTAAAGCTTTATCTTGTGCACAACTACCGCACAATACCTGCGTAAATTACTATTAGATTTGATTGGGGTTGGGTTTTTTTAACCAGACTCGGGTCATCATACAGAATTCACTCGAATCGGATATACCAAGGGTAGTATAATTAACTCGTTGATTCGATCTTTTCTCTCTTTTC

022_20170917_02_02_ndhCret-trnV-comp AATACCAAAATCGGAATAAGACTTGATATTATCAGAAATGCCCAAAAAATATCATATTCGTAAAGCAGAAACATAGATGCACTCCTATGAACATGGAAAATATACCGGATTAGTCAATCCGACTTGAAATTCTTAAGTCGTTCATAACTGTTTAGTTAAAGTAAGAATTGCTTTTGATCGAACCATCTAGTTTCCTTTCAAGATTAATCAATTAGAATCCATTTTTGTTTTTTTATTTTTTNNNAGTTTATAGTTAAAACTAACTATTGCTCTTATGCAAATTCTCTTGTTTTCATCTCAATCTTACCGAGGATTCTCTCTAAAGAAAAGGGATTCCAAATAGAATTCTCATTTTTTTCTATTTTTTATTAGTTATAGTTAAGTTATTAGGAATTGGTCAAAATTGACATCGATTTGCATTATCTTTTCTATTTTATTATCNNNNNNNNNNNNNNTTTGTTTATATTTATTTCAATTTTTTATGAATATGTCCATTTGCCTCTTTTTTTAGTGGTTTAGAATAGAACAAGTAATAAAACGTCTAGGAATTTTAANNNNNNNNNNNNNNNNNNNTATCAAGTCAAGATTGATTTATAGTATAGAATTGGTATATTNNNNNNNNNNNNNNNNNNNNCCTTTTATTTATTATTTATACCTTTTATTTATTNNNNNNNNNNNNATTTAATTAGAATCCAATCTCGAGGGAGGATTTTCTATCGAAAGAAAAGAGAAGACTAGGTCTAAGTAAGGTATACGAAGGAAAGCCCCTTTTACGTTGTTGACAATGTTTACAATGAAACTTAAATTAGCAAATTGGTTTTTAAAGCTTTATCTTGTGCACAACTACCGCACAATACCTGCGTAAATTACTATTAGATTTGATTGGGGTTGGGTTTTTTTAACCAGACTCGGGTCATCATACAGAATTCACTCGAATCGGATATACCAAGGGTAGTATAATTAACTCGTTGATTCGATCTTTTCTCTCTTTTC

023_20170917_02_03_ndhCret-trnV-comp AATACCAAAATCGGAATAAGACTTGATATTATCAGAAATGCCCAAAAAATATCATATTCGTAAAGCAGAAACATAGATGCACTCCTATGAACATGGAAAATATACCGGATTAGTCAATCCGACTTGAAATTCTTAAGTCGTTCATAACTGTTTAGTTAAAGTAAGAATTGCTTTTGATCGAACCATCTAGTTTCCTTTCAAGATTAATCAATTAGAATCCATTTTTGTTTTTTTATTTTTTNNNAGTTTATAGTTAAAACTAACTATTGCTCTTATGCAAATTCTCTTGTTTTCATCTCAATCTTACCGAGGATTCTCTCTAAAGAAAAGGGATTCCAAATAGAATTCTCATTTTTTTCTATTTTTTATTAGTTATAGTTAAGTTATTAGGAATTGGTCAAAATTGACATCGATTTGCATTATCTTTTCTATTTTATTATCNNNNNNNNNNNNNNTTTGTTTATATTTATTTCAATTTTTTATGAATATGTCCATTTGCCTCTTTTTTTAGTGGTTTAGAATAGAACAAGTAATAAAACGTCTAGGAATTTTAANNNNNNNNNNNNNNNNNNNTATCAAGTCAAGATTGATTTATAGTATAGAATTGGTATATTNNNNNNNNNNNNNNNNNNNNCCTTTTATTTATTATTTATACCTTTTATTTATTNNNNNNNNNNNNATTTAATTAGAATCCAATCTCGAGGGAGGATTTTCTATCGAAAGAAAAGAGAAGACTAGGTCTAAGTAAGGTATACGAAGGAAAGCCCCTTTTACGTTGTTGACAATGTTTACAATGAAACTTAAATTAGCAAATTGGTTTTTAAAGCTTTATCTTGTGCACAACTACCGCACAATACCTGCGTAAATTACTATTAGATTTGATTGGGGTTGGGTTTTTTTAACCAGACTCGGGTCATCATACAGAATTCACTCGAATCGGATATACCAAGGGTAGTATAATTAACTCGTTGATTCGATCTTTTCTCTCTTTTC

024_20170917_05_01_ndhCret-trnV-comp AATACCAAAATCGGAATAAGACTTGATATTATCAGAAATGCCCAAAAAATATCATATTCGTAAAGCAGAAACATAGATGCACTCCTATGAACATGGAAAATATACCGGATTAGTCAATCCGACTTGAAATTCTTAAGTCGTTCATAACTGTTTAGTTAAAGTAAGAATTGCTTTTGATCGAACCATCTAGTTTCCTTTCAAGATTAATCAATTAGAATCCATTTTTGTTTTTTTATTTTTTNNNAGTTTATAGTTAAAACTAACTATTGCTCTTATGCAAATTCTCTTGTTTTCATCTCAATCTTACCGAGGATTCTCTCTAAAGAAAAGGGATTCCAAATAGAATTCTCATTTTTTTCTATTTTTTATTAGTTATAGTTAAGTTATTAGGAATTGGTCAAAATTGACATCGATTTGCATTATCTTTTCTATTTTATTATCNNNNNNNNNNNNNNTTTGTTTATATTTATTTCAATTTTTTATGAATATGTCCATTTGCCTCTTTTTTTAGTGGTTTAGAATAGAACAAGTAATAAAACGTCTAGGAATTTTAANNNNNNNNNNNNNNNNNNNTATCAAGTCAAGATTGATTTATAGTATAGAATTGGTATATTNNNNNNNNNNNNNNNNNNNNCCTTTTATTTATTATTTATACCTTTTATTTATTNNNNNNNNNNNNATTTAATTAGAATCCAATCTCGAGGGAGGATTTTCTATCGAAAGAAAAGAGAAGACTAGGTCTAAGTAAGGTATACGAAGGAAAGCCCCTTTTACGTTGTTGACAATGTTTACAATGAAACTTAAATTAGCAAATTGGTTTTTAAAGCTTTATCTTGTGCACAACTACCGCACAATACCTGCGTAAATTACTATTAGATTTGATTGGGGTTGGGTTTTTTTAACCAGACTCGGGTCATCATACAGAATTCACTCGAATCGGATATACCAAGGGTAGTATAATTAACTCGTTGATTCGATCTTTTCTCTCTTTTC

025_20170917_05_02_ndhCret-trnV-comp AATACCAAAATCGGAATAAGACTTGATATTATCAGAAATGCCCAAAAAATATCATATTCGTAAAGCAGAAACATAGATGCACTCCTATGAACATGGAAAATATACCGGATTAGTCAATCCGACTTGAAATTCTTAAGTCGTTCATAACTGTTTAGTTAAAGTAAGAATTGCTTTTGATCGAACCATCTAGTTTCCTTTCAAGATTAATCAATTAGAATCCATTTTTGTTTTTTTATTTTTTNNNAGTTTATAGTTAAAACTAACTATTGCTCTTATGCAAATTCTCTTGTTTTCATCTCAATCTTACCGAGGATTCTCTCTAAAGAAAAGGGATTCCAAATAGAATTCTCATTTTTTTCTATTTTTTATTAGTTATAGTTAAGTTATTAGGAATTGGTCAAAATTGACATCGATTTGCATTATCTTTTCTATTTTATTATCNNNNNNNNNNNNNNTTTGTTTATATTTATTTCAATTTTTTATGAATATGTCCATTTGCCTCTTTTTTTAGTGGTTTAGAATAGAACAAGTAATAAAACGTCTAGGAATTTTAANNNNNNNNNNNNNNNNNNNTATCAAGTCAAGATTGATTTATAGTATAGAATTGGTATATTNNNNNNNNNNNNNNNNNNNNCCTTTTATTTATTATTTATACCTTTTATTTATTNNNNNNNNNNNNATTTAATTAGAATCCAATCTCGAGGGAGGATTTTCTATCGAAAGAAAAGAGAAGACTAGGTCTAAGTAAGGTATACGAAGGAAAGCCCCTTTTACGTTGTTGACAATGTTTACAATGAAACTTAAATTAGCAAATTGGTTTTTAAAGCTTTATCTTGTGCACAACTACCGCACAATACCTGCGTAAATTACTATTAGATTTGATTGGGGTTGGGTTTTTTTAACCAGACTCGGGTCATCATACAGAATTCACTCGAATCGGATATACCAAGGGTAGTATAATTAACTCGTTGATTCGATCTTTTCTCTCTTTTC

026_20170917_05_03_ndhCret-trnV-comp AATACCAAAATCGGAATAAGACTTGATATTATCAGAAATGCCCAAAAAATATCATATTCGTAAAGCAGAAACATAGATGCACTCCTATGAACATGGAAAATATACCGGATTAGTCAATCCGACTTGAAATTCTTAAGTCGTTCATAACTGTTTAGTTAAAGTAAGAATTGCTTTTGATCGAACCATCTAGTTTCCTTTCAAGATTAATCAATTAGAATCCATTTTTGTTTTTTTATTTTTTNNNAGTTTATAGTTAAAACTAACTATTGCTCTTATGCAAATTCTCTTGTTTTCATCTCAATCTTACCGAGGATTCTCTCTAAAGAAAAGGGATTCCAAATAGAATTCTCATTTTTTTCTATTTTTTATTAGTTATAGTTAAGTTATTAGGAATTGGTCAAAATTGACATCGATTTGCATTATCTTTTCTATTTTATTATCNNNNNNNNNNNNNNTTTGTTTATATTTATTTCAATTTTTTATGAATATGTCCATTTGCCTCTTTTTTTAGTGGTTTAGAATAGAACAAGTAATAAAACGTCTAGGAATTTTAANNNNNNNNNNNNNNNNNNNTATCAAGTCAAGATTGATTTATAGTATAGAATTGGTATATTNNNNNNNNNNNNNNNNNNNNCCTTTTATTTATTATTTATACCTTTTATTTATTNNNNNNNNNNNNATTTAATTAGAATCCAATCTCGAGGGAGGATTTTCTATCGAAAGAAAAGAGAAGACTAGGTCTAAGTAAGGTATACGAAGGAAAGCCCCTTTTACGTTGTTGACAATGTTTACAATGAAACTTAAATTAGCAAATTGGTTTTTAAAGCTTTATCTTGTGCACAACTACCGCACAATACCTGCGTAAATTACTATTAGATTTGATTGGGGTTGGGTTTTTTTAACCAGACTCGGGTCATCATACAGAATTCACTCGAATCGGATATACCAAGGGTAGTATAATTAACTCGTTGATTCGATCTTTTCTCTCTTTTC

027_20170917_05_04_ndhCret-trnV-comp AATACCAAAATCGGAATAAGACTTGATATTATCAGAAATGCCCAAAAAATATCATATTCGTAAAGCAGAAACATAGATGCACTCCTATGAACATGGAAAATATACCGGATTAGTCAATCCGACTTGAAATTCTTAAGTCGTTCATAACTGTTTAGTTAAAGTAAGAATTGCTTTTGATCGAACCATCTAGTTTCCTTTCAAGATTAATCAATTAGAATCCATTTTTGTTTTTTTATTTTTTNNNAGTTTATAGTTAAAACTAACTATTGCTCTTATGCAAATTCTCTTGTTTTCATCTCAATCTTACCGAGGATTCTCTCTAAAGAAAAGGGATTCCAAATAGAATTCTCATTTTTTTCTATTTTTTATTAGTTATAGTTAAGTTATTAGGAATTGGTCAAAATTGACATCGATTTGCATTATCTTTTCTATTTTATTATCNNNNNNNNNNNNNNTTTGTTTATATTTATTTCAATTTTTTATGAATATGTCCATTTGCCTCTTTTTTTAGTGGTTTAGAATAGAACAAGTAATAAAACGTCTAGGAATTTTAANNNNNNNNNNNNNNNNNNNTATCAAGTCAAGATTGATTTATAGTATAGAATTGGTATATTNNNNNNNNNNNNNNNNNNNNCCTTTTATTTATTATTTATACCTTTTATTTATTNNNNNNNNNNNNATTTAATTAGAATCCAATCTCGAGGGAGGATTTTCTATCGAAAGAAAAGAGAAGACTAGGTCTAAGTAAGGTATACGAAGGAAAGCCCCTTTTACGTTGTTGACAATGTTTACAATGAAACTTAAATTAGCAAATTGGTTTTTAAAGCTTTATCTTGTGCACAACTACCGCACAATACCTGCGTAAATTACTATTAGATTTGATTGGGGTTGGGTTTTTTTAACCAGACTCGGGTCATCATACAGAATTCACTCGAATCGGATATACCAAGGGTAGTATAATTAACTCGTTGATTCGATCTTTTCTCTCTTTTC

028_20170917_05_05_ndhCret-trnV-comp AATACCAAAATCGGAATAAGACTTGATATTATCAGAAATGCCCAAAAAATATCATATTCGTAAAGCAGAAACATAGATGCACTCCTATGAACATGGAAAATATACCGGATTAGTCAATCCGACTTGAAATTCTTAAGTCGTTCATAACTGTTTAGTTAAAGTAAGAATTGCTTTTGATCGAACCATCTAGTTTCCTTTCAAGATTAATCAATTAGAATCCATTTTTGTTTTTTTATTTTTTNNNAGTTTATAGTTAAAACTAACTATTGCTCTTATGCAAATTCTCTTGTTTTCATCTCAATCTTACCGAGGATTCTCTCTAAAGAAAAGGGATTCCAAATAGAATTCTCATTTTTTTCTATTTTTTATTAGTTATAGTTAAGTTATTAGGAATTGGTCAAAATTGACATCGATTTGCATTATCTTTTCTATTTTATTATCNNNNNNNNNNNNNNTTTGTTTATATTTATTTCAATTTTTTATGAATATGTCCATTTGCCTCTTTTTTTAGTGGTTTAGAATAGAACAAGTAATAAAACGTCTAGGAATTTTAANNNNNNNNNNNNNNNNNNNTATCAAGTCAAGATTGATTTATAGTATAGAATTGGTATATTNNNNNNNNNNNNNNNNNNNNCCTTTTATTTATTATTTATACCTTTTATTTATTNNNNNNNNNNNNATTTAATTAGAATCCAATCTCGAGGGAGGATTTTCTATCGAAAGAAAAGAGAAGACTAGGTCTAAGTAAGGTATACGAAGGAAAGCCCCTTTTACGTTGTTGACAATGTTTACAATGAAACTTAAATTAGCAAATTGGTTTTTAAAGCTTTATCTTGTGCACAACTACCGCACAATACCTGCGTAAATTACTATTAGATTTGATTGGGGTTGGGTTTTTTTAACCAGACTCGGGTCATCATACAGAATTCACTCGAATCGGATATACCAAGGGTAGTATAATTAACTCGTTGATTCGATCTTTTCTCTCTTTTC

029_20170917_08_02_ndhCret-trnV-comp AATACCAAAATCGGAATAAGACTTGATATTATCAGAAATGCCCAAAAAATATCATATTCGTAAAGCAGAAACATAGATGCACTCCTATGAACATGGAAAATATACCGGATTAGTCAATCCGACTTGAAATTCTTAAGTCGTTCATAACTGTTTAGTTAAAGTAAGAATTGCTTTTGATCGAACCATCTAGTTTCCTTTCAAGATTAATCAATTAGAATCCATTTTTGTTTTTTTATTTTTTTTTAGTTTTTAGTTAAAACTAACTATTGCTCTTATGCAAATTCTCTTGTTTTCATCTCAATCTTACCGAGGATTCTCTCTAAAGAAAAGGGATTCCAAATAGAATTCTCATTTTTTTCTATTTTTTATTAGTTATAGTTAAGTTATTAGGAATTGGTCAAAATTGACATCGATTTGCATTATCTTTTCTATTTTATTATCNNNNNNNNNNNNNNTTTGTTTATATTTATTTCAATTTTTTATGAATATGTCCATTTGCCTCTTTTTTTAGTGGTTTAGAATAGAACAAGTAATAAAACGTCTAGGAATTTTAANNNNNNNNNNNNNNNNNNNTATCAAGTCAAGATTGATTTATAGTATAGAATTGGTATATTNNNNNNNNNNNNNNNNNNNNCCTTTTATTTATTATTTATACCTTTTATTTATTNNNNNNNNNNNNATTTAATTAGAATCCAATCTCGAGGGAGGATTTTCTATCGAAAGAAAAGAGAAGACTAGGTCTAAGTAAGGTATACGAAGGAAAGCCCCTTTTACGTTGTTGACAATGTTTACAATGAAACTTAAATTAGCAAATTGGTTTTTAAAGCTTTATCTTGTGCACAACTACCGCACAATACCTGCGTAAATTACTATTAGATTTGATTGGGGTTGGGTTTTTTTAACCAGACTCGGGTCATCATACAGAATTCACTCGAATCGGATATACCAAGGGTAGTATAATTAACTCGTTGATTCGATCTTTTCTCTCCTTTC

030_20170917_08_03_ndhCret-trnV-comp AATACCAAAATCGGAATAAGACTTGATATTATCAGAAATGCCCAAAAAATATCATATTCGTAAAGCAGAAACATAGATGCACTCCTATGAACATGGAAAATATACCGGATTAGTCAATCCGACTTGAAATTCTTAAGTCGTTCATAACTGTTTAGTTAAAGTAAGAATTGCTTTTGATCGAACCATCTAGTTTCCTTTCAAGATTAATCAATTAGAATCCATTTTTGTTTTTTTATTTTTTTTTAGTTTTTAGTTAAAACTAACTATTGCTCTTATGCAAATTCTCTTGTTTTCATCTCAATCTTACCGAGGATTCTCTCTAAAGAAAAGGGATTCCAAATAGAATTCTCATTTTTTTCTATTTTTTATTAGTTATAGTTAAGTTATTAGGAATTGGTCAAAATTGACATCGATTTGCATTATCTTTTCTATTTTATTATCNNNNNNNNNNNNNNTTTGTTTATATTTATTTCAATTTTTTATGAATATGTCCATTTGCCTCTTTTTTTAGTGGTTTAGAATAGAACAAGTAATAAAACGTCTAGGAATTTTAANNNNNNNNNNNNNNNNNNNTATCAAGTCAAGATTGATTTATAGTATAGAATTGGTATATTNNNNNNNNNNNNNNNNNNNNCCTTTTATTTATTATTTATACCTTTTATTTATTNNNNNNNNNNNNATTTAATTAGAATCCAATCTCGAGGGAGGATTTTCTATCGAAAGAAAAGAGAAGACTAGGTCTAAGTAAGGTATACGAAGGAAAGCCCCTTTTACGTTGTTGACAATGTTTACAATGAAACTTAAATTAGCAAATTGGTTTTTAAAGCTTTATCTTGTGCACAACTACCGCACAATACCTGCGTAAATTACTATTAGATTTGATTGGGGTTGGGTTTTTTTAACCAGACTCGGGTCATCATACAGAATTCACTCGAATCGGATATACCAAGGGTAGTATAATTAACTCGTTGATTCGATCTTTTCTCTCCTTTC

031_20170917_08_04_ndhCret-trnV-comp AATACCAAAATCGGAATAAGACTTGATATTATCAGAAATGCCCAAAAAATATCATATTCGTAAAGCAGAAACATAGATGCACTCCTATGAACATGGAAAATATACCGGATTAGTCAATCCGACTTGAAATTCTTAAGTCGTTCATAACTGTTTAGTTAAAGTAAGAATTGCTTTTGATCGAACCATCTAGTTTCCTTTCAAGATTAATCAATTAGAATCCATTTTTGTTTTTTTATTTTTTNNNAGTTTATAGTTAAAACTAACTATTGCTCTTATGCAAATTCTCTTGTTTTCATCTCAATCTTACCGAGGATTCTCTCTAAAGAAAAGGGATTCCAAATAGAATTCTCATTTTTTTCTATTTTTTATTAGTTATAGTTAAGTTATTAGGAATTGGTCAAAATTGACATCGATTTGCATTATCTTTTCTATTTTATTATCNNNNNNNNNNNNNNTTTGTTTATATTTATTTCAATTTTTTATGAATATGTCCATTTGCCTCTTTTTTTAGTGGTTTAGAATAGAACAAGTAATAAAACGTCTAGGAATTTTAANNNNNNNNNNNNNNNNNNNTATCAAGTCAAGATTGATTTATAGTATAGAATTGGTATATTNNNNNNNNNNNNNNNNNNNNCCTTTTATTTATTATTTATACCTTTTATTTATTNNNNNNNNNNNNATTTAATTAGAATCCAATCTCGAGGGAGGATTTTCTATCGAAAGAAAAGAGAAGACTAGGTCTAAGTAAGGTATACGAAGGAAAGCCCCTTTTACGTTGTTGACAATGTTTACAATGAAACTTAAATTAGCAAATTGGTTTTTAAAGCTTTATCTTGTGCACAACTACCGCACAATACCTGCGTAAATTACTATTAGATTTGATTGGGGTTGGGTTTTTTTAACCAGACTCGGGTCATCATACAGAATTCACTCGAATCGGATATACCAAGGGTAGTATAATTAACTCGTTGATTCGATCTTTTTTCTCTTTTC

032_20170917_08_05_ndhCret-trnV-comp AATACCAAAATCGGAATAAGACTTGATATTATCAGAAATGCCCAAAAAATATCATATTCGTAAAGCAGAAACATAGATGCACTCCTATGAACATGGAAAATATACCGGATTAGTCAATCCGACTTGAAATTCTTAAGTCGTTCATAACTGTTTAGTTAAAGTAAGAATTGCTTTTGATCGAACCATCTAGTTTCCTTTCAAGATTAATCAATTAGAATCCATTTTTGTTTTTTTATTTTTTTTTAGTTTTTAGTTAAAACTAACTATTGCTCTTATGCAAATTCTCTTGTTTTCATCTCAATCTTACCGAGGATTCTCTCTAAAGAAAAGGGATTCCAAATAGAATTCTCATTTTTTTCTATTTTTTATTAGTTATAGTTAAGTTATTAGGAATTGGTCAAAATTGACATCGATTTGCATTATCTTTTCTATTTTATTATCNNNNNNNNNNNNNNTTTGTTTATATTTATTTCAATTTTTTATGAATATGTCCATTTGCCTCTTTTTTTAGTGGTTTAGAATAGAACAAGTAATAAAACGTCTAGGAATTTTAANNNNNNNNNNNNNNNNNNNTATCAAGTCAAGATTGATTTATAGTATAGAATTGGTATATTNNNNNNNNNNNNNNNNNNNNCCTTTTATTTATTATTTATACCTTTTATTTATTNNNNNNNNNNNNATTTAATTAGAATCCAATCTCGAGGGAGGATTTTCTATCGAAAGAAAAGAGAAGACTAGGTCTAAGTAAGGTATACGAAGGAAAGCCCCTTTTACGTTGTTGACAATGTTTACAATGAAACTTAAATTAGCAAATTGGTTTTTAAAGCTTTATCTTGTGCACAACTACCGCACAATACCTGCGTAAATTACTATTAGATTTGATTGGGGTTGGGTTTTTTTAACCAGACTCGGGTCATCATACAGAATTCACTCGAATCGGATATACCAAGGGTAGTATAATTAACTCGTTGATTCGATCTTTTCTCTCCTTTC

033_20170917_08_06_ndhCret-trnV-comp AATACCAAAATCGGAATAAGACTTGATATTATCAGAAATGCCCAAAAAATATCATATTCGTAAAGCAGAAACATAGATGCACTCCTATGAACATGGAAAATATACCGGATTAGTCAATCCGACTTGAAATTCTTAAGTCGTTCATAACTGTTTAGTTAAAGTAAGAATTGCTTTTGATCGAACCATCTAGTTTCCTTTCAAGATTAATCAATTAGAATCCATTTTTGTTTTTTTATTTTTTTTTAGTTTTTAGTTAAAACTAACTATTGCTCTTATGCAAATTCTCTTGTTTTCATCTCAATCTTACCGAGGATTCTCTCTAAAGAAAAGGGATTCCAAATAGAATTCTCATTTTTTTCTATTTTTTATTAGTTATAGTTAAGTTATTAGGAATTGGTCAAAATTGACATCGATTTGCATTATCTTTTCTATTTTATTATCNNNNNNNNNNNNNNTTTGTTTATATTTATTTCAATTTTTTATGAATATGTCCATTTGCCTCTTTTTTTAGTGGTTTAGAATAGAACAAGTAATAAAACGTCTAGGAATTTTAANNNNNNNNNNNNNNNNNNNTATCAAGTCAAGATTGATTTATAGTATAGAATTGGTATATTNNNNNNNNNNNNNNNNNNNNCCTTTTATTTATTATTTATACCTTTTATTTATTNNNNNNNNNNNNATTTAATTAGAATCCAATCTCGAGGGAGGATTTTCTATCGAAAGAAAAGAGAAGACTAGGTCTAAGTAAGGTATACGAAGGAAAGCCCCTTTTACGTTGTTGACAATGTTTACAATGAAACTTAAATTAGCAAATTGGTTTTTAAAGCTTTATCTTGTGCACAACTACCGCACAATACCTGCGTAAATTACTATTAGATTTGATTGGGGTTGGGTTTTTTTAACCAGACTCGGGTCATCATACAGAATTCACTCGAATCGGATATACCAAGGGTAGTATAATTAACTCGTTGATTCGATCTTTTCTCTCCTTTC

034_20170918_01_01_ndhCret-trnV-comp AATACCAAAATCGGAATAAGACTTGATATTATCAGAAATGCCCAAAAAATATCATATTCGTAAAGCAGAAACATAGATGCACTCCTATGAACATGGAAAATATACCGGATTAGTCAATCCGACTTGAAATTCTTAAGTCGTTCATAACTGTTTAGTTAAAGTAAGAATTGCTTTTGATCGAACCATCTAGTTTCCTTTCAAGATTAATCAATTAGAATCCATTTTTGTTTTTTTATTTTTTNNNAGTTTATAGTTAAAACTAACTATTGCTCTTATGCAAATTCTCTTGTTTTCATCTCAATCTTACCGAGGATTCTCTCTAAAGAAAAGGGATTCCAAATAGAATTCTCATTTTTTTCTATTTTTTATTAGTTATAGTTAAGTTATTAGGAATTGGTCAAAATTGACATCGATTTGCATTATCTTTTCTATTTTATTATCNNNNNNNNNNNNNNTTTGTTTATATTTATTTCAATTTTTTATGAATATGTCCATTTGCCTCTTTTTTTAGTGGTTTAGAATAGAACAAGTAATAAAACGTCTAGGAATTTTAANNNNNNNNNNNNNNNNNNNTATCAAGTCAAGATTGATTTATAGTATAGAATTGGTATATTNNNNNNNNNNNNNNNNNNNNCCTTTTATTTATTATTTATACCTTTTATTTATTNNNNNNNNNNNNATTTAATTAGAATCCAATCTCGAGGGAGGATTTTCTATCGAAAGAAAAGAGAAGACTAGGTCTAAGTAAGGTATACGAAGGAAAGCCCCTTTTACGTTGTTGACAATGTTTACAATGAAACTTAAATTAGCAAATTGGTTTTTAAAGCTTTATCTTGTGCACAACTACCGCACAATACCTGCGTAAATTACTATTAGATTTGATTGGGGTTGGGTTTTTTTAACCAGACTCGGGTCATCATACAGAATTCACTCGAATCGGATATACCAAGGGTAGTATAATTAACTCGTTGATTCGATCTTTTCTCTCTTTTC

035_20170918_01_02_ndhCret-trnV-comp AATACCAAAATCGGAATAAGACTTGATATTATCAGAAATGCCCAAAAAATATCATATTCGTAAAGCAGAAACATAGATGCACTCCTATGAACATGGAAAATATACCGGATTAGTCAATCCGACTTGAAATTCTTAAGTCGTTCATAACTGTTTAGTTAAAGTAAGAATTGCTTTTGATCGAACCATCTAGTTTCCTTTCAAGATTAATCAATTAGAATCCATTTTTGTTTTTTTATTTTTTTTTAGTTTTTAGTTAAAACTAACTATTGCTCTTATGCAAATTCTCTTGTTTTCATCTCAATCTTACCGAGGATTCTCTCTAAAGAAAAGGGATTCCAAATAGAATTCTCATTTTTTTCTATTTTTTATTAGTTATAGTTAAGTTATTAGGAATTGGTCAAAATTGACATCGATTTGCATTATCTTTTCTATTTTATTATCNNNNNNNNNNNNNNTTTGTTTATATTTATTTCAATTTTTTATGAATATGTCCATTTGCCTCTTTTTTTAGTGGTTTAGAATAGAACAAGTAATAAAACGTCTAGGAATTTTAANNNNNNNNNNNNNNNNNNNTATCAAGTCAAGATTGATTTATAGTATAGAATTGGTATATTNNNNNNNNNNNNNNNNNNNNCCTTTTATTTATTATTTATACCTTTTATTTATTNNNNNNNNNNNNATTTAATTAGAATCCAATCTCGAGGGAGGATTTTCTATCGAAAGAAAAGAGAAGACTAGGTCTAAGTAAGGTATACGAAGGAAAGCCCCTTTTACGTTGTTGACAATGTTTACAATGAAACTTAAATTAGCAAATTGGTTTTTAAAGCTTTATCTTGTGCACAACTACCGCACAATACCTGCGTAAATTACTATTAGATTTGATTGGGGTTGGGTTTTTTTAACCAGACTCGGGTCATCATACAGAATTCACTCGAATCGGATATACCAAGGGTAGTATAATTAACTCGTTGATTCGATCTTTTCTCTCCTTTC

036_20170918_01_03_ndhCret-trnV-comp AATACCAAAATCGGAATAAGACTTGATATTATCAGAAATGCCCAAAAAATATCATATTCGTAAAGCAGAAACATAGATGCACTCCTATGAACATGGAAAATATACCGGATTAGTCAATCCGACTTGAAATTCTTAAGTCGTTCATAACTGTTTAGTTAAAGTAAGAATTGCTTTTGATCGAACCATCTAGTTTCCTTTCAAGATTAATCAATTAGAATCCATTTTTGTTTTTTTATTTTTTNNNAGTTTATAGTTAAAACTAACTATTGCTCTTATGCAAATTCTCTTGTTTTCATCTCAATCTTACCGAGGATTCTCTCTAAAGAAAAGGGATTCCAAATAGAATTCTCATTTTTTTCTATTTTTTATTAGTTATAGTTAAGTTATTAGGAATTGGTCAAAATTGACATCGATTTGCATTATCTTTTCTATTTTATTATCNNNNNNNNNNNNNNTTTGTTTATATTTATTTCAATTTTTTATGAATATGTCCATTTGCCTCTTTTTTTAGTGGTTTAGAATAGAACAAGTAATAAAACGTCTAGGAATTTTAANNNNNNNNNNNNNNNNNNNTATCAAGTCAAGATTGATTTATAGTATAGAATTGGTATATTNNNNNNNNNNNNNNNNNNNNCCTTTTATTTATTATTTATACCTTTTATTTATTNNNNNNNNNNNNATTTAATTAGAATCCAATCTCGAGGGAGGATTTTCTATCGAAAGAAAAGAGAAGACTAGGTCTAAGTAAGGTATACGAAGGAAAGCCCCTTTTACGTTGTTGACAATGTTTACAATGAAACTTAAATTAGCAAATTGGTTTTTAAAGCTTTATCTTGTGCACAACTACCGCACAATACCTGCGTAAATTACTATTAGATTTGATTGGGGTTGGGTTTTTTTAACCAGACTCGGGTCATCATACAGAATTCACTCGAATCGGATATACCAAGGGTAGTATAATTAACTCGTTGATTCGATCTTTTCTCTCTTTTC

037_20170918_01_04_ndhCret-trnV-comp AATACCAAAATCGGAATAAGACTTGATATTATCAGAAATGCCCAAAAAATATCATATTCGTAAAGCAGAAACATAGATGCACTCCTATGAACATGGAAAATATACCGGATTAGTCAATCCGACTTGAAATTCTTAAGTCGTTCATAACTGTTTAGTTAAAGTAAGAATTGCTTTTGATCGAACCATCTAGTTTCCTTTCAAGATTAATCAATTAGAATCCATTTTTGTTTTTTTATTTTTTNNNAGTTTATAGTTAAAACTAACTATTGCTCTTATGCAAATTCTCTTGTTTTCATCTCAATCTTACCGAGGATTCTCTCTAAAGAAAAGGGATTCCAAATAGAATTCTCATTTTTTTCTATTTTTTATTAGTTATAGTTAAGTTATTAGGAATTGGTCAAAATTGACATCGATTTGCATTATCTTTTCTATTTTATTATCNNNNNNNNNNNNNNTTTGTTTATATTTATTTCAATTTTTTATGAATATGTCCATTTGCCTCTTTTTTTAGTGGTTTAGAATAGAACAAGTAATAAAACGTCTAGGAATTTTAANNNNNNNNNNNNNNNNNNNTATCAAGTCAAGATTGATTTATAGTATAGAATTGGTATATTNNNNNNNNNNNNNNNNNNNNCCTTTTATTTATTATTTATACCTTTTATTTATTNNNNNNNNNNNNATTTAATTAGAATCCAATCTCGAGGGAGGATTTTCTATCGAAAGAAAAGAGAAGACTAGGTCTAAGTAAGGTATACGAAGGAAAGCCCCTTTTACGTTGTTGACAATGTTTACAATGAAACTTAAATTAGCAAATTGGTTTTTAAAGCTTTATCTTGTGCACAACTACCGCACAATACCTGCGTAAATTACTATTAGATTTGATTGGGGTTGGGTTTTTTTAACCAGACTCGGGTCATCATACAGAATTCACTCGAATCGGATATACCAAGGGTAGTATAATTAACTCGTTGATTCGATCTTTTCTCTCTTTTC

038_20170918_01_05_ndhCret-trnV-comp AATACCAAAATCGGAATAAGACTTGATATTATCAGAAATGCCCAAAAAATATCATATTCGTAAAGCAGAAACATAGATGCACTCCTATGAACATGGAAAATATACCGGATTAGTCAATCCGACTTGAAATTCTTAAGTCGTTCATAACTGTTTAGTTAAAGTAAGAATTGCTTTTGATCGAACCATCTAGTTTCCTTTCAAGATTAATCAATTAGAATCCATTTTTGTTTTTTTATTTTTTTTTAGTTTTTAGTTAAAACTAACTATTGCTCTTATGCAAATTCTCTTGTTTTCATCTCAATCTTACCGAGGATTCTCTCTAAAGAAAAGGGATTCCAAATAGAATTCTCATTTTTTTCTATTTTTTATTAGTTATAGTTAAGTTATTAGGAATTGGTCAAAATTGACATCGATTTGCATTATCTTTTCTATTTTATTATCNNNNNNNNNNNNNNTTTGTTTATATTTATTTCAATTTTTTATGAATATGTCCATTTGCCTCTTTTTTTAGTGGTTTAGAATAGAACAAGTAATAAAACGTCTAGGAATTTTAANNNNNNNNNNNNNNNNNNNTATCAAGTCAAGATTGATTTATAGTATAGAATTGGTATATTNNNNNNNNNNNNNNNNNNNNCCTTTTATTTATTATTTATACCTTTTATTTATTNNNNNNNNNNNNATTTAATTAGAATCCAATCTCGAGGGAGGATTTTCTATCGAAAGAAAAGAGAAGACTAGGTCTAAGTAAGGTATACGAAGGAAAGCCCCTTTTACGTTGTTGACAATGTTTACAATGAAACTTAAATTAGCAAATTGGTTTTTAAAGCTTTATCTTGTGCACAACTACCGCACAATACCTGCGTAAATTACTATTAGATTTGATTGGGGTTGGGTTTTTTTAACCAGACTCGGGTCATCATACAGAATTCACTCGAATCGGATATACCAAGGGTAGTATAATTAACTCGTTGATTCGATCTTTTCTCTCCTTTC

039_20170918_02_02_ndhCret-trnV-comp AATACCAAAATCGGAATAAGACTTGATATTATCAGAAATGCCCAAAAAATATCATATTCGTAAAGCAGAAACATAGATGCACTCCTATGAACATGGAAAATATACCGGATTAGTCAATCCGACTTGAAATTCTTAAGTCGTTCATAACTGTTTAGTTAAAGTAAGAATTGCTTTTGATCGAACCATCTAGTTTCCTTTCAAGATTAATCAATTAGAATCCATTTTTGTTTTTTTATTTTTTNNNAGTTTATAGTTAAAACTAACTATTGCTCTTATGCAAATTCTCTTGTTTTCATCTCAATCTTACCGAGGATTCTCTCTAAAGAAAAGGGATTCCAAATAGAATTCTCATTTTTTTCTATTTTTTATTAGTTATAGTTAAGTTATTAGGAATTGGTCAAAATTGACATCGATTTGCATTATCTTTTCTATTTTATTATCNNNNNNNNNNNNNNTTTGTTTATATTTATTTCAATTTTTTATGAATATGTCCATTTGCCTCTTTTTTTAGTGGTTTAGAATAGAACAAGTAATAAAACGTCTAGGAATTTTAANNNNNNNNNNNNNNNNNNNTATCAAGTCAAGATTGATTTATAGTATAGAATTGGTATATTNNNNNNNNNNNNNNNNNNNNCCTTTTATTTATTATTTATACCTTTTATTTATTNNNNNNNNNNNNATTTAATTAGAATCCAATCTCGAGGGAGGATTTTCTATCGAAAGAAAAGAGAAGACTAGGTCTAAGTAAGGTATACGAAGGAAAGCCCCTTTTACGTTGTTGACAATGTTTACAATGAAACTTAAATTAGCAAATTGGTTTTTAAAGCTTTATCTTGTGCACAACTACCGCACAATACCTGCGTAAATTACTATTAGATTTGATTGGGGTTGGGTTTTTTTAACCAGACTCGGGTCATCATACAGAATTCACTCGAATCGGATATACCAAGGGTAGTATAATTAACTCGTTGATTCGATCTTTTCTCTCTTTTC

040_20170918_02_03_ndhCret-trnV-comp AATACCAAAATCGGAATAAGACTTGATATTATCAGAAATGCCCAAAAAATATCATATTCGTAAAGCAGAAACATAGATGCACTCCTATGAACATGGAAAATATACCGGATTAGTCAATCCGACTTGAAATTCTTAAGTCGTTCATAACTGTTTAGTTAAAGTAAGAATTGCTTTTGATCGAACCATCTAGTTTCCTTTCAAGATTAATCAATTAGAATCCATTTTTGTTTTTTTATTTTTTNNNAGTTTATAGTTAAAACTAACTATTGCTCTTATGCAAATTCTCTTGTTTTCATCTCAATCTTACCGAGGATTCTCTCTAAAGAAAAGGGATTCCAAATAGAATTCTCATTTTTTTCTATTTTTTATTAGTTATAGTTAAGTTATTAGGAATTGGTCAAAATTGACATCGATTTGCATTATCTTTTCTATTTTATTATCNNNNNNNNNNNNNNTTTGTTTATATTTATTTCAATTTTTTATGAATATGTCCATTTGCCTCTTTTTTTAGTGGTTTAGAATAGAACAAGTAATAAAACGTCTAGGAATTTTAANNNNNNNNNNNNNNNNNNNTATCAAGTCAAGATTGATTTATAGTATAGAATTGGTATATTNNNNNNNNNNNNNNNNNNNNCCTTTTATTTATTATTTATACCTTTTATTTATTNNNNNNNNNNNNATTTAATTAGAATCCAATCTCGAGGGAGGATTTTCTATCGAAAGAAAAGAGAAGACTAGGTCTAAGTAAGGTATACGAAGGAAAGCCCCTTTTACGTTGTTGACAATGTTTACAATGAAACTTAAATTAGCAAATTGGTTTTTAAAGCTTTATCTTGTGCACAACTACCGCACAATACCTGCGTAAATTACTATTAGATTTGATTGGGGTTGGGTTTTTTTAACCAGACTCGGGTCATCATACAGAATTCACTCGAATCGGATATACCAAGGGTAGTATAATTAACTCGTTGATTCGATCTTTTCTCTCTTTTC

041_20170918_02_04_ndhCret-trnV-comp AATACCAAAATCGGAATAAGACTTGATATTATCAGAAATGCCCAAAAAATATCATATTCGTAAAGCAGAAACATAGATGCACTCCTATGAACATGGAAAATATACCGGATTAGTCAATCCGACTTGAAATTCTTAAGTCGTTCATAACTGTTTAGTTAAAGTAAGAATTGCTTTTGATCGAACCATCTAGTTTCCTTTCAAGATTAATCAATTAGAATCCATTTTTGTTTTTTTATTTTTTNNNAGTTTATAGTTAAAACTAACTATTGCTCTTATGCAAATTCTCTTGTTTTCATCTCAATCTTACCGAGGATTCTCTCTAAAGAAAAGGGATTCCAAATAGAATTCTCATTTTTTTCTATTTTTTATTAGTTATAGTTAAGTTATTAGGAATTGGTCAAAATTGACATCGATTTGCATTATCTTTTCTATTTTATTATCNNNNNNNNNNNNNNTTTGTTTATATTTATTTCAATTTTTTATGAATATGTCCATTTGCCTCTTTTTTTAGTGGTTTAGAATAGAACAAGTAATAAAACGTCTAGGAATTTTAANNNNNNNNNNNNNNNNNNNTATCAAGTCAAGATTGATTTATAGTATAGAATTGGTATATTNNNNNNNNNNNNNNNNNNNNCCTTTTATTTATTATTTATACCTTTTATTTATTNNNNNNNNNNNNATTTAATTAGAATCCAATCTCGAGGGAGGATTTTCTATCGAAAGAAAAGAGAAGACTAGGTCTAAGTAAGGTATACGAAGGAAAGCCCCTTTTACGTTGTTGACAATGTTTACAATGAAACTTAAATTAGCAAATTGGTTTTTAAAGCTTTATCTTGTGCACAACTACCGCACAATACCTGCGTAAATTACTATTAGATTTGATTGGGGTTGGGTTTTTTTAACCAGACTCGGGTCATCATACAGAATTCACTCGAATCGGATATACCAAGGGTAGTATAATTAACTCGTTGATTCGATCTTTTCTCTCTTTTC

042_20170918_02_05_ndhCret-trnV-comp AATACCAAAATCGGAATAAGACTTGATATTATCAGAAATGCCCAAAAAATATCATATTCGTAAAGCAGAAACATAGATGCACTCCTATGAACATGGAAAATATACCGGATTAGTCAATCCGACTTGAAATTCTTAAGTCGTTCATAACTGTTTAGTTAAAGTAAGAATTGCTTTTGATCGAACCATCTAGTTTCCTTTCAAGATTAATCAATTAGAATCCATTTTTGTTTTTTTATTTTTTNNNAGTTTATAGTTAAAACTAACTATTGCTCTTATGCAAATTCTCTTGTTTTCATCTCAATCTTACCGAGGATTCTCTCTAAAGAAAAGGGATTCCAAATAGAATTCTCATTTTTTTCTATTTTTTATTAGTTATAGTTAAGTTATTAGGAATTGGTCAAAATTGACATCGATTTGCATTATCTTTTCTATTTTATTATCNNNNNNNNNNNNNNTTTGTTTATATTTATTTCAATTTTTTATGAATATGTCCATTTGCCTCTTTTTTTAGTGGTTTAGAATAGAACAAGTAATAAAACGTCTAGGAATTTTAANNNNNNNNNNNNNNNNNNNTATCAAGTCAAGATTGATTTATAGTATAGAATTGGTATATTNNNNNNNNNNNNNNNNNNNNCCTTTTATTTATTATTTATACCTTTTATTTATTNNNNNNNNNNNNATTTAATTAGAATCCAATCTCGAGGGAGGATTTTCTATCGAAAGAAAAGAGAAGACTAGGTCTAAGTAAGGTATACGAAGGAAAGCCCCTTTTACGTTGTTGACAATGTTTACAATGAAACTTAAATTAGCAAATTGGTTTTTAAAGCTTTATCTTGTGCACAACTACCGCACAATACCTGCGTAAATTACTATTAGATTTGATTGGGGTTGGGTTTTTTTAACCAGACTCGGGTCATCATACAGAATTCACTCGAATCGGATATACCAAGGGTAGTATAATTAACTCGTTGATTCGATCTTTTCTCTCTTTTC

043_20170918_02_06_ndhCret-trnV-comp AATACCAAAATCGGAATAAGACTTGATATTATCAGAAATGCCCAAAAAATATCATATTCGTAAAGCAGAAACATAGATGCACTCCTATGAACATGGAAAATATACCGGATTAGTCAATCCGACTTGAAATTCTTAAGTCGTTCATAACTGTTTAGTTAAAGTAAGAATTGCTTTTGATCGAACCATCTAGTTTCCTTTCAAGATTAATCAATTAGAATCCATTTTTGTTTTTTTATTTTTTNNNAGTTTATAGTTAAAACTAACTATTGCTCTTATGCAAATTCTCTTGTTTTCATCTCAATCTTACCGAGGATTCTCTCTAAAGAAAAGGGATTCCAAATAGAATTCTCATTTTTTTCTATTTTTTATTAGTTATAGTTAAGTTATTAGGAATTGGTCAAAATTGACATCGATTTGCATTATCTTTTCTATTTTATTATCNNNNNNNNNNNNNNTTTGTTTATATTTATTTCAATTTTTTATGAATATGTCCATTTGCCTCTTTTTTTAGTGGTTTAGAATAGAACAAGTAATAAAACGTCTAGGAATTTTAANNNNNNNNNNNNNNNNNNNTATCAAGTCAAGATTGATTTATAGTATAGAATTGGTATATTNNNNNNNNNNNNNNNNNNNNCCTTTTATTTATTATTTATACCTTTTATTTATTNNNNNNNNNNNNATTTAATTAGAATCCAATCTCGAGGGAGGATTTTCTATCGAAAGAAAAGAGAAGACTAGGTCTAAGTAAGGTATACGAAGGAAAGCCCCTTTTACGTTGTTGACAATGTTTACAATGAAACTTAAATTAGCAAATTGGTTTTTAAAGCTTTATCTTGTGCACAACTACCGCACAATACCTGCGTAAATTACTATTAGATTTGATTGGGGTTGGGTTTTTTTAACCAGACTCGGGTCATCATACAGAATTCACTCGAATCGGATATACCAAGGGTAGTATAATTAACTCGTTGATTCGATCTTTTCTCTCTTTTC

044_20170920_01_01_ndhCret-trnV-comp AATACCAAAATCGGAATAAGACTTGATATTATCAGAAATGCCCAAAAAATATCATATTCGTAAAGCAGAAACATAGATGCACTCCTATGAACATGGAAAATATACCGGATTAGTCAATCCGACTTGAAATTCTTAAGTCGTTCATAACTGTTTAGTTAAAGTAAGAATTGCTTTTGATCGAACCATCTAGTTTCCTTTCAAGATTAATCAATTAGAATCCATTTTTGTTTTTTTATTTTTTNNNAGTTTATAGTTAAAACTAACTATTGCTCTTATGCAAATTCTCTTGTTTTCATCTCAATCTTACCGAGGATTCTCTCTAAAGAAAAGGGATTCCAAATAGAATTCTCATTTTTTTCTATTTTTTATTAGTTATAGTTAAGTTATTAGGAATTGGTCAAAATTGACATCGATTTGCATTATCTTTTCTATTTTATTATCNNNNNNNNNNNNNNTTTGTTTATATTTATTTCAATTTTTTATGAATATGTCCATTTGCCTCTTTTTTTAGTGGTTTAGAATAGAACAAGTAATAAAACGTCTAGGAATTTTAANNNNNNNNNNNNNNNNNNNTATCAAGTCAAGATTGATTTATAGTATAGAATTGGTATATTNNNNNNNNNNNNNNNNNNNNCCTTTTATTTATTATTTATACCTTTTATTTATTNNNNNNNNNNNNATTTAATTAGAATCCAATCTCGAGGGAGGATTTTCTATCGAAAGAAAAGAGAAGACTAGGTCTAAGTAAGGTATACGAAGGAAAGCCCCTTTTACGTTGTTGACAATGTTTACAATGAAACTTAAATTAGCAAATTGGTTTTTAAAGCTTTATCTTGTGCACAACTACCGCACAATACCTGCGTAAATTACTATTAGATTTGATTGGGGTTGGGTTTTTTTAACCAGACTCGGGTCATCATACAGAATTCACTCGAATCGGATATACCAAGGGTAGTATAATTAACTCGTTGATTCGATCTTTTCTCTCTTTTC

045_20170920_01_02_ndhCret-trnV-comp AATACCAAAATCGGAATAAGACTTGATATTATCAGAAATGCCCAAAAAATATCATATTCGTAAAGCAGAAACATAGATGCACTCCTATGAACATGGAAAATATACCGGATTAGTCAATCCGACTTGAAATTCTTAAGTCGTTCATAACTGTTTAGTTAAAGTAAGAATTGCTTTTGATCGAACCATCTAGTTTCCTTTCAAGATTAATCAATTAGAATCCATTTTTGTTTTTTTATTTTTTNNNAGTTTATAGTTAAAACTAACTATTGCTCTTATGCAAATTCTCTTGTTTTCATCTCAATCTTACCGAGGATTCTCTCTAAAGAAAAGGGATTCCAAATAGAATTCTCATTTTTTTCTATTTTTTATTAGTTATAGTTAAGTTATTAGGAATTGGTCAAAATTGACATCGATTTGCATTATCTTTTCTATTTTATTATCNNNNNNNNNNNNNNTTTGTTTATATTTATTTCAATTTTTTATGAATATGTCCATTTGCCTCTTTTTTTAGTGGTTTAGAATAGAACAAGTAATAAAACGTCTAGGAATTTTAANNNNNNNNNNNNNNNNNNNTATCAAGTCAAGATTGATTTATAGTATAGAATTGGTATATTNNNNNNNNNNNNNNNNNNNNCCTTTTATTTATTATTTATACCTTTTATTTATTNNNNNNNNNNNNATTTAATTAGAATCCAATCTCGAGGGAGGATTTTCTATCGAAAGAAAAGAGAAGACTAGGTCTAAGTAAGGTATACGAAGGAAAGCCCCTTTTACGTTGTTGACAATGTTTACAATGAAACTTAAATTAGCAAATTGGTTTTTAAAGCTTTATCTTGTGCACAACTACCGCACAATACCTGCGTAAATTACTATTAGATTTGATTGGGGTTGGGTTTTTTTAACCAGACTCGGGTCATCATACAGAATTCACTCGAATCGGATATACCAAGGGTAGTATAATTAACTCGTTGATTCGATCTTTTCTCTCTTTTC

046_20170920_01_03_ndhCret-trnV-comp AATACCAAAATCGGAATAAGACTTGATATTATCAGAAATGCCCAAAAAATATCATATTCGTAAAGCAGAAACATAGATGCACTCCTATGAACATGGAAAATATACCGGATTAGTCAATCCGACTTGAAATTCTTAAGTCGTTCATAACTGTTTAGTTAAAGTAAGAATTGCTTTTGATCGAACCATCTAGTTTCCTTTCAAGATTAATCAATTAGAATCCATTTTTGTTTTTTTATTTTTTNNNAGTTTATAGTTAAAACTAACTATTGCTCTTATGCAAATTCTCTTGTTTTCATCTCAATCTTACCGAGGATTCTCTCTAAAGAAAAGGGATTCCAAATAGAATTCTCATTTTTTTCTATTTTTTATTAGTTATAGTTAAGTTATTAGGAATTGGTCAAAATTGACATCGATTTGCATTATCTTTTCTATTTTATTATCNNNNNNNNNNNNNNTTTGTTTATATTTATTTCAATTTTTTATGAATATGTCCATTTGCCTCTTTTTTTAGTGGTTTAGAATAGAACAAGTAATAAAACGTCTAGGAATTTTAANNNNNNNNNNNNNNNNNNNTATCAAGTCAAGATTGATTTATAGTATAGAATTGGTATATTNNNNNNNNNNNNNNNNNNNNCCTTTTATTTATTATTTATACCTTTTATTTATTNNNNNNNNNNNNATTTAATTAGAATCCAATCTCGAGGGAGGATTTTCTATCGAAAGAAAAGAGAAGACTAGGTCTAAGTAAGGTATACGAAGGAAAGCCCCTTTTACGTTGTTGACAATGTTTACAATGAAACTTAAATTAGCAAATTGGTTTTTAAAGCTTTATCTTGTGCACAACTACCGCACAATACCTGCGTAAATTACTATTAGATTTGATTGGGGTTGGGTTTTTTTAACCAGACTCGGGTCATCATACAGAATTCACTCGAATCGGATATACCAAGGGTAGTATAATTAACTCGTTGATTCGATCTTTTCTCTCTTTTC

047_20180610_01_01_ndhCret-trnV-comp AATACCAAAATCGGAATAAGACTTGATATTATCAGAAATGCCCAAAAAATATCATATTCGTAAAGCAGAAACATAGATGCACTCCTATGAACATGGAAAATATACCGGATTAGTCAATCCGACTTGAAATTCTTAAGTCGTTCATAACTGTTTAGTTAAAGTAAGAATTGCTTTTGATCGAACCATCTAGTTTCCTTTCAAGATTAATCAATTAGAATCCATTTTTGTTTTTTTATTTTTTNNNAGTTTATAGTTAAAACTAACTATTGCTCTTATGCAAATTCTCTTGTTTTCATCTCAATCTTACCGAGGATTCTCTCTAAAGAAAAGGGATTCCAAATAGAATTCTCATTTTTTTCTATTTTTTATTAGTTATAGTTAAGTTATTAGGAATTGGTCAAAATTGACATCGATTTGCATTATCTTTTCTATTTTATTATCNNNNNNNNNNNNNNTTTGTTTATATTTATTTCAATTTTTTATGAATATGTCCATTTGCCTCTTTTTTTAGTGGTTTAGAATAGAACAAGTAATAAAACGTCTAGGAATTTTAANNNNNNNNNNNNNNNNNNNTATCAAGTCAAGATTGATTTATAGTATAGAATTGGTATATTNNNNNNNNNNNNNNNNNNNNCCTTTTATTTATTATTTATACCTTTTATTTATTNNNNNNNNNNNNATTTAATTAGAATCCAATCTCGAGGGAGGATTTTCTATCGAAAGAAAAGAGAAGACTAGGTCTAAGTAAGGTATACGAAGGAAAGCCCCTTTTACGTTGTTGACAATGTTTACAATGAAACTTAAATTAGCAAATTGGTTTTTAAAGCTTTATCTTGTGCACAACTACCGCACAATACCTGCGTAAATTACTATTAGATTTGATTGGGGTTGGGTTTTTTTAACCAGACTCGGGTCATCATACAGAATTCACTCGAATCGGATATACCAAGGGTAGTATAATTAACTCGTTGATTCGATCTTTTCTCTCTTTTC

048_20180610_01_02_ndhCret-trnV-comp AATACCAAAATCGGAATAAGACTTGATATTATCAGAAATGCCCAAAAAATATCATATTCGTAAAGCAGAAACATAGATGCACTCCTATGAACATGGAAAATATACCGGATTAGTCAATCCGACTTGAAATTCTTAAGTCGTTCATAACTGTTTAGTTAAAGTAAGAATTGCTTTTGATCGAACCATCTAGTTTCCTTTCAAGATTAATCAATTAGAATCCATTTTTGTTTTTTTATTTTTTNNNAGTTTATAGTTAAAACTAACTATTGCTCTTATGCAAATTCTCTTGTTTTCATCTCAATCTTACCGAGGATTCTCTCTAAAGAAAAGGGATTCCAAATAGAATTCTCATTTTTTTCTATTTTTTATTAGTTATAGTTAAGTTATTAGGAATTGGTCAAAATTGACATCGATTTGCATTATCTTTTCTATTTTATTATCNNNNNNNNNNNNNNTTTGTTTATATTTATTTCAATTTTTTATGAATATGTCCATTTGCCTCTTTTTTTAGTGGTTTAGAATAGAACAAGTAATAAAACGTCTAGGAATTTTAANNNNNNNNNNNNNNNNNNNTATCAAGTCAAGATTGATTTATAGTATAGAATTGGTATATTNNNNNNNNNNNNNNNNNNNNCCTTTTATTTATTATTTATACCTTTTATTTATTNNNNNNNNNNNNATTTAATTAGAATCCAATCTCGAGGGAGGATTTTCTATCGAAAGAAAAGAGAAGACTAGGTCTAAGTAAGGTATACGAAGGAAAGCCCCTTTTACGTTGTTGACAATGTTTACAATGAAACTTAAATTAGCAAATTGGTTTTTAAAGCTTTATCTTGTGCACAACTACCGCACAATACCTGCGTAAATTACTATTAGATTTGATTGGGGTTGGGTTTTTTTAACCAGACTCGGGTCATCATACAGAATTCACTCGAATCGGATATACCAAGGGTAGTATAATTAACTCGTTGATTCGATCTTTTCTCTCTTTTC

049_20180610_01_03_ndhCret-trnV-comp AATACCAAAATCGGAATAAGACTTGATATTATCAGAAATGCCCAAAAAATATCATATTCGTAAAGCAGAAACATAGATGCACTCCTATGAACATGGAAAATATACCGGATTAGTCAATCCGACTTGAAATTCTTAAGTCGTTCATAACTGTTTAGTTAAAGTAAGAATTGCTTTTGATCGAACCATCTAGTTTCCTTTCAAGATTAATCAATTAGAATCCATTTTTGTTTTTTTATTTTTTNNNAGTTTATAGTTAAAACTAACTATTGCTCTTATGCAAATTCTCTTGTTTTCATCTCAATCTTACCGAGGATTCTCTCTAAAGAAAAGGGATTCCAAATAGAATTCTCATTTTTTTCTATTTTTTATTAGTTATAGTTAAGTTATTAGGAATTGGTCAAAATTGACATCGATTTGCATTATCTTTTCTATTTTATTATCNNNNNNNNNNNNNNTTTGTTTATATTTATTTCAATTTTTTATGAATATGTCCATTTGCCTCTTTTTTTAGTGGTTTAGAATAGAACAAGTAATAAAACGTCTAGGAATTTTAANNNNNNNNNNNNNNNNNNNTATCAAGTCAAGATTGATTTATAGTATAGAATTGGTATATTNNNNNNNNNNNNNNNNNNNNCCTTTTATTTATTATTTATACCTTTTATTTATTNNNNNNNNNNNNATTTAATTAGAATCCAATCTCGAGGGAGGATTTTCTATCGAAAGAAAAGAGAAGACTAGGTCTAAGTAAGGTATACGAAGGAAAGCCCCTTTTACGTTGTTGACAATGTTTACAATGAAACTTAAATTAGCAAATTGGTTTTTAAAGCTTTATCTTGTGCACAACTACCGCACAATACCTGCGTAAATTACTATTAGATTTGATTGGGGTTGGGTTTTTTTAACCAGACTCGGGTCATCATACAGAATTCACTCGAATCGGAGATACCAAGGGTAGTATAATTAACTCGTTGATTCGATCTTTTCTCTCTTTTC

050_20180610_01_04_ndhCret-trnV-comp AATACCAAAATCGGAATAAGACTTGATATTATCAGAAATGCCCAAAAAATATCATATTCGTAAAGCAGAAACATAGATGCACTCCTATGAACATGGAAAATATACCGGATTAGTCAATCCGACTTGAAATTCTTAAGTCGTTCATAACTGTTTAGTTAAAGTAAGAATTGCTTTTGATCGAACCATCTAGTTTCCTTTCAAGATTAATCAATTAGAATCCATTTTTGTTTTTTTATTTTTTTTTAGTTTATAGTTAAAACTAACTATTGCTCTTATGCAAATTCTCTTGTTTTCATCTCAATCTTACCGAGGATTCTCTCTAAAGAAAAGGGATTCCAAATAGAATTCTCATTTTTTTCTATTTTTTATTAGTTATAGTTAAGTTATTAGGAATTGGTCAAAATTGACATCGATTTGCATTATCTTTTCTATTTTATTATCNNNNNNNNNNNNNNTTTGTTTATATTTATTTCAATTTTTTATGAATATGTCCATTTGCCTCTTTTTTTAGTGGTTTAGAATAGAACAAGTAATAAAACGTCTAGGAATTTTAATATCAAGTCAAGATTGATTTATCAAGTCAAGATTGATTTATAGTATAGAATTGGTATATTCCTTTTATTTATTATTTATACCTTTTATTTATTATTTATACCTTTTATTTATTNNNNNNNNNNNNATTTAATTAGAATCCAATCTCGAGGGAGTATTTTCTATCGAAAGAAAAGAGAAGACTAGGTCTAAGTAAGGTATACGAAGGAAAGCCCCTTTTACGTTGTTGACAATGTTTACAATGAAACTTAAATTAGCAAATTGGTTTTTAAAGCTTTATCTTGTGCACAACTACCGCACAATACCTGCGTAAATTACTATTAGATTTGATTGGGGTTGGGTTTTTTTAACCAGACTCGGGTCATCATACAGAATTCACTCGAATCGGATATACCAAGGGTAGTATAATTAACTCGTTGATTCGATCTTCTCTCTCCTCTC

051_20180610_01_06_ndhCret-trnV-comp AATACCAAAATCGGAATAAGACTTGATATTATCAGAAATGCCCAAAAAATATCATATTCGTAAAGCAGAAACATAGATGCACTCCTATGAACATGGAAAATATACCGGATTAGTCAATCCGACTTGAAATTCTTAAGTCGTTCATAACTGTTTAGTTAAAGTAAGAATTGCTTTTGATCGAACCATCTAGTTTCCTTTCAAGATTAATCAATTAGAATCCATTTTTGTTTTTTTATTTTTTNNNAGTTTATAGTTAAAACTAACTATTGCTCTTATGCAAATTCTCTTGTTTTCATCTCAATCTTACCGAGGATTCTCTCTAAAGAAAAGGGATTCCAAATAGAATTCTCATTTTTTTCTATTTTTTATTAGTTATAGTTAAGTTATTAGGAATTGGTCAAAATTGACATCGATTTGCATTATCTTTTCTATTTTATTATCNNNNNNNNNNNNNNTTTGTTTATATTTATTTCAATTTTTTATGAATATGTCCATTTGCCTCTTTTTTTAGTGGTTTAGAATAGAACAAGTAATAAAACGTCTAGGAATTTTAANNNNNNNNNNNNNNNNNNNTATCAAGTCAAGATTGATTTATAGTATAGAATTGGTATATTNNNNNNNNNNNNNNNNNNNNCCTTTTATTTATTATTTATACCTTTTATTTATTNNNNNNNNNNNNATTTAATTAGAATCCAATCTCGAGGGAGGATTTTCTATCGAAAGAAAAGAGAAGACTAGGTCTAAGTAAGGTATACGAAGGAAAGCCCCTTTTACGTTGTTGACAATGTTTACAATGAAACTTAAATTAGCAAATTGGTTTTTAAAGCTTTATCTTGTGCACAACTACCGCACAATACCTGCGTAAATTACTATTAGATTTGATTGGGGTTGGGTTTTTTTAACCAGACTCGGGTCATCATACAGAATTCACTCGAATCGGATATACCAAGGGTAGTATAATTAACTCGTTGATTCGATCTTTTCTCTCTTTTC

052_20180622_02_01_ndhCret-trnV-comp AATACCAAAATCGGAATAAGACTTGATATTATCAGAAATGCCCAAAAAATATCATATTCGTAAAGCAGAAACATAGATGCACTCCTATGAACATGGAAAATATACCGGATTAGTCAATCCGACTTGAAATTCTTAAGTCGTTCATAACTGTTTAGTTAAAGTAAGAATTGCTTTTGATCGAACCATCTAGTTTCCTTTCAAGATTAATCAATTAGAATCCATTTTTGTTTTTTTATTTTTTNNNAGTTTATAGTTAAAACTAACTATTGCTCTTATGCAAATTCTCTTGTTTTCATCTCAATCTTACCGAGGATTCTCTCTAAAGAAAAGGGATTCCAAATAGAATTCTCATTTTTTTCTATTTTTTATTAGTTATAGTTAAGTTATTAGGAATTGGTCAAAATTGACATCGATTTGCATTATCTTTTCTATTTTATTATCNNNNNNNNNNNNNNTTTGTTTATATTTATTTCAATTTTTTATGAATATGTCCATTTGCCTCTTTTTTTAGTGGTTTAGAATAGAACAAGTAATAAAACGTCTAGGAATTTTAANNNNNNNNNNNNNNNNNNNTATCAAGTCAAGATTGATTTATAGTATAGAATTGGTATATTNNNNNNNNNNNNNNNNNNNNCCTTTTATTTATTATTTATACCTTTTATTTATTNNNNNNNNNNNNATTTAATTAGAATCCAATCTCGAGGGAGGATTTTCTATCGAAAGAAAAGAGAAGACTAGGTCTAAGTAAGGTATACGAAGGAAAGCCCCTTTTACGTTGTTGACAATGTTTACAATGAAACTTAAATTAGCAAATTGGTTTTTAAAGCTTTATCTTGTGCACAACTACCGCACAATACCTGCGTAAATTACTATTAGATTTGATTGGGGTTGGGTTTTTTTAACCAGACTCGGGTCATCATACAGAATTCACTCGAATCGGATATACCAAGGGTAGTATAATTAACTCGTTGATTCGATCTTTTCTCTCTTTTC

053_20180622_02_02_ndhCret-trnV-comp AATACCAAAATCGGAATAAGACTTGATATTATCAGAAATGCCCAAAAAATATCATATTCGTAAAGCAGAAACATAGATGCACTCCTATGAACATGGAAAATATACCGGATTAGTCAATCCGACTTGAAATTCTTAAGTCGTTCATAACTGTTTAGTTAAAGTAAGAATTGCTTTTGATCGAACCATCTAGTTTCCTTTCAAGATTAATCAATTAGAATCCATTTTTGTTTTTTTATTTTTTTTTAGTTTTTAGTTAAAACTAACTATTGCTCTTATGCAAATTCTCTTGTTTTCATCTCAATCTTACCGAGGATTCTCTCTAAAGAAAAGGGATTCCAAATAGAATTCTCATTTTTTTCTATTTTTTATTAGTTATAGTTAAGTTATTAGGAATTGGTCAAAATTGACATCGATTTGCATTATCTTTTCTATTTTATTATCNNNNNNNNNNNNNNTTTGTTTATATTTATTTCAATTTTTTATGAATATGTCCATTTGCCTCTTTTTTTAGTGGTTTAGAATAGAACAAGTAATAAAACGTCTAGGAATTTTAANNNNNNNNNNNNNNNNNNNTATCAAGTCAAGATTGATTTATAGTATAGAATTGGTATATTNNNNNNNNNNNNNNNNNNNNCCTTTTATTTATTATTTATACCTTTTATTTATTNNNNNNNNNNNNATTTAATTAAAATCCAATCTCGAGGGAGGATTTTCTATCGAAAGAAAAGAGAAGACTAGGTCTAAGTAAGGTATACGAAGGAAAGCCCCTTTTACGTTGTTGACAATGTTTACAATGAAACTTAAATTAGCAAATTGGTTTTTAAAGCTTTATCTTGTGCACAACTACCGCACAATACCTGCGTAAATTACTATTAGATTTGATTGGGGTTGGGTTTTTTTAACCAGACTCGGGTCATCATACAGAATTCACTCGAATCGGATATACCAAGGGTAGTATAATTAACTCGTTGATTCGATCTTTTCTCTCCTTTC

054_20180622_02_03_ndhCret-trnV-comp AATACCAAAATCGGAATAAGACTTGATATTATCAGAAATGCCCAAAAAATATCATATTCGTAAAGCAGAAACATAGATGCACTCCTATGAACATGGAAAATATACCGGATTAGTCAATCCGACTTGAAATTCTTAAGTCGTTCATAACTGTTTAGTTAAAGTAAGAATTGCTTTTGATCGAACCATCTAGTTTCCTTTCAAGATTAATCAATTAGAATCCATTTTTGTTTTTTTATTTTTTNNNAGTTTATAGTTAAAACTAACTATTGCTCTTATGCAAATTCTCTTGTTTTCATCTCAATCTTACCGAGGATTCTCTCTAAAGAAAAGGGATTCCAAATAGAATTCTCATTTTTTTCTATTTTTTATTAGTTATAGTTAAGTTATTAGGAATTGGTCAAAATTGACATCGATTTGCATTATCTTTTCTATTTTATTATCNNNNNNNNNNNNNNTTTGTTTATATTTATTTCAATTTTTTATGAATATGTCCATTTGCCTCTTTTTTTAGTGGTTTAGAATAGAACAAGTAATAAAACGTCTAGGAATTTTAANNNNNNNNNNNNNNNNNNNTATCAAGTCAAGATTGATTTATAGTATAGAATTGGTATATTNNNNNNNNNNNNNNNNNNNNCCTTTTATTTATTATTTATACCTTTTATTTATTNNNNNNNNNNNNATTTAATTAGAATCCAATCTCGAGGGAGGATTTTCTATCGAAAGAAAAGAGAAGACTAGGTCTAAGTAAGGTATACGAAGGAAAGCCCCTTTTACGTTGTTGACAATGTTTACAATGAAACTTAAATTAGCAAATTGGTTTTTAAAGCTTTATCTTGTGCACAACTACCGCACAATACCTGCGTAAATTACTATTAGATTTGATTGGGGTTGGGTTTTTTTAACCAGACTCGGGTCATCATACAGAATTCACTCGAATCGGATATACCAAGGGTAGTATAATTAACTCGTTGATTCGATCTTTTCTCTCTTTTC

055_20180622_02_04_ndhCret-trnV-comp AATACCAAAATCGGAATAAGACTTGATATTATCAGAAATGCCCAAAAAATATCATATTCGTAAAGCAGAAACATAGATGCACTCCTATGAACATGGAAAATATACCGGATTAGTCAATCCGACTTGAAATTCTTAAGTCGTTCATAACTGTTTAGTTAAAGTAAGAATTGCTTTTGATCGAACCATCTAGTTTCCTTTCAAGATTAATCAATTAGAATCCATTTTTGTTTTTTTATTTTTTNNNAGTTTATAGTTAAAACTAACTATTGCTCTTATGCAAATTCTCTTGTTTTCATCTCAATCTTACCGAGGATTCTCTCTAAAGAAAAGGGATTCCAAATAGAATTCTCATTTTTTTCTATTTTTTATTAGTTATAGTTAAGTTATTAGGAATTGGTCAAAATTGACATCGATTTGCATTATCTTTTCTATTTTATTATCNNNNNNNNNNNNNNTTTGTTTATATTTATTTCAATTTTTTATGAATATGTCCATTTGCCTCTTTTTTTAGTGGTTTAGAATAGAACAAGTAATAAAACGTCTAGGAATTTTAANNNNNNNNNNNNNNNNNNNTATCAAGTCAAGATTGATTTATAGTATAGAATTGGTATATTNNNNNNNNNNNNNNNNNNNNCCTTTTATTTATTATTTATACCTTTTATTTATTNNNNNNNNNNNNATTTAATTAGAATCCAATCTCGAGGGAGGATTTTCTATCGAAAGAAAAGAGAAGACTAGGTCTAAGTAAGGTATACGAAGGAAAGCCCCTTTTACGTTGTTGACAATGTTTACAATGAAACTTAAATTAGCAAATTGGTTTTTAAAGCTTTATCTTGTGCACAACTACCGCACAATACCTGCGTAAATTACTATTAGATTTGATTGGGGTTGGGTTTTTTTAACCAGACTCGGGTCATCATACAGAATTCACTCGAATCGGATATACCAAGGGTAGTATAATTAACTCGTTGATTCGATCTTTTCTCTCTTTTC

056_20180622_02_05_ndhCret-trnV-comp AATACCAAAATCGGAATAAGACTTGATATTATCAGAAATGCCCAAAAAATATCATATTCGTAAAGCAGAAACATAGATGCACTCCTATGAACATGGAAAATATACCGGATTAGTCAATCCGACTTGAAATTCTTAAGTCGTTCATAACTGTTTAGTTAAAGTAAGAATTGCTTTTGATCGAACCATCTAGTTTCCTTTCAAGATTAATCAATTAGAATCCATTTTTGTTTTTTTATTTTTTTTTAGTTTTTAGTTAAAACTAACTATTGCTCTTATGCAAATTCTCTTGTTTTCATCTCAATCTTACCGAGGATTCTCTCTAAAGAAAAGGGATTCCAAATAGAATTCTCATTTTTTTCTATTTTTTATTAGTTATAGTTAAGTTATTAGGAATTGGTCAAAATTGACATCGATTTGCATTATCTTTTCTATTTTATTATCNNNNNNNNNNNNNNTTTGTTTATATTTATTTCAATTTTTTATGAATATGTCCATTTGCCTCTTTTTTTAGTGGTTTAGAATAGAACAAGTAATAAAACGTCTAGGAATTTTAANNNNNNNNNNNNNNNNNNNTATCAAGTCAAGATTGATTTATAGTATAGAATTGGTATATTNNNNNNNNNNNNNNNNNNNNCCTTTTATTTATTATTTATACCTTTTATTTATTNNNNNNNNNNNNATTTAATTAGAATCCAATCTCGAGGGAGGATTTTCTATCGAAAGAAAAGAGAAGACTAGGTCTAAGTAAGGTATACGAAGGAAAGCCCCTTTTACGTTGTTGACAATGTTTACAATGAAACTTAAATTAGCAAATTGGTTTTTAAAGCTTTATCTTGTGCACAACTACCGCACAATACCTGCGTAAATTACTATTAGATTTGATTGGGGTTGGGTTTTTTTAACCAGACTCGGGTCATCATACAGAATTCACTCGAATCGGATATACCAAGGGTAGTATAATTAACTCGTTGATTCGATCTTTTCTCTCCTTTC

057_20180623_04_01_ndhCret-trnV-comp AATACCAAAATCGGAATAAGACTTGATATTATCAGAAATGCCCAAAAAATATCATATTCGTAAAGCAGAAACATAGATGCACTCCTATGAACATGGAAAATATACCGGATTAGTCAATCCGACTTGAAATTCTTAAGTCGTTCATAACTGTTTAGTTAAAGTAAGAATTGCTTTTGATCGAACCATCTAGTTTCCTTTCAAGATTAATCAATTAGAATCCATTTTTGTTTTTTTATTTTTTNNNAGTTTATAGTTAAAACTAACTATTGCTCTTATGCAAATTCTCTTGTTTTCATCTCAATCTTACCGAGGATTCTCTCTAAAGAAAAGGGATTCCAAATAGAATTCTCATTTTTTTCTATTTTTTATTAGTTATAGTTAAGTTATTAGGAATTGGTCAAAATTGACATCGATTTGCATTATCTTTTCTATTTTATTATCNNNNNNNNNNNNNNTTTGTTTATATTTATTTCAATTTTTTATGAATATGTCCATTTGCCTCTTTTTTTAGTGGTTTAGAATAGAACAAGTAATAAAACGTCTAGGAATTTTAANNNNNNNNNNNNNNNNNNNTATCAAGTCAAGATTGATTTATAGTATAGAATTGGTATATTNNNNNNNNNNNNNNNNNNNNCCTTTTATTTATTATTTATACCTTTTATTTATTNNNNNNNNNNNNATTTAATTAGAATCCAATCTCGAGGGAGGATTTTCTATCGAAAGAAAAGAGAAGACTAGGTCTAAGTAAGGTATACGAAGGAAAGCCCCTTTTACGTTGTTGACAATGTTTACAATGAAACTTAAATTAGCAAATTGGTTTTTAAAGCTTTATCTTGTGCACAACTACCGCACAATACCTGCGTAAATTACTATTAGATTTGATTGGGGTTGGGTTTTTTTAACCAGACTCGGGTCATCATACAGAATTCACTCGAATCGGATATACCAAGGGTAGTATAATTAACTCGTTGATTCGATCTTTTCTCTCTTTTC

058_20180623_04_02_ndhCret-trnV-comp AATACCAAAATCGGAATAAGACTTGATATTATCAGAAATGCCCAAAAAATATCATATTCGTAAAGCAGAAACATAGATGCACTCCTATGAACATGGAAAATATACCGGATTAGTCAATCCGACTTGAAATTCTTAAGTCGTTCATAACTGTTTAGTTAAAGTAAGAATTGCTTTTGATCGAACCATCTAGTTTCCTTTCAAGATTAATCAATTAGAATCCATTTTTGTTTTTTTATTTTTTTTTAGTTTTTAGTTAAAACTAACTATTGCTCTTATGCAAATTCTCTTGTTTTCATCTCAATCTTACCGAGGATTCTCTCTAAAGAAAAGGGATTCCAAATAGAATTCTCATTTTTTTCTATTTTTTATTAGTTATAGTTAAGTTATTAGGAATTGGTCAAAATTGACATCGATTTGCATTATCTTTTCTATTTTATTATCNNNNNNNNNNNNNNTTTGTTTATATTTATTTCAATTTTTTATGAATATGTCCATTTGCCTCTTTTTTTAGTGGTTTAGAATAGAACAAGTAATAAAACGTCTAGGAATTTTAANNNNNNNNNNNNNNNNNNNTATCAAGTCAAGATTGATTTATAGTATAGAATTGGTATATTNNNNNNNNNNNNNNNNNNNNCCTTTTATTTATTATTTATACCTTTTATTTATTNNNNNNNNNNNNATTTAATTAGAATCCAATCTCGAGGGAGGATTTTCTATCGAAAGAAAAGAGAAGACTAGGTCTAAGTAAGGTATACGAAGGAAAGCCCCTTTTACGTTGTTGACAATGTTTACAATGAAACTTAAATTAGCAAATTGGTTTTTAAAGCTTTATCTTGTGCACAACTACCGCACAATACCTGCGTAAATTACTATTAGATTTGATTGGGGTTGGGTTTTTTTAACCAGACTCGGGTCATCATACAGAATTCACTCGAATCGGATATACCAAGGGTAGTATAATTAACTCGTTGATTCGATCTTTTCTCTCCTTTC

059_20180623_04_03_ndhCret-trnV-comp AATACCAAAATCGGAATAAGACTTGATATTATCAGAAATGCCCAAAAAATATCATATTCGTAAAGCAGAAACATAGATGCACTCCTATGAACATGGAAAATATACCGGATTAGTCAATCCGACTTGAAATTCTTAAGTCGTTCATAACTGTTTAGTTAAAGTAAGAATTGCTTTTGATCGAACCATCTAGTTTCCTTTCAAGATTAATCAATTAGAATCCATTTTTGTTTTTTTATTTTTTNNNAGTTTATAGTTAAAACTAACTATTGCTCTTATGCAAATTCTCTTGTTTTCATCTCAATCTTACCGAGGATTCTCTCTAAAGAAAAGGGATTCCAAATAGAATTCTCATTTTTTTCTATTTTTTATTAGTTATAGTTAAGTTATTAGGAATTGGTCAAAATTGACATCGATTTGCATTATCTTTTCTATTTTATTATCNNNNNNNNNNNNNNTTTGTTTATATTTATTTCAATTTTTTATGAATATGTCCATTTGCCTCTTTTTTTAGTGGTTTAGAATAGAACAAGTAATAAAACGTCTAGGAATTTTAANNNNNNNNNNNNNNNNNNNTATCAAGTCAAGATTGATTTATAGTATAGAATTGGTATATTNNNNNNNNNNNNNNNNNNNNCCTTTTATTTATTATTTATACCTTTTATTTATTNNNNNNNNNNNNATTTAATTAGAATCCAATCTCGAGGGAGGATTTTCTATCGAAAGAAAAGAGAAGACTAGGTCTAAGTAAGGTATACGAAGGAAAGCCCCTTTTACGTTGTTGACAATGTTTACAATGAAACTTAAATTAGCAAATTGGTTTTTAAAGCTTTATCTTGTGCACAACTACCGCACAATACCTGCGTAAATTACTATTAGATTTGATTGGGGTTGGGTTTTTTTAACCAGACTCGGGTCATCATACAGAATTCACTCGAATCGGATATACCAAGGGTAGTATAATTAACTCGTTGATTCGATCTTTTCTCTCTTTTC

060_20180623_04_04_ndhCret-trnV-comp AATACCAAAATCGGAATAAGACTTGATATTATCAGAAATGCCCAAAAAATATCATATTCGTAAAGCAGAAACATAGATGCACTCCTATGAACATGGAAAATATACCGGATTAGTCAATCCGACTTGAAATTCTTAAGTCGTTCATAACTGTTTAGTTAAAGTAAGAATTGCTTTTGATCGAACCATCTAGTTTCCTTTCAAGATTAATCAATTAGAATCCATTTTTGTTTTTTTATTTTTTNNNAGTTTATAGTTAAAACTAACTATTGCTCTTATGCAAATTCTCTTGTTTTCATCTCAATCTTACCGAGGATTCTCTCTAAAGAAAAGGGATTCCAAATAGAATTCTCATTTTTTTCTATTTTTTATTAGTTATAGTTAAGTTATTAGGAATTGGTCAAAATTGACATCGATTTGCATTATCTTTTCTATTTTATTATCNNNNNNNNNNNNNNTTTGTTTATATTTATTTCAATTTTTTATGAATATGTCCATTTGCCTCTTTTTTTAGTGGTTTAGAATAGAACAAGTAATAAAACGTCTAGGAATTTTAANNNNNNNNNNNNNNNNNNNTATCAAGTCAAGATTGATTTATAGTATAGAATTGGTATATTNNNNNNNNNNNNNNNNNNNNCCTTTTATTTATTATTTATACCTTTTATTTATTNNNNNNNNNNNNATTTAATTAGAATCCAATCTCGAGGGAGGATTTTCTATCGAAAGAAAAGAGAAGACTAGGTCTAAGTAAGGTATACGAAGGAAAGCCCCTTTTACGTTGTTGACAATGTTTACAATGAAACTTAAATTAGCAAATTGGTTTTTAAAGCTTTATCTTGTGCACAACTACCGCACAATACCTGCGTAAATTACTATTAGATTTGATTGGGGTTGGGTTTTTTTAACCAGACTCGGGTCATCATACAGAATTCACTCGAATCGGATATACCAAGGGTAGTATAATTAACTCGTTGATTCGATCTTTTCTCTCTTTTC

061_20180623_04_05_ndhCret-trnV-comp AATACCAAAATCGGAATAAGACTTGATATTATCAGAAATGCCCAAAAAATATCATATTCGTAAAGCAGAAACATAGATGCACTCCTATGAACATGGAAAATATACCGGATTAGTCAATCCGACTTGAAATTCTTAAGTCGTTCATAACTGTTTAGTTAAAGTAAGAATTGCTTTTGATCGAACCATCTAGTTTCCTTTCAAGATTAATCAATTAGAATCCATTTTTGTTTTTTTATTTTTTNNNAGTTTATAGTTAAAACTAACTATTGCTCTTATGCAAATTCTCTTGTTTTCATCTCAATCTTACCGAGGATTCTCTCTAAAGAAAAGGGATTCCAAATAGAATTCTCATTTTTTTCTATTTTTTATTAGTTATAGTTAAGTTATTAGGAATTGGTCAAAATTGACATCGATTTGCATTATCTTTTCTATTTTATTATCNNNNNNNNNNNNNNTTTGTTTATATTTATTTCAATTTTTTATGAATATGTCCATTTGCCTCTTTTTTTAGTGGTTTAGAATAGAACAAGTAATAAAACGTCTAGGAATTTTAANNNNNNNNNNNNNNNNNNNTATCAAGTCAAGATTGATTTATAGTATAGAATTGGTATATTNNNNNNNNNNNNNNNNNNNNCCTTTTATTTATTATTTATACCTTTTATTTATTNNNNNNNNNNNNATTTAATTAGAATCCAATCTCGAGGGAGGATTTTCTATCGAAAGAAAAGAGAAGACTAGGTCTAAGTAAGGTATACGAAGGAAAGCCCCTTTTACGTTGTTGACAATGTTTACAATGAAACTTAAATTAGCAAATTGGTTTTTAAAGCTTTATCTTGTGCACAACTACCGCACAATACCTGCGTAAATTACTATTAGATTTGATTGGGGTTGGGTTTTTTTAACCAGACTCGGGTCATCATACAGAATTCACTCGAATCGGATATACCAAGGGTAGTATAATTAACTCGTTGATTCGATCTTTTCTCTCTTTTC

062_20180713_02_01_ndhCret-trnV-comp AATACCAAAATCGGAATAAGACTTGATATTATCAGAAATGCCCAAAAAATATCATATTCGTAAAGCAGAAACATAGATGCACTCCTATGAACATGGAAAATATACCGGATTAGTCAATCCGACTTGAAATTCTTAAGTCGTTCATAACTATTTAGTTAAAGTAAGAATTGCTTTTGATCGAACCATCTAGTTTCCTTTCAAGATTAATCAATTAGAATCCATTTTTGTTTTTTTATTTTTTTTTAGTTTATAGTTAAAACTAACTATTGCTCTTATGCAAATTCTCTTGTTTTCATCTCAATCTTACCGAGGATTCTCTCTAAAGAAAAGGGATTCCAAATAGAATTCTCATTTTTTTCTATTTTTTATTAGTTATAGTTAAGTTATTAGGAATTGGTCAAAATTGACATCGATTTGCATTATCTTTTCTATTTTATTATCNNNNNNNNNNNNNNTTTGTTTATATTTATTTCAATTTTTTATGAATATGTCCATTTGCCTCTTTTTTTAGTGGTTTAGAATAGAACAAGTAATAAAACGTCTAGGAATTTGAATATCAAGTCAAGATTGATTTATCAAGTCAAGATTGATTTATAGTATAGAATTGGTATATTNNNNNNNNNNNNNNNNNNNNCCTTTTATTTATTATTTATACCTTTTATTTATTNNNNNNNNNNNNATTTAATTAGAATCCAATCTCGAGGGAGGATTTTCTATCGAAAGAAAAGAGAAGACTAGGTCTAAGTAAGGTATACGAAGGAAAGNNNNNTTTACGTTGTTGACAATGTTTACAATGAAACTTAAATTAGCAAATTGGTTTTTAAAGCTTTATCTTGTGCACAACTACCGCACAATACCTGCGTAAATTACTATTAGATTTGATTGGGGTTGGGTTTTTTTAACCAGACTCGGGTCATCATACAGAATTCACTCGAATCGGATATACCAAGGGTAGTATAATTAACTCGTTGATTCGATCTCTTCTCCCCTTTT

063_20180713_02_02_ndhCret-trnV-comp AATACCAAAATCGGAATAAGACTTGATATTATCAGAAATGCCCAAAAAATATCATATTCGTAAAGCAGAAACATAGATGCACTCCTATGAACATGGAAAATATACCGGATTAGTCAATCCGACTTGAAATTCTTAAGTCGTTCATAACTATTTAGTTAAAGTAAGAATTGCTTTTGATCGAACCATCTAGTTTCCTTTCAAGATTAATCAATTAGAATCCATTTTTGTTTTTTTATTTTTTTTTAGTTTATAGTTAAAACTAACTATTGCTCTTATGCAAATTCTCTTGTTTTCATCTCAATCTTACCGAGGATTCTCTCTAAAGAAAAGGGATTCCAAATAGAATTCTCATTTTTTTCTATTTTTTATTAGTTATAGTTAAGTTATTAGGAATTGGTCAAAATTGACATCGATTTGCATTATCTTTTCTATTTTATTATCNNNNNNNNNNNNNNTTTGTTTATATTTATTTCAATTTTTTATGAATATGTCCATTTGCCTCTTTTTTTAGTGGTTTAGAATAGAACAAGTAATAAAACGTCTAGGAATTTGAATATCAAGTCAAGATTGATTTATCAAGTCAAGATTGATTTATAGTATAGAATTGGTATATTNNNNNNNNNNNNNNNNNNNNCCTTTTATTTATTATTTATACCTTTTATTTATTNNNNNNNNNNNNATTTAATTAGAATCCAATCTCGAGGGAGGATTTTCTATCGAAAGAAAAGAGAAGACTAGGTCTAAGTAAGGTATACGAAGGAAAGNNNNNTTTACGTTGTTGACAATGTTTACAATGAAACTTAAATTAGCAAATTGGTTTTTAAAGCTTTATCTTGTGCACAACTACCGCACAATACCTGCGTAAATTACTATTAGATTTGATTGGGGTTGGGTTTTTTTAACCAGACTCGGGTCATCATACAGAATTCACTCGAATCGGATATACCAAGGGTAGTATAATTAACTCGTTGATTCGATCTCTTCTCCCCTTTT

064_20180713_02_03_ndhCret-trnV-comp AATACCAAAATCGGAATAAGACTTGATATTATCAGAAATGCCCAAAAAATATCATATTCGTAAAGCAGAAACATAGATGCACTCCTATGAACATGGAAAATATACCGGATTAGTCAATCCGACTTGAAATTCTTAAGTCGTTCATAACTGTTTAGTTAAAGTAAGAATTGCTTTTGATCGAACCATCTAGTTTCCTTTCAAGATTAATCAATTAGAATCCATTTTTGTTTTTTTATTTTTTTTTAGTTTATAGTTAAAACTAACTATTGCTCTTATGCAAATTCTCTTGTTTTCATCTCAATCTTACCGAGGATTCTCTCTAAAGAAAAGGGATTCCAAATAGAATTCTCATTTTTTTCTATTTTTTATTAGTTATAGTTAAGTTATTAGGAATTGGTCAAAATTGACATCGATTTGCATTATCTTTTCTATTTTATTATCNNNNNNNNNNNNNNTTTGTTTATATTTATTTCAATTTTTTATGAATATGTCCATTTGCCTCTTTTTTTAGTGGTTTAGAATAGAACAAGTAATAAAACGTCTAGGAATTTTAANNNNNNNNNNNNNNNNNNNTATCAAGTCAAGATTGATTTATAGTATAGAATTGGTATATTNNNNNNNNNNNNNNNNNNNNCCTTTTATTTATTATTTATACCTTTTATTTATTNNNNNNNNNNNNATTTAATTAGAATCCAATCTCGAGGGAGGATTTTCTATCGAAAGAAAAGAGAAGACTAGGTCTAAGTAAGGTATACGAAGGAAAGNNNNNTTTACGTTGTTGACAATGTTTACAATGAAACTTAAATTAGCAAATTGGTTTTTAAAGCTTTATCTTGTGCACAACTACCGCACAATACCTGCGTAAATAACTATTAGATTTGATTGGGGTTGGGTTTTTTTAACCAGACTCGGGTCATCATACAGAATTCACTCGAATCGGATATACCAAGGGTAGTATAATTAACTCGTTGATTCGATCTTTTCTCTCCTTTT

065_20180713_02_04_ndhCret-trnV-comp AATACCAAAATCGGAATAAGACTTGATATTATCAGAAATGCCCAAAAAATATCATATTCGTAAAGCAGAAACATAGATGCACTCCTATGAACATGGAAAATATACCGGATTAGTCAATCCGACTTGAAATTCTTAAGTCGTTCATAACTGTTTAGTTAAAGTAAGAATTGCTTTTGATCGAACCATCTAGTTTCCTTTCAAGATTAATCAATTAGAATCCATTTTTGTTTTTTTATTTTTTTTTAGTTTATAGTTAAAACTAACTATTGCTCTTATGCAAATTCTCTTGTTTTCATCTCAATCTTACCGAGGATTCTCTCTAAAGAAAAGGGATTCCAAATAGAATTCTCATTTTTTTCTATTTTTTATTAGTTATAGTTAAGTTATTAGGAATTGGTCAAAATTGACATCGATTTGCATTATCTTTTCTATTTTATTATCNNNNNNNNNNNNNNTTTGTTTATATTTATTTCAATTTTTTATGAATATGTCCATTTGCCTCTTTTTTTAGTGGTTTAGAATAGAACAAGTAATAAAACGTCTAGGAATTTTAANNNNNNNNNNNNNNNNNNNTATCAAGTCAAGATTGATTTATAGTATAGAATTGGTATATTNNNNNNNNNNNNNNNNNNNNCCTTTTATTTATTATTTATACCTTTTATTTATTNNNNNNNNNNNNATTTAATTAGAATCCAATCTCGAGGGAGGATTTTCTATCGAAAGAAAAGAGAAGACTAGGTCTAAGTAAGGTATACGAAGGAAAGNNNNNTTTACGTTGTTGACAATGTTTACAATGAAACTTAAATTAGCAAATTGGTTTTTAAAGCTTTATCTTGTGCACAACTACCGCACAATACCTGCGTAAATTACTATTAGATTTGATTGGGGTTGGGTTTTTTTAACCAGACTCGGGTCATCATACAGAATTCACTCGAATCGGATATACCAAGGGTAGTATAATTAACTCGTTGATTCGATCTTTTCTCTCCTTTT

066_20180713_02_05_ndhCret-trnV-comp AATACCAAAATCGGAATAAGACTTGATATTATCAGAAATGCCCAAAAAATATCATATTCGTAAAGCAGAAACATAGATGCACTCCTATGAACATGGAAAATATACCGGATTAGTCAATCCGACTTGAAATTCTTAAGTCGTTCATAACTGTTTAGTTAAAGTAAGAATTGCTTTTGATCGAACCATCTAGTTTCCTTTCAAGATTAATCAATTAGAATCCATTTTTGTTTTTTTATTTTTTTTTAGTTTATAGTTAAAACTAACTATTGCTCTTATGCAAATTCTCTTGTTTTCATCTCAATCTTACCGAGGATTCTCTCTAAAGAAAAGGGATTCCAAATAGAATTCTCATTTTTTTCTATTTTTTATTAGTTATAGTTAAGTTATTAGGAATTGGTCAAAATTGACATCGATTTGCATTATCTTTTCTATTTTATTATCNNNNNNNNNNNNNNTTTGTTTATATTTATTTCAATTTTTTATGAATATGTCCATTTGCCTCTTTTTTTAGTGGTTTAGAATAGAACAAGTAATAAAACGTCTAGGAATTTTAANNNNNNNNNNNNNNNNNNNTATCAAGTCAAGATTGATTTATAGTATAGAATTGGTATATTNNNNNNNNNNNNNNNNNNNNCCTTTTATTTATTATTTATACCTTTTATTTATTNNNNNNNNNNNNATTTAATTAGAATCCAATCTCGAGGGAGGATTTTCTATCGAAAGAAAAGAGAAGACTAGGTCTAAGTAAGGTATACGAAGGAAAGNNNNNTTTACGTTGTTGACAATGTTTACAATGAAACTTAAATTAGCAAATTGGTTTTTAAAGCTTTATCTTGTGCACAACTACCGCACAATACCTGCGTAAATTACTATTAGATTTGATTGGGGTTGGGTTTTTTTAACCAGACTCGGGTCATCATACAGAATTCACTCGAATCGGATATACCAAGGGTAGTATAATTAACTCGTTGATTCGATCTTTTCTCTCCTTTT

067_20180713_03_01_ndhCret-trnV-comp AATACCAAAATCGGAATAAGACTTGATATTATCAGAAATGCCCAAAAAATATCATATTCGTAAAGCAGAAACATAGATGCACTCCTATGAACATGGAAAATATACCGGATTAGTCAATCCGACTTGAAATTCTTAAGTCGTTCATAACTGTTTAGTTAAAGTAAGAATTGCTTTTGATCGAACCATCTAGTTTCCTTTCAAGATTAATCAATTAGAATCCATTTTTGTTTTTTTATTTTTTTTTAGTTTATAGTTAAAACTAACTATTGCTCTTATGCAAATTCTCTTGTTTTCATCTCAATCTTACCGAGGATTCTCTCTAAAGAAAAGGGATTCCAAATAGAATTCTCATTTTTTTCTATTTTTTATTAGTTATAGTTAAGTTATTAGGAATTGGTCAAAATTGACATCGATTTGCATTATCTTTTCTATTTTATTATCNNNNNNNNNNNNNNTTTGTTTATATTTATTTCAATTTTTTATGAATATGTCCATTTGCCTCTTTTTTTAGTGGTTTAGAATAGAACAAGTAATAAAACGTCTAGGAATTTTAANNNNNNNNNNNNNNNNNNNTATCAAGTCAAGATTGATTTATAGTATAGAATTGGTATATTNNNNNNNNNNNNNNNNNNNNCCTTTTATTTATTATTTATACCTTTTATTTATTNNNNNNNNNNNNATTTAATTAGAATCCAATCTCGAGGGAGGATTTTCTATCGAAAGAAAAGAGAAGACTAGGTCTAAGTAAGGTATACGAAGGAAAGCCCCTTTTACGTTGTTGACAATGTTTACAATGAAACTTAAATTAGCAAATTGGTTTTTAAAGCTTTATCTTGTGCACAACTACCGCACAATACCTGCGTAAATTACTATTAGATTTGATTGGGGTTGGGTTTTTTTAACCAGACTCGGGTCATCATACAGAATTCACTCGAATCGGATATACCAAGGGTAGTATAATTAACTCGTTGATTCGATCTTTTCTCTCCTTTC

068_20180713_03_02_ndhCret-trnV-comp AATACCAAAATCGGAATAAGACTTGATATTATCAGAAATGCCCAAAAAATATCATATTCGTAAAGCAGAAACATAGATGCACTCCTATGAACATGGAAAATATACCGGATTAGTCAATCCGACTTGAAATTCTTAAGTCGTTCATAACTGTTTAGTTAAAGTAAGAATTGCTTTTGATCGAACCATCTAGTTTCCTTTCAAGATTAATCAATTAGAATCCATTTTTGTTTTTTTATTTTTTTTTAGTTTATAGTTAAAACTAACTATTGCTCTTATGCAAATTCTCTTGTTTTCATCTCAATCTTACCGAGGATTCTCTCTAAAGAAAAGGGATTCCAAATAGAATTCTCATTTTTTTCTATTTTTTATTAGTTATAGTTAAGTTATTAGGAATTGGTCAAAATTGACATCGATTTGCATTATCTTTTCTATTTTATTATCNNNNNNNNNNNNNNTTTGTTTATATTTATTTCAATTTTTTATGAATATGTCCATTTGCCTCTTTTTTTAGTGGTTTAGAATAGAACAAGTAATAAAACGTCTAGGAATTTTAANNNNNNNNNNNNNNNNNNNTATCAAGTCAAGATTGATTTATAGTATAGAATTGGTATATTNNNNNNNNNNNNNNNNNNNNCCTTTTATTTATTATTTATACCTTTTATTTATTNNNNNNNNNNNNATTTAATTAGAATCCAATCTCGAGGGAGGATTTTCTATCGAAAGAAAAGAGAAGACTAGGTCTAAGTAAGGTATACGAAGGAAAGCCCCTTTTACGTTGTTGACAATGTTTACAATGAAACTTAAATTAGCAAATTGGTTTTTAAAGCTTTATCTTGTGCACAACTACCGCACAATACCTGCGTAAATTACTATTAGATTTGATTGGGGTTGGGTTTTTTTAACCAGACTCGGGTCATCATACAGAATTCACTCGAATCGGATATACCAAGGGTAGTATAATTAACTCGTTGATTCGATCTTTTCTCTCCTTTC

069_20180713_03_03_ndhCret-trnV-comp AATACCAAAATCGGAATAAGACTTGATATTATCAGAAATGCCCAAAAAATATCATATTCGTAAAGCAGAAACATAGATGCACTCCTATGAACATGGAAAATATACCGGATTAGTCAATCCGACTTGAAATTCTTAAGTCGTTCATAACTGTTTAGTTAAAGTAAGAATTGCTTTTGATCGAACCATCTAGTTTCCTTTCAAGATTAATCAATTAGAATCCATTTTTGTTTTTTTATTTTTTTTTAGTTTATAGTTAAAACTAACTATTGCTCTTATGCAAATTCTCTTGTTTTCATCTCAATCTTACCGAGGATTCTCTCTAAAGAAAAGGGATTCCAAATAGAATTCTCATTTTTTTCTATTTTTTATTAGTTATAGTTAAGTTATTAGGAATTGGTCAAAATTGACATCGATTTGCATTATCTTTTCTATTTTATTATCNNNNNNNNNNNNNNTTTGTTTATATTTATTTCAATTTTTTATGAATATGTCCATTTGCCTCTTTTTTTAGTGGTTTAGAATAGAACAAGTAATAAAACGTCTAGGAATTTTAANNNNNNNNNNNNNNNNNNNTATCAAGTCAAGATTGATTTATAGTATAGAATTGGTATATTNNNNNNNNNNNNNNNNNNNNCCTTTTATTTATTATTTATACCTTTTATTTATTNNNNNNNNNNNNATTTAATTAGAATCCAATCTCGAGGGAGGATTTTCTATCGAAAGAAAAGAGAAGACTAGGTCTAAGTAAGGTATACGAAGGAAAGCCCCTTTTACGTTGTTGACAATGTTTACAATGAAACTTAAATTAGCAAATTGGTTTTTAAAGCTTTATCTTGTGCACAACTACCGCACAATACCTGCGTAAATTACTATTAGATTTGATTGGGGTTGGGTTTTTTTAACCAGACTCGGGTCATCATACAGAATTCACTCGAATCGGATATACCAAGGGTAGTATAATTAACTCGTTGATTCGATCTTTTCTCTCCTTTC

070_20180713_03_04_ndhCret-trnV-comp AATACCAAAATCGGAATAAGACTTGATATTATCAGAAATGCCCAAAAAATATCATATTCGTAAAGCAGAAACATAGATGCACTCCTATGAACATGGAAAATATACCGGATTAGTCAATCCGACTTGAAATTCTTAAGTCGTTCATAACTGTTTAGTTAAAGTAAGAATTGCTTTTGATCGAACCATCTAGTTTCCTTTCAAGATTAATCAATTAGAATCCATTTTTGTTTTTTTATTTTTTTTTAGTTTATAGTTAAAACTAACTATTGCTCTTATGCAAATTCTCTTGTTTTCATCTCAATCTTACCGAGGATTCTCTCTAAAGAAAAGGGATTCCAAATAGAATTCTCATTTTTTTCTATTTTTTATTAGTTATAGTTAAGTTATTAGGAATTGGTCAAAATTGACATCGATTTGCATTATCTTTTCTATTTTATTATCNNNNNNNNNNNNNNTTTGTTTATATTTATTTCAATTTTTTATGAATATGTCCATTTGCCTCTTTTTTTAGTGGTTTAGAATAGAACAAGTAATAAAACGTCTAGGAATTTTAANNNNNNNNNNNNNNNNNNNTATCAAGTCAAGATTGATTTATAGTATAGAATTGGTATATTNNNNNNNNNNNNNNNNNNNNCCTTTTATTTATTATTTATACCTTTTATTTATTNNNNNNNNNNNNATTTAATTAGAATCCAATCTCGAGGGAGGATTTTCTATCGAAAGAAAAGAGAAGACTAGGTCTAAGTAAGGTATACGAAGGAAAGCCCCTTTTACGTTGTTGACAATGTTTACAATGAAACTTAAATTAGCAAATTGGTTTTTAAAGCTTTATCTTGTGCACAACTACCGCACAATACCTGCGTAAATTACTATTAGATTTGATTGGGGTTGGGTTTTTTTAACCAGACTCGGGTCATCATACAGAATTCACTCGAATCGGATATACCAAGGGTAGTATAATTAACTCGTTGATTCGATCTTTTCTCTCCTTTC

071_20180713_03_05_ndhCret-trnV-comp AATACCAAAATCGGAATAAGACTTGATATTATCAGAAATGCCCAAAAAATATCATATTCGTAAAGCAGAAACATAGATGCACTCCTATGAACATGGAAAATATACCGGATTAGTCAATCCGACTTGAAATTCTTAAGTCGTTCATAACTGTTTAGTTAAAGTAAGAATTGCTTTTGATCGAACCATCTAGTTTCCTTTCAAGATTAATCAATTAGAATCCATTTTTGTTTTTTTATTTTTTTTTAGTTTATAGTTAAAACTAACTATTGCTCTTATGCAAATTCTCTTGTTTTCATCTCAATCTTACCGAGGATTCTCTCTAAAGAAAAGGGATTCCAAATAGAATTCTCATTTTTTTCTATTTTTTATTAGTTATAGTTAAGTTATTAGGAATTGGTCAAAATTGACATCGATTTGCATTATCTTTTCTATTTTATTATCNNNNNNNNNNNNNNTTTGTTTATATTTATTTCAATTTTTTATGAATATGTCCATTTGCCTCTTTTTTTAGTGGTTTAGAATAGAACAAGTAATAAAACGTCTAGGAATTTTAANNNNNNNNNNNNNNNNNNNTATCAAGTCAAGATTGATTTATAGTATAGAATTGGTATATTNNNNNNNNNNNNNNNNNNNNCCTTTTATTTATTATTTATACCTTTTATTTATTNNNNNNNNNNNNATTTAATTAGAATCCAATCTCGAGGGAGGATTTTCTATCGAAAGAAAAGAGAAGACTAGGTCTAAGTAAGGTATACGAAGGAAAGCCCCTTTTACGTTGTTGACAATGTTTACAATGAAACTTAAATTAGCAAATTGGTTTTTAAAGCTTTATCTTGTGCACAACTACCGCACAATACCTGCGTAAATTACTATTAGATTTGATTGGGGTTGGGTTTTTTTAACCAGACTCGGGTCATCATACAGAATTCACTCGAATCGGATATACCAAGGGTAGTATAATTAACTCGTTGATTCGATCTTTTCTCTCCTTTC

072_20180714_02_01_ndhCret-trnV-comp AATACCAAAATCGGAATAAGACTTGATATTATCAGAAATGCCCAAAAAATATCATATTCGTAAAGCAGAAACATAGATGCACTCCTATGAACATGGAAAATATACCGGATTAGTCAATCCGACTTGAAATTCTTAAGTCGTTCATAACTGTTTAGTTAAAGTAAGAATTGCTTTTGATCGAACCATCTAGTTTCCTTTCAAGATTAATCAATTAGAATCCATTTTTGTTTTTTTATTTTTTTTTAGTTTATAGTTAAAACTAACTATTGCTCTTATGCAAATTCTCTTGTTTTCATCTCAATCTTACCGAGGATTCTCTCTAAAGAAAAGGGATTCCAAATAGAATTCTCATTTTTTTCTATTTTTTATTAGTTATAGTTAAGTTATTAGGAATTGGTCAAAATTGACATCGATTTGCATTATCTTTTCTATTTTATTATCNNNNNNNNNNNNNNTTTGTTTATATTTATTTCAATTTTTTATGAATATGTCCATTTGCCTCTTTTTTTAGTGGTTTAGAATAGAACAAGTAATAAAACGTCTAGGAATTTTAATATCAAGTCAAGATTGATTTATCAAGTCAAGATTGATTTATAGTATAGAATTGGTATATTNNNNNNNNNNNNNNNNNNNNCCTTTTATTTATTATTTATACCTTTTATTTATTNNNNNNNNNNNNATTTAATTAGAATCCAATCTCGAGGGAGGATTTTCTATCGAAAGAAAAGAGAAGACTAGGTCTAAGTAAGGTATACGAAGGAAAGCCCCTTTTACGTTGTTGACAATGTTTACAATGAAACTTAAATTAGCAAATTGGTTTTTAAAGCTTTATCTTGTGCACAACTACCGCACAATACCTGCGTAAATTACTATTAGATTTGATTGGGGTTGGGTTTTTTTAACCAGACTCGGGTCATCATACAGAATTCACTCGAATCGGATATACCAAGGGTAGTATAATTAACTCGTTGATTCGATCTTTTTTCTCCTTTC

073_20180714_02_02_ndhCret-trnV-comp AATACCAAAATCGGAATAAGACTTGATATTATCAGAAATGCCCAAAAAATATCATATTCGTAAAGCAGAAACATAGATGCACTCCTATGAACATGGAAAATATACCGGATTAGTCAATCCGACTTGAAATTCTTAAGTCGTTCATAACTGTTTAGTTAAAGTAAGAATTGCTTTTGATCGAACCATCTAGTTTCCTTTCAAGATTAATCAATTAGAATCCATTTTTTTTTTTTTATTTTTTTTTAGTTTATAGTTAAAACTAACTATTGCTCTTATGCAAATTCTCTTGTTTTCATCTCAATCTTACCGAGGATTCTCTCTAAAGAAAAGGGATTCCAAATAGAATTCTCATTTTTTTCTATTTTTTATTAGTTATAGTTAAGTTATTAGGAATTGGTCAAAATTGACATCGATTTGCATTATCTTTTCTATTTTATTATCTTTGTTTATATTTATTTGTTTATATTTATTTCAATTTTTTATGAATATGTCCATTTGCCTCTTTTTTTAGTGGTTTAGAATAGAACAAGTAATAAAACGTCTAGGAATTTTAANNNNNNNNNNNNNNNNNNNTATCAAGTCAAGATTGATTTATAGTATAGAATTGGTATATTNNNNNNNNNNNNNNNNNNNNCCTTTTATTTATTATTTATACCTTTTATTTATTNNNNNNNNNNNNATTTAATTAGAATCCAATCTCGAGGGAGGATTTTCTATCGAAAGAAAAGAGAAGACTAGGTCTAAGTAAGGTATACGAAGGAAAGCCCCTTTTACGTTGTTGACAATGTTTACAATGAAACTTAAATTAGCAAATTGGTTTTTAAAGCTTTATCTTGTGCACAACTACCGCACAATACCTGCGTAAATTACTATTAGATTTGATTGGGGTTGGGTTTTTTTAACCAGACTCGGGTCATCATACAGAATTCACTCGAATCGGATATACCAAGGGTAGTATAATTAACTCGTTGATTCGATCTTTTCTCTCCCTTC

074_20180714_03_01_ndhCret-trnV-comp AATACCAAAATCGGAATAAGACTTGATATTATCAGAAATGCCCAAAAAATATCATATTCGTAAAGCAGAAACATAGATGCACTCCTATGAACATGGAAAATATACCGGATTAGTCAATCCGACTTGAAATTCTTAAGTCGTTCATAACTGTTTAGTTAAAGTAAGAATTGCTTTTGATCGAACCATCTAGTTTCCTTTCAAGATTAATCAATTAGAATCCATTTTTGTTTTTTTATTTTTTTTTAGTTTATAGTTAAAACTAACTATTGCTCTTATGCAAATTCTCTTGTTTTCATCTCAATCTTACCGAGGATTCTCTCTAAAGAAAAGGGATTCCAAATAGAATTCTCATTTTTTTCTATTTTTTATTAGTTATAGTTAAGTTATTAGGAATTGGTCAAAATTGACATCGATTTGCATTATCTTTTCTATTTTATTATCNNNNNNNNNNNNNNTTTGTTTATATTTATTTCAATTTTTTATGAATATGTCCATTTGCCTCTTTTTTTAGTGGTTTAGAATAGAACAAGTAATAAAACGTCTAGGAATTTTAATATCAAGTCAAGATTGATTTATCAAGTCAAGATTGATTTATAGTATAGAATTGGTATATTNNNNNNNNNNNNNNNNNNNNCCTTTTATTTATTATTTATACCTTTTATTTATTNNNNNNNNNNNNATTTAATTAGAATCCAATCTCGAGGGAGGATTTTCTATCGAAAGAAAAGAGAAGACTAGGTCTAAGTAAGGTATACGAAGGAAAGCCCCTTTTACGTTGTTGACAATGTTTACAATGAAACTTAAATTAGCAAATTGGTTTTTAAAGCTTTATCTTGTGCACAACTACCGCACAATACCTGCGTAAATTACTATTAGATTTGATTGGGGTTGGGTTTTTTTAACCAGACTCGGGTCATCATACAGAATTCACTCGAATCGGATATACCAAGGGTAGTATAATTAACTCGTTGATTCGATCTTTTTTCTCCTTTC

075_20180714_03_02_ndhCret-trnV-comp AATACCAAAATCGGAATAAGACTTGATATTATCAGAAATGCCCAAAAAATATCATATTCGTAAAGCAGAAACATAGATGCACTCCTATGAACATGGAAAATATACCGGATTAGTCAATCCGACTTGAAATTCTTAAGTCGTTCATAACTGTTTAGTTAAAGTAAGAATTGCTTTTGATCGAACCATCTAGTTTCCTTTCAAGATTAATCAATTAGAATCCATTTTTGTTTTTTTATTTTTTTTTAGTTTATAGTTAAAACTAACTATTGCTCTTATGCAAATTCTCTTGTTTTCATCTCAATCTTACCGAGGATTCTCTCTAAAGAAAAGGGATTCCAAATAGAATTCTCATTTTTTTCTATTTTTTATTAGTTATAGTTAAGTTATTAGGAATTGGTCAAAATTGACATCGATTTGCATTATCTTTTCTATTTTATTATCNNNNNNNNNNNNNNTTTGTTTATATTTATTTCAATTTTTTATGAATATGTCCATTTGCCTCTTTTTTTAGTGGTTTAGAATAGAACAAGTAATAAAACGTCTAGGAATTTTAATATCAAGTCAAGATTGATTTATCAAGTCAAGATTGATTTATAGTATAGAATTGGTATATTNNNNNNNNNNNNNNNNNNNNCCTTTTATTTATTATTTATACCTTTTATTTATTNNNNNNNNNNNNATTTAATTAGAATCCAATCTCGAGGGAGGATTTTCTATCGAAAGAAAAGAGAAGACTAGGTCTAAGTAAGGTATACGAAGGAAAGCCCCTTTTACGTTGTTGACAATGTTTACAATGAAACTTAAATTAGCAAATTGGTTTTTAAAGCTTTATCTTGTGCACAACTACCGCACAATACCTGCGTAAATTACTATTAGATTTGATTGGGGTTGGGTTTTTTTAACCAGACTCGGGTCATCATACAGAATTCACTCGAATCGGATATACCAAGGGTAGTATAATTAACTCGTTGATTCGATCTTTTTTCTCCTTTC

076_20180714_03_03_ndhCret-trnV-comp AATACCAAAATCGGAATAAGACTTGATATTATCAGAAATGCCCAAAAAATATCATATTCGTAAAGCAGAAACATAGATGCACTCCTATGAACATGGAAAATATACCGGATTAGTCAATCCGACTTGAAATTCTTAAGTCGTTCATAACTGTTTAGTTAAAGTAAGAATTGCTTTTGATCGAACCATCTAGTTTCCTTTCAAGATTAATCAATTAGAATCCATTTTTGTTTTTTTATTTTTTTTTAGTTTATAGTTAAAACTAACTATTGCTCTTATGCAAATTCTCTTGTTTTCATCTCAATCTTACCGAGGATTCTCTCTAAAGAAAAGGGATTCCAAATAGAATTCTCATTTTTTTCTATTTTTTATTAGTTATAGTTAAGTTATTAGGAATTGGTCAAAATTGACATCGATTTGCATTATCTTTTCTATTTTATTATCNNNNNNNNNNNNNNTTTGTTTATATTTATTTCAATTTTTTATGAATATGTCCATTTGCCTCTTTTTTTAGTGGTTTAGAATAGAACAAGTAATAAAACGTCTAGGAATTTTAATATCAAGTCAAGATTGATTTATCAAGTCAAGATTGATTTATAGTATAGAATTGGTATATTNNNNNNNNNNNNNNNNNNNNCCTTTTATTTATTATTTATACCTTTTATTTATTNNNNNNNNNNNNATTTAATTAGAATCCAATCTCGAGGGAGGATTTTCTATCGAAAGAAAAGAGAAGACTAGGTCTAAGTAAGGTATACGAAGGAAAGCCCCTTTTACGTTGTTGACAATGTTTACAATGAAACTTAAATTAGCAAATTGGTTTTTAAAGCTTTATCTTGTGCACAACTACCGCACAATACCTGCGTAAATTACTATTAGATTTGATTGGGGTTGGGTTTTTTTAACCAGACTCGGGTCATCATACAGAATTCACTCGAATCGGATATACCAAGGGTAGTATAATTAACTCGTTGATTCGATCTTTTTTCTCCTTTC

077_20180715_01_01_ndhCret-trnV-comp AATACCAAAATCGGAATAAGACTTGATATTATCAGAAATGCCCAAAAAATATCATATTCGTAAAGCAGAAACATAGATGCACTCCTATGAACATGGAAAATATACCGGATTAGTCAATCCGACTTGAAATTCTTAAGTCGTTCATAACTGTTTAGTTAAAGTAAGAATTGCTTTTGATCGAACCATCTAGTTTCCTTTCAAGATTAATCAATTAGAATCCATTTTTTTTTTTTTATTTTTTTTTAGTTTATAGTTAAAACTAACTATTGCTCTTATGCAAATTCTCTTGTTTTCATCTCAATCTTACCGAGGATTCTCTCTAAAGAAAAGGGATTCCAAATAGAATTCTCATTTTTTTCTATTTTTTATTAGTTATAGTTAAGTTATTAGGAATTGGTCAAAATTGACATCGATTTGCATTATCTTTTCTATTTTATTATCTTTGTTTATATTTATTTGTTTATATTTATTTCAATTTTTTATGAATATGTCCATTTGCCTCTTTTTTTAGTGGTTTAGAATAGAACAAGTAATAAAACGTCTAGGAATTTTAANNNNNNNNNNNNNNNNNNNTATCAAGTCAAGATTGATTTATAGTATAGAATTGGTATATTNNNNNNNNNNNNNNNNNNNNCCTTTTATTTATTATTTATACCTTTTATTTATTNNNNNNNNNNNNATTTAATTAGAATCCAATCTCGAGGGAGGATTTTCTATCGAAAGAAAAGAGAAGACTAGGTCTAAGTAAGGTATACGAAGGAAAGCCCCTTTTACGTTGTTGACAATGTTTACAATGAAACTTAAATTAGCAAATTGGTTTTTAAAGCTTTATCTTGTGCACAACTACCGCACAATACCTGCGTAAATTACTATTAGATTTGATTGGGGTTGGGTTTTTTTAACCAGACTCGGGTCATCATACAGAATTCACTCGAATCGGATATACCAAGGGTAGTATAATTAACTCGTTGATTCGATCTTTTCTCTCCCTTC

078_20180715_01_02_ndhCret-trnV-comp AATACCAAAATCGGAATAAGACTTGATATTATCAGAAATGCCCAAAAAATATCATATTCGTAAAGCAGAAACATAGATGCACTCCTATGAACATGGAAAATATACCGGATTAGTCAATCCGACTTGAAATTCTTAAGTCGTTCATAACTGTTTAGTTAAAGTAAGAATTGCTTTTGATCGAACCATCTAGTTTCCTTTCAAGATTAATCAATTAGAATCCATTTTTTTTTTTTTATTTTTTTTTAGTTTATAGTTAAAACTAACTATTGCTCTTATGCAAATTCTCTTGTTTTCATCTCAATCTTACCGAGGATTCTCTCTAAAGAAAAGGGATTCCAAATAGAATTCTCATTTTTTTCTATTTTTTATTAGTTATAGTTAAGTTATTAGGAATTGGTCAAAATTGACATCGATTTGCATTATCTTTTCTATTTTATTATCTTTGTTTATATTTATTTGTTTATATTTATTTCAATTTTTTATGAATATGTCCATTTGCCTCTTTTTTTAGTGGTTTAGAATAGAACAAGTAATAAAACGTCTAGGAATTTTAANNNNNNNNNNNNNNNNNNNTATCAAGTCAAGATTGATTTATAGTATAGAATTGGTATATTNNNNNNNNNNNNNNNNNNNNCCTTTTATTTATTATTTATACCTTTTATTTATTNNNNNNNNNNNNATTTAATTAGAATCCAATCTCGAGGGAGGATTTTCTATCGAAAGAAAAGAGAAGACTAGGTCTAAGTAAGGTATACGAAGGAAAGCCCCTTTTACGTTGTTGACAATGTTTACAATGAAACTTAAATTAGCAAATTGGTTTTTAAAGCTTTATCTTGTGCACAACTACCGCACAATACCTGCGTAAATTACTATTAGATTTGATTGGGGTTGGGTTTTTTTAACCAGACTCGGGTCATCATACAGAATTCACTCGAATCGGATATACCAAGGGTAGTATAATTAACTCGTTGATTCGATCTTTTCTCTCCCTTC

079_20180715_01_03_ndhCret-trnV-comp AATACCAAAATCGGAATAAGACTTGATATTATCAGAAATGCCCAAAAAATATCATATTCGTAAAGCAGAAACATAGATGCACTCCTATGAACATGGAAAATATACCGGATTAGTCAATCCGACTTGAAATTCTTAAGTCGTTCATAACTGTTTAGTTAAAGTAAGAATTGCTTTTGATCGAACCATCTAGTTTCCTTTCAAGATTAATCAATTAGAATCCATTTTTTTTTTTTTATTTTTTTTTAGTTTATAGTTAAAACTAACTATTGCTCTTATGCAAATTCTCTTGTTTTCATCTCAATCTTACCGAGGATTCTCTCTAAAGAAAAGGGATTCCAAATAGAATTCTCATTTTTTTCTATTTTTTATTAGTTATAGTTAAGTTATTAGGAATTGGTCAAAATTGACATCGATTTGCATTATCTTTTCTATTTTATTATCTTTGTTTATATTTATTTGTTTATATTTATTTCAATTTTTTATGAATATGTCCATTTGCCTCTTTTTTTAGTGGTTTAGAATAGAACAAGTAATAAAACGTCTAGGAATTTTAANNNNNNNNNNNNNNNNNNNTATCAAGTCAAGATTGATTTATAGTATAGAATTGGTATATTNNNNNNNNNNNNNNNNNNNNCCTTTTATTTATAATTTATACCTTTTATTTATTNNNNNNNNNNNNATTTAATTAGAATCCAATCTCGAGGGAGGATTTTCTATCGAAAGAAAAGAGAAGACTAGGTCTAAGTAAGGTATACGAAGGAAAGCCCCTTTTACGTTGTTGACAATGTTTACAATGAAACTTAAATTAGCAAATTGGTTTTTAAAGCTTTATCTTGTGCACAACTACCGCACAATACCTGCGTAAATTACTATTAGATTTGATTGGGGTTGGGTTTTTTTAACCAGACTCGGGTCATCATACAGAATTCACTCGAATCGGATATACCAAGGGTAGTATAATTAACTCGTTGATTCGATCTTTTCTCTCCCTTC

080_20180715_01_04_ndhCret-trnV-comp AATACCAAAATCGGAATAAGACTTGATATTATCAGAAATGCCCAAAAAATATCATATTCGTAAAGCAGAAACATAGATGCACTCCTATGAACATGGAAAATATACCGGATTAGTCAATCCGACTTGAAATTCTTAAGTCGTTCATAACTGTTTAGTTAAAGTAAGAATTGCTTTTGATCGAACCATCTAGTTTCCTTTCAAGATTAATCAATTAGAATCCATTTTTTTTTTTTTATTTTTTTTTAGTTTATAGTTAAAACTAACTATTGCTCTTATGCAAATTCTCTTGTTTTCATCTCAATCTTACCGAGGATTCTCTCTAAAGAAAAGGGATTCCAAATAGAATTCTCATTTTTTTCTATTTTTTATTAGTTATAGTTAAGTTATTAGGAATTGGTCAAAATTGACATCGATTTGCATTATCTTTTCTATTTTATTATCTTTGTTTATATTTATTTGTTTATATTTATTTCAATTTTTTATGAATATGTCCATTTGCCTCTTTTTTTAGTGGTTTAGAATAGAACAAGTAATAAAACGTCTAGGAATTTTAANNNNNNNNNNNNNNNNNNNTATCAAGTCAAGATTGATTTATAGTATAGAATTGGTATATTNNNNNNNNNNNNNNNNNNNNCCTTTTATTTATTATTTATACCTTTTATTTATTNNNNNNNNNNNNATTTAATTAGAATCCAATCTCGAGGGAGGATTTTCTATCGAAAGAAAAGAGAAGACTAGGTCTAAGTAAGGTATACGAAGGAAAGCCCCTTTTACGTTGTTGACAATGTTTACAATGAAACTTAAATTAGCAAATTGGTTTTTAAAGCTTTATCTTGTGCACAACTACCGCACAATACCTGCGTAAATTACTATTAGATTTGATTGGGGTTGGGTTTTTTTAACCAGACTCGGGTCATCATACAGAATTCACTCGAATCGGATATACCAAGGGTAGTATAATTAACTCGTTGATTCGATCTTTTCTCTCCCTTC

081_20180715_01_05_ndhCret-trnV-comp AATACCAAAATCGGAATAAGACTTGATATTATCAGAAATGCCCAAAAAATATCATATTCGTAAAGCAGAAACATAGATGCACTCCTATGAACATGGAAAATATACCGGATTAGTCAATCCGACTTGAAATTCTTAAGTCGTTCATAACTGTTTAGTTAAAGTAAGAATTGCTTTTGATCGAACCATCTAGTTTCCTTTCAAGATTAATCAATTAGAATCCATTTTTTTTTTTTTATTTTTTTTTAGTTTATAGTTAAAACTAACTATTGCTCTTATGCAAATTCTCTTGTTTTCATCTCAATCTTACCGAGGATTCTCTCTAAAGAAAAGGGATTCCAAATAGAATTCTCATTTTTTTCTATTTTTTATTAGTTATAGTTAAGTTATTAGGAATTGGTCAAAATTGACATCGATTTGCATTATCTTTTCTATTTTATTATCTTTGTTTATATTTATTTGTTTATATTTATTTCAATTTTTTATGAATATGTCCATTTGCCTCTTTTTTTAGTGGTTTAGAATAGAACAAGTAATAAAACGTCTAGGAATTTTAANNNNNNNNNNNNNNNNNNNTATCAAGTCAAGATTGATTTATAGTATAGAATTGGTATATTNNNNNNNNNNNNNNNNNNNNCCTTTTATTTATTATTTATACCTTTTATTTATTNNNNNNNNNNNNATTTAATTAGAATCCAATCTCGAGGGAGGATTTTCTATCGAAAGAAAAGAGAAGACTAGGTCTAAGTAAGGTATACGAAGGAAAGCCCCTTTTACGTTGTTGACAATGTTTACAATGAAACTTAAATTAGCAAATTGGTTTTTAAAGCTTTATCTTGTGCACAACTACCGCACAATACCTGCGTAAATTACTATTAGATTTGATTGGGGTTGGGTTTTTTTAACCAGACTCGGGTCATCATACAGAATTCACTCGAATCGGATATACCAAGGGTAGTATAATTAACTCGTTGATTCGATCTTTTCTCTCCCTTC

082_20180716_01_01_ndhCret-trnV-comp AATACCAAAATCGGAATAAGACTTGATATTATCAGAAATGCCCAAAAAATATCATATTCGTAAAGCAGAAACATAGATGCACTCCTATGAACATGGAAAATATACCGGATTAGTCAATCCGACTTGAAATTCTTAAGTCGTTCATAACTGTTTAGTTAAAGTAAGAATTGCTTTTGATCGAACCATCTAGTTTCCTTTCAAGATTAATCAATTAGAATCCATTTTTGTTTTTTTATTTTTTTTTAGTTTATAGTTAAAACTAACTATTGCTCTTATGCAAATTCTCTTGTTTTCATCTCAATCTTACCGAGGATTCTCTCTAAAGAAAAGGGATTCCAAATAGAATTCTCATTTTTTTCTATTTTTTATTAGTTATAGTTAAGTTATTAGGAATTGGTCAAAATTGACATCGATTTGCATTATCTTTTCTATTTTATTATCNNNNNNNNNNNNNNTTTGTTTATATTTATTTCAATTTTTTATGAATATGTCCATTTGCCTCTTTTTTTAGTGGTTTAGAATAGAACAAGTAATAAAACGTCTAGGAATTTTAATATCAAGCCAAGATTGATTTATCAAGTCAAGATTGATTTATAGTATAGAATTGGTATATTNNNNNNNNNNNNNNNNNNNNCCTTTTATTTATTATTTATACCTTTTATTTATTNNNNNNNNNNNNATTTAATTAGAATCCAATCTCGAGGGAGGATTTTCTATCGAAAGAAAAGAGAAGACTAGGTCTAAGTAAGGTATACGAAGGAAAGCCCCTTTTACGTTGTTGACAATGTTTACAATGAAACTTAAATTAGCAAATTGGTTTTTAAAGCTTTATCTTGTGCACAACTACCGCACAATACCTGCGTAAATTACTATTAGATTTGATTGGGGTTGGGTTTTTTTAACCAGACTCGGGTCATCATACAGAATTCACTCGAATCGGATATACCAAGGGTAGTATAATTAACTCGTTGATTCGATCTTTTCTCTCCTTTC

083_20180716_02_01_ndhCret-trnV-comp AATACCAAAATCGGAATAAGACTTGATATTATCAGAAATGCCCAAAAAATATCATATTCGTAAAGCAGAAACATAGATGCACTCCTATGAACATGGAAAATATACCGGATTAGTCAATCCGACTTGAAATTCTTAAGTCGTTCATAACTGTTTAGTTAAAGTAAGAATTGCTTTTGATCGAACCATCTAGTTTCCTTTCAAGATTAATCAATTAGAATCCATTTTTGTTTTTTTATTTTTTTTTAGTTTATAGTTAAAACTAACTATTGCTCTTATGCAAATTCTCTTGTTTTCATCTCAATCTTACCGAGGATTCTCTCTAAAGAAAAGGGATTCCAAATAGAATTCTCATTTTTTTCTATTTTTTATTAGTTATAGTTAAGTTATTAGGAATTGGTCAAAATTGACATCGATTTGCATTATCTTTTCTATTTTATTATCNNNNNNNNNNNNNNTTTGTTTATATTTATTTCAATTTTTTATGAATATGTCCATTTGCCTCTTTTTTTAGTGGTTTAGAATAGAACAAGTAATAAAACGTCTAGGAATTTTAATATCAAGCCAAGATTGATTTATCAAGTCAAGATTGATTTATAGTATAGAATTGGTATATTNNNNNNNNNNNNNNNNNNNNCCTTTTATTTATTATTTATACCTTTTATTTATTNNNNNNNNNNNNATTTAATTAGAATCCAATCTCGAGGGAGGATTTTCTATCGAAAGAAAAGAGAAGACTAGGTCTAAGTAAGGTATACGAAGGAAAGCCCCTTTTACGTTGTTGACAATGTTTACAATGAAACTTAAATTAGCAAATTGGTTTTTAAAGCTTTATCTTGTGCACAACTACCGCACAATACCTGCGTAAATTACTATTAGATTTGATTGGGGTTGGGTTTTTTTAACCAGACTCGGGTCATCATACAGAATTCACTCGAATCGGATATACCAAGGGTAGTATAATTAACTCGTTGATTCGATCTTTTCTCTCCTTTC

084_20180716_02_02_ndhCret-trnV-comp AATACCAAAATCGGAATAAGACTTGATATTATCAGAAATGCCCAAAAAATATCATATTCGTAAAGCAGAAACATAGATGCACTCCTATGAACATGGAAAATATACCGGATTAGTCAATCCGACTTGAAATTCTTAAGTCGTTCATAACTGTTTAGTTAAAGTAAGAATTGCTTTTGATCGAACCATCTAGTTTCCTTTCAAGATTAATCAATTAGAATCCATTTTTGTTTTTTTATTTTTTTTTAGTTTATAGTTAAAACTAACTATTGCTCTTATGCAAATTCTCTTGTTTTCATCTCAATCTTACCGAGGATTCTCTCTAAAGAAAAGGGATTCCAAATAGAATTCTCATTTTTTTCTATTTTTTATTAGTTATAGTTAAGTTATTAGGAATTGGTCAAAATTGACATCGATTTGCATTATCTTTTCTATTTTATTATCNNNNNNNNNNNNNNTTTGTTTATATTTATTTCAATTTTTTATGAATATGTCCATTTGCCTCTTTTTTTAGTGGTTTAGAATAGAACAAGTAATAAAACGTCTAGGAATTTTAATATCAAGCCAAGATTGATTTATCAAGTCAAGATTGATTTATAGTATAGAATTGGTATATTNNNNNNNNNNNNNNNNNNNNCCTTTTATTTATTATTTATACCTTTTATTTATTNNNNNNNNNNNNATTTAATTAGAATCCAATCTCGAGGGAGGATTTTCTATCGAAAGAAAAGAGAAGACTAGGTCTAAGTAAGGTATACGAAGGAAAGCCCCTTTTACGTTGTTGACAATGTTTACAATGAAACTTAAATTAGCAAATTGGTTTTTAAAGCTTTATCTTGTGCACAACTACCGCACAATACCTGCGTAAATTACTATTAGATTTGATTGGGGTTGGGTTTTTTTAACCAGACTCGGGTCATCATACAGAATTCACTCGAATCGGATATACCAAGGGTAGTATAATTAACTCGTTGATTCGATCTTTTCTCTCCTTTC

085_20180716_02_03_ndhCret-trnV-comp AATACCAAAATCGGAATAAGACTTGATATTATCAGAAATGCCCAAAAAATATCATATTCGTAAAGCAGAAACATAGATGCACTCCTATGAACATGGAAAATATACCGGATTAGTCAATCCGACTTGAAATTCTTAAGTCGTTCATAACTGTTTAGTTAAAGTAAGAATTGCTTTTGATCGAACCATCTAGTTTCCTTTCAAGATTAATCAATTAGAATCCATTTTTGTTTTTTTATTTTTTTTTAGTTTATAGTTAAAACTAACTATTGCTCTTATGCAAATTCTCTTGTTTTCATCTCAATCTTACCGAGGATTCTCTCTAAAGAAAAGGGATTCCAAATAGAATTCTCATTTTTTTCTATTTTTTATTAGTTATAGTTAAGTTATTAGGAATTGGTCAAAATTGACATCGATTTGCATTATCTTTTCTATTTTATTATCNNNNNNNNNNNNNNTTTGTTTATATTTATTTCAATTTTTTATGAATATGTCCATTTGCCTCTTTTTTTAGTGGTTTAGAATAGAACAAGTAATAAAACGTCTAGGAATTTTAATATCAAGCCAAGATTGATTTATCAAGTCAAGATTGATTTATAGTATAGAATTGGTATATTNNNNNNNNNNNNNNNNNNNNCCTTTTATTTATTATTTATACCTTTTATTTATTNNNNNNNNNNNNATTTAATTAAAATCCAATCTCGAGGGAGGATTTTCTATCGAAAGAAAAGAGAAGACTAGGTCTAAGTAAGGTATACGAAGGAAAGCCCCTTTTACGTTGTTGACAATGTTTACAATGAAACTTAAATTAGCAAATTGGTTTTTAAAGCTTTATCTTGTGCACAACTACCGCACAATACCTGCGTAAATTACTATTAGATTTGATTGGGGTTGGGTTTTTTTAACCAGACTCGGGTCATCATACAGAATTCACTCGAATCGGATATACCAAGGGTAGTATAATTAACTCGTTGATTCGATCTTTTCTCTCCTTTC

086_20180716_02_04_ndhCret-trnV-comp AATACCAAAATCGGAATAAGACTTGATATTATCAGAAATGCCCAAAAAATATCATATTCGTAAAGCAGAAACATAGATGCACTCCTATGAACATGGAAAATATACCGGATTAGTCAATCCGACTTGAAATTCTTAAGTCGTTCATAACTGTTTAGTTAAAGTAAGAATTGCTTTTGATCGAACCATCTAGTTTCCTTTCAAGATTAATCAATTAGAATCCATTTTTGTTTTTTTATTTTTTTTTAGTTTATAGTTAAAACTAACTATTGCTCTTATGCAAATTCTCTTGTTTTCATCTCAATCTTACCGAGGATTCTCTCTAAAGAAAAGGGATTCCAAATAGAATTCTCATTTTTTTCTATTTTTTATTAGTTATAGTTAAGTTATTAGGAATTGGTCAAAATTGACATCGATTTGCATTATCTTTTCTATTTTATTATCNNNNNNNNNNNNNNTTTGTTTATATTTATTTCAATTTTTTATGAATATGTCCATTTGCCTCTTTTTTTAGTGGTTTAGAATAGAACAAGTAATAAAACGTCTAGGAATTTTAATATCAAGCCAAGATTGATTTATCAAGTCAAGATTGATTTATAGTATAGAATTGGTATATTNNNNNNNNNNNNNNNNNNNNCCTTTTATTTATTATTTATACCTTTTATTTATTNNNNNNNNNNNNATTTAATTAGAATCCAATCTCGAGGGAGGATTTTCTATCGAAAGAAAAGAGAAGACTAGGTCTAAGTAAGGTATACGAAGGAAAGCCCCTTTTACGTTGTTGACAATGTTTACAATGAAACTTAAATTAGCAAATTGGTTTTTAAAGCTTTATCTTGTGCACAACTACCGCACAATACCTGCGTAAATTACTATTAGATTTGATTGGGGTTGGGTTTTTTTAACCAGACTCGGGTCATCATACAGAATTCACTCGAATCGGATATACCAAGGGTAGTATAATTAACTCGTTGATTCGATCTTTTCTCTCCTTTC

087_20180717_01_01_ndhCret-trnV-comp AATACCAAAATCGGAATAAGACTTGATATTATCAGAAATGCCCAAAAAATATCATATTCGTAAAGCAGAAACATAGATGCACTCCTATGAACATGGAAAATATACCGGATTAGTCAATCCGACTTGAAATTCTTAAGTCGTTCATAACTGTTTAGTTAAAGTAAGAATTGCTTTTGATCGAACCATCTAGTTTCCTTTCAAGATTAATCAATTAGAATCCATTTTTGTTTTTTTATTTTTTTTTAGTTTATAGTTAAAACTAACTATTGCTCTTATGCAAATTCTCTTGTTTTCATCTCAATCTTACCGAGGATTCTCTCTAAAGAAAAGGGATTCCAAATAGAATTCTCATTTTTTTCTATTTTTTATTAGTTATAGTTAAGTTATTAGGAATTGGTCAAAATTGACATCGATTTGCATTATCTTTTCTATTTTATTATCNNNNNNNNNNNNNNTTTGTTTATATTTATTTCAATTTTTTATGAATATGTCCATTTGCCTCTTTTTTTAGTGGTTTAGAATAGAACAAGTAATAAAACGTCTAGGAATTTTAATATCAAGTCAAGATTGATTTATCAAGTCAAGATTGATTTATAGTATAGAATTGGTATATTNNNNNNNNNNNNNNNNNNNNCCTTTTATTTATTATTTATACCTTTTATTTATTNNNNNNNNNNNNATTTAATTAGAATCCAATCTCGAGGGAGGATTTTCTATCGAAAGAAAAGAGAAGACTAGGTCTAAGTAAGGTATACGAAGGAAAGCCCCTTTTACGTTGTTGACAATGTTTACAATGAAACTTAAATTAGCAAATTGGTTTTTAAAGCTTTATCTTGTGCACAACTACCGCACAATACCTGCGTAAATTACTATTAGATTTGATTGGGGTTGGGTTTTTTTAACCAGACTCGGGTCATCATACAGAATTCACTCGAATCGGATATACCAAGGGTAGTATAATTAACTCGTTGATTCGATCTTTTCTCTCCTTTC

088_20180717_01_02_ndhCret-trnV-comp AATACCAAAATCGGAATAAGACTTGATATTATCAGAAATGCCCAAAAAATATCATATTCGTAAAGCAGAAACATAGATGCACTCCTATGAACATGGAAAATATACCGGATTAGTCAATCCGACTTGAAATTCTTAAGTCGTTCATAACTGTTTAGTTAAAGTAAGAATTGCTTTTGATCGAACCATCTAGTTTCCTTTCAAGATTAATCAATTAGAATCCATTTTTGTTTTTTTATTTTTTTTTAGTTTATAGTTAAAACTAACTATTGCTCTTATGCAAATTCTCTTGTTTTCATCTCAATCTTACCGAGGATTCTCTCTAAAGAAAAGGGATTCCAAATAGAATTCTCATTTTTTTCTATTTTTTATTAGTTATAGTTAAGTTATTAGGAATTGGTCAAAATTGACATCGATTTGCATTATCTTTTCTATTTTATTATCNNNNNNNNNNNNNNTTTGTTTATATTTATTTCAATTTTTTATGAATATGTCCATTTGCCTCTTTTTTTAGTGGTTTAGAATAGAACAAGTAATAAAACGTCTAGGAATTTTAATATCAAGTCAAGATTGATTTATCAAGTCAAGATTGATTTATAGTATAGAATTGGTATATTNNNNNNNNNNNNNNNNNNNNCCTTTTATTTATTATTTATACCTTTTATTTATTNNNNNNNNNNNNATTTAATTAGAATCCAATCTCGAGGGAGGATTTTCTATCGAAAGAAAAGAGAAGACTAGGTCTAAGTAAGGTATACGAAGGAAAGCCCCTTTTACGTTGTTGACAATGTTTACAATGAAACTTAAATTAGCAAATTGGTTTTTAAAGCTTTATCTTGTGCACAACTACCGCACAATACCTGCGTAAATTACTATTAGATTTGATTGGGGTTGGGTTTTTTTAACCAGACTCGGGTCATCATACAGAATTCACTCGAATCGGATATACCAAGGGTAGTATAATTAACTCGTTGATTCGATCTTTTCTCTCCTTTC

089_20180717_01_03_ndhCret-trnV-comp AATACCAAAATCGGAATAAGACTTGATATTATCAGAAATGCCCAAAAAATATCATATTCGTAAAGCAGAAACATAGATGCACTCCTATGAACATGGAAAATATACCGGATTAGTCAATCCGACTTGAAATTCTTAAGTCGTTCATAACTGTTTAGTTAAAGTAAGAATTGCTTTTGATCGAACCATCTAGTTTCCTTTCAAGATTAATCAATTAGAATCCATTTTTGTTTTTTTATTTTTTTTTAGTTTATAGTTAAAACTAACTATTGCTCTTATGCAAATTCTCTTGTTTTCATCTCAATCTTACCGAGGATTCTCTCTAAAGAAAAGGGATTCCAAATAGAATTCTCATTTTTTTCTATTTTTTATTAGTTATAGTTAAGTTATTAGGAATTGGTCAAAATTGACATCGATTTGCATTATCTTTTCTATTTTATTATCNNNNNNNNNNNNNNTTTGTTTATATTTATTTCAATTTTTTATGAATATGTCCATTTGCCTCTTTTTTTAGTGGTTTAGAATAGAACAAGTAATAAAACGTCTAGGAATTTTAATATCAAGTCAAGATTGATTTATCAAGTCAAGATTGATTTATAGTATAGAATTGGTATATTNNNNNNNNNNNNNNNNNNNNCCTTTTATTTATTATTTATACCTTTTATTTATTNNNNNNNNNNNNATTTAATTAGAATCCAATCTCGAGGGAGGATTTTCTATCGAAAGAAAAGAGAAGACTAGGTCTAAGTAAGGTATACGAAGGAAAGCCCCTTTTACGTTGTTGACAATGTTTACAATGAAACTTAAATTAGCAAATTGGTTTTTAAAGCTTTATCTTGTGCACAACTACCGCACAATACCTGCGTAAATTACTATTAGATTTGATTGGGGTTGGGTTTTTTTAACCAGACTCGGGTCATCATACAGAATTCACTCGAATCGGATATACCAAGGGTAGTATAATTAACTCGTTGATTCGATCTTTTCTCTCCTTTC

090_20180717_01_04_ndhCret-trnV-comp AATACCAAAATCGGAATAAGACTTGATATTATCAGAAATGCCCAAAAAATATCATATTCGTAAAGCAGAAACATAGATGCACTCCTATGAACATGGAAAATATACCGGATTAGTCAATCCGACTTGAAATTCTTAAGTCGTTCATAACTGTTTAGTTAAAGTAAGAATTGCTTTTGATCGAACCATCTAGTTTCCTTTCAAGATTAATCAATTAGAATCCATTTTTGTTTTTTTATTTTTTTTTAGTTTATAGTTAAAACTAACTATTGCTCTTATGCAAATTCTCTTGTTTTCATCTCAATCTTACCGAGGATTCTCTCTAAAGAAAAGGGATTCCAAATAGAATTCTCATTTTTTTCTATTTTTTATTAGTTATAGTTAAGTTATTAGGAATTGGTCAAAATTGACATCGATTTGCATTATCTTTTCTATTTTATTATCNNNNNNNNNNNNNNTTTGTTTATATTTATTTCAATTTTTTATGAATATGTCCATTTGCCTCTTTTTTTAGTGGTTTAGAATAGAACAAGTAATAAAACGTCTAGGAATTTTAATATCAAGTCAAGATTGATTTATCAAGTCAAGATTGATTTATAGTATAGAATTGGTATATTNNNNNNNNNNNNNNNNNNNNCCTTTTATTTATTATTTATACCTTTTATTTATTNNNNNNNNNNNNATTTAATTAGAATCCAATCTCGAGGGAGGATTTTCTATCGAAAGAAAAGAGAAGACTAGGTCTAAGTAAGGTATACGAAGGAAAGCCCCTTTTACGTTGTTGACAATGTTTACAATGAAACTTAAATTAGCAAATTGGTTTTTAAAGCTTTATCTTGTGCACAACTACCGCACAATACCTGCGTAAATTACTATTAGATTTGATTGGGGTTGGGTTTTTTTAACCAGACTCGGGTCATCATACAGAATTCACTCGAATCGGATATACCAAGGGTAGTATAATTAACTCGTTGATTCGATCTTTTCTCTCCTTTC

091_20180717_01_05_ndhCret-trnV-comp AATACCAAAATCGGAATAAGACTTGATATTATCAGAAATGCCCAAAAAATATCATATTCGTAAAGCAGAAACATAGATGCACTCCTATGAACATGGAAAATATACCGGATTAGTCAATCCGACTTGAAATTCTTAAGTCGTTCATAACTGTTTAGTTAAAGTAAGAATTGCTTTTGATCGAACCATCTAGTTTCCTTTCAAGATTAATCAATTAGAATCCATTTTTGTTTTTTTATTTTTTTTTAGTTTATAGTTAAAACTAACTATTGCTCTTATGCAAATTCTCTTGTTTTCATCTCAATCTTACCGAGGATTCTCTCTAAAGAAAAGGGATTCCAAATAGAATTCTCATTTTTTTCTATTTTTTATTAGTTATAGTTAAGTTATTAGGAATTGGTCAAAATTGACATCGATTTGCATTATCTTTTCTATTTTATTATCNNNNNNNNNNNNNNTTTGTTTATATTTATTTCAATTTTTTATGAATATGTCCATTTGCCTCTTTTTTTAGTGGTTTAGAATAGAACAAGTAATAAAACGTCTAGGAATTTTAATATCAAGTCAAGATTGATTTATCAAGTCAAGATTGATTTATAGTATAGAATTGGTATATTNNNNNNNNNNNNNNNNNNNNCCTTTTATTTATTATTTATACCTTTTATTTATTNNNNNNNNNNNNATTTAATTAGAATCCAATCTCGAGGGAGGATTTTCTATCGAAAGAAAAGAGAAGACTAGGTCTAAGTAAGGTATACGAAGGAAAGCCCCTTTTACGTTGTTGACAATGTTTACAATGAAACTTAAATTAGCAAATTGGTTTTTAAAGCTTTATCTTGTGCACAACTACCGCACAATACCTGCGTAAATTACTATTAGATTTGATTGGGGTTGGGTTTTTTTAACCAGACTCGGGTCATCATACAGAATTCACTCGAATCGGATATACCAAGGGTAGTATAATTAACTCGTTGATTCGATCTTTTCTCTCCTTTC

092_20180717_03_01_ndhCret-trnV-comp AATACCAAAATCGGAATAAGACTTGATATTATCAGAAATGCCCAAAAAATATCATATTCGTAAAGCAGAAACATAGATGCACTCCTATGAACATGGAAAATATACCGGATTAGTCAATCCGACTTGAAATTCTTAAGTCGTTCATAACTGTTTAGTTAAAGTAAGAATTGCTTTTGATCGAACCATCTAGTTTCCTTTCAAGATTAATCAATTAGAATCCATTTTTGTTTTTTTATTTTTTTTTAGTTTATAGTTAAAACTAACTATTGCTCTTATGCAAATTCTCTTGTTTTCATCTCAATCTTACCGAGGATTCTCTCTAAAGAAAAGGGATTCCAAATAGAATTCTCATTTTTTTCTATTTTTTATTAGTTATAGTTAAGTTATTAGGAATTGGTCAAAATTGACATCGATTTGCATTATCTTTTCTATTTTATTATCNNNNNNNNNNNNNNTTTGTTTATATTTATTTCAATTTTTTATGAATATGTCCATTTGCCTCTTTTTTTAGTGGTTTAGAATAGAACAAGTAATAAAACGTCTAGGAATTTTAATATCAAGTCAAGATTGATTTATCAAGTCAAGATTGATTTATAGTATAGAATTGGTATATTNNNNNNNNNNNNNNNNNNNNCCTTTTATTTATTATTTATACCTTTTATTTATTNNNNNNNNNNNNATTTAATTAAAATCCAATCTCGAGGGAGGATTTTCTATCGAAAGAAAAGAGAAGACTAGGTCTAAGTAAGGTATACGAAGGAAAGCCCCTTTTACGTTGTTGACAATGTTTACAATGAAACTTAAATTAGCAAATTGGTTTTTAAAGCTTTATCTTGTGCACAACTACCGCACAATACCTGCGTAAATTACTATTAGATTTGATTGGGGTTGGGTTTTTTTAACCAGACTCGGGTCATCATACAGAATTCACTCGAATCGGATATACCAAGGGTAGTATAATTAACTCGTTGATTCGATCTTTTCTCTCCTTTC

093_20180717_03_02_ndhCret-trnV-comp AATACCAAAATCGGAATAAGACTTGATATTATCAGAAATGCCCAAAAAATATCATATTCGTAAAGCAGAAACATAGATGCACTCCTATGAACATGGAAAATATACCGGATTAGTCAATCCGACTTGAAATTCTTAAGTCGTTCATAACTGTTTAGTTAAAGTAAGAATTGCTTTTGATCGAACCATCTAGTTTCCTTTCAAGATTAATCAATTAGAATCCATTTTTGTTTTTTTATTTTTTTTTAGTTTATAGTTAAAACTAACTATTGCTCTTATGCAAATTCTCTTGTTTTCATCTCAATCTTACCGAGGATTCTCTCTAAAGAAAAGGGATTCCAAATAGAATTCTCATTTTTTTCTATTTTTTATTAGTTATAGTTAAGTTATTAGGAATTGGTCAAAATTGACATCGATTTGCATTATCTTTTCTATTTTATTATCNNNNNNNNNNNNNNTTTGTTTATATTTATTTCAATTTTTTATGAATATGTCCATTTGCCTCTTTTTTTAGTGGTTTAGAATAGAACAAGTAATAAAACGTCTAGGAATTTTAATATCAAGTCAAGATTGATTTATCAAGTCAAGATTGATTTATAGTATAGAATTGGTATATTNNNNNNNNNNNNNNNNNNNNCCTTTTATTTATTATTTATACCTTTTATTTATTNNNNNNNNNNNNATTTAATTAGAATCCAATCTCGAGGGAGGATTTTCTATCGAAAGAAAAGAGAAGACTAGGTCTAAGTAAGGTATACGAAGGAAAGCCCCTTTTACGTTGTTGACAATGTTTACAATGAAACTTAAATTAGCAAATTGGTTTTTAAAGCTTTATCTTGTGCACAACTACCGCACAATACCTGCGTAAATTACTATTAGATTTGATTGGGGTTGGGTTTTTTTAACCAGACTCGGGTCATCATACAGAATTCACTCGAATCGGATATACCAAGGGTAGTATAATTAACTCGTTGATTCGATCTTTTCTCTCCTTTC

094_20180717_03_03_ndhCret-trnV-comp AATACCAAAATCGGAATAAGACTTGATATTATCAGAAATGCCCAAAAAATATCATATTCGTAAAGCAGAAACATAGATGCACTCCTATGAACATGGAAAATATACCGGATTAGTCAATCCGACTTGAAATTCTTAAGTCGTTCATAACTGTTTAGTTAAAGTAAGAATTGCTTTTGATCGAACCATCTAGTTTCCTTTCAAGATTAATCAATTAGAATCCATTTTTGTTTTTTTATTTTTTTTTAGTTTATAGTTAAAACTAACTATTGCTCTTATGCAAATTCTCTTGTTTTCATCTCAATCTTACCGAGGATTCTCTCTAAAGAAAAGGGATTCCAAATAGAATTCTCATTTTTTTCTATTTTTTATTAGTTATAGTTAAGTTATTAGGAATTGGTCAAAATTGACATCGATTTGCATTATCTTTTCTATTTTATTATCNNNNNNNNNNNNNNTTTGTTTATATTTATTTCAATTTTTTATGAATATGTCCATTTGCCTCTTTTTTTAGTGGTTTAGAATAGAACAAGTAATAAAACGTCTAGGAATTTTAATATCAAGTCAAGATTGATTTATCAAGTCAAGATTGATTTATAGTATAGAATTGGTATATTNNNNNNNNNNNNNNNNNNNNCCTTTTATTTATTATTTATACCTTTTATTTATTNNNNNNNNNNNNATTTAATTAGAATCCAATCTCGAGGGAGGATTTTCTATCGAAAGAAAAGAGAAGACTAGGTCTAAGTAAGGTATACGAAGGAAAGCCCCTTTTACGTTGTTGACAATGTTTACAATGAAACTTAAATTAGCAAATTGGTTTTTAAAGCTTTATCTTGTGCACAACTACCGCACAATACCTGCGTAAATTACTATTAGATTTGATTGGGGTTGGGTTTTTTTAACCAGACTCGGGTCATCATACAGAATTCACTCGAATCGGATATACCAAGGGTAGTATAATTAACTCGTTGATTCGATCTTTTCTCTCCTTTC

095_20180717_03_04_ndhCret-trnV-comp AATACCAAAATCGGAATAAGACTTGATATTATCAGAAATGCCCAAAAAATATCATATTCGTAAAGCAGAAACATAGATGCACTCCTATGAACATGGAAAATATACCGGATTAGTCAATCCGACTTGAAATTCTTAAGTCGTTCATAACTGTTTAGTTAAAGTAAGAATTGCTTTTGATCGAACCATCTAGTTTCCTTTCAAGATTAATCAATTAGAATCCATTTTTGTTTTTTTATTTTTTTTTAGTTTATAGTTAAAACTAACTATTGCTCTTATGCAAATTCTCTTGTTTTCATCTCAATCTTACCGAGGATTCTCTCTAAAGAAAAGGGATTCCAAATAGAATTCTCATTTTTTTCTATTTTTTATTAGTTATAGTTAAGTTATTAGGAATTGGTCAAAATTGACATCGATTTGCATTATCTTTTCTATTTTATTATCNNNNNNNNNNNNNNTTTGTTTATATTTATTTCAATTTTTTATGAATATGTCCATTTGCCTCTTTTTTTAGTGGTTTAGAATAGAACAAGTAATAAAACGTCTAGGAATTTTAATATCAAGTCAAGATTGATTTATCAAGTCAAGATTGATTTATAGTATAGAATTGGTATATTNNNNNNNNNNNNNNNNNNNNCCTTTTATTTATTATTTATACCTTTTATTTATTNNNNNNNNNNNNATTTAATTAGAATCCAATCTCGAGGGAGGATTTTCTATCGAAAGAAAAGAGAAGACTAGGTCTAAGTAAGGTATACGAAGGAAAGCCCCTTTTACGTTGTTGACAATGTTTACAATGAAACTTAAATTAGCAAATTGGTTTTTAAAGCTTTATCTTGTGCACAACTACCGCACAATACCTGCGTAAATTACTATTAGATTTGATTGGGGTTGGGTTTTTTTAACCAGACTCGGGTCATCATACAGAATTCACTCGAATCGGATATACCAAGGGTAGTATAATTAACTCGTTGATTCGATCTTTTCTCTCCTTTC

096_20180717_03_05_ndhCret-trnV-comp AATACCAAAATCGGAATAAGACTTGATATTATCAGAAATGCCCAAAAAATATCATATTCGTAAAGCAGAAACATAGATGCACTCCTATGAACATGGAAAATATACCGGATTAGTCAATCCGACTTGAAATTCTTAAGTCGTTCATAACTGTTTAGTTAAAGTAAGAATTGCTTTTGATCGAACCATCTAGTTTCCTTTCAAGATTAATCAATTAGAATCCATTTTTGTTTTTTTATTTTTTTTTAGTTTATAGTTAAAACTAACTATTGCTCTTATGCAAATTCTCTTGTTTTCATCTCAATCTTACCGAGGATTCTCTCTAAAGAAAAGGGATTCCAAATAGAATTCTCATTTTTTTCTATTTTTTATTAGTTATAGTTAAGTTATTAGGAATTGGTCAAAATTGACATCGATTTGCATTATCTTTTCTATTTTATTATCNNNNNNNNNNNNNNTTTGTTTATATTTATTTCAATTTTTTATGAATATGTCCATTTGCCTCTTTTTTTAGTGGTTTAGAATAGAACAAGTAATAAAACGTCTAGGAATTTTAATATCAAGTCAAGATTGATTTATCAAGTCAAGATTGATTTATAGTATAGAATTGGTATATTNNNNNNNNNNNNNNNNNNNNCCTTTTATTTATTATTTATACCTTTTATTTATTNNNNNNNNNNNNATTTAATTAGAATCCAATCTCGAGGGAGGATTTTCTATCGAAAGAAAAGAGAAGACTAGGTCTAAGTAAGGTATACGAAGGAAAGCCCCTTTTACGTTGTTGACAATGTTTACAATGAAACTTAAATTAGCAAATTGGTTTTTAAAGCTTTATCTTGTGCACAACTACCGCACAATACCTGCGTAAATTACTATTAGATTTGATTGGGGTTGGGTTTTTTTAACCAGACTCGGGTCATCATACAGAATTCACTCGAATCGGATATACCAAGGGTAGTATAATTAACTCGTTGATTCGATCTTTTCTCTCCTTTC

097_20180718_01_01_ndhCret-trnV-comp AATACCAAAATCGGAATAAGACTTGATATTATCAGAAATGCCCAAAAAATATCATATTCGTAAAGCAGAAACATAGATGCACTCCTATGAACATGGAAAATATACCGGATTAGTCAATCCGACTTGAAATTCTTAAGTCGTTCATAACTGTTTAGTTAAAGTAAGAATTGCTTTTGATCGAACCATCTAGTTTCCTTTCAAGATTAATCAATTAGAATCCATTTTTGTTTTTTTATTTTTTNNNAGTTTATAGTTAAAACTAACTATTGCTCTTATGCAAATTCTCTTGTTTTCATCTCAATCTTACCGAGGATTCTCTCTAAAGAAAAGGGATTCCAAATAGAATTCTCATTTTTTTCTATTTTTTATTAGTTATAGTTAAGTTATTAGGAATTGGTCAAAATTGACATCGATTTGCATTATCTTTTCTATTTTATTATCNNNNNNNNNNNNNNTTTGTTTATATTTATTTCAATTTTTTATGAATATGTCCATTTGCCTCTTTTTTTAGTGGTTTAGAATAGAACAAGTAATAAAACGTCTAGGAATTTTAANNNNNNNNNNNNNNNNNNNTATCAAGTCAAGATTGATTTATAGTATAGAATTGGTATATTNNNNNNNNNNNNNNNNNNNNCCTTTTATTTATTATTTATACCTTTTATTTATTNNNNNNNNNNNNATTTAATTAGAATCCAATCTCGAGGGAGGATTTTCTATCGAAAGAAAAGAGAAGACTAGGTCTAAGTAAGGTATACGAAGGAAAGCCCCTTTTACGTTGTTGACAATGTTTACAATGAAACTTAAATTAGCAAATTGGTTTTTAAAGCTTTATCTTGTGCACAACTACCGCACAATACCTGCGTAAATTACTATTAGATTTGATTGGGGTTGGGTTTTTTTAACCAGACTCGGGTCATCATACAGAATTCACTCGAATCGGATATACCAAGGGTAGTATAATTAACTCGTTGATTCGATCTTTTCTCTCTTTTC

098_20180718_01_02_ndhCret-trnV-comp AATACCAAAATCGGAATAAGACTTGATATTATCAGAAATGCCCAAAAAATATCATATTCGTAAAGCAGAAACATAGATGCACTCCTATGAACATGGAAAATATACCGGATTAGTCAATCCGACTTGAAATTCTTAAGTCGTTCATAACTGTTTAGTTAAAGTAAGAATTGCTTTTGATCGAACCATCTAGTTTCCTTTCAAGATTAATCAATTAGAATCCATTTTTGTTTTTTTATTTTTTNNNAGTTTATAGTTAAAACTAACTATTGCTCTTATGCAAATTCTCTTGTTTTCATCTCAATCTTACCGAGGATTCTCTCTAAAGAAAAGGGATTCCAAATAGAATTCTCATTTTTTTCTATTTTTTATTAGTTATAGTTAAGTTATTAGGAATTGGTCAAAATTGACATCGATTTGCATTATCTTTTCTATTTTATTATCNNNNNNNNNNNNNNTTTGTTTATATTTATTTCAATTTTTTATGAATATGTCCATTTGCCTCTTTTTTTAGTGGTTTAGAATAGAACAAGTAATAAAACGTCTAGGAATTTTAANNNNNNNNNNNNNNNNNNNTATCAAGTCAAGATTGATTTATAGTATAGAATTGGTATATTNNNNNNNNNNNNNNNNNNNNCCTTTTATTTATTATTTATACCTTTTATTTATTNNNNNNNNNNNNATTTAATTAGAATCCAATCTCGAGGGAGGATTTTCTATCGAAAGAAAAGAGAAGACTAGGTCTAAGTAAGGTATACGAAGGAAAGCCCCTTTTACGTTGTTGACAATGTTTACAATGAAACTTAAATTAGCAAATTGGTTTTTAAAGCTTTATCTTGTGCACAACTACCGCACAATACCTGCGTAAATTACTATTAGATTTGATTGGGGTTGGGTTTTTTTAACCAGACTCGGGTCATCATACAGAATTCACTCGAATCGGATATACCAAGGGTAGTATAATTAACTCGTTGATTCGATCTTTTCTCTCTTTTC

099_20180718_01_03_ndhCret-trnV-comp AATACCAAAATCGGAATAAGACTTGATATTATCAGAAATGCCCAAAAAATATCATATTCGTAAAGCAGAAACATAGATGCACTCCTATGAACATGGAAAATATACCGGATTAGTCAATCCGACTTGAAATTCTTAAGTCGTTCATAACTGTTTAGTTAAAGTAAGAATTGCTTTTGATCGAACCATCTAGTTTCCTTTCAAGATTAATCAATTAGAATCCATTTTTGTTTTTTTATTTTTTNNNAGTTTATAGTTAAAACTAACTATTGCTCTTATGCAAATTCTCTTGTTTTCATCTCAATCTTACCGAGGATTCTCTCTAAAGAAAAGGGATTCCAAATAGAATTCTCATTTTTTTCTATTTTTTATTAGTTATAGTTAAGTTATTAGGAATTGGTCAAAATTGACATCGATTTGCATTATCTTTTCTATTTTATTATCNNNNNNNNNNNNNNTTTGTTTATATTTATTTCAATTTTTTATGAATATGTCCATTTGCCTCTTTTTTTAGTGGTTTAGAATAGAACAAGTAATAAAACGTCTAGGAATTTTAANNNNNNNNNNNNNNNNNNNTATCAAGTCAAGATTGATTTATAGTATAGAATTGGTATATTNNNNNNNNNNNNNNNNNNNNCCTTTTATTTATTATTTATACCTTTTATTTATTNNNNNNNNNNNNATTTAATTAGAATCCAATCTCGAGGGAGGATTTTCTATCGAAAGAAAAGAGAAGACTAGGTCTAAGTAAGGTATACGAAGGAAAGCCCCTTTTACGTTGTTGACAATGTTTACAATGAAACTTAAATTAGCAAATTGGTTTTTAAAGCTTTATCTTGTGCACAACTACCGCACAATACCTGCGTAAATTACTATTAGATTTGATTGGGGTTGGGTTTTTTTAACCAGACTCGGGTCATCATACAGAATTCACTCGAATCGGATATACCAAGGGTAGTATAATTAACTCGTTGATTCGATCTTTTCTCTCTTTTC

100_20180718_01_04_ndhCret-trnV-comp AATACCAAAATCGGAATAAGACTTGATATTATCAGAAATGCCCAAAAAATATCATATTCGTAAAGCAGAAACATAGATGCACTCCTATGAACATGGAAAATATACCGGATTAGTCAATCCGACTTGAAATTCTTAAGTCGTTCATAACTGTTTAGTTAAAGTAAGAATTGCTTTTGATCGAACCATCTAGTTTCCTTTCAAGATTAATCAATTAGAATCCATTTTTGTTTTTTTATTTTTTNNNAGTTTATAGTTAAAACTAACTATTGCTCTTATGCAAATTCTCTTGTTTTCATCTCAATCTTACCGAGGATTCTCTCTAAAGAAAAGGGATTCCAAATAGAATTCTCATTTTTTTCTATTTTTTATTAGTTATAGTTAAGTTATTAGGAATTGGTCAAAATTGACATCGATTTGCATTATCTTTTCTATTTTATTATCNNNNNNNNNNNNNNTTTGTTTATATTTATTTCAATTTTTTATGAATATGTCCATTTGCCTCTTTTTTTAGTGGTTTAGAATAGAACAAGTAATAAAACGTCTAGGAATTTTAANNNNNNNNNNNNNNNNNNNTATCAAGTCAAGATTGATTTATAGTATAGAATTGGTATATTNNNNNNNNNNNNNNNNNNNNCCTTTTATTTATTATTTATACCTTTTATTTATTNNNNNNNNNNNNATTTAATTAGAATCCAATCTCGAGGGAGGATTTTCTATCGAAAGAAAAGAGAAGACTAGGTCTAAGTAAGGTATACGAAGGAAAGCCCCTTTTACGTTGTTGACAATGTTTACAATGAAACTTAAATTAGCAAATTGGTTTTTAAAGCTTTATCTTGTGCACAACTACCGCACAATACCTGCGTAAATTACTATTAGATTTGATTGGGGTTGGGTTTTTTTAACCAGACTCGGGTCATCATACAGAATTCACTCGAATCGGATATACCAAGGGTAGTATAATTAACTCGTTGATTCGATCTTTTCTCTCTTTTC

101_20180718_01_05_ndhCret-trnV-comp AATACCAAAATCGGAATAAGACTTGATATTATCAGAAATGCCCAAAAAATATCATATTCGTAAAGCAGAAACATAGATGCACTCCTATGAACATGGAAAATATACCGGATTAGTCAATCCGACTTGAAATTCTTAAGTCGTTCATAACTGTTTAGTTAAAGTAAGAATTGCTTTTGATCGAACCATCTAGTTTCCTTTCAAGATTAATCAATTAGAATCCATTTTTGTTTTTTTATTTTTTNNNAGTTTATAGTTAAAACTAACTATTGCTCTTATGCAAATTCTCTTGTTTTCATCTCAATCTTACCGAGGATTCTCTCTAAAGAAAAGGGATTCCAAATAGAATTCTCATTTTTTTCTATTTTTTATTAGTTATAGTTAAGTTATTAGGAATTGGTCAAAATTGACATCGATTTGCATTATCTTTTCTATTTTATTATCNNNNNNNNNNNNNNTTTGTTTATATTTATTTCAATTTTTTATGAATATGTCCATTTGCCTCTTTTTTTAGTGGTTTAGAATAGAACAAGTAATAAAACGTCTAGGAATTTTAANNNNNNNNNNNNNNNNNNNTATCAAGTCAAGATTGATTTATAGTATAGAATTGGTATATTNNNNNNNNNNNNNNNNNNNNCCTTTTATTTATTATTTATACCTTTTATTTATTNNNNNNNNNNNNATTTAATTAGAATCCAATCTCGAGGGAGGATTTTCTATCGAAAGAAAAGAGAAGACTAGGTCTAAGTAAGGTATACGAAGGAAAGCCCCTTTTACGTTGTTGACAATGTTTACAATGAAACTTAAATTAGCAAATTGGTTTTTAAAGCTTTATCTTGTGCACAACTACCGCACAATACCTGCGTAAATTACTATTAGATTTGATTGGGGTTGGGTTTTTTTAACCAGACTCGGGTCATCATACAGAATTCACTCGAATCGGATATACCAAGGGTAGTATAATTAACTCGTTGATTCGATCTTTTCTCTCTTTTC

102_20180718_03_02_ndhCret-trnV-comp AATACCAAAATCGGAATAAGACTTGATATTATCAGAAATGCCCAAAAAATATCATATTCGTAAAGCAGAAACATAGATGCACTCCTATGAACATGGAAAATATACCGGATTAGTCAATCCGACTTGAAATTCTTAAGTCGTTCATAACTGTTTAGTTAAAGTAAGAATTGCTTTTGATCGAACCATCTAGTTTCCTTTCAAGATTAATCAATTAGAATCCATTTTTGTTTTTTTATTTTTTNNNAGTTTATAGTTAAAACTAACTATTGCTCTTATGCAAATTCTCTTGTTTTCATCTCAATCTTACCGAGGATTCTCTCTAAAGAAAAGGGATTCCAAATAGAATTCTCATTTTTTTCTATTTTTTATTAGTTATAGTTAAGTTATTAGGAATTGGTCAAAATTGACATCGATTTGCATTATCTTTTCTATTTTATTATCNNNNNNNNNNNNNNTTTGTTTATATTTATTTCAATTTTTTATGAATATGTCCATTTGCCTCTTTTTTTAGTGGTTTAGAATAGAACAAGTAATAAAACGTCTAGGAATTTTAANNNNNNNNNNNNNNNNNNNTATCAAGTCAAGATTGATTTATAGTATAGAATTGGTATATTNNNNNNNNNNNNNNNNNNNNCCTTTTATTTATTATTTATACCTTTTATTTATTNNNNNNNNNNNNATTTAATTAGAATCCAATCTCGAGGGAGGATTTTCTATCGAAAGAAAAGAGAAGACTAGGTCTAAGTAAGGTATACGAAGGAAAGCCCCTTTTACGTTGTTGACAATGTTTACAATGAAACTTAAATTAGCAAATTGGTTTTTAAAGCTTTATCTTGTGCACAACTACCGCACAATACCTGCGTAAATTACTATTAGATTTGATTGGGGTTGGGTTTTTTTAACCAGACTCGGGTCATCATACAGAATTCACTCGAATCGGATATACCAAGGGTAGTATAATTAACTCGTTGATTCGATCTTTTCTCTCTTTTC

103_20180718_03_03_ndhCret-trnV-comp AATACCAAAATCGGAATAAGACTTGATATTATCAGAAATGCCCAAAAAATATCATATTCGTAAAGCAGAAACATAGATGCACTCCTATGAACATGGAAAATATACCGGATTAGTCAATCCGACTTGAAATTCTTAAGTCGTTCATAACTGTTTAGTTAAAGTAAGAATTGCTTTTGATCGAACCATCTAGTTTCCTTTCAAGATTAATCAATTAGAATCCATTTTTGTTTTTTTATTTTTTTTTAGTTTATAGTTAAAACTAACTATTGCTCTTATGCAAATTCTCTTGTTTTCATCTCAATCTTACCGAGGATTCTCTCTAAAGAAAAGGGATTCCAAATAGAATTCTCATTTTTTTCTATTTTTTATTAGTTATAGTTAAGTTATTAGGAATTGGTCAAAATTGACATCGATTTGCATTATCTTTTCTATTTTATTATCNNNNNNNNNNNNNNTTTGTTTATATTTATTTCAATTTTTTATGAATATGTCCATTTGCCTCTTTTTTTAGTGGTTTAGAATAGAACAAGTAATAAAACGTCTAGGAATTTTAANNNNNNNNNNNNNNNNNNNTATCAAGTCAAGATTGATTTATAGTATAGAATTGGTATATTNNNNNNNNNNNNNNNNNNNNCCTTTTATTTATTATTTATACCTTTTATTTATTNNNNNNNNNNNNATTTAATTAGAATCCAATCTCGAGGGAGGATTTTCTATCGAAAGAAAAGAGAAGACTAGGTCTAAGTAAGGTATACGAAGGAAAGCCCCTTTTACGTTGTTGACAATGTTTACAATGAAACTTAAATTAGCAAATTGGTTTTTAAAGCTTTATCTTGTGCACAACTACCGCACAATACCTGCGTAAATTACTATTAGATTTGATTGGGGTTGGGTTTTTTTAACCAGACTCGGGTCATCATACAGAATTCACTCGAATCGGATATACCAAGGGTAGTATAATTAACTCGTTGATTCGATCTTTTCTCTCCTTTC

104_20180718_03_04_ndhCret-trnV-comp AATACCAAAATCGGAATAAGACTTGATATTATCAGAAATGCCCAAAAAATATCATATTCGTAAAGCAGAAACATAGATGCACTCCTATGAACATGGAAAATATACCGGATTAGTCAATCCGACTTGAAATTCTTAAGTCGTTCATAACTGTTTAGTTAAAGTAAGAATTGCTTTTGATCGAACCATCTAGTTTCCTTTCAAGATTAATCAATTAGAATCCATTTTTGTTTTTTTATTTTTTNNNAGTTTATAGTTAAAACTAACTATTGCTCTTATGCAAATTCTCTTGTTTTCATCTCAATCTTACCGAGGATTCTCTCTAAAGAAAAGGGATTCCAAATAGAATTCTCATTTTTTTCTATTTTTTATTAGTTATAGTTAAGTTATTAGGAATTGGTCAAAATTGACATCGATTTGCATTATCTTTTCTATTTTATTATCNNNNNNNNNNNNNNTTTGTTTATATTTATTTCAATTTTTTATGAATATGTCCATTTGCCTCTTTTTTTAGTGGTTTAGAATAGAACAAGTAATAAAACGTCTAGGAATTTTAANNNNNNNNNNNNNNNNNNNTATCAAGTCAAGATTGATTTATAGTATAGAATTGGTATATTNNNNNNNNNNNNNNNNNNNNCCTTTTATTTATTATTTATACCTTTTATTTATTNNNNNNNNNNNNATTTAATTAGAATCCAATCTCGAGGGAGGATTTTCTATCGAAAGAAAAGAGAAGACTAGGTCTAAGTAAGGTATACGAAGGAAAGCCCCTTTTACGTTGTTGACAATGTTTACAATGAAACTTAAATTAGCAAATTGGTTTTTAAAGCTTTATCTTGTGCACAACTACCGCACAATACCTGCGTAAATTACTATTAGATTTGATTGGGGTTGGGTTTTTTTAACCAGACTCGGGTCATCATACAGAATTCACTCGAATCGGATATACCAAGGGTAGTATAATTAACTCGTTGATTCGATCTTTTCTCTCTTTTC

105_20180718_03_05_ndhCret-trnV-comp AATACCAAAATCGGAATAAGACTTGATATTATCAGAAATGCCCAAAAAATATCATATTCGTAAAGCAGAAACATAGATGCACTCCTATGAACATGGAAAATATACCGGATTAGTCAATCCGACTTGAAATTCTTAAGTCGTTCATAACTGTTTAGTTAAAGTAAGAATTGCTTTTGATCGAACCATCTAGTTTCCTTTCAAGATTAATCAATTAGAATCCATTTTTGTTTTTTTATTTTTTTTTAGTTTATAGTTAAAACTAACTATTGCTCTTATGCAAATTCTCTTGTTTTCATCTCAATCTTACCGAGGATTCTCTCTAAAGAAAAGGGATTCCAAATAGAATTCTCATTTTTTTCTATTTTTTATTAGTTATAGTTAAGTTATTAGGAATTGGTCAAAATTGACATCGATTTGCATTATCTTTTCTATTTTATTATCNNNNNNNNNNNNNNTTTGTTTATATTTATTTCAATTTTTTATGAATATGTCCATTTGCCTCTTTTTTTAGTGGTTTAGAATAGAACAAGTAATAAAACGTCTAGGAATTTTAANNNNNNNNNNNNNNNNNNNTATCAAGTCAAGATTGATTTATAGTATAGAATTGGTATATTNNNNNNNNNNNNNNNNNNNNCCTTTTATTTATTATTTATACCTTTTATTTATTNNNNNNNNNNNNATTTAATTAGAATCCAATCTCGAGGGAGGATTTTCTATCGAAAGAAAAGAGAAGACTAGGTCTAAGTAAGGTATACGAAGGAAAGCCCCTTTTACGTTGTTGACAATGTTTACAATGAAACTTAAATTAGCAAATTGGTTTTTAAAGCTTTATCTTGTGCACAACTACCGCACAATACCTGCGTAAATTACTATTAGATTTGATTGGGGTTGGGTTTTTTTAACCAGACTCGGGTCATCATACAGAATTCACTCGAATCGGATATACCAAGGGTAGTATAATTAACTCGTTGATTCGATCTTTTCTCTCCTTTC

106_20180718_03_06_ndhCret-trnV-comp AATACCAAAATCGGAATAAGACTTGATATTATCAGAAATGCCCAAAAAATATCATATTCGTAAAGCAGAAACATAGATGCACTCCTATGAACATGGAAAATATACCGGATTAGTCAATCCGACTTGAAATTATTAAGTCGTTCATAACTGTTTAGTTAAAGTAAGAATTGCTTTTGATCGAACCATCTAGTTTCCTTTCAAGATTAATCAATTAGAATCCATTTTTGTTTTTTTATTTTTTTTTAGTTTATAGTTAAAACTAACTATTGCTCTTATGCAAATTCTCTTGTTTTCATCTCAATCTTACCGAGGATTCTCTCTAAAGAAAAGGGATTCCAAATAGAATTCTCATTTTTTTCTATTTTTTATTAGTTATAGTTAAGTTATTAGGAATTGGTCAAAATTGACATCGATTTGCATTATCTTTTCTATTTTATTATCNNNNNNNNNNNNNNTTTGTTTATATTTATTTCAATTTTTTATGAATATGTCCATTTGCCTCTTTTTTTAGTGGTTTAGAATAGAACAAGTAATAAAACGTCTAGGAATTTTAANNNNNNNNNNNNNNNNNNNTATCAAGTCAAGATTGATTTATAGTATAGAATTGGTATATTNNNNNNNNNNNNNNNNNNNNCCTTTTATTTATTATTTATACCTTTTATTTATTNNNNNNNNNNNNATTTAATTAGAATCCAATCTCGAGGGAGGATTTTCTATCGAAAGAAAAGAGAAGACTAGGTCTAAGTAAGGTATACGAAGGAAAGCCCCTTTTACGTTGTTGACAATGTTTACAATGAAACTTAAATTAGCAAATTGGTTTTTAAAGCTTTATCTTGTGCACAACTACCGCACAATACCTGCGTAAATTACTATTAGATTTGATTGGGGTTGGGTTTTTTTAACCAGACTCGGGTCATCATACAGAATTCACTCGAATCGGATATACCAAGGGTAGTATAATTAACTCGTTGATTCGATCTTTTCTCTCCTTTC

107_20180718_06_01_ndhCret-trnV-comp AATACCAAAATCGGAATAAGACTTGATATTATCAGAAATGCCCAAAAAATATCATATTCGTAAAGCAGAAACATAGATGCACTCCTATGAACATGGAAAATATACCGGATTAGTCAATCCGACTTGAAATTCTTAAGTCGTTCATAACTGTTTAGTTAAAGTAAGAATTGCTTTTGATCGAACCATCTAGTTTCCTTTCAAGATTAATCAATTAGAATCCATTTTTGTTTTTTTATTTTTTTTTAGTTTATAGTTAAAACTAACTATTGCTCTTATGCAAATTCTCTTGTTTTCATCTCAATCTTACCGAGGATTCTCTCTAAAGAAAAGGGATTCCAAATAGAATTCTCATTTTTTTCTATTTTTTATTAGTTATAGTTAAGTTATTAGGAATTGGTCAAAATTGACATCGATTTGCATTATCTTTTCTATTTTATTATCNNNNNNNNNNNNNNTTTGTTTATATTTATTTCAATTTTTTATGAATATGTCCATTTGCCTCTTTTTTTAGTGGTTTAGAATAGAACAAGTAATAAAACGTCTAGGAATTTTAANNNNNNNNNNNNNNNNNNNTATCAAGTCAAGATTGATTTATAGTATAGAATTGGTATATTNNNNNNNNNNNNNNNNNNNNCCTTTTATTTATTATTTATACCTTTTATTTATTNNNNNNNNNNNNATTTAATTAGAATCCAATCTCGAGGGAGGATTTTCTATCGAAAGAAAAGAGAAGACTAGGTCTAAGTAAGGTATACGAAGGAAAGCCCCTTTTACGTTGTTGACAATGTTTACAATGAAACTTAAATTAGCAAATTGGTTTTTAAAGCTTTATCTTGTGCACAACTACCGCACAATACCTGCGTAAATTACTATTAGATTTGATTGGGGTTGGGTTTTTTTAACCAGACTCGGGTCATCATACAGAATTCACTCGAATCGGATATACCAAGGGTAGTATAATTAACTCGTTGATTCGATCTTTTCTCTCCTTTC

108_20180718_06_02_ndhCret-trnV-comp AATACCAAAATCGGAATAAGACTTGATATTATCAGAAATGCCCAAAAAATATCATATTCGTAAAGCAGAAACATAGATGCACTCCTATGAACATGGAAAATATACCGGATTAGTCAATCCGACTTGAAATTCTTAAGTCGTTCATAACTGTTTAGTTAAAGTAAGAATTGCTTTTGATCGAACCATCTAGTTTCCTTTCAAGATTAATCAATTAGAATCCATTTTTGTTTTTTTATTTTTTTTTAGTTTATAGTTAAAACTAACTATTGCTCTTATGCAAATTCTCTTGTTTTCATCTCAATCTTACCGAGGATTCTCTCTAAAGAAAAGGGATTCCAAATAGAATTCTCATTTTTTTCTATTTTTTATTAGTTATAGTTAAGTTATTAGGAATTGGTCAAAATTGACATCGATTTGCATTATCTTTTCTATTTTATTATCNNNNNNNNNNNNNNTTTGTTTATATTTATTTCAATTTTTTATGAATATGTCCATTTGCCTCTTTTTTTAGTGGTTTAGAATAGAACAAGTAATAAAACGTCTAGGAATTTTAATATCAAGTCAAGATTGATTTATCAAGTCAAGATTGATTTATAGTATAGAATTGGTATATTNNNNNNNNNNNNNNNNNNNNCCTTTTATTTATTATTTATACCTTTTATTTATTNNNNNNNNNNNNATTTAATTAGAATCCAATCTCGAGGGAGGATTTTCTATCGAAAGAAAAGAGAAGACTAGGTCTAAGTAAGGTATACGAAGGAAAGCCCCTTTTACGTTGTTGACAATGTTTACAATGAAACTTAAATTAGCAAATTGGTTTTTAAAGCTTTATCTTGTGCACAACTACCGCACAATACCTGCGTAAATTACTATTAGATTTGATTGGGGTTGGGTTTTTTTAACCAGACTCGGGTCATCATACAGAATTCACTCGAATCGGATATACCAAGGGTAGTATAATTAACTCGTTGATTCGATCTTTTTTCTCCTTTC

109_20180718_06_03_ndhCret-trnV-comp AATACCAAAATCGGAATAAGACTTGATATTATCAGAAATGCCCAAAAAATATCATATTCGTAAAGCAGAAACATAGATGCACTCCTATGAACATGGAAAATATACCGGATTAGTCAATCCGACTTGAAATTCTTAAGTCGTTCATAACTGTTTAGTTAAAGTAAGAATTGCTTTTGATCGAACCATCTAGTTTCCTTTCAAGATTAATCAATTAGAATCCATTTTTGTTTTTTTATTTTTTTTTAGTTTATAGTTAAAACTAACTATTGCTCTTATGCAAATTCTCTTGTTTTCATCTCAATCTTACCGAGGATTCTCTCTAAAGAAAAGGGATTCCAAATAGAATTCTCATTTTTTTCTATTTTTTATTAGTTATAGTTAAGTTATTAGGAATTGGTCAAAATTGACATCGATTTGCATTATCTTTTCTATTTTATTATCNNNNNNNNNNNNNNTTTGTTTATATTTATTTCAATTTTTTATGAATATGTCCATTTGCCTCTTTTTTTAGTGGTTTAGAATAGAACAAGTAATAAAACGTCTAGGAATTTTAANNNNNNNNNNNNNNNNNNNTATCAAGTCAAGATTGATTTATAGTATAGAATTGGTATATTNNNNNNNNNNNNNNNNNNNNCCTTTTATTTATTATTTATACCTTTTATTTATTNNNNNNNNNNNNATTTAATTAGAATCCAATCTCGAGGGAGGATTTTCTATCGAAAGAAAAGAGAAGACTAGGTCTAAGTAAGGTATACGAAGGAAAGCCCCTTTTACGTTGTTGACAATGTTTACAATGAAACTTAAATTAGCAAATTGGTTTTTAAAGCTTTATCTTGTGCACAACTACCGCACAATACCTGCGTAAATTACTATTAGATTTGATTGGGGTTGGGTTTTTTTAACCAGACTCGGGTCATCATACAGAATTCACTCGAATCGGATATACCAAGGGTAGTATAATTAACTCGTTGATTCGATCTTTTCTCTCCTTTC

110_20180718_06_04_ndhCret-trnV-comp AATACCAAAATCGGAATAAGACTTGATATTATCAGAAATGCCCAAAAAATATCATATTCGTAAAGCAGAAACATAGATGCACTCCTATGAACATGGAAAATATACCGGATTAGTCAATCCGACTTGAAATTCTTAAGTCGTTCATAACTGTTTAGTTAAAGTAAGAATTGCTTTTGATCGAACCATCTAGTTTCCTTTCAAGATTAATCAATTAGAATCCATTTTTGTTTTTTTATTTTTTTTTAGTTTATAGTTAAAACTAACTATTGCTCTTATGCAAATTCTCTTGTTTTCATCTCAATCTTACCGAGGATTCTCTCTAAAGAAAAGGGATTCCAAATAGAATTCTCATTTTTTTCTATTTTTTATTAGTTATAGTTAAGTTATTAGGAATTGGTCAAAATTGACATCGATTTGCATTATCTTTTCTATTTTATTATCNNNNNNNNNNNNNNTTTGTTTATATTTATTTCAATTTTTTATGAATATGTCCATTTGCCTCTTTTTTTAGTGGTTTAGAATAGAACAAGTAATAAAACGTCTAGGAATTTTAANNNNNNNNNNNNNNNNNNNTATCAAGTCAAGATTGATTTATAGTATAGAATTGGTATATTNNNNNNNNNNNNNNNNNNNNCCTTTTATTTATTATTTATACCTTTTATTTATTNNNNNNNNNNNNATTTAATTAGAATCCAATCTCGAGGGAGGATTTTCTATCGAAAGAAAAGAGAAGACTAGGTCTAAGTAAGGTATACGAAGGAAAGCCCCTTTTACGTTGTTGACAATGTTTACAATGAAACTTAAATTAGCAAATTGGTTTTTAAAGCTTTATCTTGTGCACAACTACCGCACAATACCTGCGTAAATTACTATTAGATTTGATTGGGGTTGGGTTTTTTTAACCAGACTCGGGTCATCATACAGAATTCACTCGAATCGGATATACCAAGGGTAGTATAATTAACTCGTTGATTCGATCTTTTCTCTCCTTTC

111_20180718_06_05_ndhCret-trnV-comp AATACCAAAATCGGAATAAGACTTGATATTATCAGAAATGCCCAAAAAATATCATATTCGTAAAGCAGAAACATAGATGCACTCCTATGAACATGGAAAATATACCGGATTAGTCAATCCGACTTGAAATTCTTAAGTCGTTCATAACTGTTTAGTTAAAGTAAGAATTGCTTTTGATCGAACCATCTAGTTTCCTTTCAAGATTAATCAATTAGAATCCATTTTTGTTTTTTTATTTTTTTTTAGTTTATAGTTAAAACTAACTATTGCTCTTATGCAAATTCTCTTGTTTTCATCTCAATCTTACCGAGGATTCTCTCTAAAGAAAAGGGATTCCAAATAGAATTCTCATTTTTTTCTATTTTTTATTAGTTATAGTTAAGTTATTAGGAATTGGTCAAAATTGACATCGATTTGCATTATCTTTTCTATTTTATTATCNNNNNNNNNNNNNNTTTGTTTATATTTATTTCAATTTTTTATGAATATGTCCATTTGCCTCTTTTTTTAGTGGTTTAGAATAGAACAAGTAATAAAACGTCTAGGAATTTTAANNNNNNNNNNNNNNNNNNNTATCAAGTCAAGATTGATTTATAGTATAGAATTGGTATATTNNNNNNNNNNNNNNNNNNNNCCTTTTATTTATTATTTATACCTTTTATTTATTNNNNNNNNNNNNATTTAATTAGAATCCAATCTCGAGGGAGGATTTTCTATCGAAAGAAAAGAGAAGACTAGGTCTAAGTAAGGTATACGAAGGAAAGCCCCTTTTACGTTGTTGACAATGTTTACAATGAAACTTAAATTAGCAAATTGGTTTTTAAAGCTTTATCTTGTGCACAACTACCGCACAATACCTGCGTAAATTACTATTAGATTTGATTGGGGTTGGGTTTTTTTAACCAGACTCGGGTCATCATACAGAATTCACTCGAATCGGATATACCAAGGGTAGTATAATTAACTCGTTGATTCGATCTTTTCTCTCCTTTC

112_20180718_08_01_ndhCret-trnV-comp AATACCAAAATCGGAATAAGACTTGATATTATCAGAAATGCCCAAAAAATATCATATTCGTAAAGCAGAAACATAGATGCACTCCTATGAACATGGAAAATATACCGGATTAGTCAATCCGACTTGAAATTCTTAAGTCGTTCATAACTGTTTAGTTAAAGTAAGAATTGCTTTTGATCGAACCATCTAGTTTCCTTTCAAGATTAATCAATTAGAATCCATTTTTGTTTTTTTATTTTTTTTTAGTTTATAGTTAAAACTAACTATTGCTCTTATGCAAATTCTCTTGTTTTCATCTCAATCTTACCGAGGATTCTCTCTAAAGAAAAGGGATTCCAAATAGAATTCTCATTTTTTTCTATTTTTTATTAGTTATAGTTAAGTTATTAGGAATTGGTCAAAATTGACATCGATTTGCATTATCTTTTCTATTTTATTATCNNNNNNNNNNNNNNTTTGTTTATATTTATTTCAATTTTTTATGAATATGTCCATTTGCCTCTTTTTTTAGTGGTTTAGAATAGAACAAGTAATAAAACGTCTAGGAATTTTAANNNNNNNNNNNNNNNNNNNTATCAAGTCAAGATTGATTTATAGTATAGAATTGGTATATTNNNNNNNNNNNNNNNNNNNNCCTTTTATTTATTATTTATACCTTTTATTTATTNNNNNNNNNNNNATTTAATTAGAATCCAATCTCGAGGGAGGATTTTCTATCGAAAGAAAAGAGAAGACTAGGTCTAAGTAAGGTATACGAAGGAAAGCCCCTTTTACGTTGTTGACAATGTTTACAATGAAACTTAAATTAGCAAATTGGTTTTTAAAGCTTTATCTTGTGCACAACTACCGCACAATACCTGCGTAAATTACTATTAGATTTGATTGGGGTTGGGTTTTTTTAACCAGACTCGGGTCATCATACAGAATTCACTCGAATCGGATATACCAAGGGTAGTATAATTAACTCGTTGATTCGATCTTTTCTCTCCTTTC

113_20180718_08_02_ndhCret-trnV-comp AATACCAAAATCGGAATAAGACTTGATATTATCAGAAATGCCCAAAAAATATCATATTCGTAAAGCAGAAACATAGATGCACTCCTATGAACATGGAAAATATACCGGATTAGTCAATCCGACTTGAAATTCTTAAGTCGTTCATAACTGTTTAGTTAAAGTAAGAATTGCTTTTGATCGAACCATCTAGTTTCCTTTCAAGATTAATCAATTAGAATCCATTTTTGTTTTTTTATTTTTTTTTAGTTTATAGTTAAAACTAACTATTGCTCTTATGCAAATTCTCTTGTTTTCATCTCAATCTTACCGAGGATTCTCTCTAAAGAAAAGGGATTCCAAATAGAATTCTCATTTTTTTCTATTTTTTATTAGTTATAGTTAAGTTATTAGGAATTGGTCAAAATTGACATCGATTTGCATTATCTTTTCTATTTTATTATCNNNNNNNNNNNNNNTTTGTTTATATTTATTTCAATTTTTTATGAATATGTCCATTTGCCTCTTTTTTTAGTGGTTTAGAATAGAACAAGTAATAAAACGTCTAGGAATTTTAANNNNNNNNNNNNNNNNNNNTATCAAGTCAAGATTGATTTATAGTATAGAATTGGTATATTNNNNNNNNNNNNNNNNNNNNCCTTTTATTTATTATTTATACCTTTTATTTATTNNNNNNNNNNNNATTTAATTAGAATCCAATCTCGAGGGAGGATTTTCTATCGAAAGAAAAGAGAAGACTAGGTCTAAGTAAGGTATACGAAGGAAAGCCCCTTTTACGTTGTTGACAATGTTTACAATGAAACTTAAATTAGCAAATTGGTTTTTAAAGCTTTATCTTGTGCACAACTACCGCACAATACCTGCGTAAATTACTATTAGATTTGATTGGGGTTGGGTTTTTTTAACCAGACTCGGGTCATCATACAGAATTCACTCGAATCGGATATACCAAGGGTAGTATAATTAACTCGTTGATTCGATCTTTTCTCTCCTTTC

114_20180718_08_03_ndhCret-trnV-comp AATACCAAAATCGGAATAAGACTTGATATTATCAGAAATGCCCAAAAAATATCATATTCGTAAAGCAGAAACATAGATGCACTCCTATGAACATGGAAAATATACCGGATTAGTCAATCCGACTTGAAATTCTTAAGTCGTTCATAACTGTTTAGTTAAAGTAAGAATTGCTTTTGATCGAACCATCTAGTTTCCTTTCAAGATTAATCAATTAGAATCCATTTTTGTTTTTTTATTTTTTTTTAGTTTATAGTTAAAACTAACTATTGCTCTTATGCAAATTCTCTTGTTTTCATCTCAATCTTACCGAGGATTCTCTCTAAAGAAAAGGGATTCCAAATAGAATTCTCATTTTTTTCTATTTTTTATTAGTTATAGTTAAGTTATTAGGAATTGGTCAAAATTGACATCGATTTGCATTATCTTTTCTATTTTATTATCNNNNNNNNNNNNNNTTTGTTTATATTTATTTCAATTTTTTATGAATATGTCCATTTGCCTCTTTTTTTAGTGGTTTAGAATAGAACAAGTAATAAAACGTCTAGGAATTTTAANNNNNNNNNNNNNNNNNNNTATCAAGTCAAGATTGATTTATAGTATAGAATTGGTATATTNNNNNNNNNNNNNNNNNNNNCCTTTTATTTATTATTTATACCTTTTATTTATTNNNNNNNNNNNNATTTAATTAGAATCCAATCTCGAGGGAGGATTTTCTATCGAAAGAAAAGAGAAGACTAGGTCTAAGTAAGGTATACGAAGGAAAGCCCCTTTTACGTTGTTGACAATGTTTACAATGAAACTTAAATTAGCAAATTGGTTTTTAAAGCTTTATCTTGTGCACAACTACCGCACAATACCTGCGTAAATTACTATTAGATTTGATTGGGGTTGGGTTTTTTTAACCAGACTCGGGTCATCATACAGAATTCACTCGAATCGGATATACCAAGGGTAGTATAATTAACTCGTTGATTCGATCTTTTCTCTCCTTTC

115_20180718_08_04_ndhCret-trnV-comp AATACCAAAATCGGAATAAGACTTGATATTATCAGAAATGCCCAAAAAATATCATATTCGTAAAGCAGAAACATAGATGCACTCCTATGAACATGGAAAATATACCGGATTAGTCAATCCGACTTGAAATTCTTAAGTCGTTCATAACTGTTTAGTTAAAGTAAGAATTGCTTTTGATCGAACCATCTAGTTTCCTTTCAAGATTAATCAATTAGAATCCATTTTTGTTTTTTTATTTTTTTTTAGTTTATAGTTAAAACTAACTATTGCTCTTATGCAAATTCTCTTGTTTTCATCTCAATCTTACCGAGGATTCTCTCTAAAGAAAAGGGATTCCAAATAGAATTCTCATTTTTTTCTATTTTTTATTAGTTATAGTTAAGTTATTAGGAATTGGTCAAAATTGACATCGATTTGCATTATCTTTTCTATTTTATTATCNNNNNNNNNNNNNNTTTGTTTATATTTATTTCAATTTTTTATGAATATGTCCATTTGCCTCTTTTTTTAGTGGTTTAGAATAGAACAAGTAATAAAACGTCTAGGAATTTTAANNNNNNNNNNNNNNNNNNNTATCAAGTCAAGATTGATTTATAGTATAGAATTGGTATATTNNNNNNNNNNNNNNNNNNNNCCTTTTATTTATTATTTATACCTTTTATTTATTNNNNNNNNNNNNATTTAATTAGAATCCAATCTCGAGGGAGGATTTTCTATCGAAAGAAAAGAGAAGACTAGGTCTAAGTAAGGTATACGAAGGAAAGCCCCTTTTACGTTGTTGACAATGTTTACAATGAAACTTAAATTAGCAAATTGGTTTTTAAAGCTTTATCTTGTGCACAACTACCGCACAATACCTGCGTAAATTACTATTAGATTTGATTGGGGTTGGGTTTTTTTAACCAGACTCGGGTCATCATACAGAATTCACTCGAATCGGATATACCAAGGGTAGTATAATTAACTCGTTGATTCGATCTTTTCTCTCCTTTC

116_20180718_08_05_ndhCret-trnV-comp AATACCAAAATCGGAATAAGACTTGATATTATCAGAAATGCCCAAAAAATATCATATTCGTAAAGCAGAAACATAGATGCACTCCTATGAACATGGAAAATATACCGGATTAGTCAATCCGACTTGAAATTCTTAAGTCGTTCATAACTGTTTAGTTAAAGTAAGAATTGCTTTTGATCGAACCATCTAGTTTCCTTTCAAGATTAATCAATTAGAATCCATTTTTGTTTTTTTATTTTTTTTTAGTTTATAGTTAAAACTAACTATTGCTCTTATGCAAATTCTCTTGTTTTCATCTCAATCTTACCGAGGATTCTCTCTAAAGAAAAGGGATTCCAAATAGAATTCTCATTTTTTTCTATTTTTTATTAGTTATAGTTAAGTTATTAGGAATTGGTCAAAATTGACATCGATTTGCATTATCTTTTCTATTTTATTATCNNNNNNNNNNNNNNTTTGTTTATATTTATTTCAATTTTTTATGAATATGTCCATTTGCCTCTTTTTTTAGTGGTTTAGAATAGAACAAGTAATAAAACGTCTAGGAATTTTAANNNNNNNNNNNNNNNNNNNTATCAAGTCAAGATTGATTTATAGTATAGAATTGGTATATTNNNNNNNNNNNNNNNNNNNNCCTTTTATTTATTATTTATACCTTTTATTTATTNNNNNNNNNNNNATTTAATTAGAATCCAATCTCGAGGGAGGATTTTCTATCGAAAGAAAAGAGAAGACTAGGTCTAAGTAAGGTATACGAAGGAAAGCCCCTTTTACGTTGTTGACAATGTTTACAATGAAACTTAAATTAGCAAATTGGTTTTTAAAGCTTTATCTTGTGCACAACTACCGCACAATACCTGCGTAAATTACTATTAGATTTGATTGGGGTTGGGTTTTTTTAACCAGACTCGGGTCATCATACAGAATTCACTCGAATCGGATATACCAAGGGTAGTATAATTAACTCGTTGATTCGATCTTTTCTCTCCTTTC

117_20180807_03_01_ndhCret-trnV-comp AATACCAAAATCGGAATAAGACTTGATATTATCAGAAATGCCCAAAAAATATCATATTCGTAAAGCAGAAACATAGATGCACTCCTATGAACATGGAAAATATACCGGATTAGTCAATCCGACTTGAAATTCTTAAGTCGTTCATAACTGTTTAGTTAAAGTAAGAATTGCTTTTGATCGAACCATCTAGTTTCCTTTCAAGATTAATCAATTAGAATCCATTTTTGTTTTTTTATTTTTTTTTAGTTTATAGTTAAAACTAACTATTGCTCTTATGCAAATTCTCTTGTTTTCATCTCAATCTTACCGAGGATTCTCTCTAAAGAAAAGGGATTCCAAATAGAATTCTCATTTTTTTCTATTTTTTATTAGTTATAGTTAAGTTATTAGGAATTGGTCAAAATTGACATCGATTTGCATTATCTTTTCTATTTTATTATCNNNNNNNNNNNNNNTTTGTTTATATTTATTTCAATTTTTTATGAATATGTCCATTTGCCTCTTTTTTTAGTGGTTTAGAATAGAACAAGTAATAAAACGTCTAGGAATTTTAATATCAAGTCAAGATTGATTTATCAAGTCAAGATTGATTTATAGTATAGAATTGGTATATTNNNNNNNNNNNNNNNNNNNNCCTTTTATTTATTATTTATACCTTTTATTTATTNNNNNNNNNNNNATTTAATTAGAATCCAATCTCGAGGGAGGATTTTCTATCGAAAGAAAAGAGAAGACTAGGTCTAAGTAAGGTATACGAAGGAAAGCCCCTTTTACGTTGTTGACAATGTTTACAATGAAACTTAAATTAGCAAATTGGTTTTTAAAGCTTTATCTTGTGCACAACTACCGCACAATACCTGCGTAAATTACTATTAGATTTGATTGGGGTTGGGTTTTTTTAACCAGACTCGGGTCATCATACAGAATTCACTCGAATCGGATAGACCAAGGGTAGTATAATTAACTCGTTGATTCGATTTTCTCTCTCCTTTC

118_20180807_03_02_ndhCret-trnV-comp AATACCAAAATCGGAATAAGACTTGATATTATCAGAAATGCCCAAAAAATATCATATTCGTAAAGCAGAAACATAGATGCACTCCTATGAACATGGAAAATATACCGGATTAGTCAATCCGACTTGAAATTCTTAAGTCGTTCATAACTGTTTAGTTAAAGTAAGAATTGCTTTTGATCGAACCATCTAGTTTCCTTTCAAGATTAATCAATTAGAATCCATTTTTGTTTTTTTATTTTTTTTTAGTTTATAGTTAAAACTAACTATTGCTCTTATGCAAATTCTCTTGTTTTCATCTCAATCTTACCGAGGATTCTCTCTAAAGAAAAGGGATTCCAAATAGAATTCTCATTTTTTTCTATTTTTTATTAGTTATAGTTAAGTTATTAGGAATTGGTCAAAATTGACATCGATTTGCATTATCTTTTCTATTTTATTATCNNNNNNNNNNNNNNTTTGTTTATATTTATTTCAATTTTTTATGAATATGTCCATTTGCCTCTTTTTTTAGTGGTTTAGAATAGAACAAGTAATAAAACGTCTAGGAATTTTAATATCAAGTCAAGATTGATTTATCAAGTCAAGATTGATTTATAGTATAGAATTGGTATATTNNNNNNNNNNNNNNNNNNNNCCTTTTATTTATTATTTATACCTTTTATTTATTNNNNNNNNNNNNATTTAATTAGAATCCAATCTCGAGGGAGGATTTTCTATCGAAAGAAAAGAGAAGACTAGGTCTAAGTAAGGTATACGAAGGAAAGCCCCTTTTACGTTGTTGACAATGTTTACAATGAAACTTAAATTAGCAAATTGGTTTTTAAAGCTTTATCTTGTGCACAACTACCGCACAATACCTGCGTAAATTACTATTAGATTTGATTGGGGTTGGGTTTTTTTAACCAGACTCGGGTCATCATACAGAATTCACTCGAATCGGATAGACCAAGGGTAGTATAATTAACTCGTTGATTCGATTTTCTCTCTCCTTTC

119_20180807_03_03_ndhCret-trnV-comp AATACCAAAATCGGAATAAGACTTGATATTATCAGAAATGCCCAAAAAATATCATATTCGTAAAGCAGAAACATAGATGCACTCCTATGAACATGGAAAATATACCGGATTAGTCAATCCGACTTGAAATTCTTAAGTCGTTCATAACTGTTTAGTTAAAGTAAGAATTGCTTTTGATCGAACCATCTAGTTTCCTTTCAAGATTAATCAATTAGAATCCATTTTTGTTTTTTTATTTTTTTTTAGTTTATAGTTAAAACTAACTATTGCTCTTATGCAAATTCTCTTGTTTTCATCTCAATCTTACCGAGGATTCTCTCTAAAGAAAAGGGATTCCAAATAGAATTCTCATTTTTTTCTATTTTTTATTAGTTATAGTTAAGTTATTAGGAATTGGTCAAAATTGACATCGATTTGCATTATCTTTTCTATTTTATTATCNNNNNNNNNNNNNNTTTGTTTATATTTATTTCAATTTTTTATGAATATGTCCATTTGCCTCTTTTTTTAGTGGTTTAGAATAGAACAAGTAATAAAACGTCTAGGAATTTTAATATCAAGTCAAGATTGATTTATCAAGTCAAGATTGATTTATAGTATAGAATTGGTATATTNNNNNNNNNNNNNNNNNNNNCCTTTTATTTATTATTTATACCTTTTATTTATTNNNNNNNNNNNNATTTAATTAGAATCCAATCTCGAGGGAGGATTTTCTATCGAAAGAAAAGAGAAGACTAGGTCTAAGTAAGGTATACGAAGGAAAGCCCCTTTTACGTTGTTGACAATGTTTACAATGAAACTTAAATTAGCAAATTGGTTTTTAAAGCTTTATCTTGTGCACAACTACCGCACAATACCTGCGTAAATTACTATTAGATTTGATTGGGGTTGGGTTTTTTTAACCAGACTCGGGTCATCATACAGAATTCACTCGAATCGGATAGACCAAGGGTAGTATAATTAACTCGTTGATTCGATTTTCTCTCTCCTTTC

120_20180807_03_04_ndhCret-trnV-comp AATACCAAAATCGGAATAAGACTTGATATTATCAGAAATGCCCAAAAAATATCATATTCGTAAAGCAGAAACATAGATGCACTCCTATGAACATGGAAAATATACCGGATTAGTCAATCCGACTTGAAATTCTTAAGTCGTTCATAACTGTTTAGTTAAAGTAAGAATTGCTTTTGATCGAACCATCTAGTTTCCTTTCAAGATTAATCAATTAGAATCCATTTTTGTTTTTTTATTTTTTTTTAGTTTATAGTTAAAACTAACTATTGCTCTTATGCAAATTCTCTTGTTTTCATCTCAATCTTACCGAGGATTCTCTCTAAAGAAAAGGGATTCCAAATAGAATTCTCATTTTTTTCTATTTTTTATTAGTTATAGTTAAGTTATTAGGAATTGGTCAAAATTGACATCGATTTGCATTATCTTTTCTATTTTATTATCNNNNNNNNNNNNNNTTTGTTTATATTTATTTCAATTTTTTATGAATATGTCCATTTGCCTCTTTTTTTAGTGGTTTAGAATAGAACAAGTAATAAAACGTCTAGGAATTTTAATATCAAGTCAAGATTGATTTATCAAGTCAAGATTGATTTATAGTATAGAATTGGTATATTNNNNNNNNNNNNNNNNNNNNCCTTTTATTTATTATTTATACCTTTTATTTATTNNNNNNNNNNNNATTTAATTAGAATCCAATCTCGAGGGAGGATTTTCTATCGAAAGAAAAGAGAAGACTAGGTCTAAGTAAGGTATACGAAGGAAAGCCCCTTTTACGTTGTTGACAATGTTTACAATGAAACTTAAATTAGCAAATTGGTTTTTAAAGCTTTATCTTGTGCACAACTACCGCACAATACCTGCGTAAATTACTATTAGATTTGATTGGGGTTGGGTTTTTTTAACCAGACTCGGGTCATCATACAGAATTCACTCGAATCGGATAGACCAAGGGTAGTATAATTAACTCGTTGATTCGATTTTCTCTCTCCTTTC

121_20180807_03_05_ndhCret-trnV-comp AATACCAAAATCGGAATAAGACTTGATATTATCAGAAATGCCCAAAAAATATCATATTCGTAAAGCAGAAACATAGATGCACTCCTATGAACATGGAAAATATACCGGATTAGTCAATCCGACTTGAAATTCTTAAGTCGTTCATAACTGTTTAGTTAAAGTAAGAATTGCTTTTGATCGAACCATCTAGTTTCCTTTCAAGATTAATCAATTAGAATCCATTTTTGTTTTTTTATTTTTTTTTAGTTTATAGTTAAAACTAACTATTGCTCTTATGCAAATTCTCTTGTTTTCATCTCAATCTTACCGAGGATTCTCTCTAAAGAAAAGGGATTCCAAATAGAATTCTCATTTTTTTCTATTTTTTATTAGTTATAGTTAAGTTATTAGGAATTGGTCAAAATTGACATCGATTTGCATTATCTTTTCTATTTTATTATCNNNNNNNNNNNNNNTTTGTTTATATTTATTTCAATTTTTTATGAATATGTCCATTTGCCTCTTTTTTTAGTGGTTTAGAATAGAACAAGTAATAAAACGTCTAGGAATTTTAATATCAAGTCAAGATTGATTTATCAAGTCAAGATTGATTTATAGTATAGAATTGGTATATTNNNNNNNNNNNNNNNNNNNNCCTTTTATTTATTATTTATACCTTTTATTTATTNNNNNNNNNNNNATTTAATTAGAATCCAATCTCGAGGGAGGATTTTCTATCGAAAGAAAAGAGAAGACTAGGTCTAAGTAAGGTATACGAAGGAAAGCCCCTTTTACGTTGTTGACAATGTTTACAATGAAACTTAAATTAGCAAATTGGTTTTTAAAGCTTTATCTTGTGCACAACTACCGCACAATACCTGCGTAAATTACTATTAGATTTGATTGGGGTTGGGTTTTTTTAACCAGACTCGGGTCATCATACAGAATTCACTCGAATCGGATAGACCAAGGGTAGTATAATTAACTCGTTGATTCGATTTTCTCTCTCCTTTC

122_20180827_01_01_ndhCret-trnV-comp AATACCAAAATCGGAATAAGACTTGATATTATCAGAAATGCCCAAAAAATATCATATTCGTAAAGCAGAAACATAGATGCACTCCTATGAACATGGAAAATATACCGGATTAGTCAATCCGACTTGAAATTCTTAAGTCGTTCATAACTGTTTAGTTAAAGTAAGAATTGCTTTTGATCGAACCATCTAGTTTCCTTTCAAGATTAATCAATTAGAATCCATTTTTGTTTTTTTATTTTTTNNNAGTTTATAGTTAAAACTAACTATTGCTCTTATGCAAATTCTCTTGTTTTCATCTCAATCTTACCGAGGATTCTCTCTAAAGAAAAGGGATTCCAAATAGAATTCTCATTTTTTTCTATTTTTTATTAGTTATAGTTAAGTTATTAGGAATTGGTCAAAATTGACATCGATTTGCATTATCTTTTCTATTTTATTATCNNNNNNNNNNNNNNTTTGTTTATATTTATTTCAATTTTTTATGAATATGTCCATTTGCCTCTTTTTTTAGTGGTTTAGAATAGAACAAGTAATAAAACGTCTAGGAATTTTAANNNNNNNNNNNNNNNNNNNTATCAAGTCAAGATTGATTTATAGTATAGAATTGGTATATTNNNNNNNNNNNNNNNNNNNNCCTTTTATTTATTATTTATACCTTTTATTTATTNNNNNNNNNNNNATTTAATTAGAATCCAATCTCGAGGGAGGATTTTCTATCGAAAGAAAAGAGAAGACTAGGTCTAAGTAAGGTATACGAAGGAAAGCCCCTTTTACGTTGTTGACAATGTTTACAATGAAACTTAAATTAGCAAATTGGTTTTTAAAGCTTTATCTTGTGCACAACTACCGCACAATACCTGCGTAAATTACTATTAGATTTGATTGGGGTTGGGTTTTTTTAACCAGACTCGGGTCATCATACAGAATTCACTCGAATCGGATATACCAAGGGTAGTATAATTAACTCGTTGATTCGATCTTTTCTCTCTTTTC

123_20180827_01_02_ndhCret-trnV-comp AATACCAAAATCGGAATAAGACTTGATATTATCAGAAATGCCCAAAAAATATCATATTCGTAAAGCAGAAACATAGATGCACTCCTATGAACATGGAAAATATACCGGATTAGTCAATCCGACTTGAAATTCTTAAGTCGTTCATAACTGTTTAGTTAAAGTAAGAATTGCTTTTGATCGAACCATCTAGTTTCCTTTCAAGATTAATCAATTAGAATCCATTTTTGTTTTTTTATTTTTTNNNAGTTTATAGTTAAAACTAACTATTGCTCTTATGCAAATTCTCTTGTTTTCATCTCAATCTTACCGAGGATTCTCTCTAAAGAAAAGGGATTCCAAATAGAATTCTCATTTTTTTCTATTTTTTATTAGTTATAGTTAAGTTATTAGGAATTGGTCAAAATTGACATCGATTTGCATTATCTTTTCTATTTTATTATCNNNNNNNNNNNNNNTTTGTTTATATTTATTTCAATTTTTTATGAATATGTCCATTTGCCTCTTTTTTTAGTGGTTTAGAATAGAACAAGTAATAAAACGTCTAGGAATTTTAANNNNNNNNNNNNNNNNNNNTATCAAGTCAAGATTGATTTATAGTATAGAATTGGTATATTNNNNNNNNNNNNNNNNNNNNCCTTTTATTTATTATTTATACCTTTTATTTATTNNNNNNNNNNNNATTTAATTAGAATCCAATCTCGAGGGAGGATTTTCTATCGAAAGAAAAGAGAAGACTAGGTCTAAGTAAGGTATACGAAGGAAAGCCCCTTTTACGTTGTTGACAATGTTTACAATGAAACTTAAATTAGCAAATTGGTTTTTAAAGCTTTATCTTGTGCACAACTACCGCACAATACCTGCGTAAATTACTATTAGATTTGATTGGGGTTGGGTTTTTTTAACCAGACTCGGGTCATCATACAGAAATCACTCGAATCGGATATACCAAGGGTAGTATAATTAACTCGTTGATTCGATCTTTTCTCTCTTTTC

124_20180827_01_03_ndhCret-trnV-comp AATACCAAAATCGGAATAAGACTTGATATTATCAGAAATGCCCAAAAAATATCATATTCGTAAAGCAGAAACATAGATGCACTCCTATGAACATGGAAAATATACCGGATTAGTCAATCCGACTTGAAATTCTTAAGTCGTTCATAACTGTTTAGTTAAAGTAAGAATTGCTTTTGATCGAACCATCTAGTTTCCTTTCAAGATTAATCAATTAGAATCCATTTTTGTTTTTTTATTTTTTNNNAGTTTATAGTTAAAACTAACTATTGCTCTTATGCAAATTCTCTTGTTTTCATCTCAATCTTACCGAGGATTCTCTCTAAAGAAAAGGGATTCCAAATAGAATTCTCATTTTTTTCTATTTTTTATTAGTTATAGTTAAGTTATTAGGAATTGGTCAAAATTGACATCGATTTGCATTATCTTTTCTATTTTATTATCNNNNNNNNNNNNNNTTTGTTTATATTTATTTCAATTTTTTATGAATATGTCCATTTGCCTCTTTTTTTAGTGGTTTAGAATAGAACAAGTAATAAAACGTCTAGGAATTTTAANNNNNNNNNNNNNNNNNNNTATCAAGTCAAGATTGATTTATAGTATAGAATTGGTATATTNNNNNNNNNNNNNNNNNNNNCCTTTTATTTATTATTTATACCTTTTATTTATTNNNNNNNNNNNNATTTAATTAGAATCCAATCTCGAGGGAGGATTTTCTATCGAAAGAAAAGAGAAGACTAGGTCTAAGTAAGGTATACGAAGGAAAGCCCCTTTTACGTTGTTGACAATGTTTACAATGAAACTTAAATTAGCAAATTGGTTTTTAAAGCTTTATCTTGTGCACAACTACCGCACAATACCTGCGTAAATTACTATTAGATTTGATTGGGGTTGGGTTTTTTTAACCAGACTCGGGTCATCATACAGAATTCACTCGAATCGGATATACCAAGGGTAGTATAATTAACTCGTTGATTCGATCTTTTCTCTCTTTTC

125_20180827_01_04_ndhCret-trnV-comp AATACCAAAATCGGAATAAGACTTGATATTATCAGAAATGCCCAAAAAATATCATATTCGTAAAGCAGAAACATAGATGCACTCCTATGAACATGGAAAATATACCGGATTAGTCAATCCGACTTGAAATTCTTAAGTCGTTCATAACTGTTTAGTTAAAGTAAGAATTGCTTTTGATCGAACCATCTAGTTTCCTTTCAAGATTAATCAATTAGAATCCATTTTTGTTTTTTTATTTTTTNNNAGTTTATAGTTAAAACTAACTATTGCTCTTATGCAAATTCTCTTGTTTTCATCTCAATCTTACCGAGGATTCTCTCTAAAGAAAAGGGATTCCAAATAGAATTCTCATTTTTTTCTATTTTTTATTAGTTATAGTTAAGTTATTAGGAATTGGTCAAAATTGACATCGATTTGCATTATCTTTTCTATTTTATTATCNNNNNNNNNNNNNNTTTGTTTATATTTATTTCAATTTTTTATGAATATGTCCATTTGCCTCTTTTTTTAGTGGTTTAGAATAGAACAAGTAATAAAACGTCTAGGAATTTTAANNNNNNNNNNNNNNNNNNNTATCAAGTCAAGATTGATTTATAGTATAGAATTGGTATATTNNNNNNNNNNNNNNNNNNNNCCTTTTATTTATTATTTATACCTTTTATTTATTNNNNNNNNNNNNATTTAATTAGAATCCAATCTCGAGGGAGGATTTTCTATCGAAAGAAAAGAGAAGACTAGGTCTAAGTAAGGTATACGAAGGAAAGCCCCTTTTACGTTGTTGACAATGTTTACAATGAAACTTAAATTAGCAAATTGGTTTTTAAAGCTTTATCTTGTGCACAACTACCGCACAATACCTGCGTAAATTACTATTAGATTTGATTGGGGTTGGGTTTTTTTAACCAGACTCGGGTCATCATACAGAATTCACTCGAATCGGATATACCAAGGGTAGTATAATTAACTCGTTGATTCGATCTTTTCTCTCTTTTC

126_20180827_01_05_ndhCret-trnV-comp AATACCAAAATCGGAATAAGACTTGATATTATCAGAAATGCCCAAAAAATATCATATTCGTAAAGCAGAAACATAGATGCACTCCTATGAACATGGAAAATATACCGGATTAGTCAATCCGACTTGAAATTCTTAAGTCGTTCATAACTGTTTAGTTAAAGTAAGAATTGCTTTTGATCGAACCATCTAGTTTCCTTTCAAGATTAATCAATTAGAATCCATTTTTGTTTTTTTATTTTTTNNNAGTTTATAGTTAAAACTAACTATTGCTCTTATGCAAATTCTCTTGTTTTCATCTCAATCTTACCGAGGATTCTCTCTAAAGAAAAGGGATTCCAAATAGAATTCTCATTTTTTTCTATTTTTTATTAGTTATAGTTAAGTTATTAGGAATTGGTCAAAATTGACATCGATTTGCATTATCTTTTCTATTTTATTATCNNNNNNNNNNNNNNTTTGTTTATATTTATTTCAATTTTTTATGAATATGTCCATTTGCCTCTTTTTTTAGTGGTTTAGAATAGAACAAGTAATAAAACGTCTAGGAATTTTAANNNNNNNNNNNNNNNNNNNTATCAAGTCAAGATTGATTTATAGTATAGAATTGGTATATTNNNNNNNNNNNNNNNNNNNNCCTTTTATTTATTATTTATACCTTTTATTTATTNNNNNNNNNNNNATTTAATTAGAATCCAATCTCGAGGGAGGATTTTCTATCGAAAGAAAAGAGAAGACTAGGTCTAAGTAAGGTATACGAAGGAAAGCCCCTTTTACGTTGTTGACAATGTTTACAATGAAACTTAAATTAGCAAATTGGTTTTTAAAGCTTTATCTTGTGCACAACTACCGCACAATACCTGCGTAAATTACTATTAGATTTGATTGGGGTTGGGTTTTTTTAACCAGACTCGGGTCATCATACAGAATTCACTCGAATCGGATATACCAAGGGTAGTATAATTAACTCGTTGATTCGATCTTTTCTCTCTTTTC

127_20180828_01_01_ndhCret-trnV-comp AATACCAAAATCGGAATAAGACTTGATATTATCAGAAATGCCCAAAAAATATCATATTCGTAAAGCAGAAACATAGATGCACTCCTATGAACATGGAAAATATACCGGATTAGTCAATCCGACTTGAAATTCTTAAGTCGTTCATAACTGTTTAGTTAAAGTAAGAATTGCTTTTGATCGAACCATCTAGTTTCCTTTCAAGATTAATCAATTAGAATCCATTTTTGTTTTTTTATTTTTTNNNAGTTTATAGTTAAAACTAACTATTGCTCTTATGCAAATTCTCTTGTTTTCATCTCAATCTTACCGAGGATTCTCTCTAAAGAAAAGGGATTCCAAATAGAATTCTCATTTTTTTCTATTTTTTATTAGTTATAGTTAAGTTATTAGGAATTGGTCAAAATTGACATCGATTTGCATTATCTTTTCTATTTTATTATCNNNNNNNNNNNNNNTTTGTTTATATTTATTTCAATTTTTTATGAATATGTCCATTTGCCTCTTTTTTTAGTGGTTTAGAATAGAACAAGTAATAAAACGTCTAGGAATTTTAANNNNNNNNNNNNNNNNNNNTATCAAGTCAAGATTGATTTATAGTATAGAATTGGTATATTNNNNNNNNNNNNNNNNNNNNCCTTTTATTTATTATTTATACCTTTTATTTATTNNNNNNNNNNNNATTTAATTAGAATCCAATCTCGAGGGAGGATTTTCTATCGAAAGAAAAGAGAAGACTAGGTCTAAGTAAGGTATACGAAGGAAAGCCCCTTTTACGTTGTTGACAATGTTTACAATGAAACTTAAATTAGCAAATTGGTTTTTAAAGCTTTATCTTGTGCACAACTACCGCACAATACCTGCGTAAATTACTATTAGATTTGATTGGGGTTGGGTTTTTTTAACCAGACTCGGGTCATCATACAGAATTCACTCGAATCGGATATACCAAGGGTAGTATAATTAACTCGTTGATTCGATCTTTTCTCTCTTTTC

128_20180828_01_02_ndhCret-trnV-comp AATACCAAAATCGGAATAAGACTTGATATTATCAGAAATGCCCAAAAAATATCATATTCGTAAAGCAGAAACATAGATGCACTCCTATGAACATGGAAAATATACCGGATTAGTCAATCCGACTTGAAATTCTTAAGTCGTTCATAACTGTTTAGTTAAAGTAAGAATTGCTTTTGATCGAACCATCTAGTTTCCTTTCAAGATTAATCAATTAGAATCCATTTTTGTTTTTTTATTTTTTNNNAGTTTATAGTTAAAACTAACTATTGCTCTTATGCAAATTCTCTTGTTTTCATCTCAATCTTACCGAGGATTCTCTCTAAAGAAAAGGGATTCCAAATAGAATTCTCATTTTTTTCTATTTTTTATTAGTTATAGTTAAGTTATTAGGAATTGGTCAAAATTGACATCGATTTGCATTATCTTTTCTATTTTATTATCNNNNNNNNNNNNNNTTTGTTTATATTTATTTCAATTTTTTATGAATATGTCCATTTGCCTCTTTTTTTAGTGGTTTAGAATAGAACAAGTAATAAAACGTCTAGGAATTTTAANNNNNNNNNNNNNNNNNNNTATCAAGTCAAGATTGATTTATAGTATAGAATTGGTATATTNNNNNNNNNNNNNNNNNNNNCCTTTTATTTATTATTTATACCTTTTATTTATTNNNNNNNNNNNNATTTAATTAGAATCCAATCTCGAGGGAGGATTTTCTATCGAAAGAAAAGAGAAGACTAGGTCTAAGTAAGGTATACGAAGGAAAGCCCCTTTTACGTTGTTGACAATGTTTACAATGAAACTTAAATTAGCAAATTGGTTTTTAAAGCTTTATCTTGTGCACAACTACCGCACAATACCTGCGTAAATTACTATTAGATTTGATTGGGGTTGGGTTTTTTTAACCAGACTCGGGTCATCATACAGAATTCACTCGAATCGGATATACCAAGGGTAGTATAATTAACTCGTTGATTCGATCTTTTCTCTCTTTTC

129_20180828_01_03_ndhCret-trnV-comp AATACCAAAATCGGAATAAGACTTGATATTATCAGAAATGCCCAAAAAATATCATATTCGTAAAGCAGAAACATAGATGCACTCCTATGAACATGGAAAATATACCGGATTAGTCAATCCGACTTGAAATTCTTAAGTCGTTCATAACTGTTTAGTTAAAGTAAGAATTGCTTTTGATCGAACCATCTAGTTTCCTTTCAAGATTAATCAATTAGAATCCATTTTTGTTTTTTTATTTTTTNNNAGTTTATAGTTAAAACTAACTATTGCTCTTATGCAAATTCTCTTGTTTTCATCTCAATCTTACCGAGGATTCTCTCTAAAGAAAAGGGATTCCAAATAGAATTCTCATTTTTTTCTATTTTTTATTAGTTATAGTTAAGTTATTAGGAATTGGTCAAAATTGACATCGATTTGCATTATCTTTTCTATTTTATTATCNNNNNNNNNNNNNNTTTGTTTATATTTATTTCAATTTTTTATGAATATGTCCATTTGCCTCTTTTTTTAGTGGTTTAGAATAGAACAAGTAATAAAACGTCTAGGAATTTTAANNNNNNNNNNNNNNNNNNNTATCAAGTCAAGATTGATTTATAGTATAGAATTGGTATATTNNNNNNNNNNNNNNNNNNNNCCTTTTATTTATTATTTATACCTTTTATTTATTNNNNNNNNNNNNATTTAATTAGAATCCAATCTCGAGGGAGGATTTTCTATCGAAAGAAAAGAGAAGACTAGGTCTAAGTAAGGTATACGAAGGAAAGCCCCTTTTACGTTGTTGACAATGTTTACAATGAAACTTAAATTAGCAAATTGGTTTTTAAAGCTTTATCTTGTGCACAACTACCGCACAATACCTGCGTAAATTACTATTAGATTTGATTGGGGTTGGGTTTTTTTAACCAGACTCGGGTCATCATACAGAATTCACTCGAATCGGATATACCAAGGGTAGTATAATTAACTCGTTGATTCGATCTTTTCTCTCTTTTC

130_20180828_02_01_ndhCret-trnV-comp AATACCAAAATCGGAATAAGACTTGATATTATCAGAAATGCCCAAAAAATATCATATTCGTAAAGCAGAAACATAGATGCACTCCTATGAACATGGAAAATATACCGGATTAGTCAATCCGACTTGAAATTCTTAAGTCGTTCATAACTGTTTAGTTAAAGTAAGAATTGCTTTTGATCGAACCATCTAGTTTCCTTTCAAGATTAATCAATTAGAATCCATTTTTGTTTTTTTATTTTTTNNNAGTTTATAGTTAAAACTAACTATTGCTCTTATGCAAATTCTCTTGTTTTCATCTCAATCTTACCGAGGATTCTCTCTAAAGAAAAGGGATTCCAAATAGAATTCTCATTTTTTTCTATTTTTTATTAGTTATAGTTAAGTTATTAGGAATTGGTCAAAATTGACATCGATTTGCATTATCTTTTCTATTTTATTATCNNNNNNNNNNNNNNTTTGTTTATATTTATTTCAATTTTTTATGAATATGTCCATTTGCCTCTTTTTTTAGTGGTTTAGAATAGAACAAGTAATAAAACGTCTAGGAATTTTAANNNNNNNNNNNNNNNNNNNTATCAAGTCAAGATTGATTTATAGTATAGAATTGGTATATTNNNNNNNNNNNNNNNNNNNNCCTTTTATTTATTATTTATACCTTTTATTTATTNNNNNNNNNNNNATTTAATTAGAATCCAATCTCGAGGGAGGATTTTCTATCGAAAGAAAAGAGAAGACTAGGTCTAAGTAAGGTATACGAAGGAAAGCCCCTTTTACGTTGTTGACAATGTTTACAATGAAACTTAAATTAGCAAATTGGTTTTTAAAGCTTTATCTTGTGCACAACTACCGCACAATACCTGCGTAAATTACTATTAGATTTGATTGGGGTTGGGTTTTTTTAACCAGACTCGGGTCATCATACAGAATTCACTCGAATCGGATATACCAAGGGTAGTATAATTAACTCGTTGATTCGATCTTTTCTCTCTTTTC

131_20180828_02_02_ndhCret-trnV-comp AATACCAAAATCGGAATAAGACTTGATATTATCAGAAATGCCCAAAAAATATCATATTCGTAAAGCAGAAACATAGATGCACTCCTATGAACATGGAAAATATACCGGATTAGTCAATCCGACTTGAAATTCTTAAGTCGTTCATAACTGTTTAGTTAAAGTAAGAATTGCTTTTGATCGAACCATCTAGTTTCCTTTCAAGATTAATCAATTAGAATCCATTTTTGTTTTTTTATTTTTTNNNAGTTTATAGTTAAAACTAACTATTGCTCTTATGCAAATTCTCTTGTTTTCATCTCAATCTTACCGAGGATTCTCTCTAAAGAAAAGGGATTCCAAATAGAATTCTCATTTTTTTCTATTTTTTATTAGTTATAGTTAAGTTATTAGGAATTGGTCAAAATTGACATCGATTTGCATTATCTTTTCTATTTTATTATCNNNNNNNNNNNNNNTTTGTTTATATTTATTTCAATTTTTTATGAATATGTCCATTTGCCTCTTTTTTTAGTGGTTTAGAATAGAACAAGTAATAAAACGTCTAGGAATTTTAANNNNNNNNNNNNNNNNNNNTATCAAGTCAAGATTGATTTATAGTATAGAATTGGTATATTNNNNNNNNNNNNNNNNNNNNCCTTTTATTTATTATTTATACCTTTTATTTATTNNNNNNNNNNNNATTTAATTAGAATCCAATCTCGAGGGAGGATTTTCTATCGAAAGAAAAGAGAAGACTAGGTCTAAGTAAGGTATACGAAGGAAAGCCCCTTTTACGTTGTTGACAATGTTTACAATGAAACTTAAATTAGCAAATTGGTTTTTAAAGCTTTATCTTGTGCACAACTACCGCACAATACCTGCGTAAATTACTATTAGATTTGATTGGGGTTGGGTTTTTTTAACCAGACTCGGGTCATCATACAGAATTCACTCGAATCGGATATACCAAGGGTAGTATAATTAACTCGTTGATTCGATCTTTTCTCTCTTTTC

132_20180828_02_03_ndhCret-trnV-comp AATACCAAAATCGGAATAAGACTTGATATTATCAGAAATGCCCAAAAAATATCATATTCGTAAAGCAGAAACATAGATGCACTCCTATGAACATGGAAAATATACCGGATTAGTCAATCCGACTTGAAATTCTTAAGTCGTTCATAACTGTTTAGTTAAAGTAAGAATTGCTTTTGATCGAACCATCTAGTTTCCTTTCAAGATTAATCAATTAGAATCCATTTTTGTTTTTTTATTTTTTNNNAGTTTATAGTTAAAACTAACTATTGCTCTTATGCAAATTCTCTTGTTTTCATCTCAATCTTACCGAGGATTCTCTCTAAAGAAAAGGGATTCCAAATAGAATTCTCATTTTTTTCTATTTTTTATTAGTTATAGTTAAGTTATTAGGAATTGGTCAAAATTGACATCGATTTGCATTATCTTTTCTATTTTATTATCNNNNNNNNNNNNNNTTTGTTTATATTTATTTCAATTTTTTATGAATATGTCCATTTGCCTCTTTTTTTAGTGGTTTAGAATAGAACAAGTAATAAAACGTCTAGGAATTTTAANNNNNNNNNNNNNNNNNNNTATCAAGTCAAGATTGATTTATAGTATAGAATTGGTATATTNNNNNNNNNNNNNNNNNNNNCCTTTTATTTATTATTTATACCTTTTATTTATTNNNNNNNNNNNNATTTAATTAGAATCCAATCTCGAGGGAGGATTTTCTATCGAAAGAAAAGAGAAGACTAGGTCTAAGTAAGGTATACGAAGGAAAGCCCCTTTTACGTTGTTGACAATGTTTACAATGAAACTTAAATTAGCAAATTGGTTTTTAAAGCTTTATCTTGTGCACAACTACCGCACAATACCTGCGTAAATTACTATTAGATTTGATTGGGGTTGGGTTTTTTTAACCAGACTCGGGTCATCATACAGAATTCACTCGAATCGGATATACCAAGGGTAGTATAATTAACTCGTTGATTCGATCTTTTCTCTCTTTTC

133_20190626_01_01_ndhCret-trnV-comp AATACCAAAATCGGAATAAGACTTGATATTATCAGAAATGCCCAAAAAATATCATATTCGTAAAGCAGAAACATAGATGCACTCCTATGAACATGGAAAATATACCGGATTAGTCAATCCGACTTGAAATTCTTAAGTCGTTCATAACTGTTTAGTTAAAGTAAGAATTGCTTTTGATCGAACCATCTAGTTTCCTTTCAAGATTAATCAATTAGAATCCATTTTTGTTTTTTTATTTTTTNNNAGTTTATAGTTAAAACTAACTATTGCTCTTATGCAAATTCTCTTGTTTTCATCTCAATCTTACCGAGGATTCTCTCTAAAGAAAAGGGATTCCAAATAGAATTCTCATTTTTTTCTATTTTTTATTAGTTATAGTTAAGTTATTAGGAATTGGTCAAAATTGACATCGATTTGCATTATCTTTTCTATTTTATTATCNNNNNNNNNNNNNNTTTGTTTATATTTATTTCAATTTTTTATGAATATGTCCATTTGCCTCTTTTTTTAGTGGTTTAGAATAGAACAAGTAATAAAACGTCTAGGAATTTTAANNNNNNNNNNNNNNNNNNNTATCAAGTCAAGATTGATTTATAGTATAGAATTGGTATATTNNNNNNNNNNNNNNNNNNNNCCTTTTATTTATTATTTATACCTTTTATTTATTNNNNNNNNNNNNATTTAATTAGAATCCAATCTCGAGGGAGGATTTTCTATCGAAAGAAAAGAGAAGACTAGGTCTAAGTAAGGTATACGAAGGAAAGCCCCTTTTACGTTGTTGACAATGTTTACAATGAAACTTAAATTAGCAAATTGGTTTTTAAAGCTTTATCTTGTGCACAACTACCGCACAATACCTGCGTAAATTACTATTAGATTTGATTGGGGTTGGGTTTTTTTAACCAGACTCGGGTCATCATACAGAATTCACTCGAATCGGATATACCAAGGGTAGTATAATTAACTCGTTGATTCGATCTTTTCTCTCTTTTC

134_20190626_01_02_ndhCret-trnV-comp AATACCAAAATCGGAATAAGACTTGATATTATCAGAAATGCCCAAAAAATATCATATTCGTAAAGCAGAAACATAGATGCACTCCTATGAACATGGAAAATATACCGGATTAGTCAATCCGACTTGAAATTCTTAAGTCGTTCATAACTGTTTAGTTAAAGTAAGAATTGCTTTTGATCGAACCATCTAGTTTCCTTTCAAGATTAATCAATTAGAATCCATTTTTGTTTTTTTATTTTTTNNNAGTTTATAGTTAAAACTAACTATTGCTCTTATGCAAATTCTCTTGTTTTCATCTCAATCTTACCGAGGATTCTCTCTAAAGAAAAGGGATTCCAAATAGAATTCTCATTTTTTTCTATTTTTTATTAGTTATAGTTAAGTTATTAGGAATTGGTCAAAATTGACATCGATTTGCATTATCTTTTCTATTTTATTATCNNNNNNNNNNNNNNTTTGTTTATATTTATTTCAATTTTTTATGAATATGTCCATTTGCCTCTTTTTTTAGTGGTTTAGAATAGAACAAGTAATAAAACGTCTAGGAATTTTAANNNNNNNNNNNNNNNNNNNTATCAAGTCAAGATTGATTTATAGTATAGAATTGGTATATTNNNNNNNNNNNNNNNNNNNNCCTTTTATTTATTATTTATACCTTTTATTTATTNNNNNNNNNNNNATTTAATTAGAATCCAATCTCGAGGGAGGATTTTCTATCGAAAGAAAAGAGAAGACTAGGTCTAAGTAAGGTATACGAAGGAAAGCCCCTTTTACGTTGTTGACAATGTTTACAATGAAACTTAAATTAGCAAATTGGTTTTTAAAGCTTTATCTTGTGCACAACTACCGCACAATACCTGCGTAAATTACTATTAGATTTGATTGGGGTTGGGTTTTTTTAACCAGACTCGGGTCATCATACAGAATTCACTCGAATCGGATATACCAAGGGTAGTATAATTAACTCGTTGATTCGATCTTTTCTCTCTTTTC

135_20190626_02_01_ndhCret-trnV-comp AATACCAAAATCGGAATAAGACTTGATATTATCAGAAATGCCCAAAAAATATCATATTCGTAAAGCAGAAACATAGATGCACTCCTATGAACATGGAAAATATACCGGATTAGTCAATCCGACTTGAAATTCTTAAGTCGTTCATAACTGTTTAGTTAAAGTAAGAATTGCTTTTGATCGAACCATCTAGTTTCCTTTCAAGATTAATCAATTAGAATCCATTTTTGTTTTTTTATTTTTTNNNAGTTTATAGTTAAAACTAACTATTGCTCTTATGCAAATTCTCTTGTTTTCATCTCAATCTTACCGAGGATTCTCTCTAAAGAAAAGGGATTCCAAATAGAATTCTCATTTTTTTCTATTTTTTATTAGTTATAGTTAAGTTATTAGGAATTGGTCAAAATTGACATCGATTTGCATTATCTTTTCTATTTTATTATCNNNNNNNNNNNNNNTTTGTTTATATTTATTTCAATTTTTTATGAATATGTCCATTTGCCTCTTTTTTTAGTGGTTTAGAATAGAACAAGTAATAAAACGTCTAGGAATTTTAANNNNNNNNNNNNNNNNNNNTATCAAGTCAAGATTGATTTATAGTATAGAATTGGTATATTNNNNNNNNNNNNNNNNNNNNCCTTTTATTTATTATTTATACCTTTTATTTATTNNNNNNNNNNNNATTTAATTAGAATCCAATCTCGAGGGAGGATTTTCTATCGAAAGAAAAGAGAAGACTAGGTCTAAGTAAGGTATACGAAGGAAAGCCCCTTTTACGTTGTTGACAATGTTTACAATGAAACTTAAATTAGCAAATTGGTTTTTAAAGCTTTATCTTGTGCACAACTACCGCACAATACCTGCGTAAATTACTATTAGATTTGATTGGGGTTGGGTTTTTTTAACCAGACTCGGGTCATCATACAGAATTCACTCGAATCGGATATACCAAGGGTAGTATAATTAACTCGTTGATTCGATCTTTTCTCTCTTTTC

136_20190626_03_01_ndhCret-trnV-comp AATACCAAAATCGGAATAAGACTTGATATTATCAGAAATGCCCAAAAAATATCATATTCGTAAAGCAGAAACATAGATGCACTCCTATGAACATGGAAAATATACCGGATTAGTCAATCCGACTTGAAATTCTTAAGTCGTTCATAACTGTTTAGTTAAAGTAAGAATTGCTTTTGATCGAACCATCTAGTTTCCTTTCAAGATTAATCAATTAGAATCCATTTTTGTTTTTTTATTTTTTTTTAGTTTTTAGTTAAAACTAACTATTGCTCTTATGCAAATTCTCTTGTTTTCATCTCAATCTTACCGAGGATTCTCTCTAAAGAAAAGGGATTCCAAATAGAATTCTCATTTTTTTCTATTTTTTATTAGTTATAGTTAAGTTATTAGGAATTGGTCAAAATTGACATCGATTTGCATTATCTTTTCTATTTTATTATCNNNNNNNNNNNNNNTTTGTTTATATTTATTTCAATTTTTTATGAATATGTCCATTTGCCTCTTTTTTTAGTGGTTTAGAATAGAACAAGTAATAAAACGTCTAGGAATTTTAANNNNNNNNNNNNNNNNNNNTATCAAGTCAAGATTGATTTATAGTATAGAATTGGTATATTNNNNNNNNNNNNNNNNNNNNCCTTTTATTTATTATTTATACCTTTTATTTATTNNNNNNNNNNNNATTTAATTAGAATCCAATCTCGAGGGAGGATTTTCTATCGAAAGAAAAGAGAAGACTAGGTCTAAGTAAGGTATACGAAGGAAAGCCCCTTTTACGTTGTTGACAATGTTTACAATGAAACTTAAATTAGCAAATTGGTTTTTAAAGCTTTATCTTGTGCACAACTACCGCACAATACCTGCGTAAATTACTATTAGATTTGATTGGGGTTGGGTTTTTTTAACCAGACTCGGGTCATCATACAGAATTCACTCGAATCGGATATACCAAGGGTAGTATAATTAACTCGTTGATTCGATCTTTTCTCTCCTTTC

137_20190626_05_04_ndhCret-trnV-comp AATACCAAAATCGGAATAAGACTTGATATTATCAGAAATGCCCAAAAAATATCATATTCGTAAAGCAGAAACATAGATGCACTCCTATGAACATGGAAAATATACCGGATTAGTCAATCCGACTTGAAATTCTTAAGTCGTTCATAACTGTTTAGTTAAAGTAAGAATTGCTTTTGATCGAACCATCTAGTTTCCTTTCAAGATTAATCAATTAGAATCCATTTTTGTTTTTTTATTTTTTTTTAGTTTTTAGTTAAAACTAACTATTGCTCTTATGCAAATTCTCTTGTTTTCATCTCAATCTTACCGAGGATTCTCTCTAAAGAAAAGGGATTCCAAATAGAATTCTCATTTTTTTCTATTTTTTATTAGTTATAGTTAAGTTATTAGGAATTGGTCAAAATTGACATCGATTTGCATTATCTTTTCTATTTTATTATCNNNNNNNNNNNNNNTTTGTTTATATTTATTTCAATTTTTTATGAATATGTCCATTTGCCTCTTTTTTTAGTGGTTTAGAATAGAACAAGTAATAAAACGTCTAGGAATTTTAANNNNNNNNNNNNNNNNNNNTATCAAGTCAAGATTGATTTATAGTATAGAATTGGTATATTNNNNNNNNNNNNNNNNNNNNCCTTTTATTTATTATTTATACCTTTTATTTATTNNNNNNNNNNNNATTTAATTAGAATCCAATCTCGAGGGAGGATTTTCTATCGAAAGAAAAGAGAAGACTAGGTCTAAGTAAGGTATACGAAGGAAAGCCCCTTTTACGTTGTTGACAATGTTTACAATGAAACTTAAATTAGCAAATTGGTTTTTAAAGCTTTATCTTGTGCACAACTACCGCACAATACCTGCGTAAATTACTATTAGATTTGATTGGGGTTGGGTTTTTTTAACCAGACTCGGGTCATCATACAGAATTCACTCGAATCGGATATACCAAGGGTAGTATAATTAACTCGTTGATTCGATCTTTTCTCTCCTTTC

138_20190626_07_01_ndhCret-trnV-comp AATACCAAAATCGGAATAAGACTTGATATTATCAGAAATGCCCAAAAAATATCATATTCGTAAAGCAGAAACATAGATGCACTCCTATGAACATGGAAAATATACCGGATTAGTCAATCCGACTTGAAATTCTTAAGTCGTTCATAACTGTTTAGTTAAAGTAAGAATTGCTTTTGATCGAACCATCTAGTTTCCTTTCAAGATTAATCAATTAGAATCCATTTTTGTTTTTTTATTTTTTTTTAGTTTTTAGTTAAAACTAACTATTGCTCTTATGCAAATTCTCTTGTTTTCATCTCAATCTTACCGAGGATTCTCTCTAAATAAAAGGGATTCCAAATAGAATTCTCATTTTTTTCTATTTTTTATTAGTTATAGTTAAGTTATTAGGAATTGGTCAAAATTGACATCGATTTGCATTATCTTTTCTATTTTATTATCNNNNNNNNNNNNNNTTTGTTTATATTTATTTCAATTTTTTATGAATATGTCCATTTGCCTCTTTTTTTAGTGGTTTAGAATAGAACAAGTAATAAAACGTCTAGGAATTTTAANNNNNNNNNNNNNNNNNNNTATCAAGTCAAGATTGATTTATAGTATAGAATTGGTATATTNNNNNNNNNNNNNNNNNNNNCCTTTTATTTATTATTTATACCTTTTATTTATTNNNNNNNNNNNNATTTAATTAGAATCCAATCTCGAGGGAGGATTTTCTATCGAAAGAAAAGAGAAGACTAGGTCTAAGTAAGGTATACGAAGGAAAGCCCCTTTTACGTTGTTGACAATGTTTACAATGAAACTTAAATTAGCAAATTGGTTTTTAAAGCTTTATCTTGTGCACAACTACCGCACAATACCTGCGTAAATTACTATTAGATTTGATTGGGGTTGGGTTTTTTTAACCAGACTCGGGTCATCATACAGAATTCACTCGAATCGGATATACCAAGGGTAGTATAATTAACTCGTTGATTCGATCTTTTCTCTCCTTTC

139_20190626_07_02_ndhCret-trnV-comp AATACCAAAATCGGAATAAGACTTGATATTATCAGAAATGCCCAAAAAATATCATATTCGTAAAGCAGAAACATAGATGCACTCCTATGAACATGGAAAATATACCGGATTAGTCAATCCGACTTGAAATTCTTAAGTCGTTCATAACTGTTTAGTTAAAGTAAGAATTGCTTTTGATCGAACCATCTAGTTTCCTTTCAAGATTAATCAATTAGAATCCATTTTTGTTTTTTTATTTTTTNNNAGTTTATAGTTAAAACTAACTATTGCTCTTATGCAAATTCTCTTGTTTTCATCTCAATCTTACCGAGGATTCTCTCTAAAGAAAAGGGATTCCAAATAGAATTCTCATTTTTTTCTATTTTTTATTAGTTATAGTTAAGTTATTAGGAATTGGTCAAAATTGACATCGATTTGCATTATCTTTTCTATTTTATTATCNNNNNNNNNNNNNNTTTGTTTATATTTATTTCAATTTTTTATGAATATGTCCATTTGCCTCTTTTTTTAGTGGTTTAGAATAGAACAAGTAATAAAACGTCTAGGAATTTTAANNNNNNNNNNNNNNNNNNNTATCAAGTCAAGATTGATTTATAGTATAGAATTGGTATATTNNNNNNNNNNNNNNNNNNNNCCTTTTATTTATTATTTATACCTTTTATTTATTNNNNNNNNNNNNATTTAATTAGAATCCAATCTCGAGGGAGGATTTTCTATCGAAAGAAAAGAGAAGACTAGGTCTAAGTAAGGTATACGAAGGAAAGCCCCTTTTACGTTGTTGACAATGTTTACAATGAAACTTAAATTAGCAAATTGGTTTTTAAAGCTTTATCTTGTGCACAACTACCGCACAATACCTGCGTAAATTACTATTAGATTTGATTGGGGTTGGGTTTTTTTAACCAGACTCGGGTCATCATACAGAATTCACTCGAATCGGATATACCAAGGGTAGTATAATTAACTCGTTGATTCGATCTTTTCTCTCTTTTC

140_20190626_08_01_ndhCret-trnV-comp AATACCAAAATCGGAATAAGACTTGATATTATCAGAAATGCCCAAAAAATATCATATTCGTAAAGCAGAAACATAGATGCACTCCTATGAACATGGAAAATATACCGGATTAGTCAATCCGACTTGAAATTCTTAAGTCGTTCATAACTGTTTAGTTAAAGTAAGAATTGCTTTTGATCGAACCATCTAGTTTCCTTTCAAGATTAATCAATTAGAATCCATTTTTGTTTTTTTATTTTTTNNNAGTTTATAGTTAAAACTAACTATTGCTCTTATGCAAATTCTCTTGTTTTCATCTCAATCTTACCGAGGATTCTCTCTAAAGAAAAGGGATTCCAAATAGAATTCTCATTTTTTTCTATTTTTTATTAGTTATAGTTAAGTTATTAGGAATTGGTCAAAATTGACATCGATTTGCATTATCTTTTCTATTTTATTATCNNNNNNNNNNNNNNTTTGTTTATATTTATTTCAATTTTTTATGAATATGTCCATTTGCCTCTTTTTTTAGTGGTTTAGAATAGAACAAGTAATAAAACGTCTAGGAATTTTAANNNNNNNNNNNNNNNNNNNTATCAAGTCAAGATTGATTTATAGTATAGAATTGGTATATTNNNNNNNNNNNNNNNNNNNNCCTTTTATTTATTATTTATACCTTTTATTTATTNNNNNNNNNNNNATTTAATTAGAATCCAATCTCGAGGGAGGATTTTCTATCGAAAGAAAAGAGAAGACTAGGTCTAAGTAAGGTATACGAAGGAAAGCCCCTTTTACGTTGTTGACAATGTTTACAATGAAACTTAAATTAGCAAATTGGTTTTTAAAGCTTTATCTTGTGCACAACTACCGCACAATACCTGCGTAAATTACTATTAGATTTGATTGGGGTTGGGTTTTTTTAACCAGACTCGGGTCATCATACAGAATTCACTCGAATCGGATATACCAAGGGTAGTATAATTAACTCGTTGATTCGATCTTTTCTCTCTTTTC

141_20190626_09_02_ndhCret-trnV-comp AATACCAAAATCGGAATAAGACTTGATATTATCAGAAATGCCCAAAAAATATCATATTCGTAAAGCAGAAACATAGATGCACTCCTATGAACATGGAAAATATACCGGATTAGTCAATCCGACTTGAAATTCTTAAGTCGTTCATAACTGTTTAGTTAAAGTAAGAATTGCTTTTGATCGAACCATCTAGTTTCCTTTCAAGATTAATCAATTAGAATCCATTTTTGTTTTTTTATTTTTTNNNAGTTTATAGTTAAAACTAACTATTGCTCTTATGCAAATTCTCTTGTTTTCATCTCAATCTTACCGAGGATTCTCTCTAAAGAAAAGGGATTCCAAATAGAATTCTCATTTTTTTCTATTTTTTATTAGTTATAGTTAAGTTATTAGGAATTGGTCAAAATTGACATCGATTTGCATTATCTTTTCTATTTTATTATCNNNNNNNNNNNNNNTTTGTTTATATTTATTTCAATTTTTTATGAATATGTCCATTTGCCTCTTTTTTTAGTGGTTTAGAATAGAACAAGTAATAAAACGTCTAGGAATTTTAANNNNNNNNNNNNNNNNNNNTATCAAGTCAAGATTGATTTATAGTATAGAATTGGTATATTNNNNNNNNNNNNNNNNNNNNCCTTTTATTTATTATTTATACCTTTTATTTATTNNNNNNNNNNNNATTTAATTAGAATCCAATCTCGAGGGAGGATTTTCTATCGAAAGAAAAGAGAAGACTAGGTCTAAGTAAGGTATACGAAGGAAAGCCCCTTTTACGTTGTTGACAATGTTTACAATGAAACTTAAATTAGCAAATTGGTTTTTAAAGCTTTATCTTGTGCACAACTACCGCACAATACCTGCGTAAATTACTATTAGATTTGATTGGGGTTGGGTTTTTTTAACCAGACTCGGGTCATCATACAGAATTCACTCGAATCGGATATACCAAGGGTAGTATAATTAACTCGTTGATTCGATCTTTTCTCTCTTTTC

142_20190627_01_01_ndhCret-trnV-comp AATACCAAAATCGGAATAAGACTTGATATTATCAGAAATGCCCAAAAAATATCATATTCGTAAAGCAGAAACATAGATGCACTCCTATGAACATGGAAAATATACCGGATTAGTCAATCCGACTTGAAATTCTTAAGTCGTTCATAACTGTTTAGTTAAAGTAAGAATTGCTTTTGATCGAACCATCTAGTTTCCTTTCAAGATTAATCAATTAGAATCCATTTTTGTTTTTTTATTTTTTNNNAGTTTATAGTTAAAACTAACTATTGCTCTTATGCAAATTCTCTTGTTTTCATCTCAATCTTACCGAGGATTCTCTCTAAAGAAAAGGGATTCCAAATAGAATTCTCATTTTTTTCTATTTTTTATTAGTTATAGTTAAGTTATTAGGAATTGGTCAAAATTGACATCGATTTGCATTATCTTTTCTATTTTATTATCNNNNNNNNNNNNNNTTTGTTTATATTTATTTCAATTTTTTATGAATATGTCCATTTGCCTCTTTTTTTAGTGGTTTAGAATAGAACAAGTAATAAAACGTCTAGGAATTTTAANNNNNNNNNNNNNNNNNNNTATCAAGTCAAGATTGATTTATAGTATAGAATTGGTATATTNNNNNNNNNNNNNNNNNNNNCCTTTTATTTATTATTTATACCTTTTATTTATTNNNNNNNNNNNNATTTAATTAGAATCCAATCTCGAGGGAGGATTTTCTATCGAAAGAAAAGAGAAGACTAGGTCTAAGTAAGGTATACGAAGGAAAGCCCCTTTTACGTTGTTGACAATGTTTACAATGAAACTTAAATTAGCAAATTGGTTTTTAAAGCTTTATCTTGTGCACAACTACCGCACAATACCTGCGTAAATTACTATTAGATTTGATTGGGGTTGGGTTTTTTTAACCAGACTCGGGTCATCATACAGAATTCACTCGAATCGGATATACCAAGGGTAGTATAATTAACTCGTTGATTCGATCTTTTCTCTCTTTTC
[truncated: 245,605 more chars]
